# A high throughput computational investigation of the solid solution mechanisms of actinides and lanthanides in zirconolite†

**DOI:** 10.1039/d1ra02914b

**Published:** 2021-07-20

**Authors:** Stavrina Dimosthenous, Christopher M. Handley, Lewis R. Blackburn, Colin L. Freeman, Neil C. Hyatt

**Affiliations:** Department of Materials Science and Engineering, The University of Sheffield Mappin Street Sheffield UK stavrinadimosthenous@gmail.com n.c.hyatt@sheffield.ac.uk; The Digital Research Service, The School of Computing, Jubilee Campus, The University of Nottingham Nottingham UK

## Abstract

In this work, we perform a theoretical investigation of the actinide and lanthanide solid solution mechanisms of zirconolite-2M, prototypically CaZrTi_2_O_7_, as a candidate immobilisation matrix for plutonium. Solid solution energies were calculated using static atomistic simulations by means of the General Utility Lattice Program, for formulations of relevance to ceramic wasteform deployment, with substitution on the Ca^2+^ and Zr^4+^ sites by Ce^4+^, Pu^4+^, Th^4+^, and U^4+^, and appropriate charge balance by substitution of Al^3+^ or Fe^3+^ on Ti^4+^ sites. In simple solid solutions involving substitution on the Zr^4+^ site, we found that whereas substitution of Ce^4+^, U^4+^ and Pu^4+^ were energetically favoured, substitution of Th^4+^ was not energetically favoured. For more complex solid solutions involving Ce^4+^, Pu^4+^, Th^4+^, and U^4+^ substitution on the Ca^2+^ site, we found the most energetically favoured scheme involved co-substitution of Al^3+^ or Fe^3+^ on the five-fold co-ordinate Ti^4+^ site in the zirconolite-2M structure. Comparison of these computational data with experimental evidence, where available, demonstrated broad agreement. Consequently, this study provides useful insight into formulation design and the efficacy of Ce^4+^, U^4+^ and Th^4+^ as Pu^4+^ surrogates in zirconolite-2M ceramic wasteforms for plutonium disposition.

## Abstract 2

Computer simulations identify the energetics of different defects in the zirconolite system highlighting the different chemistries and sizes.

## Graphical abstract

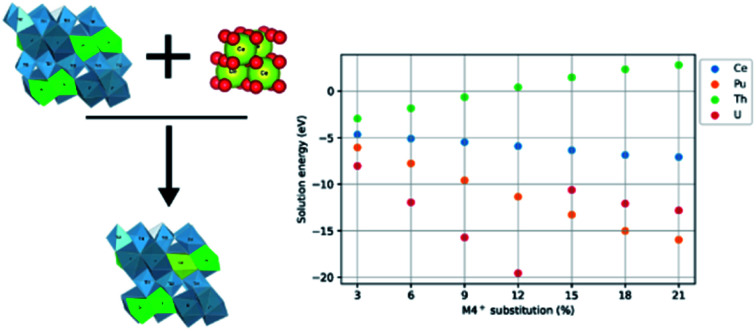

## 1 Introduction

The UK holds the world's largest inventory of separated civil plutonium, forecast to reach 140 tons at the end of reprocessing operations.^1^ UK government policy is to manage this material to a safe and secure end point, the preferred strategy for which is reuse in mixed oxide (MOX) fuel in light water reactors. However, should this strategy not prove implementable, immobilisation of the inventory will be required, along with the fraction of material known to be unsuitable for MOX fuel manufacture.

Numerous natural and synthetic materials have been proposed as wasteforms for the immobilisation of actinides, these including ceramics, glasses, and glass-ceramics.^2–6^ Geological disposal of actinides places greater emphasis on the performance of the wasteform and near field barriers, so as to assure adequate containment of fissile material over the required timescales, which, in the geological context, are comparably short.^2–5,7^ Zirconolite, prototypically CaZrTi_2_O_7_, is a naturally occurring mineral and the dominant actinide bearing phase in the SYNROC C ceramic wasteform;^8,9^ it is known to be highly resistant to alteration and dissolution.^10,11^ As a result, zirconolite is an attractive material for plutonium immobilisation and its potential as a wasteform has been well established.^8,12–15^ The zirconolite-2M polytype structure (space group *C*2/*c*), adopted by the prototypical composition CaZrTi_2_O_7_, comprises alternating layers of CaO_8_ and ZrO_7_ polyhedra aligned parallel to (110); parallel to the [001] direction, these polyhedra are interleaved 1 : 1 with hexagonal tungsten bronze motifs formed by corner sharing TiO_6_ and TiO_5_ polyhedra.^16,17^ The 2M nomenclature thus signifies a monoclinic unit cell with a two layer repeat sequence along [001]; other zirconolite polytype structures with different interlayer relationships are known, as discussed below.

The use of Pu in laboratory based studies is hazardous, challenging and expensive. Consequently, Ce, U and Th are frequently used as inactive or low active surrogates to emulate the behaviour of Pu in laboratory based studies.^18,19^ This is due to the similarity of the ionic size of Ce^4+^, U^4+^, Th^4+^ and Pu^4+^ and to CeO_2_, PuO_2_, ThO_2_, and UO_2_ all having a common fluorite crystal structure and exhibiting solid solution at any ratio, implying similar solid state chemistry.^20–23^

In this work we aim to investigate the plutonium immobilisation potential of zirconolite-2M by atomistic simulations. Previous simulation based studies of zirconolite-2M at the atomistic level have focused on studying the defect chemistry of actinide additions,^24^ and within the regime of molecular dynamics for investigations the radiation damaged structure, and crystalline to amorphous phase transition, arising from α-decay of Pu.^25–27^ More recently, Ce and actinide solid solution mechanisms in zirconolite-2M were studied at the electronic structure level, within the density functional theory (DFT) regime.^28^ Importantly, the DFT investigation of Tanti *et al.* broadly agreed with findings of the atomistic simulations of Gilbert *et al.* for Ce-substituted zirconolite-2M. The choice of methodology and accuracy level is a critical consideration in such investigations. The broad agreement between DFT and atomistic simulations shows that we can obtain accurate insight with the atomistic approach. Further, given the low computational cost, it is feasible to simulate relatively large lattices at the atomistic level employing an innovative high-throughput workflow described herein.

Our investigation develops and extends a previous computational study of the defect chemistry of zirconolite-2M,^24^ with regard to incorporation of Ce^3+/4+^ and Pu^3+/4+^. In this contribution we expand the previous study by examining the solid solution of Pu^4+^ and its typical surrogates, Ce^4+^, U^4+^, Th^4+^, on the Ca^2+^ and Zr^4+^ sites, at concentrations greater than point defects, with necessary charge compensation provided by Al^3+^ and Fe^3+^ substitution on the Ti^4+^ sites.

We focus on the Pu^4+^ oxidation state which has been shown to be the dominant species in fluorite related zirconolite-2M and pyrochlore structured ceramics synthesised under conditions relevant to wasteform manufacture.^29–33^ Under conditions of hot isostatic pressing with PuO_2_ as a feedstock, synthesis of the zirconolite ceramic wasteform will be under the redox control of the Fe/FeO buffer imposed by the stainless steel can. Consideration of Ellingham diagrams shows that this will not be sufficient to effect PuO_2_/Pu_2_O_3_ reduction.^34^ Indeed, Pu^3+^ is stabilised by annealing only under strongly reducing 5% H_2_/N_2_, or 5% H_2_/Ar, which is not relevant to the technological focus of wasteform manufacture by hot isostatic pressing.^31,32^

## 2 Theory

The work presented in this paper examines three substitution schemes to investigate Pu and surrogate incorporation in zirconolite-2M. The substitution schemes are based on compositions relevant to optimisation of zirconolite ceramic formulations. We used the *supercell* approach to study the defects of the system. Here, the defects are added as absolute concentrations in a solid solution, and our concentration values can therefore be directly compared to experimental compositions. This differs from previous work on this system^24^ that used a Mott–Littleton method where the substitution defects were effectively at infinite dilution.

In the first substitution scheme, we replaced Zr^4+^ sites in prototypical zirconolite-2M with Ce^4+^, Pu^4+^, Th^4+^ and U^4+^. The chemical reaction for the substitution scheme was as follows,CaZrTi_2_O_7_ + *x*MO_2_ → CaZr_1−*x*_M_*x*_Ti_2_O_7_ + *x*ZrO_2_where M = Ce, Pu, Th, U. Here, 2M denotes the polytype structure of monoclinic symmetry.

The second substitution scheme targeted the substitution of Ce^4+^, Pu^4+^, Th^4+^ and U^4+^ on the Ca^2+^ site, with charge balance provided by replacing 2Ti^4+^ sites with Al^3+^, for every Ca^2+^ ion replaced.^35–37^ The second substitution scheme followed the reaction,CaZrTi_2_O_7_ + *x*MO_2_ + *x*Al_2_O_3_ → Ca_1−*x*_M_*x*_ZrTi_2−2*x*_Al_2*x*_O_7_ + *x*CaO + 2*x*TiO_2_where M = Ce, Pu, Th, U.

**Fig. 1:**
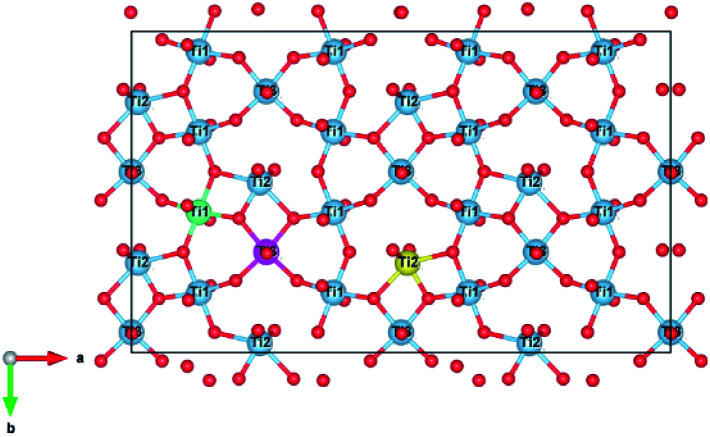
Ti^4+^ site orientation in our base zirconolite-2M system (Ca^2+^, Zr^4+^ hidden). Ti sites are labelled, example Ti(1), Ti(2) and Ti(3) sites are coloured green, yellow and fuchsia, respectively. The Ti(1) and Ti(3) sites have octahedral coordination, whereas the Ti(2) site has trigonal bipyramidal co-ordination and is 50% occupied. Figure generated in VESTA.^40^

There are three unique Ti^4+^ sites in the zirconolite-2M structure that may accommodate charge balancing cations such as Al^3+^. An illustration of the Ti^4+^ site orientations and coordination is shown in Fig. 1, where example Ti(1), Ti(2) and Ti(3) sites are coloured in green, yellow and fuschia respectively, with Ca and Zr omitted for clarity. The Ti(1) and Ti(3) sites adopt octahedral co-ordination by O^2−^, whereas the Ti(2) site adopts a trigonal bipyramidal configuration by O^2−^. The Ti(2) site is partially occupied, with a 50% probability of lying either side of the site axis.^16^

Experimental studies have shown that charge balancing ions are generally preferentially accommodated in the 5-fold coordinate Ti(2) site.^36–38^ Although, in some instances, charge balancing species such as Cr^3+^ have been shown to preferentially adopt 6-fold Ti^4+^ sites as may be expected from consideration of crystal field stabilisation energy.^34^ Therefore, this substitution scheme needs to consider potential preferential substitution of the charge balancing ions for particular Ti^4+^ sites. To address this question, we considered 6 different Al^3+^ site combinations: two Ti(1) sites; two Ti(2) sites; two Ti(3) sites; one Ti(1) and one Ti(2); one Ti(1) and one Ti(3); and one Ti(2) and one Ti(3).

The third substitution scheme was identical to the second scheme, however, the reaction was charge balanced with Fe^3+^.^39^ The third substitution scheme followed the reaction,CaZrTi_2_O_7_ + *x*MO_2_ + *x*Fe_2_O_3_ → Ca_1−*x*_M_*x*_ZrTi_2−2*x*_Fe_2*x*_O_7_ + *x*CaO + 2*x*TiO_2_where M = Ce, Pu, Th, U.

## 3 Method

All the calculations in this work were performed with the General Utility Lattice Program (GULP).^41^ Ions are treated as charged spheres represented by their formal charge^42^ with a coulombic attraction/repulsion. Short range interactions between ions are described with Buckingham potentials of the form,
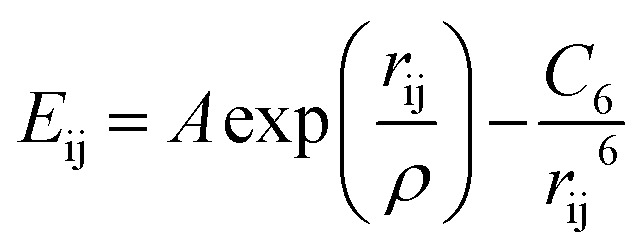
where *r*_ij_ is the distance between two ions i and j, and *A*, *ρ* and *C*_6_ are parametrised constants specific to each interaction pair, as summarised in Table 1.

**Table 1:** Force field parameters for Buckingham potentials used in this work

Interaction	*A* (eV)	*ρ* (Å)	*C*_6_ (eV Å^6^)	Ref.
O^2−^–O^2−^	25.410	0.6937	32.320	48
Ca^2+^–O^2−^	2272.741	0.2986	0.000	48
Zr^4+^–O^2−^	7290.347	0.2610	0.000	49
Ti^4+^–O^2−^	4545.823	0.2610	0.000	49
Al^3+^–O^2−^	2409.505	0.2649	0.000	48
Fe^3+^–O^2−^	3219.335	0.2641	0.000	48
Ce^4+^–O^2−^	2409.505	0.3260	0.000	49
Pu^4+^–O^2−^	752.224	0.4007	0.000	24
Th^4+^–O^2−^	8638.5	0.2856	70.000	50
U^4+^–O^2−^	9296.65	0.2796	90.000	50

The polarisability of the system is described by the shell model^43^ where the charged core interacts with a massless “shell” *via* a spring constant, *k*. Only O^2−^ was polarised in this study so the interaction potentials presented in Table 1 are cation–anion core–shell interaction potentials. The shell model data for O^2−^ are presented in Table 2.

**Table 2:** Shell parameters for O^2−^ used in this work

Effective charge (core/shell)	*k* (eV Å^−2^)	Ref.
0.513/−2.513	20.53	51

The model zirconolite lattice is the stoichiometric 2M polytype taken from the work of Gilbert *et al.*,^24^ based on the structure published by Rossell.^17^ For our simulations the structure was expanded to a 2 × 2 × 2 supercell (704 atoms). The calculations were performed at constant pressure and the structure and atomic positions were optimised using a Broyden–Fletcher–Goldfarb–Shanno (BFGS) algorithm.^44–47^

Substitution sites were randomly chosen. Our base zirconolite-2M system was composed of 704 atoms, 64 of those were Zr^4+^, 64 were Ca^2+^, 128 were Ti^4+^ (64 Ti(1), 32 Ti(2), 32 Ti(3)). For the first solid solution scheme, we chose to produce 30 random site substitution configurations. For the second solid solution scheme we produced 120 random configurations per site combination, *e.g.* CeTi(1)Ti(1). That is, for every substitution introduced to the system we replaced one Ca^2+^ site at random and two of the chosen type of Ti^4+^ sites at random with Al^3+^. For the third solid solution scheme, our aim was to directly compare Al^3+^ and Fe^3+^ as charge balancing species. Therefore, we made a direct substitution of Fe^3+^ on the sites that were occupied by Al^3+^ in the second solid solution scheme. The structures remained identical, otherwise. In our simulations we considered substitution concentrations of 3, 6, 9, 12, 15, 18 and 21%. In practice, this was the percentage of the number of atoms of the element in the cell to be replaced by the substitution rounded to the nearest integer, which was 2, 4, 6, 8, 10, 12 and 13 sites for Zr^4+^ and Ca^2+^, and 4, 8, 12, 20, 24 and 26 sites for Ti^4+^, when considering Al^3+^ and Fe^3+^ charge compensation. For a cell of 704 atoms, there were 64 Zr^4+^ and 64 Ca^2+^ sites that were potential substitution sites. Therefore, we were able to sample a wide range of configurations without artificial symmetry restrictions and provide data that could be compared to experimental investigation. Importantly, the upper substitution limit in this study concentration approximates that required for wasteform deployment.

Once the groundstate energy for each randomly substituted lattice was obtained we calculated the solution energy, that is the enthalpy of solid solution, for each substitution scheme. The calculation was based on the reactions presented in Section 2. We obtained the energy of each component by performing a geometry optimisation calculation on each structure, we assumed the polymorph of TiO_2_ to be rutile.

Configurations that failed to optimise were removed from the spread of data. The solution energies were averaged. For an optimised spread the ground state energy differed by 2 eV to 3 eV. Each point on the graphs presented in Fig. 3 is the average solution energy for the denoted M^4+^ concentration. Each line presented in Fig. 4 and 5 is the trend in the average solution energy over all substitutions for each scheme. Where the trend in the solution energy of a substitution did not follow the global trend in the scheme, it is presented separately on the same graph with a dashed line.

**Fig. 2:**
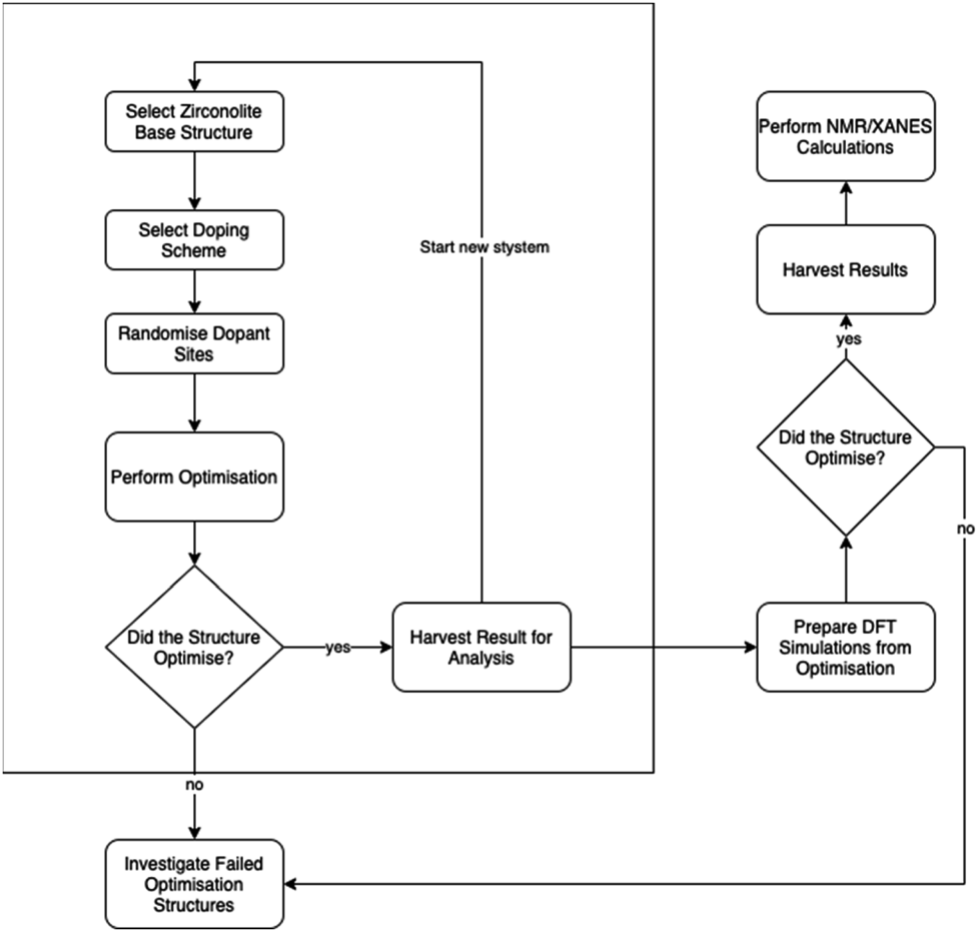
Flowchart demonstrating the high throughput methodology described in the Method section.

To perform the above randomisation of site substitution in structures for each solid solution scheme, we wrote Python based software to enable the automation of the generation of the structures that are then passed to a high-performance computer server, enabling us to rapidly perform simulations and, from the following analysis, provide meaningful suggestions for material synthesis. An example time frame would be about 1–12 hours per simulation on a single core, with simulations queued as an array job; this resulted in about 2 days for the 120 simulation cell analyses from queueing to data clean-up and analysis. The above workflow is illustrated in Fig. 2.

Importantly, Fig. 2 extends the work performed in this study to a pipeline workflow for high-throughput materials discovery. Enclosed within the box is the work performed within this paper. Outside of the box are future steps that can be performed where more expensive computational methods are applied. This demonstrates the method of use computationally cheaper methods – force fields – to scan the search space of crystal structure to quickly provide targets for investigation by more expensive methods. Within the context of the work here, this is beneficial when it is impractical to physically synthesise all possible solid solution stoichiometries. Within the wider context of materials science, our method has application to the exploration of similar materials *e.g.* high entropy alloys, capacitor ceramics, perovskites. Furthermore, the use of this initial screening can then direct subsequent, more costly, *ab initio* simulations, where the initial screening method has narrowed down the range of target compositions. The full calculation outputs are presented in the associated ESI.†

## 4 Results

### 4.1 CaZr_1−*x*_M_*x*_Ti_2_O_7_ (M = Ce, Pu, Th, U)

**Fig. 3:**
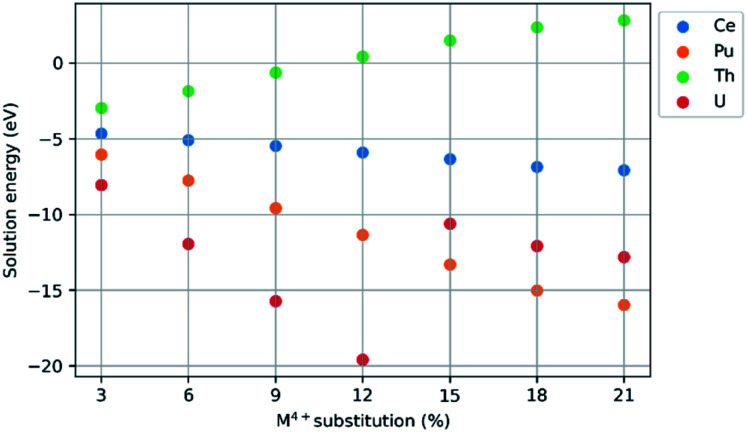
Comparison of solution energy as a function of M^4+^ substitution on the Zr^4+^ site (M = Ce, Pu, Th, U).

Fig. 3 shows the mean solution energy for the following scheme, plotted against Ce, Pu, Th, and U concentration.CaZrTi_2_O_7_ + *x*MO_2_ → CaZr_1−*x*_M_*x*_Ti_2_O_7_ + *x*ZrO_2_

The solution energies of Ce^4+^ and Pu^4+^ substitution on the Zr^4+^ site become progressively more negative with increasing Ce^4+^ and Pu^4+^ concentration; the slope for Pu^4+^ is greater than for Ce^4+^. In contrast, for Th^4+^ substitution, an increase in solution energy is observed with increasing concentration. The solution energy for U^4+^ substitution initially follows a downward trend, however, beyond a U concentration of 12%, there is an abrupt increase in solution energy. Thereafter, the solution energies for U^4+^ substitution follow a downward trend despite the discontinuity in solution energy from 12–15% concentration.

### 4.2 Ca_1−*x*_M_*x*_ZrTi_2−2*x*_Al_2*x*_O_7_ (M = Ce, Pu, Th, U)

**Fig. 4:**
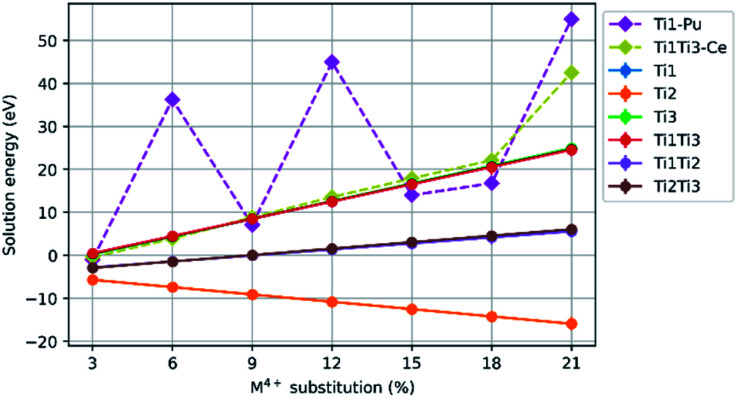
Comparison of solution energy as a function of M^4+^ substitution on the Ca^2+^ site with charge balance of Al^3+^ on Ti^4+^ site combinations, Ti(1)Ti(1), Ti(2)Ti(2), Ti(3)Ti(3), Ti(1)Ti(2), Ti(1)Ti(3), and Ti(2)Ti(3) sites (M = Ce, Pu, Th, U).

Results for Ce^4+^, Pu^4+^, Th^4+^ and U^4+^ substitution on the Ca^2+^ site and charge balance by replacement of Ti^4+^ with Al^3+^, are presented in Fig. 4. The mean solution energies are plotted against M^4+^ substitution concentration, with Al^3+^ as the charge balancing species substituted on sites Ti(1)Ti(1), Ti(2)Ti(2), Ti(3)Ti(3), Ti(1)Ti(2), Ti(1)Ti(3), and Ti(2)Ti(3). The solution energies of particular substitution schemes which do not follow the general trends are plotted separately with their own dashed lines. There is an upward trend in solution energy with increasing Ce, Pu, Th and U concentration for all schemes involving substitution on either the Ti(1) or Ti(3) sites, and the solution energy is always positive above an M^4+^ concentration of 6%. The solution energy only decreases with increasing M^4+^ concentration when charge balancing with Al^3+^ on the Ti(2)Ti(2) site combination, for which the solution energy is always negative.

While Pu^4+^ substitution with Al^3+^ charge balance on two Ti(1) sites follows the general trend of the other substitutions, where the solution energy increases with increasing concentration, we observe maxima in the solution energy at 6%, 12% and 21% M^4+^ concentration. In the case of Al^3+^ charge balance on the combination of Ti(1) and Ti(3) sites, Ce^4+^ substitution did not follow the trend of Pu^4+^, Th^4+^ and U^4+^ so the data for it is plotted individually on the graph.

### 4.3 Ca_1−*x*_M_*x*_ZrTi_2−2*x*_Fe_2*x*_O_7_ (M = Ce, Pu, Th, U)

**Fig. 5:**
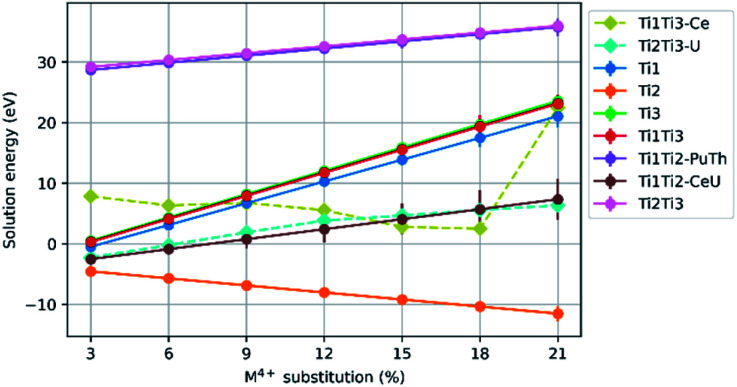
Comparison of solution energy as a function of M^4+^ substitution on the Ca^2+^ site and charge balance of Fe^3+^ on Ti^4+^ site combinations, Ti(1)Ti(1), Ti(2)Ti(2), Ti(3)Ti(3), Ti(1)Ti(2), Ti(1)Ti(3), and Ti(2)Ti(3) sites (M = Ce, Pu, Th, U).

Fig. 5 shows the results for substitution of Ce^4+^, Pu^4+^, Th^4+^ and U^4+^ substitution on the Ca^2+^ site of the zirconolite-2M structure with charge balance by replacement of Ti^4+^ with Fe^3+^. The mean solution energies are plotted against M^4+^ substitution concentration, with Fe^3+^ as the charge balancing species substituted on sites Ti(1)Ti(1), Ti(2)Ti(2), Ti(3)Ti(3), Ti(1)Ti(2), Ti(1)Ti(3), and Ti(2)Ti(3). Again, the solution energies of particular substitution schemes which do not follow the general trends are plotted separately with their own dashed lines.

The general trends in solution energy with Fe^3+^ as a charge balancing species are similar to those of Al^3+^. All substitution schemes showed an increase in solution energy with increasing M^4+^ concentration, with two exceptions. In the case of Ce^4+^ substitution with Fe^3+^ charge balancing on the Ti(1)Ti(3) site, the solution energy decreased with increased Ce^4+^ concentration, however, we observe a large increase in solution energy in the compositional interval between 18–21% Ce^4+^ incorporation. Whereas, in the case of U^4+^ substitution with Fe^3+^ charge balancing on Ti(2)Ti(3) sites, the solution energies for each compositional interval are much lower than for counterpart M^4+^ substitutions.

## 5 Discussion

The negative solution energies presented in Fig. 3 suggest that zirconolite-2M may fully accommodate Ce^4+^, U^4+^, Th^4+^ and Pu^4+^ on the Zr^4+^ site at low to moderate concentrations, which is in broad agreement with experimental validations for corresponding CaZr_1−*x*_M_*x*_Ti_2_O_7_ solid solutions (M = Ce, U, Th, Pu). Furthermore, as the concentration of substitution is increased, the mixing of Ce^4+^, U^4+^ and Pu^4+^ is increasingly favoured, but only up to a value of 15% in the case of U^4+^, where we observe a discontinuity in solution energy. The observed discontinuity in solution energy for U^4+^ is consistent with the apparent solid solution limit of U in the zirconolite-2M structure as reported by Vance *et al.*^13^ Transformation to the zirconolite-4M polytype structure was reported in excess of approximately 15% U^4+^ substitution in the Zr^4+^ site of the zirconolite-2M structure.^13^ The 4M polytype also crystallises in the space group *C*2/*c* and is commonly described as an intergrowth of zirconolite-2M and pyrochlore-type layers, parallel to the [001] axis, resulting in a doubling of the unit cell.^52^ The zirconolite-4M phase remains the dominant structure in the CaZr_1−*x*_U_*x*_Ti_2_O_7_ system up to a value of approximately 40% substitution, after which the cubic pyrochlore CaUTi_2_O_7_-type structure is preferred. Similar solid solution limits for Ce in the corresponding CaZr_1−*x*_Ce_*x*_Ti_2_O_7_ system were reported by Blackburn *et al.*^53^ and Begg *et al.*^54^ with the Ce inventory preferentially accommodated in the zirconolite-4M structure above 20% incorporation. However, it must be recognised that the tendency of Ce^4+^ to undergo reduction to Ce^3+^, when processing under inert or reducing conditions, does not permit formation of zirconolite-4M in the same solid solution, rather a Ce-rich CaTiO_3_ phase is preferentially formed.^53^ Nevertheless, targeting equimolar Ce^3+^ substitution between Ca^2+^ and Zr^4+^ sites, *i.e.* Ca_1−*x*_Zr_1−*x*_Ce_2*x*_Ti_2_O_7_ was observed to yield a transformation to zirconolite-4M.^55^ Begg *et al.* fabricated the CaZr_1−*x*_Pu_*x*_Ti_2_O_7_ solid solution confirming that Pu^4+^ was preferentially accommodated in the 4M structure at around 15% incorporation, consistent with data for Ce^4+^ and U^4+^.^56^ Consequently, simulation studies of substituent reduction and polytype transitions of zirconolite are necessary.

It follows that a similar trend would be expected for Pu in the data presented in Fig. 3, however, a continuous trend of negative solution energy was observed. It should be noted that a number of configurations did fail to optimise in our simulations, suggesting that certain defect arrangements are highly unfavourable. This may correspond to experimental observations, in which the 2M structure becomes less favourable towards high substitution concentrations, possibly due to substituent proximity within a lattice. Our observation that Ce^4+^ and Pu^4+^ substitute favourably for Zr^4+^ in zirconolite is further supported by the observations of Gilbert *et al.*^24^ These data indicate that, whilst Ce remains a safe and practical analogue for Pu in wasteform development trials, it cannot fully replicate the substitution behaviour of Pu in zirconolite. Despite a similar trend to Pu^4+^ and U^4+^ at low concentrations, a clear variation in the solution energy, as a function of substitution, was observed. Nevertheless, the limitations of Ce–Pu surrogacy have been previously discussed in the context of Pu immobilisation in ceramic materials.^57,58^

In contrast, the substitution of Th^4+^ for Zr^4+^ produces a continuous positive upward trend in solution energy, which becomes positive above 9% Th substitution, suggesting that Th^4+^ may have a narrow solid solution range in the zirconolite-2M structure. These data are consistent with recent observations by Blackburn *et al.*^59^ in which it was confirmed that the solubility of Th^4+^ in the CaZr_1−*x*_Th_*x*_Ti_2_O_7_ solid solution was limited to 10% substitution for Zr^4+^, with Th^4+^ preferentially accommodated in a pyrochlore-structured phase between 0.10 ≤ *x* ≤ 0.50. The single phase pyrochlore compound CaZr_0.40_Th_0.60_Ti_2_O_7_ was produced when targeting *x* = 0.60. Interestingly, a phase transition to the zirconolite-4M structure, as reported in analogue Ce and U solid solutions, was not observed.

The data presented in Fig. 4 and 5 demonstrate that the substitution of Ce^4+^, U^4+^, Th^4+^ and Pu^4+^ in the Ca^2+^ site, with charge balance provided by Al^3+^ and Fe^3+^ is favoured at M^4+^ concentrations around 3%. Yet, these solid solutions become rapidly unfavourable, tending towards positive solution energy with the exception of the substitution scheme in which charge compensators were accommodated in the Ti(2) site. This substitution scheme follows a downward trend in solution energy, suggesting that surrogate species may be accommodated in the Ca^2+^ site up to a 21% substitution, with Al^3+^ and Fe^3+^ preferentially accommodated in the Ti(2) site, consistent with some experimental observations. Loiseau *et al.*^60^ fabricated the Ca_1−*x*_Nd_*x*_ZrTi_2−*x*_Al_*x*_O_7_ solid solution, confirming that zirconolite-2M was produced as a single phase in the compositional range *x* ≤ 0.60, with Nd^3+^ deployed as a trivalent actinide surrogate, and further substitution resulted in the formation of the orthorhombic 3O polytype. Rietveld refinement of a zirconolite-2M structural model, in which Al^3+^ was constrained in the Ti(2) site, was refined against powder X-ray diffraction data for Ca_0.7_Nd_0.3_ZrTi_1.7_Al_0.3_O_7_, confirmed that Al^3+^ preferentially occupied this site relative to Ti(1) and Ti(3). Similarly, Fe^3+^ was demonstrated by Whittle *et al.*^37^ to substitute for Ti(2) in the CaZrTi_2−2*x*_Nb_*x*_Fe_*x*_O_7_ solid solution, however, it must be recognised that no surrogate as targeted to replace Ca^2+^. Conversely, Fe K-edge XANES has failed to resolve any preferential occupation of Fe^3+^ between Ti(1)/Ti(3) and Ti(2) sites in the Ca_1−*x*_Ho_*x*_ZrTi_2−*x*_Fe_*x*_O_7_.^61^ Similarly, Forder *et al.*^62^ resolved Fe^3+^ coordination in single phase zirconolite-2M (targeting Ca_1−*x*_Ce_*x*_ZrTi_2−2*x*_Fe_2*x*_O_7_) using ^57^Fe Mössbauer spectroscopy, confirming that whilst Fe^3+^ occupied both 5- and 6-fold coordination Ti^4+^ sites, occupation of the Ti(2) site was preferred at low Fe^3+^ concentration. Previous simulation studies did not identify a significant preference of Fe^3+^ in any particular Ti^4+^ site of zirconolite-2M.^24^ Cr^3+^ coordination was probed by Blackburn *et al.* in the Ca_1−*x*_Ce_*x*_ZrTi_2−2*x*_Cr_2*x*_O_7_ system, with deconvolution of the pre-edge Cr K-edge XANES region consistent with Cr^3+^ arranged in 6-fold coordination, inferring occupation in the octahedral Ti(1) and Ti(3) sites, as expected from consideration of crystal field stabilisation energy.^63^

The variation in dominant charge compensation mechanism and preferential site occupancy of charge compensation species in zirconolite-2M may be attributed to a several factors, not limited to: the choice of Pu surrogate deployed, the valence state, electronic structure and ionic radius of the charge compensation cation, and the partial oxygen pressure imposed during the fabrication route, which has been systematically demonstrated to influence both surrogate oxidation state and partitioning in the zirconolite-2M structure. This work also provided some evidence that, in line with experimental observations, Pu^4+^ may be favourably accommodated on the Ca^2+^ site in the zirconolite structure, whereas substitution of Ce^4+^ may be unfavourable in some instances. However, it is important to note that the underlying mechanism constraining the site occupancy of Ce within zirconolite-2M is controlled by the prevailing redox conditions imposed during synthesis, and is not entirely governed by the chosen solid solution regime. Whereas Pu^4+^ is readily incorporated in the zirconolite-2M phase under oxidising conditions, Ce^4+^ has a tendency to partially reduce to Ce^3+^ regardless of sintering environment. For example, synthesis of the CaZr_1−*x*_Ce_*x*_Ti_2_O_7_ and Ca_1−*x*_Ce_*x*_ZrTi_2−2*x*_Cr_2*x*_O_7_ solid solutions in air consistently resulted in partial reduction of the Ce^4+^ inventory to Ce^3+^. It is this underlying auto-reduction tendency, that does not present itself with Pu under such conditions, that is the limiting factor in Ce–Pu surrogacy. Near single phase zirconolite-2M materials with nominal composition Ca_0.8_Pu_0.2_HfTi_1.6_Al_0.4_O_7_ and Ca_0.8_Pu_0.2_ZrTi_1.8_Al_0.2_O_7_ have been previously reported by both conventional sintering and hot pressing techniques, targeting Pu^4+^ and Pu^3+^ respectively.^20,64^ Analysis of the compositionally analogous Ca_1−*x*_Ce_*x*_ZrTi_2−2*x*_Al_2*x*_O_7_ solid solution, at *x* = 0.10, has been observed to produce a minor perovskite phase, attributed to partial Ce^3+^ speciation-2M.^65^ The differences between simulated Ce^4+^ and Pu^4+^ substitution behaviour suggests that clustering and localised substituent–substituent interactions, which would be present in the current work yet excluded from previous data,^24^ may be key for stabilising the substituents. The combined presence of numerous defects may relieve the localised stress they create, as opposed to lone, or few, defects.^28^ This may also explain the data presented by Ji *et al.*^61^ where the incorporation of Ln^3+^ species in the Ca^2+^ site would lead to a weaker binding energy with Fe^3+^ defects distributed across the Ti^4+^ sites, and thus preferential occupation of Fe^3+^ within any specific Ti^4+^ site was not reported. It remains clear that in the present study, and as has been confirmed in a selection of laboratory investigations, that low valence charge balancing cations preferentially occupy the trigonal biprymidal TiO_5_ site. It follows that the partially occupied nature of this site (50% occupied), relative to Ti(1) and Ti(3), permits the accommodation of cations of varied size.

From examination of the collective substituent behaviour we establish that at low concentrations (*e.g.* 3%) a selection of charge compensated substitution schemes are viable within the zirconolite-2M phase, and it has been experimentally determined that environmental conditions, chemical activity and ion mobility may dictate the solid solution mechanisms that occur. As the nominal concentration of substitution is increased, it is to be expected that clustering of defects will occur in either the Zr^4+^ site or Ca^2+^ site, facilitated by charge compensation on the Ti(2) site. The investigation presented herein shows that substitution schemes involving the Ca^2+^ site reach lower solution energies when charge balancing with Al^3+^ on the Ti(2) site, this requires further computational work to elucidate, an interpretation is that Al^3+^ being smaller than Fe^3+^ can be accommodated more easily. Nevertheless, these data support the deployment of zirconolite-2M as a potential single host phase for the immobilisation of Pu oxides, and are in general agreement with a selection of recent publications concerning the solid solution behaviour of Ce, U, Th and Pu.

## 6 Conclusions

We have demonstrated trends in the energetics of zirconolite-2M solid solutions with Ce^4+^, Pu^4+^, Th^4+^, and U^4+^ cations that are in general agreement with published experimental data concerning the deployment of zirconolite-2M as a host for actinides. Consequently, we have shown that atomistic simulations can effectively guide the formulation development of these materials, and inform experimental validation. For example, using the high throughput methodology developed and reported here, it is possible to rapidly screen many solid solution schemes *in silico* and evaluate their relative stability, to guide more resource intensive *ab initio* simulations and laboratory investigation for validation. Indeed, this method should have much wider utility in the exploration in the optimisation of functional materials such as high entropy alloys, capacitor ceramics, and perovskite catalysts. Our investigations have also shown that Ce is not a direct analogue for the actinide cations such as Pu, as has been validated in a number of wasteform development trials.

## Author contributions

S. D. – data curation, investigation, formal analysis, methodology, software, visualization, writing – original draft, writing – review & editing. C. M. H. – conceptualization, formal analysis, investigation, methodology, software, writing – review & editing. L. R. B. – investigation, validation, writing – review & editing. C. L. F. – project administration, resources, supervision, writing – review & editing. N. C. H. – conceptualization, funding acquisition, project administration, resources, supervision, writing – review & editing.

## Conflicts of interest

There are no conflicts to declare.

## Supplementary Material

RA-011-D1RA02914B-s001
