# Supplementary material for: A high throughput computational investigation of the solid solution mechanisms of actinides and lanthanides in zirconolite
Source: RSC Adv. 2021 Jul 20;11(41):25179–86. doi: 10.1039/d1ra02914b (PMC9037047; doi:10.1039/d1ra02914b)
Supplement: RA-011-D1RA02914B-s001 [file RA-011-D1RA02914B-s001.pdf]

Electronic Supplementary Material (ESI) for RSC Advances.
This journal is © The Royal Society of Chemistry 2021

$Ca_{1-x}Ce_xZrTi_{2-2x}Al_{2x}O_7$

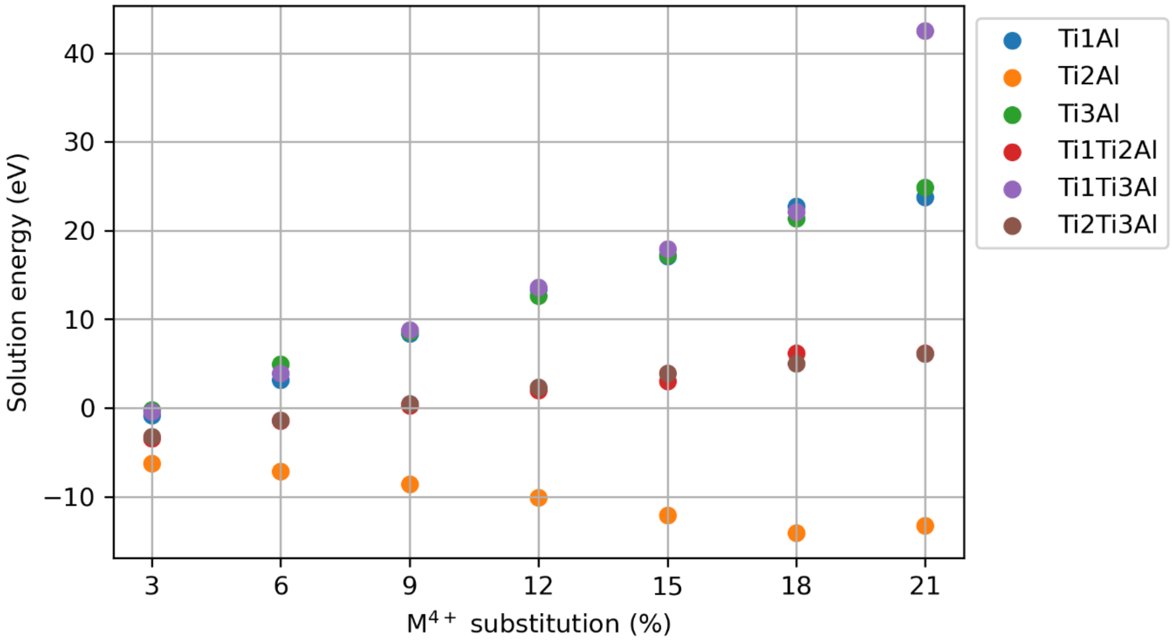

$Ca_{1-x}Ce_xZrTi_{2-2x}Fe_{2x}O_7$

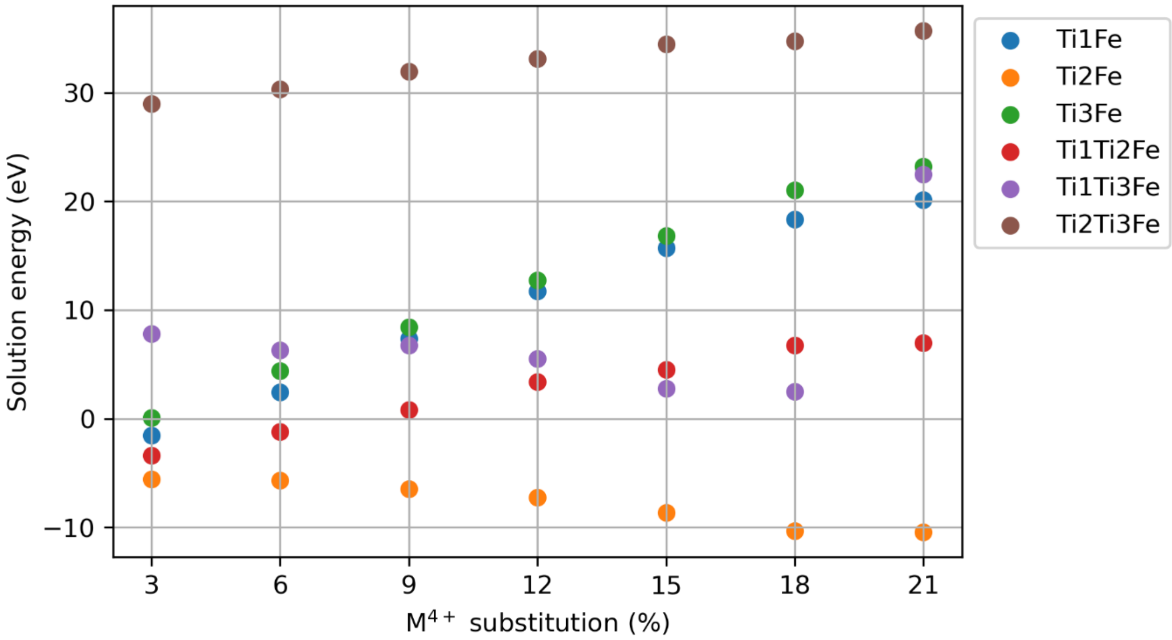

$Ca_{1-x}Pu_xZrTi_{2-2x}Al_{2x}O_7$

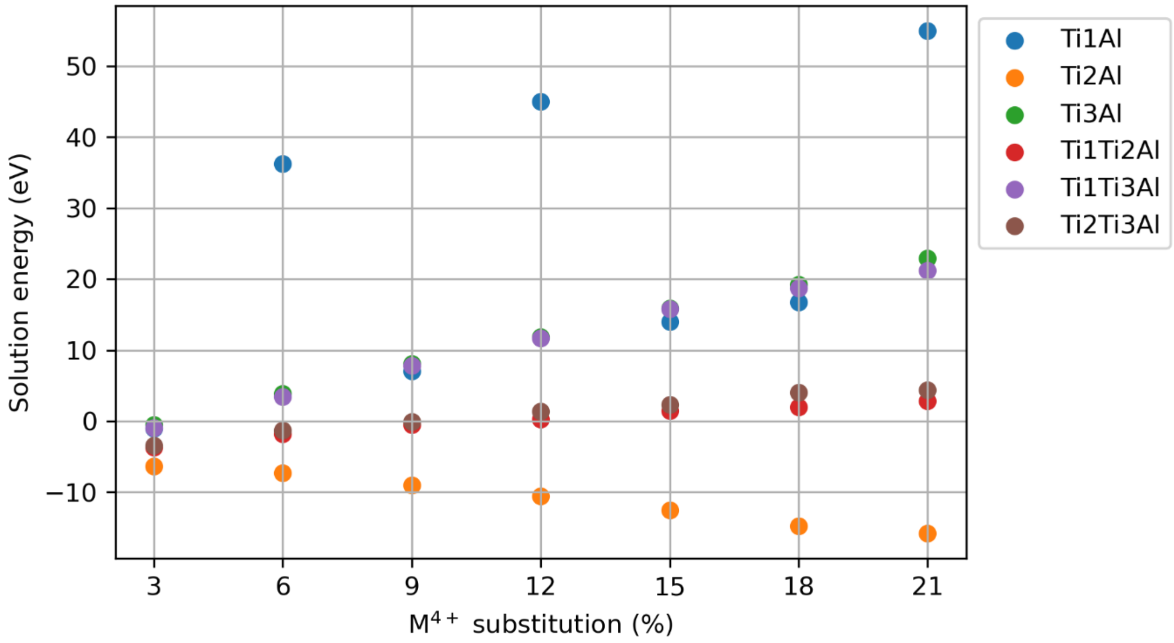

$Ca_{1-x}Pu_xZrTi_{2-2x}Fe_{2x}O_7$

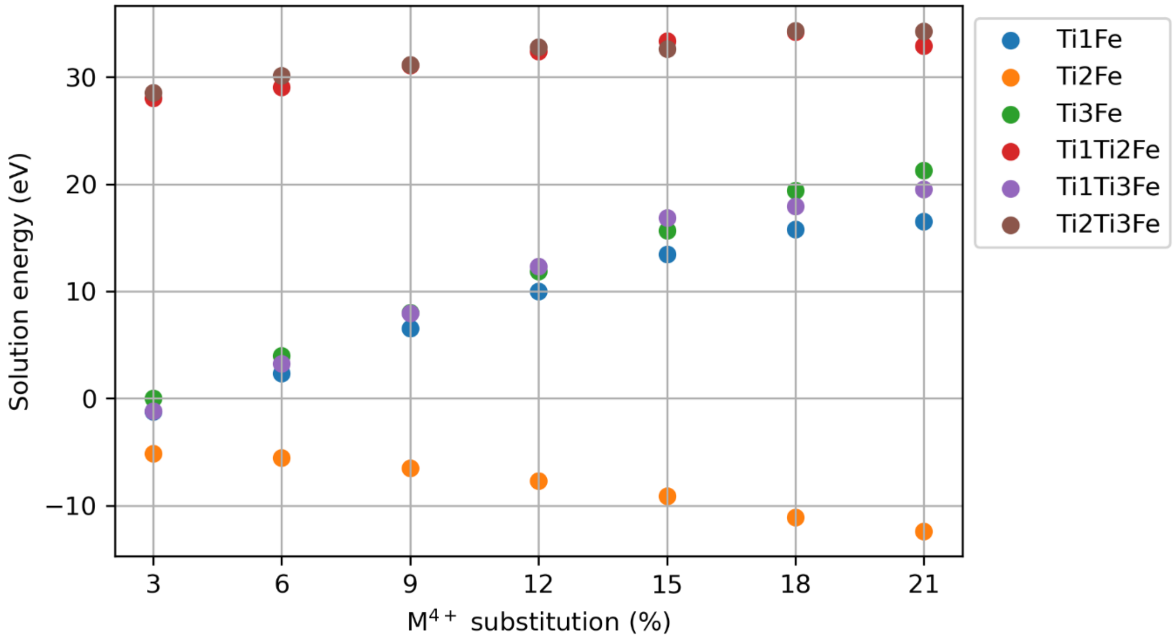

$Ca_{1-x}Th_xZrTi_{2-2x}Al_{2x}O_7$

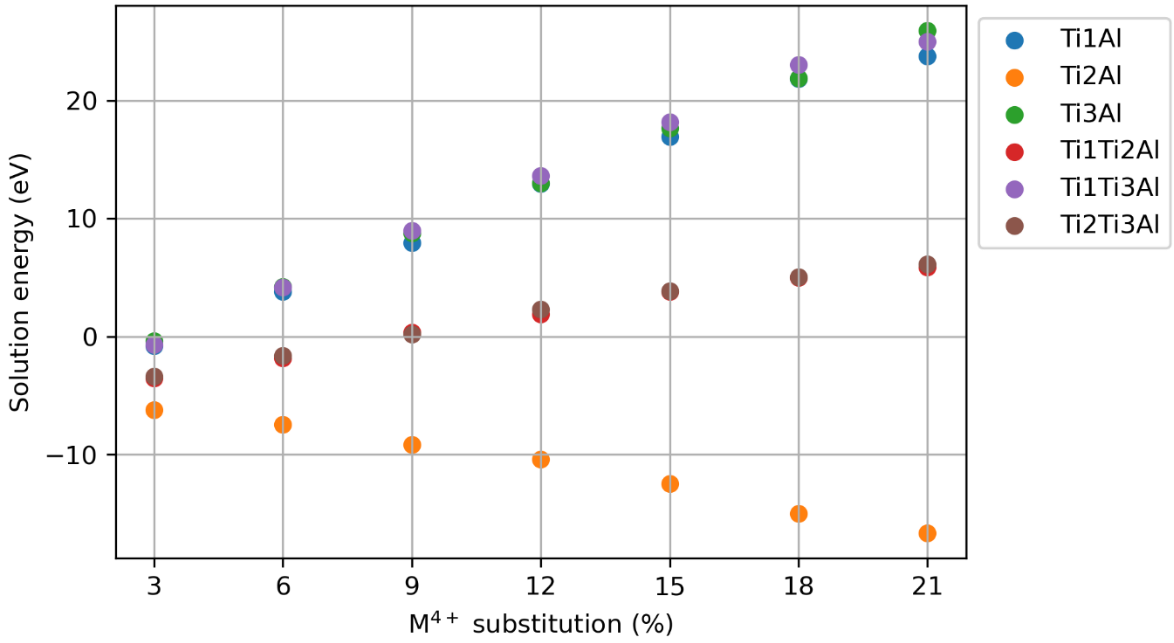

$Ca_{1-x}Th_xZrTi_{2-2x}Fe_{2x}O_7$

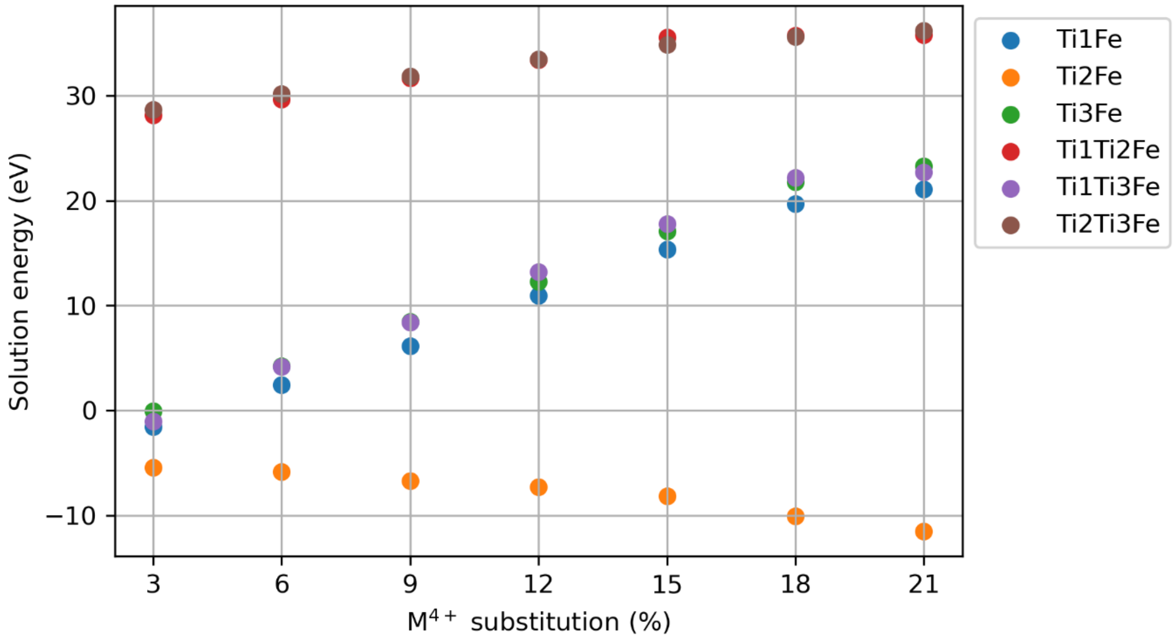

$Ca_{1-x}U_xZrTi_{2-2x}Al_{2x}O_7$

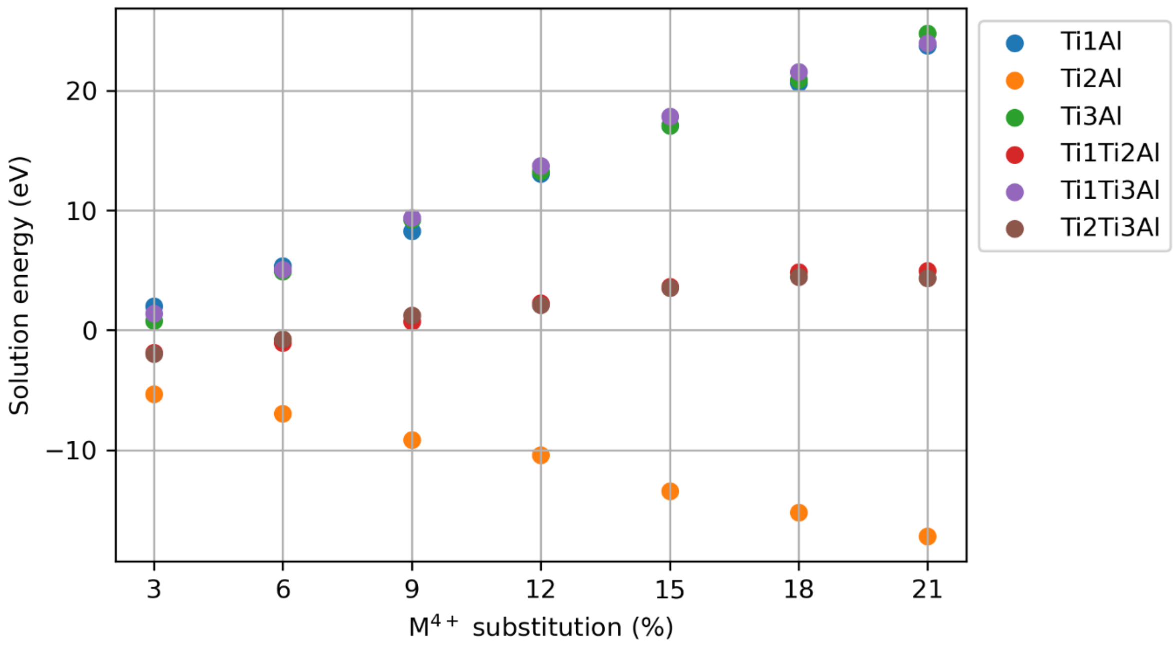

$Ca_{1-x}U_xZrTi_{2-2x}Fe_{2x}O_7$

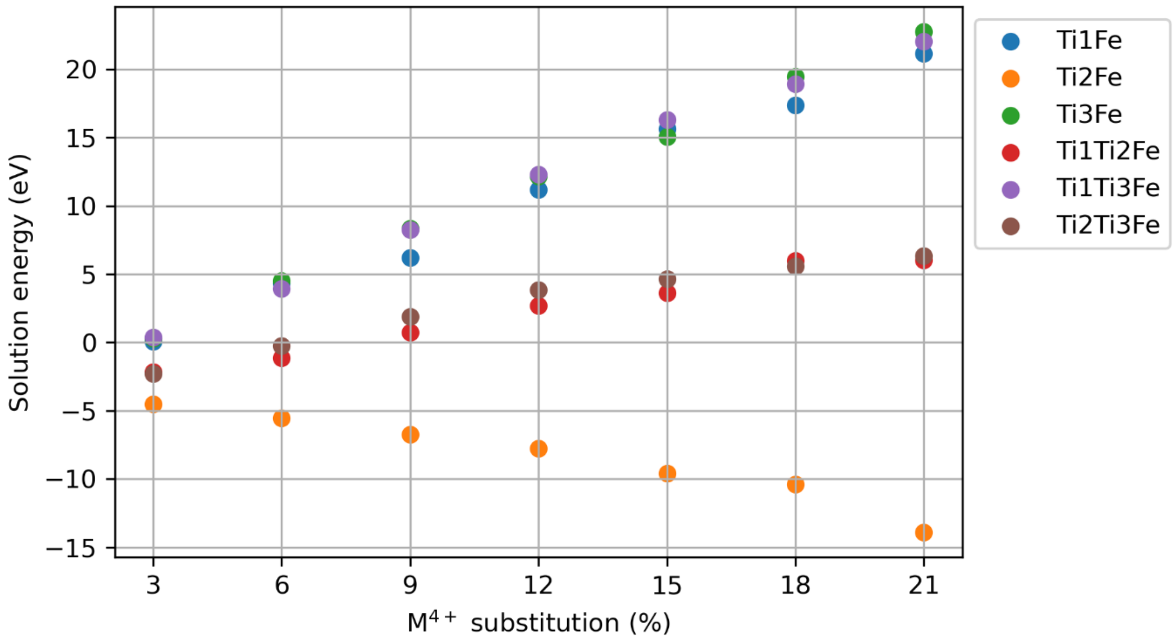

$CaZr_{1-x}M_xTi_2O_7$, M = (Ce, Pu, Th, U)

N.B. All stated energies are in eV

Ce

	ground state energy	solid solution energy												
concentration in solid solution (%)	3		6		9		12		15		18		21	
number of atoms	2		4		6		8		10		12		13	
	-25173.43	-4.67	-25156.75	-5.29	-25139.56	-5.41	-25122.93	-6.09	-25105.88	-6.34	-25089.43	-7.20	-25081.07	-7.50
	-25173.31	-4.55	-25156.60	-5.15	-25139.70	-5.55	-25122.75	-5.91	-25105.65	-6.11	-25089.20	-6.97	-25080.98	-7.40
	-25173.42	-4.67	-25156.50	-5.05	-25139.39	-5.24	-25122.98	-6.14	-25105.71	-6.18	-25088.99	-6.76	-25080.76	-7.19
	-25173.30	-4.55	-25156.63	-5.18	-25139.75	-5.60	-25122.77	-5.93	-25106.19	-6.66	-25089.21	-6.98	-25080.74	-7.16
	-25173.33	-4.57	-25156.62	-5.17	-25139.58	-5.44	-25122.98	-6.14	-25105.99	-6.46	-25089.06	-6.83	-25080.28	-6.71
	-25173.44	-4.68	-25156.63	-5.18	-25139.41	-5.27	-25122.62	-5.78	-25105.89	-6.35	-25089.29	-7.06	-25080.97	-7.39
	-25173.52	-4.77	-25156.50	-5.05	-25139.33	-5.18	-25122.62	-5.78	-25105.63	-6.10	-25089.24	-7.01	-25080.07	-6.49
	-25173.55	-4.79	-25156.42	-4.97	-25139.51	-5.37	-25122.75	-5.91	-25105.74	-6.21	-25089.10	-6.87	-25080.83	-7.26
	-25173.42	-4.67	-25156.52	-5.06	-25139.46	-5.32	-25122.75	-5.91	-25105.73	-6.19	-25089.09	-6.86	-25080.81	-7.23
	-25173.30	-4.55	-25156.39	-4.94	-25139.63	-5.49	-25122.64	-5.79	-25105.92	-6.38	-25089.17	-6.94	-25080.31	-6.73
	-25173.30	-4.55	-25156.36	-4.91	-25139.47	-5.33	-25123.07	-6.23	-25105.68	-6.15	-25088.85	-6.62	-25080.25	-6.67
	-25173.42	-4.66	-25156.62	-5.17	-25139.66	-5.52	-25122.38	-5.54	-25105.81	-6.28	-25089.30	-7.07	-25080.79	-7.21
	-25173.30	-4.54	-25156.54	-5.09	-25139.72	-5.57	-25122.40	-5.56	-25106.04	-6.50	-25089.16	-6.92	-25080.70	-7.12
	-25173.42	-4.67	-25156.39	-4.93	-25139.68	-5.53	-25122.88	-6.04	-25106.05	-6.51	-25088.98	-6.75	-25080.54	-6.96
	-25173.44	-4.68	-25156.53	-5.08	-25139.37	-5.22	-25122.62	-5.78	-25106.11	-6.57	-25089.02	-6.79	-25080.48	-6.90
	-25173.43	-4.67	-25156.51	-5.05	-25139.49	-5.34	-25122.85	-6.01	-25105.99	-6.46	-25089.03	-6.80	-25080.59	-7.02
	-25173.42	-4.67	-25156.62	-5.17	-25139.95	-5.81	-25123.12	-6.28	-25106.17	-6.64	-25089.11	-6.88	-25080.58	-7.00
	-25173.44	-4.68	-25156.76	-5.31	-25139.85	-5.71	-25122.42	-5.58	-25105.74	-6.20	-25088.77	-6.54	-25081.10	-7.52
	-25173.54	-4.79	-25156.51	-5.05	-25139.73	-5.58	-25122.67	-5.83	-25105.76	-6.22	-25088.47	-6.24	-25080.67	-7.10
	-25173.42	-4.67	-25156.54	-5.09	-25139.79	-5.64	-25122.59	-5.75	-25105.68	-6.14	-25089.19	-6.96	-25080.64	-7.06
	-25173.31	-4.55	-25156.39	-4.94	-25139.75	-5.60	-25122.76	-5.92	-25106.18	-6.65	-25088.91	-6.68	-25080.72	-7.14
	-25173.41	-4.65	-25156.53	-5.07	-25139.72	-5.57	-25122.83	-5.98	-25105.70	-6.16	-25089.06	-6.83	-25080.32	-6.74
	-25173.42	-4.67	-25156.48	-5.02	-25139.64	-5.49	-25122.56	-5.72	-25105.82	-6.28	-25089.27	-7.04	-25080.65	-7.08
	-25173.30	-4.55	-25156.66	-5.21	-25139.51	-5.37	-25122.75	-5.91	-25105.93	-6.39	-25089.46	-7.23	-25080.69	-7.11
	-25173.54	-4.79	-25156.61	-5.16	-25139.50	-5.36	-25122.66	-5.81	-25105.92	-6.39	-25088.97	-6.74	-25080.66	-7.08
	-25173.42	-4.66	-25156.56	-5.11	-25139.77	-5.62	-25122.74	-5.90	-25105.98	-6.44	-25088.79	-6.56	-25080.83	-7.26
	-25173.31	-4.55	-25156.50	-5.05	-25139.84	-5.69	-25122.76	-5.92	-25106.06	-6.52	-25089.21	-6.98	-25080.89	-7.31
	-25173.54	-4.79	-25156.56	-5.11	-25139.76	-5.62	-25122.86	-6.02	-25105.78	-6.24	-25089.19	-6.96	-25080.98	-7.40
	-25173.54	-4.78	-25156.52	-5.07	-25139.52	-5.38	-25122.80	-5.96	-25105.66	-6.12	-25089.22	-6.99	-25080.47	-6.90
	-25173.31	-4.55	-25156.69	-5.24	-25139.62	-5.47	-25122.77	-5.93	-25105.88	-6.34	-25089.22	-6.99	-25080.48	-6.90

Average energies	-25173.41	-4.65	-25156.55	-5.10	-25139.62	-5.48	-25122.74	-5.90	-25105.88	-6.34	-25089.10	-6.87	-25080.66	-7.08

Pu

	ground state energy	solid solution energy												
concentration in solid solution (%)	3		6		9		12		15		18		21	
number of atoms	2		4		6		8		10		12		13	
	-25168.81	-6.04	-25147.09	-7.61	-25125.86	-9.67	-25104.07	-11.18	-25082.77	-13.16	-25061.21	-14.89	-25050.48	-15.81
	-25168.89	-6.12	-25147.26	-7.78	-25125.79	-9.60	-25104.25	-11.36	-25082.76	-13.15	-25061.00	-14.68	-25050.81	-16.14
	-25168.81	-6.03	-25147.24	-7.76	-25125.92	-9.73	-25104.04	-11.14	-25083.06	-13.45	-25061.38	-15.07	-25050.45	-15.78
	-25168.89	-6.12	-25147.18	-7.70	-25125.67	-9.48	-25104.27	-11.37	-25082.97	-13.36	-25061.25	-14.94	-25050.18	-15.50
	-25168.92	-6.15	-25147.17	-7.69	-25125.68	-9.49	-25104.08	-11.18	-25083.00	-13.39	-25061.44	-15.12	-25050.56	-15.89
	-25168.81	-6.04	-25147.13	-7.65	-25125.64	-9.45	-25104.25	-11.35	-25082.96	-13.35	-25061.51	-15.20	-25050.62	-15.95
	-25168.85	-6.08	-25147.26	-7.78	-25125.75	-9.56	-25104.24	-11.34	-25082.87	-13.26	-25061.44	-15.12	-25050.59	-15.92
	-25168.73	-5.96	-25147.38	-7.90	-25125.86	-9.67	-25104.19	-11.29	-25082.83	-13.22	-25061.40	-15.09	-25051.08	-16.41
	-25168.80	-6.03	-25147.29	-7.81	-25125.96	-9.77	-25104.43	-11.53	-25082.79	-13.18	-25061.03	-14.71	-25050.68	-16.01
	-25168.89	-6.12	-25147.39	-7.91	-25125.65	-9.46	-25104.29	-11.39	-25083.00	-13.39	-25061.26	-14.94	-25050.37	-15.70
	-25168.89	-6.12	-25147.44	-7.96	-25126.08	-9.89	-25104.12	-11.23	-25083.04	-13.43	-25061.72	-15.40	-25050.93	-16.26
	-25168.81	-6.04	-25147.17	-7.69	-25125.76	-9.57	-25104.49	-11.59	-25083.20	-13.59	-25061.09	-14.77	-25050.60	-15.93
	-25168.89	-6.12	-25147.28	-7.80	-25125.53	-9.34	-25104.68	-11.78	-25082.67	-13.06	-25061.04	-14.72	-25051.07	-16.40
	-25168.80	-6.03	-25147.33	-7.85	-25125.89	-9.70	-25104.13	-11.23	-25082.66	-13.05	-25061.15	-14.83	-25050.71	-16.04
	-25168.82	-6.05	-25147.30	-7.82	-25125.94	-9.75	-25104.17	-11.27	-25082.86	-13.25	-25061.63	-15.32	-25050.66	-15.98
	-25168.80	-6.03	-25147.25	-7.77	-25125.91	-9.72	-25104.03	-11.13	-25082.89	-13.29	-25061.24	-14.92	-25050.63	-15.96
	-25168.80	-6.03	-25147.18	-7.70	-25125.66	-9.47	-25104.18	-11.28	-25082.87	-13.26	-25061.21	-14.89	-25050.77	-16.09
	-25168.81	-6.04	-25147.13	-7.65	-25125.54	-9.35	-25104.58	-11.68	-25082.80	-13.20	-25061.52	-15.21	-25050.24	-15.57
	-25168.73	-5.96	-25147.22	-7.73	-25125.59	-9.40	-25104.31	-11.41	-25082.78	-13.17	-25061.45	-15.13	-25050.73	-16.05
	-25168.81	-6.04	-25147.29	-7.81	-25125.71	-9.52	-25104.23	-11.33	-25083.05	-13.44	-25061.21	-14.89	-25050.91	-16.24
	-25168.87	-6.09	-25147.37	-7.89	-25125.58	-9.39	-25104.25	-11.35	-25082.93	-13.32	-25061.37	-15.06	-25050.58	-15.91
	-25168.80	-6.03	-25147.27	-7.79	-25125.69	-9.50	-25104.13	-11.23	-25082.83	-13.23	-25061.61	-15.29	-25050.56	-15.88
	-25168.80	-6.03	-25147.17	-7.69	-25125.80	-9.61	-25104.20	-11.30	-25082.66	-13.05	-25061.25	-14.94	-25050.63	-15.96
	-25168.89	-6.12	-25147.18	-7.70	-25125.87	-9.68	-25104.21	-11.31	-25082.85	-13.25	-25061.16	-14.84	-25050.81	-16.14
	-25168.73	-5.96	-25147.15	-7.67	-25125.83	-9.64	-25104.33	-11.43	-25082.93	-13.32	-25061.55	-15.23	-25050.45	-15.78
	-25168.79	-6.02	-25147.32	-7.84	-25125.80	-9.61	-25104.23	-11.33	-25082.94	-13.33	-25061.49	-15.18	-25050.82	-16.15
	-25168.89	-6.12	-25147.25	-7.77	-25125.64	-9.45	-25104.31	-11.41	-25082.74	-13.13	-25061.13	-14.81	-25050.48	-15.81
	-25168.73	-5.96	-25147.31	-7.83	-25125.67	-9.48	-25104.33	-11.43	-25083.29	-13.68	-25061.58	-15.26	-25050.44	-15.77
	-25168.72	-5.95	-25147.27	-7.79	-25125.94	-9.75	-25104.25	-11.35	-25083.05	-13.44	-25061.45	-15.13	-25050.51	-15.84
	-25168.90	-6.13	-25147.11	-7.63	-25125.88	-9.69	-25104.22	-11.32	-25082.90	-13.29	-25061.45	-15.14	-25050.86	-16.19
Average energies	-25168.82	-6.05	-25147.25	-7.77	-25125.77	-9.58	-25104.25	-11.35	-25082.90	-13.29	-25061.34	-15.02	-25050.64	-15.97

Th

	ground state energy	solid solution energy												
concentration in solid solution (%)	3		6		9		12		15		18		21	
number of atoms	2		4		6		8		10		12		13	
	-25171.40	-3.00	-25153.09	-2.35	-25133.57	-0.49	-25115.39	0.03	-25096.14	1.62	-25078.38	1.72	-25068.31	2.96
	-25171.12	-2.71	-25152.73	-1.99	-25133.89	-0.81	-25114.88	0.54	-25096.16	1.60	-25078.15	1.95	-25069.11	2.16
	-25171.39	-2.99	-25152.55	-1.80	-25133.55	-0.47	-25115.45	-0.03	-25095.55	2.21	-25078.25	1.85	-25069.25	2.02
	-25171.09	-2.69	-25152.84	-2.10	-25134.03	-0.95	-25115.16	0.26	-25095.84	1.92	-25077.49	2.61	-25068.20	3.07
	-25171.15	-2.75	-25152.79	-2.05	-25133.61	-0.53	-25115.53	-0.11	-25096.22	1.54	-25077.84	2.26	-25068.59	2.68
	-25171.46	-3.05	-25152.82	-2.08	-25133.21	-0.13	-25114.46	0.96	-25095.84	1.92	-25078.17	1.93	-25068.77	2.50
	-25171.64	-3.24	-25152.31	-1.57	-25133.00	0.08	-25114.74	0.68	-25096.96	0.80	-25078.05	2.05	-25068.30	2.97
	-25171.71	-3.31	-25152.26	-1.52	-25133.43	-0.35	-25115.15	0.27	-25096.23	1.53	-25078.64	1.46	-25068.87	2.40
	-25171.40	-2.99	-25152.52	-1.78	-25133.36	-0.28	-25114.58	0.84	-25096.91	0.85	-25076.74	3.36	-25068.01	3.26
	-25171.09	-2.69	-25152.19	-1.45	-25133.81	-0.73	-25114.68	0.74	-25096.47	1.29	-25077.21	2.89	-25068.67	2.60
	-25171.09	-2.69	-25152.23	-1.49	-25133.29	-0.21	-25115.83	-0.41	-25095.82	1.94	-25077.15	2.95	-25069.29	1.98
	-25171.38	-2.98	-25152.80	-2.06	-25133.60	-0.52	-25114.13	1.29	-25096.82	0.94	-25077.73	2.37	-25068.15	3.12
	-25171.06	-2.66	-25152.61	-1.86	-25134.00	-0.92	-25114.37	1.06	-25095.74	2.02	-25077.88	2.22	-25068.66	2.61
	-25171.40	-3.00	-25152.19	-1.45	-25133.80	-0.72	-25115.32	0.10	-25096.30	1.46	-25077.32	2.78	-25068.40	2.87
	-25171.46	-3.06	-25152.58	-1.84	-25133.15	-0.06	-25114.73	0.69	-25096.58	1.18	-25077.84	2.26	-25067.37	3.90
	-25171.42	-3.02	-25152.10	-1.36	-25133.37	-0.29	-25115.23	0.19	-25096.34	1.42	-25077.84	2.26	-25068.65	2.62
	-25171.40	-3.00	-25152.80	-2.06	-25134.50	-1.42	-25115.90	-0.48	-25096.24	1.52	-25077.44	2.66	-25067.81	3.46
	-25171.44	-3.04	-25153.12	-2.38	-25134.10	-1.01	-25114.39	1.03	-25096.45	1.31	-25077.15	2.95	-25068.19	3.08
	-25171.70	-3.30	-25152.54	-1.80	-25133.99	-0.91	-25115.30	0.12	-25096.03	1.73	-25077.84	2.26	-25068.10	3.17
	-25171.40	-3.00	-25152.62	-1.88	-25134.17	-1.09	-25114.69	0.73	-25096.04	1.72	-25078.57	1.53	-25068.10	3.17
	-25171.11	-2.71	-25152.01	-1.27	-25134.11	-1.03	-25114.98	0.44	-25096.66	1.10	-25077.17	2.93	-25069.05	2.22
	-25171.34	-2.94	-25152.56	-1.82	-25133.73	-0.65	-25115.25	0.17	-25096.85	0.91	-25077.74	2.36	-25068.59	2.68
	-25171.40	-3.00	-25152.46	-1.71	-25133.73	-0.65	-25114.42	1.00	-25095.37	2.39	-25078.58	1.52	-25068.93	2.34
	-25171.09	-2.69	-25152.94	-2.20	-25133.22	-0.14	-25114.92	0.50	-25096.80	0.97	-25077.83	2.27	-25068.51	2.76
	-25171.70	-3.30	-25152.77	-2.03	-25133.39	-0.30	-25114.56	0.86	-25096.30	1.46	-25077.73	2.37	-25067.74	3.53
	-25171.39	-2.99	-25152.67	-1.93	-25134.01	-0.93	-25115.05	0.37	-25096.23	1.53	-25077.57	2.53	-25067.53	3.74
	-25171.10	-2.70	-25152.47	-1.73	-25134.25	-1.17	-25114.86	0.56	-25095.87	1.89	-25077.19	2.91	-25068.50	2.77
	-25171.70	-3.30	-25152.66	-1.92	-25134.04	-0.96	-25115.53	-0.11	-25096.45	1.31	-25076.82	3.28	-25068.74	2.53
	-25171.69	-3.28	-25152.54	-1.80	-25134.11	-1.03	-25115.17	0.25	-25096.23	1.53	-25077.76	2.34	-25068.39	2.88
	-25171.11	-2.71	-25152.95	-2.21	-25133.68	-0.60	-25115.05	0.37	-25096.85	0.91	-25078.05	2.05	-25068.79	2.48
Average energies	-25171.36	-2.96	-25152.59	-1.85	-25133.72	-0.64	-25114.99	0.43	-25096.28	1.48	-25077.74	2.36	-25068.45	2.82

U

	ground state energy	solid solution energy													
concentration in solid solution (%)	3		6		9		12		15		18		21		
number of atoms	2		4		6		8		10		12		13		
	-25179.58	-8.07	-25169.23	-12.28	-25157.96	-15.57	-25147.73	-19.89	-25124.00	-10.72	-25110.94	-12.21	-25103.46	-12.01	
	-25179.38	-7.87	-25168.99	-12.04	-25158.25	-15.86	-25147.43	-19.59	-25124.06	-10.78	-25111.33	-12.60	-25103.99	-12.54	
	-25179.57	-8.07	-25168.82	-11.87	-25157.70	-15.31	-25147.82	-19.98	-25124.32	-11.03	-25111.17	-12.44	-25104.00	-12.55	
	-25179.37	-7.86	-25169.04	-12.09	-25158.31	-15.92	-25147.49	-19.65	-25124.01	-10.72	-25111.01	-12.28	-25104.76	-13.31	
	-25179.39	-7.88	-25169.02	-12.07	-25158.06	-15.66	-25147.80	-19.96	-25123.86	-10.57	-25110.17	-11.45	-25104.63	-13.18	
	-25179.60	-8.09	-25169.03	-12.08	-25157.81	-15.42	-25147.20	-19.36	-25123.36	-10.08	-25111.16	-12.44	-25104.77	-13.32	
	-25179.76	-8.25	-25168.81	-11.86	-25157.64	-15.25	-25147.18	-19.34	-25123.85	-10.57	-25110.46	-11.74	-25104.42	-12.97	
	-25179.79	-8.28	-25168.65	-11.70	-25157.90	-15.51	-25147.41	-19.57	-25123.67	-10.39	-25110.68	-11.95	-25104.37	-12.93	
	-25179.57	-8.07	-25168.82	-11.87	-25157.87	-15.48	-25147.41	-19.58	-25123.91	-10.63	-25110.87	-12.15	-25104.39	-12.95	
	-25179.37	-7.86	-25168.61	-11.66	-25158.14	-15.75	-25147.20	-19.36	-25123.78	-10.50	-25111.13	-12.40	-25104.61	-13.16	
	-25179.37	-7.86	-25168.62	-11.67	-25157.90	-15.51	-25147.91	-20.07	-25123.83	-10.54	-25110.47	-11.74	-25103.95	-12.51	
	-25179.57	-8.06	-25169.02	-12.07	-25158.15	-15.76	-25146.88	-19.05	-25124.10	-10.82	-25110.07	-11.34	-25104.46	-13.01	
	-25179.36	-7.85	-25168.86	-11.91	-25158.30	-15.91	-25146.80	-18.96	-25123.68	-10.40	-25110.86	-12.13	-25104.58	-13.13	
	-25179.58	-8.07	-25168.60	-11.65	-25158.21	-15.82	-25147.63	-19.80	-25123.59	-10.31	-25110.91	-12.18	-25104.27	-12.82	
	-25179.60	-8.09	-25168.84	-11.89	-25157.72	-15.33	-25147.21	-19.37	-25123.84	-10.56	-25110.52	-11.80	-25103.79	-12.34	
	-25179.58	-8.08	-25168.82	-11.87	-25157.86	-15.47	-25147.59	-19.76	-25123.44	-10.16	-25111.16	-12.43	-25104.18	-12.73	
	-25179.58	-8.07	-25169.02	-12.07	-25158.67	-16.28	-25148.03	-20.20	-25124.20	-10.92	-25111.45	-12.73	-25104.12	-12.67	
	-25179.60	-8.09	-25169.24	-12.29	-25158.52	-16.12	-25146.92	-19.09	-25123.93	-10.65	-25110.40	-11.68	-25104.11	-12.66	
	-25179.78	-8.28	-25168.83	-11.88	-25158.30	-15.91	-25147.30	-19.47	-25123.76	-10.48	-25110.38	-11.66	-25105.07	-13.62	
	-25179.58	-8.07	-25168.87	-11.92	-25158.37	-15.98	-25147.18	-19.35	-25123.11	-9.83	-25110.71	-11.98	-25104.98	-13.53	
	-25179.37	-7.87	-25168.60	-11.65	-25158.34	-15.95	-25147.41	-19.57	-25124.24	-10.96	-25110.55	-11.83	-25104.03	-12.59	
	-25179.55	-8.05	-25168.84	-11.89	-25158.29	-15.90	-25147.60	-19.76	-25124.11	-10.83	-25110.59	-11.86	-25104.18	-12.74	
	-25179.58	-8.07	-25168.80	-11.85	-25158.11	-15.71	-25147.10	-19.26	-25124.16	-10.88	-25110.95	-12.22	-25104.08	-12.64	
	-25179.37	-7.86	-25169.08	-12.13	-25157.91	-15.51	-25147.38	-19.54	-25124.22	-10.94	-25110.55	-11.83	-25103.54	-12.09	
	-25179.78	-8.28	-25169.01	-12.06	-25157.88	-15.49	-25147.22	-19.38	-25123.84	-10.56	-25110.85	-12.13	-25104.55	-13.10	
	-25179.57	-8.07	-25168.88	-11.93	-25158.37	-15.98	-25147.40	-19.56	-25124.61	-11.33	-25111.08	-12.35	-25103.57	-12.12	
	-25179.37	-7.87	-25168.80	-11.85	-25158.48	-16.09	-25147.46	-19.63	-25123.88	-10.60	-25111.32	-12.60	-25104.35	-12.91	
	-25179.78	-8.28	-25168.88	-11.93	-25158.31	-15.92	-25147.58	-19.74	-25124.33	-11.04	-25110.53	-11.81	-25104.46	-13.01	
	-25179.78	-8.27	-25168.83	-11.88	-25157.94	-15.54	-25147.48	-19.64	-25123.34	-10.06	-25111.26	-12.54	-25104.03	-12.58	
	-25179.37	-7.87	-25169.17	-12.22	-25158.08	-15.69	-25147.42	-19.59	-25123.59	-10.31	-25110.97	-12.25	-25104.22	-12.78	
Average energies	-25179.55	-8.04	-25168.89	-11.94	-25158.11	-15.72	-25147.41	-19.57	-25123.89	-10.61	-25110.82	-12.09	-25104.26	-12.82	

$Ca_{1-x}M_xZrTi_{2-2x}Al_{2x}O_7$ M=(Ce, Pu, Th, U)

Ti(1)Ti(1)

N.B. All stated energies are in eV

Ce

	ground state energy	solid solution energy												
concentration in solid solution (%)	3		6		9		12		15		18		21	
number of atoms	2		4		6		8		10		12		13	
	-25153.88	-0.50	-25116.21	4.49	-25077.28	10.73	-25041.73	13.60	-25005.68	16.97	-24972.58	17.39	-24947.74	25.89
	-25153.62	-0.24	-25115.75	4.95	-25074.65	13.36	-25042.13	13.20	-25005.94	16.72	-24970.31	19.66	-24948.15	25.47
	-25153.41	-0.03	-25116.28	4.42	-25079.60	8.41	-25040.27	15.07	-25006.49	16.16	-24968.04	21.93	-24949.87	23.76
	-25153.91	-0.53	-25117.13	3.57	-25077.47	10.55	-25044.98	10.35	-25006.18	16.47	-24969.26	20.70	-24948.70	24.93
	-25152.93	0.45	-25116.77	3.93	-25074.21	13.80	-25043.90	11.44	-25002.45	20.20	-24967.26	22.70	-24951.58	22.04
	-25154.18	-0.80	-25118.77	1.93	-25081.03	6.99	-25039.01	16.33	-25005.88	16.77	-24969.64	20.33	-24943.61	30.02
	-25153.36	0.02	-25113.58	7.12	-25081.65	6.37	-25041.03	14.31	-25007.47	15.19	-24967.13	22.84	-24950.04	23.59
	-25155.23	-1.85	-25119.14	1.56	-25080.46	7.55	-25037.95	17.39	-25004.77	17.88	-24972.02	17.95	-24953.35	20.28
	-25153.84	-0.46	-25118.47	2.23	-25078.25	9.77	-25041.54	13.79	-25003.61	19.04	-24967.08	22.89	-24954.37	19.25
	-25153.46	-0.08	-25115.30	5.40	-25082.14	5.88	-25043.12	12.22	-25005.45	17.20	-24962.90	27.07	-24950.32	23.31
	-25154.90	-1.52	-25113.53	7.17	-25076.69	11.32	-25040.76	14.58	-25008.33	14.32	-24967.15	22.82	-24950.41	23.22
	-25153.90	-0.52	-25118.06	2.64	-25081.21	6.81	-25043.68	11.65	-25006.15	16.50	-24971.98	17.99	-24950.06	23.57
	-25154.73	-1.35	-25116.72	3.98	-25078.92	9.09	-25039.63	15.71	-25007.75	14.90	-24967.36	22.61	-24950.84	22.79
	-25155.16	-1.78	-25116.91	3.79	-25079.23	8.78	-25043.91	11.43	-25008.33	14.32	-24968.35	21.62	-24945.63	27.99
	-25154.95	-1.57	-25116.64	4.06	-25077.76	10.26	-25043.06	12.27	-25004.79	17.86	-24971.07	18.90	-24949.46	24.17
	-25154.55	-1.17	-25118.30	2.40	-25078.01	10.00	-25043.44	11.90	-25002.81	19.84	-24965.47	24.50	-24951.86	21.77
	-25153.12	0.26	-25116.43	4.27	-25080.45	7.57	-25042.74	12.59	-25005.67	16.98	-24969.60	20.37	-24948.66	24.97
	-25152.86	0.52	-25116.46	4.24	-25077.82	10.20	-25041.20	14.13	-25007.45	15.20	-24970.38	19.59	-24948.94	24.69
	-25153.05	0.33	-25119.62	1.08	-25078.21	9.81	-25039.86	15.48	-25004.87	17.79	-24969.46	20.51	-24949.12	24.51
	-25155.45	-2.07	-25116.75	3.95	-25076.23	11.78	-25044.02	11.32	-25001.91	20.74	-24971.54	18.43	-24952.96	20.67
	-25153.76	-0.38	-25115.92	4.78	-25079.39	8.63	-25039.76	15.58	-25005.56	17.09	-24965.96	24.01	-24953.36	20.27
	-25154.41	-1.03	-25116.84	3.86	-25080.61	7.40	-25045.87	9.46	-25007.60	15.05	-24967.88	22.09	-24950.83	22.80
	-25153.45	-0.07	-25116.73	3.97	-25079.49	8.52	-25040.32	15.02	-25004.30	18.36	-24968.49	21.47	-24947.95	25.68
	-25154.37	-0.99	-25117.80	2.90	-25080.35	7.67	-25043.08	12.26	-25004.42	18.24	-24966.26	23.71	-24949.80	23.82
	-25153.87	-0.49	-25114.83	5.86	-25079.20	8.81	-25040.37	14.96	-25008.18	14.47	-24970.48	19.48	-24950.14	23.48
	-25154.13	-0.75	-25117.29	3.40	-25078.68	9.34	-25042.15	13.18	-25005.29	17.36	-24971.79	18.18	-24946.87	26.76
	-25154.09	-0.71	-25117.34	3.35	-25081.21	6.81	-25041.04	14.30	-25004.25	18.40	-24969.68	20.29	-24950.80	22.83
	-25153.74	-0.36	-25117.29	3.41	-25081.69	6.32	-25042.96	12.37	-25004.18	18.47	-24968.84	21.13	-24946.97	26.66
	-25154.01	-0.63	-25118.27	2.43	-25077.68	10.33	-25035.70	19.64	-25006.82	15.83	-24969.83	20.14	-24953.49	20.13

-25154.94	-1.56	-25117.98	2.72	-25078.10	9.92	-25042.06	13.28	-25005.32	17.33	-24832.60	157.37	-24946.14	27.48
-25154.13	-0.75	-25117.96	2.74	-25080.09	7.92	-25043.08	12.25	-25005.51	17.14	-24970.87	19.10	-24948.96	24.67
-25153.49	-0.11	-25116.02	4.68	-25081.14	6.88	-25044.44	10.89	-25006.22	16.43	-24971.66	18.31	-24948.54	25.09
-25154.53	-1.15	-25117.15	3.55	-25081.43	6.58	-25044.81	10.53	-25007.16	15.49	-24969.41	20.56	-24947.71	25.92
-25153.01	0.37	-25116.07	4.63	-25080.02	8.00	-25043.02	12.31	-25002.61	20.04	-24971.50	18.47	-24949.19	24.44
-25154.53	-1.15	-25116.05	4.65	-25079.53	8.49	-25043.02	12.31	-25005.69	16.96	-24971.42	18.55	-24952.27	21.35
-25154.87	-1.49	-25118.53	2.17	-25078.36	9.65	-25039.22	16.12	-25007.63	15.03	-24963.44	26.53	-24949.47	24.16
-25155.54	-2.16	-25116.21	4.49	-25080.00	8.01	-25043.28	12.05	-25002.44	20.22	-24968.85	21.12	-24951.70	21.93
-25154.16	-0.78	-25115.88	4.82	-25078.73	9.28	-25046.21	9.13	-25003.62	19.03	-24967.72	22.25	-24948.99	24.64
-25153.33	0.05	-25116.96	3.74	-25074.60	13.42	-25041.39	13.94	-25007.24	15.42	-24967.46	22.51	-24952.55	21.08
-25154.61	-1.23	-25118.55	2.15	-25080.10	7.91	-25041.43	13.91	-25007.81	14.84	-24970.17	19.80	-24950.30	23.33
-25155.10	-1.72	-25119.10	1.60	-25079.92	8.10	-25043.73	11.60	-25003.72	18.93	-24969.24	20.73	-24952.65	20.98
-25153.69	-0.31	-25114.47	6.23	-25073.28	14.73	-25040.18	15.16	-25005.57	17.08	-24965.96	24.01	-24954.10	19.53
-25153.68	-0.30	-25113.29	7.40	-25080.29	7.73	-25042.79	12.54	-25007.67	14.99	-24970.83	19.14	-24942.55	31.08
-25153.85	-0.47	-25118.59	2.11	-25079.35	8.67	-25044.79	10.54	-25003.53	19.12	-24969.06	20.91	-24945.82	27.80
-25153.40	-0.02	-25117.32	3.38	-25079.96	8.06	-25042.77	12.57	-25008.02	14.64	-24969.71	20.25	-24952.60	21.03
-25153.24	0.14	-25117.98	2.71	-25081.21	6.81	-25045.46	9.88	-25005.92	16.73	-24966.16	23.81	-24951.65	21.98
-25154.85	-1.47	-25114.88	5.81	-25077.61	10.41	-25042.10	13.24	-25007.25	15.40	-24970.79	19.18	-24946.53	27.09
-25155.33	-1.96	-25114.45	6.25	-25080.50	7.51	-25041.12	14.22	-25005.39	17.26	-24964.88	25.09	-24948.66	24.97
-25154.14	-0.77	-25111.99	8.70	-25083.07	4.95	-25038.78	16.55	-25009.59	13.07	-24965.82	24.15	-24950.82	22.81
-25153.76	-0.38	-25158.57	-37.87	-25076.91	11.11	-25042.91	12.43	-25004.99	17.66	-24969.39	20.57	-24952.43	21.20
-25154.93	-1.55	-25117.47	3.23	-25079.08	8.94	-25042.49	12.84	-25007.46	15.19	-24965.53	24.44	-24952.10	21.53
-25154.94	-1.56	-25117.04	3.66	-25079.59	8.42	-25041.59	13.75	-25004.63	18.02	-24969.87	20.10	-24950.06	23.57
-25154.72	-1.34	-25117.73	2.97	-25082.27	5.75	-25040.06	15.27	-25005.84	16.82	-24967.81	22.16	-24949.81	23.82
-25152.76	0.62	-25117.21	3.49	-25074.51	13.51	-25041.50	13.84	-25003.87	18.78	-24965.26	24.71	-24947.82	25.81
-25154.05	-0.67	-25117.00	3.69	-25078.58	9.43	-25038.34	17.00	-25006.03	16.62	-24969.62	20.34	-24954.16	19.47
-25153.43	-0.05	-25117.32	3.38	-25077.99	10.03	-25045.37	9.96	-25007.12	15.53	-24962.21	27.76	-24949.44	24.19
-25155.01	-1.64	-25117.54	3.16	-25081.30	6.72	-25040.66	14.68	-25008.09	14.56	-24964.90	25.07	-24951.41	22.22
-25156.46	-3.09	-25116.10	4.59	-25081.60	6.42	-25042.78	12.55	-25003.92	18.73	-24966.55	23.42	-24950.95	22.68
-25153.67	-0.29	-25116.35	4.35	-25079.12	8.89	-25044.99	10.35	-25004.12	18.53	-24965.92	24.05	-24946.40	27.23
-25154.35	-0.97	-25118.81	1.88	-25079.01	9.01	-25042.66	12.67	-25007.43	15.22	-24972.90	17.07	-24946.59	27.04
-25153.17	0.21	-25116.74	3.96	-25078.17	9.85	-25041.31	14.02	-25001.59	21.06	-24967.94	22.03	-24950.48	23.15
-25153.59	-0.21	-25118.11	2.59	-25079.08	8.94	-25039.52	15.81	-25008.22	14.43	-24968.83	21.14	-24949.15	24.48
-25155.53	-2.15	-25116.52	4.18	-25081.21	6.81	-25044.79	10.55	-25006.65	16.01	-24969.58	20.39	-24950.91	22.72
-25154.45	-1.07	-25118.18	2.52	-25076.29	11.72	-25043.68	11.66	-25005.15	17.50	-24971.72	18.25	-24944.31	29.32
-25154.73	-1.35	-25116.48	4.22	-25079.72	8.30	-25043.73	11.60	-25003.00	19.65	-24969.08	20.89	-24949.15	24.48
-25154.50	-1.12	-25116.65	4.04	-25080.94	7.08	-25044.22	11.11	-25005.16	17.49	-24969.48	20.49	-24950.42	23.20
-25154.41	-1.03	-25117.89	2.81	-25078.55	9.47	-25043.40	11.93	-25005.06	17.59	-24968.33	21.64	-24951.82	21.81
-25155.15	-1.77	-25117.50	3.20	-25079.86	8.15	-25039.03	16.30	-25005.53	17.13	-24971.01	18.96	-24950.77	22.86
-25153.81	-0.43	-25114.85	5.85	-25082.22	5.79	-25040.65	14.68	-25008.92	13.73	-24970.99	18.98	-24947.07	26.56
-25153.76	-0.38	-25117.65	3.05	-25078.90	9.12	-25043.43	11.91	-25006.13	16.52	-24965.39	24.58	-24943.80	29.82

-25154.90	-1.52	-25113.82	6.87	-25080.74	7.28	-25040.65	14.68	-25004.44	18.21	-24969.51	20.46	-24948.34	25.29
-25154.22	-0.84	-25115.79	4.91	-25076.54	11.48	-25044.24	11.10	-25005.95	16.70	-24966.98	22.99	-24954.56	19.06
-25154.52	-1.14	-25151.44	-30.74	-25079.85	8.17	-25042.86	12.47	-25006.36	16.29	-24971.03	18.94	-24949.42	24.20
-25154.36	-0.98	-25115.96	4.74	-25075.11	12.90	-25041.50	13.84	-25007.03	15.62	-24965.55	24.42	-24947.86	25.77
-25154.05	-0.67	-25117.57	3.13	-25077.73	10.29	-25041.93	13.40	-25004.00	18.66	-24971.20	18.77	-24954.89	18.73
-25154.98	-1.60	-25117.63	3.07	-25078.25	9.77	-25041.13	14.21	-25002.81	19.84	-24963.72	26.25	-24946.30	27.33
-25154.92	-1.54	-25118.48	2.22	-25079.88	8.13	-25041.80	13.53	-24996.54	26.11	-24968.82	21.15	-24950.16	23.47
-25152.37	1.00	-25115.19	5.50	-25078.73	9.29	-25040.39	14.94	-25006.68	15.97	-24965.96	24.01	-24951.99	21.64
-25154.68	-1.30	-25117.49	3.21	-25080.77	7.25	-25035.05	20.28	-25008.70	13.96	-24966.61	23.36	-24951.81	21.82
-25153.54	-0.16	-25117.95	2.75	-25079.20	8.81	-25045.22	10.11	-25006.05	16.61	-24967.98	21.99	-24952.47	21.16
-25154.57	-1.19	-25116.04	4.66	-25081.36	6.66	-25044.60	10.74	-25005.81	16.84	-24970.97	18.99	-24948.98	24.65
-25152.91	0.47	-25117.55	3.15	-25081.56	6.45	-25041.71	13.62	-25003.42	19.23	-24964.45	25.52	-24947.43	26.20
-25153.92	-0.54	-25118.33	2.36	-25080.51	7.51	-25044.81	10.52	-25005.28	17.38	-24972.17	17.80	-24948.15	25.47
-25154.51	-1.13	-25115.51	5.19	-25079.54	8.48	-25038.94	16.39	-25002.95	19.70	-24967.14	22.83	-24950.10	23.53
-25154.38	-1.00	-25116.52	4.17	-25078.93	9.08	-25042.72	12.62	-25007.44	15.21	-24964.81	25.16	-24950.09	23.54
-25154.70	-1.32	-25115.19	5.51	-25080.93	7.09	-25036.88	18.45	-25007.56	15.09	-24970.53	19.44	-24952.48	21.15
-25154.95	-1.57	-25116.65	4.05	-25080.71	7.31	-25043.32	12.01	-25005.93	16.73	-24970.56	19.41	-24945.95	27.67
-25153.89	-0.51	-25118.65	2.05	-25119.32	-31.31	-25039.89	15.44	-25005.84	16.81	-24970.33	19.64	-24944.73	28.90
-25154.45	-1.07	-25118.40	2.29	-25078.76	9.25	-25041.33	14.01	-25003.94	18.71	-24970.72	19.25	-24950.95	22.68
-25153.76	-0.38	-25116.88	3.82	-25080.42	7.59	-25046.11	9.23	-25004.50	18.15	-24969.17	20.80	-24951.86	21.77
-25153.89	-0.51	-25118.81	1.89	-25080.94	7.08	-25043.81	11.53	-25007.12	15.53	-24963.70	26.26	-24947.61	26.02
-25155.25	-1.87	-25118.81	1.89	-25079.02	8.99	-25043.10	12.23	-25009.14	13.51	-24970.14	19.83	-24950.18	23.45
-25154.64	-1.26	-25113.78	6.92	-25079.95	8.07	-25043.15	12.19	-25005.65	17.00	-24972.32	17.65	-24950.38	23.25
-25154.97	-1.59	-25117.26	3.44	-25079.72	8.29	-25041.29	14.04	-25007.87	14.78	-24967.82	22.15	-24951.34	22.28
-25154.62	-1.24	-25117.94	2.76	-25079.12	8.89	-25042.05	13.29	-25007.88	14.77	-24965.29	24.68	-24951.46	22.17
-25156.03	-2.65	-25117.34	3.36	-25077.51	10.51	-25040.31	15.02	-25006.10	16.55	-24967.01	22.96	-24950.28	23.34
-25155.23	-1.85	-25116.21	4.48	-25081.07	6.94	-25040.79	14.55	-25006.96	15.69	-24965.59	24.38	-24953.53	20.10
-25154.18	-0.80	-25117.52	3.17	-25079.10	8.91	-25041.17	14.16	-25007.01	15.64	-24965.99	23.98	-24949.77	23.86
-25153.92	-0.54	-25117.55	3.15	-25077.79	10.23	-25044.22	11.11	-25007.09	15.56	-24970.35	19.62	-24952.42	21.21
-25153.66	-0.28	-25118.85	1.84	-25077.16	10.86	-25042.38	12.95	-25002.29	20.36	-24970.40	19.57	-24943.38	30.25
-25153.50	-0.12	-25115.93	4.77	-25080.42	7.60	-25042.38	12.95	-25005.93	16.73	-24969.37	20.59	-24950.44	23.18
-25155.18	-1.80	-25118.49	2.21	-25081.14	6.88	-25041.42	13.91	-25007.52	15.13	-24967.80	22.17	-24953.34	20.29
-25154.33	-0.95	-25117.25	3.45	-25078.44	9.58	-25042.41	12.93	-24996.67	25.98	-24965.36	24.61	-24950.69	22.93
-25154.11	-0.73	-25115.71	4.98	-25080.89	7.13	-25041.37	13.97	-25003.45	19.20	-24965.40	24.57	-24951.24	22.39
-25154.42	-1.04	-25117.97	2.72	-25080.45	7.57	-25043.15	12.18	-25008.70	13.95	-24969.97	19.99	-24952.72	20.91
-25153.80	-0.42	-25116.89	3.80	-25079.90	8.12	-25042.91	12.42	-24998.88	23.77	-24969.06	20.91	-24950.34	23.29
-25152.90	0.48	-25118.10	2.60	-25078.82	9.19	-25043.40	11.93			-24966.94	23.03	-24951.98	21.65
-25154.62	-1.24	-25115.21	5.48	-25079.99	8.02	-25040.40	14.93			-24969.33	20.64		
-25154.36	-0.98	-25115.15	5.54	-25077.08	10.94	-25042.34	12.99			-24972.10	17.87		
-25153.56	-0.18	-25117.33	3.37	-25080.24	7.78	-25039.65	15.68						
-25153.94	-0.56	-25116.37	4.32	-25078.77	9.25	-25042.60	12.73						

	-25154.56	-1.18	-25118.24	2.46	-25080.07	7.95	-25042.81	12.53						
	-25154.01	-0.63	-25117.25	3.45			-25037.50	17.83						
	-25153.50	-0.12	-25116.17	4.52			-25038.99	16.34						
	-25154.09	-0.71	-25116.39	4.31			-25033.31	22.03						
	-25155.64	-2.27	-25117.98	2.71										
	-25154.37	-0.99												
	-25154.09	-0.71												
Average energies	-25154.22	-0.84	-25117.55	3.15	-25079.63	8.39	-25041.93	13.40	-25005.55	17.10	-24967.25	22.72	-24949.87	23.76

Pu

	ground state energy	solid solution energy												
concentration in solid solution (%)	3		6		9		12		15		18		21	
number of atoms	2		4		6		8		10		12		13	
	-25149.13	-1.74	-25074.43	34.30	-25064.51	5.55	-24985.99	45.41	-24978.56	14.16	-24929.65	24.40	-24878.36	56.36
	-25148.31	-0.92	-25075.26	33.47	-25062.19	7.87	-24987.81	43.58	-24982.38	10.34	-24937.96	16.09	-24877.37	57.36
	-25148.03	-0.63	-25071.21	37.51	-25061.15	8.91	-24985.24	46.15	-24976.78	15.94	-24936.45	17.60	-24879.86	54.87
	-25148.74	-1.34	-25070.73	38.00	-25059.10	10.96	-24986.16	45.23	-24976.73	15.99	-24939.13	14.93	-24883.05	51.68
	-25148.36	-0.97	-25074.99	33.74	-25061.84	8.22	-24983.46	47.93	-24974.58	18.14	-24934.63	19.42	-24881.34	53.38
	-25148.41	-1.01	-25072.17	36.56	-25063.77	6.29	-24984.28	47.11	-24976.76	15.97	-24938.90	15.15	-24877.25	57.47
	-25146.85	0.54	-25073.11	35.62	-25064.87	5.19	-24986.58	44.81	-24978.55	14.17	-24935.09	18.96	-24872.46	62.26
	-25148.64	-1.25	-25074.69	34.04	-25064.15	5.90	-24989.86	41.53	-24979.31	13.41	-24938.12	15.94	-24884.51	50.21
	-25149.09	-1.69	-25073.71	35.02	-25061.44	8.61	-24981.79	49.60	-24976.98	15.74	-24938.40	15.65	-24881.03	53.69
	-25149.52	-2.13	-25074.23	34.50	-25064.33	5.73	-24986.26	45.13	-24980.12	12.61	-24938.92	15.14	-24878.71	56.01
	-25149.29	-1.90	-25072.54	36.18	-25064.94	5.12	-24987.46	43.93	-24976.79	15.93	-24937.60	16.46	-24880.94	53.79
	-25148.73	-1.33	-25073.06	35.66	-25064.29	5.77	-24989.01	42.39	-24976.81	15.91	-24938.32	15.73	-24885.89	48.83
	-25147.29	0.10	-25072.48	36.24	-25060.80	9.26	-24981.98	49.41	-24979.12	13.61	-24937.82	16.24	-24876.54	58.18
	-25149.33	-1.94	-25071.68	37.04	-25062.17	7.89	-24985.24	46.15	-24978.15	14.58	-24934.50	19.56	-24879.29	55.43
	-25148.86	-1.46	-25072.84	35.89	-25063.58	6.48	-24990.31	41.08	-24980.56	12.16	-24937.33	16.73	-24879.43	55.29
	-25148.40	-1.01	-25070.41	38.31	-25064.43	5.63	-24984.55	46.84	-24976.97	15.75	-24934.73	19.33	-24879.05	55.68
	-25148.79	-1.40	-25075.20	33.53	-25061.35	8.71	-24988.91	42.48	-24978.69	14.03	-24939.80	14.26	-24879.96	54.77
	-25147.03	0.36	-25069.73	38.99	-25062.50	7.56	-24979.74	51.65	-24980.65	12.07	-24937.65	16.41	-24883.70	51.02
	-25148.22	-0.83	-25072.93	35.79	-25062.80	7.26	-24982.91	48.48	-24980.04	12.68	-24933.98	20.08	-24873.62	61.10
	-25148.51	-1.12	-25074.96	33.77	-25061.52	8.54	-24988.46	42.93	-24975.78	16.94	-24939.48	14.57	-24878.83	55.90
	-25147.87	-0.47	-25071.04	37.69	-25064.87	5.19	-24986.12	45.27	-24980.56	12.17	-24938.53	15.53	-24875.98	58.75
	-25146.93	0.46	-25072.64	36.09	-25063.81	6.24	-24988.53	42.87	-24978.08	14.64	-24939.15	14.91	-24884.06	50.66
	-25148.90	-1.51	-25074.17	34.56	-25060.28	9.78	-24986.84	44.55	-24978.63	14.09	-24934.73	19.32	-24880.58	54.14
	-25147.40	-0.01	-25072.50	36.23	-25062.43	7.63	-24989.82	41.58	-24981.32	11.41	-24938.27	15.79	-24878.57	56.15
	-25148.48	-1.08	-25071.50	37.23	-25062.92	7.13	-24983.91	47.48	-24979.48	13.25	-24936.04	18.02	-24879.25	55.48

-25149.42	-2.02	-25071.19	37.53	-25061.50	8.56	-24987.08	44.31	-24974.29	18.44	-24936.38	17.68	-24876.29	58.43
-25147.24	0.15	-25074.06	34.67	-25063.65	6.41	-24985.60	45.79	-24978.94	13.78	-24936.86	17.19	-24883.57	51.15
-25149.73	-2.33	-25072.91	35.82	-25065.11	4.95	-24981.95	49.45	-24975.11	17.61	-24930.14	23.91	-24881.13	53.59
-25147.50	-0.10	-25073.48	35.24	-25058.58	11.48	-24986.13	45.26	-24980.65	12.07	-24935.04	19.01	-24880.46	54.26
-25149.74	-2.34	-25073.05	35.67	-25064.19	5.87	-24983.32	48.07	-24979.54	13.18	-24933.27	20.79	-24876.16	58.56
-25147.37	0.02	-25073.78	34.94	-25064.57	5.48	-24981.82	49.57	-24976.56	16.17	-24937.88	16.18	-24881.97	52.75
-25149.15	-1.75	-25072.38	36.34	-25065.03	5.03	-24986.34	45.05	-24979.39	13.34	-24932.32	21.73	-24883.42	51.30
-25147.95	-0.56	-25071.22	37.51	-25061.56	8.50	-24988.96	42.43	-24976.96	15.76	-24935.07	18.99	-24881.00	53.72
-25149.60	-2.20	-25071.16	37.57	-25064.66	5.40	-24987.55	43.84	-24983.01	9.71	-24936.59	17.46	-24883.54	51.18
-25147.71	-0.31	-25073.77	34.96	-25061.47	8.59	-24985.48	45.91	-24977.07	15.66	-24939.20	14.86	-24880.70	54.02
-25148.18	-0.78	-25074.07	34.66	-25061.25	8.81	-24988.51	42.88	-24979.44	13.29	-24940.56	13.49	-24879.52	55.20
-25147.75	-0.36	-25071.64	37.09	-25062.47	7.59	-24983.81	47.58	-24979.31	13.41	-24938.12	15.93	-24879.35	55.38
-25147.46	-0.07	-25073.18	35.55	-25063.15	6.91	-24990.87	40.52	-24977.53	15.19	-24937.42	16.64	-24884.10	50.63
-25149.62	-2.23	-25073.15	35.58	-25064.58	5.48	-24986.80	44.59	-24977.80	14.92	-24938.49	15.57	-24886.29	48.43
-25146.97	0.42	-25073.11	35.62	-25064.41	5.64	-24985.69	45.70	-24979.93	12.79	-24935.87	18.19	-24875.60	59.12
-25149.60	-2.21	-25070.72	38.00	-25064.23	5.83	-24985.43	45.96	-24980.84	11.89	-24936.70	17.36	-24880.33	54.40
-25149.55	-2.16	-25071.64	37.09	-25063.35	6.71	-24989.02	42.37	-24977.64	15.08	-24932.12	21.93	-24877.76	56.96
-25147.65	-0.25	-25072.34	36.39	-25064.17	5.89	-24988.06	43.33	-24979.29	13.43	-24935.68	18.37	-24879.57	55.15
-25148.18	-0.79	-25072.78	35.94	-25061.44	8.62	-24984.69	46.70	-24978.51	14.21	-24933.27	20.79	-24882.86	51.87
-25147.41	-0.02	-25072.79	35.93	-25062.67	7.39	-24987.66	43.73	-24978.53	14.20	-24937.23	16.83	-24876.04	58.68
-25147.69	-0.30	-25072.18	36.55	-25062.31	7.75	-24987.31	44.08	-24978.51	14.21	-24937.39	16.66	-24881.18	53.55
-25147.75	-0.36	-25074.26	34.47	-25063.46	6.60	-24990.38	41.01	-24978.13	14.59	-24938.78	15.28	-24882.12	52.60
-25149.00	-1.61	-25072.08	36.65	-25062.64	7.42	-24986.05	45.35	-24981.86	10.86	-24939.36	14.70	-24879.40	55.32
-25148.01	-0.62	-25073.64	35.09	-25065.20	4.86	-24986.90	44.50	-24978.22	14.51	-24937.09	16.97	-24878.57	56.15
-25149.77	-2.37	-25072.97	35.76	-25060.69	9.37	-24980.31	51.08	-24979.56	13.16	-24937.98	16.07	-24877.34	57.38
-25149.41	-2.02	-25072.81	35.92	-25060.60	9.46	-24981.37	50.03	-24978.20	14.53	-24934.55	19.51	-24880.71	54.01
-25148.23	-0.84	-25073.89	34.83	-25061.14	8.92	-24987.71	43.69	-24974.42	18.30	-24938.55	15.50	-24871.60	63.12
-25146.53	0.87	-25074.52	34.20	-25063.39	6.67	-24985.97	45.42	-24978.53	14.20	-24935.48	18.58	-24881.76	52.96
-25148.36	-0.97	-25072.21	36.51	-25062.66	7.40	-24982.01	49.39	-24977.60	15.13	-24935.04	19.01	-24884.06	50.66
-25149.70	-2.30	-25070.31	38.41	-25063.06	7.00	-24989.81	41.58	-24979.78	12.95	-24938.16	15.90	-24873.47	61.26
-25148.22	-0.83	-25071.87	36.86	-25065.17	4.89	-24987.32	44.07	-24979.49	13.23	-24941.50	12.56	-24879.23	55.49
-25150.35	-2.95	-25070.49	38.24	-25064.38	5.68	-24985.24	46.16	-24980.77	11.95	-24939.86	14.19	-24880.25	54.47
-25147.99	-0.60	-25069.68	39.05	-25060.13	9.93	-24988.74	42.65	-24977.39	15.33	-24939.47	14.58	-24883.34	51.38
-25148.56	-1.17	-25074.27	34.46	-25064.40	5.66	-24990.27	41.12	-24980.02	12.70	-24942.00	12.05	-24883.32	51.40
-25148.32	-0.93	-25068.81	39.92	-25061.61	8.45	-24981.80	49.59	-24975.32	17.40	-24930.05	24.00	-24879.84	54.89
-25149.39	-2.00	-25072.65	36.08	-25060.86	9.20	-24985.49	45.90	-24980.25	12.47	-24932.67	21.38	-24882.62	52.10
-25149.53	-2.13	-25071.47	37.26	-25059.20	10.86	-24985.70	45.69	-24978.45	14.27	-24941.20	12.86	-24877.21	57.52
-25149.26	-1.87	-25071.68	37.05	-25063.54	6.52	-24986.61	44.78	-24980.40	12.32	-24934.24	19.81	-24881.35	53.37
-25148.44	-1.04	-25071.45	37.28	-25063.29	6.77	-24988.74	42.65	-24981.80	10.92	-24937.39	16.67	-24881.54	53.18
-25148.37	-0.98	-25073.92	34.81	-25066.28	3.78	-24985.60	45.79	-24981.17	11.56	-24940.92	13.14	-24884.65	50.07
-25148.48	-1.09	-25070.25	38.48	-25059.91	10.15	-24986.90	44.50	-24978.79	13.93	-24939.87	14.19	-24876.26	58.46

-25147.03	0.36	-25073.13	35.60	-25063.37	6.69	-24988.07	43.32	-24974.69	18.03	-24942.40	11.66	-24876.41	58.32
-25147.39	0.01	-25070.34	38.38	-25061.17	8.89	-24985.54	45.86	-24974.28	18.45	-24937.86	16.19	-24875.03	59.69
-25149.01	-1.62	-25074.05	34.68	-25064.55	5.50	-24985.82	45.57	-24980.24	12.48	-24938.35	15.71	-24882.30	52.42
-25147.23	0.16	-25071.20	37.52	-25064.16	5.90	-24988.03	43.36	-24977.86	14.86	-24939.31	14.74	-24882.81	51.91
-25149.15	-1.75	-25069.51	39.22	-25065.28	4.77	-24987.24	44.16	-24975.20	17.52	-24938.96	15.10	-24877.87	56.85
-25149.02	-1.63	-25070.11	38.62	-25063.10	6.95	-24987.28	44.11	-24982.02	10.70	-24936.83	17.23	-24881.84	52.88
-25148.85	-1.45	-25071.02	37.70	-25061.86	8.20	-24988.16	43.23	-24977.46	15.27	-24936.78	17.27	-24884.51	50.21
-25148.49	-1.10	-25074.90	33.82	-25062.88	7.18	-24982.84	48.55	-24980.20	12.52	-24937.20	16.86	-24877.77	56.95
-25148.37	-0.97	-25071.37	37.36	-25064.21	5.84	-24988.03	43.36	-24977.29	15.43	-24939.41	14.64	-24877.46	57.26
-25147.82	-0.42	-25074.20	34.52	-25064.37	5.68	-24986.65	44.74	-24978.71	14.01	-24937.34	16.71	-24879.94	54.78
-25148.87	-1.47	-25073.94	34.79	-25064.86	5.19	-24989.65	41.74	-24980.94	11.78	-24938.59	15.46	-24877.63	57.10
-25146.92	0.48	-25074.00	34.73	-25060.34	9.72	-24986.94	44.45	-24982.27	10.45	-24936.15	17.91	-24881.55	53.17
-25147.42	-0.03	-25072.21	36.52	-25062.88	7.18	-24987.42	43.97	-24980.13	12.60	-24938.72	15.34	-24870.41	64.31
-25147.97	-0.58	-25072.38	36.34	-25061.75	8.31	-24988.07	43.32	-24978.07	14.65	-24942.76	11.30	-24873.92	60.81
-25148.96	-1.57	-25073.34	35.38	-25062.04	8.02	-24989.31	42.08	-24975.80	16.92	-24936.81	17.25	-24876.99	57.73
-25148.96	-1.57	-25072.55	36.18	-25061.20	8.85	-24989.48	41.91	-24975.77	16.96	-24938.23	15.83	-24875.38	59.35
-25149.49	-2.09	-25074.42	34.30	-25065.13	4.93	-24987.13	44.26	-24980.89	11.83	-24937.64	16.41	-24880.31	54.41
-25147.62	-0.23	-25072.79	35.94	-25064.69	5.37	-24987.19	44.20	-24981.57	11.16	-24938.38	15.68	-24878.27	56.45
-25148.64	-1.24	-25073.63	35.09	-25063.53	6.53	-24986.39	45.00	-24979.60	13.13	-24940.49	13.57	-24883.98	50.75
-25149.66	-2.26	-25073.12	35.61	-25059.03	11.03	-24985.69	45.71	-24981.03	11.69	-24939.19	14.87	-24877.09	57.64
-25147.68	-0.29	-25074.23	34.50	-25063.82	6.23	-24984.74	46.65	-24981.15	11.57	-24938.06	15.99	-24879.06	55.66
-25149.24	-1.84	-25071.66	37.07	-25059.75	10.30	-24986.56	44.83	-24977.34	15.38	-24938.80	15.25	-24880.57	54.15
-25147.19	0.21	-25071.43	37.30	-25064.08	5.98	-24986.46	44.93	-24975.99	16.73	-24937.06	16.99	-24877.46	57.26
-25148.53	-1.13	-25073.76	34.96	-25062.68	7.38	-24985.14	46.25	-24983.67	9.05	-24937.21	16.84	-24882.52	52.20
-25149.23	-1.83	-25074.26	34.46	-25063.11	6.95	-24987.57	43.82	-24978.00	14.72	-24936.10	17.96	-24876.84	57.88
-25149.60	-2.21	-25071.16	37.57	-25062.43	7.63	-24988.41	42.98	-24979.06	13.66	-24937.60	16.46	-24879.26	55.46
-25149.26	-1.87	-25073.64	35.08	-25064.59	5.47	-24986.75	44.64	-24977.40	15.32	-24936.04	18.01	-24879.74	54.98
-25148.19	-0.80	-25071.40	37.32	-25064.62	5.44	-24982.77	48.62	-24978.13	14.59	-24938.01	16.04	-24873.55	61.18
-25149.47	-2.08	-25074.47	34.26	-25062.61	7.45	-24989.95	41.44	-24977.90	14.82	-24937.08	16.98	-24880.43	54.29
-25147.17	0.23	-25072.11	36.61	-25061.78	8.27	-24985.52	45.87	-24979.85	12.87	-24938.82	15.24	-24880.13	54.59
-25148.64	-1.25	-25074.16	34.57	-25064.97	5.09	-24986.44	44.95	-24976.41	16.31	-24938.88	15.17	-24880.40	54.32
-25147.18	0.21	-25072.62	36.11	-25064.49	5.57	-24985.30	46.09	-24978.16	14.57	-24937.79	16.27	-24879.12	55.60
-25148.85	-1.45	-25075.23	33.49	-25064.18	5.88	-24984.82	46.57	-24981.21	11.51	-24937.17	16.89	-24876.75	57.97
-25148.30	-0.91	-25067.40	41.33	-25064.05	6.01	-24985.58	45.81	-24982.26	10.46	-24937.37	16.68	-24883.36	51.36
-25149.68	-2.29	-25070.47	38.25	-25063.25	6.81	-24989.29	42.11	-24977.84	14.88	-24937.36	16.70	-24878.86	55.86
-25147.94	-0.54	-25071.53	37.20	-25063.23	6.83	-24984.74	46.65	-24981.29	11.43	-24933.78	20.28	-24877.51	57.21
-25146.20	1.20	-25071.40	37.33	-25062.08	7.98	-24985.29	46.10	-24978.66	14.06	-24937.23	16.83	-24879.77	54.95
-25148.20	-0.81	-25072.02	36.70	-25063.10	6.96	-24989.25	42.14	-24982.19	10.53	-24939.10	14.95	-24882.76	51.96
-25148.03	-0.64	-25072.85	35.87	-25060.63	9.43	-24986.41	44.98	-24972.50	20.22	-24937.15	16.91	-24881.41	53.31
-25148.08	-0.69	-25067.58	41.15	-25058.97	11.09	-24981.64	49.75	-24974.71	18.01	-24934.94	19.12	-24881.76	52.96
-25149.37	-1.97	-25071.92	36.80	-25064.60	5.46	-24987.95	43.44	-24981.07	11.66	-24936.65	17.41	-24881.91	52.81

	-25148.61	-1.21	-25073.37	35.36	-25063.28	6.78	-24985.12	46.27	-24981.93	10.79	-24938.72	15.34	-24879.35	55.37
	-25148.08	-0.69	-25071.34	37.39	-25064.45	5.61	-24988.49	42.90	-24978.46	14.26	-24937.40	16.65	-24880.40	54.32
	-25149.67	-2.27	-25071.25	37.48	-25065.23	4.83	-24983.84	47.55	-24976.55	16.17	-24938.04	16.02	-24884.21	50.52
	-25148.42	-1.03	-25071.45	37.27	-25064.04	6.02	-24983.12	48.27	-24978.36	14.36	-24934.58	19.48	-24880.74	53.98
	-25148.77	-1.38	-25072.77	35.96	-25064.80	5.26	-24988.88	42.51	-24980.13	12.59	-24936.37	17.69	-24881.88	52.85
	-25148.45	-1.06	-25072.24	36.48	-25063.29	6.77	-24991.77	39.62	-24980.91	11.81				
	-25147.10	0.30	-25073.28	35.45	-25062.58	7.48			-24977.72	15.01				
	-25148.66	-1.27	-25069.62	39.10	-25065.47	4.59								
	-25148.17	-0.77												
Average energies	-25148.42	-1.03	-25072.46	36.27	-25062.98	7.08	-24986.40	44.99	-24978.72	14.00	-24937.26	16.79	-24879.73	54.99

Th

	ground state energy	solid solution energy												
concentration in solid solution (%)	3		6		9		12		15		18		21	
number of atoms	2		4		6		8		10		12		13	
	-25153.91	-0.89	-25116.62	3.37	-25078.24	8.71	-25041.16	12.75	-25006.37	14.50	-24967.87	19.97	-24949.11	22.21
	-25153.14	-0.12	-25116.75	3.24	-25079.87	7.08	-25040.57	13.35	-25004.32	16.56	-24965.20	22.64	-24944.82	26.50
	-25153.68	-0.65	-25116.52	3.47	-25077.89	9.06	-25043.06	10.85	-25003.59	17.29	-24970.15	17.69	-24947.05	24.27
	-25155.10	-2.08	-25116.72	3.27	-25080.10	6.85	-25042.09	11.82	-24999.88	21.00	-24967.31	20.52	-24945.20	26.12
	-25152.92	0.11	-25116.17	3.82	-25077.13	9.82	-25042.37	11.54	-25004.12	16.75	-24961.25	26.59	-24949.55	21.77
	-25153.40	-0.37	-25116.37	3.61	-25079.83	7.12	-25042.64	11.28	-25002.48	18.40	-24966.11	21.73	-24947.53	23.79
	-25153.09	-0.06	-25117.52	2.46	-25078.17	8.78	-25042.13	11.78	-25001.28	19.59	-24965.70	22.14	-24942.61	28.71
	-25154.72	-1.70	-25115.83	4.16	-25118.82	-31.87	-25041.88	12.03	-25004.84	16.04	-24966.06	21.78	-24951.86	19.46
	-25155.33	-2.30	-25117.30	2.68	-25077.53	9.42	-25041.91	12.00	-25005.05	15.82	-24969.24	18.60	-24949.69	21.63
	-25152.48	0.55	-25114.63	5.36	-25078.18	8.77	-25043.61	10.30	-25002.19	18.69	-24965.42	22.42	-24950.11	21.21
	-25155.30	-2.28	-25117.21	2.78	-25079.12	7.83	-25040.82	13.09	-25000.45	20.43	-24967.90	19.94	-24947.43	23.89
	-25153.36	-0.33	-25117.00	2.99	-25076.70	10.25	-25041.68	12.23	-25000.08	20.79	-24967.75	20.09	-24946.00	25.32
	-25155.08	-2.05	-25116.71	3.27	-25078.51	8.44	-25043.20	10.72	-25005.35	15.52	-24965.23	22.61	-24950.80	20.52
	-25150.92	2.11	-25115.80	4.19	-25074.40	12.55	-25040.68	13.23	-25005.47	15.41	-24964.50	23.34	-24942.87	28.45
	-25153.44	-0.41	-25118.24	1.75	-25078.27	8.68	-25042.51	11.40	-25005.27	15.61	-24966.09	21.75	-24949.58	21.74
	-25153.95	-0.93	-25114.27	5.71	-25077.51	9.44	-25038.89	15.02	-25006.81	14.06	-24963.06	24.78	-24940.91	30.41
	-25154.30	-1.28	-25117.43	2.56	-25080.38	6.57	-25043.97	9.95	-25001.08	19.80	-24960.76	27.08	-24949.43	21.89
	-25154.81	-1.78	-25116.63	3.35	-25079.42	7.53	-25042.33	11.58	-25003.92	16.95	-24962.82	25.02	-24944.73	26.59
	-25153.21	-0.18	-25115.82	4.16	-25075.31	11.64	-25037.02	16.89	-25005.03	15.84	-24969.78	18.06	-24946.68	24.64
	-25153.57	-0.54	-25116.20	3.79	-25080.08	6.87	-25038.93	14.99	-25004.30	16.58	-24970.70	17.13	-24950.47	20.85
	-25154.88	-1.85	-25115.85	4.13	-25079.76	7.19	-25041.60	12.31	-25001.54	19.34	-24961.12	26.72	-24947.91	23.41

-25153.97	-0.94	-25114.33	5.66	-25079.96	6.99	-25040.01	13.91	-25002.65	18.23	-24967.36	20.48	-24949.86	21.46
-25154.03	-1.01	-25117.59	2.39	-25079.21	7.74	-25041.34	12.57	-25004.73	16.14	-24965.31	22.53	-24950.17	21.15
-25153.22	-0.19	-25115.47	4.51	-25080.00	6.95	-25041.09	12.82	-25001.58	19.30	-24968.72	19.12	-24945.97	25.35
-25154.98	-1.95	-25118.00	1.99	-25078.92	8.03	-25040.70	13.21	-25004.70	16.17	-24957.87	29.97	-24944.57	26.75
-25154.39	-1.37	-25116.16	3.83	-25079.82	7.13	-25039.79	14.12	-25004.29	16.58	-24964.97	22.87	-24950.85	20.47
-25154.14	-1.11	-25116.73	3.26	-25081.11	5.84	-25041.39	12.52	-25007.76	13.12	-24968.55	19.29	-24949.20	22.12
-25154.82	-1.79	-25115.60	4.39	-25079.27	7.68	-25038.90	15.01	-25002.85	18.02	-24967.30	20.54	-24951.02	20.30
-25153.28	-0.25	-25117.35	2.64	-25080.12	6.83	-25041.17	12.75	-24995.44	25.44	-24964.95	22.88	-24948.70	22.62
-25153.23	-0.21	-25117.98	2.00	-25078.99	7.96	-25040.06	13.85	-25004.20	16.68	-24966.32	21.51	-24948.49	22.83
-25154.16	-1.13	-25118.43	1.55	-25075.61	11.34	-25041.48	12.43	-25006.49	14.39	-24968.13	19.71	-24941.58	29.74
-25152.16	0.87	-25116.40	3.59	-25080.14	6.81	-25038.68	15.23	-25005.11	15.77	-24962.97	24.87	-24948.56	22.76
-25153.87	-0.85	-25117.11	2.88	-25079.25	7.70	-25040.59	13.32	-25005.92	14.95	-24969.23	18.61	-24949.47	21.85
-25153.91	-0.89	-25112.22	7.77	-25077.93	9.02	-25041.41	12.51	-25004.80	16.07	-24967.22	20.62	-24948.13	23.19
-25153.36	-0.34	-25115.34	4.65	-25079.51	7.44	-25039.60	14.31	-25006.39	14.49	-24965.25	22.58	-24951.09	20.23
-25153.47	-0.44	-25116.83	3.15	-25078.43	8.52	-25041.63	12.28	-25006.71	14.17	-24965.62	22.22	-24950.43	20.89
-25154.07	-1.04	-25114.88	5.11	-25079.95	7.00	-25041.33	12.58	-25006.50	14.38	-24968.23	19.61	-24945.37	25.95
-25154.18	-1.15	-25115.60	4.39	-25079.49	7.46	-25043.59	10.32	-25002.87	18.00	-24966.85	20.99	-24944.99	26.33
-25154.15	-1.13	-25114.44	5.55	-25076.71	10.24	-25043.22	10.69	-25004.17	16.71	-24964.16	23.68	-24951.22	20.10
-25153.33	-0.30	-25116.20	3.78	-25078.29	8.66	-25040.70	13.22	-25007.14	13.73	-24964.14	23.70	-24944.82	26.50
-25154.18	-1.15	-25116.96	3.03	-25077.48	9.47	-25042.85	11.06	-25001.48	19.39	-24967.65	20.18	-24942.64	28.68
-25153.40	-0.38	-25115.81	4.18	-25079.85	7.10	-25037.06	16.85	-25007.68	13.20	-24962.54	25.30	-24950.03	21.29
-25153.45	-0.43	-25115.08	4.91	-25078.68	8.27	-25039.51	14.41	-25005.95	14.93	-24965.62	22.22	-24948.70	22.62
-25153.72	-0.70	-25117.01	2.98	-25078.35	8.60	-25037.01	16.91	-25005.78	15.09	-24965.08	22.76	-24949.96	21.36
-25153.65	-0.62	-25117.34	2.64	-25079.14	7.81	-25041.26	12.65	-25002.40	18.48	-24967.98	19.86	-24947.07	24.25
-25154.67	-1.65	-25113.44	6.55	-25078.54	8.41	-25042.79	11.12	-25001.71	19.16	-24967.12	20.72	-24949.17	22.15
-25154.08	-1.05	-25114.67	5.32	-25075.31	11.64	-25044.42	9.50	-25001.51	19.37	-24966.14	21.70	-24948.09	23.23
-25153.67	-0.65	-25114.22	5.77	-25078.96	7.99	-25040.36	13.55	-25001.91	18.96	-24967.40	20.44	-24947.14	24.18
-25154.09	-1.07	-25115.59	4.40	-25078.78	8.17	-25043.73	10.18	-25004.67	16.21	-24965.02	22.82	-24946.50	24.82
-25153.15	-0.12	-25117.12	2.87	-25078.47	8.48	-25042.33	11.58	-25004.29	16.59	-24968.11	19.73	-24938.50	32.82
-25155.10	-2.08	-25116.71	3.28	-25080.69	6.26	-25039.91	14.00	-25004.50	16.37	-24969.58	18.26	-24946.35	24.97
-25154.23	-1.21	-25114.79	5.20	-25078.39	8.56	-25042.25	11.66	-25005.60	15.28	-24961.73	26.11	-24944.92	26.40
-25154.25	-1.22	-25115.54	4.45	-25078.91	8.04	-25037.50	16.42	-25006.55	14.33	-24965.74	22.10	-24942.23	29.09
-25151.97	1.05	-25117.31	2.67	-25078.37	8.58	-25042.26	11.65	-25005.64	15.23	-24966.16	21.67	-24948.38	22.94
-25153.31	-0.28	-25117.17	2.82	-25080.52	6.43	-25042.55	11.36	-25003.52	17.35	-24966.14	21.70	-24952.27	19.05
-25153.71	-0.68	-25116.23	3.76	-25079.75	7.20	-25041.96	11.95	-25003.24	17.63	-24958.34	29.49	-24947.27	24.05
-25154.20	-1.18	-25115.71	4.28	-25077.46	9.49	-25035.09	18.82	-25005.65	15.22	-24964.09	23.75	-24947.88	23.44
-25152.78	0.24	-25116.91	3.07	-25078.80	8.15	-25038.61	15.31	-25005.23	15.65	-24967.83	20.01	-24950.64	20.68
-25153.34	-0.32	-25118.36	1.63	-25077.68	9.27	-25038.64	15.28	-24998.73	22.14	-24967.77	20.07	-24948.35	22.97
-25153.95	-0.93	-25117.03	2.96	-25080.69	6.26	-25041.52	12.39	-25004.25	16.62	-24967.44	20.40	-24949.46	21.86
-25153.32	-0.30	-25112.91	7.08	-25078.31	8.64	-25038.90	15.01	-25000.88	20.00	-24966.42	21.42	-24946.31	25.01
-25154.62	-1.60	-25117.17	2.82	-25079.32	7.63	-25042.82	11.09	-25007.80	13.07	-24967.44	20.40	-24948.25	23.07

-25153.96	-0.94	-25117.76	2.23	-25078.37	8.58	-25038.23	15.68	-25002.80	18.08	-24964.77	23.07	-24950.00	21.32
-25152.73	0.29	-25116.03	3.96	-25078.52	8.43	-25041.76	12.15	-25001.62	19.26	-24966.59	21.25	-24944.66	26.66
-25155.12	-2.10	-25116.32	3.66	-25077.99	8.96	-25041.89	12.02	-25004.48	16.40	-24959.99	27.85	-24949.02	22.30
-25152.68	0.35	-25117.10	2.88	-25079.26	7.69	-25039.51	14.40	-25001.98	18.90	-24964.95	22.89	-24945.52	25.80
-25155.02	-1.99	-25117.62	2.37	-25079.72	7.23	-25042.93	10.98	-25004.99	15.88	-24964.01	23.83	-24948.70	22.62
-25153.45	-0.43	-25116.94	3.05	-25078.15	8.80	-25041.83	12.09	-24999.54	21.33	-24963.81	24.03	-24944.25	27.07
-25154.18	-1.15	-25116.84	3.15	-25078.02	8.93	-25040.15	13.77	-25007.89	12.99	-24965.11	22.73	-24946.36	24.96
-25154.44	-1.42	-25114.90	5.09	-25078.19	8.76	-25040.75	13.16	-25003.14	17.73	-24966.55	21.29	-24949.76	21.56
-25153.13	-0.10	-25116.82	3.17	-25079.95	7.00	-25042.58	11.34	-25003.12	17.76	-24968.68	19.16	-24949.04	22.28
-25154.52	-1.49	-25117.81	2.18	-25081.59	5.36	-25043.44	10.48	-25006.07	14.81	-24967.17	20.67	-24948.49	22.83
-25154.19	-1.16	-25115.95	4.04	-25080.05	6.90	-25040.74	13.18	-25004.88	15.99	-24963.63	24.21	-24949.80	21.52
-25154.04	-1.02	-25115.34	4.65	-25072.63	14.32	-25042.79	11.13	-25002.80	18.08	-24966.69	21.14	-24952.62	18.70
-25154.07	-1.05	-25116.31	3.68	-25079.02	7.93	-25038.59	15.33	-25001.54	19.34	-24967.78	20.06	-24949.32	22.00
-25154.20	-1.17	-25116.07	3.92	-25081.75	5.20	-25041.16	12.76	-25002.23	18.65	-24966.41	21.43	-24944.27	27.05
-25154.47	-1.45	-25115.15	4.84	-25076.61	10.34	-25038.64	15.28	-25003.41	17.47	-24968.36	19.48	-24948.49	22.83
-25154.30	-1.27	-25116.75	3.24	-25081.04	5.91	-25043.04	10.88	-25005.26	15.62	-24961.10	26.74	-24946.88	24.44
-25154.24	-1.21	-25115.66	4.33	-25080.70	6.25	-25041.89	12.02	-25003.52	17.35	-24969.31	18.53	-24949.96	21.35
-25153.52	-0.50	-25116.83	3.16	-25079.42	7.53	-25039.69	14.23	-25000.70	20.18	-24967.55	20.28	-24948.20	23.12
-25154.04	-1.01	-25116.15	3.84	-25077.69	9.26	-25044.74	9.17	-25002.73	18.15	-24966.89	20.95	-24951.38	19.94
-25153.20	-0.17	-25116.33	3.66	-25078.95	8.00	-25039.74	14.17	-25004.89	15.99	-24968.47	19.37	-24940.72	30.60
-25153.93	-0.90	-25114.02	5.97	-25074.85	12.10	-25041.83	12.08	-25002.60	18.28	-24968.90	18.93	-24947.20	24.12
-25154.37	-1.35	-25115.51	4.47	-25079.46	7.49	-25040.94	12.97	-25004.50	16.37	-24963.66	24.18	-24942.28	29.04
-25154.52	-1.49	-25117.45	2.54	-25079.04	7.91	-25040.74	13.17	-25005.39	15.49	-24963.88	23.96	-24936.46	34.86
-25152.56	0.47	-25116.39	3.60	-25079.96	7.00	-25041.90	12.01	-25006.19	14.69	-24964.66	23.18	-24948.24	23.08
-25152.41	0.61	-25114.18	5.81	-25076.05	10.90	-25039.24	14.67	-25004.42	16.46	-24967.81	20.02	-24942.95	28.37
-25154.61	-1.58	-25113.69	6.30	-25077.09	9.86	-25042.25	11.66	-25007.95	12.92	-24969.14	18.70	-24944.62	26.70
-25152.70	0.32	-25116.02	3.96	-25077.99	8.96	-25037.15	16.76	-25001.46	19.42	-24963.34	24.50	-24950.45	20.87
-25153.66	-0.63	-25116.19	3.80	-25080.08	6.87	-25039.13	14.78	-25006.64	14.23	-24961.03	26.81	-24946.86	24.46
-25153.68	-0.65	-25116.16	3.83	-25079.62	7.33	-25042.08	11.84	-25003.84	17.04	-24966.47	21.37	-24984.46	-13.14
-25153.62	-0.59	-25116.73	3.26	-25077.92	9.03	-25040.61	13.30	-25004.14	16.73	-24967.08	20.76	-24947.88	23.44
-25153.88	-0.86	-25114.24	5.75	-25079.34	7.61	-25041.23	12.68	-25005.94	14.94	-24967.22	20.62	-24946.24	25.08
-25152.72	0.30	-25116.46	3.53	-25080.17	6.78	-25042.20	11.71	-25006.39	14.49	-24966.26	21.58	-24940.86	30.46
-25154.35	-1.32	-25114.57	5.42	-25079.95	7.00	-25043.23	10.69	-24998.97	21.91	-24964.15	23.69	-24950.68	20.64
-25153.84	-0.82	-25116.88	3.11	-25077.89	9.06	-25042.37	11.54	-25000.10	20.77	-24966.08	21.76	-24949.45	21.87
-25153.45	-0.43	-25116.20	3.79	-25079.07	7.88	-25040.51	13.41	-25004.82	16.05	-24968.66	19.18	-24942.80	28.52
-25153.54	-0.51	-25117.12	2.87	-25079.67	7.28	-25041.77	12.14	-25003.54	17.34	-24966.63	21.21	-24947.38	23.94
-25153.50	-0.48	-25118.89	1.09	-25077.86	9.09	-25041.36	12.55	-25002.88	18.00	-24967.94	19.90	-24949.76	21.56
-25154.23	-1.21	-25115.55	4.44	-25078.63	8.32	-25042.47	11.44	-25004.08	16.80	-24964.14	23.70	-24947.39	23.93
-25153.90	-0.88	-25114.99	5.00	-25076.90	10.05	-25042.42	11.49	-25003.01	17.87	-24967.82	20.02	-24947.11	24.21
-25154.07	-1.04	-25116.88	3.11	-25079.28	7.67	-25038.10	15.82	-25006.46	14.42	-24967.21	20.63	-24944.74	26.58
-25154.24	-1.21	-25115.71	4.28	-25079.01	7.94	-25039.95	13.97	-25007.71	13.17	-24965.43	22.41	-24946.39	24.93

	-25153.92	-0.90	-25117.53	2.46	-25079.81	7.14	-25036.99	16.92	-25005.37	15.50	-24967.16	20.68	-24949.37	21.95
	-25154.27	-1.24	-25115.27	4.72	-25077.83	9.12	-25042.69	11.23	-25003.31	17.57	-24966.64	21.19	-24946.08	25.24
	-25153.19	-0.16	-25117.66	2.33	-25078.48	8.47	-25040.71	13.20	-25000.36	20.52	-24967.11	20.72	-24943.87	27.45
	-25153.80	-0.78	-25116.01	3.98	-25078.69	8.26	-25038.62	15.29	-25005.20	15.68	-24967.44	20.40	-24946.54	24.78
	-25153.45	-0.42	-25113.66	6.33	-25075.85	11.10	-25039.15	14.76	-25003.13	17.75	-24968.29	19.54	-24945.22	26.10
	-25153.90	-0.88	-25114.37	5.62	-25078.92	8.03	-25041.72	12.19	-25003.79	17.08	-24967.56	20.27	-24943.01	28.31
	-25153.62	-0.60	-25116.98	3.01	-25076.66	10.29	-25039.86	14.05	-25004.37	16.50	-24961.12	26.72	-24950.45	20.87
	-25154.07	-1.04	-25116.29	3.70	-25079.27	7.68			-25001.82	19.06	-24968.14	19.70		
	-25153.81	-0.79	-25115.93	4.06	-25075.68	11.27			-25004.37	16.51				
	-25153.72	-0.69	-25117.74	2.24	-25078.95	8.00			-25006.50	14.37				
	-25153.02	0.00	-25117.49	2.49					-25004.92	15.96				
	-25153.88	-0.86	-25116.44	3.55					-25001.38	19.50				
	-25153.75	-0.72							-25006.06	14.82				
	-25153.17	-0.14												
	-25154.61	-1.58												
	-25154.39	-1.37												
Average energies	-25153.82	-0.79	-25116.20	3.79	-25079.01	7.94	-25040.99	12.93	-25003.93	16.95	-24966.01	21.83	-24947.59	23.73

U

	ground state energy	solid solution energy												
concentration in solid solution (%)	3		6		9		12		15		18		21	
number of atoms	2		4		6		8		10		12		13	
	-25156.26	1.79	-25126.65	3.38	-25095.44	6.57	-25062.24	11.75	-25025.98	19.99	-24997.81	20.14	-24977.46	26.48
	-25157.70	0.34	-25125.08	4.94	-25093.82	8.18	-25062.52	11.46	-25026.82	19.14	-25000.57	17.38	-24973.96	29.98
	-25158.71	-0.67	-25124.81	5.22	-25095.27	6.73	-25062.56	11.43	-25031.39	14.58	-24991.32	26.63	-24975.24	28.70
	-25158.91	-0.87	-25124.00	6.02	-25092.99	9.01	-25061.01	12.98	-25027.72	18.25	-24993.74	24.21	-24977.54	26.41
	-25148.31	9.73	-25125.38	4.64	-25092.16	9.85	-25062.99	11.00	-25030.48	15.49	-25001.72	16.23	-24981.69	22.25
	-25157.41	0.63	-25125.67	4.36	-25091.07	10.93	-25062.98	11.01	-25030.25	15.72	-24998.02	19.93	-24981.33	22.61
	-25156.65	1.39	-25123.08	6.94	-25093.03	8.98	-25058.06	15.93	-25025.80	20.17	-24995.20	22.75	-24984.83	19.11
	-25149.06	8.98	-25123.05	6.98	-25092.82	9.19	-25063.43	10.56	-25030.18	15.79	-25000.43	17.52	-24981.20	22.75
	-25158.67	-0.62	-25108.92	21.10	-25095.76	6.25	-25058.48	15.51	-25028.60	17.37	-24999.10	18.86	-24982.08	21.86
	-25148.43	9.61	-25123.98	6.04	-25093.35	8.66	-25060.84	13.15	-25029.54	16.43	-24999.00	18.95	-24980.23	23.71
	-25156.05	1.99	-25124.58	5.44	-25093.64	8.37	-25059.49	14.50	-25030.64	15.33	-24994.85	23.11	-24984.02	19.92
	-25158.98	-0.93	-25124.19	5.84	-25092.94	9.07	-25062.61	11.38	-25028.51	17.46	-24993.87	24.08	-24973.82	30.12
	-25155.02	3.03	-25124.89	5.13	-25093.51	8.50	-25060.08	13.91	-25029.05	16.91	-24997.95	20.00	-24978.73	25.22
	-25157.78	0.27	-25125.34	4.68	-25091.16	10.84	-25061.68	12.30	-25032.90	13.07	-24998.66	19.29	-24981.57	22.37
	-25158.72	-0.68	-25127.92	2.10	-25093.51	8.49	-25062.37	11.61	-25025.61	20.35	-24998.65	19.30	-24978.48	25.46
	-25158.82	-0.78	-25124.92	5.10	-25096.10	5.91	-25062.63	11.36	-25032.62	13.35	-24998.08	19.87	-24981.38	22.56

-25148.10	9.95	-25125.55	4.47	-25093.70	8.31	-25056.74	17.25	-25030.98	14.99	-24996.68	21.27	-24981.61	22.33
-25158.20	-0.16	-25126.13	3.90	-25096.17	5.84	-25061.10	12.89	-25028.17	17.80	-24998.03	19.92	-24975.47	28.47
-25157.90	0.14	-25122.36	7.66	-25095.49	6.52	-25062.21	11.78	-25026.41	19.56	-24994.21	23.74	-24972.24	31.70
-25158.65	-0.60	-25124.25	5.77	-25093.88	8.13	-25061.39	12.60	-25028.17	17.80	-25000.36	17.59	-24968.86	35.08
-25159.16	-1.11	-25125.96	4.07	-25094.55	7.45	-25058.43	15.56	-25025.80	20.17	-24997.13	20.82	-24980.24	23.70
-25159.30	-1.26	-25126.82	3.21	-25093.22	8.78	-25060.84	13.15	-25028.40	17.57	-24998.73	19.23	-24983.03	20.91
-25157.32	0.72	-25125.67	4.36	-25096.04	5.97	-25061.20	12.78	-25028.98	16.99	-24996.20	21.75	-24982.27	21.67
-25148.24	9.81	-25123.73	6.29	-25092.98	9.02	-25059.00	14.99	-25030.73	15.24	-24996.44	21.51	-24981.72	22.22
-25157.87	0.17	-25124.01	6.01	-25093.85	8.16	-25061.43	12.56	-25028.69	17.28	-24997.87	20.08	-24983.45	20.49
-25158.00	0.05	-25123.30	6.72	-25093.72	8.29	-25062.80	11.19	-25025.89	20.08	-24995.73	22.22	-24980.65	23.30
-25157.28	0.77	-25125.24	4.79	-25091.08	10.92	-25061.95	12.03	-25028.23	17.74	-24996.39	21.56	-24981.36	22.58
-25158.43	-0.38	-25124.06	5.97	-25091.63	10.38	-25062.98	11.01	-25028.71	17.26	-24997.91	20.04	-24981.74	22.20
-25156.44	1.60	-25122.13	7.89	-25093.00	9.00	-25060.81	13.17	-25029.81	16.16	-24999.45	18.50	-24980.07	23.87
-25158.05	0.00	-25123.90	6.13	-25094.43	7.57	-25065.31	8.68	-25030.31	15.66	-24996.71	21.24	-24980.99	22.95
-25157.71	0.33	-25125.02	5.00	-25094.93	7.07	-25059.68	14.31	-25032.15	13.82	-24999.16	18.79	-24982.80	21.14
-25158.36	-0.31	-25125.84	4.18	-25093.22	8.78	-25061.70	12.29	-25029.38	16.59	-24995.63	22.32	-24977.87	26.07
-25158.14	-0.10	-25125.90	4.12	-25094.41	7.60	-25059.39	14.59	-25029.36	16.61	-24994.35	23.60	-24982.75	21.19
-25157.21	0.84	-25125.87	4.16	-25095.00	7.00	-25061.79	12.20	-25033.64	12.33	-25000.88	17.07	-24983.01	20.93
-25157.69	0.35	-25126.40	3.63	-25096.37	5.64	-25063.28	10.71	-25026.80	19.17	-24998.85	19.10	-24981.58	22.36
-25157.90	0.14	-25126.00	4.03	-25092.36	9.65	-25062.35	11.64	-25025.97	20.00	-24995.39	22.56	-24977.84	26.10
-25158.76	-0.71	-25124.23	5.80	-25094.95	7.06	-25059.85	14.13	-25029.03	16.94	-24993.62	24.34	-24983.80	20.14
-25149.93	8.12	-25126.06	3.97	-25093.88	8.13	-25059.03	14.96	-25027.97	18.00	-25000.11	17.84	-24979.86	24.09
-25156.29	1.75	-25123.72	6.30	-25095.54	6.47	-25061.55	12.43	-25030.50	15.47	-24996.01	21.94	-24982.47	21.47
-25147.73	10.31	-25122.81	7.22	-25092.91	9.09	-25061.54	12.45	-25030.48	15.49	-24999.33	18.62	-24978.97	24.97
-25156.72	1.32	-25127.35	2.68	-25094.05	7.96	-25060.11	13.88	-25029.84	16.13	-24996.85	21.11	-24982.45	21.49
-25156.94	1.11	-25125.92	4.10	-25091.55	10.46	-25058.46	15.53	-25021.05	24.92	-25000.10	17.85	-24978.72	25.22
-25147.77	10.28	-25124.51	5.51	-25096.73	5.28	-25060.67	13.32	-25027.25	18.72	-24999.87	18.08	-24980.68	23.26
-25158.71	-0.66	-25125.58	4.45	-25094.08	7.92	-25061.46	12.53	-25033.18	12.79	-24995.02	22.93	-24978.97	24.97
-25158.79	-0.74	-25124.94	5.08	-25091.94	10.07	-25058.94	15.05	-25029.99	15.98	-24996.86	21.09	-24983.94	20.01
-25148.65	9.40	-25123.31	6.71	-25092.82	9.19	-25059.54	14.45	-25022.09	23.88	-24988.48	29.48	-24979.64	24.30
-25158.73	-0.69	-25123.81	6.21	-25093.43	8.57	-25064.63	9.35	-25031.70	14.27	-24996.99	20.97	-24981.07	22.87
-25158.31	-0.27	-25125.04	4.98	-25094.85	7.15	-25062.21	11.78	-25031.98	13.99	-24996.01	21.94	-24983.92	20.02
-25158.81	-0.76	-25125.69	4.33	-25093.87	8.14	-25053.76	20.22	-25030.39	15.58	-24995.75	22.20	-24978.68	25.26
-25158.28	-0.23	-25126.28	3.74	-25094.03	7.98	-25048.17	25.82	-25029.16	16.81	-24999.46	18.49	-24981.28	22.66
-25147.70	10.35	-25126.05	3.98	-25089.28	12.73	-25060.49	13.50	-25029.27	16.70	-24992.14	25.81	-24980.21	23.73
		-25125.99	4.04	-25094.88	7.13	-25060.52	13.46	-25031.98	13.99	-24996.69	21.26	-24979.87	24.07
		-25125.08	4.94	-25093.90	8.11	-25060.57	13.41	-25029.53	16.44	-24999.94	18.01	-24978.09	25.85
		-25124.63	5.40	-25093.19	8.81	-25063.75	10.24	-25024.99	20.98	-24997.90	20.05	-24985.06	18.88
		-25125.11	4.91			-25061.36	12.63	-25029.75	16.21	-25000.73	17.23	-24978.48	25.46
		-25125.97	4.06	-25093.65	8.36	-25062.91	11.08	-25029.18	16.79	-24992.98	24.97	-24985.59	18.35
		-25125.51	4.52	-25093.50	8.51	-25062.80	11.18	-25028.04	17.93	-24996.43	21.52	-24975.78	28.16

			-25115.85	14.17	-25092.82	9.18	-25059.57	14.42	-25025.89	20.08	-24996.45	21.50	-24982.60	21.34
			-25124.76	5.26	-25093.30	8.70	-25059.27	14.71	-25029.14	16.83	-24999.46	18.49	-24977.93	26.02
			-25125.71	4.32	-25093.98	8.03	-25061.81	12.18	-25028.24	17.73	-24996.52	21.43	-24979.01	24.93
			-25123.80	6.22	-25092.07	9.93	-25061.65	12.33			-24998.50	19.45	-24981.17	22.77
			-25124.38	5.65	-25094.47	7.53	-25062.27	11.71			-24999.62	18.33	-24978.67	25.28
			-25124.87	5.15	-25095.45	6.56					-24997.00	20.95	-24979.41	24.53
			-25125.37	4.66	-25092.41	9.59					-25001.58	16.37	-24980.75	23.19
			-25123.08	6.95	-25092.65	9.36					-24997.71	20.24		
			-25124.92	5.11	-25094.64	7.37					-24997.00	20.95		
			-25125.57	4.46							-24997.43	20.52		
			-25127.97	2.05							-24998.15	19.80		
											-24994.31	23.64		
											-24995.17	22.78		
											-24997.49	20.46		
											-24999.55	18.40		
											-24997.04	20.91		
											-24997.71	20.25		
											-24999.26	18.69		
											-24995.84	22.11		
											-24997.87	20.08		
											-24996.71	21.24		
Average energies	-25153.82	-0.79	-25116.20	3.79	-25079.01	7.94	-25040.99	12.93	-25003.93	16.95	-24966.01	21.83	-24947.59	23.73

$Ca_{1-x}M_xZrTi_{2-2x}Al_{2x}O_7$ M=(Ce, Pu, Th, U)

Ti(2)Ti(2)

N.B. All stated energies are in eV

Ce

	ground state energy	solid solution energy													
concentration in solid solution (%)	3		6		9		12		15		18		21		
number of atoms	2		4		6		8		10		12		13		
	-25159.61	-6.23	-25128.62	-7.93	-25098.35	-10.33	-25064.60	-9.26	-25034.56	-11.91	-25006.68	-16.71	-24965.69	7.94	
	-25161.00	-7.62	-25128.81	-8.11	-25094.29	-6.27	-25068.61	-13.28	-25035.83	-13.18	-25004.75	-14.78	-24985.81	-12.18	
	-25158.98	-5.60	-25127.06	-6.36	-25097.81	-9.79	-25066.09	-10.75	-25032.79	-10.14	-25006.83	-16.86	-24965.51	8.12	
	-25157.99	-4.61	-25127.56	-6.86	-25095.62	-7.60	-25065.02	-9.69	-25030.49	-7.83	-25004.32	-14.35	-24987.97	-14.34	
	-25158.70	-5.32	-25127.45	-6.75	-25096.66	-8.65	-25064.01	-8.67	-25036.68	-14.03	-25005.17	-15.21	-24961.01	12.62	
	-25159.30	-5.92	-25127.36	-6.67	-25096.86	-8.85	-25067.81	-12.48	-25031.19	-8.54	-25003.33	-13.36	-24988.62	-14.99	
	-25159.61	-6.23	-25126.86	-6.17	-25097.08	-9.07	-25066.67	-11.34	-25034.81	-12.16	-25006.87	-16.91	-24992.02	-18.40	
	-25160.27	-6.89	-25125.83	-5.14	-25095.57	-7.56	-25065.65	-10.32	-25034.20	-11.55	-25001.63	-11.66	-24989.43	-15.80	
	-25159.48	-6.10	-25128.87	-8.17	-25096.51	-8.49	-25063.34	-8.01	-25035.26	-12.61	-25007.71	-17.75	-24964.16	9.47	
	-25159.55	-6.17	-25128.03	-7.33	-25097.72	-9.71	-25063.31	-7.98	-25034.90	-12.25	-25005.47	-15.50	-24965.46	8.17	
	-25159.99	-6.61	-25127.98	-7.29	-25097.75	-9.73	-25065.42	-10.09	-25035.28	-12.63	-25006.25	-16.29	-24981.56	-7.94	
	-25159.81	-6.43	-25127.20	-6.51	-25096.94	-8.92	-25064.73	-9.40	-25034.22	-11.56	-25007.36	-17.39	-24968.15	5.48	
	-25159.03	-5.65	-25128.37	-7.67	-25098.07	-10.06	-25066.53	-11.20	-25036.62	-13.97	-25002.82	-12.85	-24987.71	-14.08	
	-25160.32	-6.94	-25129.42	-8.72	-25096.76	-8.75	-25065.59	-10.25	-25035.16	-12.51	-25002.16	-12.20	-24987.62	-13.99	
	-25159.43	-6.05	-25128.89	-8.19	-25096.26	-8.24	-25067.17	-11.84	-25035.60	-12.95	-25005.71	-15.74	-24990.55	-16.92	
	-25159.87	-6.49	-25128.62	-7.93	-25097.38	-9.36	-25067.01	-11.68	-25032.96	-10.31	-25000.83	-10.86	-24988.80	-15.17	
	-25159.94	-6.56	-25127.84	-7.14	-25096.23	-8.22	-25066.58	-11.24	-25035.53	-12.88	-25005.63	-15.66	-24987.61	-13.98	
	-25158.53	-5.15	-25128.34	-7.64	-25095.31	-7.29	-25063.58	-8.25	-25037.11	-14.46	-24999.82	-9.85	-24990.71	-17.08	
	-25160.81	-7.43	-25127.38	-6.69	-25096.67	-8.66	-25064.34	-9.01	-25033.09	-10.44	-25006.08	-16.11	-24986.88	-13.25	
	-25159.76	-6.38	-25127.85	-7.16	-25095.82	-7.81	-25065.24	-9.91	-25033.89	-11.24	-25004.15	-14.18	-24986.53	-12.90	
	-25159.41	-6.03	-25128.59	-7.90	-25095.37	-7.36	-25066.54	-11.21	-25033.50	-10.85	-25005.40	-15.43	-24989.50	-15.87	
	-25160.01	-6.63	-25123.67	-2.98	-25097.42	-9.41	-25066.12	-10.79	-25036.20	-13.55	-24999.34	-9.37	-24987.40	-13.77	
	-25159.30	-5.92	-25127.20	-6.50	-25092.44	-4.43	-25062.89	-7.56	-25037.75	-15.10	-24998.58	-8.61	-24990.75	-17.12	
	-25160.50	-7.12	-25129.43	-8.74	-25095.61	-7.60	-25065.22	-9.89	-25035.77	-13.12	-25004.43	-14.46	-24988.11	-14.49	
	-25159.38	-6.00	-25129.55	-8.85	-25097.45	-9.44	-25063.69	-8.36	-25034.81	-12.16	-25004.31	-14.34	-24986.67	-13.05	
	-25159.55	-6.18	-25128.14	-7.44	-25096.12	-8.10	-25066.42	-11.09	-25033.79	-11.14	-24999.97	-10.00	-24987.32	-13.69	
	-25159.46	-6.08	-25124.40	-3.70	-25096.90	-8.89	-25065.82	-10.48	-25034.93	-12.28	-25002.65	-12.68	-24993.72	-20.09	
	-25158.69	-5.31	-25125.70	-5.00	-25097.15	-9.13	-25066.68	-11.35	-25035.50	-12.85	-25004.08	-14.11	-24988.09	-14.46	
	-25160.01	-6.63	-25128.26	-7.56	-25095.66	-7.65	-25058.94	-3.61	-25033.67	-11.02	-25006.33	-16.36	-24966.09	7.54	

-25160.13	-6.75	-25128.80	-8.10	-25097.03	-9.01	-25066.58	-11.24	-25032.38	-9.73	-25003.72	-13.75	-24986.51	-12.88
-25158.70	-5.32	-25128.50	-7.80	-25098.09	-10.08	-25061.86	-6.52	-25034.95	-12.30	-25000.07	-10.10	-24988.15	-14.52
-25158.98	-5.60	-25128.04	-7.35	-25096.30	-8.28	-25066.97	-11.63	-25037.10	-14.44	-25005.32	-15.35	-24986.50	-12.87
-25159.67	-6.29	-25128.88	-8.18	-25096.06	-8.05	-25066.53	-11.19	-25032.57	-9.92	-25005.40	-15.43	-24989.85	-16.22
-25159.81	-6.43	-25127.91	-7.22	-25096.32	-8.31	-25065.80	-10.46	-25030.37	-7.72	-25005.30	-15.33	-24988.02	-14.40
-25159.88	-6.50	-25126.69	-5.99	-25096.14	-8.12	-25065.82	-10.48	-25033.26	-10.61	-25006.07	-16.10	-24987.49	-13.87
-25159.16	-5.78	-25126.47	-5.77	-25095.34	-7.32	-25066.91	-11.57	-25033.70	-11.05	-25004.60	-14.64	-24984.59	-10.96
-25159.88	-6.50	-25128.27	-7.57	-25098.12	-10.11	-25065.17	-9.84	-25035.21	-12.56	-25002.44	-12.47	-24987.10	-13.47
-25159.69	-6.31	-25130.30	-9.60	-25097.90	-9.88	-25064.02	-8.68	-25035.72	-13.07	-25003.51	-13.54	-24989.15	-15.52
-25160.66	-7.28	-25129.57	-8.88	-25096.19	-8.18	-25061.45	-6.12	-25037.95	-15.30	-25003.56	-13.59	-24990.34	-16.71
-25159.50	-6.12	-25127.50	-6.80	-25096.79	-8.78	-25064.75	-9.42	-25035.97	-13.31	-25001.67	-11.70	-24989.40	-15.77
-25159.67	-6.29	-25129.12	-8.42	-25096.81	-8.79	-25065.78	-10.44	-25036.02	-13.37	-25002.60	-12.63	-24987.42	-13.79
-25159.44	-6.06	-25129.09	-8.39	-25098.82	-10.81	-25064.12	-8.79	-25033.62	-10.97	-25003.38	-13.41	-24990.61	-16.98
-25161.23	-7.85	-25126.91	-6.21	-25098.31	-10.29	-25068.34	-13.01	-25035.58	-12.93	-25002.51	-12.54	-24991.17	-17.55
-25160.17	-6.79	-25128.15	-7.45	-25096.81	-8.80	-25066.58	-11.25	-25036.08	-13.43	-25004.65	-14.68	-24989.65	-16.02
-25159.98	-6.60	-25128.91	-8.21	-25096.30	-8.28	-25066.10	-10.77	-25033.92	-11.27	-25006.58	-16.61	-24990.04	-16.42
-25160.11	-6.73	-25127.68	-6.98	-25099.31	-11.30	-25063.96	-8.63	-25034.86	-12.20	-25006.05	-16.08	-24990.98	-17.35
-25159.30	-5.92	-25128.16	-7.46	-25096.66	-8.65	-25067.36	-12.03	-25036.82	-14.16	-25006.72	-16.75	-24990.22	-16.59
-25159.79	-6.41	-25129.55	-8.85	-25096.89	-8.88	-25065.30	-9.97	-25036.18	-13.53	-25005.11	-15.14	-24989.17	-15.54
-25159.66	-6.28	-25127.94	-7.25	-25097.34	-9.33	-25063.24	-7.90	-25035.05	-12.40	-25002.57	-12.60	-24991.23	-17.60
-25159.66	-6.28	-25128.29	-7.59	-25096.87	-8.86	-25065.13	-9.79	-25034.63	-11.98	-25008.25	-18.28	-24984.77	-11.14
-25159.29	-5.91	-25128.18	-7.49	-25098.44	-10.42	-25066.13	-10.80	-25035.09	-12.44	-25001.51	-11.54	-24990.65	-17.02
-25159.50	-6.12	-25128.08	-7.38	-25098.32	-10.31	-25062.02	-6.69	-25035.05	-12.40	-25004.92	-14.95	-24988.01	-14.38
-25158.26	-4.88	-25127.01	-6.31	-25097.08	-9.06	-25066.22	-10.89	-25032.87	-10.22	-25005.85	-15.88	-24989.04	-15.42
-25160.18	-6.80	-25129.06	-8.36	-25096.58	-8.56	-25067.56	-12.22	-25034.34	-11.68	-25003.22	-13.25	-24987.49	-13.87
-25159.32	-5.94	-25129.33	-8.63	-25095.96	-7.94	-25068.86	-13.52	-25034.45	-11.80	-25005.23	-15.26	-24991.92	-18.29
-25159.75	-6.37	-25128.83	-8.13	-25096.60	-8.59	-25063.49	-8.16	-25035.91	-13.26	-25005.25	-15.28	-24989.19	-15.56
-25161.11	-7.73	-25125.72	-5.02	-25098.88	-10.86	-25068.56	-13.23	-25035.62	-12.97	-25002.88	-12.91	-24988.40	-14.77
-25160.10	-6.72	-25128.11	-7.41	-25096.66	-8.65	-25064.88	-9.54	-25033.44	-10.79	-25007.55	-17.58	-24987.24	-13.61
-25160.05	-6.67	-25127.06	-6.36	-25093.71	-5.70	-25067.34	-12.00	-25037.42	-14.77	-25006.00	-16.03	-24985.56	-11.94
-25160.07	-6.69	-25127.38	-6.68	-25099.22	-11.21	-25062.61	-7.28	-25030.16	-7.51	-25005.37	-15.40	-24988.53	-14.90
-25158.33	-4.95	-25126.62	-5.92	-25095.72	-7.70	-25063.76	-8.42	-25034.08	-11.43	-25006.13	-16.16	-24989.94	-16.31
-25160.78	-7.40	-25128.25	-7.56	-25096.38	-8.36	-25065.84	-10.51	-25034.83	-12.18	-25000.78	-10.81	-24987.68	-14.06
-25159.09	-5.71	-25127.24	-6.54	-25094.31	-6.29	-25068.30	-12.97	-25034.24	-11.59	-25003.12	-13.15	-24982.34	-8.71
-25158.59	-5.21	-25127.32	-6.62	-25093.64	-5.62	-25068.14	-12.81	-25036.89	-14.24	-25003.19	-13.22	-24988.41	-14.78
-25160.44	-7.06	-25126.26	-5.56	-25097.15	-9.14	-25065.27	-9.94	-25035.12	-12.47	-25003.24	-13.27	-24992.44	-18.81
-25158.32	-4.94	-25127.53	-6.83	-25095.18	-7.16	-25065.58	-10.24	-25036.75	-14.10	-25005.92	-15.95	-24987.27	-13.64
-25160.01	-6.63	-25127.27	-6.57	-25095.36	-7.35	-25067.20	-11.86	-25035.68	-13.03	-25005.33	-15.36	-24990.07	-16.44
-25161.42	-8.04	-25128.42	-7.72	-25097.92	-9.90	-25064.41	-9.08	-25036.98	-14.33	-25005.63	-15.66	-24984.57	-10.94
-25160.30	-6.92	-25128.96	-8.26	-25095.59	-7.57	-25061.88	-6.55	-25031.82	-9.17	-25001.48	-11.51	-24990.34	-16.71
-25159.73	-6.35	-25128.63	-7.93	-25094.59	-6.57	-25066.66	-11.33	-25032.23	-9.58	-24998.82	-8.85	-24986.98	-13.35

-25159.36	-5.98	-25127.18	-6.48	-25097.28	-9.27	-25066.44	-11.11	-25035.85	-13.20	-24994.49	-4.52	-24989.31	-15.68
-25159.06	-5.68	-25127.80	-7.10	-25095.89	-7.88	-25064.75	-9.42	-25036.17	-13.51	-25005.86	-15.89	-24984.24	-10.62
-25159.47	-6.09	-25127.17	-6.47	-25098.11	-10.09	-25066.96	-11.62	-25033.33	-10.68	-25006.06	-16.09	-24989.69	-16.06
-25160.65	-7.27	-25128.38	-7.68	-25094.74	-6.72	-25065.99	-10.65	-25029.83	-7.18	-24997.68	-7.71	-24987.94	-14.31
-25159.07	-5.69	-25125.73	-5.03	-25097.96	-9.95	-25067.01	-11.68	-25036.13	-13.48	-25006.01	-16.05	-24989.18	-15.56
-25159.76	-6.38	-25127.52	-6.82	-25097.35	-9.33	-25064.98	-9.64	-25034.52	-11.87	-25005.31	-15.34	-24991.63	-18.00
-25159.68	-6.30	-25127.06	-6.36	-25097.45	-9.44	-25064.78	-9.44	-25036.62	-13.97	-25002.15	-12.18	-24987.76	-14.13
-25159.79	-6.41	-25129.02	-8.33	-25094.54	-6.53	-25065.03	-9.70	-25033.05	-10.40	-25005.44	-15.47	-24990.35	-16.73
-25160.26	-6.88	-25127.68	-6.99	-25098.12	-10.11	-25063.85	-8.52	-25033.10	-10.45	-25001.99	-12.02	-24990.58	-16.95
-25161.40	-8.02	-25127.24	-6.55	-25097.68	-9.66	-25066.05	-10.71	-25035.86	-13.21	-25001.52	-11.55	-24988.81	-15.18
-25159.45	-6.07	-25128.35	-7.65	-25097.81	-9.80	-25066.04	-10.71	-25033.95	-11.30	-25002.92	-12.95	-24991.38	-17.75
-25160.26	-6.88	-25125.67	-4.97	-25092.37	-4.36	-25062.40	-7.06	-25032.92	-10.27	-25008.53	-18.56	-24986.95	-13.32
-25158.24	-4.86	-25128.73	-8.03	-25096.74	-8.73	-25065.05	-9.71	-25031.31	-8.66	-25004.81	-14.84	-24981.69	-8.06
-25159.22	-5.84	-25129.09	-8.39	-25097.33	-9.31	-25064.76	-9.42	-25037.88	-15.23	-25004.89	-14.92	-24989.03	-15.40
-25157.99	-4.61	-25128.39	-7.70	-25094.24	-6.22	-25066.60	-11.27	-25033.56	-10.90	-25000.06	-10.09	-24986.61	-12.98
-25159.26	-5.88	-25129.04	-8.34	-25095.94	-7.93	-25065.83	-10.50	-25033.14	-10.49	-25004.58	-14.61	-24987.71	-14.08
-25159.64	-6.26	-25128.00	-7.30	-25095.59	-7.57	-25066.41	-11.07	-25035.33	-12.68	-25006.06	-16.09	-24989.65	-16.02
-25159.74	-6.37	-25127.87	-7.17	-25096.29	-8.28	-25065.91	-10.58	-25035.70	-13.05	-25005.23	-15.26	-24987.52	-13.90
-25160.03	-6.65	-25129.06	-8.36	-25096.09	-8.07	-25064.30	-8.97	-25034.07	-11.42	-25003.91	-13.94	-24988.36	-14.73
-25159.79	-6.41	-25129.57	-8.87	-25097.73	-9.72	-25062.83	-7.50	-25032.81	-10.16	-24998.73	-8.77	-24989.84	-16.21
-25159.19	-5.81	-25128.99	-8.29	-25095.67	-7.66	-25058.18	-2.84	-25034.15	-11.50	-25002.20	-12.23	-24987.04	-13.41
-25160.57	-7.19	-25128.06	-7.36	-25097.60	-9.58	-25064.99	-9.66	-25034.70	-12.05	-25004.48	-14.51	-24983.08	-9.45
-25158.69	-5.31	-25125.57	-4.87	-25095.88	-7.87	-25066.21	-10.88	-25037.69	-15.04	-25000.68	-10.71	-24987.56	-13.94
-25159.51	-6.13	-25126.91	-6.22	-25097.10	-9.08	-25067.88	-12.55	-25032.28	-9.63	-25004.05	-14.08	-24985.89	-12.26
-25159.36	-5.99	-25128.11	-7.41	-25097.42	-9.41	-25067.16	-11.82	-25037.08	-14.43	-25001.20	-11.23	-24987.92	-14.29
-25160.07	-6.69	-25127.47	-6.77	-25097.43	-9.42	-25065.20	-9.87	-25036.49	-13.84	-25004.87	-14.90	-24990.06	-16.43
-25159.28	-5.90	-25126.90	-6.21	-25096.75	-8.74	-25067.12	-11.79	-25035.54	-12.89	-25006.39	-16.42	-24990.42	-16.79
-25158.32	-4.94	-25129.44	-8.75	-25095.80	-7.79	-25066.84	-11.50	-25035.10	-12.44	-25006.56	-16.59	-24987.17	-13.54
-25160.22	-6.84	-25128.34	-7.64	-25096.38	-8.36	-25063.68	-8.35	-25035.17	-12.52	-25003.38	-13.41	-24987.55	-13.92
-25158.38	-5.00	-25127.97	-7.28	-25097.69	-9.67	-25064.70	-9.37	-25035.96	-13.31	-25007.23	-17.27	-24990.09	-16.46
-25158.25	-4.87	-25128.03	-7.33	-25097.09	-9.08	-25065.93	-10.60	-25033.62	-10.97	-25003.85	-13.88	-24988.46	-14.83
-25159.12	-5.74	-25127.30	-6.60	-25092.44	-4.42	-25065.00	-9.67	-25036.81	-14.16	-25007.52	-17.55	-24989.48	-15.85
-25159.89	-6.51	-25128.12	-7.42	-25096.54	-8.53	-25068.36	-13.03	-25033.86	-11.21	-24998.91	-8.94	-24986.21	-12.58
-25159.77	-6.39	-25128.99	-8.29	-25097.35	-9.33	-25066.41	-11.08	-25034.88	-12.23	-25006.58	-16.61	-24986.87	-13.25
-25159.34	-5.96	-25126.68	-5.98	-25092.56	-4.54	-25067.08	-11.74	-25034.74	-12.09	-25006.05	-16.08	-24983.79	-10.16
-25160.38	-7.00	-25128.28	-7.59	-25096.16	-8.15	-25063.92	-8.59	-25036.64	-13.99	-25004.93	-14.96	-24990.92	-17.29
-25160.69	-7.31	-25128.92	-8.22	-25097.36	-9.34	-25063.00	-7.67	-25035.11	-12.46	-25005.92	-15.95	-24990.71	-17.08
-25159.35	-5.97	-25127.94	-7.24	-25098.65	-10.63	-25066.43	-11.10	-25033.86	-11.20	-25004.91	-14.94	-24988.19	-14.56
-25160.07	-6.69	-25126.06	-5.36	-25098.19	-10.17	-25065.49	-10.16	-25033.07	-10.42	-25005.11	-15.14	-24987.12	-13.49
-25160.54	-7.16	-25127.62	-6.92	-25096.16	-8.14	-25066.19	-10.85	-25035.75	-13.10	-25006.28	-16.31	-24988.15	-14.52
-25161.02	-7.64	-25127.42	-6.72	-25097.38	-9.36	-25062.57	-7.23	-25034.70	-12.05	-25004.48	-14.51	-24989.50	-15.87

	-25159.85	-6.47	-25127.16	-6.46	-25097.74	-9.72	-25065.84	-10.50	-25036.15	-13.50	-25005.14	-15.17	-24987.11	-13.48
	-25159.76	-6.38	-25128.45	-7.75	-25096.48	-8.47	-25065.42	-10.09	-25034.35	-11.69	-25004.26	-14.29	-24989.13	-15.50
	-25159.71	-6.33	-25128.73	-8.03	-25097.49	-9.47	-25064.76	-9.43	-25033.77	-11.12	-25005.83	-15.86	-24989.90	-16.27
	-25158.70	-5.32	-25127.58	-6.88	-25099.08	-11.07	-25065.89	-10.55	-25035.70	-13.05	-25003.59	-13.62		
	-25160.23	-6.85	-25125.78	-5.08	-25096.36	-8.34	-25064.06	-8.73	-25034.40	-11.75	-25003.34	-13.37		
	-25160.42	-7.04	-25126.62	-5.92	-25097.75	-9.73	-25066.84	-11.51			-24996.64	-6.67		
	-25159.24	-5.86	-25128.07	-7.37	-25093.90	-5.89	-25065.28	-9.95						
	-25157.65	-4.27	-25128.04	-7.34	-25092.80	-4.79	-25066.59	-11.25						
	-25160.12	-6.74												
Average energies	-25161.42	-8.04	-25130.30	-9.60	-25099.31	-11.30	-25068.86	-13.52	-25037.95	-15.30	-25008.53	-18.56	-24993.72	-20.09

Pu

	ground state energy	solid solution energy												
concentration in solid solution (%)	3		6		9		12		15		18		21	
number of atoms	2		4		6		8		10		12		13	
	-25154.68	-7.28	-25116.62	-7.89	-25076.05	-5.99	-25041.58	-10.19	-25005.95	-13.23	-24970.61	-16.55	-24944.90	-10.18
	-25153.68	-6.28	-25116.83	-8.11	-25080.58	-10.52	-25042.83	-11.44	-25006.10	-13.38	-24968.87	-14.82	-24951.49	-16.76
	-25154.42	-7.03	-25116.83	-8.10	-25078.46	-8.40	-25042.63	-11.24	-25004.05	-11.32	-24967.31	-13.25	-24949.18	-14.45
	-25155.14	-7.74	-25114.66	-5.94	-25079.64	-9.59	-25043.67	-12.28	-25006.60	-13.88	-24967.58	-13.52	-24950.67	-15.94
	-25153.10	-5.70	-25115.22	-6.50	-25076.92	-6.86	-25041.86	-10.47	-25007.10	-14.38	-24969.65	-15.60	-24949.71	-14.99
	-25154.19	-6.80	-25116.18	-7.45	-25077.70	-7.64	-25040.77	-9.38	-25004.15	-11.43	-24968.75	-14.70	-24951.85	-17.12
	-25152.57	-5.18	-25114.97	-6.25	-25076.57	-6.51	-25040.58	-9.19	-25005.31	-12.58	-24969.98	-15.93	-24950.47	-15.75
	-25152.80	-5.41	-25114.99	-6.26	-25080.48	-10.42	-25041.98	-10.58	-25006.37	-13.64	-24967.44	-13.38	-24951.23	-16.51
	-25154.31	-6.92	-25117.72	-8.99	-25079.10	-9.04	-25041.98	-10.59	-25005.52	-12.80	-24967.67	-13.62	-24951.85	-17.13
	-25153.77	-6.38	-25116.22	-7.50	-25076.79	-6.73	-25040.25	-8.86	-25006.37	-13.65	-24970.22	-16.16	-24949.94	-15.22
	-25153.72	-6.33	-25115.27	-6.55	-25079.57	-9.51	-25042.77	-11.38	-25005.89	-13.17	-24969.56	-15.50	-24950.51	-15.79
	-25154.44	-7.04	-25115.51	-6.78	-25079.39	-9.33	-25042.70	-11.30	-25004.74	-12.02	-24966.68	-12.63	-24951.01	-16.29
	-25154.84	-7.45	-25116.24	-7.52	-25080.31	-10.25	-25044.43	-13.04	-25007.39	-14.66	-24970.25	-16.20	-24952.61	-17.89
	-25153.48	-6.08	-25115.67	-6.94	-25079.70	-9.64	-25039.48	-8.09	-25005.99	-13.27	-24967.37	-13.32	-24951.18	-16.45
	-25154.70	-7.30	-25117.39	-8.66	-25079.80	-9.74	-25041.79	-10.40	-25003.85	-11.13	-24968.90	-14.84	-24950.51	-15.79
	-25154.59	-7.19	-25114.78	-6.06	-25077.57	-7.51	-25040.22	-8.82	-25006.31	-13.59	-24968.06	-14.00	-24950.67	-15.95
	-25153.00	-5.60	-25117.57	-8.85	-25080.12	-10.06	-25043.88	-12.49	-25004.35	-11.62	-24967.56	-13.51	-24948.35	-13.62
	-25154.52	-7.12	-25116.46	-7.73	-25077.63	-7.57	-25041.11	-9.72	-25005.46	-12.74	-24969.22	-15.17	-24950.07	-15.35
	-25153.14	-5.75	-25114.79	-6.06	-25078.49	-8.43	-25044.24	-12.84	-25002.01	-9.29	-24965.68	-11.63	-24951.10	-16.37
	-25153.89	-6.49	-25116.21	-7.48	-25080.21	-10.15	-25042.38	-10.98	-25004.75	-12.03	-24970.85	-16.80	-24949.08	-14.35
	-25154.20	-6.81	-25116.42	-7.69	-25079.57	-9.52	-25042.47	-11.08	-25006.81	-14.08	-24970.28	-16.23	-24952.28	-17.55
	-25153.86	-6.47	-25117.38	-8.65	-25079.94	-9.88	-25041.60	-10.20	-25006.54	-13.81	-24968.55	-14.50	-24950.90	-16.18
	-25153.93	-6.53	-25115.51	-6.79	-25076.81	-6.75	-25042.63	-11.23	-25006.53	-13.81	-24969.71	-15.65	-24950.64	-15.91

-25152.98	-5.58	-25116.45	-7.72	-25076.55	-6.49	-25043.85	-12.46	-25005.74	-13.02	-24966.35	-12.30	-24950.69	-15.97
-25153.59	-6.20	-25115.04	-6.31	-25075.30	-5.24	-25042.10	-10.71	-25004.67	-11.94	-24969.53	-15.47	-24946.56	-11.84
-25154.29	-6.89	-25115.81	-7.08	-25079.88	-9.82	-25040.19	-8.80	-25006.23	-13.51	-24966.58	-12.53	-24949.63	-14.91
-25154.56	-7.17	-25116.06	-7.34	-25078.83	-8.77	-25043.74	-12.34	-25006.62	-13.90	-24969.50	-15.45	-24951.51	-16.78
-25153.67	-6.27	-25116.12	-7.40	-25075.50	-5.44	-25041.17	-9.78	-25002.82	-10.10	-24969.77	-15.72	-24951.81	-17.09
-25153.42	-6.03	-25114.01	-5.28	-25078.71	-8.65	-25043.71	-12.31	-25003.47	-10.75	-24967.32	-13.27	-24950.78	-16.06
-25153.07	-5.68	-25117.08	-8.35	-25079.42	-9.36	-25038.92	-7.53	-25007.77	-15.05	-24969.99	-15.94	-24952.03	-17.30
-25153.40	-6.01	-25115.86	-7.13	-25078.71	-8.65	-25042.65	-11.25	-25006.42	-13.70	-24971.07	-17.01	-24951.27	-16.55
-25153.97	-6.57	-25114.80	-6.07	-25078.77	-8.71	-25041.98	-10.59	-25004.72	-12.00	-24965.35	-11.29	-24950.04	-15.32
-25152.92	-5.53	-25116.93	-8.21	-25077.39	-7.33	-25043.22	-11.83	-25005.04	-12.32	-24968.37	-14.32	-24952.48	-17.76
-25153.56	-6.17	-25116.58	-7.85	-25081.85	-11.79	-25044.19	-12.80	-25004.78	-12.05	-24969.34	-15.28	-24949.45	-14.73
-25154.39	-6.99	-25116.29	-7.57	-25080.12	-10.06	-25041.40	-10.01	-25006.62	-13.89	-24970.34	-16.28	-24948.50	-13.78
-25151.48	-4.08	-25116.40	-7.68	-25080.57	-10.51	-25043.81	-12.42	-25004.29	-11.57	-24970.38	-16.33	-24948.60	-13.88
-25153.66	-6.26	-25117.70	-8.97	-25078.96	-8.90	-25042.34	-10.95	-25004.67	-11.94	-24965.96	-11.90	-24950.75	-16.03
-25153.09	-5.70	-25116.02	-7.29	-25079.96	-9.90	-25042.32	-10.93	-25004.75	-12.02	-24969.58	-15.52	-24949.79	-15.07
-25154.89	-7.49	-25115.56	-6.83	-25079.67	-9.61	-25043.31	-11.92	-25005.30	-12.58	-24966.94	-12.88	-24952.15	-17.43
-25154.37	-6.97	-25116.02	-7.30	-25079.49	-9.44	-25041.02	-9.63	-25005.98	-13.26	-24969.05	-15.00	-24949.42	-14.70
-25152.09	-4.70	-25116.67	-7.95	-25079.00	-8.94	-25038.40	-7.01	-25005.35	-12.63	-24967.63	-13.58	-24951.26	-16.54
-25154.67	-7.28	-25115.99	-7.27	-25079.42	-9.36	-25040.17	-8.78	-25005.56	-12.84	-24966.99	-12.93	-24948.98	-14.25
-25154.69	-7.29	-25115.47	-6.75	-25077.99	-7.93	-25041.03	-9.63	-25003.79	-11.06	-24970.75	-16.69	-24951.44	-16.72
-25153.28	-5.89	-25116.70	-7.98	-25080.42	-10.36	-25041.17	-9.78	-25005.93	-13.21	-24968.93	-14.87	-24948.92	-14.19
-25153.98	-6.58	-25116.21	-7.48	-25077.48	-7.42	-25040.99	-9.60	-25006.95	-14.23	-24968.42	-14.37	-24950.03	-15.31
-25154.07	-6.68	-25116.76	-8.03	-25079.46	-9.40	-25041.97	-10.57	-25002.92	-10.19	-24970.86	-16.80	-24950.06	-15.34
-25152.96	-5.57	-25117.75	-9.02	-25078.40	-8.34	-25042.22	-10.83	-25006.14	-13.41	-24968.59	-14.53	-24950.98	-16.26
-25151.94	-4.54	-25117.81	-9.08	-25078.10	-8.04	-25043.30	-11.90	-25005.57	-12.84	-24970.75	-16.70	-24945.59	-10.87
-25153.40	-6.00	-25116.01	-7.28	-25078.84	-8.78	-25041.04	-9.64	-25005.47	-12.75	-24965.07	-11.01	-24952.28	-17.56
-25153.66	-6.27	-25116.69	-7.96	-25080.39	-10.33	-25041.40	-10.01	-25002.95	-10.22	-24970.44	-16.38	-24951.10	-16.38
-25153.85	-6.45	-25116.03	-7.30	-25078.35	-8.29	-25043.09	-11.70	-25006.28	-13.56	-24969.29	-15.23	-24952.16	-17.44
-25153.36	-5.97	-25116.12	-7.39	-25079.03	-8.97	-25040.96	-9.57	-25007.11	-14.39	-24968.58	-14.53	-24951.50	-16.78
-25154.08	-6.68	-25114.92	-6.19	-25079.07	-9.01	-25041.70	-10.31	-25005.54	-12.82	-24969.77	-15.72	-24948.47	-13.74
-25152.67	-5.27	-25115.86	-7.13	-25079.72	-9.66	-25041.84	-10.45	-25005.87	-13.15	-24967.85	-13.80	-24951.02	-16.30
-25153.53	-6.14	-25116.40	-7.68	-25078.38	-8.32	-25042.58	-11.19	-25005.79	-13.07	-24971.32	-17.26	-24953.12	-18.40
-25153.39	-5.99	-25116.00	-7.27	-25080.32	-10.26	-25043.24	-11.84	-25005.76	-13.04	-24968.98	-14.93	-24948.32	-13.59
-25154.25	-6.86	-25115.56	-6.83	-25079.06	-9.01	-25037.84	-6.45	-25005.76	-13.03	-24966.86	-12.81	-24950.27	-15.55
-25154.22	-6.82	-25116.74	-8.01	-25079.71	-9.65	-25042.35	-10.96	-25004.65	-11.93	-24968.24	-14.18	-24949.72	-15.00
-25153.69	-6.29	-25115.63	-6.90	-25080.03	-9.98	-25042.10	-10.71	-25005.73	-13.00	-24966.19	-12.14	-24951.33	-16.61
-25155.24	-7.84	-25116.05	-7.32	-25079.94	-9.88	-25041.47	-10.08	-25006.14	-13.41	-24968.23	-14.17	-24950.96	-16.24
-25154.15	-6.75	-25115.18	-6.46	-25080.26	-10.20	-25042.30	-10.91	-25007.95	-15.22	-24967.11	-13.05	-24950.07	-15.35
-25153.63	-6.24	-25114.44	-5.72	-25080.64	-10.58	-25039.65	-8.26	-25006.76	-14.04	-24969.13	-15.08	-24952.26	-17.54
-25154.03	-6.64	-25114.58	-5.85	-25079.87	-9.81	-25040.33	-8.94	-25003.16	-10.43	-24970.54	-16.48	-24952.09	-17.36
-25153.63	-6.23	-25114.43	-5.71	-25078.68	-8.62	-25044.24	-12.85	-25004.40	-11.68	-24967.81	-13.76	-24952.05	-17.33

-25154.53	-7.13	-25115.61	-6.88	-25080.34	-10.28	-25041.92	-10.53	-25002.87	-10.15	-24968.41	-14.35	-24947.86	-13.14
-25154.65	-7.26	-25118.21	-9.49	-25075.98	-5.93	-25041.95	-10.56	-25004.67	-11.94	-24969.28	-15.22	-24952.63	-17.91
-25152.79	-5.40	-25116.19	-7.46	-25078.30	-8.25	-25042.86	-11.47	-25006.42	-13.69	-24968.52	-14.46	-24951.75	-17.03
-25154.55	-7.15	-25114.78	-6.05	-25079.37	-9.31	-25042.64	-11.25	-25001.11	-8.39	-24966.82	-12.77	-24950.46	-15.74
-25152.20	-4.81	-25116.23	-7.51	-25078.87	-8.81	-25041.93	-10.54	-25003.91	-11.19	-24970.48	-16.42	-24952.93	-18.21
-25154.52	-7.13	-25115.69	-6.96	-25079.77	-9.71	-25041.16	-9.77	-25006.42	-13.70	-24968.02	-13.96	-24952.81	-18.09
-25152.07	-4.67	-25117.47	-8.74	-25079.87	-9.81	-25042.08	-10.69	-25006.69	-13.97	-24970.24	-16.18	-24948.69	-13.96
-25153.04	-5.65	-25116.98	-8.25	-25079.51	-9.45	-25041.64	-10.24	-25005.42	-12.70	-24968.34	-14.29	-24949.28	-14.56
-25153.20	-5.81	-25117.18	-8.46	-25079.19	-9.13	-25043.06	-11.67	-25007.68	-14.95	-24967.06	-13.00	-24950.65	-15.92
-25153.19	-5.79	-25116.04	-7.31	-25079.46	-9.40	-25042.76	-11.37	-25004.49	-11.77	-24969.66	-15.60	-24952.48	-17.76
-25153.88	-6.49	-25114.73	-6.01	-25080.61	-10.55	-25039.73	-8.34	-25005.43	-12.70	-24969.65	-15.60	-24950.56	-15.84
-25153.21	-5.81	-25116.71	-7.98	-25077.76	-7.71	-25042.06	-10.67	-25003.96	-11.24	-24970.42	-16.36	-24950.34	-15.62
-25153.94	-6.55	-25113.08	-4.36	-25078.77	-8.71	-25041.73	-10.34	-25006.12	-13.40	-24967.23	-13.18	-24952.64	-17.92
-25153.18	-5.79	-25116.39	-7.67	-25078.92	-8.86	-25040.84	-9.45	-25005.53	-12.80	-24968.07	-14.02	-24950.85	-16.13
-25153.30	-5.90	-25116.55	-7.82	-25079.97	-9.91	-25040.36	-8.97	-25005.77	-13.04	-24965.57	-11.52	-24951.34	-16.62
-25152.77	-5.38	-25116.97	-8.25	-25081.00	-10.94	-25044.18	-12.79	-25005.34	-12.62	-24969.68	-15.63	-24951.31	-16.59
-25155.08	-7.68	-25115.77	-7.04	-25075.83	-5.77	-25041.19	-9.80	-25007.75	-15.03	-24969.72	-15.67	-24949.45	-14.72
-25153.83	-6.43	-25117.06	-8.33	-25078.49	-8.43	-25041.87	-10.47	-25007.45	-14.73	-24969.19	-15.13	-24949.67	-14.95
-25154.29	-6.90	-25116.84	-8.11	-25078.43	-8.37	-25042.54	-11.15	-25007.10	-14.37	-24969.13	-15.07	-24950.83	-16.11
-25152.92	-5.52	-25115.10	-6.38	-25080.58	-10.52	-25041.11	-9.71	-25005.38	-12.66	-24968.84	-14.78	-24949.08	-14.36
-25153.55	-6.15	-25115.33	-6.60	-25079.96	-9.90	-25042.46	-11.07	-25005.30	-12.57	-24967.79	-13.73	-24952.01	-17.28
-25154.71	-7.32	-25117.04	-8.31	-25078.97	-8.92	-25039.95	-8.56	-25006.21	-13.48	-24969.98	-15.93	-24950.24	-15.52
-25152.53	-5.13	-25114.76	-6.04	-25081.05	-10.99	-25042.67	-11.27	-25006.32	-13.60	-24967.19	-13.13	-24951.62	-16.90
-25153.63	-6.24	-25117.22	-8.50	-25078.70	-8.64	-25040.97	-9.58	-25005.88	-13.16	-24968.39	-14.33	-24947.51	-12.78
-25153.94	-6.55	-25116.76	-8.03	-25079.29	-9.23	-25041.82	-10.43	-25004.84	-12.11	-24968.67	-14.62	-24948.87	-14.15
-25155.27	-7.88	-25115.55	-6.82	-25079.52	-9.46	-25041.85	-10.46	-25006.00	-13.28	-24968.56	-14.50	-24950.98	-16.26
-25154.51	-7.11	-25116.06	-7.33	-25078.78	-8.72	-25042.09	-10.70	-25005.54	-12.81	-24967.76	-13.71	-24949.99	-15.27
-25153.13	-5.74	-25115.76	-7.03	-25079.08	-9.02	-25042.08	-10.69	-25003.98	-11.25	-24968.83	-14.77	-24948.62	-13.90
-25155.16	-7.76	-25115.18	-6.46	-25077.85	-7.79	-25043.05	-11.66	-25003.59	-10.87	-24970.47	-16.42	-24952.20	-17.48
-25154.13	-6.74	-25116.15	-7.42	-25078.99	-8.93	-25043.47	-12.08	-25006.43	-13.70	-24969.85	-15.79	-24952.07	-17.35
-25153.06	-5.66	-25117.18	-8.46	-25080.37	-10.31	-25041.51	-10.12	-24994.22	-1.50	-24969.11	-15.06	-24947.47	-12.75
-25153.29	-5.90	-25116.97	-8.24	-25078.15	-8.09	-25038.75	-7.36	-25005.24	-12.52	-24969.63	-15.58	-24950.05	-15.33
-25152.67	-5.27	-25113.37	-4.65	-25079.75	-9.69	-25043.29	-11.90	-25006.84	-14.11	-24970.38	-16.32	-24948.00	-13.28
-25152.87	-5.47	-25116.34	-7.61	-25078.90	-8.84	-25042.43	-11.04	-25007.31	-14.58	-24971.80	-17.74	-24950.22	-15.49
-25152.57	-5.17	-25117.52	-8.79	-25079.20	-9.14	-25041.96	-10.57	-25004.47	-11.75	-24969.03	-14.97	-24951.43	-16.71
-25154.28	-6.89	-25116.01	-7.28	-25081.29	-11.23	-25040.07	-8.68	-25002.16	-9.44	-24969.66	-15.60	-24947.30	-12.58
-25153.87	-6.48	-25114.72	-6.00	-25077.47	-7.41	-25039.81	-8.42	-25003.65	-10.92	-24968.56	-14.50	-24950.53	-15.81
-25153.66	-6.26	-25115.03	-6.30	-25079.58	-9.52	-25043.57	-12.17	-25005.01	-12.29	-24968.06	-14.01	-24951.76	-17.04
-25152.89	-5.50	-25115.23	-6.50	-25079.55	-9.49	-25040.72	-9.33	-25006.15	-13.42	-24968.03	-13.97	-24950.38	-15.65
-25155.43	-8.04	-25115.12	-6.39	-25081.05	-10.99	-25040.79	-9.40	-25005.02	-12.30	-24969.93	-15.87	-24950.53	-15.81
-25153.91	-6.51	-25114.93	-6.21	-25078.46	-8.40	-25042.33	-10.94	-25003.42	-10.70	-24967.92	-13.87	-24948.75	-14.02

	-25154.27	-6.88	-25114.86	-6.14	-25079.39	-9.33	-25043.99	-12.59	-25003.78	-11.06	-24968.88	-14.83	-24951.11	-16.39
	-25153.39	-5.99	-25116.96	-8.24	-25078.53	-8.47	-25043.64	-12.25	-25001.79	-9.07	-24970.72	-16.66	-24952.52	-17.79
	-25153.31	-5.92	-25116.10	-7.38	-25079.23	-9.17	-25044.05	-12.66	-25005.12	-12.40	-24970.76	-16.70	-24949.05	-14.33
	-25153.26	-5.87	-25117.00	-8.27	-25080.61	-10.55	-25044.57	-13.18	-25005.33	-12.61	-24971.16	-17.10	-24952.37	-17.65
	-25154.44	-7.05	-25116.87	-8.15	-25080.55	-10.50	-25042.22	-10.83	-25004.17	-11.44	-24970.16	-16.11	-24951.22	-16.50
	-25153.86	-6.46	-25114.90	-6.17	-25079.51	-9.46	-25041.38	-9.99	-25005.35	-12.62	-24969.72	-15.66	-24950.00	-15.28
	-25153.09	-5.70	-25116.93	-8.20	-25080.99	-10.93	-25043.77	-12.38	-25004.14	-11.41	-24969.89	-15.83	-24951.25	-16.52
	-25154.05	-6.65	-25116.10	-7.37	-25079.65	-9.59	-25040.28	-8.89	-25005.65	-12.92	-24968.83	-14.77	-24948.38	-13.66
	-25153.02	-5.62	-25114.74	-6.01	-25081.34	-11.28	-25042.30	-10.91	-25004.35	-11.63	-24967.62	-13.56	-24952.44	-17.72
	-25154.18	-6.78	-25114.72	-5.99	-25078.36	-8.30	-25041.79	-10.40	-25005.36	-12.64	-24966.79	-12.74	-24950.16	-15.43
	-25154.65	-7.26	-25116.47	-7.74	-25078.86	-8.80	-25043.27	-11.88	-25005.66	-12.94	-24968.77	-14.72	-24950.87	-16.15
	-25153.29	-5.89	-25114.14	-5.42	-25078.70	-8.64	-25037.89	-6.50	-25005.04	-12.32			-24952.38	-17.65
	-25153.02	-5.62	-25116.37	-7.65			-25040.96	-9.57					-24950.13	-15.40
	-25153.26	-5.86	-25116.44	-7.71			-25042.94	-11.55						
	-25154.42	-7.02												
Average energies	-25153.72	-6.33	-25116.01	-7.28	-25079.11	-9.06	-25041.93	-10.54	-25005.23	-12.51	-24968.81	-14.75	-24950.49	-15.77

Th

	ground state energy	solid solution energy												
concentration in solid solution (%)	3		6		9		12		15		18		21	
number of atoms	2		4		6		8		10		12		13	
	-25157.98	-4.96	-25128.64	-8.65	-25096.14	-9.19	-25064.10	-10.19	-25034.33	-13.45	-25004.58	-16.74	-24986.81	-15.49
	-25159.15	-6.13	-25128.95	-8.96	-25095.17	-8.22	-25064.35	-10.44	-25033.22	-12.34	-25002.94	-15.10	-24985.88	-14.56
	-25157.93	-4.90	-25126.72	-6.73	-25096.57	-9.62	-25064.51	-10.60	-25033.79	-12.91	-25002.48	-14.64	-24988.42	-17.10
	-25159.87	-6.84	-25129.23	-9.24	-25093.73	-6.78	-25064.84	-10.93	-25032.46	-11.58	-25002.14	-14.30	-24991.11	-19.79
	-25160.39	-7.37	-25126.93	-6.94	-25097.59	-10.64	-25063.73	-9.82	-25032.69	-11.82	-25000.31	-12.47	-24986.89	-15.57
	-25159.83	-6.81	-25125.73	-5.74	-25096.50	-9.55	-25064.82	-10.91	-25034.54	-13.66	-24998.03	-10.19	-24984.96	-13.64
	-25158.70	-5.68	-25125.12	-5.13	-25094.80	-7.85	-25065.03	-11.11	-25034.96	-14.09	-25004.55	-16.72	-24988.16	-16.84
	-25158.54	-5.51	-25127.70	-7.72	-25097.18	-10.23	-25067.59	-13.68	-25035.60	-14.72	-25004.00	-16.16	-24986.68	-15.36
	-25160.12	-7.09	-25126.69	-6.70	-25097.11	-10.16	-25066.12	-12.20	-25031.64	-10.76	-25000.77	-12.93	-24986.30	-14.98
	-25158.42	-5.40	-25124.42	-4.43	-25097.18	-10.23	-25066.05	-12.13	-25033.29	-12.42	-25003.73	-15.89	-24988.06	-16.74
	-25159.31	-6.28	-25128.25	-8.26	-25096.44	-9.49	-25064.24	-10.33	-25032.98	-12.11	-25005.59	-17.76	-24987.28	-15.96
	-25157.98	-4.95	-25128.03	-8.04	-25094.39	-7.44	-25066.59	-12.68	-25035.20	-14.32	-25002.37	-14.53	-24987.40	-16.08
	-25159.69	-6.67	-25127.78	-7.80	-25095.97	-9.02	-25065.98	-12.06	-25032.47	-11.59	-24997.31	-9.48	-24988.80	-17.48
	-25159.61	-6.59	-25128.86	-8.88	-25095.21	-8.26	-25063.39	-9.48	-25033.84	-12.96	-25004.57	-16.73	-24988.65	-17.33
	-25158.19	-5.16	-25126.29	-6.30	-25097.55	-10.60	-25065.96	-12.04	-25034.84	-13.96	-25000.67	-12.83	-24983.73	-12.41
	-25157.82	-4.80	-25127.29	-7.30	-25096.87	-9.92	-25063.88	-9.97	-25033.92	-13.04	-24988.67	-0.83	-24990.88	-19.56
	-25157.95	-4.93	-25127.30	-7.32	-25096.57	-9.62	-25065.75	-11.83	-25033.85	-12.97	-25005.96	-18.12	-24991.34	-20.02

-25157.64	-4.62	-25127.59	-7.60	-25093.56	-6.60	-25063.64	-9.73	-25031.89	-11.02	-25004.95	-17.11	-24989.93	-18.61
-25159.41	-6.39	-25130.70	-10.71	-25095.57	-8.62	-25061.13	-7.22	-25032.21	-11.34	-25002.97	-15.14	-24990.30	-18.98
-25158.86	-5.84	-25127.22	-7.23	-25097.63	-10.68	-25064.76	-10.85	-25035.89	-15.01	-24999.95	-12.12	-24987.07	-15.75
-25158.37	-5.34	-25126.91	-6.92	-25098.20	-11.25	-25063.96	-10.05	-25032.64	-11.77	-25007.23	-19.39	-24987.27	-15.95
-25159.35	-6.32	-25129.55	-9.56	-25097.03	-10.08	-25065.80	-11.89	-25035.02	-14.14	-25002.66	-14.82	-24984.58	-13.26
-25159.32	-6.30	-25127.51	-7.52	-25096.47	-9.52	-25065.79	-11.88	-25034.65	-13.77	-25000.57	-12.73	-24990.98	-19.66
-25158.83	-5.81	-25129.24	-9.25	-25096.65	-9.70	-25062.44	-8.53	-25033.36	-12.48	-25003.41	-15.57	-24990.09	-18.77
-25160.74	-7.71	-25128.51	-8.52	-25097.27	-10.32	-25062.82	-8.91	-25032.10	-11.22	-25001.17	-13.33	-24986.67	-15.35
-25159.62	-6.59	-25127.76	-7.77	-25094.92	-7.96	-25062.55	-8.63	-25035.61	-14.74	-25003.47	-15.63	-24987.86	-16.54
-25160.31	-7.29	-25127.65	-7.66	-25096.22	-9.27	-25062.44	-8.53	-25036.62	-15.75	-25002.99	-15.15	-24989.11	-17.79
-25159.31	-6.29	-25127.56	-7.57	-25094.47	-7.51	-25067.09	-13.18	-25034.70	-13.83	-25002.86	-15.02	-24987.45	-16.13
-25159.67	-6.65	-25127.74	-7.75	-25096.79	-9.84	-25063.67	-9.76	-25031.04	-10.16	-25004.88	-17.04	-24985.07	-13.75
-25158.43	-5.41	-25128.39	-8.41	-25098.03	-11.08	-25066.67	-12.76	-25033.65	-12.77	-25006.02	-18.18	-24979.04	-7.72
-25160.70	-7.67	-25127.11	-7.12	-25096.64	-9.69	-25064.63	-10.71	-25035.42	-14.54	-25002.90	-15.06	-24987.07	-15.75
-25159.07	-6.04	-25127.12	-7.13	-25096.92	-9.97	-25061.36	-7.44	-25035.46	-14.58	-25004.07	-16.23	-24991.62	-20.30
-25158.62	-5.59	-25128.09	-8.10	-25095.43	-8.48	-25067.21	-13.29	-25035.85	-14.97	-25004.67	-16.83	-24991.16	-19.84
-25159.46	-6.44	-25128.50	-8.51	-25094.48	-7.53	-25066.19	-12.28	-25032.43	-11.55	-25002.76	-14.92	-24987.93	-16.61
-25158.74	-5.71	-25127.41	-7.43	-25095.88	-8.93	-25065.98	-12.06	-25035.07	-14.20	-24999.51	-11.67	-24990.06	-18.74
-25159.10	-6.07	-25126.01	-6.02	-25095.39	-8.44	-25065.81	-11.89	-25035.67	-14.80	-25002.19	-14.35	-24988.51	-17.19
-25158.98	-5.95	-25128.51	-8.52	-25098.68	-11.73	-25066.42	-12.51	-25031.09	-10.22	-25006.13	-18.29	-24987.20	-15.88
-25160.57	-7.54	-25125.89	-5.90	-25095.16	-8.21	-25065.21	-11.30	-25026.28	-5.40	-25001.74	-13.90	-24990.70	-19.38
-25157.14	-4.11	-25128.16	-8.18	-25094.04	-7.09	-25056.72	-2.81	-25034.85	-13.97	-25005.41	-17.57	-24988.60	-17.28
-25159.99	-6.97	-25126.36	-6.37	-25096.05	-9.10	-25061.76	-7.85	-25032.44	-11.57	-25004.65	-16.81	-24988.34	-17.02
-25159.46	-6.44	-25127.30	-7.31	-25095.71	-8.76	-25063.02	-9.11	-25030.10	-9.22	-25000.77	-12.93	-24989.44	-18.12
-25158.74	-5.71	-25126.60	-6.61	-25098.60	-11.65	-25067.10	-13.19	-25031.95	-11.08	-25004.12	-16.29	-24987.31	-15.99
-25159.63	-6.60	-25127.97	-7.98	-25095.13	-8.18	-25064.11	-10.20	-25034.73	-13.85	-25002.22	-14.38	-24988.70	-17.38
-25159.66	-6.64	-25125.78	-5.79	-25094.91	-7.96	-25064.75	-10.83	-25029.91	-9.03	-25002.98	-15.15	-24980.95	-9.63
-25159.06	-6.04	-25128.52	-8.53	-25096.08	-9.13	-25062.28	-8.37	-25035.52	-14.64	-25005.58	-17.75	-24991.27	-19.95
-25159.34	-6.31	-25128.42	-8.43	-25096.84	-9.89	-25065.59	-11.68	-25032.22	-11.34	-25002.61	-14.77	-24985.69	-14.37
-25159.30	-6.28	-25126.98	-6.99	-25095.95	-9.00	-25064.50	-10.59	-25035.07	-14.20	-25003.19	-15.36	-24989.88	-18.56
-25159.98	-6.95	-25125.76	-5.77	-25096.98	-10.03	-25063.83	-9.92	-25031.22	-10.35	-25000.67	-12.83	-24991.18	-19.86
-25159.42	-6.39	-25125.48	-5.49	-25095.24	-8.29	-25063.31	-9.40	-25034.86	-13.99	-25002.60	-14.76	-24991.11	-19.79
-25159.02	-6.00	-25126.69	-6.70	-25097.40	-10.45	-25063.28	-9.37	-25033.69	-12.82	-25005.35	-17.51	-24987.88	-16.56
-25160.06	-7.03	-25127.10	-7.11	-25095.94	-8.99	-25064.46	-10.55	-25035.32	-14.44	-25004.70	-16.87	-24983.02	-11.70
-25159.78	-6.76	-25127.20	-7.21	-25095.32	-8.37	-25065.81	-11.90	-25032.88	-12.00	-25001.75	-13.91	-24991.14	-19.82
-25159.48	-6.46	-25126.05	-6.07	-25097.82	-10.87	-25061.45	-7.54	-25036.82	-15.94	-24999.66	-11.82	-24988.34	-17.02
-25160.73	-7.70	-25129.17	-9.18	-25096.26	-9.31	-25058.42	-4.51	-25030.89	-10.01	-25003.06	-15.22	-24989.56	-18.24
-25159.09	-6.06	-25127.61	-7.62	-25096.05	-9.10	-25067.19	-13.27	-25035.37	-14.49	-25005.13	-17.29	-24986.63	-15.31
-25160.07	-7.05	-25127.96	-7.97	-25094.12	-7.17	-25063.79	-9.87	-25031.39	-10.51	-25002.20	-14.36	-24990.19	-18.87
-25160.72	-7.70	-25124.71	-4.73	-25095.63	-8.68	-25065.43	-11.52	-25032.09	-11.22	-25001.01	-13.17	-24984.04	-12.72
-25160.53	-7.51	-25126.78	-6.79	-25096.56	-9.61	-25064.09	-10.17	-25034.51	-13.64	-25006.86	-19.02	-24985.74	-14.42

-25160.37	-7.35	-25129.59	-9.60	-25095.90	-8.95	-25062.18	-8.27	-25034.01	-13.14	-25004.36	-16.52	-24990.26	-18.94
-25159.59	-6.56	-25128.07	-8.08	-25095.17	-8.22	-25066.23	-12.32	-25037.68	-16.80	-25003.30	-15.46	-24988.01	-16.69
-25158.76	-5.74	-25128.71	-8.72	-25093.80	-6.85	-25059.85	-5.94	-25033.39	-12.52	-25002.22	-14.38	-24988.88	-17.56
-25159.20	-6.18	-25127.08	-7.09	-25096.79	-9.84	-25065.53	-11.62	-25032.66	-11.79	-25000.20	-12.36	-24985.79	-14.47
-25159.84	-6.82	-25126.23	-6.24	-25094.20	-7.25	-25064.20	-10.29	-25027.70	-6.83	-25003.93	-16.09	-24986.17	-14.85
-25159.45	-6.43	-25127.42	-7.44	-25094.87	-7.92	-25064.52	-10.60	-25033.74	-12.87	-24999.80	-11.96	-24989.58	-18.26
-25159.65	-6.63	-25129.00	-9.01	-25096.94	-9.99	-25059.26	-5.34	-25032.98	-12.10	-25005.03	-17.19	-24985.55	-14.23
-25159.63	-6.60	-25128.53	-8.54	-25096.46	-9.51	-25064.08	-10.17	-25029.15	-8.27	-25003.52	-15.68	-24989.47	-18.15
-25159.75	-6.72	-25126.09	-6.10	-25096.59	-9.64	-25062.56	-8.65	-25034.54	-13.66	-24999.92	-12.08	-24988.05	-16.73
-25160.35	-7.33	-25128.25	-8.26	-25096.90	-9.95	-25066.12	-12.21	-25035.24	-14.36	-25005.31	-17.47	-24989.57	-18.25
-25159.26	-6.24	-25128.50	-8.52	-25096.39	-9.44	-25064.68	-10.77	-25031.52	-10.64	-25000.06	-12.22	-24988.39	-17.07
-25158.82	-5.80	-25128.50	-8.51	-25097.58	-10.63	-25067.44	-13.52	-25034.50	-13.62	-25002.42	-14.59	-24988.76	-17.44
-25159.19	-6.17	-25126.83	-6.84	-25094.39	-7.44	-25065.05	-11.14	-25035.02	-14.14	-25005.24	-17.41	-24988.92	-17.60
-25159.63	-6.60	-25125.24	-5.26	-25096.37	-9.42	-25064.10	-10.19	-25032.60	-11.72	-24998.62	-10.78	-24987.55	-16.23
-25159.95	-6.93	-25127.46	-7.47	-25093.67	-6.72	-25063.10	-9.19	-25027.38	-6.51	-25001.78	-13.94	-24990.45	-19.13
-25159.17	-6.15	-25127.40	-7.42	-25095.85	-8.90	-25062.80	-8.89	-25032.32	-11.44	-24997.99	-10.15	-24986.25	-14.93
-25158.86	-5.83	-25127.46	-7.47	-25094.11	-7.16	-25064.58	-10.66	-25034.39	-13.51	-25000.82	-12.98	-24987.40	-16.08
-25157.57	-4.54	-25127.70	-7.71	-25092.66	-5.71	-25062.73	-8.82	-25036.02	-15.14	-25001.39	-13.55	-24983.42	-12.10
-25158.51	-5.49	-25128.73	-8.74	-25097.13	-10.18	-25061.98	-8.06	-25027.00	-6.12	-25003.15	-15.31	-24990.82	-19.50
-25159.26	-6.23	-25128.48	-8.49	-25096.45	-9.50	-25063.28	-9.37	-25033.59	-12.72	-25000.59	-12.75	-24988.76	-17.44
-25158.39	-5.37	-25126.80	-6.81	-25094.85	-7.90	-25063.59	-9.68	-25031.64	-10.77	-25003.42	-15.58	-24987.83	-16.51
-25159.79	-6.77	-25126.72	-6.73	-25098.32	-11.37	-25064.23	-10.32	-25033.96	-13.08	-25002.34	-14.50	-24988.16	-16.84
-25159.38	-6.36	-25127.60	-7.61	-25096.13	-9.18	-25065.58	-11.67	-25034.12	-13.24	-25005.83	-17.99	-24986.01	-14.69
-25158.40	-5.37	-25126.33	-6.34	-25098.46	-11.51	-25064.09	-10.18	-25033.88	-13.00	-25003.87	-16.03	-24987.43	-16.11
-25159.45	-6.42	-25128.25	-8.26	-25097.04	-10.09	-25062.76	-8.85	-25035.02	-14.15	-25002.04	-14.20	-24988.94	-17.62
-25160.25	-7.22	-25125.61	-5.62	-25097.50	-10.55	-25065.54	-11.63	-25036.42	-15.54	-25007.08	-19.24	-24989.58	-18.26
-25160.10	-7.08	-25125.93	-5.94	-25095.76	-8.81	-25065.62	-11.70	-25037.28	-16.40	-24999.98	-12.15	-24990.04	-18.72
-25158.53	-5.51	-25129.33	-9.34	-25096.85	-9.90	-25063.59	-9.68	-25029.19	-8.32	-25002.13	-14.29	-24986.39	-15.07
-25158.78	-5.76	-25126.30	-6.31	-25094.80	-7.85	-25064.65	-10.74	-25030.23	-9.36	-25000.01	-12.17	-24983.28	-11.96
-25158.82	-5.80	-25123.93	-3.94	-25096.41	-9.46	-25062.80	-8.89	-25030.00	-9.12	-25002.34	-14.50	-24987.65	-16.33
-25158.44	-5.41	-25129.22	-9.23	-25094.54	-7.59	-25068.13	-14.21	-25031.85	-10.98	-25005.28	-17.45	-24988.86	-17.54
-25158.89	-5.86	-25128.36	-8.38	-25096.13	-9.18	-25062.90	-8.99	-25034.13	-13.26	-25005.10	-17.26	-24989.31	-17.99
-25160.15	-7.13	-25128.66	-8.67	-25094.58	-7.63	-25066.34	-12.42	-25033.24	-12.36	-25000.40	-12.57	-24989.25	-17.93
-25159.49	-6.46	-25129.66	-9.67	-25096.71	-9.76	-25062.84	-8.92	-25033.95	-13.08	-25003.91	-16.07	-24991.10	-19.78
-25157.76	-4.73	-25127.38	-7.39	-25096.20	-9.25	-25065.68	-11.77	-25028.40	-7.52	-25004.55	-16.71	-24988.49	-17.17
-25159.74	-6.71	-25127.08	-7.09	-25095.15	-8.20	-25064.00	-10.09	-25036.89	-16.02	-25005.15	-17.31	-24987.88	-16.56
-25159.15	-6.12	-25127.68	-7.69	-25094.65	-7.69	-25067.02	-13.11	-25032.77	-11.90	-25002.30	-14.46	-24990.66	-19.34
-25157.58	-4.55	-25127.87	-7.88	-25097.18	-10.23	-25065.76	-11.85	-25033.72	-12.85	-25002.57	-14.73	-24986.94	-15.62
-25159.55	-6.52	-25126.64	-6.66	-25094.74	-7.79	-25066.12	-12.21	-25030.31	-9.44	-25004.72	-16.88	-24985.53	-14.21
-25158.60	-5.58	-25128.11	-8.12	-25097.43	-10.48	-25064.05	-10.13	-25035.33	-14.46	-25004.74	-16.90	-24988.21	-16.89
-25160.17	-7.14	-25126.43	-6.45	-25094.62	-7.67	-25062.75	-8.84	-25032.78	-11.91	-25005.29	-17.46	-24988.68	-17.36

	-25157.82	-4.80	-25125.87	-5.88	-25098.12	-11.17	-25067.51	-13.60	-25036.10	-15.23	-25000.90	-13.06	-24988.26	-16.94
	-25158.86	-5.83	-25124.30	-4.31	-25097.60	-10.65	-25064.32	-10.41	-25030.78	-9.91	-25001.20	-13.36	-24990.32	-19.00
	-25160.56	-7.53	-25128.42	-8.43	-25097.35	-10.40	-25063.64	-9.73	-25030.59	-9.71	-25007.84	-20.00	-24986.39	-15.07
	-25160.76	-7.74	-25128.20	-8.21	-25096.45	-9.50	-25062.78	-8.87	-25032.45	-11.58	-25002.47	-14.64	-24986.07	-14.75
	-25157.88	-4.85	-25126.16	-6.17	-25094.17	-7.22	-25064.30	-10.39	-25034.72	-13.85	-25000.71	-12.88	-24984.63	-13.31
	-25160.20	-7.17	-25128.52	-8.53	-25096.72	-9.77	-25063.57	-9.65	-25034.09	-13.21	-24998.24	-10.40	-24987.42	-16.10
	-25158.95	-5.92	-25127.72	-7.73	-25094.74	-7.79	-25064.22	-10.31	-25031.29	-10.42	-25003.04	-15.20	-24985.95	-14.63
	-25158.62	-5.60	-25129.61	-9.62	-25093.53	-6.58	-25061.42	-7.51	-25035.12	-14.24	-25005.05	-17.21	-24990.66	-19.34
	-25160.61	-7.58	-25127.38	-7.39	-25097.19	-10.24	-25062.47	-8.56	-25033.33	-12.45	-25005.39	-17.55	-24989.28	-17.96
	-25158.66	-5.64	-25127.21	-7.23	-25096.60	-9.65	-25062.02	-8.11	-25032.86	-11.99	-25003.19	-15.35	-24981.62	-10.30
	-25160.55	-7.53	-25127.08	-7.10	-25094.79	-7.84	-25064.93	-11.02	-25035.82	-14.95	-25003.50	-15.66	-24989.00	-17.68
	-25159.28	-6.26	-25126.14	-6.15	-25098.22	-11.27	-25063.22	-9.31	-25033.19	-12.31	-25006.01	-18.17	-24988.62	-17.30
	-25159.86	-6.84	-25128.16	-8.18	-25096.17	-9.22	-25062.84	-8.93	-25035.24	-14.36	-25002.27	-14.43	-24989.15	-17.83
	-25159.19	-6.16	-25128.00	-8.01	-25098.58	-11.63	-25066.52	-12.60	-25033.70	-12.83	-25002.77	-14.93		
	-25158.97	-5.95	-25126.95	-6.96			-25065.63	-11.72	-25032.18	-11.30	-25004.87	-17.03		
	-25159.43	-6.40	-25127.30	-7.31			-25065.72	-11.81	-25034.42	-13.54				
	-25157.74	-4.72	-25126.06	-6.07			-25066.64	-12.73	-25034.71	-13.83				
	-25159.77	-6.75	-25130.52	-10.53			-25066.86	-12.95	-25034.10	-13.22				
	-25158.82	-5.80	-25127.08	-7.09					-25032.23	-11.36				
	-25159.62	-6.60												
	-25158.85	-5.83												
Average energies	-25159.26	-6.23	-25127.45	-7.46	-25096.08	-9.13	-25064.31	-10.40	-25033.34	-12.46	-25002.81	-14.97	-24987.94	-16.62

U

	ground state energy	solid solution energy												
concentration in solid solution (%)	3		6		9		12		15		18		21	
number of atoms	2		4		6		8		10		12		13	
	-25164.48	-6.44	-25136.71	-6.68	-25111.98	-9.97	-25085.48	-11.49	-25059.97	-14.00	-25031.03	-13.08	-25020.96	-17.02
	-25163.17	-5.13	-25137.86	-7.84	-25107.76	-5.76	-25085.32	-11.33	-25058.41	-12.44	-25034.81	-16.86	-25021.00	-17.06
	-25163.64	-5.59	-25137.17	-7.15	-25109.54	-7.54	-25086.46	-12.48	-25061.75	-15.78	-25033.41	-15.46	-25021.98	-18.03
	-25163.11	-5.07	-25138.69	-8.66	-25111.89	-9.89	-25081.91	-7.93	-25062.86	-16.89	-25033.24	-15.29	-25021.53	-17.59
	-25164.21	-6.17	-25137.06	-7.04	-25112.29	-10.28	-25086.01	-12.02	-25060.21	-14.24	-25035.59	-17.64	-25022.02	-18.08
	-25164.71	-6.67	-25137.07	-7.04	-25108.96	-6.96	-25086.05	-12.06	-25059.07	-13.10	-25036.73	-18.78	-25021.25	-17.31
	-25164.03	-5.98	-25137.09	-7.06	-25111.02	-9.02	-25082.93	-8.94	-25057.10	-11.13	-25033.57	-15.62	-25020.76	-16.81
	-25162.03	-3.99	-25137.79	-7.76	-25108.71	-6.70	-25085.78	-11.79	-25060.10	-14.13	-25032.40	-14.45	-25020.68	-16.73
	-25163.24	-5.20	-25137.83	-7.80	-25111.87	-9.86	-25087.73	-13.74	-25058.18	-12.21	-25032.52	-14.57	-25021.19	-17.24
	-25164.34	-6.30	-25136.78	-6.76	-25112.48	-10.48	-25085.08	-11.09	-25058.60	-12.63	-25031.78	-13.83	-25018.98	-15.04
	-25163.49	-5.44	-25137.00	-6.97	-25111.54	-9.53	-25083.36	-9.37	-25060.48	-14.51	-25034.38	-16.43	-25016.92	-12.98

-25162.74	-4.69	-25137.70	-7.67	-25111.97	-9.97	-25082.46	-8.47	-25059.12	-13.15	-25035.95	-18.00	-25023.96	-20.01
-25164.03	-5.99	-25137.80	-7.78	-25111.23	-9.23	-25083.85	-9.87	-25059.35	-13.38	-25030.16	-12.21	-25021.56	-17.62
-25163.97	-5.93	-25135.97	-5.95	-25110.89	-8.88	-25082.97	-8.99	-25058.85	-12.88	-25033.10	-15.15	-25022.49	-18.54
-25164.03	-5.98	-25137.32	-7.30	-25111.54	-9.53	-25083.57	-9.58	-25058.32	-12.35	-25034.58	-16.62	-25021.02	-17.07
-25156.89	1.15	-25137.70	-7.68	-25112.27	-10.26	-25085.82	-11.83	-25063.07	-17.10	-25032.88	-14.93	-25020.85	-16.91
-25162.97	-4.93	-25136.80	-6.78	-25107.98	-5.97	-25084.26	-10.27	-25058.59	-12.62	-25031.61	-13.65	-25023.47	-19.53
-25163.26	-5.21	-25138.24	-8.22	-25112.83	-10.82	-25084.26	-10.27	-25055.85	-9.88	-25031.86	-13.91	-25020.61	-16.67
-25162.45	-4.40	-25136.90	-6.88	-25110.56	-8.55	-25085.74	-11.75	-25054.62	-8.65	-25028.61	-10.66	-25020.95	-17.00
-25163.92	-5.87	-25134.86	-4.84	-25112.95	-10.94	-25085.06	-11.07	-25059.47	-13.50	-25032.51	-14.56	-25019.68	-15.74
-25164.50	-6.46	-25137.95	-7.93	-25112.08	-10.07	-25081.61	-7.62	-25058.68	-12.71	-25030.49	-12.54	-25015.75	-11.81
-25163.08	-5.03	-25136.01	-5.99	-25109.09	-7.09	-25086.07	-12.09	-25060.59	-14.62	-25037.23	-19.28	-25020.82	-16.88
-25163.71	-5.67	-25134.10	-4.07	-25110.46	-8.45	-25083.90	-9.91	-25062.87	-16.90	-25030.63	-12.68	-25026.00	-22.05
-25163.83	-5.79	-25137.57	-7.54	-25112.30	-10.29	-25085.88	-11.89	-25054.05	-8.08	-25032.35	-14.39	-25022.17	-18.23
-25162.62	-4.57	-25138.23	-8.21	-25112.71	-10.70	-25084.42	-10.44	-25056.79	-10.82	-25031.17	-13.22	-25021.05	-17.11
-25165.00	-6.95	-25137.94	-7.91	-25112.36	-10.35	-25084.07	-10.08	-25061.43	-15.46	-25035.38	-17.43	-25019.74	-15.80
-25162.50	-4.46	-25137.13	-7.10	-25110.47	-8.46	-25085.65	-11.66	-25055.54	-9.57	-25032.66	-14.71	-25017.42	-13.48
-25163.51	-5.46	-25136.14	-6.12	-25109.44	-7.44	-25082.29	-8.31	-25056.71	-10.74	-25034.49	-16.54	-25016.26	-12.32
-25163.39	-5.34	-25137.72	-7.70	-25108.59	-6.58	-25084.54	-10.55	-25061.30	-15.33	-25036.84	-18.89	-25023.72	-19.78
-25158.15	-0.11	-25136.96	-6.94	-25110.50	-8.50	-25086.90	-12.91	-25061.46	-15.49	-25034.97	-17.02	-25023.85	-19.91
-25164.00	-5.96	-25139.05	-9.03	-25112.16	-10.15	-25086.58	-12.59	-25059.41	-13.44	-25033.94	-15.98	-25022.54	-18.60
-25162.72	-4.68	-25138.42	-8.40	-25110.44	-8.43	-25085.73	-11.75	-25059.16	-13.19	-25037.04	-19.09	-25019.55	-15.60
-25163.84	-5.80	-25133.97	-3.94	-25111.28	-9.28	-25083.93	-9.94	-25059.32	-13.35	-25033.47	-15.52	-25024.06	-20.11
-25163.36	-5.31	-25137.91	-7.89	-25110.23	-8.23	-25082.12	-8.13	-25060.46	-14.49	-25030.87	-12.92	-25023.39	-19.44
-25163.43	-5.39	-25136.39	-6.37	-25111.57	-9.56	-25085.07	-11.08	-25062.11	-16.14	-25035.11	-17.16	-25021.92	-17.98
-25163.24	-5.19	-25136.68	-6.65	-25112.57	-10.56	-25087.35	-13.36	-25059.17	-13.20	-25034.59	-16.64	-25024.16	-20.22
-25162.75	-4.71	-25135.30	-5.28	-25112.97	-10.96	-25085.77	-11.78	-25061.70	-15.73	-25033.65	-15.70	-25022.84	-18.90
-25162.68	-4.64	-25130.78	-0.75	-25111.50	-9.50	-25083.29	-9.30	-25061.51	-15.54	-25035.34	-17.38	-25021.44	-17.50
-25163.96	-5.91	-25137.44	-7.42	-25111.46	-9.45	-25086.13	-12.14	-25060.19	-14.22	-25033.96	-16.01	-25022.96	-19.02
-25163.18	-5.13	-25137.07	-7.04	-25108.41	-6.40	-25084.29	-10.30	-25057.62	-11.65	-25034.59	-16.64	-25020.39	-16.44
-25164.36	-6.32	-25136.94	-6.92	-25112.27	-10.26	-25083.16	-9.17	-25060.20	-14.23	-25031.23	-13.28	-25021.81	-17.87
-25164.23	-6.19	-25136.88	-6.86	-25111.06	-9.06	-25083.71	-9.73	-25061.55	-15.58	-25033.38	-15.43	-25023.37	-19.42
-25163.50	-5.45	-25135.71	-5.69	-25111.90	-9.89	-25083.12	-9.13	-25059.86	-13.89	-25032.76	-14.81	-25022.68	-18.74
-25163.08	-5.04	-25137.52	-7.49	-25108.05	-6.04	-25081.65	-7.67	-25058.82	-12.85	-25031.17	-13.22	-25022.98	-19.04
-25164.44	-6.40	-25137.76	-7.74	-25110.59	-8.59	-25086.83	-12.84	-25059.85	-13.88	-25033.50	-15.55	-25021.21	-17.27
-25156.92	1.12	-25130.01	0.01	-25111.49	-9.49	-25086.52	-12.53	-25057.61	-11.64	-25033.42	-15.46	-25015.19	-11.25
-25163.63	-5.59	-25137.49	-7.47	-25112.42	-10.41	-25084.40	-10.41	-25060.05	-14.08	-25029.36	-11.41	-25020.79	-16.85
-25163.30	-5.25	-25136.59	-6.56	-25110.65	-8.64	-25085.30	-11.31	-25058.63	-12.66	-25033.02	-15.07	-25020.08	-16.14
-25163.70	-5.66	-25136.67	-6.65	-25112.97	-10.96	-25086.22	-12.23	-25058.02	-12.05	-25031.71	-13.76	-25020.83	-16.89
-25163.96	-5.92	-25138.26	-8.23	-25113.71	-11.71	-25082.67	-8.68	-25061.83	-15.86	-25030.81	-12.86		
-25163.02	-4.98	-25138.69	-8.67	-25110.51	-8.50	-25083.87	-9.88	-25059.37	-13.40	-25031.45	-13.50	-25023.15	-19.21
-25164.25	-6.20	-25136.96	-6.94	-25112.90	-10.89	-25072.82	1.17	-25059.39	-13.42	-25035.09	-17.14	-25020.37	-16.42

-25163.05	-5.01	-25136.09	-6.07	-25112.20	-10.19	-25084.35	-10.36	-25060.44	-14.47	-25031.62	-13.67	-25021.66	-17.72
-25164.02	-5.98	-25139.21	-9.18	-25111.84	-9.83	-25083.58	-9.59	-25062.21	-16.24	-25032.22	-14.27	-25021.89	-17.95
-25162.99	-4.95	-25135.07	-5.04	-25110.19	-8.18	-25085.78	-11.80	-25063.39	-17.42	-25032.69	-14.74	-25018.09	-14.14
-25162.90	-4.86	-25139.01	-8.99	-25110.29	-8.29	-25085.10	-11.11	-25063.25	-17.28	-25028.79	-10.84	-25018.82	-14.88
-25163.73	-5.69	-25135.13	-5.10	-25112.66	-10.65	-25088.05	-14.06	-25056.28	-10.31	-25027.74	-9.79	-25024.06	-20.12
-25164.69	-6.65	-25138.87	-8.84	-25112.63	-10.63	-25082.46	-8.47	-25061.21	-15.24	-25029.69	-11.74	-25018.19	-14.25
-25162.82	-4.78	-25138.15	-8.12	-25112.51	-10.50	-25086.88	-12.89	-25058.20	-12.23	-25034.49	-16.54	-25023.22	-19.27
-25161.43	-3.38	-25138.08	-8.06	-25112.86	-10.86	-25084.32	-10.34	-25059.38	-13.41	-25037.26	-19.31	-25021.58	-17.64
-25163.60	-5.55	-25138.26	-8.24	-25111.44	-9.44	-25080.32	-6.34	-25057.76	-11.79	-25031.19	-13.24	-25019.11	-15.16
-25163.44	-5.40	-25134.89	-4.87	-25111.27	-9.27	-25084.73	-10.74	-25059.88	-13.91	-25035.38	-17.43	-25017.49	-13.54
-25163.25	-5.21	-25137.77	-7.75	-25111.21	-9.20	-25085.95	-11.96	-25060.02	-14.05	-25031.75	-13.80	-25018.95	-15.01
-25163.34	-5.30	-25138.73	-8.71	-25110.31	-8.31	-25083.31	-9.32	-25053.39	-7.42	-25037.16	-19.21	-25025.19	-21.25
-25162.90	-4.86	-25138.16	-8.14	-25112.14	-10.13	-25084.76	-10.77	-25055.28	-9.31	-25033.47	-15.52	-25023.29	-19.35
-25161.93	-3.89	-25138.06	-8.03	-25112.50	-10.49	-25083.88	-9.89	-25060.27	-14.30	-25035.66	-17.71	-25021.36	-17.41
-25162.72	-4.68	-25136.64	-6.62	-25112.58	-10.58	-25087.23	-13.24	-25058.40	-12.43	-25029.37	-11.42	-25023.18	-19.24
-25161.71	-3.67	-25135.68	-5.66	-25109.16	-7.15	-25081.99	-8.01	-25061.62	-15.65	-25034.82	-16.87	-25020.25	-16.31
-25165.63	-7.59	-25137.12	-7.10	-25110.44	-8.43	-25084.21	-10.23	-25055.41	-9.45	-25032.70	-14.75	-25023.67	-19.73
-25164.45	-6.41	-25135.13	-5.10	-25110.46	-8.46	-25084.50	-10.51	-25059.78	-13.81	-25033.25	-15.30	-25021.43	-17.49
-25164.39	-6.35	-25136.31	-6.28	-25111.35	-9.35	-25086.36	-12.37	-25055.56	-9.59	-25034.02	-16.07	-25022.11	-18.16
-25163.64	-5.59	-25135.33	-5.30	-25112.32	-10.31	-25083.52	-9.53	-25061.66	-15.69	-25034.19	-16.24	-25023.12	-19.18
-25164.28	-6.24	-25136.87	-6.85	-25110.45	-8.44	-25086.55	-12.57	-25061.83	-15.87	-25036.17	-18.22	-25021.61	-17.67
-25164.34	-6.30	-25137.82	-7.79	-25112.67	-10.66	-25086.27	-12.28	-25059.12	-13.15	-25035.62	-17.67	-25017.96	-14.02
-25163.74	-5.70	-25135.50	-5.48	-25110.18	-8.18	-25084.86	-10.87	-25057.44	-11.48	-25033.98	-16.03	-25018.87	-14.93
-25163.80	-5.75	-25138.22	-8.20	-25111.30	-9.29	-25085.34	-11.35	-25062.46	-16.49	-25033.36	-15.41	-25020.58	-16.63
-25164.03	-5.98	-25138.28	-8.25	-25110.70	-8.69	-25081.96	-7.97	-25059.09	-13.12	-25030.56	-12.61	-25020.28	-16.33
-25163.12	-5.08	-25137.21	-7.18	-25108.77	-6.76	-25084.27	-10.29	-25057.40	-11.43	-25029.44	-11.49	-25024.43	-20.48
-25164.61	-6.56	-25138.08	-8.06	-25110.58	-8.57	-25083.83	-9.84	-25061.10	-15.13	-25035.97	-18.02	-25019.31	-15.37
-25163.74	-5.70	-25136.35	-6.33	-25111.40	-9.39	-25086.04	-12.05	-25058.73	-12.76	-25032.29	-14.34	-25018.17	-14.23
-25163.07	-5.02	-25137.20	-7.17	-25108.73	-6.72	-25085.14	-11.15	-25058.87	-12.90	-25034.31	-16.36	-25021.96	-18.02
-25163.64	-5.60	-25137.31	-7.29	-25111.61	-9.60	-25085.76	-11.77	-25060.93	-14.96	-25035.51	-17.56	-25014.99	-11.05
-25163.96	-5.92	-25137.75	-7.73	-25112.26	-10.26	-25083.86	-9.87	-25059.00	-13.03	-25032.60	-14.65	-25020.12	-16.18
-25163.69	-5.65	-25138.34	-8.32			-25083.79	-9.80	-25057.42	-11.45	-25030.50	-12.55	-25025.37	-21.43
-25163.82	-5.78	-25137.87	-7.84			-25083.84	-9.85	-25061.60	-15.63	-25031.30	-13.35	-25019.33	-15.39
-25162.32	-4.28	-25137.37	-7.34			-25086.34	-12.36			-25035.53	-17.58	-25020.83	-16.89
-25163.90	-5.85	-25138.17	-8.15			-25083.52	-9.53					-25022.14	-18.20
-25163.66	-5.61	-25137.05	-7.03			-25082.46	-8.47					-25021.42	-17.48
-25163.97	-5.93	-25136.52	-6.50			-25086.11	-12.12						
-25164.20	-6.15	-25137.02	-6.99			-25082.97	-8.98						
-25162.96	-4.91	-25136.82	-6.79			-25084.15	-10.16						
-25164.17	-6.12	-25138.71	-8.68			-25083.29	-9.30						
		-25137.29	-7.26			-25082.41	-8.42						

			-25137.58	-7.55			-25085.10	-11.11						
			-25135.55	-5.52			-25078.76	-4.77						
			-25133.00	-2.98			-25085.24	-11.25						
			-25134.84	-4.81			-25084.22	-10.23						
			-25137.67	-7.64			-25083.01	-9.02						
							-25085.89	-11.90						
Average energies	-25163.35	-5.31	-25136.97	-6.94	-25111.19	-9.18	-25084.41	-10.42	-25059.38	-13.41	-25033.15	-15.20	-25021.13	-17.19

$Ca_{1-x}M_xZrTi_{2-2x}Al_{2x}O_7$ M=(Ce, Pu, Th, U)

Ti(3)Ti(3)

N.B. All stated energies are in eV

Ce

	ground state energy	solid solution energy														
concentration in solid solution (%)	3		6		9		12		15		18		21			
number of atoms	2		4		6		8		10		12		14			
	-25153.25	0.13	-25116.58	4.12	-25079.93	8.09	-25043.01	12.32	-25004.67	17.98	-24968.06	21.91	-24931.86	25.43		
	-25155.56	-2.18	-25117.40	3.30	-25080.11	7.91	-25042.80	12.53	-25005.34	17.31	-24968.94	21.03	-24933.61	23.68		
	-25153.71	-0.33	-25116.32	4.38	-25078.98	9.04	-25043.69	11.64	-25006.70	15.95	-24966.79	23.18	-24928.88	28.41		
	-25151.89	1.49	-25115.88	4.81	-25080.42	7.59	-25040.67	14.67	-25005.59	17.06	-24969.55	20.42	-24934.63	22.66		
	-25152.73	0.65	-25116.26	4.44	-25081.42	6.59	-25043.01	12.32	-25004.31	18.34	-24967.82	22.15	-24933.11	24.17		
	-25153.50	-0.12	-25116.18	4.51	-25079.35	8.66	-25043.11	12.22	-25007.26	15.39	-24970.54	19.43	-24933.92	23.36		
	-25153.67	-0.29	-25116.27	4.42	-25079.47	8.54	-25044.51	10.83	-25006.70	15.95	-24967.16	22.81	-24933.12	24.17		
	-25152.31	1.07	-25116.70	4.00	-25078.40	9.61	-25044.28	11.06	-25000.78	21.87	-24967.50	22.47	-24933.76	23.53		
	-25154.29	-0.91	-25118.05	2.64	-25079.69	8.33	-25040.68	14.66	-25003.86	18.79	-24968.84	21.13	-24934.06	23.22		
	-25153.83	-0.45	-25114.52	6.18	-25073.11	14.90	-25041.70	13.63	-25006.11	16.54	-24969.01	20.96	-24932.46	24.83		
	-25155.07	-1.69	-25117.83	2.87	-25079.47	8.55	-25043.85	11.48	-25003.09	19.56	-24971.08	18.89	-24930.96	26.33		
	-25153.92	-0.54	-25117.23	3.46	-25078.49	9.53	-25044.45	10.88	-25006.69	15.97	-24962.35	27.62	-24933.99	23.30		
	-25154.18	-0.80	-25117.67	3.03	-25080.94	7.07	-25043.63	11.70	-25006.31	16.34	-24966.65	23.31	-24934.83	22.45		
	-25153.42	-0.04	-25115.96	4.74	-25076.66	11.35	-25044.86	10.47	-25004.30	18.35	-24968.24	21.73	-24932.17	25.12		
	-25151.68	1.70	-25115.63	5.07	-25078.58	9.44	-25040.70	14.63	-25005.60	17.06	-24970.90	19.07	-24933.53	23.76		
	-25152.55	0.83	-25115.93	4.76	-25079.83	8.19	-25041.87	13.47	-25005.49	17.16	-24969.64	20.33	-24932.51	24.77		
	-25153.26	0.12	-25116.95	3.75	-25079.82	8.20	-25043.78	11.55	-25005.85	16.80	-24969.80	20.17	-24932.48	24.81		
	-25154.11	-0.73	-25112.12	8.58	-25080.23	7.78	-25045.48	9.86	-25007.65	15.00	-24967.80	22.17	-24933.68	23.60		
	-25153.50	-0.12	-25116.92	3.78	-25076.08	11.93	-25039.48	15.85	-25006.28	16.38	-24970.83	19.14	-24929.06	28.23		
	-25154.27	-0.89	-25117.58	3.12	-25078.43	9.59	-25037.77	17.56	-24998.86	23.79	-24969.90	20.07	-24934.84	22.45		
	-25153.09	0.29	-25116.29	4.40	-25080.46	7.55	-25041.56	13.77	-25005.03	17.62	-24968.78	21.19	-24931.36	25.93		
	-25154.38	-1.01	-25117.21	3.48	-25081.44	6.58	-25043.74	11.59	-25003.44	19.21	-24967.56	22.41	-24925.29	31.99		
	-25151.52	1.86	-25116.84	3.86	-25079.29	8.73	-25044.27	11.07	-25006.99	15.66	-24968.29	21.68	-24930.81	26.47		
	-25154.24	-0.86	-25115.14	5.56	-25076.82	11.19	-25043.04	12.29	-25004.49	18.17	-24967.45	22.52	-24931.72	25.56		
	-25151.70	1.68	-25117.56	3.14	-25077.87	10.14	-25043.32	12.01	-25007.22	15.43	-24966.58	23.39	-24934.44	22.85		
	-25153.27	0.11	-25117.03	3.67	-25080.04	7.97	-25042.95	12.38	-25004.36	18.29	-24969.75	20.22	-24934.38	22.91		
	-25153.39	-0.01	-25115.19	5.51	-25078.33	9.69	-25041.39	13.94	-25007.14	15.51	-24969.57	20.40	-24934.11	23.17		
	-25153.66	-0.28	-25116.12	4.58	-25079.59	8.42	-25041.67	13.66	-25006.84	15.81	-24963.07	26.90	-24933.58	23.71		
	-25154.45	-1.07	-25117.73	2.97	-25078.29	9.73	-25043.03	12.31	-25008.46	14.19	-24967.89	22.08	-24935.15	22.13		

-25152.24	1.14	-25115.95	4.75	-25081.14	6.87	-25042.51	12.83	-25006.12	16.53	-24968.58	21.39	-24935.29	22.00
-25154.38	-1.00	-25117.34	3.35	-25080.28	7.74	-25044.57	10.76	-25005.96	16.70	-24968.21	21.76	-24928.01	29.28
-25154.38	-1.00	-25116.37	4.33	-25079.93	8.08	-25047.11	8.23	-25003.97	18.68	-24968.44	21.53	-24933.53	23.75
-25153.07	0.31	-25115.96	4.73	-25078.59	9.43	-25041.19	14.14	-25006.66	15.99	-24970.95	19.02	-24930.92	26.37
-25153.95	-0.57	-25114.42	6.27	-25080.06	7.96	-25041.00	14.33	-25005.87	16.78	-24970.97	18.99	-24934.59	22.70
-25154.49	-1.11	-25116.42	4.28	-25078.32	9.69	-25042.77	12.57	-25006.02	16.63	-24970.66	19.31	-24932.70	24.58
-25152.57	0.81	-25117.34	3.36	-25080.74	7.28	-25038.71	16.62	-25003.58	19.07	-24968.11	21.86	-24932.90	24.39
-25153.73	-0.35	-25116.49	4.21	-25079.70	8.32	-25041.47	13.86	-25004.75	17.90	-24967.66	22.31	-24930.24	27.04
-25153.74	-0.36	-25116.57	4.13	-25079.15	8.86	-25044.71	10.63	-25004.27	18.39	-24965.27	24.70	-24932.00	25.29
-25153.99	-0.61	-25116.21	4.48	-25080.26	7.75	-25039.27	16.07	-25006.47	16.18	-24969.87	20.09	-24927.08	30.20
-25152.36	1.02	-25116.14	4.56	-25080.05	7.97	-25041.03	14.30	-25007.26	15.40	-24971.98	17.99	-24930.35	26.94
-25153.77	-0.39	-25116.08	4.61	-25079.67	8.34	-25042.89	12.44	-25000.20	22.45	-24968.84	21.13	-24933.85	23.43
-25155.05	-1.67	-25117.51	3.19	-25081.32	6.69	-25043.11	12.22	-25001.60	21.05	-24965.73	24.24	-24931.35	25.93
-25153.87	-0.49	-25117.38	3.32	-25079.65	8.36	-25043.74	11.59	-25008.37	14.28	-24970.18	19.79	-24932.72	24.56
-25153.93	-0.55	-25117.04	3.66	-25081.41	6.60	-25042.31	13.02	-25004.68	17.97	-24968.61	21.36	-24932.72	24.57
-25154.13	-0.75	-25117.09	3.61	-25079.87	8.14	-25042.99	12.34	-25003.98	18.67	-24967.91	22.06	-24933.38	23.91
-25154.08	-0.70	-25116.12	4.58	-25078.63	9.39	-25042.00	13.33	-25003.27	19.38	-24966.03	23.94	-24932.40	24.89
-25153.88	-0.50	-25116.76	3.94	-25076.49	11.53	-25040.87	14.46	-25005.98	16.67	-24971.01	18.96	-24932.79	24.50
-25153.36	0.01	-25116.43	4.27	-25079.59	8.42	-25043.85	11.48	-25006.32	16.33	-24970.20	19.77	-24932.12	25.17
-25154.38	-1.00	-25116.60	4.10	-25080.83	7.18	-25044.46	10.87	-25006.05	16.60	-24971.58	18.39	-24933.05	24.23
-25153.64	-0.27	-25117.85	2.85	-25080.90	7.12	-25044.93	10.40	-25006.08	16.58	-24971.13	18.84	-24932.78	24.50
-25153.95	-0.57	-25115.84	4.85	-25078.42	9.60	-25044.69	10.64	-25005.66	16.99	-24965.86	24.11	-24933.34	23.95
-25153.55	-0.17	-25114.61	6.09	-25078.82	9.19	-25041.77	13.57	-25008.31	14.34	-24969.07	20.90	-24936.72	20.57
-25152.76	0.62	-25117.57	3.12	-25080.91	7.10	-25041.65	13.68	-25005.69	16.97	-24969.46	20.51	-24933.52	23.77
-25153.82	-0.44	-25114.77	5.93	-25079.59	8.43	-25043.14	12.20	-25005.96	16.69	-24968.29	21.68	-24932.91	24.37
-25153.10	0.28	-25117.17	3.53	-25080.78	7.23	-25045.04	10.29	-25005.77	16.88	-24966.90	23.07	-24928.62	28.67
-25152.61	0.77	-25115.45	5.25	-25078.19	9.82	-25043.68	11.65	-25008.18	14.47	-24967.18	22.79	-24931.37	25.92
-25148.25	5.13	-25115.51	5.19	-25080.21	7.81	-25039.64	15.69	-25004.29	18.36	-24968.59	21.38	-24931.36	25.93
-25154.92	-1.54	-25113.82	6.88	-25080.17	7.84	-25044.43	10.90	-25005.46	17.19	-24962.23	27.74	-24933.76	23.53
-25153.28	0.10	-25117.19	3.51	-25079.86	8.15	-25043.39	11.94	-25006.52	16.13	-24967.47	22.50	-24929.85	27.44
-25153.81	-0.43	-25116.58	4.12	-25079.46	8.56	-25036.82	18.51	-25009.20	13.45	-24971.01	18.96	-24931.33	25.96
-25152.58	0.80	-25117.12	3.58	-25079.55	8.47	-25043.77	11.56	-25006.78	15.87	-24966.85	23.12	-24933.03	24.25
-25154.16	-0.78	-25117.72	2.98	-25082.33	5.69	-25042.76	12.58	-25003.55	19.10	-24971.19	18.78	-24931.04	26.25
-25153.85	-0.47	-25116.70	4.00	-25079.79	8.23	-25040.86	14.47	-25005.65	17.01	-24968.60	21.37	-24932.12	25.16
-25153.40	-0.02	-25116.66	4.04	-25080.77	7.24	-25043.83	11.50	-25006.50	16.15	-24965.01	24.96	-24933.58	23.71
-25153.84	-0.46	-25116.95	3.75	-25074.08	13.93	-25043.45	11.89	-25004.69	17.96	-24965.54	24.43	-24932.46	24.82
-25154.16	-0.78	-25116.63	4.06	-25080.27	7.75	-25042.40	12.94	-25006.01	16.64	-24969.10	20.87	-24931.40	25.89
-25152.91	0.47	-25117.18	3.52	-25077.29	10.73	-25042.15	13.18	-25005.10	17.55	-24969.59	20.38	-24931.61	25.68
-25154.12	-0.74	-25115.18	5.52	-25076.70	11.32	-25042.94	12.40	-25007.03	15.63	-24972.06	17.91	-24932.81	24.47
-25153.54	-0.16	-25117.59	3.11	-25081.31	6.70	-25044.08	11.25	-25007.08	15.57	-24968.49	21.47	-24936.45	20.84
-25153.49	-0.11	-25117.71	2.99	-25079.76	8.26	-25042.41	12.93	-25003.81	18.84	-24971.84	18.13	-24929.95	27.34

-25153.58	-0.20	-25117.36	3.34	-25079.15	8.87	-25042.60	12.73	-25007.07	15.58	-24971.81	18.16	-24932.73	24.56
-25153.71	-0.33	-25117.13	3.57	-25080.18	7.83	-25042.82	12.51	-25006.77	15.88	-24969.37	20.60	-24930.87	26.42
-25154.03	-0.65	-25076.70	44.00	-25079.20	8.82	-25042.64	12.69	-25001.17	21.49	-24968.90	21.07	-24935.55	21.74
-25154.44	-1.06	-25116.17	4.53	-25078.89	9.13	-25040.15	15.18	-25005.45	17.21	-24967.77	22.20	-24934.59	22.70
-25154.60	-1.22	-25115.18	5.52	-25080.20	7.81	-25041.51	13.83	-25006.37	16.28	-24970.67	19.30	-24932.37	24.92
-25153.03	0.35	-25115.29	5.40	-25079.49	8.52	-25041.01	14.32	-25006.06	16.59	-24971.64	18.33	-24932.19	25.09
-25154.94	-1.56	-25116.85	3.84	-25080.05	7.97	-25041.37	13.96	-25003.78	18.87	-24967.89	22.08	-24929.46	27.82
-25153.47	-0.09	-25116.74	3.95	-25079.57	8.45	-25043.56	11.78	-25002.07	20.58	-24968.12	21.85	-24933.90	23.38
-25153.56	-0.19	-25116.13	4.57	-25079.45	8.57	-25040.65	14.68	-25004.21	18.44	-24973.04	16.93	-24935.48	21.81
-25152.51	0.87	-25117.73	2.97	-25079.55	8.46	-25043.20	12.13	-25005.29	17.36	-24966.48	23.49	-24929.21	28.08
-25154.03	-0.65	-25116.14	4.56	-25075.38	12.63	-25042.58	12.76	-25005.12	17.53	-24970.37	19.60	-24930.31	26.97
-25153.86	-0.48	-25115.38	5.31	-25077.76	10.26	-25044.13	11.21	-25006.41	16.24	-24969.25	20.72	-24934.99	22.30
-25154.54	-1.16	-25115.37	5.33	-25080.40	7.61	-25043.52	11.82	-25004.25	18.41	-24971.52	18.45	-24933.50	23.79
-25154.00	-0.62	-25115.84	4.86	-25080.87	7.14	-25043.60	11.73	-25007.74	14.91	-24964.38	25.59	-24930.10	27.19
-25153.30	0.08	-25079.00	41.70	-25081.55	6.46	-25046.21	9.13	-25005.69	16.96	-24967.82	22.15	-24932.47	24.82
-25153.11	0.27	-25115.99	4.70	-25078.62	9.40	-25044.12	11.22	-25006.48	16.17	-24970.93	19.04	-24933.85	23.43
-25154.50	-1.12	-25115.83	4.87	-25078.07	9.95	-25045.56	9.78	-25002.69	19.96	-24971.36	18.61	-24933.47	23.81
-25153.94	-0.56	-25117.81	2.89	-25078.60	9.42	-25039.97	15.36	-25006.07	16.58	-24966.43	23.54	-24935.98	21.30
-25154.19	-0.81	-25116.29	4.41	-25080.88	7.14	-25040.63	14.70	-25001.21	21.44	-24962.70	27.27	-24932.42	24.87
-25154.64	-1.26	-25114.86	5.84	-25081.13	6.89	-25041.91	13.43	-25008.17	14.48	-24968.84	21.13	-24933.18	24.10
-25154.22	-0.84	-25117.14	3.56	-25080.46	7.56	-25041.29	14.04	-25006.08	16.57	-24967.21	22.76	-24932.85	24.43
-25153.27	0.11	-25115.00	5.69	-25079.38	8.63	-25043.62	11.71	-25003.67	18.99	-24970.03	19.93	-24929.25	28.04
-25151.97	1.41	-25117.00	3.70	-25079.56	8.46	-25042.86	12.47	-25007.10	15.55	-24967.94	22.03	-24934.84	22.45
-25153.26	0.12	-25116.84	3.86	-25079.09	8.93	-25041.31	14.03	-25005.40	17.25	-24969.48	20.49	-24934.07	23.22
-25152.96	0.42	-25116.78	3.92	-25081.09	6.92	-25042.73	12.61	-25004.90	17.76	-24970.97	19.00	-24932.66	24.63
-25153.90	-0.52	-25115.36	5.34	-25077.88	10.13	-25044.77	10.56	-25005.50	17.15	-24967.75	22.22	-24935.41	21.88
-25154.16	-0.78	-25114.64	6.06	-25079.60	8.42	-25044.12	11.22	-25004.22	18.43	-24968.67	21.30	-24928.27	29.02
-25153.15	0.23	-25117.11	3.59	-25080.69	7.33	-25042.89	12.44	-25006.98	15.67	-24968.86	21.11	-24931.61	25.68
-25151.86	1.52	-25114.76	5.94	-25078.73	9.29	-25040.22	15.11	-25007.97	14.68	-24968.71	21.26	-24931.74	25.55
-25152.71	0.67	-25117.41	3.28	-25080.38	7.63	-25040.03	15.30	-25005.70	16.95	-24971.47	18.50	-24933.95	23.34
-25153.93	-0.55	-25118.53	2.17	-25081.18	6.83	-25042.70	12.63	-25004.80	17.85	-24969.73	20.24	-24933.24	24.04
-25153.29	0.09	-25115.90	4.80	-25077.97	10.04	-25043.14	12.20	-25002.87	19.78	-24969.96	20.01	-24931.11	26.18
-25154.14	-0.76	-25115.92	4.77	-25079.08	8.94	-25044.06	11.27	-25007.75	14.90	-24966.56	23.41	-24925.53	31.76
-25153.15	0.23	-25117.73	2.96	-25079.34	8.68	-25040.76	14.57	-25003.66	18.99	-24968.45	21.51	-24934.85	22.43
-25153.54	-0.16	-25116.08	4.62	-25079.42	8.60	-25040.99	14.34	-25004.84	17.81	-24970.29	19.68	-24933.01	24.28
-25153.90	-0.52	-25115.81	4.89	-25078.41	9.61	-25044.70	10.64	-25006.50	16.16	-24968.10	21.87	-24932.04	25.25
-25154.79	-1.41	-25116.42	4.28	-25080.86	7.16	-25044.56	10.77	-25006.64	16.01	-24969.15	20.82	-24931.77	25.52
-25153.53	-0.15	-25114.77	5.93	-25079.07	8.94	-25044.46	10.87	-25006.18	16.47	-24970.21	19.76	-24932.78	24.51
-25153.49	-0.11	-25115.57	5.13	-25078.12	9.89	-25043.31	12.03	-25003.44	19.21	-24969.48	20.49	-24930.22	27.07
-25153.54	-0.16	-25117.50	3.19	-25076.03	11.99	-25042.41	12.92	-25004.48	18.17	-24968.85	21.12	-24932.52	24.76
-25152.37	1.01	-25117.54	3.16	-25079.17	8.85	-25043.74	11.60	-25005.61	17.05	-24969.18	20.79	-24933.60	23.69

	-25154.03	-0.65	-25118.10	2.60			-25041.44	13.89	-25007.58	15.07	-24967.41	22.56	-24931.80	25.49
	-25154.31	-0.93	-25115.69	5.01			-25041.80	13.53	-25005.84	16.82	-24965.91	24.05	-24932.46	24.83
	-25153.32	0.06	-25116.25	4.45			-25043.95	11.39	-25007.31	15.34	-24963.51	26.46	-24931.08	26.21
	-25153.84	-0.46	-25116.89	3.81			-25040.87	14.46	-25005.09	17.56	-24968.37	21.60	-24932.45	24.83
	-25154.45	-1.07	-25117.82	2.88			-25042.68	12.65	-25005.06	17.59	-24964.32	25.65	-24931.97	25.32
	-25153.65	-0.27	-25117.39	3.31							-24970.04	19.93	-24932.10	25.19
	-25154.52	-1.14	-25115.96	4.73							-24969.97	20.00		
	-25153.76	-0.38	-25115.34	5.35							-24969.79	20.18		
	-25151.93	1.45	-25116.20	4.50										
Average energies	-25153.57	-0.19	-25115.79	4.91	-25079.42	8.59	-25042.66	12.66	-25005.48	17.20	-24968.61	21.35	-24932.43	24.86

**Pu**	ground state energy	solid solution energy												
concentration in solid solution (%)	3		6		9		12		15		18		21	
number of atoms	2		4		6		8		10		12		14	
	-25148.99	-1.60	-25104.72	4.00	-25060.15	9.91	-25017.47	13.92	-24973.51	19.21	-24936.60	17.46	-24893.98	21.41
	-25148.06	-0.67	-25105.58	3.15	-25060.85	9.21	-25019.27	12.12	-24977.91	14.81	-24935.19	18.86	-24893.23	22.16
	-25148.80	-1.41	-25104.21	4.52	-25062.12	7.94	-25019.34	12.05	-24975.69	17.03	-24935.17	18.89	-24891.62	23.77
	-25147.46	-0.07	-25104.15	4.58	-25063.03	7.03	-25016.51	14.88	-24975.41	17.32	-24934.80	19.25	-24894.17	21.22
	-25148.39	-1.00	-25103.35	5.38	-25058.42	11.64	-25020.42	10.97	-24975.31	17.42	-24932.51	21.55	-24892.73	22.66
	-25147.76	-0.37	-25105.05	3.68	-25061.40	8.66	-25017.98	13.42	-24978.40	14.33	-24934.75	19.30	-24889.07	26.32
	-25148.40	-1.01	-25106.12	2.61	-25063.00	7.06	-25016.51	14.88	-24976.41	16.31	-24933.80	20.25	-24892.00	23.39
	-25148.20	-0.80	-25105.18	3.54	-25061.61	8.44	-25017.84	13.55	-24976.67	16.05	-24936.07	17.99	-24894.75	20.64
	-25146.73	0.66	-25105.18	3.55	-25061.18	8.88	-25016.17	15.22	-24976.66	16.06	-24934.24	19.81	-24892.86	22.53
	-25148.41	-1.01	-25104.62	4.10	-25060.81	9.25	-25021.36	10.03	-24977.34	15.39	-24934.03	20.03	-24893.60	21.78
	-25148.61	-1.21	-25103.81	4.91	-25063.78	6.28	-25018.88	12.51	-24978.89	13.83	-24936.16	17.89	-24893.28	22.11
	-25148.02	-0.63	-25103.29	5.44	-25063.52	6.54	-25016.55	14.84	-24977.35	15.38	-24936.76	17.29	-24890.77	24.62
	-25147.86	-0.47	-25105.72	3.00	-25062.97	7.09	-25017.04	14.35	-24978.13	14.59	-24936.45	17.61	-24891.77	23.62
	-25149.24	-1.85	-25103.24	5.48	-25060.97	9.09	-25020.57	10.82	-24978.82	13.90	-24935.16	18.89	-24889.92	25.47
	-25146.24	1.16	-25104.33	4.39	-25063.40	6.65	-25018.31	13.08	-24976.97	15.76	-24938.58	15.48	-24892.15	23.24
	-25147.76	-0.37	-25103.69	5.03	-25063.71	6.35	-25020.24	11.15	-24977.05	15.68	-24936.56	17.49	-24890.65	24.74
	-25148.05	-0.65	-25104.95	3.78	-25059.44	10.62	-25020.76	10.63	-24976.27	16.46	-24935.08	18.98	-24893.44	21.95
	-25147.55	-0.15	-25105.04	3.69	-25061.37	8.69	-25019.41	11.98	-24976.34	16.38	-24931.17	22.89	-24891.45	23.94
	-25148.24	-0.84	-25103.88	4.85	-25061.68	8.38	-25020.14	11.25	-24978.96	13.76	-24935.50	18.56	-24891.57	23.82
	-25147.78	-0.38	-25104.82	3.91	-25061.75	8.31	-25020.60	10.79	-24977.26	15.46	-24935.05	19.00	-24892.57	22.82
	-25149.13	-1.74	-25104.37	4.36	-25063.58	6.48	-25018.40	12.99	-24979.55	13.17	-24935.40	19.00	-24893.99	21.40
	-25148.37	-0.97	-25103.25	5.47	-25064.69	5.37	-25018.18	13.22	-24977.15	15.58	-24935.38	18.66	-24891.50	23.89
	-25146.93	0.46	-25103.99	4.74	-25062.10	7.96	-25019.90	11.49	-24974.51	18.21	-24936.23	18.68	-24890.70	24.69

-25147.46	-0.06	-25105.30	3.43	-25059.41	10.65	-25020.61	10.78	-24976.91	15.82	-24935.61	17.83	-24891.74	23.65
-25148.04	-0.65	-25103.95	4.77	-25061.71	8.35	-25020.38	11.01	-24977.15	15.57	-24934.82	18.44	-24894.55	20.84
-25148.83	-1.44	-25104.45	4.27	-25064.03	6.03	-25019.49	11.90	-24972.76	19.96	-24935.42	19.23	-24890.11	25.27
-25146.90	0.50	-25104.22	4.51	-25064.36	5.70	-25018.41	12.98	-24974.12	18.60	-24935.58	18.64	-24892.11	23.28
-25149.00	-1.60	-25106.62	2.10	-25061.79	8.27	-25018.96	12.43	-24976.40	16.33	-24931.86	18.48	-24893.22	22.17
-25147.47	-0.08	-25104.28	4.44	-25063.03	7.03	-25017.95	13.44	-24979.01	13.71	-24935.03	22.19	-24893.12	22.27
-25146.95	0.44	-25106.16	2.57	-25057.59	12.47	-25020.56	10.84	-24974.71	18.02	-24932.64	19.03	-24893.15	22.24
-25147.94	-0.54	-25104.79	3.94	-25063.26	6.80	-25021.12	10.27	-24974.92	17.81	-24938.24	21.42	-24890.10	25.29
-25147.78	-0.39	-25104.23	4.50	-25063.25	6.81	-25021.62	9.77	-24976.34	16.39	-24935.76	15.81	-24895.36	20.03
-25147.50	-0.10	-25104.66	4.06	-25062.18	7.88	-25016.30	15.10	-24975.97	16.75	-24934.83	18.30	-24894.09	21.30
-25148.02	-0.63	-25105.62	3.10	-25064.12	5.94	-25019.89	11.50	-24979.75	12.97	-24935.58	19.23	-24891.84	23.55
-25148.20	-0.80	-25105.07	3.65	-25064.14	5.92	-25021.96	9.43	-24976.82	15.91	-24932.39	18.47	-24891.99	23.40
-25147.54	-0.14	-25105.99	2.74	-25061.94	8.12	-25017.30	14.09	-24976.48	16.24	-24935.23	21.67	-24893.29	22.10
-25147.41	-0.01	-25104.92	3.81	-25061.23	8.83	-25019.15	12.24	-24977.06	15.67	-24937.78	18.83	-24891.72	23.66
-25146.73	0.66	-25105.04	3.69	-25061.66	8.40	-25017.15	14.24	-24977.40	15.32	-24936.91	16.27	-24891.32	24.07
-25147.61	-0.22	-25105.12	3.61	-25061.85	8.21	-25015.60	15.79	-24978.28	14.44	-24935.59	17.15	-24894.09	21.30
-25147.76	-0.37	-25105.23	3.49	-25064.01	6.05	-25017.95	13.44	-24977.23	15.50	-24935.30	18.47	-24887.91	27.48
-25148.65	-1.25	-25103.50	5.23	-25061.51	8.55	-25019.85	11.54	-24977.10	15.62	-24935.41	18.76	-24892.35	23.04
-25147.02	0.38	-25105.90	2.83	-25063.98	6.08	-25018.65	12.74	-24976.88	15.85	-24930.70	18.65	-24892.62	22.77
-25146.80	0.60	-25105.17	3.55	-25061.80	8.26	-25018.53	12.86	-24977.76	14.96	-24934.93	23.35	-24894.58	20.81
-25148.73	-1.34	-25104.63	4.10	-25062.94	7.12	-25018.22	13.17	-24976.22	16.51	-24936.86	19.12	-24892.25	23.14
-25148.67	-1.28	-25104.43	4.30	-25064.11	5.95	-25021.23	10.16	-24976.39	16.33	-24934.56	17.19	-24892.71	22.68
-25148.32	-0.93	-25105.20	3.53	-25062.19	7.87	-25018.69	12.70	-24976.35	16.38	-24936.82	19.49	-24892.75	22.64
-25147.93	-0.53	-25103.56	5.16	-25063.19	6.87	-25019.29	12.10	-24976.07	16.65	-24935.10	17.24	-24894.23	21.16
-25147.59	-0.20	-25105.70	3.03	-25061.41	8.65	-25018.33	13.06	-24977.15	15.57	-24935.16	18.96	-24890.90	24.48
-25149.00	-1.60	-25105.66	3.07	-25059.72	10.34	-25020.16	11.23	-24974.90	17.83	-24932.42	18.90	-24892.45	22.94
-25148.12	-0.72	-25106.08	2.65	-25061.71	8.35	-25019.31	12.08	-24979.28	13.44	-24935.62	21.64	-24894.84	20.55
-25148.44	-1.04	-25105.66	3.07	-25064.01	6.05	-25018.95	12.44	-24977.05	15.67	-24935.58	18.43	-24892.84	22.54
-25148.04	-0.65	-25105.23	3.49	-25058.36	11.70	-25019.96	11.43	-24976.05	16.67	-24934.92	18.47	-24892.34	23.04
-25148.99	-1.59	-25105.07	3.66	-25061.77	8.29	-25020.01	11.39	-24974.31	18.41	-24937.03	19.14	-24891.76	23.63
-25147.66	-0.27	-25104.91	3.82	-25061.30	8.76	-25019.48	11.91	-24977.13	15.59	-24933.53	17.03	-24892.74	22.65
-25149.08	-1.68	-25106.26	2.47	-25063.21	6.85	-25020.00	11.39	-24977.94	14.78	-24931.78	20.53	-24891.78	23.61
-25147.87	-0.48	-25105.97	2.76	-25061.27	8.79	-25020.36	11.03	-24978.55	14.17	-24935.18	22.27	-24892.55	22.83
-25147.51	-0.12	-25105.60	3.13	-25063.28	6.78	-25020.37	11.02	-24976.51	16.22	-24934.94	18.88	-24892.17	23.22
-25148.03	-0.63	-25103.34	5.39	-25063.89	6.16	-25016.32	15.07	-24977.47	15.26	-24935.20	19.12	-24894.57	20.82
-25147.36	0.03	-25106.88	1.84	-25062.96	7.10	-25021.17	10.22	-24973.83	18.89	-24935.41	18.85	-24892.03	23.36
-25148.84	-1.45	-25105.29	3.43	-25061.51	8.55	-25015.75	15.64	-24976.48	16.25	-24936.22	18.64	-24892.88	22.51
-25146.23	1.17	-25102.62	6.10	-25060.89	9.17	-25020.42	10.97	-24979.90	12.83	-24935.08	17.83	-24892.58	22.81
-25147.92	-0.53	-25104.93	3.79	-25062.49	7.56	-25020.85	10.54	-24975.51	17.22	-24936.50	18.97	-24893.96	21.43
-25147.55	-0.16	-25103.34	5.39	-25062.45	7.61	-25017.05	14.35	-24977.93	14.79	-24935.50	17.55	-24892.86	22.53
-25149.24	-1.84	-25103.60	5.13	-25061.04	9.02	-25019.14	12.25	-24977.77	14.95	-24937.82	18.56	-24893.08	22.30

-25148.02	-0.63	-25105.64	3.08	-25062.16	7.90	-25021.23	10.16	-24975.62	17.10	-24937.99	16.24	-24891.36	24.03
-25145.51	1.88	-25104.85	3.08	-25063.51	6.55	-25014.59	16.80	-24978.52	14.21	-24935.41	16.07	-24891.26	24.13
-25147.82	-0.42	-25104.38	3.87	-25058.74	11.32	-25019.60	11.79	-24976.36	16.36	-24936.55	18.64	-24895.50	19.89
-25148.39	-0.99	-25105.89	4.34	-25061.41	8.65	-25020.23	11.16	-24975.40	17.33	-24927.83	17.51	-24894.48	20.91
-25148.58	-1.19	-25106.82	2.83	-25062.55	7.51	-25021.04	10.36	-24977.96	14.77	-24935.88	26.23	-24892.12	23.27
-25147.58	-0.18	-25102.08	1.91	-25062.63	7.43	-25018.99	12.41	-24978.45	14.27	-24932.20	18.18	-24890.59	24.80
-25147.86	-0.47	-25105.48	6.65	-25063.29	6.76	-25020.13	11.27	-24975.31	17.41	-24933.49	21.85	-24892.79	22.60
-25150.44	-3.05	-25105.96	3.24	-25060.51	9.55	-25021.15	10.24	-24977.13	15.59	-24935.18	21.85	-24892.54	22.85
-25147.04	0.35	-25102.79	2.77	-25059.82	10.24	-25018.72	12.67	-24975.25	17.47	-24937.91	20.57	-24892.36	23.02
-25147.10	0.29	-25103.70	5.94	-25062.54	7.52	-25019.62	11.77	-24976.30	16.42	-24934.21	18.87	-24893.27	22.11
-25147.67	-0.27	-25104.47	5.03	-25063.24	6.82	-25021.53	9.86	-24977.22	15.50	-24936.86	16.15	-24891.32	24.06
-25147.67	-0.27	-25104.75	4.26	-25063.47	6.59	-25019.82	11.57	-24977.85	14.87	-24936.61	19.84	-24893.34	22.04
-25147.39	0.00	-25104.04	3.98	-25059.48	10.58	-25020.49	10.91	-24974.90	17.82	-24933.28	17.20	-24890.26	25.12
-25147.89	-0.50	-25105.59	4.69	-25062.02	8.04	-25021.27	10.12	-24977.88	14.84	-24931.43	17.45	-24894.78	20.61
-25147.02	0.37	-25105.02	3.14	-25062.69	7.37	-25019.36	12.03	-24974.16	18.56	-24931.19	20.78	-24892.19	23.20
-25147.67	-0.27	-25104.27	3.71	-25064.06	6.00	-25020.71	10.68	-24978.08	14.64	-24929.77	22.63	-24894.56	20.83
-25147.08	0.31	-25104.24	4.45	-25061.87	8.19	-25022.09	9.30	-24977.72	15.00	-24934.12	22.86	-24894.22	21.17
-25147.79	-0.40	-25103.84	4.49	-25061.72	8.34	-25020.12	11.27	-24977.78	14.95	-24934.14	24.28	-24893.73	21.65
-25147.59	-0.20	-25103.20	4.89	-25061.38	8.68	-25022.86	8.53	-24971.68	21.04	-24931.76	19.94	-24889.62	25.77
-25146.96	0.44	-25104.69	5.53	-25062.13	7.93	-25017.81	13.58	-24977.40	15.32	-24932.70	19.92	-24891.82	23.57
-25147.86	-0.47	-25103.71	4.04	-25060.39	9.67	-25022.08	9.31	-24973.64	19.09	-24935.64	22.29	-24894.15	21.24
-25145.88	1.52	-25105.26	5.02	-25062.83	7.23	-25018.62	12.77	-24977.92	14.80	-24933.09	21.36	-24892.20	23.19
-25148.11	-0.71	-25106.01	3.47	-25061.89	8.17	-25021.41	9.98	-24974.07	18.65	-24933.27	18.42	-24895.11	20.28
-25149.05	-1.66	-25106.09	2.72	-25062.74	7.32	-25019.67	11.72	-24977.41	15.32	-24936.32	20.97	-24890.88	24.50
-25149.42	-2.02	-25105.86	2.64	-25062.18	7.88	-25017.69	13.70	-24978.65	14.07	-24932.92	20.79	-24894.35	21.04
-25147.43	-0.03	-25104.64	2.86	-25063.00	7.06	-25022.28	9.11	-24976.35	16.37	-24934.90	17.74	-24895.26	20.13
-25149.07	-1.68	-25105.80	4.09	-25060.74	9.32	-25020.86	10.53	-24978.17	14.55	-24934.16	21.14	-24889.09	26.30
-25148.51	-1.11	-25105.33	2.93	-25060.87	9.19	-25021.41	9.98	-24976.55	16.17	-24934.79	19.16	-24891.33	24.06
-25146.71	0.69	-25104.25	3.40	-25060.98	9.08	-25019.50	11.89	-24977.93	14.80	-24933.67	19.90	-24886.11	29.28
-25148.61	-1.21	-25105.43	4.48	-25063.90	6.16	-25021.64	9.75	-24978.19	14.53	-24936.28	19.27	-24890.78	24.61
-25147.87	-0.48	-25105.71	3.30	-25062.44	7.62	-25020.69	10.70	-24977.29	15.43	-24935.91	20.39	-24892.14	23.25
-25148.59	-1.20	-25106.18	3.02	-25063.57	6.49	-25021.18	10.21	-24978.46	14.26	-24935.20	17.78	-24890.16	25.23
-25147.85	-0.46	-25104.90	2.55	-25062.97	7.09	-25018.27	13.12	-24976.00	16.73	-24936.12	18.14	-24894.01	21.38
-25148.05	-0.66	-25105.28	3.82	-25060.42	9.64	-25017.41	13.98	-24979.48	13.25	-24937.20	18.86	-24895.47	19.92
-25146.38	1.02	-25106.72	3.45	-25061.28	8.78	-25018.67	12.72	-24977.21	15.51	-24933.25	17.93	-24892.57	22.82
-25146.95	0.44	-25102.08	2.01	-25060.89	9.17	-25021.26	10.13	-24975.02	17.70	-24933.41	16.86	-24892.17	23.22
-25147.89	-0.50	-25106.05	6.64	-25060.78	9.28	-25018.24	13.16	-24975.07	17.65	-24936.67	20.81	-24891.27	24.12
-25148.53	-1.13	-25106.14	2.68	-25061.58	8.48	-25022.25	9.14	-24977.77	14.95	-24935.28	20.64	-24893.48	21.91
-25149.29	-1.89	-25104.31	2.58	-25061.87	8.19	-25018.64	12.75	-24977.75	14.97	-24934.53	17.39	-24894.97	20.42
-25147.82	-0.43	-25104.56	4.42	-25062.31	7.75	-25021.06	10.33	-24976.64	16.08	-24931.06	18.77	-24893.72	21.67
-25146.57	0.83	-25106.15	4.17	-25062.11	7.95	-25020.75	10.64	-24977.60	15.13	-24936.26	19.53	-24893.48	21.91

	-25148.35	-0.95	-25103.96	2.58	-25059.93	10.13	-25019.30	12.09	-24979.34	13.38	-24935.32	23.00	-24888.52	26.87
	-25148.73	-1.34	-25104.87	4.76	-25062.85	7.21	-25020.34	11.05	-24979.74	12.98	-24934.28	17.80	-24891.03	24.36
	-25148.43	-1.03	-25105.85	3.86	-25061.42	8.64	-25020.18	11.21	-24978.16	14.56	-24934.97	18.73	-24891.56	23.83
	-25147.99	-0.60	-25104.86	2.88	-25060.15	9.91	-25018.70	12.69	-24977.82	14.90	-24934.26	19.78	-24893.58	21.81
	-25148.20	-0.81	-25102.91	3.87	-25060.16	9.90	-25020.64	10.75	-24976.63	16.10	-24938.17	19.09	-24892.23	23.16
	-25147.52	-0.13	-25104.62	5.81	-25063.84	6.22	-25018.82	12.57	-24975.50	17.23	-24931.89	19.80	-24891.70	23.69
	-25148.09	-0.69	-25105.55	4.11	-25061.92	8.14	-25019.36	12.03	-24976.51	16.21	-24932.78	15.89	-24892.34	23.05
	-25147.92	-0.52	-25106.23	3.18	-25062.20	7.86	-25018.10	13.29	-24979.49	13.24	-24937.29	22.17	-24892.68	22.71
	-25148.51	-1.12	-25103.95	2.50	-25062.21	7.85	-25021.51	9.89	-24975.16	17.57	-24935.83	21.28	-24893.53	21.86
	-25148.16	-0.76	-25106.35	4.77	-25059.08	10.98	-25021.17	10.22	-24977.76				-24893.48	21.91
	-25148.04	-0.64	-25104.50	2.38	-25062.60	7.46			-24977.29				-24889.08	26.31
	-25147.80	-0.53	-25104.99	4.23	-25061.41	8.65			-24976.49				-24893.73	21.66
	-25147.75	-0.52	-25105.08	3.74	-25059.73	10.33			-24978.77				-24891.92	23.47
	-25148.04	-0.52	-25106.05	3.64										
	-25147.99	-0.51												
Average energies	-25147.92	-0.53	-25104.86	3.87	-25061.97	8.09	-25019.49	11.90	-24976.89	15.86	-24934.87	19.24	-24892.45	22.94

Th

	ground state energy	solid solution energy												
concentration in solid solution (%)	3		6		9		12		15		18		21	
number of atoms	2		4		6		8		10		12		14	
	-25153.93	-0.91	-25115.85	4.14	-25078.81	8.14	-25037.52	16.39	-25006.80	14.07	-24967.04	20.80	-24930.28	24.53
	-25153.94	-0.91	-25113.53	6.46	-25074.76	12.19	-25042.04	11.87	-25006.51	14.37	-24967.78	20.05	-24925.21	29.59
	-25153.63	-0.61	-25117.20	2.78	-25077.49	9.46	-25040.21	13.70	-25001.52	19.36	-24970.46	17.38	-24929.93	24.87
	-25154.06	-1.04	-25116.09	3.90	-25079.42	7.53	-25036.83	17.08	-25003.10	17.78	-24969.01	18.83	-24928.30	26.50
	-25153.28	-0.26	-25116.70	3.29	-25078.81	8.14	-25039.30	14.61	-25006.56	14.31	-24967.68	20.16	-24928.40	26.40
	-25153.31	-0.29	-25114.61	5.38	-25078.38	8.57	-25041.26	12.65	-25004.78	16.09	-24967.17	20.66	-24925.23	29.57
	-25153.50	-0.48	-25116.58	3.41	-25079.49	7.46	-25041.39	12.52	-25004.74	16.14	-24966.30	21.54	-24925.97	28.84
	-25153.17	-0.14	-25115.20	4.79	-25079.39	7.56	-25039.78	14.13	-25000.34	20.53	-24967.67	20.17	-24925.19	29.61
	-25153.22	-0.19	-25114.93	5.06	-25074.63	12.32	-25042.63	11.28	-25000.10	20.78	-24964.44	23.39	-24927.76	27.04
	-25152.68	0.35	-25116.82	3.17	-25078.39	8.56	-25041.49	12.42	-25003.26	17.62	-24967.29	20.55	-24925.29	29.51
	-25154.53	-1.50	-25117.32	2.67	-25079.20	7.75	-25041.73	12.18	-25003.19	17.68	-24960.87	26.97	-24931.56	23.24
	-25152.07	0.96	-25115.14	4.84	-25076.43	10.52	-25044.43	9.49	-25001.85	19.03	-24964.03	23.81	-24927.52	27.28
	-25153.70	-0.67	-25114.47	5.51	-25079.27	7.68	-25041.74	12.17	-25006.15	14.72	-24967.77	20.07	-24931.72	23.08
	-25152.62	0.40	-25115.92	4.06	-25079.48	7.47	-25039.60	14.31	-25004.32	16.55	-24964.51	23.33	-24928.27	26.53
	-25153.15	-0.13	-25116.51	3.48	-25077.37	9.58	-25040.73	13.19	-24999.98	20.90	-24964.80	23.04	-24926.39	28.41
	-25152.84	0.19	-25116.00	3.99	-25080.85	6.10	-25038.67	15.24	-25000.04	20.83	-24963.46	24.37	-24931.47	23.33
	-25154.42	-1.39	-25117.18	2.81	-25078.86	8.09	-25042.60	11.31	-25001.78	19.10	-24963.41	24.43	-24928.19	26.61

-25153.47	-0.45	-25116.08	3.91	-25076.43	10.52	-25036.68	17.23	-25004.21	16.66	-24964.61	23.23	-24927.36	27.44
-25151.84	1.18	-25115.76	4.22	-25075.32	11.63	-25043.97	9.94	-25002.79	18.08	-24964.35	23.49	-24928.66	26.14
-25153.74	-0.72	-25116.70	3.29	-25077.60	9.35	-25040.67	13.24	-25001.92	18.96	-24966.31	21.53	-24930.43	24.37
-25153.24	-0.22	-25115.06	4.93	-25078.89	8.06	-25040.02	13.89	-25005.47	15.41	-24967.36	20.48	-24925.52	29.28
-25153.60	-0.58	-25116.69	3.30	-25077.43	9.52	-25039.40	14.51	-25002.92	17.96	-24966.29	21.54	-24929.03	25.77
-25153.12	-0.09	-25115.90	4.09	-25078.70	8.25	-25040.38	13.53	-25004.93	15.94	-24967.00	20.84	-24928.11	26.69
-25153.85	-0.83	-25115.57	4.41	-25077.03	9.92	-25040.78	13.13	-25004.89	15.98	-24965.75	22.09	-24928.89	25.91
-25152.67	0.36	-25114.75	5.24	-25077.90	9.05	-25043.23	10.68	-25002.20	18.68	-24964.05	23.79	-24930.38	24.42
-25154.39	-1.37	-25115.19	4.80	-25077.33	9.62	-25038.77	15.15	-25003.47	17.41	-24966.48	21.36	-24931.08	23.72
-25153.42	-0.39	-25112.75	7.24	-25075.65	11.30	-25038.60	15.31	-25001.63	19.24	-24966.54	21.29	-24930.83	23.97
-25153.62	-0.60	-25116.68	3.30	-25075.19	11.76	-25038.24	15.67	-25000.94	19.94	-24963.77	24.07	-24930.50	24.30
-25152.68	0.35	-25116.06	3.92	-25078.87	8.08	-25040.81	13.10	-25001.19	19.69	-24967.30	20.54	-24930.84	23.97
-25154.72	-1.70	-25115.22	4.77	-25080.02	6.93	-25043.81	10.10	-25005.55	15.33	-24966.83	21.01	-24931.28	23.52
-25153.94	-0.92	-25117.85	2.14	-25080.89	6.06	-25042.22	11.69	-25003.72	17.16	-24967.61	20.23	-24928.24	26.56
-25152.39	0.63	-25113.89	6.09	-25079.91	7.04	-25040.03	13.88	-25004.78	16.09	-24967.54	20.30	-24928.62	26.18
-25153.29	-0.26	-25115.56	4.42	-25079.19	7.77	-25041.36	12.56	-25000.86	20.01	-24963.54	24.29	-24928.50	26.30
-25154.06	-1.03	-25116.78	3.20	-25075.68	11.27	-25040.83	13.09	-25006.92	13.95	-24967.47	20.37	-24929.68	25.12
-25153.98	-0.95	-25115.32	4.67	-25077.16	9.79	-25040.41	13.51	-25004.76	16.12	-24964.57	23.26	-24932.20	22.60
-25154.13	-1.10	-25114.97	5.02	-25076.75	10.20	-25040.95	12.97	-25007.28	13.60	-24965.86	21.98	-24926.39	28.41
-25153.39	-0.37	-25114.35	5.64	-25077.20	9.75	-25039.58	14.33	-25005.28	15.60	-24959.70	28.14	-24926.17	28.63
-25153.07	-0.04	-25116.69	3.30	-25077.54	9.41	-25041.58	12.33	-25004.68	16.20	-24966.29	21.54	-24931.13	23.67
-25153.78	-0.76	-25115.52	4.47	-25078.51	8.44	-25042.34	11.57	-25006.83	14.05	-24964.00	23.84	-24923.80	31.00
-25152.76	0.26	-25115.05	4.94	-25078.02	8.93	-25039.20	14.72	-25002.81	18.06	-24968.05	19.79	-24929.50	25.30
-25153.43	-0.40	-25115.48	4.51	-25076.95	10.00	-25041.92	11.99	-24999.46	21.42	-24966.00	21.84	-24930.83	23.97
-25153.13	-0.10	-25115.82	4.17	-25078.39	8.56	-25040.39	13.53	-25003.51	17.36	-24965.88	21.96	-24923.78	31.02
-25152.88	0.14	-25115.66	4.32	-25079.13	7.82	-25041.48	12.43	-25002.63	18.24	-24966.79	21.05	-24929.27	25.53
-25152.33	0.69	-25116.55	3.43	-25079.78	7.17	-25041.82	12.09	-25002.02	18.85	-24968.05	19.79	-24928.74	26.06
-25153.08	-0.06	-25116.69	3.30	-25076.48	10.47	-25042.96	10.95	-25003.90	16.98	-24960.31	27.53	-24921.65	33.16
-25152.26	0.76	-25116.27	3.71	-25080.94	6.01	-25041.77	12.14	-25004.15	16.73	-24964.59	23.25	-24928.22	26.58
-25152.42	0.60	-25114.92	5.07	-25078.07	8.88	-25041.64	12.27	-24998.60	22.28	-24966.43	21.41	-24928.33	26.47
-25153.74	-0.71	-25114.17	5.82	-25077.37	9.58	-25041.77	12.15	-25003.57	17.30	-24964.78	23.06	-24928.06	26.74
-25153.88	-0.86	-25114.40	5.59	-25079.66	7.29	-25040.40	13.51	-25005.11	15.76	-24967.87	19.96	-24930.37	24.43
-25153.22	-0.20	-25116.65	3.34	-25079.36	7.59	-25041.31	12.60	-25001.96	18.91	-24965.57	22.26	-24930.12	24.68
-25153.63	-0.61	-25115.93	4.06	-25079.30	7.65	-25041.61	12.30	-25005.36	15.52	-24964.51	23.33	-24929.06	25.74
-25154.22	-1.20	-25115.85	4.14	-25075.51	11.44	-25038.24	15.67	-25005.43	15.45	-24961.28	26.56	-24928.82	25.98
-25153.60	-0.57	-25115.70	4.29	-25078.09	8.86	-25041.42	12.49	-25000.70	20.17	-24967.82	20.01	-24929.90	24.90
-25153.74	-0.71	-25115.01	4.97	-25079.58	7.37	-25036.23	17.68	-25002.90	17.97	-24967.55	20.29	-24928.55	26.25
-25153.53	-0.51	-25116.19	3.79	-25076.38	10.57	-25035.70	18.22	-25003.39	17.49	-24968.27	19.57	-24922.97	31.83
-25152.24	0.78	-25115.79	4.19	-25077.31	9.64	-25042.66	11.25	-25003.70	17.18	-24966.64	21.19	-24930.82	23.98
-25151.40	1.62	-25115.48	4.50	-25080.08	6.87	-25041.38	12.54	-25002.27	18.61	-24966.25	21.59	-24927.47	27.33
-25153.52	-0.50	-25116.17	3.82	-25077.54	9.41	-25041.81	12.10	-25005.57	15.31	-24967.88	19.96	-24930.18	24.62

-25153.10	-0.07	-25116.77	3.21	-25077.59	9.36	-25039.70	14.22	-25005.19	15.69	-24966.60	21.24	-24927.87	26.93
-25153.46	-0.44	-25116.26	3.73	-25078.57	8.38	-25042.57	11.34	-25005.91	14.97	-24965.25	22.59	-24927.19	27.61
-25154.63	-1.61	-25114.98	5.01	-25079.47	7.48	-25043.20	10.71	-25004.96	15.91	-24965.13	22.71	-24929.04	25.76
-25152.77	0.26	-25116.35	3.64	-25078.33	8.62	-25038.57	15.35	-25002.23	18.64	-24965.63	22.20	-24933.25	21.55
-25154.23	-1.21	-25116.43	3.55	-25080.96	5.99	-25041.80	12.11	-25003.40	17.48	-24966.50	21.34	-24929.70	25.10
-25153.55	-0.53	-25115.21	4.78	-25077.61	9.35	-25039.47	14.44	-25004.96	15.91	-24969.84	18.00	-24928.93	25.87
-25153.26	-0.24	-25115.18	4.81	-25078.28	8.67	-25039.41	14.50	-25002.31	18.57	-24960.60	27.24	-24932.77	22.03
-25153.41	-0.38	-25117.19	2.79	-25078.83	8.12	-25041.70	12.21	-25000.53	20.34	-24967.05	20.79	-24929.16	25.64
-25153.31	-0.29	-25115.45	4.53	-25077.91	9.04	-25042.82	11.09	-25003.55	17.33	-24966.46	21.38	-24932.18	22.62
-25152.48	0.54	-25113.90	6.09	-25078.56	8.39	-25044.66	9.25	-25002.60	18.27	-24958.21	29.62	-24929.43	25.37
-25153.82	-0.80	-25113.57	6.42	-25078.52	8.43	-25043.04	10.88	-25005.11	15.77	-24965.03	22.80	-24931.56	23.24
-25153.29	-0.26	-25114.69	5.30	-25078.96	7.99	-25037.79	16.12	-25003.25	17.62	-24967.49	20.35	-24928.62	26.18
-25153.76	-0.73	-25114.56	5.43	-25078.28	8.67	-25042.46	11.45	-25002.41	18.47	-24966.39	21.45	-24929.70	25.10
-25153.36	-0.33	-25116.58	3.40	-25078.73	8.22	-25041.97	11.94	-25002.80	18.07	-24966.36	21.48	-24928.79	26.01
-25152.97	0.05	-25115.91	4.08	-25081.05	5.90	-25040.61	13.30	-25000.09	20.78	-24967.69	20.15	-24926.36	28.45
-25153.58	-0.56	-25114.73	5.25	-25077.36	9.59	-25036.93	16.98	-25001.90	18.97	-24965.62	22.22	-24930.88	23.93
-25152.96	0.07	-25116.34	3.65	-25079.55	7.40	-25041.89	12.02	-25006.63	14.25	-24966.94	20.90	-24926.38	28.42
-25152.96	0.06	-25114.13	5.86	-25078.73	8.22	-25037.55	16.37	-25000.08	20.80	-24968.13	19.71	-24929.64	25.16
-25152.65	0.38	-25115.97	4.01	-25079.63	7.32	-25040.07	13.84	-25001.71	19.17	-24966.34	21.50	-24932.18	22.63
-25154.16	-1.14	-25116.07	3.92	-25076.21	10.74	-25044.20	9.72	-24999.60	21.27	-24966.03	21.81	-24928.14	26.66
-25152.71	0.32	-25114.58	5.41	-25077.01	9.94	-25040.34	13.57	-25003.79	17.09	-24965.42	22.42	-24927.93	26.87
-25152.69	0.33	-25114.57	5.42	-25079.39	7.56	-25042.64	11.27	-25004.01	16.86	-24968.90	18.94	-24929.01	25.79
-25152.40	0.63	-25114.57	5.41	-25078.37	8.58	-25040.87	13.04	-25005.44	15.44	-24968.18	19.66	-24931.86	22.94
-25153.34	-0.32	-25117.21	2.78	-25079.55	7.40	-25038.72	15.19	-25004.33	16.54	-24959.59	28.25	-24930.05	24.75
-25152.45	0.57	-25116.44	3.55	-25077.00	9.95	-25042.17	11.75	-25005.19	15.69	-24966.17	21.67	-24929.17	25.63
-25154.17	-1.14	-25118.05	1.94	-25073.84	13.11	-25043.14	10.78	-25005.00	15.87	-24965.30	22.54	-24928.08	26.72
-25152.83	0.19	-25116.04	3.95	-25079.49	7.46	-25041.64	12.27	-25003.91	16.97	-24966.61	21.23	-24925.72	29.08
-25152.02	1.00	-25117.37	2.62	-25078.98	7.97	-25042.35	11.56	-25002.80	18.07	-24966.16	21.68	-24924.97	29.83
-25153.97	-0.95	-25117.42	2.57	-25079.63	7.32	-25041.45	12.47	-25001.56	19.31	-24962.26	25.58	-24922.08	32.72
-25153.37	-0.35	-25117.03	2.96	-25077.71	9.24	-25039.01	14.91	-25000.00	20.88	-24967.90	19.94	-24926.00	28.80
-25151.66	1.37	-25114.97	5.02	-25077.11	9.84	-25039.61	14.30	-25003.83	17.04	-24965.96	21.88	-24930.57	24.23
-25152.61	0.42	-25116.52	3.47	-25077.99	8.96	-25042.64	11.27	-25004.13	16.74	-24967.02	20.81	-24930.55	24.26
-25153.25	-0.22	-25116.66	3.32	-25078.85	8.10	-25041.33	12.58	-25004.76	16.12	-24963.03	24.81	-24932.22	22.58
-25154.04	-1.01	-25114.79	5.20	-25077.65	9.30	-25042.04	11.87	-25001.61	19.27	-24962.22	25.62	-24928.82	25.98
-25154.21	-1.19	-25117.84	2.15	-25080.67	6.28	-25038.27	15.65	-25004.23	16.64	-24968.71	19.13	-24931.92	22.89
-25153.23	-0.20	-25116.81	3.17	-25078.05	8.90	-25041.59	12.33	-25005.41	15.47	-24965.89	21.95	-24927.82	26.98
-25152.76	0.26	-25113.66	6.33	-25076.06	10.89	-25042.22	11.69	-25002.73	18.14	-24968.23	19.61	-24927.79	27.01
-25154.63	-1.60	-25116.02	3.97	-25079.74	7.21	-25044.22	9.69	-25002.74	18.13	-24967.80	20.04	-24930.22	24.58
-25152.65	0.37	-25117.39	2.60	-25079.72	7.23	-25040.04	13.88	-25003.45	17.43	-24965.52	22.31	-24930.23	24.57
-25153.29	-0.27	-25114.20	5.79	-25078.49	8.46	-25041.13	12.78	-25003.41	17.46	-24967.13	20.71	-24930.12	24.68
-25154.37	-1.35	-25115.58	4.41	-25080.06	6.89	-25039.07	14.84	-25000.47	20.41	-24967.79	20.05	-24927.64	27.16

	-25153.26	-0.23	-25114.46	5.53	-25078.76	8.19	-25040.70	13.21	-25000.31	20.56	-24967.95	19.89	-24926.62	28.18
	-25153.11	-0.09	-25117.72	2.26	-25076.50	10.45	-25044.01	9.90	-25006.25	14.62	-24968.04	19.80	-24928.67	26.13
	-25153.92	-0.89	-25116.74	3.24	-25077.61	9.34	-25040.53	13.38	-25000.34	20.53	-24966.43	21.40	-24931.65	23.15
	-25153.95	-0.92	-25116.17	3.82	-25073.21	13.74	-25036.07	17.84	-25006.17	14.71	-24967.14	20.70	-24927.60	27.20
	-25153.83	-0.80	-25116.73	3.26	-25077.15	9.80	-25041.14	12.77	-25005.86	15.01	-24967.57	20.27	-24928.57	26.23
	-25154.10	-1.07	-25116.73	3.25	-25079.50	7.45	-25041.76	12.15	-25005.58	15.30	-24964.58	23.26	-24928.80	26.00
	-25153.10	-0.08	-25113.69	6.29	-25078.35	8.60	-25041.04	12.88	-25000.00	20.88	-24965.15	22.69	-24930.93	23.87
	-25153.72	-0.70	-25116.30	3.69	-25075.29	11.66	-25041.75	12.16	-25001.60	19.27	-24962.73	25.10	-24931.49	23.31
	-25154.04	-1.02	-25116.19	3.80	-25075.65	11.30	-25041.38	12.53	-25003.41	17.47	-24966.59	21.24	-24932.13	22.67
	-25154.62	-1.59	-25115.55	4.44	-25076.01	10.94	-25040.21	13.70	-25001.58	19.30	-24964.55	23.29	-24928.20	26.61
	-25154.31	-1.29	-25114.97	5.01	-25078.77	8.18	-25041.73	12.18	-25003.12	17.75	-24966.99	20.85	-24929.05	25.75
	-25150.58	2.44	-25113.55	6.44	-25076.84	10.11	-25042.79	11.12	-25003.44	17.43	-24966.61	21.23	-24926.46	28.34
	-25153.61	-0.59	-25116.65	3.34	-25080.71	6.24	-25042.07	11.84	-25000.59	20.28	-24963.66	24.18	-24929.78	25.02
	-25154.73	-1.71	-25114.98	5.01	-25079.08	7.87	-25039.45	14.47	-25002.02	18.85	-24967.12	20.72	-24929.29	25.51
	-25154.55	-1.52	-25115.40	4.59	-25080.56	6.39	-25040.40	13.51	-25002.90	17.98	-24966.26	21.58	-24929.24	25.56
	-25153.67	-0.65	-25115.90	4.08	-25077.21	9.74	-25041.87	12.04	-25005.24	15.64	-24967.13	20.71	-24934.39	20.42
	-25153.57	-0.55	-25115.84	4.15	-25078.53	8.42	-25044.49	9.42	-25002.32	18.55	-24965.81	22.03	-24932.74	22.06
	-25154.36	-1.33			-25079.92	7.03			-25000.99	19.88			-24927.73	27.07
	-25154.45	-1.42							-24993.78	27.09				
	-25152.96	0.06							-25004.30	16.58				
Average energies	-25153.38	-0.36	-25115.75	4.24	-25078.17	8.78	-25040.92	12.99	-25003.20	17.67	-24965.94	21.90	-24928.88	25.92

U

	ground state energy	solid solution energy												
concentration in solid solution (%)	3		6		9		12		15		18		21	
number of atoms	2		4		6		8		10		12		14	
	-25157.61	0.43	-25124.24	5.79	-25093.63	8.37	-25053.70	20.28	-25030.65	15.32	-24995.27	22.68	-24963.06	26.88
	-25158.64	-0.59	-25125.35	4.68	-25090.38	11.63	-25061.51	12.48	-25028.79	17.18	-25000.52	17.43	-24963.97	25.96
	-25157.72	0.32	-25126.55	3.47	-25092.51	9.50	-25061.47	12.52	-25029.46	16.51	-24998.94	19.01	-24963.50	26.43
	-25157.80	0.24	-25126.73	3.29	-25096.23	5.78	-25061.21	12.78	-25029.43	16.54	-24992.21	25.74	-24963.56	26.38
	-25157.72	0.32			-25090.87	11.13	-25063.02	10.96	-25024.32	21.65	-24997.42	20.53	-24967.01	22.93
	-25156.42	1.62	-25126.07	3.96	-25091.61	10.39	-25063.01	10.98	-25028.76	17.21	-25000.35	17.60	-24964.90	25.03
	-25158.69	-0.64	-25126.55	3.47	-25091.09	10.92	-25061.09	12.89	-25027.55	18.42	-24997.88	20.08	-24966.36	23.57
	-25157.79	0.26	-25124.60	5.42	-25091.07	10.94	-25058.87	15.12	-25030.36	15.61	-24995.28	22.67	-24966.89	23.05
	-25156.62	1.43	-25126.45	3.57	-25091.87	10.13	-25063.84	10.15	-25030.55	15.42	-24994.50	23.45	-24964.67	25.26
	-25157.69	0.35	-25126.97	3.05	-25090.38	11.62	-25061.16	12.83	-25032.65	13.32	-24997.00	20.95	-24969.54	20.39
	-25151.87	6.17	-25116.61	13.41	-25092.38	9.62	-25062.58	11.41	-25027.13	18.83	-24999.53	18.42	-24968.59	21.34
	-25158.48	-0.44	-25126.19	3.83	-25093.02	8.98	-25057.55	16.44	-25027.80	18.17	-24999.60	18.35	-24962.68	27.25

-25157.93	0.11	-25125.33	4.69	-25093.28	8.73	-25057.23	16.76	-25031.70	14.27	-24997.54	20.41	-24962.91	27.03
-25158.69	-0.65	-25124.05	5.98	-25093.64	8.37	-25059.64	14.34	-25030.56	15.41	-24996.79	21.17	-24962.63	27.30
-25159.26	-1.22	-25125.75	4.27	-25092.30	9.71	-25061.53	12.46	-25029.88	16.09	-24995.69	22.26	-24965.49	24.44
-25157.50	0.54	-25125.39	4.64	-25092.04	9.97	-25060.96	13.02	-25029.71	16.26	-24994.17	23.78	-24968.66	21.27
-25158.82	-0.77	-25125.86	4.17	-25093.01	9.00	-25060.80	13.19	-25028.40	17.57	-24998.80	19.15	-24963.40	26.53
-25157.62	0.42	-25121.62	8.41	-25089.99	12.01	-25059.17	14.82	-25029.01	16.96	-24996.78	21.18	-24963.66	26.27
-25157.09	0.95	-25125.96	4.06	-25091.82	10.18	-25057.39	16.59	-25029.79	16.18	-24997.11	20.84	-24966.34	23.59
-25158.86	-0.82	-25124.69	5.33	-25091.56	10.45	-25060.24	13.75	-25028.92	17.05	-24996.91	21.04	-24965.10	24.83
-25157.73	0.31	-25125.78	4.25	-25093.37	8.64	-25061.05	12.94	-25029.35	16.62	-24998.43	19.52		
-25157.51	0.53	-25126.88	3.14	-25092.74	9.27	-25058.06	15.93	-25023.72	22.25	-24997.00	20.95	-24964.79	25.14
-25155.51	2.53	-25123.49	6.53	-25092.66	9.35	-25061.58	12.41	-25030.31	15.66	-24998.90	19.05	-24962.42	27.52
-25157.10	0.94	-25124.16	5.87	-25092.44	9.56	-25060.01	13.97	-25029.96	16.01	-24999.20	18.75	-24961.36	28.57
-25157.87	0.18	-25125.87	4.16	-25090.60	11.40	-25058.40	15.59	-25030.15	15.82	-24997.63	20.32	-24965.45	24.48
-25158.77	-0.73	-25125.24	4.79	-25093.07	8.94	-25059.21	14.78	-25028.00	17.97	-24997.56	20.39	-24966.19	23.75
-25156.40	1.64	-25122.94	7.09	-25094.81	7.19	-25060.46	13.53	-25033.10	12.86	-24997.98	19.98	-24965.80	24.13
-25152.13	5.91	-25124.94	5.08	-25094.16	7.85	-25060.83	13.16	-25027.94	18.03	-24998.38	19.57	-24963.07	26.86
-25158.71	-0.66	-25123.62	6.41	-25092.83	9.18	-25061.65	12.34	-25030.20	15.77	-25000.87	17.08	-24968.78	21.15
-25151.49	6.56	-25125.95	4.08	-25094.42	7.59	-25063.00	10.98	-25031.81	14.16	-24995.73	22.22	-24966.47	23.46
-25158.85	-0.81	-25127.08	2.95	-25091.00	11.01	-25061.52	12.47	-25024.34	21.63	-24997.04	20.91	-24964.89	25.04
-25158.18	-0.14	-25119.36	10.66	-25095.15	6.86	-25058.86	15.13	-25027.89	18.08	-24994.38	23.57	-24964.56	25.37
-25158.82	-0.78	-25126.19	3.83	-25092.74	9.27	-25060.91	13.08	-25023.03	22.94	-24997.25	20.70	-24965.50	24.43
-25156.82	1.22	-25125.36	4.66	-25093.04	8.97	-25063.38	10.61	-25024.38	21.59	-24994.42	23.53	-24966.96	22.98
-25150.82	7.22	-25127.01	3.02	-25090.94	11.06	-25063.38	10.61	-25029.28	16.69	-24997.63	20.32	-24957.90	32.03
-25150.25	7.79	-25123.18	6.84	-25093.10	8.91	-25059.70	14.28	-25030.82	15.15	-24993.19	24.76	-24952.61	37.32
-25157.95	0.09	-25126.35	3.68	-25094.72	7.29	-25059.28	14.71	-25023.72	22.25	-24996.64	21.31	-24966.76	23.17
-25158.20	-0.16	-25125.91	4.11	-25093.16	8.85	-25063.67	10.32	-25026.53	19.44	-24996.74	21.21	-24964.20	25.73
-25151.05	6.99	-25123.71	6.32	-25095.03	6.98	-25058.10	15.88	-25029.10	16.87	-24998.26	19.69	-24965.35	24.58
-25158.84	-0.80	-25126.64	3.38	-25090.85	11.16	-25061.92	12.07	-25027.51	18.46	-24998.85	19.10	-24963.15	26.78
-25157.67	0.37	-25123.85	6.18	-25094.38	7.63	-25061.81	12.18	-25029.94	16.03	-24995.97	21.98	-24966.08	23.85
-25151.38	6.66	-25126.81	3.22	-25095.09	6.91	-25056.61	17.38	-25030.11	15.86	-24998.32	19.63	-24963.58	26.36
-25158.10	-0.05	-25126.73	3.30	-25094.48	7.52	-25058.43	15.56	-25030.20	15.77	-25000.43	17.52	-24969.37	20.57
-25156.98	1.06	-25125.51	4.51	-25091.79	10.22	-25060.10	13.89	-25030.21	15.76	-24996.31	21.64	-24968.29	21.65
-25155.91	2.14	-25123.84	6.18	-25094.43	7.58	-25061.45	12.54	-25029.31	16.66	-24996.27	21.68	-24965.49	24.44
-25157.55	0.49	-25124.67	5.36	-25094.68	7.32	-25058.34	15.64	-25029.47	16.50	-24995.33	22.62	-24964.50	25.44
-25156.71	1.33	-25126.08	3.95	-25092.65	9.35	-25062.06	11.92	-25029.45	16.52	-24994.27	23.68	-24967.68	22.25
-25157.98	0.06	-25123.48	6.55	-25091.86	10.15	-25063.24	10.75	-25029.69	16.28	-24994.26	23.69	-24963.33	26.60
-25156.10	1.94	-25126.67	3.35	-25094.57	7.43	-25060.20	13.78	-25032.69	13.28	-24995.91	22.04	-24967.83	22.10
-25158.30	-0.26	-25124.68	5.34	-25090.76	11.25	-25062.55	11.44	-25024.83	21.13	-24998.13	19.82	-24966.64	23.30
-25157.82	0.22	-25119.71	10.32	-25089.99	12.01	-25057.11	16.87	-25028.37	17.60	-24999.94	18.01	-24965.37	24.56
-25158.45	-0.41	-25126.84	3.19	-25093.39	8.62	-25062.47	11.52	-25030.67	15.30	-24995.94	22.01	-24968.03	21.90
-25158.80	-0.76	-25125.82	4.21	-25091.85	10.16	-25060.89	13.10	-25029.49	16.48	-24993.65	24.30	-24966.88	23.06

-25156.38	1.66	-25126.50	3.53	-25095.34	6.66	-25057.53	16.46	-25030.10	15.87	-24997.33	20.62	-24964.62	25.31
-25156.98	1.07	-25125.46	4.57	-25093.76	8.25	-25061.87	12.12	-25031.20	14.77	-24997.62	20.33	-24966.37	23.56
-25159.06	-1.02	-25125.30	4.73	-25092.09	9.91	-25060.73	13.26	-25029.11	16.86	-24995.46	22.49	-24967.56	22.38
-25158.00	0.05	-25123.64	6.38	-25091.15	10.85	-25059.27	14.72	-25029.38	16.58	-24997.88	20.07	-24968.50	21.43
-25158.08	-0.04	-25125.34	4.68	-25092.36	9.64	-25060.65	13.34	-25031.31	14.66	-24994.80	23.15	-24967.56	22.37
-25156.09	1.95	-25124.22	5.81	-25092.00	10.00	-25062.65	11.34	-25026.45	19.51	-24998.32	19.63	-24963.46	26.48
-25158.75	-0.71	-25124.96	5.06	-25094.06	7.95	-25061.48	12.51	-25030.49	15.48	-24998.38	19.57	-24961.16	28.78
-25157.18	0.87	-25125.65	4.38	-25092.35	9.66	-25061.73	12.25	-25025.33	20.64	-24997.71	20.24	-24965.81	24.13
-25158.01	0.03	-25125.47	4.55	-25092.15	9.85	-25060.74	13.25	-25027.83	18.14	-24998.77	19.18	-24966.36	23.57
-25158.31	-0.27	-25123.41	6.62	-25093.35	8.66	-25060.85	13.14	-25031.30	14.67	-24998.34	19.61	-24965.40	24.54
-25157.57	0.48	-25125.16	4.87	-25093.16	8.84	-25062.45	11.54	-25026.54	19.43	-24998.29	19.66	-24964.84	25.10
-25158.00	0.05	-25125.51	4.51	-25089.04	12.96	-25057.43	16.56	-25028.33	17.64	-24992.70	25.25	-24965.61	24.33
-25156.55	1.50	-25126.61	3.42	-25092.92	9.09	-25061.73	12.25	-25028.61	17.36	-24994.84	23.11	-24965.13	24.80
-25157.61	0.43	-25125.70	4.32	-25091.00	11.00	-25057.79	16.20	-25030.28	15.69	-24998.10	19.85	-24965.73	24.20
-25157.67	0.38	-25124.53	5.50	-25092.72	9.29	-25059.27	14.71	-25030.63	15.34	-24994.70	23.25	-24968.14	21.79
-25157.99	0.05	-25126.08	3.95	-25095.18	6.83	-25063.35	10.64	-25025.47	20.50	-24998.09	19.86	-24961.24	28.70
-25157.18	0.86	-25126.45	3.58	-25093.90	8.11	-25062.45	11.54	-25028.93	17.04	-24999.97	17.98	-24965.99	23.94
-25158.16	-0.12	-25126.53	3.49	-25092.90	9.11	-25057.88	16.10	-25026.61	19.36	-24995.89	22.06	-24967.35	22.58
-25158.97	-0.92	-25124.39	5.64	-25091.75	10.26	-25062.98	11.01	-25023.72	22.25	-24995.30	22.66	-24961.89	28.04
-25157.79	0.25	-25125.75	4.28	-25094.46	7.54	-25062.48	11.51	-25031.93	14.04	-24995.51	22.44	-24966.71	23.22
-25156.61	1.43	-25127.33	2.69	-25093.72	8.29	-25060.58	13.40	-25030.38	15.59	-24990.39	27.56	-24960.59	29.34
-25157.81	0.23	-25124.75	5.27	-25093.72	8.29	-25064.34	9.65	-25026.95	19.02	-24997.97	19.98	-24967.19	22.74
-25157.04	1.01	-25125.78	4.25	-25094.41	7.60	-25062.44	11.55	-25029.63	16.34	-24998.97	18.98		
-25157.79	0.25	-25126.66	3.36	-25092.14	9.86	-25059.18	14.80	-25027.12	18.85	-24996.48	21.47		
-25158.21	-0.17	-25124.64	5.38	-25088.73	13.27	-25061.79	12.20	-25031.31	14.66	-25000.45	17.50		
-25157.97	0.08	-25127.13	2.89	-25094.20	7.81	-25062.31	11.68	-25032.02	13.94	-24998.70	19.25		
-25159.43	-1.39	-25124.30	5.73	-25093.15	8.86	-25063.89	10.10	-25029.85	16.12	-24994.88	23.07		
-25157.45	0.60	-25125.82	4.20	-25093.11	8.89			-25028.98	16.99	-24998.14	19.81		
-25159.50	-1.46	-25126.61	3.42	-25093.11	8.90			-25027.40	18.57	-24998.19	19.76		
-25157.62	0.43	-25125.27	4.75	-25094.18	7.82			-25030.49	15.48	-24995.41	22.54		
-25158.32	-0.28	-25120.28	9.75	-25092.65	9.36			-25030.28	15.69	-24998.05	19.90		
-25156.87	1.17	-25125.94	4.09	-25094.73	7.28			-25029.23	16.74	-24995.65	22.31		
-25158.01	0.03	-25125.74	4.29	-25091.31	10.70			-25027.14	18.83	-24997.07	20.88		
-25156.72	1.32			-25091.89	10.11			-25025.53	20.44	-24999.94	18.01		
-25158.50	-0.45							-25028.10	17.87				
-25157.22	0.82							-25030.25	15.72				
-25156.88	1.17												
-25156.98	1.06												
-25158.63	-0.58												
-25156.68	1.36												
-25156.69	1.36												

Average energies	-25157.26	0.78	-25125.13	4.89	-25092.76	9.25	-25060.70	13.29	-25028.87	17.10	-24997.03	20.92	-24965.13	24.81

$Ca_{1-x}M_xZrTi_{2-2x}Al_{2x}O_7$ M=(Ce, Pu, Th, U)

Ti(1)Ti(2)

N.B. All stated energies are in eV

Ce

	ground state energy	solid solution energy												
concentration in solid solution (%)	3		6		9		12		15		18		21	
number of atoms	2		4		6		8		10		12		13	
	-25155.94	-2.56	-25122.29	-1.59	-25090.64	-2.63	-25048.09	7.25	-25014.59	8.07	-24975.73	14.24	-24966.45	7.17
	-25157.73	-4.35	-25122.52	-1.82	-25088.36	-0.34	-25050.89	4.45	-25019.31	3.34	-24986.68	3.29	-24967.26	6.37
	-25156.60	-3.22	-25123.79	-3.09	-25089.13	-1.11	-25053.25	2.08	-25020.13	2.52	-24985.29	4.68	-24971.42	2.21
	-25154.28	-0.90	-25123.52	-2.82	-25088.54	-0.52	-25047.98	7.35	-25021.12	1.54	-24982.56	7.41	-24969.36	4.27
	-25157.85	-4.47	-25121.25	-0.55	-25087.83	0.19	-25055.07	0.27	-25019.93	2.72	-24988.21	1.76	-24968.91	4.72
	-25156.72	-3.34	-25119.46	1.23	-25088.28	-0.27	-25054.58	0.75	-25021.14	1.51	-24984.82	5.15	-24967.62	6.01
	-25156.45	-3.07	-25122.40	-1.70	-25086.11	1.91	-25051.98	3.35	-25020.01	2.64	-24980.82	9.15	-24967.73	5.90
	-25157.50	-4.12	-25122.48	-1.78	-25085.29	2.73	-25052.42	2.92	-25018.83	3.82	-24984.33	5.64	-24966.97	6.66
	-25156.32	-2.94	-25120.83	-0.14	-25086.37	1.65	-25056.89	-1.56	-25018.05	4.60	-24984.51	5.46	-24967.31	6.32
	-25156.90	-3.52	-25121.70	-1.00	-25090.56	-2.55	-25053.73	1.61	-25059.83	-37.18	-24985.19	4.78	-24969.59	4.04
	-25157.56	-4.18	-25120.91	-0.21	-25086.69	1.32	-25053.41	1.92	-25015.89	6.76	-24985.38	4.59	-24968.74	4.89
	-25156.60	-3.22	-25120.75	-0.05	-25085.89	2.12	-25054.78	0.55	-25015.42	7.23	-24986.62	3.35	-24970.52	3.10
	-25156.41	-3.03	-25123.07	-2.37	-25089.23	-1.22	-25054.32	1.01	-25056.90	-34.25	-24983.84	6.13	-24965.25	8.38
	-25158.28	-4.90	-25121.78	-1.09	-25086.95	1.07	-25052.15	3.19	-25018.95	3.70	-24988.99	0.98	-24966.73	6.90
	-25155.64	-2.26	-25123.55	-2.85	-25087.81	0.21	-25054.47	0.86	-25019.79	2.86	-24984.38	5.59	-24961.65	11.98
	-25157.62	-4.24	-25122.91	-2.21	-25084.70	3.31	-25055.74	-0.41	-25022.19	0.46	-24982.58	7.39	-24965.14	8.49
	-25156.04	-2.66	-25120.53	0.16	-25085.52	2.49	-25048.34	6.99	-25016.75	5.90	-24983.56	6.41	-24968.57	5.06
	-25157.36	-3.98	-25121.47	-0.77	-25085.14	2.88	-25051.56	3.77	-25021.69	0.97	-24987.22	2.75	-24959.05	14.58
	-25158.77	-5.39	-25122.86	-2.16	-25087.40	0.61	-25055.02	0.32	-25017.90	4.75	-24987.16	2.81	-24967.95	5.68
	-25157.46	-4.08	-25122.71	-2.01	-25088.35	-0.33	-25053.50	1.83	-25013.43	9.22	-24986.98	2.99	-24968.14	5.49
	-25157.13	-3.75	-25121.19	-0.49	-25086.72	1.30	-25055.64	-0.31	-25018.18	4.47	-24984.02	5.95	-24971.14	2.49
	-25157.71	-4.33	-25122.38	-1.68	-25088.98	-0.97	-25052.86	2.48	-25021.86	0.79	-24984.59	5.38	-24969.08	4.55
	-25156.16	-2.78	-25121.04	-0.34	-25087.83	0.19	-25054.18	1.16	-25018.45	4.20	-24985.22	4.75	-24960.42	13.21
	-25157.46	-4.08	-25121.16	-0.46	-25087.97	0.05	-25049.57	5.76	-25019.57	3.08	-24982.09	7.88	-24964.03	9.60
	-25157.40	-4.03	-25122.61	-1.91	-25087.90	0.11	-25050.54	4.79	-25019.73	2.92	-24985.78	4.19	-24961.84	11.79
	-25157.09	-3.71	-25123.53	-2.83	-25085.63	2.38	-25053.37	1.97	-25011.39	11.26	-24987.66	2.31	-24965.61	8.02
	-25158.03	-4.65	-25123.19	-2.50	-25088.95	-0.94	-25050.31	5.02	-25017.73	4.93	-24986.01	3.96	-24966.18	7.44
	-25156.54	-3.16	-25122.48	-1.78	-25087.61	0.41	-25051.02	4.32	-25018.40	4.25	-24983.02	6.95	-24963.84	9.79
	-25156.84	-3.46	-25120.82	-0.12	-25089.88	-1.86	-25055.46	-0.13	-25018.83	3.82	-24984.03	5.94	-24968.91	4.72

-25156.31	-2.93	-25118.98	1.72	-25087.45	0.57	-25055.96	-0.63	-25014.72	7.93	-24982.87	7.10	-24971.67	1.96
-25158.10	-4.72	-25123.41	-2.71	-25089.22	-1.20	-25051.76	3.57	-25018.81	3.84	-24983.00	6.97	-24969.03	4.60
-25156.66	-3.28	-25121.69	-1.00	-25087.68	0.33	-25055.71	-0.38	-25021.73	0.92	-24974.22	15.75	-24966.26	7.36
-25156.38	-3.00	-25123.68	-2.98	-25087.78	0.24	-25054.81	0.53	-25017.30	5.35	-24987.26	2.71	-24961.31	12.32
-25156.44	-3.06	-25122.52	-1.83	-25085.06	2.96	-25052.96	2.38	-25018.30	4.35	-24981.65	8.32	-24965.01	8.62
-25157.66	-4.28	-25123.22	-2.52	-25088.27	-0.25	-25054.70	0.64	-25022.58	0.08	-24981.08	8.89	-24970.34	3.28
-25157.43	-4.05	-25122.45	-1.75	-25089.51	-1.50	-25054.97	0.36	-25022.23	0.42	-24984.89	5.08	-24971.93	1.70
-25155.98	-2.60	-25124.19	-3.50	-25087.04	0.97	-25053.45	1.88	-25020.95	1.70	-24982.45	7.52	-24964.59	9.03
-25156.44	-3.06	-25123.55	-2.86	-25087.49	0.52	-25055.12	0.21	-25017.01	5.64	-24983.84	6.13	-24970.70	2.93
-25157.39	-4.01	-25122.61	-1.91	-25087.54	0.48	-25054.44	0.89	-25017.07	5.58	-24985.46	4.51	-24965.60	8.03
-25156.30	-2.92	-25121.71	-1.02	-25089.11	-1.09	-25055.31	0.02	-25020.49	2.16	-24986.23	3.74	-24966.29	7.34
-25156.89	-3.51	-25119.87	0.82	-25087.98	0.03	-25055.64	-0.30	-25020.24	2.41	-24987.09	2.88	-24962.93	10.70
-25155.17	-1.79	-25122.62	-1.92	-25088.14	-0.13	-25055.13	0.20	-25019.05	3.60	-24985.05	4.92	-24968.69	4.94
-25157.93	-4.55	-25124.41	-3.71	-25090.40	-2.38	-25052.43	2.90	-25018.68	3.97	-24986.40	3.57	-24963.42	10.21
-25155.56	-2.18	-25121.95	-1.25	-25087.71	0.30	-25053.07	2.26	-25021.38	1.27	-24980.28	9.69	-24969.48	4.15
-25157.24	-3.86	-25122.89	-2.20	-25085.20	2.82	-25054.14	1.19	-25021.50	1.15	-24979.62	10.35	-24965.33	8.30
-25157.16	-3.78	-25121.85	-1.15	-25088.04	-0.03	-25054.32	1.01	-25021.15	1.50	-24988.14	1.83	-24968.99	4.63
-25156.71	-3.33	-25121.75	-1.05	-25087.82	0.20	-25055.72	-0.39	-25020.06	2.59	-24981.72	8.25	-24967.03	6.60
-25157.31	-3.93	-25122.40	-1.70	-25088.63	-0.61	-25056.59	-1.26	-25019.68	2.97	-24986.74	3.23	-24969.93	3.69
-25157.18	-3.80	-25122.92	-2.23	-25088.21	-0.19	-25052.88	2.45	-25020.85	1.80	-24983.98	5.99	-24967.54	6.09
-25156.28	-2.90	-25123.87	-3.17	-25089.06	-1.04	-25056.33	-1.00	-25021.13	1.52	-24988.66	1.31	-24967.30	6.33
-25155.79	-2.41	-25123.26	-2.56	-25087.59	0.43	-25048.10	7.24	-25020.72	1.93	-24989.90	0.07	-24969.76	3.87
-25156.05	-2.67	-25122.03	-1.34	-25088.30	-0.29	-25055.65	-0.32	-25019.14	3.51	-24980.74	9.23	-24965.29	8.34
-25157.02	-3.64	-25119.00	1.69	-25089.99	-1.97	-25054.70	0.64	-25020.56	2.09	-24986.19	3.78	-24967.39	6.24
-25156.76	-3.38	-25120.64	0.06	-25088.57	-0.56	-25048.51	6.83	-25014.23	8.42	-24984.14	5.83	-24970.62	3.01
-25157.07	-3.69	-25123.30	-2.61	-25089.35	-1.34	-25052.75	2.58	-25021.15	1.50	-24985.64	4.33	-24970.68	2.95
-25157.24	-3.86	-25123.45	-2.75	-25088.32	-0.30	-25052.93	2.40	-25011.91	10.74	-24986.20	3.77	-24967.69	5.93
-25157.87	-4.49	-25119.25	1.45	-25088.86	-0.84	-25054.31	1.02	-25018.82	3.83	-24983.21	6.76	-24965.61	8.02
-25155.74	-2.36	-25121.29	-0.59	-25086.75	1.26	-25053.51	1.82	-25018.06	4.59	-24987.60	2.37	-24969.88	3.75
-25156.67	-3.29	-25119.29	1.41	-25077.79	10.22	-25049.35	5.98	-25020.04	2.61	-24986.64	3.32	-24966.20	7.43
-25155.57	-2.19	-25121.72	-1.02	-25088.65	-0.63	-25052.39	2.94	-25017.34	5.31	-24985.15	4.82	-24967.76	5.87
-25157.23	-3.85	-25124.45	-3.76	-25088.71	-0.69	-25052.95	2.39	-25018.22	4.43	-24983.39	6.58	-24970.60	3.03
-25157.46	-4.08	-25121.85	-1.15	-25083.20	4.81	-25055.25	0.08	-25020.63	2.02	-24987.39	2.58	-24966.81	6.81
-25157.23	-3.85	-25120.99	-0.30	-25087.90	0.12	-25051.95	3.38	-25020.30	2.35	-24983.84	6.13	-24967.00	6.63
-25157.16	-3.78	-25122.67	-1.97	-25087.38	0.63	-25053.65	1.68	-25018.70	3.95	-24985.11	4.86	-24972.45	1.18
-25156.92	-3.54	-25122.33	-1.63	-25089.29	-1.27	-25054.91	0.42	-25020.21	2.44	-24988.17	1.80	-24966.54	7.09
-25156.14	-2.76	-25122.16	-1.46	-25086.91	1.10	-25054.19	1.14	-25022.08	0.57	-24984.07	5.90	-24970.08	3.55
-25156.56	-3.18	-25122.90	-2.21	-25087.36	0.66	-25055.91	-0.58	-25020.27	2.38	-24983.82	6.15	-24962.30	11.33
-25156.93	-3.55	-25122.64	-1.95	-25089.02	-1.01	-25053.31	2.02	-25021.01	1.64	-24979.81	10.15	-24970.40	3.22
-25156.93	-3.55	-25119.19	1.51	-25089.35	-1.33	-25053.57	1.76	-25017.29	5.36	-24981.78	8.19	-24970.30	3.33
-25154.73	-1.35	-25122.61	-1.91	-25087.28	0.73	-25053.02	2.31	-25019.12	3.53	-24988.41	1.56	-24964.67	8.95

-25156.22	-2.84	-25122.25	-1.55	-25081.14	6.88	-25055.76	-0.43	-25019.22	3.43	-24986.16	3.81	-24972.46	1.17
-25157.96	-4.59	-25122.69	-1.99	-25086.64	1.37	-25050.06	5.27	-25022.64	0.02	-24984.83	5.14	-24968.64	4.99
-25157.86	-4.48	-25122.97	-2.27	-25088.50	-0.48	-25052.66	2.67	-25020.83	1.82	-24983.64	6.33	-24971.58	2.05
-25157.97	-4.59	-25121.01	-0.31	-25087.24	0.77	-25056.16	-0.83	-25017.85	4.80	-24983.68	6.29	-24971.70	1.93
-25156.82	-3.44	-25119.87	0.83	-25088.85	-0.83	-25053.63	1.70	-25013.93	8.72	-24987.75	2.21	-24965.32	8.31
-25156.84	-3.46	-25123.64	-2.94	-25088.77	-0.75	-25056.50	-1.16	-25020.68	1.98	-24983.24	6.73	-24967.13	6.50
-25156.66	-3.28	-25121.77	-1.08	-25087.44	0.57	-25051.81	3.53	-25019.40	3.25	-24981.38	8.59	-24971.63	2.00
-25157.98	-4.60	-25121.30	-0.60	-25085.96	2.06	-25051.91	3.42	-25018.30	4.35	-24986.94	3.03	-24971.54	2.09
-25156.37	-2.99	-25122.28	-1.58	-25088.14	-0.13	-25053.64	1.70	-25018.90	3.75	-24983.64	6.33	-24964.17	9.46
-25157.64	-4.26	-25122.63	-1.93	-25089.07	-1.06	-25053.51	1.82	-25020.50	2.15	-24980.76	9.21	-24966.16	7.47
-25157.51	-4.13	-25123.40	-2.71	-25087.45	0.57	-25054.56	0.77	-25021.46	1.19	-24987.21	2.76	-24968.52	5.10
-25157.46	-4.08	-25123.09	-2.39	-25088.46	-0.44	-25052.20	3.13	-25019.61	3.04	-24836.29	153.68	-24967.05	6.58
-25155.13	-1.75	-25121.09	-0.40	-25089.33	-1.31	-25053.89	1.44	-25020.30	2.36	-24982.07	7.90	-24970.39	3.24
-25157.73	-4.35	-25120.27	0.43	-25088.99	-0.98	-25046.53	8.80	-25012.34	10.31	-24981.10	8.87	-24971.10	2.52
-25156.95	-3.57	-25123.12	-2.42	-25088.24	-0.22	-25055.96	-0.62	-25018.30	4.35	-24982.57	7.40	-24969.27	4.36
-25157.23	-3.85	-25121.24	-0.55	-25085.41	2.61	-25054.05	1.29	-25020.35	2.30	-24987.18	2.79	-24969.01	4.62
-25156.18	-2.80	-25119.42	1.28	-25089.38	-1.36	-25054.96	0.38	-25018.38	4.27	-24986.71	3.26	-24966.13	7.50
-25155.93	-2.55	-25123.55	-2.85	-25089.39	-1.37	-25052.36	2.97	-25017.12	5.54	-24982.63	7.34	-24968.71	4.91
-25157.80	-4.42	-25121.96	-1.26	-25087.15	0.86	-25052.30	3.03	-25019.29	3.36	-25028.47	-38.50	-24966.80	6.83
-25157.01	-3.63	-25120.78	-0.08	-25087.37	0.65	-25053.20	2.13	-25021.38	1.27	-24984.77	5.20	-24966.01	7.62
-25156.28	-2.90	-25122.49	-1.79	-25089.30	-1.28	-25055.86	-0.53	-25014.01	8.64	-24981.60	8.37	-24963.33	10.30
-25155.85	-2.47	-25121.87	-1.17	-25088.93	-0.91	-25052.63	2.70	-25021.03	1.62	-24980.41	9.56	-24968.29	5.34
-25155.38	-2.00	-25120.10	0.60	-25087.17	0.85	-25053.78	1.56	-25022.14	0.51	-24986.93	3.04	-24960.50	13.13
-25155.59	-2.21	-25122.48	-1.78	-25084.53	3.49	-25046.53	8.80	-25019.96	2.69	-24988.73	1.24	-24965.21	8.42
-25156.73	-3.35	-25124.13	-3.43	-25087.57	0.44	-25056.19	-0.86	-25018.98	3.68	-24983.39	6.58	-24967.52	6.11
-25156.73	-3.36	-25121.46	-0.77	-25088.60	-0.58	-25056.04	-0.71	-25017.97	4.68	-24989.18	0.79	-24966.77	6.86
-25156.06	-2.68	-25123.20	-2.50	-25088.12	-0.11	-25053.54	1.79	-25017.34	5.31	-24988.33	1.64	-24967.88	5.75
-25156.94	-3.57	-25122.27	-1.58	-25087.26	0.75	-25054.58	0.75	-25021.29	1.36	-24984.13	5.84	-24970.88	2.75
-25156.88	-3.50	-25121.93	-1.23	-25089.58	-1.57	-25054.54	0.79	-25020.03	2.62	-24987.57	2.40	-24965.01	8.62
-25157.25	-3.87	-25123.60	-2.91	-25086.44	1.57	-25055.92	-0.58	-25013.42	9.24	-24987.57	2.40	-24970.41	3.22
-25156.29	-2.91	-25121.85	-1.15	-25088.28	-0.27	-25054.18	1.15	-25019.81	2.84	-24981.85	8.12	-24968.67	4.96
-25156.44	-3.06	-25122.25	-1.56	-25086.93	1.09	-25050.48	4.85	-25020.27	2.38	-24985.77	4.20	-24963.67	9.96
-25156.40	-3.02	-25122.22	-1.53	-25088.36	-0.35	-25052.39	2.94	-25017.90	4.75	-24982.67	7.30	-24969.27	4.36
-25156.70	-3.32	-25121.85	-1.15	-25089.50	-1.49	-25050.58	4.75	-25021.38	1.27	-24984.18	5.78	-24970.14	3.49
-25156.41	-3.03	-25121.51	-0.81	-25088.81	-0.79	-25054.60	0.73	-25017.92	4.73	-24987.73	2.24	-24965.94	7.68
-25156.80	-3.43	-25121.63	-0.93	-25090.01	-1.99	-25048.89	6.44	-25016.37	6.28	-24985.01	4.96	-24970.22	3.41
-25156.13	-2.75	-25123.13	-2.43	-25086.02	1.99	-25051.90	3.43	-25015.41	7.24	-24984.96	5.01	-24967.15	6.48
-25157.91	-4.53	-25121.95	-1.25	-25087.19	0.82	-25052.47	2.86	-25016.70	5.95	-24985.64	4.32	-24969.54	4.09
-25157.77	-4.39	-25121.32	-0.63	-25086.01	2.01	-25051.58	3.75	-25020.70	1.95	-24983.78	6.19	-24969.18	4.45
-25155.57	-2.19	-25122.32	-1.62	-25088.50	-0.48	-25053.41	1.93	-25008.92	13.73	-24988.19	1.78	-24966.03	7.59
-25154.32	-0.94	-25123.67	-2.98	-25088.02	0.00	-25054.60	0.73	-25020.70	1.95	-24987.45	2.52	-24967.92	5.71

	-25157.63	-4.25	-25122.71	-2.01	-25089.20	-1.19	-25050.70	4.63	-25021.71	0.94	-24982.67	7.30	-24963.69	9.94
	-25158.78	-5.40	-25123.04	-2.34	-25085.66	2.35	-25054.04	1.29	-25022.39	0.26	-24986.95	3.02	-24959.68	13.94
	-25157.09	-3.71	-25124.53	-3.83	-25089.55	-1.53	-25055.58	-0.25	-25021.97	0.68	-24986.18	3.79	-24969.96	3.67
	-25157.24	-3.86	-25123.06	-2.36	-25089.60	-1.59	-25054.04	1.29	-25022.05	0.60	-24982.73	7.24		
	-25157.17	-3.79	-25122.03	-1.33	-25087.66	0.36	-25052.69	2.65	-25014.08	8.57	-24983.16	6.81		
	-25157.04	-3.66	-25122.73	-2.03	-25086.66	1.35			-25019.64	3.01	-24980.15	9.82		
	-25157.68	-4.30			-25089.48	-1.46					-24986.78	3.19		
	-25155.46	-2.08												
	-25157.10	-3.72												
Average energies	-25156.82	-3.44	-25122.14	-1.44	-25087.75	0.27	-25053.32	2.02	-25019.62	3.03	-24983.76	6.21	-24967.46	6.16

Pu

	ground state energy	solid solution energy												
concentration in solid solution (%)	3		6		9		12		15		18		21	
number of atoms	2		4		6		8		10		12		13	
	-25150.15	-2.76	-25110.06	-1.34	-25070.87	-0.82	-25028.39	3.00	-24992.65	0.07	-24954.29	-0.23	-24928.78	5.94
	-25150.21	-2.82	-25110.67	-1.94	-25070.43	-0.37	-25032.93	-1.54	-24991.18	1.54	-24949.06	4.99	-24930.40	4.32
	-25149.70	-2.31	-25111.47	-2.74	-25071.60	-1.54	-25033.32	-1.93	-24994.61	-1.89	-24951.55	2.50	-24932.59	2.13
	-25152.44	-5.05	-25111.19	-2.46	-25069.83	0.23	-25029.50	1.89	-24991.49	1.23	-24955.23	-1.17	-24926.95	7.77
	-25150.17	-2.78	-25110.21	-1.48	-25073.57	-3.51	-25033.32	-1.93	-24993.35	-0.63	-24953.56	0.50	-24933.41	1.31
	-25149.43	-2.04	-25110.59	-1.86	-25073.44	-3.38	-25031.57	-0.18	-24992.18	0.55	-24949.55	4.51	-24935.42	-0.70
	-25150.61	-3.21	-25110.81	-2.09	-25071.39	-1.33	-25029.24	2.15	-24989.92	2.80	-24949.57	4.49	-24926.58	8.14
	-25151.09	-3.70	-25110.28	-1.55	-25071.75	-1.69	-25032.04	-0.65	-24993.28	-0.55	-24951.59	2.46	-24934.02	0.70
	-25150.88	-3.49	-25113.10	-4.38	-25064.43	5.63	-25034.01	-2.62	-24989.99	2.74	-24955.01	-0.95	-24931.13	3.59
	-25150.06	-2.66	-25111.37	-2.64	-25074.60	-4.54	-25032.96	-1.57	-24992.72	0.00	-24949.07	4.99	-24930.77	3.95
	-25151.02	-3.63	-25112.61	-3.88	-25069.28	0.78	-25030.96	0.43	-24992.79	-0.07	-24952.18	1.88	-24935.76	-1.04
	-25150.50	-3.10	-25111.73	-3.00	-25071.11	-1.05	-25032.08	-0.69	-24988.15	4.57	-24946.00	8.06	-24932.22	2.50
	-25152.16	-4.76	-25111.16	-2.43	-25072.22	-2.16	-25030.73	0.66	-24992.45	0.27	-24951.07	2.99	-24931.14	3.58
	-25152.82	-5.43	-25110.97	-2.24	-25070.66	-0.60	-25030.93	0.47	-24991.62	1.11	-24956.09	-2.04	-24930.63	4.09
	-25151.62	-4.23	-25110.56	-1.84	-25070.84	-0.78	-25033.33	-1.94	-24992.75	-0.03	-24951.04	3.02	-24931.12	3.60
	-25151.06	-3.67	-25111.80	-3.07	-25070.45	-0.39	-25031.74	-0.35	-24991.57	1.16	-24954.58	-0.52	-24934.55	0.17
	-25150.93	-3.53	-25111.80	-3.08	-25070.99	-0.93	-25029.89	1.50	-24991.74	0.98	-24949.19	4.86	-24932.69	2.03
	-25151.33	-3.94	-25111.03	-2.30	-25072.00	-1.94	-25032.66	-1.26	-24987.80	4.93	-24953.59	0.47	-24932.90	1.83
	-25151.72	-4.33	-25108.04	0.68	-25071.23	-1.17	-25032.40	-1.01	-24992.13	0.59	-24952.67	1.39	-24928.67	6.05
	-25151.01	-3.62	-25109.39	-0.66	-25071.37	-1.31	-25032.40	-1.01	-24993.74	-1.02	-24949.92	4.13	-24935.85	-1.13
	-25151.29	-3.90	-25111.75	-3.02	-25069.39	0.67	-25031.53	-0.14	-24993.02	-0.30	-24951.60	2.46	-24933.06	1.66
	-25151.93	-4.53	-25111.81	-3.09	-25072.10	-2.04	-25032.66	-1.27	-24992.53	0.19	-24953.01	1.04	-24931.45	3.27
	-25152.81	-5.42	-25111.54	-2.82	-25072.06	-2.00	-25032.05	-0.66	-24989.96	2.76	-24950.39	3.67	-24930.33	4.39

-25151.74	-4.35	-25111.11	-2.39	-25068.27	1.79	-25033.17	-1.78	-24990.12	2.60	-24952.01	2.05	-24925.96	8.76
-25151.01	-3.61	-25109.39	-0.66	-25069.50	0.56	-25024.69	6.70	-24990.53	2.19	-24945.26	8.80	-24928.93	5.79
-25151.03	-3.64	-25109.37	-0.64	-25070.23	-0.17	-25032.97	-1.57	-24993.28	-0.56	-24950.94	3.11	-24922.92	11.80
-25151.64	-4.25	-25112.06	-3.34	-25072.49	-2.43	-25030.56	0.83	-24989.71	3.01	-24953.33	0.73	-24934.84	-0.12
-25150.10	-2.71	-25110.59	-1.86	-25070.88	-0.82	-25032.27	-0.88	-24991.19	1.53	-24952.17	1.89	-24932.83	1.89
-25150.49	-3.10	-25107.16	1.57	-25072.92	-2.86	-25032.63	-1.24	-24987.71	5.01	-24945.46	8.60	-24925.21	9.51
-25151.76	-4.37	-25111.91	-3.18	-25070.64	-0.58	-25034.16	-2.77	-24994.47	-1.74	-24953.10	0.96	-24932.18	2.55
-25151.11	-3.72	-25110.58	-1.85	-25069.06	1.00	-25031.38	0.01	-24991.69	1.03	-24945.80	8.25	-24933.07	1.65
-25151.32	-3.93	-25112.65	-3.92	-25071.57	-1.51	-25031.96	-0.57	-24991.55	1.18	-24947.36	6.70	-24927.63	7.09
-25152.28	-4.89	-25110.54	-1.81	-25072.85	-2.79	-25029.23	2.16	-24989.18	3.54	-24952.84	1.21	-24932.18	2.54
-25150.58	-3.18	-25112.07	-3.34	-25068.96	1.10	-25031.18	0.21	-24994.08	-1.36	-24980.70	-26.64	-24930.96	3.76
-25152.33	-4.94	-25111.50	-2.78	-25073.30	-3.24	-25030.57	0.82	-24992.33	0.40	-24949.04	5.02	-24929.83	4.89
-25151.99	-4.60	-25108.90	-0.17	-25072.71	-2.65	-25033.84	-2.45	-24986.65	6.08	-24953.88	0.18	-24932.37	2.35
-25151.24	-3.85	-25111.25	-2.53	-25069.80	0.26	-25028.64	2.75	-24993.41	-0.69	-24951.46	2.60	-24934.61	0.11
-25151.32	-3.92	-25112.04	-3.32	-25067.47	2.59	-25034.54	-3.15	-24984.10	8.63	-24952.42	1.63	-24927.83	6.90
-25151.75	-4.36	-25112.39	-3.67	-25069.56	0.50	-25032.52	-1.13	-24992.91	-0.19	-24951.75	2.31	-24934.82	-0.10
-25150.80	-3.40	-25111.48	-2.75	-25067.78	2.28	-25030.90	0.49	-24990.04	2.69	-24953.57	0.48	-24933.36	1.37
-25151.72	-4.32	-25111.08	-2.35	-25070.53	-0.48	-25031.02	0.37	-24991.67	1.05	-24952.48	1.58	-24933.77	0.95
-25150.62	-3.23	-25110.98	-2.25	-25070.88	-0.83	-25031.33	0.06	-24990.83	1.90	-24954.91	-0.85	-24933.38	1.34
-25151.28	-3.88	-25111.66	-2.93	-25071.89	-1.83	-25034.26	-2.87	-24991.33	1.39	-24951.91	2.14	-24931.52	3.20
-25151.59	-4.20	-25108.37	0.36	-25070.74	-0.68	-25033.19	-1.80	-24991.85	0.87	-24953.37	0.69	-24935.47	-0.75
-25150.86	-3.46	-25109.26	-0.53	-25071.41	-1.35	-25033.18	-1.79	-24989.27	3.45	-24947.57	6.49	-24932.83	1.89
-25151.84	-4.44	-25109.00	-0.27	-25070.88	-0.82	-25030.91	0.49	-24991.69	1.04	-24947.83	6.23	-24933.91	0.81
-25152.15	-4.75	-25111.34	-2.61	-25069.58	0.48	-25032.32	-0.93	-24992.79	-0.07	-24951.71	2.35	-24936.95	-2.23
-25151.40	-4.00	-25110.54	-1.82	-25070.33	-0.27	-25032.13	-0.74	-24993.05	-0.33	-24955.13	-1.07	-24929.55	5.17
-25151.16	-3.76	-25110.71	-1.98	-25070.70	-0.64	-25031.09	0.30	-24989.91	2.82	-24948.43	5.63	-24932.94	1.78
-25151.44	-4.05	-25112.32	-3.60	-25071.24	-1.18	-25029.00	2.39	-24990.82	1.91	-24952.63	1.42	-24935.24	-0.52
-25150.87	-3.48	-25111.17	-2.44	-25072.45	-2.39	-25031.15	0.24	-24991.99	0.73	-24951.48	2.58	-24934.18	0.54
-25150.34	-2.95	-25112.29	-3.57	-25070.20	-0.14	-25032.76	-1.37	-24991.18	1.54	-24948.99	5.06	-24931.32	3.40
-25150.81	-3.42	-25110.02	-1.30	-25069.40	0.66	-25029.17	2.22	-24991.05	1.67	-24953.35	0.71	-24934.57	0.15
-25151.14	-3.74	-25110.19	-1.46	-25071.42	-1.36	-25032.41	-1.02	-24991.78	0.94	-24950.41	3.65	-24930.72	4.00
-25149.47	-2.07	-25112.35	-3.63	-25071.41	-1.35	-25031.37	0.02	-24991.38	1.34	-24955.21	-1.15	-24933.26	1.47
-25151.22	-3.82	-25106.92	1.80	-25070.30	-0.24	-25031.49	-0.09	-24993.61	-0.89	-24951.36	2.69	-24932.25	2.47
-25151.39	-4.00	-25109.40	-0.68	-25070.97	-0.91	-25030.91	0.48	-24991.85	0.88	-24954.48	-0.43	-24934.96	-0.23
-25151.06	-3.67	-25110.51	-1.78	-25070.14	-0.08	-25026.75	4.64	-24991.06	1.66	-24954.97	-0.91	-24933.11	1.62
-25150.96	-3.57	-25109.16	-0.43	-25071.90	-1.84	-25030.94	0.45	-24993.25	-0.53	-24953.39	0.66	-24933.30	1.43
-25150.47	-3.08	-25111.77	-3.04	-25071.00	-0.94	-25032.12	-0.73	-24989.39	3.34	-24955.19	-1.13	-24930.91	3.81
-25150.76	-3.36	-25111.54	-2.81	-25070.67	-0.61	-25029.60	1.80	-24992.07	0.65	-24955.41	-1.35	-24932.17	2.55
-25151.25	-3.85	-25109.34	-0.62	-25071.01	-0.95	-25031.98	-0.59	-24994.09	-1.36	-24950.68	3.37	-24932.26	2.46
-25150.45	-3.06	-25107.80	0.93	-25071.12	-1.06	-25030.32	1.08	-24989.54	3.18	-24949.61	4.44	-24926.06	8.66
-25151.28	-3.88	-25111.54	-2.81	-25068.84	1.22	-25028.78	2.61	-24992.18	0.54	-24951.31	2.74	-24932.76	1.96

-25151.64	-4.25	-25112.01	-3.28	-25070.72	-0.66	-25032.77	-1.38	-24990.85	1.88	-24950.43	3.62	-24923.88	10.84
-25152.87	-5.47	-25110.03	-1.30	-25067.56	2.50	-25032.63	-1.24	-24989.62	3.10	-24951.42	2.63	-24934.84	-0.12
-25149.88	-2.49	-25110.82	-2.09	-25071.65	-1.59	-25030.26	1.13	-24989.44	3.29	-24949.79	4.27	-24931.69	3.03
-25150.04	-2.64	-25110.95	-2.23	-25070.59	-0.53	-25033.75	-2.35	-24989.09	3.64	-24955.88	-1.83	-24935.54	-0.82
-25151.12	-3.73	-25110.85	-2.13	-25071.83	-1.77	-25031.80	-0.41	-24989.45	3.27	-24952.85	1.20	-24934.62	0.11
-25149.68	-2.28	-25111.67	-2.95	-25072.10	-2.04	-25031.05	0.35	-24991.79	0.93	-24989.39	-35.33	-24928.34	6.38
-25151.06	-3.67	-25109.33	-0.60	-25069.79	0.27	-25031.78	-0.39	-24992.85	-0.13	-24950.19	3.86	-24935.12	-0.40
-25150.26	-2.86	-25112.04	-3.31	-25073.71	-3.65	-25033.26	-1.87	-24993.41	-0.68	-24948.52	5.54	-24935.69	-0.97
-25152.02	-4.62	-25106.21	2.52	-25071.47	-1.41	-25032.70	-1.30	-24994.10	-1.37	-24951.10	2.95	-24930.85	3.87
-25151.75	-4.35	-25110.86	-2.13	-25069.07	0.98	-25032.11	-0.72	-24987.82	4.91	-24953.54	0.52	-24925.80	8.92
-25151.14	-3.75	-25112.47	-3.74	-25071.10	-1.04	-25030.25	1.14	-24988.88	3.84	-24948.36	5.70	-24933.01	1.71
-25149.13	-1.74	-25111.62	-2.89	-25071.82	-1.76	-25029.74	1.65	-24991.33	1.39	-24949.92	4.13	-24931.97	2.75
-25152.28	-4.89	-25110.70	-1.98	-25070.97	-0.91	-25030.70	0.69	-24990.19	2.54	-24950.87	3.18	-24934.93	-0.21
-25151.91	-4.51	-25110.47	-1.74	-25070.43	-0.37	-25031.24	0.15	-24984.83	7.89	-24952.34	1.72	-24934.91	-0.19
-25150.37	-2.97	-25109.12	-0.39	-25070.13	-0.07	-25031.40	-0.01	-24992.55	0.18	-24946.63	7.42	-24931.61	3.12
-25151.85	-4.45	-25112.01	-3.28	-25070.21	-0.15	-25030.28	1.11	-24993.63	-0.91	-24948.21	5.85	-24925.24	9.48
-25151.11	-3.72	-25112.07	-3.34	-25068.47	1.59	-25030.74	0.65	-24993.81	-1.09	-24951.65	2.41	-24930.93	3.79
-25151.80	-4.40	-25110.89	-2.16	-25071.81	-1.75	-25031.16	0.23	-24992.40	0.32	-24952.48	1.57	-24930.28	4.44
-25150.69	-3.30	-25110.53	-1.80	-25070.47	-0.41	-25030.52	0.87	-24994.35	-1.63	-24952.20	1.85	-24935.49	-0.77
-25151.13	-3.73	-25110.66	-1.93	-25071.29	-1.23	-25029.61	1.78	-24993.36	-0.64	-24955.49	-1.43	-24930.53	4.19
-25152.39	-5.00	-25112.95	-4.22	-25071.65	-1.59	-25028.54	2.85	-24990.92	1.81	-24948.82	5.24	-24934.55	0.17
-25151.02	-3.63	-25112.03	-3.30	-25071.20	-1.15	-25030.75	0.64	-24989.67	3.05	-24952.79	1.27	-24930.71	4.01
-25151.03	-3.63	-25110.40	-1.67	-25070.94	-0.88	-25031.33	0.06	-24989.19	3.54	-24949.87	4.19	-24931.38	3.34
-25150.73	-3.34	-25110.58	-1.85	-25070.84	-0.78	-25031.49	-0.10	-24994.10	-1.38	-24950.08	3.98	-24934.87	-0.15
-25151.11	-3.72	-25111.74	-3.01	-25071.44	-1.38	-25031.13	0.26	-24990.38	2.34	-24946.54	7.51	-24934.23	0.49
-25151.40	-4.00	-25110.63	-1.90	-25071.39	-1.33	-25028.89	2.50	-24994.33	-1.60	-24950.61	3.45	-24932.32	2.40
-25150.54	-3.15	-25110.74	-2.02	-25070.23	-0.17	-25031.94	-0.55	-24994.71	-1.99	-24948.40	5.65	-24935.19	-0.46
-25149.94	-2.55	-25111.80	-3.07	-25069.63	0.43	-25032.03	-0.64	-24992.79	-0.06	-24952.98	1.07	-24930.45	4.27
-25149.61	-2.22	-25111.17	-2.44	-25071.67	-1.61	-25027.14	4.25	-24993.36	-0.64	-24952.73	1.33	-24930.19	4.53
-25151.80	-4.40	-25109.37	-0.65	-25069.76	0.30	-25026.02	5.38	-24991.95	0.78	-24953.68	0.37	-24932.26	2.46
-25151.26	-3.87	-25112.22	-3.49	-25069.89	0.17	-25028.30	3.09	-24992.12	0.60	-24952.53	1.52	-24929.12	5.60
-25151.49	-4.10	-25111.22	-2.50	-25072.48	-2.42	-25033.42	-2.03	-24988.89	3.83	-24955.18	-1.12	-24927.37	7.35
-25151.15	-3.76	-25112.98	-4.26	-25070.25	-0.19	-25031.58	-0.19	-24990.35	2.37	-24952.22	1.84	-24932.41	2.31
-25150.78	-3.38	-25111.27	-2.54	-25071.98	-1.92	-25030.02	1.37	-24987.66	5.06	-24951.65	2.40	-24931.35	3.38
-25150.67	-3.28	-25111.31	-2.58	-25072.00	-1.94	-25029.53	1.86	-24991.49	1.24	-24949.14	4.92	-24930.11	4.62
-25150.40	-3.00	-25110.56	-1.84	-25070.90	-0.84	-25028.44	2.95	-24991.45	1.28	-24951.22	2.84	-24929.32	5.40
-25150.32	-2.92	-25110.96	-2.24	-25069.81	0.25	-25029.24	2.15	-24986.37	6.35	-24953.83	0.23	-24935.01	-0.29
-25152.09	-4.70	-25081.24	27.49	-25070.54	-0.49	-25032.54	-1.15	-24992.23	0.50	-24949.78	4.27	-24934.72	0.00
-25153.37	-5.97	-25110.28	-1.56	-25070.16	-0.10	-25032.35	-0.96	-24991.31	1.41	-24951.77	2.28	-24933.83	0.89
-25150.27	-2.88	-25111.12	-2.39	-25071.09	-1.03	-25033.59	-2.19	-24991.00	1.72	-24951.59	2.47	-24931.85	2.87
-25150.51	-3.12	-25110.84	-2.11	-25071.16	-1.10	-25031.25	0.14	-24987.77	4.95	-24954.10	-0.05	-24932.38	2.34

	-25151.31	-3.92	-25111.27	-2.54	-25071.99	-1.93	-25026.08	5.32	-24992.99	-0.26	-24950.69	3.37	-24931.13	3.60
	-25150.80	-3.41	-25111.08	-2.35	-25071.19	-1.13	-25028.83	2.56	-24991.45	1.28	-24947.64	6.42	-24934.76	-0.04
	-25151.25	-3.86	-25110.40	-1.67	-25072.98	-2.92	-25029.22	2.17	-24991.19	1.53	-24950.78	3.28	-24935.65	-0.93
	-25150.67	-3.28	-25110.52	-1.79	-25070.17	-0.11	-25032.70	-1.31	-24984.30	8.42	-24951.95	2.10	-24929.30	5.42
	-25152.00	-4.60	-25109.94	-1.21	-25070.31	-0.25	-25029.83	1.56	-24986.95	5.77	-24952.62	1.44	-24933.14	1.58
	-25150.89	-3.50	-25110.52	-1.79	-25071.37	-1.31	-25031.61	-0.21	-24991.81	0.91	-24949.35	4.70	-24929.58	5.15
	-25151.19	-3.79	-25106.77	1.95	-25070.71	-0.65	-25030.90	0.49	-24994.16	-1.43	-24950.01	4.05	-24929.71	5.01
	-25151.80	-4.41	-25109.35	-0.63	-25070.42	-0.36	-25028.16	3.23	-24991.99	0.73	-24953.74	0.32	-24930.39	4.33
	-25152.04	-4.65	-25110.65	-1.93	-25071.81	-1.75	-25032.35	-0.96	-24991.44	1.29	-24955.93	-1.87		
	-25150.27	-2.87			-25071.96	-1.90	-25028.64	2.76	-24988.82	3.90				
	-25151.01	-3.62			-25070.51	-0.45			-24990.74	1.99				
	-25150.62	-3.22			-25071.35	-1.29			-24990.52	2.20				
	-25151.14	-3.74			-25072.58	-2.52								
	-25151.59	-4.20			-25038.23	31.82								
	-25151.34	-3.94												
Average energies	-25151.12	-3.73	-25110.52	-1.79	-25070.61	-0.55	-25031.13	0.26	-24991.23	1.49	-24952.07	1.98	-24931.86	2.86

Th

	ground state energy	solid solution energy												
concentration in solid solution (%)	3		6		9		12		15		18		21	
number of atoms	2		4		6		8		10		12		13	
	-25157.3	-4.22783	-25121.9	-1.91598	-25084.9	2.036416	-25049.9	3.968899	-25019.4	1.469365	-24973.2	14.6546	-24966.3	4.998474
	-25156.4	-3.32855	-25121.8	-1.86111	-25088.6	-1.64931	-25051.9	1.989577	-25015.9	4.990805	-24978.7	9.168477	-24967.1	4.263415
	-25156.9	-3.83644	-25120.9	-0.91866	-25086.1	0.858515	-25051.6	2.267629	-25019	1.825912	-24986.6	1.202963	-24967.7	3.635914
	-25157.5	-4.45367	-25121.6	-1.57994	-25086	0.981974	-25051.8	2.157499	-25018.4	2.487137	-24983.6	4.190706	-24959.3	12.05972
	-25157.2	-4.2114	-25122.8	-2.76818	-25087.1	-0.11134	-25051.7	2.176556	-25019.3	1.5889	-24986.4	1.480186	-24970.9	0.403499
	-25155.7	-2.69488	-25124.2	-4.24962	-25083.4	3.574469	-25051.9	2.000738	-25017	3.922392	-24981.5	6.347626	-24967.3	4.022436
	-25155.9	-2.87001	-25122.3	-2.34107	-25085.5	1.437631	-25052.8	1.091401	-25015.6	5.24322	-24985.2	2.649457	-24972.1	-0.81318
	-25157.5	-4.44763	-25120.3	-0.33144	-25085.6	1.392892	-25053.3	0.579821	-25019	1.85241	-24984.6	3.274363	-24966.5	4.791846
	-25156.7	-3.70592	-25120.9	-0.86817	-25087.7	-0.71982	-25051.8	2.104597	-25014.7	6.165108	-24979.8	8.070165	-24967.2	4.075742
	-25156.9	-3.82565	-25122.8	-2.78369	-25086.7	0.238353	-25054.8	-0.8689	-25017	3.91665	-24979.6	8.208576	-24970.4	0.940209
	-25158.3	-5.23694	-25122.8	-2.84055	-25084.5	2.47048	-25055.7	-1.7764	-25018.2	2.721874	-24986.5	1.334092	-24969.2	2.074515
	-25156.8	-3.82332	-25121.4	-1.44125	-25087.8	-0.83633	-25053.2	0.675102	-25017.5	3.353479	-24976	11.78874	-24962.7	8.606322
	-25155.2	-2.20956	-25120.1	-0.12111	-25088.9	-1.96687	-25053.4	0.518042	-25020.1	0.734677	-24983	4.815441	-24964.9	6.456978
	-25156.7	-3.66938	-25122.3	-2.34383	-25086.7	0.289045	-25053.2	0.690126	-25017.9	2.940541	-24981	6.824952	-24963.9	7.44271
	-25151.9	1.120658	-25121.9	-1.87227	-25084.1	2.853524	-25055	-1.13068	-25016.4	4.437281	-24984.1	3.701935	-24969.1	2.233905
	-25157.7	-4.6583	-25122.5	-2.53372	-25084.3	2.687844	-25052.4	1.504634	-25017.5	3.415258	-24984.4	3.402879	-24953.1	18.20312
	-25156.5	-3.50187	-25122.9	-2.92983	-25085.9	1.002449	-25053.1	0.84965	-25015.9	4.990354	-24985.1	2.716564	-24967.3	3.970999

-25155.9	-2.84418	-25122.3	-2.33937	-25085.2	1.7205	-25051.2	2.71349	-25017.9	2.976325	-24982.2	5.660856	-24968	3.32377
-25157.6	-4.5566	-25119.5	0.486249	-25086.7	0.218487	-25052.8	1.151148	-25016.5	4.406892	-24973	14.80201	-24961.5	9.794532
-25157	-3.93138	-25122.1	-2.11718	-25087.7	-0.73771	-25054.2	-0.29754	-25020	0.895868	-24981.9	5.906316	-24969.8	1.531028
-25156.7	-3.71119	-25122.1	-2.11628	-25086.3	0.635206	-25048	5.954556	-25017.7	3.1418	-24986.4	1.44148	-24955.6	15.76084
-25157.3	-4.32079	-25121.1	-1.11998	-25087.6	-0.62893	-25051.3	2.604742	-25015.8	5.095732	-24979.5	8.368544	-24966.3	4.987965
-25155.9	-2.88738	-25122.8	-2.78541	-25084.2	2.763747	-25052.1	1.862087	-25016.6	4.286801	-24986.5	1.371921	-24962.9	8.460192
-25156.5	-3.51863	-25123.2	-3.21583	-25087.1	-0.17413	-25051.9	2.007535	-25014.2	6.657547	-24979	8.836582	-24967.6	3.755049
-25156.8	-3.73908	-25122.3	-2.29678	-25084.3	2.648861	-25053.1	0.7946	-25019	1.911879	-24986.1	1.78353	-24967.5	3.832213
-25154.5	-1.42541	-25121.7	-1.68705	-25088.5	-1.58901	-25052.2	1.751638	-25021.3	-0.44568	-24986.9	0.933164	-24959.6	11.75189
-25156.4	-3.40861	-25123	-2.99688	-25085.5	1.48812	-25052.2	1.688869	-25019.8	1.027994	-24981.6	6.200662	-24965.7	5.659438
-25156.9	-3.90644	-25121.6	-1.63805	-25089	-2.01415	-25055.3	-1.40655	-25018.3	2.600296	-24984	3.812043	-24971.6	-0.26414
-25155	-1.98372	-25122.2	-2.18317	-25088.3	-1.39171	-25053.5	0.445448	-25018.9	2.017167	-24985	2.84397	-24966.5	4.826989
-25155.3	-2.29858	-25122.2	-2.25234	-25085.2	1.792285	-25053.5	0.382725	-25019.5	1.377535	-24979.2	8.645995	-24944.4	26.95957
-25156.2	-3.17769	-25122.9	-2.91198	-25087.6	-0.64722	-25048.6	5.337665	-25018.7	2.145851	-24982.2	5.628349	-24964.7	6.633225
-25154.9	-1.87544	-25121.9	-1.88239	-25080.8	6.140936	-25052.7	1.207853	-25013.9	6.989447	-24982.9	4.936595	-24963.9	7.43119
-25157	-4.02006	-25120.7	-0.66985	-25085.8	1.162261	-25053.8	0.151813	-25018.7	2.132504	-24980	7.820258	-24968.4	2.930696
-25157.8	-4.79704	-25120.3	-0.32729	-25088.2	-1.28115	-25049.8	4.116821	-25015.9	4.994846	-24980	7.856801	-24959.9	11.40193
-25156.6	-3.58359	-25118.4	1.557224	-25086	0.952445	-25052.7	1.185165	-25017.7	3.126307	-24983.5	4.351389	-24969.1	2.237759
-25156.9	-3.92374	-25122.9	-2.89226	-25087	-0.07488	-25050.3	3.577497	-25018.6	2.320767	-24985.1	2.70912	-24966	5.299509
-25156.3	-3.23869	-25121.5	-1.46751	-25089.1	-2.11297	-25054.6	-0.68903	-25016	4.859426	-24985.7	2.102266	-24964.7	6.646546
-25157.9	-4.85412	-25119.8	0.178328	-25083.5	3.404173	-25046	7.869459	-25015.8	5.083997	-24975.9	11.96991	-24966.5	4.789419
-25156.6	-3.62291	-25123.8	-3.80821	-25084	2.907162	-25054.7	-0.75254	-25020	0.8985	-24982.4	5.474145	-24970.3	1.002737
-25157.5	-4.48611	-25122.7	-2.72448	-25085.6	1.386395	-25051.8	2.129146	-25016.1	4.824911	-24981.3	6.524471	-24966.9	4.431011
-25155.6	-2.54711	-25122.6	-2.63642	-25088.9	-1.90136	-25049.5	4.456694	-25014.7	6.196374	-24987.3	0.586628	-6.6E+11	-6.6E+11
-25156.7	-3.66713	-25123.1	-3.12987	-25088.8	-1.89386	-25043.3	10.5931	-25009.1	11.78021	-24977.5	10.36626	-24971.1	0.178396
-25155.8	-2.81503	-25123.8	-3.77097	-25086.7	0.292491	-25043.9	10.05116	-25016.1	4.785258	-24982.2	5.649306	-24958.6	12.69268
-25155.9	-2.83467	-25122.8	-2.80528	-25085.5	1.400892	-25053	0.92428	-25015.1	5.76826	-24983	4.838617	-24960.1	11.25682
-25156.7	-3.69714	-25120.6	-0.65349	-25087.9	-0.9668	-25053.9	0.043885	-25020.5	0.32583	-24984.1	3.702093	-24966.1	5.20651
-25157.2	-4.21235	-25122.8	-2.80095	-25082.8	4.178718	-25050.6	3.30063	-25012.3	8.594429	-24983	4.848694	-24971.5	-0.1593
-25158.6	-5.55611	-25122.5	-2.50372	-25087.2	-0.27956	-25052.5	1.460991	-25013.5	7.371191	-24980.4	7.413365	-24960.2	11.11781
-25156.7	-3.69551	-25122.2	-2.16555	-25087.1	-0.17477	-25049.3	4.641413	-25016.6	4.272185	-24984.6	3.205544	-24964.4	6.923437
-25156.5	-3.43567	-25122.1	-2.08995	-25086.3	0.669684	-25051.4	2.471286	-25018.1	2.80209	-24975.5	12.37003	-24969.1	2.248727
-25155.6	-2.53164	-25121.8	-1.82369	-25088.9	-1.95812	-25051.1	2.782749	-25012.9	7.945497	-24984.9	2.967718	-24968.3	2.986785
-25156.9	-3.83191	-25121.1	-1.06876	-25088.3	-1.35769	-25053.3	0.647391	-25017.2	3.64949	-24981.6	6.223857	-24965.1	6.258832
-25158.4	-5.33515	-25121.5	-1.55574	-25085.8	1.20003	-25053.2	0.704857	-25018.9	1.955547	-24983.3	4.509695	-24967.5	3.777233
-25156.2	-3.14138	-25122.1	-2.13352	-25081.9	5.008564	-25052.1	1.801621	-25016	4.883451	-24980.3	7.539783	-24963.9	7.469919
-25157.2	-4.14751	-25120.6	-0.62915	-25087.1	-0.15446	-25051.8	2.139943	-25014.2	6.646174	-24985.7	2.109531	-24963.5	7.849784
-25157.2	-4.20695	-25121.2	-1.17313	-25085.8	1.114742	-25052.3	1.656351	-25018.6	2.250566	-24984.5	3.300378	-24966.6	4.7554
-25156.6	-3.54087	-25119.2	0.805587	-25088.2	-1.27716	-25051.7	2.163619	-25015.5	5.39618	-24984.8	3.07547	-24969.4	1.87465
-25155.6	-2.57359	-25122	-2.05047	-25086.9	0.055112	-25056.8	-2.8396	-25009.2	11.70729	-24982.7	5.107353	-24962.2	9.10785
-25154.9	-1.8467	-25122.7	-2.73755	-25086.4	0.545495	-25050.1	3.843847	-25014.6	6.250913	-24981.1	6.717173	-24967.9	3.428059

-25156.1	-3.07168	-25122.4	-2.3786	-25082	4.91884	-25054.4	-0.44144	-25017.7	3.133295	-24982.9	4.891817	-24964.7	6.581982
-25155.4	-2.39491	-25123.4	-3.37508	-25086.7	0.233938	-25052.6	1.275887	-25013.4	7.430785	-24980.4	7.391847	-24961.6	9.728458
-25158.1	-5.06639	-25122.1	-2.11122	-25088.1	-1.19312	-25051.8	2.104353	-25018.7	2.17766	-24980	7.85139	-24961.4	9.901603
-25156.9	-3.92063	-25123.2	-3.23613	-25088.3	-1.36528	-25049.8	4.119491	-25019.9	0.929361	-24984.5	3.308638	-24970.1	1.216334
-25156.2	-3.19811	-25121.1	-1.12409	-25087.4	-0.40743	-25054.3	-0.37917	-25017.9	3.021443	-24985.1	2.78375	-24962.5	8.791384
-25157.4	-4.38111	-25124.2	-4.22225	-25084.7	2.284006	-25054.3	-0.34595	-25015.8	5.113971	-24977.9	9.908107	-24962.8	8.519147
-25155.6	-2.60281	-25123.6	-3.62613	-25086.1	0.820908	-25049.6	4.3466	-25019.9	0.936985	-24984.1	3.747507	-24967.5	3.780072
-25156.2	-3.14739	-25122.7	-2.68871	-25087.5	-0.55839	-25054	-0.06469	-25019.9	0.946022	-24987.3	0.55016	-24962.6	8.690989
-25156.5	-3.46084	-25122.4	-2.41458	-25084.3	2.609837	-25050.9	2.998059	-25021.4	-0.52649	-24981.9	5.888637	-24966.6	4.740087
-25156.4	-3.3577	-25122.4	-2.38202	-25090	-3.06749	-25053	0.891269	-25016.4	4.476638	-24978.1	9.694602	-24967.3	3.994982
-25155.7	-2.66561	-25121.8	-1.78547	-25086.3	0.606387	-25045.3	8.648925	-25016.3	4.537507	-24981.6	6.219801	-24958.9	12.38745
-25156.3	-3.27209	-25120.2	-0.20687	-25086.8	0.195486	-25049.5	4.391228	-25019.6	1.305168	-24980.9	6.921759	-24966.6	4.676275
-25154.8	-1.7752	-25122.6	-2.6583	-25087.4	-0.45094	-25052.4	1.476163	-25013.2	7.684949	-24986.2	1.659062	-24967.8	3.554543
-25156.6	-3.53988	-25122.1	-2.11487	-25084.7	2.238707	-25054.2	-0.33321	-25011.9	8.938111	-24984.7	3.174074	-24965.5	5.828108
-25156	-2.96105	-25120.4	-0.46233	-25084.8	2.160226	-25051.5	2.376615	-25016.1	4.824709	-24982.7	5.14349	-24967	4.313585
-25156.5	-3.44267	-25123.9	-3.89967	-25086.5	0.444957	-25053.7	0.218434	-25018.9	2.00545	-24981.5	6.379728	-24967.7	3.602818
-25155.8	-2.76765	-25120.5	-0.50838	-25087	-0.02057	-25050.4	3.553068	-25014.6	6.231993	-24980.3	7.526482	-24965.4	5.926646
-25156.6	-3.56839	-25122	-2.05173	-25088.2	-1.28385	-25042.7	11.25552	-25017.1	3.741782	-24982.2	5.627013	-24965.1	6.269946
-25156.6	-3.58218	-25121.7	-1.66257	-25087.9	-0.96515	-25055.1	-1.15521	-25017.6	3.309849	-24982.7	5.153708	-24963.6	7.753819
-25158.2	-5.19566	-25121	-1.02743	-25086.3	0.647173	-25055.1	-1.13721	-25019.9	0.926244	-24984.6	3.282254	-24966	5.356712
-25157.5	-4.50258	-25122	-1.99762	-25087.7	-0.76591	-25050.7	3.170464	-25015.2	5.688645	-24980	7.855067	-24967.3	4.013199
-25156.9	-3.89463	-25118.7	1.24925	-25088.2	-1.25163	-25049.5	4.458488	-25017.7	3.177061	-24978.7	9.188557	-24961.2	10.10021
-25156.4	-3.40623	-25122.3	-2.30378	-25086.7	0.221265	-25053.2	0.668356	-25017.8	3.097638	-24985.6	2.18995	-24958	13.35514
-25157	-3.96574	-25123	-3.03438	-25087.9	-0.94973	-25050.5	3.419188	-25017.1	3.747185	-24987.5	0.306533	-24965.3	6.004404
-25157.5	-4.4911	-25120.3	-0.34725	-25086.7	0.222404	-25055.2	-1.24652	-25018.4	2.432207	-24984.3	3.569334	-24962.2	9.151911
-25156.8	-3.78221	-25122.4	-2.45625	-25089	-2.0815	-25054.3	-0.36809	-25016.1	4.763764	-24980.4	7.488335	-24971.8	-0.46212
-25158.4	-5.40061	-25122.3	-2.32273	-25087	-0.00917	-25053.4	0.553352	-25020	0.85792	-24984.5	3.341269	-24964.2	7.152485
-25155.5	-2.51245	-25117.9	2.117402	-25085.5	1.432907	-25048.7	5.241964	-25017.3	3.578247	-24984.8	3.06044	-24965.1	6.18075
-25157.2	-4.13611	-25118	1.940947	-25085.1	1.807553	-25053.3	0.594325	-25016.7	4.213305	-24982.7	5.10209	-24966.3	5.004572
-25156.3	-3.32301	-25122.2	-2.18413	-25089	-2.00187	-25052.4	1.501292	-25019.5	1.401511	-24981.7	6.169616	-24969.5	1.825146
-25156.6	-3.6101	-25122.3	-2.2965	-25088.5	-1.51659	-25054.3	-0.34496	-25016.2	4.643397	-24980.8	7.079006	-24968.4	2.897131
-25157.8	-4.80988	-25122.1	-2.14986	-25086.2	0.798985	-25056.1	-2.22877	-25017	3.857064	-24981.7	6.146286	-24962	9.356307
-25157.7	-4.6478	-25121.6	-1.6087	-25087.5	-0.58125	-25051.9	1.967464	-25016.9	3.984396	-24988.9	-1.06459	-24965.2	6.150472
-25156.4	-3.38151	-25119	0.996379	-25088.4	-1.40455	-25054.5	-0.56329	-25018.8	2.108427	-24985.5	2.322789	-24968.9	2.435823
-25157.2	-4.15984	-25120.9	-0.88776	-25086.1	0.828313	-25050.7	3.220432	-25014.5	6.418344	-24983.6	4.230414	-24967.5	3.848243
-25156.7	-3.72073	-25120.1	-0.07264	-25089.2	-2.25671	-25049.8	4.102777	-25021	-0.10857	-24985.2	2.604383	-24965	6.347953
-25157.3	-4.30267	-25121.5	-1.50254	-25084.5	2.403703	-25051.3	2.587328	-25016.9	3.985204	-24978	9.874724	-24964.2	7.159382
-25157.4	-4.34898	-25119.8	0.212442	-25088.9	-1.96326	-25054	-0.05538	-25020.5	0.3552	-24984.5	3.347339	-24964.3	7.049859
-25154.7	-1.69678	-25123.2	-3.25753	-25086.3	0.610729	-25054.1	-0.19609	-25019.3	1.587104	-24984.6	3.210101	-24969.5	1.804304
-25156.7	-3.72114	-25121.2	-1.24597	-25089.8	-2.87403	-25046	7.912437	-25017.3	3.605859	-24984.2	3.632768	-24967	4.290639
-25156.4	-3.4171	-25122.8	-2.77422	-25086	0.99954	-25051	2.949057	-25017.5	3.375641	-24982.2	5.617689	-24963.5	7.775378

	-25156	-3.00358	-25119.9	0.047223	-25089.5	-2.51746	-25052.4	1.527637	-25018.5	2.330408	-24983	4.838492	-24964.8	6.498486
	-25155.3	-2.28865	-25123.7	-3.71553	-25086.7	0.216113	-25052.7	1.199221	-25019.8	1.049166	-24980.3	7.528513	-24964.8	6.47429
	-25155.4	-2.36992	-25121.3	-1.28097	-25085	1.921368	-25052.5	1.431121	-25020.3	0.585298	-25021	-33.1273	-24967.9	3.380964
	-25155.7	-2.67107	-25121.1	-1.12397	-25082.2	4.714212	-25053.9	0.030778	-25013.5	7.399193	-24982.2	5.618964	-24964.6	6.676832
	-25157.3	-4.2517	-25123.1	-3.14459	-25086.6	0.30948	-25050.2	3.742397	-25021.1	-0.20504	-24987.4	0.40791	-24966.8	4.523617
	-25156.3	-3.2379	-25122.2	-2.26198	-25086.8	0.124241	-25051.8	2.095462	-25014.6	6.252262	-24981.7	6.105115	-24967	4.338364
	-25157.3	-4.32358	-25122.5	-2.52561	-25087.2	-0.23121	-25049.3	4.606138	-25019.5	1.35222	-24979.5	8.304921	-24967.9	3.403688
	-25157	-3.92896	-25122.2	-2.16965	-25086.1	0.871393	-25054.5	-0.53836	-25013.1	7.790662	-24985.2	2.621524	-24969.6	1.674247
	-25156.7	-3.66401	-25121.9	-1.87065	-25083.4	3.547654	-25050.1	3.859117	-25017.7	3.143494	-24981.1	6.756729	-24967.2	4.09747
	-25156.4	-3.36815	-25122.2	-2.1811	-25086	0.985314	-25051.6	2.285129	-25014.8	6.04016	-24985.1	2.708596	-24965.8	5.489214
	-25157	-3.97799	-25121	-1.05411	-25087.2	-0.23381	-25052.3	1.607034	-25012.7	8.133388	-24978.3	9.57116	-24965.4	5.906737
	-25155.9	-2.86629	-25122.8	-2.85791	-25087.7	-0.70138	-25055.2	-1.32722	-25013.9	7.007806	-24985.2	2.681515	-24964.7	6.63909
	-25156.8	-3.74067	-25120.5	-0.5339	-25088.3	-1.35718	-25051.9	2.00733	-25017.4	3.427568	-24984.7	3.106652	-24966.8	4.475444
	-25156.3	-3.22995	-25123.3	-3.36016	-25087.6	-0.6789	-25050.6	3.263033	-25014.2	6.648479	-24981.5	6.36927	-24964	7.300746
	-25157.4	-4.42377	-25122.1	-2.06823	-25088.1	-1.13713	-25055.1	-1.15993	-25017.8	3.116389	-24974.3	13.51187	-24965.2	6.14548
	-25157.9	-4.84886	-25121.2	-1.18931	-25087.4	-0.42372			-25017.5	3.420312			-24965.3	6.052817
	-25157.1	-4.08006			-25088.2	-1.27992			-25016	4.850855				
	-25153.1	-0.08932							-25019.1	1.774605				
	-25156.3	-3.29708												
	-25158	-4.97081												
	-25155.6	-2.58249												
Average energies	-25156.56	-3.53	-25121.80	-1.81	-25086.59	0.36	-25052.01	1.90	-25017.10	3.77	-24982.82	5.02	-24965.46	5.86

U

	ground state energy	solid solution energy												
concentration in solid solution (%)	3		6		9		12		15		18		21	
number of atoms	2		4		6		8		10		12		13	
	-25160.98	-2.93	-25131.46	-1.43	-25102.30	-0.30	-25071.50	2.49	-25042.88	3.09	-25015.69	2.26	-24998.92	5.03
	-25160.62	-2.58	-25130.59	-0.57	-25103.45	-1.45	-25074.24	-0.25	-25044.61	1.36	-25009.38	8.57	-24995.91	8.03
	-25153.70	4.34	-25125.38	4.65	-25102.22	-0.22	-25073.27	0.71	-25044.63	1.33	-25017.11	0.84	-24999.25	4.69
	-25161.67	-3.63	-25132.71	-2.69	-25103.68	-1.68	-25064.01	9.98	-25041.98	3.98	-25015.46	2.49	-24999.78	4.16
	-25162.24	-4.20	-25129.12	0.91	-25092.29	9.71	-25072.86	1.13	-25038.47	7.50	-25016.51	1.44	-25000.82	3.12
	-25161.65	-3.61	-25132.92	-2.89	-25100.56	1.45	-25066.71	7.28	-25044.50	1.47	-25011.28	6.67	-25002.29	1.65
	-25159.63	-1.59	-25132.02	-1.99	-25101.25	0.76	-25072.39	1.60	-25046.61	-0.64	-25015.24	2.71	-24998.06	5.88
	-25159.47	-1.43	-25132.39	-2.37	-25100.36	1.64	-25070.62	3.37	-25039.84	6.13	-25013.80	4.15	-25000.62	3.32
	-25158.94	-0.90	-25130.84	-0.81	-25102.17	-0.16	-25070.87	3.11	-25042.82	3.15	-25016.28	1.67	-25003.86	0.09
	-25159.18	-1.13	-25130.07	-0.04	-25100.15	1.86	-25069.31	4.68	-25040.78	5.19	-25017.03	0.92	-24998.76	5.19
	-25160.46	-2.42	-25132.47	-2.44	-25102.16	-0.15	-25072.97	1.02	-25043.03	2.94	-25008.31	9.64	-25003.44	0.50

-25159.97	-1.93	-25129.38	0.64	-25099.76	2.25	-25069.75	4.24	-25041.25	4.72	-25013.69	4.26	-24994.51	9.43
-25160.20	-2.16	-25131.93	-1.90	-25101.98	0.03	-25073.19	0.80	-25044.63	1.34	-25011.09	6.86	-24994.58	9.36
-25161.24	-3.20	-25132.43	-2.41	-25100.18	1.83	-25070.51	3.48	-25042.99	2.98	-25011.87	6.08	-25000.55	3.39
-25160.51	-2.47	-25130.78	-0.76	-25100.32	1.69	-25073.93	0.05	-25043.00	2.96	-25006.44	11.51	-24998.73	5.21
-25161.64	-3.60	-25131.83	-1.81	-25102.31	-0.30	-25069.92	4.07	-25043.63	2.34	-25003.67	14.28	-25000.84	3.11
-25160.77	-2.72	-25132.38	-2.35	-25097.58	4.42	-25070.70	3.29	-25043.18	2.79	-25014.73	3.22	-25001.27	2.67
-25160.01	-1.97	-25131.73	-1.71	-25101.95	0.06	-25072.86	1.13	-25042.34	3.63	-25013.17	4.79	-25001.42	2.52
-25160.05	-2.01	-25131.56	-1.53	-25099.90	2.10	-25072.42	1.56	-25042.30	3.67	-25013.28	4.67	-24997.06	6.88
-25161.16	-3.12	-25130.48	-0.46	-25099.76	2.24	-25072.16	1.83	-25041.43	4.54	-25011.69	6.26	-24999.75	4.19
-25160.98	-2.94	-25130.79	-0.77	-25101.73	0.28	-25073.01	0.98	-25043.93	2.04	-25016.26	1.70	-25001.66	2.28
-25161.10	-3.05	-25131.01	-0.99	-25102.59	-0.59	-25075.26	-1.28	-25045.11	0.86	-25016.91	1.04	-24997.83	6.12
-25158.63	-0.58	-25131.42	-1.40	-25103.95	-1.95	-25072.78	1.20	-25045.71	0.26	-25013.60	4.35	-24995.15	8.79
-25158.92	-0.88	-25132.26	-2.24	-25101.08	0.92	-25068.69	5.30	-25045.55	0.42	-25011.50	6.45	-25001.14	2.80
-25161.00	-2.96	-25129.95	0.07	-25100.44	1.57	-25070.11	3.87	-25044.05	1.92	-25014.56	3.39	-24995.35	8.60
-25161.74	-3.69	-25131.54	-1.51	-25094.61	7.39	-25071.49	2.50	-25044.18	1.79	-25015.75	2.20	-24992.66	11.29
-25161.43	-3.39	-25132.07	-2.05	-25103.15	-1.14	-25074.35	-0.36	-25041.04	4.93	-25012.14	5.81	-24998.04	5.90
-25160.70	-2.66	-25131.98	-1.96	-25096.67	5.33	-25068.09	5.90	-25044.78	1.19	-25012.97	4.98	-24999.33	4.62
-25160.90	-2.85	-25132.52	-2.50	-25103.72	-1.71	-25076.24	-2.25	-25042.61	3.36	-25013.65	4.30	-24998.21	5.73
-25160.97	-2.93	-25132.57	-2.54	-25100.50	1.51	-25073.56	0.42	-25042.36	3.61	-25014.77	3.18	-24997.55	6.39
-25160.64	-2.60	-25131.01	-0.98	-25103.14	-1.13	-25072.06	1.93	-25038.60	7.37	-25011.30	6.65	-25002.84	1.10
-25161.44	-3.40	-25132.26	-2.23	-25099.67	2.34	-25070.71	3.28	-25045.65	0.32	-25015.45	2.51	-24997.92	6.02
-25161.23	-3.18	-25132.96	-2.94	-25097.51	4.50	-25073.04	0.95	-25046.24	-0.27	-25014.83	3.12	-25000.35	3.60
-25159.79	-1.75	-25130.61	-0.58	-25100.36	1.64	-25073.78	0.21	-25046.75	-0.78	-25012.40	5.55	-24999.08	4.86
-25160.65	-2.60	-25131.85	-1.83	-25100.83	1.18	-25074.35	-0.36	-25025.85	20.12	-25011.72	6.23	-24996.87	7.07
-25161.05	-3.00	-25129.88	0.15	-25102.69	-0.68	-25073.93	0.06	-25043.99	1.98	-25012.28	5.67	-24995.74	8.20
-25160.73	-2.69	-25130.83	-0.80	-25100.87	1.13	-25071.03	2.96	-25042.54	3.42	-25012.61	5.34	-25000.86	3.09
-25160.47	-2.43	-25131.67	-1.65	-25100.86	1.14	-25068.80	5.19	-25040.87	5.10	-25017.25	0.70	-24996.13	7.81
-25160.84	-2.79	-25130.90	-0.88	-25099.70	2.31	-25067.89	6.10	-25042.53	3.44	-25014.43	3.52	-24996.12	7.83
-25161.46	-3.42	-25130.82	-0.79	-25102.89	-0.88	-25070.82	3.16	-25041.67	4.30	-25009.33	8.62	-24997.59	6.36
-25159.19	-1.15	-25131.14	-1.11	-25103.22	-1.22	-25069.62	4.36	-25044.64	1.33	-25016.72	1.23	-25002.70	1.24
-25160.59	-2.55	-25132.94	-2.91	-25103.76	-1.76	-25072.91	1.08	-25035.36	10.61	-25012.27	5.68	-25000.22	3.72
-25159.50	-1.46	-25131.00	-0.97	-25104.60	-2.60	-25072.79	1.20	-25035.01	10.96	-25016.63	1.33	-25001.88	2.06
-25159.93	-1.89	-25124.19	5.83	-25102.57	-0.56	-25070.55	3.44	-25043.87	2.10	-25010.03	7.92	-25002.73	1.21
-25160.53	-2.49	-25132.46	-2.44	-25101.58	0.42	-25073.00	0.99	-25039.90	6.07	-25012.05	5.90	-24999.91	4.04
-25160.12	-2.08	-25130.24	-0.21	-25103.14	-1.14	-25074.79	-0.80	-25041.16	4.81	-25015.27	2.68	-24996.42	7.52
-25159.98	-1.93	-25132.15	-2.13	-25103.02	-1.02	-25075.90	-1.91	-25038.10	7.87	-25014.82	3.13	-24994.73	9.21
-25151.65	6.40	-25128.52	1.50	-25101.39	0.61	-25071.27	2.72	-25043.33	2.64	-25012.61	5.34	-24999.04	4.91
-25161.58	-3.54	-25133.04	-3.02	-25103.02	-1.01	-25072.50	1.48	-25041.27	4.70	-25015.26	2.69	-24997.05	6.89
-25159.64	-1.60	-25131.89	-1.87	-25100.67	1.34	-25073.71	0.28	-25048.28	-2.31	-25014.49	3.46	-24997.53	6.41
-25156.28	1.76	-25132.64	-2.61	-25103.39	-1.39	-25065.76	8.23	-25043.63	2.34	-25005.66	12.29	-24998.41	5.54
-25160.47	-2.43	-25131.25	-1.23	-25103.19	-1.19	-25071.91	2.08	-25042.69	3.28	-25010.68	7.28	-25000.38	3.57

	-25158.18	-0.14	-25132.19	-2.16	-25102.36	-0.36	-25070.28	3.70	-25046.10	-0.13	-25010.42	7.53	-25001.60	2.34
	-25157.80	0.25	-25131.23	-1.20	-25103.54	-1.53	-25073.54	0.45	-25043.40	2.57	-25016.40	1.56	-24992.02	11.92
	-25161.45	-3.41	-25129.58	0.44	-25101.31	0.70	-25073.06	0.93	-25043.24	2.73	-25014.36	3.59	-25000.00	3.95
	-25160.45	-2.41	-25133.02	-3.00	-25098.49	3.51	-25067.70	6.28	-25045.79	0.18	-25011.62	6.33	-25003.85	0.09
	-25159.17	-1.12	-25130.02	0.00	-25100.31	1.70	-25070.48	3.51	-25040.33	5.64	-25012.30	5.65	-24999.03	4.91
	-25158.41	-0.37	-25129.58	0.45			-25074.81	-0.82	-25041.01	4.96	-25016.37	1.58	-25001.20	2.74
	-25160.36	-2.32	-25129.15	0.88			-25070.66	3.32	-25043.16	2.81	-25012.27	5.68	-24999.24	4.71
	-25161.57	-3.53	-25130.94	-0.92			-25069.83	4.16	-25038.73	7.24	-25005.07	12.88	-24999.89	4.05
	-25161.16	-3.12	-25127.75	2.28			-25074.32	-0.34	-25046.10	-0.13	-25012.35	5.60	-24998.25	5.69
	-25161.09	-3.05	-25131.80	-1.78			-25073.58	0.41	-25043.88	2.09	-25014.07	3.88	-25000.78	3.17
	-25161.09	-3.05					-25070.60	3.39	-25037.92	8.05	-25011.99	5.96	-25000.85	3.09
	-25159.53	-1.48					-25069.50	4.49	-25034.98	10.99			-24994.93	9.02
	-25159.67	-1.63					-25072.79	1.20	-25038.51	7.46				
	-25160.97	-2.92					-25074.17	-0.18	-25042.88	3.09				
	-25154.69	3.35					-25070.02	3.97						
	-25161.37	-3.33					-25070.85	3.13						
	-25154.32	3.72					-25072.54	1.44						
	-25152.64	5.40												
	-25159.98	-1.94												
	-25160.42	-2.38												
	-25161.35	-3.31												
	-25160.19	-2.14												
Average energies	-25159.92	-1.88	-25131.07	-1.04	-25101.24	0.76	-25071.70	2.28	-25042.31	3.66	-25013.07	4.89	-24998.96	4.98

$Ca_{1-x}M_xZrTi_{2-2x}Al_{2x}O_7$ M=(Ce, Pu, Th, U)

Ti(1)Ti(3)

N.B. All stated energies are in eV

Ce

	ground state energy	solid solution energy													
concentration in solid solution (%)	3		6		9		12		15		18		21	
number of atoms	2		4		6		8		10		12		13	
	-25154.13	-0.75	-25118.38	2.32	-25078.07	9.94	-25042.25	13.08	-25003.97	18.68	-24966.98	22.99	-24930.59	43.04
	-25154.64	-1.26	-25116.81	3.89	-25073.58	14.43	-25041.19	14.14	-25005.75	16.91	-24969.05	20.92	-24933.07	40.55
	-25153.42	-0.04	-25116.41	4.28	-25080.04	7.97	-25043.71	11.62	-25004.38	18.28	-24968.24	21.73	-24932.27	41.36
	-25153.05	0.33	-25116.39	4.31	-25077.56	10.46	-25042.18	13.16	-25006.19	16.47	-24964.88	25.09	-24931.37	42.26
	-25153.23	0.15	-25116.24	4.46	-25079.08	8.94	-25043.18	12.15	-25004.10	18.55	-24968.93	21.04	-24933.21	40.42
	-25154.41	-1.03	-25117.41	3.29	-25080.11	7.90	-25042.38	12.95	-25005.27	17.38	-24968.29	21.68	-24924.83	48.80
	-25154.50	-1.12	-25116.54	4.16	-25077.53	10.49	-25043.57	11.77	-25008.73	13.92	-24965.79	24.18	-24925.72	47.91
	-25153.72	-0.34	-25118.08	2.62	-25078.49	9.53	-25036.24	19.09	-25005.38	17.28	-24968.62	21.35	-24933.60	40.03
	-25154.18	-0.80	-25116.82	3.88	-25078.29	9.73	-25040.93	14.40	-25000.35	22.30	-24968.31	21.66	-24927.95	45.68
	-25153.74	-0.36	-25118.97	1.72	-25079.79	8.23	-25040.60	14.73	-25006.43	16.22	-24966.44	23.53	-24931.12	42.51
	-25154.06	-0.68	-25116.68	4.01	-25079.65	8.37	-25042.10	13.23	-25006.55	16.11	-24969.98	19.98	-24929.86	43.77
	-25154.04	-0.66	-25116.26	4.44	-25077.31	10.70	-25041.07	14.27	-24998.35	24.31	-24969.69	20.28	-24929.80	43.83
	-25153.24	0.14	-25116.62	4.08	-25080.17	7.85	-25040.30	15.03	-25006.69	15.96	-24964.39	25.58	-24934.14	39.49
	-25154.87	-1.49	-25115.05	5.64	-25079.81	8.20	-25043.92	11.41	-25005.21	17.44	-24963.62	26.35	-24931.15	42.48
	-25154.09	-0.71	-25115.71	4.99	-25077.46	10.56	-25040.79	14.54	-25007.20	15.45	-24968.47	21.50	-24930.19	43.44
	-25153.89	-0.51	-25117.99	2.71	-25079.92	8.09	-25043.01	12.33	-25003.46	19.19	-24970.36	19.61	-24931.21	42.42
	-25154.32	-0.95	-25116.05	4.65	-25078.79	9.22	-25043.77	11.56	-25000.31	22.34	-24966.24	23.73	-24930.19	43.43
	-25151.80	1.58	-25115.57	5.12	-25080.24	7.78	-25041.50	13.83	-25003.06	19.59	-24966.92	23.05	-24931.45	42.18
	-25153.72	-0.34	-25117.32	3.38	-25080.64	7.38	-25040.11	15.22	-25001.94	20.71	-24966.75	23.22	-24933.25	40.38
	-25153.54	-0.16	-25116.31	4.39	-25081.41	6.60	-25042.57	12.76	-25001.94	20.72	-24968.21	21.76	-24928.83	44.80
	-25153.82	-0.44	-25117.74	2.95	-25079.35	8.67	-25041.51	13.82	-25005.77	16.88	-24966.06	23.91	-24931.01	42.62
	-25152.36	1.02	-25116.58	4.12	-25081.11	6.91	-25042.41	12.92	-25004.17	18.48	-24968.90	21.07	-24933.87	39.76
	-25153.94	-0.56	-25118.33	2.37	-25081.11	6.90	-25041.96	13.37	-25005.32	17.33	-24969.63	20.34	-24928.44	45.18
	-25151.80	1.58	-25117.40	3.30	-25079.11	8.91	-25041.30	14.03	-25004.82	17.83	-24969.18	20.79	-24934.14	39.49
	-25153.73	-0.35	-25116.92	3.78	-25077.39	10.62	-25044.71	10.62	-25004.61	18.04	-24965.02	24.95	-24932.88	40.75
	-25154.40	-1.02	-25118.42	2.28	-25080.48	7.53	-25040.29	15.04	-25001.70	20.95	-24963.31	26.66	-24933.76	39.87
	-25154.12	-0.74	-25116.78	3.91	-25078.33	9.69	-25041.31	14.03	-25006.47	16.18	-24961.32	28.65	-24930.69	42.94
	-25153.46	-0.08	-25117.45	3.24	-25080.35	7.66	-25043.91	11.42	-25005.58	17.08	-24967.59	22.38	-24934.80	38.83
	-25154.68	-1.30	-25117.21	3.49	-25079.71	8.30	-25040.68	14.66	-25006.15	16.50	-24969.21	20.76	-24931.78	41.85

-25154.20	-0.82	-25117.23	3.47	-25078.06	9.96	-25043.42	11.92	-25006.39	16.26	-24963.37	26.60	-24927.23	46.39
-25154.06	-0.68	-25116.81	3.89	-25078.29	9.73	-25043.39	11.94	-25008.41	14.24	-24972.04	17.93	-24934.15	39.48
-25154.04	-0.66	-25117.19	3.51	-25079.38	8.64	-25043.95	11.38	-25001.78	20.87	-24966.97	23.00	-24928.31	45.32
-25152.60	0.78	-25116.40	4.30	-25080.66	7.36	-25045.06	10.28	-25005.90	16.75	-24968.95	21.02	-24928.44	45.19
-25153.66	-0.28	-25117.17	3.53	-25078.79	9.23	-25041.39	13.94	-25006.78	15.87	-24968.89	21.08	-24932.41	41.22
-25154.55	-1.17	-25118.41	2.29	-25077.70	10.32	-25044.29	11.05	-25006.04	16.61	-25008.58	-18.61	-24932.01	41.62
-25153.86	-0.48	-25117.11	3.59	-25079.39	8.62	-25040.50	14.83	-25006.03	16.63	-24964.52	25.45	-24931.15	42.48
-25154.70	-1.32	-25117.12	3.57	-25081.56	6.45	-25043.29	12.04	-25005.27	17.38	-24970.27	19.70	-24931.76	41.87
-25153.33	0.05	-25116.06	4.64	-25079.02	8.99	-25038.23	17.11	-25007.34	15.31	-24969.52	20.45	-24931.02	42.61
-25154.34	-0.96	-25116.02	4.68	-25080.49	7.53	-25043.79	11.54	-25006.22	16.43	-24966.29	23.68	-24927.15	46.48
-25154.30	-0.92	-25116.96	3.73	-25080.03	7.99	-25039.46	15.87	-25003.13	19.52	-24968.38	21.59	-24929.70	43.92
-25154.95	-1.57	-25116.86	3.83	-25080.90	7.12	-25044.77	10.57	-25004.23	18.42	-24967.09	22.88	-24934.86	38.77
-25154.72	-1.34	-25116.30	4.40	-25080.42	7.60	-25040.88	14.45	-25002.75	19.90	-24970.95	19.02	-24930.66	42.97
-25154.09	-0.71	-25115.41	5.29	-25077.72	10.30	-25044.01	11.32	-25006.17	16.48	-24969.02	20.95	-24925.49	48.14
-25154.80	-1.42	-25115.37	5.33	-25081.32	6.70	-25044.19	11.15	-25005.69	16.96	-24968.76	21.21	-24922.35	51.28
-25153.80	-0.42	-25117.72	2.98	-25079.90	8.12	-25040.31	15.02	-25004.56	18.10	-24965.73	24.24	-24927.40	46.23
-25154.64	-1.26	-25115.57	5.13	-25079.75	8.26	-25040.62	14.71	-25009.35	13.30	-24969.78	20.19	-24929.72	43.90
-25154.18	-0.80	-25116.40	4.30	-25077.90	10.12	-25044.56	10.77	-25004.93	17.72	-24966.31	23.66	-24928.56	45.07
-25153.05	0.33	-25117.35	3.34	-25078.79	9.22	-25043.35	11.98	-25007.20	15.45	-24966.45	23.52	-24933.25	40.37
-25154.03	-0.65	-25116.49	4.20	-25078.95	9.06	-25042.54	12.80	-25004.12	18.53	-24968.25	21.72	-24933.34	40.29
-25153.80	-0.42	-25117.56	3.13	-25078.44	9.57	-25043.38	11.95	-25007.31	15.34	-24970.56	19.41	-24931.53	42.10
-25153.79	-0.41	-25115.91	4.78	-25080.69	7.33	-25039.22	16.12	-25004.85	17.81	-24966.77	23.19	-24929.12	44.50
-25152.93	0.45	-25116.76	3.94	-25079.97	8.04	-25042.63	12.70	-25004.47	18.18	-24971.00	18.97	-24930.69	42.94
-25153.92	-0.54	-25117.91	2.79	-25078.79	9.22	-25041.96	13.37	-25004.18	18.47	-24961.91	28.05	-24928.88	44.74
-25154.99	-1.61	-25116.75	3.95	-25077.61	10.41	-25040.80	14.53	-25007.55	15.10	-24966.94	23.03	-24932.42	41.21
-25152.98	0.40	-25117.70	3.00	-25078.88	9.14	-25043.19	12.14	-25002.96	19.69	-24963.73	26.23	-24929.78	43.84
-25153.42	-0.04	-25116.63	4.06	-25077.59	10.42	-25043.58	11.75	-24999.34	23.31	-24968.35	21.62	-24931.53	42.09
-25153.86	-0.48	-25115.72	4.98	-25077.84	10.17	-25043.53	11.81	-25007.52	15.13	-24965.37	24.60	-24925.02	48.61
-25153.22	0.16	-25119.08	1.62	-25079.53	8.49	-25040.04	15.29	-25007.57	15.08	-24965.14	24.83	-24931.51	42.12
-25153.49	-0.12	-25116.78	3.92	-25078.08	9.94	-25040.44	14.90	-25004.86	17.79	-24963.88	26.09	-24928.37	45.26
-25152.73	0.65	-25115.88	4.81	-25081.21	6.80	-25040.26	15.07	-25001.91	20.74	-24968.69	21.28	-24932.22	41.40
-25154.45	-1.07	-25116.93	3.76	-25078.81	9.20	-25041.11	14.23	-25003.77	18.88	-24961.25	28.72	-24933.23	40.40
-25154.93	-1.55	-25116.02	4.68	-25077.57	10.44	-25045.12	10.21	-25001.91	20.74	-24964.36	25.61	-24929.61	44.02
-25154.81	-1.43	-25115.94	4.76	-25078.50	9.51	-25042.23	13.11	-25001.27	21.38	-24969.61	20.36	-24928.61	45.02
-25154.00	-0.62	-25116.75	3.95	-25078.99	9.02	-25043.02	12.31	-25002.61	20.04	-24966.12	23.85	-24927.14	46.49
-25153.39	-0.01	-25117.60	3.10	-25081.32	6.69	-25041.55	13.79	-25007.16	15.50	-24968.93	21.04	-24933.08	40.55
-25154.73	-1.36	-25116.52	4.18	-25079.81	8.21	-25043.19	12.14	-25004.45	18.20	-24965.27	24.70	-24933.50	40.12
-25152.81	0.57	-25116.04	4.66	-25079.77	8.24	-25041.92	13.41	-25002.57	20.08	-24969.13	20.84	-24932.78	40.85
-25154.18	-0.80	-25118.08	2.62	-25079.42	8.60	-25042.65	12.69	-25004.40	18.25	-24966.55	23.42	-24929.95	43.68
-25153.43	-0.05	-25116.67	4.03	-25080.52	7.49	-25038.70	16.63	-25003.51	19.14	-24963.60	26.37	-24932.36	41.26
-25153.71	-0.33	-25117.07	3.63	-25080.07	7.94	-25039.69	15.65	-25003.60	19.06	-24968.36	21.61	-24932.21	41.42

-25153.19	0.19	-25115.67	5.02	-25079.92	8.09	-25042.58	12.75	-25002.81	19.84	-24970.38	19.59	-24931.43	42.20
-25154.29	-0.91	-25117.94	2.75	-25078.51	9.50	-25042.14	13.20	-25004.35	18.30	-24967.70	22.27	-24931.19	42.44
-25154.69	-1.31	-25117.51	3.19	-25079.47	8.55	-25041.44	13.90	-25006.82	15.83	-24967.40	22.57	-24927.39	46.23
-25152.11	1.27	-25116.75	3.94	-25079.21	8.81	-25040.65	14.68	-25007.46	15.19	-24966.72	23.25	-24932.05	41.58
-25153.92	-0.54	-25115.17	5.53	-25078.91	9.10	-25039.12	16.22	-25005.29	17.36	-24964.27	25.70	-24932.71	40.92
-25152.73	0.65	-25117.95	2.75	-25078.26	9.76	-25042.59	12.75	-25003.28	19.37	-24969.40	20.57	-24929.40	44.23
-25151.63	1.75	-25116.84	3.85	-25080.46	7.55	-25041.91	13.42	-25007.24	15.41	-24969.33	20.64	-24931.81	41.82
-25154.41	-1.03	-25115.31	5.39	-25076.19	11.82	-25040.34	14.99	-25004.80	17.86	-24966.74	23.23	-24933.25	40.37
-25154.79	-1.41	-25118.06	2.63	-25081.37	6.65	-25043.40	11.94	-25005.73	16.92	-24966.84	23.13	-24932.84	40.79
-25154.14	-0.76	-25118.54	2.15	-25080.03	7.98	-25038.47	16.86	-25003.92	18.73	-24962.07	27.90	-24924.24	49.39
-25153.99	-0.61	-25116.87	3.83	-25076.48	11.54	-25043.19	12.14	-25005.42	17.23	-24971.15	18.81	-24926.96	46.67
-25153.41	-0.03	-25117.93	2.77	-25080.90	7.12	-25037.91	17.43	-25004.50	18.16	-24970.41	19.56	-24934.94	38.69
-25154.80	-1.42	-25115.72	4.97	-25079.46	8.56	-25039.98	15.35	-25006.88	15.77	-24969.81	20.16	-24932.46	41.17
-25153.58	-0.20	-25117.72	2.97	-25080.01	8.00	-25045.35	9.98	-25007.77	14.88	-24971.21	18.76	-24930.05	43.57
-25155.04	-1.66	-25118.39	2.31	-25079.30	8.71	-25037.78	17.55	-25004.21	18.45	-24968.80	21.17	-24932.81	40.82
-25154.64	-1.26	-25114.16	6.53	-25081.77	6.25	-25042.75	12.58	-25005.23	17.42	-24971.11	18.86	-24931.56	42.07
-25153.09	0.29	-25117.58	3.11	-25079.94	8.07	-25040.87	14.46	-25007.45	15.21	-24965.59	24.38	-24928.97	44.66
-25154.03	-0.65	-25115.52	5.18	-25080.06	7.95	-25040.57	14.76	-25002.97	19.68	-24970.80	19.17	-24934.29	39.33
-25152.88	0.50	-25115.89	4.80	-25077.46	10.56	-25039.29	16.04	-25003.79	18.86	-24968.10	21.87	-24930.71	42.92
-25153.24	0.14	-25118.11	2.59	-25077.82	10.19	-25043.57	11.76	-25005.32	17.33	-24965.20	24.77	-24926.62	47.01
-25154.17	-0.79	-25115.85	4.85	-25080.50	7.51	-25041.57	13.77	-25004.38	18.28	-24970.55	19.42	-24931.86	41.77
-25153.91	-0.53	-25116.30	4.39	-25075.55	12.47	-25043.45	11.88	-25005.63	17.02	-24966.52	23.45	-24932.74	40.89
-25153.51	-0.13	-25115.83	4.86	-25081.07	6.94	-25041.55	13.78	-25005.93	16.72	-24966.36	23.61	-24929.09	44.54
-25153.62	-0.24	-25113.82	6.88	-25080.45	7.57	-25042.64	12.70	-25007.25	15.40	-24966.37	23.60	-24932.05	41.58
-25154.33	-0.95	-25115.63	5.07	-25080.08	7.93	-25041.95	13.38	-25003.76	18.89	-24969.03	20.93	-24934.24	39.39
-25154.68	-1.30	-25117.82	2.87	-25078.05	9.97	-25044.30	11.03	-25006.19	16.46	-24965.86	24.11	-24925.66	47.97
-25154.05	-0.67	-25117.55	3.15	-25078.59	9.43	-25041.42	13.92	-24997.32	25.34	-24967.10	22.87	-24934.24	39.39
-25153.82	-0.44	-25117.65	3.04	-25078.85	9.16	-25042.63	12.71	-25001.62	21.03	-24961.63	28.34	-24934.99	38.64
-25153.20	0.18	-25118.68	2.01	-25080.82	7.19	-25042.57	12.76	-25001.58	21.07	-24969.70	20.27	-24925.31	48.32
-25154.34	-0.96	-25115.85	4.85	-25079.26	8.76	-25042.08	13.25	-25006.41	16.24	-24966.69	23.28	-24929.95	43.67
-25152.59	0.79	-25116.11	4.58	-25076.40	11.62	-25040.32	15.02	-25003.74	18.91	-24966.26	23.70	-24929.78	43.85
-25153.49	-0.11	-25117.08	3.62	-25077.75	10.27	-25040.12	15.22	-25002.93	19.72	-24969.53	20.44	-24927.87	45.76
-25152.47	0.91	-25115.86	4.84	-25081.89	6.13	-25043.41	11.93	-25006.01	16.64	-24967.14	22.83	-24932.06	41.57
-25153.77	-0.39	-25114.62	6.07	-25076.52	11.50	-25039.29	16.04	-25004.43	18.23	-24971.36	18.61	-24932.63	41.00
-25154.36	-0.98	-25116.35	4.35	-25077.82	10.19	-25041.73	13.60	-25005.82	16.83	-24967.57	22.40	-24932.29	41.34
-25152.48	0.90	-25117.94	2.76	-25080.39	7.63	-25041.24	14.10	-25006.22	16.43	-24965.68	24.29	-24933.97	39.66
-25154.41	-1.03	-25117.19	3.50	-25080.42	7.60	-25042.97	12.36	-25007.45	15.20	-24969.39	20.58	-24932.33	41.29
-25153.63	-0.25	-25119.32	1.38	-25078.08	9.94	-25038.60	16.73	-25001.36	21.29	-24970.58	19.39	-24931.89	41.74
-25154.57	-1.19	-25116.77	3.92	-25077.82	10.19	-25039.33	16.00	-25003.03	19.62	-24966.69	23.27	-24930.50	43.13
-25154.59	-1.21	-25116.15	4.55	-25079.05	8.96	-25041.45	13.88	-25005.57	17.08			-24932.56	41.06
-25154.01	-0.63	-25115.89	4.80	-25080.57	7.45	-25036.78	18.55	-25001.77	20.88			-24929.20	44.43

	-25154.38	-1.00	-25115.71	4.99	-25077.91	10.11	-25043.07	12.26	-25007.14	15.51			-24925.83	47.79
	-25154.58	-1.20			-25076.83	11.19	-25039.64	15.70					-24933.25	40.38
	-25153.57	-0.19			-25081.51	6.51	-25042.29	13.05					-24932.90	40.73
	-25152.82	0.56			-25079.59	8.43	-25042.48	12.85						
	-25153.71	-0.33			-25079.47	8.55	-25042.33	13.00						
	-25153.54	-0.16			-25078.46	9.56	-25038.61	16.73						
	-25154.24	-0.86					-25041.97	13.36						
	-25153.93	-0.55					-25043.27	12.07						
	-25153.36	0.02												
Average energies	-25153.83	-0.45	-25116.81	3.89	-25079.20	8.81	-25041.77	13.56	-25004.74	17.91	-24967.83	22.14	-24930.79	42.83

Pu

	ground state energy	solid solution energy												
concentration in solid solution (%)	3		6		9		12		15		18		21	
number of atoms	2		4		6		8		10		12		13	
	-25148.40	-1.00	-25105.45	3.27	-25063.86	6.20	-25023.07	8.32	-24970.49	22.23	-24934.39	19.67	-24913.57	21.16
	-25148.87	-1.48	-25105.26	3.47	-25058.54	11.51	-25019.39	12.00	-24978.30	14.43	-24934.77	19.29	-24910.44	24.28
	-25148.85	-1.46	-25105.22	3.51	-25062.61	7.45	-25022.59	8.80	-24977.33	15.39	-24935.14	18.92	-24912.06	22.66
	-25146.92	0.47	-25105.36	3.37	-25060.82	9.23	-25021.61	9.78	-24973.94	18.78	-24937.04	17.01	-24913.46	21.26
	-25148.19	-0.80	-25104.52	4.21	-25062.18	7.87	-25018.39	13.00	-24978.93	13.80	-24932.82	21.24	-24914.29	20.44
	-25148.24	-0.85	-25108.17	0.56	-25061.47	8.59	-25023.15	8.25	-24976.24	16.48	-24934.24	19.81	-24913.50	21.22
	-25147.94	-0.55	-25104.77	3.96	-25062.87	7.19	-25016.45	14.94	-24976.56	16.16	-24937.12	16.94	-24912.42	22.31
	-25148.07	-0.68	-25106.42	2.31	-25061.00	9.06	-25021.83	9.56	-24975.08	17.65	-24931.94	22.12	-24916.50	18.23
	-25148.06	-0.67	-25105.13	3.60	-25063.68	6.38	-25016.66	14.73	-24978.69	14.04	-24934.81	19.24	-24910.29	24.44
	-25147.69	-0.30	-25106.36	2.36	-25059.00	11.06	-25019.91	11.48	-24973.82	18.91	-24934.84	19.21	-24910.45	24.27
	-25149.21	-1.82	-25103.32	5.41	-25063.88	6.17	-25018.95	12.44	-24973.66	19.06	-24934.48	19.57	-24910.93	23.79
	-25147.66	-0.27	-25104.84	3.89	-25062.63	7.43	-25020.93	10.46	-24976.91	15.82	-24929.47	24.58	-24915.65	19.07
	-25147.31	0.09	-25104.38	4.34	-25061.82	8.23	-25022.63	8.77	-24979.62	13.10	-24932.84	21.22	-24912.17	22.56
	-25150.23	-2.84	-25104.15	4.58	-25061.28	8.78	-25022.69	8.71	-24977.98	14.74	-24932.67	21.38	-24914.48	20.24
	-25148.16	-0.76	-25106.50	2.23	-25062.06	7.99	-25021.23	10.16	-24978.06	14.67	-24936.82	17.24	-24909.41	25.31
	-25148.03	-0.64	-25106.60	2.13	-25062.61	7.45	-25018.28	13.11	-24977.16	15.57	-24936.97	17.09	-24917.23	17.49
	-25149.04	-1.65	-25104.62	4.10	-25061.50	8.55	-25020.90	10.49	-24977.82	14.91	-24936.42	17.64	-24912.04	22.68
	-25147.41	-0.02	-25105.76	2.96	-25063.75	6.31	-25020.21	11.18	-24978.24	14.48	-24933.25	20.80	-24908.46	26.26
	-25147.88	-0.48	-25106.64	2.09	-25065.50	4.56	-25019.09	12.30	-24975.11	17.61	-24935.44	18.62	-24912.71	22.01
	-25149.49	-2.09	-25105.10	3.63	-25061.33	8.73	-25017.54	13.85	-24975.58	17.15	-24934.52	19.54	-24913.33	21.39
	-25148.15	-0.75	-25106.69	2.04	-25062.30	7.76	-25016.91	14.48	-24976.16	16.57	-24932.65	21.41	-24916.51	18.21
	-25148.62	-1.23	-25104.79	3.94	-25063.08	6.98	-25020.54	10.85	-24974.77	17.95	-24935.32	18.74	-24912.63	22.09
	-25149.24	-1.85	-25106.87	1.86	-25062.81	7.25	-25021.74	9.65	-24979.40	13.32	-24938.79	15.27	-24914.24	20.49

-25148.38	-0.99	-25105.27	3.45	-25060.49	9.56	-25019.01	12.38	-24978.69	14.04	-24935.53	18.53	-24912.22	22.50
-25148.82	-1.42	-25103.18	5.55	-25063.26	6.80	-25018.19	13.20	-24978.70	14.02	-24936.44	17.62	-24912.77	21.95
-25147.49	-0.09	-25105.53	3.20	-25062.66	7.40	-25021.60	9.79	-24978.53	14.20	-24937.33	16.72	-24914.59	20.14
-25148.07	-0.67	-25104.03	4.70	-25059.75	10.30	-25023.08	8.31	-24975.26	17.46	-24938.00	16.05	-24911.89	22.83
-25148.21	-0.82	-25105.53	3.20	-25062.98	7.07	-25020.00	11.40	-24973.53	19.19	-24936.31	17.75	-24916.17	18.55
-25147.89	-0.50	-25107.26	1.47	-25062.22	7.84	-25013.49	17.90	-24978.68	14.04	-24938.52	15.53	-24911.74	22.98
-25149.10	-1.71	-25102.38	6.35	-25061.85	8.21	-25019.24	12.15	-24980.61	12.11	-24932.50	21.56	-24913.63	21.10
-25148.19	-0.80	-25104.71	4.01	-25064.52	5.54	-25021.31	10.08	-24979.05	13.67	-24940.22	13.83	-24915.31	19.41
-25148.40	-1.01	-25105.77	2.96	-25063.84	6.22	-25017.30	14.09	-24975.05	17.68	-24938.49	15.57	-24909.74	24.98
-25149.35	-1.96	-25106.25	2.48	-25060.61	9.45	-25020.69	10.70	-24976.16	16.57	-24938.36	15.69	-24916.24	18.49
-25147.75	-0.36	-25104.11	4.61	-25062.52	7.54	-25021.81	9.58	-24977.76	14.96	-24934.13	19.92	-24913.35	21.37
-25147.84	-0.44	-25104.48	4.24	-25061.50	8.55	-25021.85	9.54	-24978.07	14.65	-24933.76	20.30	-24908.95	25.77
-25149.28	-1.89	-25105.53	3.20	-25062.60	7.46	-25018.91	12.48	-24979.61	13.11	-24938.07	15.98	-24916.35	18.37
-25149.59	-2.19	-25103.83	4.90	-25061.90	8.16	-25021.83	9.56	-24976.42	16.30	-24930.79	23.27	-24910.54	24.18
-25148.85	-1.45	-25106.44	2.29	-25059.69	10.37	-25018.70	12.69	-24979.19	13.53	-24934.61	19.44	-24916.75	17.98
-25149.18	-1.78	-25105.12	3.61	-25060.25	9.81	-25020.18	11.21	-24978.02	14.71	-24938.63	15.43	-24915.04	19.68
-25148.16	-0.77	-25107.34	1.39	-25062.79	7.27	-25021.42	9.97	-24977.49	15.23	-24935.99	18.07	-24911.19	23.53
-25148.57	-1.18	-25106.31	2.42	-25063.50	6.55	-25018.64	12.76	-24978.19	14.53	-24938.45	15.60	-24917.08	17.65
-25147.79	-0.39	-25105.62	3.10	-25060.56	9.49	-25018.68	12.71	-24980.77	11.95	-24931.16	22.90	-24915.63	19.10
-25147.03	0.37	-25106.79	1.94	-25061.58	8.47	-25019.32	12.07	-24974.41	18.31	-24930.99	23.06	-24916.35	18.37
-25146.46	0.94	-25101.89	6.83	-25060.24	9.82	-25017.04	14.36	-24976.56	16.17	-24932.95	21.11	-24915.80	18.92
-25149.33	-1.93	-25104.22	4.50	-25062.08	7.98	-25020.96	10.43	-24970.24	22.49	-24936.09	17.97	-24915.83	18.89
-25150.00	-2.61	-25105.53	3.19	-25064.31	5.75	-25020.03	11.36	-24973.68	19.04	-24935.06	19.00	-24912.20	22.52
-25149.97	-2.58	-25105.09	3.64	-25065.35	4.70	-25019.10	12.29	-24978.59	14.14	-24935.04	19.02	-24915.16	19.57
-25148.27	-0.87	-25102.29	6.44	-25064.88	5.18	-25019.06	12.33	-24976.51	16.22	-24938.20	15.86	-24913.38	21.34
-25148.67	-1.28	-25105.62	3.11	-25063.79	6.27	-25019.90	11.50	-24979.28	13.44	-24939.63	14.43	-24914.57	20.15
-25148.34	-0.95	-25105.27	3.46	-25062.24	7.82	-25017.13	14.26	-24979.66	13.07	-24936.29	17.77	-24914.14	20.59
-25148.44	-1.05	-25105.39	3.34	-25063.47	6.59	-25019.91	11.48	-24977.41	15.31	-24931.38	22.67	-24913.87	20.85
-25149.61	-2.22	-25104.99	3.74	-25062.86	7.20	-25020.30	11.09	-24976.55	16.17	-24933.84	20.21	-24908.76	25.96
-25146.66	0.73	-25106.13	2.60	-25060.97	9.09	-25018.99	12.40	-24975.10	17.62	-24932.19	21.87	-24909.83	24.90
-25146.94	0.45	-25106.46	2.27	-25062.11	7.95	-25018.46	12.94	-24979.92	12.80	-24934.95	19.11	-24909.80	24.93
-25148.75	-1.35	-25105.60	3.13	-25063.74	6.31	-25022.05	9.34	-24976.79	15.94	-24937.43	16.63	-24916.74	17.98
-25148.71	-1.31	-25104.79	3.94	-25062.63	7.43	-25020.65	10.74	-24975.82	16.90	-24937.92	16.13	-24911.50	23.22
-25148.55	-1.16	-25104.23	4.49	-25064.60	5.46	-25020.81	10.58	-24975.24	17.49	-24935.94	18.12	-24917.89	16.83
-25148.19	-0.80	-25105.62	3.10	-25063.24	6.82	-25018.83	12.56	-24975.81	16.91	-24936.02	18.04	-24911.14	23.58
-25148.97	-1.58	-25104.11	4.62	-25061.53	8.53	-25020.25	11.14	-24979.46	13.27	-24935.21	18.85	-24912.96	21.77
-25147.37	0.02	-25104.92	3.80	-25065.40	4.66	-25016.16	15.23	-24976.77	15.96	-24935.48	18.58	-24912.74	21.98
-25149.05	-1.66	-25106.51	2.22	-25063.62	6.44	-25017.97	13.42	-24976.47	16.26	-24936.87	17.18	-24917.30	17.42
-25148.99	-1.59	-25105.58	3.15	-25061.84	8.22	-25019.55	11.84	-24973.17	19.55	-24932.64	21.42	-24916.10	18.63
-25147.90	-0.50	-25105.99	2.73	-25064.14	5.92	-25020.75	10.64	-24974.53	18.20	-24936.12	17.94	-24911.73	22.99
-25147.67	-0.27	-25105.35	3.38	-25060.87	9.19	-25018.42	12.97	-24975.52	17.20	-24938.19	15.87	-24912.22	22.50

-25148.81	-1.41	-25103.64	5.09	-25061.91	8.15	-25018.02	13.37	-24979.06	13.66	-24938.36	15.70	-24913.18	21.54
-25147.97	-0.57	-25105.39	3.34	-25062.05	8.01	-25017.57	13.82	-24974.24	18.49	-24934.63	19.42	-24914.04	20.68
-25149.11	-1.71	-25103.96	4.77	-25061.99	8.07	-25022.86	8.53	-24976.11	16.61	-24933.71	20.35	-24914.61	20.11
-25147.73	-0.33	-25106.29	2.44	-25064.60	5.46	-25019.85	11.54	-24976.72	16.01	-24936.82	17.24	-24909.96	24.77
-25148.31	-0.92	-25106.06	2.66	-25064.23	5.83	-25018.23	13.16	-24978.43	14.30	-24935.45	18.61	-24914.94	19.79
-25147.37	0.02	-25104.96	3.77	-25061.91	8.15	-25019.10	12.29	-24976.64	16.09	-24933.23	20.83	-24920.51	14.22
-25148.65	-1.26	-25105.82	2.90	-25064.99	5.07	-25021.09	10.30	-24977.83	14.90	-24934.03	20.03	-24914.75	19.97
-25149.37	-1.97	-25106.27	2.45	-25063.37	6.69	-25020.53	10.87	-24978.32	14.40	-24935.33	18.73	-24914.69	20.03
-25148.17	-0.78	-25105.51	3.22	-25063.73	6.33	-25023.26	8.13	-24975.40	17.33	-24936.17	17.89	-24908.14	26.58
-25148.06	-0.67	-25104.79	3.93	-25062.60	7.46	-25022.52	8.87	-24980.75	11.98	-24937.26	16.80	-24913.54	21.18
-25148.73	-1.33	-25105.43	3.30	-25061.96	8.10	-25017.99	13.40	-24979.32	13.40	-24938.29	15.76	-24911.76	22.96
-25149.08	-1.69	-25106.88	1.85	-25064.41	5.65	-25016.92	14.47	-24975.42	17.31	-24935.82	18.24	-24911.44	23.28
-25147.82	-0.42	-25104.29	4.43	-25061.61	8.45	-25019.18	12.21	-24976.07	16.66	-24936.72	17.33	-24916.52	18.20
-25146.98	0.42	-25105.21	3.51	-25061.07	8.99	-25020.15	11.24	-24977.40	15.33	-24930.53	23.52	-24914.83	19.89
-25149.09	-1.69	-25106.44	2.29	-25060.12	9.94	-25020.25	11.15	-24975.13	17.59	-24935.33	18.72	-24916.30	18.42
-25146.78	0.62	-25105.12	3.61	-25060.84	9.21	-25019.36	12.04	-24977.94	14.78	-24935.11	18.94	-24912.23	22.49
-25149.68	-2.29	-25107.13	1.59	-25061.00	9.06	-25021.10	10.29	-24974.34	18.38	-24933.48	20.58	-24911.22	23.50
-25147.68	-0.28	-25104.16	4.56	-25063.83	6.23	-25020.84	10.55	-24975.66	17.07	-24931.80	22.25	-24915.84	18.89
-25147.96	-0.57	-25105.64	3.09	-25062.17	7.89	-25020.25	11.14	-24977.73	14.99	-24936.71	17.34	-24910.10	24.62
-25147.66	-0.27	-25105.09	3.64	-25061.96	8.10	-25018.06	13.33	-24976.96	15.76	-24937.42	16.64	-24915.65	19.07
-25149.20	-1.80	-25101.36	7.37	-25061.47	8.59	-25015.90	15.49	-24978.90	13.82	-24936.11	17.94	-24912.83	21.89
-25148.54	-1.15	-25105.23	3.50	-25062.46	7.60	-25020.38	11.01	-24974.05	18.67	-24934.44	19.62	-24915.56	19.16
-25149.76	-2.37	-25104.26	4.46	-25064.47	5.59	-25020.39	11.00	-24980.38	12.35	-24933.06	21.00	-24914.05	20.67
-25148.20	-0.81	-25105.69	3.04	-25054.92	15.14	-25018.93	12.46	-24978.76	13.96	-24935.68	18.38	-24916.63	18.09
-25147.86	-0.47	-25105.98	2.74	-25061.78	8.27	-25019.04	12.35	-24976.53	16.20	-24934.64	19.42	-24914.33	20.39
-25148.01	-0.61	-25106.91	1.82	-25062.67	7.39	-25021.42	9.97	-24976.92	15.80	-24933.01	21.05	-24914.16	20.56
-25149.36	-1.96	-25103.29	5.43	-25062.43	7.63	-25020.04	11.35	-24978.58	14.14	-24937.48	16.58	-24914.33	20.40
-25147.94	-0.55	-25106.39	2.34	-25059.10	10.96	-25015.27	16.12	-24977.64	15.08	-24937.47	16.58	-24908.91	25.81
-25148.85	-1.46	-25103.28	5.44	-25062.62	7.44	-25019.23	12.16	-24977.01	15.72	-24936.04	18.02	-24910.83	23.89
-25149.07	-1.67	-25107.02	1.71	-25062.95	7.11	-25019.17	12.22	-24978.48	14.24	-24936.62	17.43	-24912.73	21.99
-25149.82	-2.42	-25106.13	2.60	-25061.48	8.57	-25020.26	11.13	-24974.31	18.42	-24936.38	17.68	-24913.55	21.17
-25148.71	-1.32	-25103.18	5.54	-25062.85	7.21	-25021.13	10.26	-24975.45	17.27	-24932.80	21.26	-24913.44	21.28
-25147.50	-0.10	-25106.07	2.66	-25061.89	8.17	-25018.17	13.22	-24980.88	11.84	-24936.36	17.70	-24915.73	19.00
-25147.33	0.06	-25101.23	7.50	-25061.53	8.53	-25020.12	11.27	-24979.27	13.45	-24933.10	20.95	-24914.97	19.76
-25148.24	-0.85	-25105.24	3.48	-25061.61	8.45	-25018.37	13.03	-24976.86	15.87	-24935.02	19.03	-24916.29	18.43
-25149.02	-1.63	-25105.47	3.26	-25061.04	9.02	-25020.54	10.85	-24976.12	16.60	-24932.80	21.26	-24908.96	25.76
-25147.77	-0.38	-25108.61	0.12	-25061.19	8.87	-25020.99	10.40	-24976.93	15.80	-24938.30	15.75	-24914.54	20.18
-25148.03	-0.64	-25105.71	3.01	-25061.16	8.90	-25020.71	10.68	-24978.99	13.74	-24939.67	14.39	-24917.15	17.57
-25148.20	-0.81	-25104.85	3.88	-25062.18	7.87	-25020.51	10.88	-24974.57	18.16	-24936.48	17.58	-24913.46	21.26
-25148.44	-1.04	-25105.24	3.49	-25062.31	7.75	-25020.26	11.13	-24978.76	13.96	-24935.74	18.31	-24914.08	20.65
-25147.06	0.34	-25106.40	2.33	-25063.55	6.51	-25016.15	15.24	-24976.58	16.15	-24933.36	20.70	-24914.75	19.97

	-25148.26	-0.86	-25104.12	4.61	-25061.51	8.55	-25020.36	11.03	-24979.08	13.65	-24932.19	21.87	-24913.25	21.47
	-25146.88	0.52	-25105.70	3.02	-25056.37	13.69	-25021.47	9.92	-24977.88	14.84	-24930.53	23.53	-24914.78	19.95
	-25146.05	1.34	-25104.40	4.33	-25063.93	6.13	-25018.09	13.30	-24977.31	15.41	-24936.82	17.23	-24914.49	20.23
	-25146.65	0.75	-25106.24	2.48	-25061.80	8.26	-25017.86	13.53	-24968.89	23.83	-24934.71	19.35	-24915.62	19.10
	-25147.66	-0.26	-25106.09	2.64	-25063.38	6.68	-25021.63	9.76	-24974.66	18.06	-24931.88	22.18	-24909.79	24.93
	-25149.97	-2.57	-25105.90	2.83	-25062.08	7.98	-25020.06	11.33	-24975.86	16.87	-24936.03	18.03	-24913.51	21.22
	-25148.28	-0.89	-25106.45	2.28	-25060.08	9.98	-25019.11	12.28	-24979.43	13.29	-24937.67	16.39		
	-25148.47	-1.07	-25105.61	3.12	-25061.22	8.84	-25016.65	14.74	-24976.42	16.30	-24934.85	19.20		
	-25148.66	-1.27	-25106.94	1.79	-25062.61	7.45	-25021.44	9.95	-24979.34	13.38	-24936.54	17.52		
	-25148.07	-0.67	-25105.67	3.06			-25019.39	12.00						
	-25146.87	0.52	-25105.41	3.31			-25019.99	11.40						
	-25149.49	-2.10					-25018.84	12.55						
Average energies	-25148.32	-0.93	-25105.31	3.42	-25062.22	7.84	-25019.70	11.69	-24976.94	15.78	-24935.30	18.75	-24913.54	21.18

Th

	ground state energy	solid solution energy												
concentration in solid solution (%)	3		6		9		12		15		18		21	
number of atoms	2		4		6		8		10		12		13	
	-25152.93	0.09	-25115.53	4.45	-25080.24	6.71	-25042.77	11.14	-24997.01	23.87	-24966.59	21.25	-24944.70	26.62
	-25153.91	-0.88	-25115.94	4.05	-25079.27	7.68	-25040.68	13.24	-25003.15	17.73	-24963.28	24.56	-24949.69	21.63
	-25153.19	-0.16	-25118.15	1.84	-25076.91	10.04	-25038.26	15.65	-25004.08	16.79	-24962.96	24.88	-24950.75	20.57
	-25153.98	-0.96	-25114.86	5.13	-25078.68	8.27	-25043.06	10.86	-25001.54	19.34	-24965.28	22.56	-24949.53	21.79
	-25155.19	-2.16	-25116.53	3.46	-25077.63	9.32	-25040.21	13.71	-25001.32	19.55	-24967.57	20.27	-24946.70	24.62
	-25153.94	-0.91	-25115.07	4.92	-25078.44	8.51	-25041.40	12.51	-24999.75	21.13	-24960.88	26.96	-24948.89	22.43
	-25153.95	-0.93	-25116.23	3.76	-25080.17	6.78	-25041.67	12.25	-25000.06	20.82	-24964.38	23.46	-24945.67	25.65
	-25154.11	-1.09	-25116.35	3.64	-25077.40	9.55	-25039.14	14.78	-25005.79	15.09	-24966.52	21.32	-24944.81	26.51
	-25153.78	-0.75	-25115.94	4.05	-25077.65	9.30	-25042.66	11.25	-25004.10	16.78	-24965.07	22.77	-24947.45	23.87
	-25153.60	-0.57	-25116.54	3.45	-25080.77	6.18	-25041.77	12.15	-25000.39	20.48	-24964.68	23.16	-24946.11	25.21
	-25155.19	-2.16	-25115.06	4.93	-25078.88	8.07	-25038.55	15.37	-24998.03	22.85	-24961.80	26.04	-24949.27	22.05
	-25153.75	-0.73	-25116.86	3.13	-25078.14	8.81	-25039.15	14.76	-25001.75	19.12	-24967.38	20.46	-24944.06	27.26
	-25153.25	-0.22	-25117.06	2.93	-25078.83	8.12	-25041.50	12.42	-25004.11	16.76	-24965.76	22.08	-24946.93	24.39
	-25153.59	-0.57	-25115.76	4.23	-25078.36	8.59	-25042.74	11.17	-25004.60	16.27	-24959.73	28.11	-24945.13	26.19
	-25153.48	-0.45	-25114.81	5.18	-25076.56	10.39	-25040.16	13.76	-25004.10	16.77	-24964.46	23.37	-24946.95	24.37
	-25153.45	-0.42	-25114.64	5.35	-25079.87	7.08	-25040.70	13.22	-25003.88	16.99	-24963.62	24.22	-24948.14	23.18
	-25153.73	-0.71	-25115.60	4.38	-25079.16	7.79	-25042.10	11.81	-24999.08	21.80	-24966.83	21.01	-24948.66	22.66
	-25152.48	0.54	-25115.12	4.86	-25079.71	7.24	-25040.42	13.49	-25002.94	17.93	-24968.72	19.12	-24946.06	25.26

-25153.73	-0.70	-25115.77	4.22	-25076.96	9.99	-25040.56	13.35	-25003.69	17.18	-24966.10	21.73	-24948.87	22.45
-25154.79	-1.76	-25115.69	4.29	-25079.75	7.20	-25039.27	14.64	-25004.09	16.79	-24966.38	21.46	-24946.88	24.44
-25154.20	-1.18	-25115.94	4.05	-25078.96	7.99	-25039.28	14.64	-25003.60	17.28	-24962.85	24.99	-24949.90	21.42
-25154.25	-1.23	-25115.01	4.98	-25078.52	8.43	-25032.75	21.17	-25001.32	19.55	-24965.46	22.38	-24943.30	28.02
-25153.47	-0.45	-25115.98	4.01	-25076.15	10.80	-25042.73	11.18	-25005.05	15.83	-24963.51	24.33	-24945.40	25.92
-25154.18	-1.15	-25113.91	6.08	-25076.76	10.19	-25041.26	12.66	-25004.11	16.77	-24963.27	24.57	-24948.92	22.40
-25153.23	-0.20	-25116.38	3.60	-25078.23	8.72	-25037.65	16.27	-25002.19	18.68	-24966.85	20.98	-24951.14	20.18
-25154.47	-1.45	-25115.97	4.02	-25076.51	10.44	-25041.23	12.69	-25000.97	19.91	-24961.41	26.43	-24950.47	20.85
-25152.52	0.50	-25116.33	3.66	-25078.78	8.17	-25038.85	15.06	-24996.82	24.05	-24964.44	23.40	-24948.02	23.30
-25154.20	-1.18	-25115.90	4.09	-25078.56	8.39	-25041.80	12.11	-24998.90	21.97	-24968.59	19.25	-24946.02	25.30
-25152.73	0.29	-25116.49	3.50	-25079.28	7.67	-25041.53	12.38	-25005.25	15.63	-24963.26	24.58	-24947.68	23.64
-25154.87	-1.85	-25116.08	3.91	-25077.13	9.82	-25041.19	12.72	-25001.70	19.18	-24963.71	24.13	-24943.11	28.21
-25154.00	-0.97	-25113.98	6.00	-25079.84	7.11	-25040.18	13.73	-25003.19	17.69	-24968.75	19.08	-24944.99	26.33
-25151.80	1.22	-25116.22	3.76	-25079.19	7.76	-25042.89	11.02	-25005.05	15.83	-24968.99	18.85	-24949.92	21.40
-25152.52	0.50	-25115.55	4.44	-25078.23	8.72	-25042.20	11.72	-25002.27	18.61	-24966.44	21.40	-24946.06	25.26
-25153.13	-0.11	-25116.95	3.04	-25081.54	5.41	-25042.89	11.03	-25003.94	16.94	-24966.16	21.68	-24944.89	26.43
-25154.77	-1.75	-25114.87	5.12	-25078.47	8.48	-25040.97	12.94	-25000.65	20.23	-24965.96	21.88	-24946.81	24.51
-25151.87	1.16	-25115.19	4.79	-25080.78	6.17	-25039.72	14.19	-25002.02	18.86	-24964.42	23.42	-24950.13	21.19
-25154.15	-1.13	-25114.68	5.31	-25081.30	5.65	-25040.91	13.00	-25004.47	16.40	-24964.17	23.66	-24949.65	21.67
-25154.76	-1.73	-25115.42	4.57	-25077.87	9.08	-25038.91	15.00	-25005.39	15.49	-24963.49	24.35	-24946.67	24.65
-25154.06	-1.04	-25119.00	0.98	-25078.01	8.94	-25039.54	14.37	-25003.83	17.04	-24962.70	25.13	-24946.23	25.09
-25154.18	-1.16	-25114.71	5.27	-25075.72	11.23	-25040.92	12.99	-25001.79	19.09	-24967.59	20.25	-24947.66	23.66
-25153.70	-0.67	-25115.30	4.69	-25076.34	10.61	-25038.66	15.26	-25003.07	17.81	-24960.93	26.90	-24947.40	23.92
-25153.92	-0.89	-25116.04	3.95	-25077.48	9.47	-25037.61	16.30	-25002.64	18.24	-24967.55	20.29	-24947.60	23.72
-25153.66	-0.63	-25118.18	1.80	-25075.45	11.50	-25038.43	15.48	-25003.99	16.88	-24967.52	20.32	-24938.61	32.71
-25154.47	-1.45	-25115.10	4.89	-25078.97	7.98	-25041.82	12.10	-25000.26	20.61	-24963.35	24.48	-24939.68	31.64
-25152.85	0.17	-25116.17	3.82	-25075.84	11.11	-25041.65	12.26	-25002.73	18.14	-24963.42	24.42	-24945.41	25.91
-25153.07	-0.04	-25115.96	4.03	-25079.63	7.32	-25043.96	9.95	-25005.44	15.43	-24964.62	23.22	-24943.90	27.42
-25154.90	-1.87	-25114.43	5.55	-25077.00	9.95	-25037.14	16.77	-25002.45	18.43	-24949.55	38.29	-24946.36	24.96
-25154.49	-1.46	-25114.27	5.72	-25078.46	8.49	-25041.25	12.66	-25004.95	15.92	-24967.62	20.22	-24947.68	23.64
-25153.01	0.02	-25116.79	3.20	-25076.20	10.75	-25040.53	13.39	-24998.81	22.07	-24964.80	23.04	-24946.23	25.09
-25153.30	-0.28	-25116.80	3.19	-25078.45	8.50	-25039.12	14.79	-25003.87	17.01	-24960.54	27.30	-24941.89	29.43
-25153.42	-0.40	-25113.76	6.23	-25076.05	10.91	-25036.76	17.15	-25004.21	16.67	-24965.67	22.17	-24943.55	27.77
-25154.25	-1.22	-25116.30	3.68	-25073.67	13.28	-25040.14	13.77	-25000.14	20.74	-24967.91	19.92	-24948.39	22.93
-25153.85	-0.83	-25116.60	3.39	-25078.27	8.68	-25039.43	14.48	-25003.06	17.82	-24966.82	21.02	-24946.40	24.92
-25154.18	-1.16	-25115.77	4.22	-25078.11	8.84	-25041.31	12.61	-25008.31	12.56	-24969.21	18.63	-24949.17	22.15
-25153.11	-0.09	-25117.00	2.98	-25076.94	10.01	-25039.62	14.29	-25003.98	16.90	-24966.93	20.90	-24944.46	26.86
-25152.76	0.26	-25116.12	3.87	-25075.69	11.27	-25041.32	12.59	-25003.77	17.11	-24962.57	25.27	-24948.33	22.99
-25153.67	-0.65	-25117.54	2.45	-25075.73	11.22	-25042.91	11.00	-25002.61	18.27	-24967.54	20.30	-24942.57	28.75
-25153.91	-0.89	-25116.61	3.38	-25078.72	8.23	-25037.23	16.68	-24997.38	23.49	-24965.52	22.32	-24948.31	23.01
-25154.22	-1.19	-25113.24	6.74	-25078.41	8.54	-25036.27	17.65	-25044.61	-23.74	-24964.75	23.09	-24949.20	22.12

-25153.85	-0.83	-25116.79	3.20	-25079.27	7.68	-25039.23	14.68	-25002.59	18.28	-24963.68	24.16	-24941.29	30.03
-25154.23	-1.20	-25115.89	4.10	-25077.48	9.47	-25041.42	12.49	-25003.88	16.99	-24962.29	25.55	-24946.88	24.44
-25153.55	-0.52	-25115.70	4.29	-25078.06	8.89	-25041.66	12.25	-24968.92	51.96	-24958.33	29.51	-24946.89	24.43
-25153.81	-0.79	-25114.95	5.04	-25076.75	10.20	-25040.33	13.58	-25000.49	20.39	-24964.61	23.22	-24942.26	29.06
-25152.63	0.40	-25116.94	3.05	-25079.87	7.08	-25041.40	12.51	-25006.00	14.88	-24962.90	24.94	-24941.80	29.52
-25151.64	1.38	-25114.19	5.79	-25077.05	9.90	-25040.77	13.14	-25003.95	16.93	-24959.36	28.48	-24945.43	25.89
-25154.73	-1.70	-25113.88	6.11	-25077.80	9.15	-25041.05	12.86	-25002.97	17.90	-24967.51	20.33	-24943.31	28.01
-25153.46	-0.44	-25117.08	2.90	-25073.40	13.55	-25039.15	14.77	-25003.68	17.19	-24965.87	21.96	-24940.24	31.08
-25152.18	0.84	-25116.61	3.38	-25078.94	8.01	-25039.84	14.08	-25003.55	17.33	-24963.38	24.46	-24949.14	22.18
-25153.17	-0.14	-25116.58	3.40	-25078.01	8.94	-25037.88	16.04	-25003.55	17.33	-24967.89	19.95	-24944.86	26.46
-25153.43	-0.41	-25115.42	4.57	-25077.38	9.57	-25041.13	12.78	-25004.34	16.53	-24960.57	27.27	-24948.50	22.82
-25154.21	-1.18	-25115.53	4.46	-25072.05	14.90	-25041.14	12.78	-25000.78	20.09	-24965.58	22.26	-24946.65	24.67
-25153.67	-0.65	-25113.99	6.00	-25076.31	10.64	-25039.22	14.69	-25003.70	17.17	-24964.15	23.69	-24945.61	25.71
-25154.19	-1.17	-25115.04	4.95	-25078.88	8.07	-25041.74	12.18	-25002.09	18.79	-24963.75	24.09	-24944.42	26.90
-25154.33	-1.31	-25115.17	4.82	-25077.84	9.11	-25040.00	13.91	-25004.55	16.33	-24968.37	19.47	-24942.91	28.41
-25154.12	-1.09	-25116.82	3.16	-25080.01	6.94	-25039.66	14.26	-25000.99	19.89	-24966.08	21.76	-24949.79	21.53
-25153.65	-0.63	-25116.16	3.83	-25078.69	8.26	-25037.30	16.61	-25002.79	18.08	-24961.28	26.56	-24949.19	22.13
-25153.77	-0.74	-25114.03	5.96	-25079.59	7.36	-25042.97	10.94	-25004.56	16.31	-24967.13	20.71	-24946.95	24.37
-25153.09	-0.07	-25117.95	2.04	-25077.84	9.11	-25041.14	12.78	-25004.99	15.89	-24961.98	25.86	-24948.42	22.90
-25153.80	-0.78	-25116.33	3.66	-25079.18	7.77	-25041.91	12.00	-25001.80	19.07	-24966.90	20.94	-24942.25	29.07
-25153.55	-0.53	-25115.03	4.95	-25076.68	10.27	-25038.60	15.31	-25003.75	17.13	-24964.73	23.11	-24946.17	25.15
-25154.14	-1.12	-25115.00	4.99	-25079.52	7.43	-25042.55	11.37	-25000.28	20.60	-24968.22	19.62	-24944.58	26.74
-25155.52	-2.49	-25117.77	2.22	-25078.50	8.45	-25038.01	15.91	-25001.21	19.67	-24961.80	26.04	-24937.98	33.34
-25153.82	-0.79	-25118.01	1.98	-25079.42	7.53	-25035.61	18.30	-25001.23	19.64	-24963.01	24.83	-24948.29	23.03
-25154.40	-1.38	-25115.51	4.48	-25079.95	7.00	-25041.34	12.58	-25005.42	15.45	-24965.51	22.33	-24946.93	24.39
-25153.01	0.01	-25117.77	2.22	-25078.09	8.86	-25043.07	10.84	-25004.22	16.66	-24965.78	22.06	-24944.36	26.96
-25153.47	-0.45	-25115.43	4.55	-25078.63	8.33	-25039.51	14.41	-24996.65	24.23	-24964.32	23.52	-24948.03	23.29
-25155.20	-2.18	-25115.40	4.58	-25077.35	9.60	-25040.25	13.67	-25001.11	19.77	-24965.28	22.55	-24947.66	23.66
-25153.69	-0.66	-25116.14	3.85	-25080.88	6.07	-25043.43	10.48	-24999.26	21.61	-24964.45	23.38	-24944.95	26.37
-25153.61	-0.58	-25117.36	2.63	-25075.47	11.48	-25038.39	15.52	-25001.42	19.45	-24965.51	22.33	-24946.40	24.92
-25154.05	-1.03	-25117.66	2.33	-25073.33	13.62	-25040.85	13.06	-24998.84	22.03	-24965.98	21.86	-24944.31	27.01
-25153.27	-0.25	-25117.01	2.98	-25074.72	12.23	-25042.50	11.41	-25002.35	18.53	-24956.16	31.68	-24948.71	22.61
-25153.62	-0.59	-25117.26	2.73	-25076.69	10.26	-25042.88	11.03	-25005.36	15.51	-24968.71	19.13	-24940.83	30.49
-25153.80	-0.77	-25113.81	6.18	-25078.41	8.54	-25040.58	13.33	-25003.22	17.66	-24965.32	22.52	-24948.55	22.77
-25155.15	-2.12	-25115.86	4.13	-25078.46	8.49	-25038.30	15.61	-24996.78	24.09	-24966.42	21.42	-24945.93	25.39
-25154.40	-1.37	-25115.02	4.97	-25071.54	15.41	-25039.54	14.37	-25000.96	19.92	-24964.47	23.37	-24947.03	24.29
-25153.00	0.02	-25116.85	3.14	-25078.11	8.84	-25038.39	15.52	-25002.35	18.52	-24968.51	19.33	-24938.89	32.43
-25154.34	-1.32	-25117.25	2.74	-25079.71	7.24	-25039.80	14.11	-25004.86	16.02	-24967.54	20.30	-24948.97	22.35
-25154.31	-1.28	-25113.80	6.19	-25078.16	8.79	-25039.26	14.65	-25003.19	17.69	-24967.38	20.46	-24945.36	25.96
-25153.14	-0.11	-25111.86	8.13	-25077.15	9.81	-25040.38	13.53	-25002.95	17.93	-24963.26	24.58	-24947.29	24.03
-25153.93	-0.91	-25116.68	3.31	-25078.35	8.60	-25043.24	10.67	-25004.98	15.90	-24970.45	17.39	-24951.05	20.27

	-25153.75	-0.73	-25115.98	4.00	-25076.05	10.91	-25039.73	14.18	-25003.24	17.64	-24967.26	20.58	-24943.15	28.17
	-25154.06	-1.04	-25117.22	2.77	-25079.63	7.32	-25041.37	12.54	-24998.10	22.77	-24966.80	21.04	-24945.50	25.82
	-25152.59	0.43	-25115.10	4.89	-25077.44	9.51	-25039.09	14.82	-25004.22	16.66	-24961.47	26.37	-24951.35	19.97
	-25153.18	-0.16	-25114.63	5.36	-25078.97	7.98	-25041.15	12.77	-25002.64	18.23	-31573.07		-24943.50	27.82
	-25154.49	-1.47	-25115.97	4.01	-25078.13	8.82	-25040.27	13.65	-25002.55	18.32	-24961.62	26.21	-24946.43	24.89
	-25154.37	-1.34	-25114.77	5.22	-25080.67	6.28	-25039.08	14.84	-25004.11	16.77	-24965.44	22.39	-24948.32	23.00
	-25154.81	-1.79	-25116.59	3.40	-25076.22	10.73	-25040.55	13.36	-25003.47	17.41	-24964.10	23.74	-24949.72	21.60
	-25153.40	-0.38	-25116.81	3.18	-25076.78	10.17	-25037.32	16.59	-25004.97	15.90	-24966.09	21.75	-24941.45	29.86
	-25153.50	-0.48	-25114.88	5.11	-25076.18	10.77	-25042.04	11.88	-25004.43	16.44	-24965.93	21.91	-24945.13	26.19
	-25153.86	-0.84	-25116.72	3.27	-25081.16	5.79	-25041.96	11.96	-25004.81	16.07	-24963.52	24.32	-24945.74	25.58
	-25153.13	-0.10	-25116.11	3.88	-25078.89	8.06	-25042.27	11.65	-25003.86	17.02	-24964.76	23.08	-24947.56	23.76
	-25153.07	-0.05	-25114.68	5.31	-25078.60	8.35	-25038.31	15.60			-24966.79	21.04	-24948.87	22.45
	-25154.05	-1.02	-25115.39	4.60	-25079.38	7.57	-25034.88	19.03			-24964.61	23.23	-24947.93	23.39
	-25153.95	-0.92	-25117.04	2.95	-25078.43	8.52	-25039.90	14.01			-24965.73	22.10	-24944.67	26.65
	-25153.23	-0.21	-25115.10	4.89	-25079.35	7.60							-24942.71	28.61
	-25151.52	1.50	-25114.46	5.53									-24950.56	20.76
	-25154.01	-0.98	-25114.40	5.58									-24948.72	22.60
			-25117.25	2.74										
Average energies	-25153.73	-0.70	-25115.84	4.15	-25077.98	8.97	-25040.29	13.63	-25002.70	18.18	-25022.79	23.01	-24946.31	25.01

U

	ground state energy	solid solution energy												
concentration in solid solution (%)	3		6		9		12		15		18		21	
number of atoms	2		4		6		8		10		12		13	
	-25157.65	0.39	-25127.36	2.67	-25094.66	7.35	-25063.30	10.68	-25028.05	17.91	-24997.49	20.46	-24982.11	21.83
	-25157.59	0.45	-25122.21	7.81	-25090.36	11.64	-25059.57	14.41	-25026.29	19.68	-24997.66	20.29	-24973.01	30.93
	-25158.81	-0.76	-25122.73	7.29	-25094.82	7.19	-25055.76	18.23	-25027.76	18.21	-24997.75	20.20	-24984.15	19.79
	-25155.95	2.09	-25125.99	4.03	-25095.49	6.51	-25061.84	12.15	-25022.14	23.83	-24992.77	25.18	-24979.82	24.12
	-25158.13	-0.09	-25125.24	4.79	-25093.15	8.86	-25057.50	16.49	-25029.23	16.74	-24998.56	19.39	-24978.59	25.35
	-25149.24	8.80	-25124.62	5.41	-25094.60	7.41	-25061.53	12.46	-25032.15	13.82	-24989.86	28.09	-24978.88	25.06
	-25157.47	0.57	-25125.81	4.22	-25090.11	11.89	-25060.23	13.76	-25028.51	17.46	-24997.43	20.52	-24979.54	24.40
	-25149.67	8.37	-25125.38	4.65	-25092.36	9.65	-25058.61	15.38	-25032.38	13.59	-24995.96	21.99	-24981.50	22.44
	-25157.87	0.17	-25126.14	3.88	-25093.09	8.92	-25060.74	13.25	-25028.21	17.76	-24996.12	21.83	-24978.15	25.79
	-25156.37	1.68	-25126.17	3.85	-25095.63	6.38	-25063.81	10.18	-25028.86	17.11	-24994.58	23.37	-24983.85	20.10
	-25156.64	1.40	-25126.76	3.27	-25092.38	9.63	-25058.58	15.41	-25027.81	18.16	-24994.46	23.49	-24979.31	24.64
	-25157.98	0.07	-25125.88	4.15	-25090.12	11.89	-25042.99	31.00	-25030.22	15.75	-24997.52	20.43	-24979.37	24.57
	-25158.96	-0.92	-25126.51	3.51	-25087.89	14.12	-25062.80	11.18	-25030.09	15.88	-25000.62	17.33	-24972.73	31.21
	-25157.27	0.77	-25123.43	6.60	-25091.54	10.46	-25059.98	14.01	-25028.11	17.86	-24997.10	20.85	-24980.88	23.06

-25157.66	0.38	-25125.00	5.03	-25091.13	10.88	-25063.43	10.55	-25032.00	13.97	-24996.65	21.31	-24979.43	24.51
-25158.17	-0.12	-25123.68	6.35	-25092.04	9.97	-25057.71	16.28	-25030.18	15.79	-24996.40	21.55	-24976.88	27.06
-25158.13	-0.09	-25125.35	4.68	-25094.92	7.08	-25060.75	13.24	-25031.24	14.72	-24998.18	19.77	-24982.20	21.74
-25149.92	8.12	-25124.15	5.88	-25095.31	6.70	-25057.88	16.11	-25029.56	16.41	-24997.63	20.32	-24980.31	23.63
-25151.79	6.25	-25123.31	6.72	-25094.15	7.85	-25058.24	15.75	-25025.10	20.87	-24995.02	22.93	-24973.69	30.25
-25159.18	-1.13	-25123.48	6.55	-25090.73	11.27	-25062.08	11.90	-25026.91	19.06	-24997.97	19.98	-24978.15	25.80
-25157.17	0.88	-25125.91	4.11	-25095.66	6.35	-25062.21	11.78	-25026.04	19.93	-24997.61	20.34	-24977.26	26.68
-25157.94	0.11	-25126.86	3.17	-25092.41	9.60	-25060.08	13.91	-25022.68	23.29	-24996.92	21.03	-24981.12	22.82
-25149.43	8.62	-25125.46	4.57	-25092.69	9.32	-25060.43	13.56	-25029.74	16.23	-24997.99	19.96	-24979.72	24.22
-25158.82	-0.77	-25125.63	4.40	-25089.26	12.75	-25057.50	16.49	-25027.15	18.81	-24997.58	20.38	-24984.09	19.85
-25157.81	0.23	-25125.30	4.72	-25094.78	7.23	-25061.44	12.55	-25031.17	14.80	-24992.66	25.30	-24975.49	28.45
-25149.89	8.15	-25128.03	2.00	-25093.29	8.72	-25062.26	11.73	-25027.49	18.48	-24997.58	20.37	-24981.24	22.70
-25158.24	-0.20	-25125.50	4.52	-25092.36	9.65	-25061.99	12.00	-25025.78	20.19	-24992.54	25.41	-24977.67	26.28
-25158.43	-0.38	-25127.17	2.85	-25093.64	8.36	-25058.27	15.72	-25031.19	14.78	-24996.96	20.99	-24980.49	23.45
-25157.32	0.73	-25125.72	4.31	-25090.42	11.58	-25061.16	12.83	-25029.42	16.55	-24997.32	20.63	-24981.20	22.74
-25157.67	0.37	-25125.95	4.07	-25091.18	10.83	-25063.73	10.26	-25031.30	14.67	-24996.04	21.91	-24979.05	24.89
-25157.03	1.01	-25126.61	3.42	-25093.73	8.27	-25063.62	10.37	-25030.91	15.06	-24995.60	22.35	-24984.43	19.51
-25157.31	0.74	-25125.03	4.99	-25093.79	8.22	-25058.18	15.81	-25029.11	16.86	-24994.29	23.66	-24983.65	20.29
-25158.74	-0.70	-25126.61	3.42	-25092.21	9.80	-25061.86	12.13	-25028.79	17.18	-24999.20	18.75	-24979.51	24.44
-25157.07	0.97	-25125.63	4.39	-25092.36	9.65	-25058.35	15.64	-25024.48	21.49	-24992.91	25.04	-24981.17	22.77
-25157.95	0.10	-25127.16	2.87	-25091.95	10.05	-25063.08	10.91	-25027.50	18.47	-24996.38	21.57	-24981.81	22.13
-25156.88	1.16	-25125.12	4.90	-25093.45	8.56	-25057.64	16.35	-25029.87	16.10	-24997.53	20.42	-24978.05	25.90
-25157.43	0.61	-25124.13	5.89	-25093.44	8.57	-25060.49	13.50	-25029.65	16.32	-24998.23	19.72	-24982.46	21.48
-25158.10	-0.05	-25121.70	8.32	-25091.29	10.72	-25058.64	15.35	-25028.95	17.02	-24997.66	20.29	-24981.85	22.09
-25157.78	0.26	-25122.11	7.92	-25093.28	8.73	-25058.51	15.48	-25031.07	14.90	-24998.87	19.08	-24979.79	24.15
-25150.11	7.94	-25125.65	4.38	-25092.37	9.64	-25063.71	10.28	-25029.45	16.52	-24998.16	19.79	-24979.78	24.16
-25157.04	1.00	-25121.84	8.18	-25095.68	6.32	-25060.99	12.99	-25030.12	15.85	-25001.31	16.64	-24979.74	24.20
-25157.49	0.55	-25124.03	6.00	-25091.09	10.91	-25061.37	12.61	-25028.11	17.86	-24987.43	30.52	-24981.71	22.23
-25157.26	0.78	-25125.02	5.01	-25093.90	8.11	-25059.54	14.45	-25024.62	21.35	-24997.23	20.72	-24976.97	26.97
-25156.56	1.48	-25124.42	5.61	-25091.32	10.69	-25059.23	14.76	-25029.79	16.18	-24993.86	24.09	-24980.81	23.13
-25159.06	-1.02	-25127.30	2.73	-25095.46	6.54	-25061.70	12.29	-25025.52	20.45	-24992.86	25.09	-24972.62	31.33
-25150.72	7.33	-25122.82	7.20	-25093.12	8.89	-25059.99	13.99	-25025.81	20.16	-24996.72	21.23	-24984.52	19.43
-25149.43	8.61	-25125.21	4.81	-25092.08	9.93	-25061.75	12.24	-25027.55	18.42	-24998.23	19.72	-24974.56	29.38
-25156.48	1.56	-25115.27	14.76	-25092.52	9.49	-25059.31	14.67	-25027.81	18.16	-24995.22	22.73	-24982.22	21.72
-25158.27	-0.23	-25125.77	4.26	-25093.45	8.56	-25059.28	14.71	-25024.93	21.04	-24995.15	22.80	-24981.46	22.49
-25156.59	1.46	-25124.93	5.09	-25095.19	6.81	-25061.39	12.60	-25030.04	15.93	-24999.54	18.41	-24981.70	22.25
-25156.56	1.48	-25124.82	5.21	-25092.42	9.58	-25062.50	11.49	-25026.77	19.20	-24995.20	22.75	-24980.33	23.61
-25157.69	0.36	-25125.73	4.29	-25092.27	9.73	-25061.84	12.15	-25031.10	14.87	-24995.66	22.29	-24981.16	22.78
-25157.82	0.23	-25126.42	3.60	-25088.08	13.93	-25058.45	15.53	-25022.39	23.58	-24991.87	26.08	-24980.43	23.51
-25158.92	-0.87	-25125.73	4.30	-25093.46	8.55	-25061.29	12.70	-25026.46	19.51	-24996.02	21.93	-24979.37	24.57
-25149.58	8.46	-25125.86	4.16	-25092.32	9.69	-25061.92	12.07	-25027.20	18.77	-24995.59	22.37	-24984.22	19.72

	-25156.03	2.01	-25123.83	6.20	-25091.90	10.10	-25058.56	15.43	-25030.52	15.45	-24997.80	20.15	-24981.57	22.37
	-25157.40	0.64	-25124.59	5.44	-25094.54	7.47	-25061.93	12.06	-25028.95	17.02	-24997.34	20.61	-24981.92	22.02
	-25158.79	-0.75	-25123.15	6.87	-25092.87	9.14	-25061.82	12.16	-25028.83	17.14	-24996.86	21.10		
	-25157.45	0.60	-25124.50	5.52	-25091.14	10.86			-25027.89	18.07	-24997.95	20.00		
	-25157.14	0.90	-25123.07	6.95	-25093.59	8.41			-25027.20	18.77	-24993.30	24.65		
	-25157.91	0.14	-25124.68	5.34	-25094.08	7.93			-25026.43	19.54	-24997.53	20.42		
	-25155.19	2.85	-25124.51	5.52	-25091.42	10.59			-25030.85	15.12	-24996.12	21.83		
	-25158.73	-0.69	-25123.19	6.84	-25090.88	11.13			-25021.36	24.61	-24996.64	21.31		
	-25157.90	0.15	-25125.65	4.37	-25091.58	10.43			-25026.18	19.79	-24998.48	19.47		
	-25158.15	-0.11	-25125.91	4.12	-25093.74	8.27			-25026.32	19.65	-24997.11	20.84		
	-25157.65	0.40	-25125.40	4.63	-25094.25	7.75			-25027.42	18.55	-24995.68	22.27		
	-25157.82	0.23	-25125.41	4.62	-25089.25	12.75			-25028.18	17.78	-24995.41	22.54		
	-25156.94	1.11	-25126.23	3.80	-25094.92	7.08			-25031.30	14.67	-24998.75	19.20		
	-25158.34	-0.30	-25124.66	5.36	-25092.50	9.51			-25027.25	18.72	-24997.14	20.81		
	-25157.99	0.05	-25126.38	3.64	-25089.85	12.16			-25023.12	22.85				
	-25158.59	-0.55	-25126.83	3.19	-25092.85	9.15			-25032.66	13.31				
	-25157.72	0.33	-25125.13	4.90	-25089.49	12.52			-25026.07	19.90				
	-25158.14	-0.09	-25123.20	6.82	-25091.94	10.07			-25026.85	19.12				
	-25158.86	-0.82			-25092.27	9.74			-25028.25	17.72				
	-25156.11	1.93							-25024.90	21.07				
									-25030.67	15.30				
Average energies	-25156.64	1.40	-25124.96	5.06	-25092.61	9.39	-25060.23	13.76	-25028.11	17.86	-24996.37	21.58	-24979.96	23.98

$Ca_{1-x}M_xZrTi_{2-2x}Al_{2x}O_7$ M=(Ce, Pu, Th, U)

Ti(2)Ti(3)

N.B. All stated energies are in eV

Ce

	ground state energy	solid solution energy												
concentration in solid solution (%)	3		6		9		12		15		18		21	
number of atoms	2		4		6		8		10		12		13	
	-25156.65	-3.27	-25119.64	1.06	-25085.66	2.35	-25051.93	3.41	-25017.34	5.31	-24987.77	2.20	-24968.59	5.04
	-25157.69	-4.31	-25122.66	-1.96	-25086.90	1.11	-25051.47	3.86	-25019.91	2.74	-24986.27	3.70	-24966.11	7.51
	-25156.49	-3.11	-25122.91	-2.21	-25089.01	-0.99	-25053.71	1.62	-25020.16	2.49	-24983.84	6.13	-24970.93	2.70
	-25156.66	-3.28	-25122.74	-2.05	-25088.16	-0.15	-25052.61	2.73	-25015.15	7.51	-24987.63	2.33	-24963.29	10.34
	-25156.85	-3.47	-25121.96	-1.26	-25089.32	-1.31	-25054.86	0.47	-25019.08	3.57	-24985.85	4.12	-24966.13	7.50
	-25157.70	-4.32	-25121.22	-0.52	-25085.64	2.37	-25049.20	6.13	-25013.10	9.56	-24984.38	5.59	-24966.48	7.15
	-25156.80	-3.42	-25121.88	-1.18	-25087.02	1.00	-25055.95	-0.61	-25018.75	3.90	-24984.61	5.36	-24963.99	9.64
	-25155.64	-2.26	-25122.37	-1.67	-25088.56	-0.55	-25052.87	2.46	-25019.10	3.55	-24983.74	6.23	-24969.76	3.87
	-25155.73	-2.35	-25122.67	-1.98	-25089.57	-1.56	-25050.69	4.64	-25021.74	0.91	-24984.27	5.70	-24969.54	4.09
	-25156.00	-2.62	-25122.13	-1.43	-25086.86	1.16	-25051.77	3.56	-25017.24	5.41	-24982.20	7.77	-24969.29	4.33
	-25155.55	-2.17	-25122.41	-1.71	-25087.80	0.22	-25051.92	3.41	-25016.29	6.36	-24980.13	9.83	-24968.73	4.90
	-25156.65	-3.27	-25122.59	-1.89	-25086.33	1.69	-25054.73	0.60	-25018.44	4.21	-24986.71	3.26	-24970.89	2.73
	-25157.36	-3.98	-25122.66	-1.96	-25088.16	-0.15	-25055.29	0.04	-25018.08	4.57	-24984.96	5.01	-24968.58	5.05
	-25157.34	-3.96	-25122.42	-1.72	-25086.68	1.34	-25055.32	0.01	-25021.73	0.92	-24984.27	5.70	-24969.07	4.55
	-25156.81	-3.43	-25122.26	-1.56	-25086.44	1.57	-25054.36	0.97	-25018.75	3.90	-24987.03	2.93	-24964.18	9.44
	-25157.33	-3.95	-25120.83	-0.13	-25088.01	0.00	-25049.91	5.43	-25019.99	2.66	-24982.40	7.57	-24968.43	5.20
	-25156.22	-2.84	-25121.50	-0.81	-25088.25	-0.24	-25051.83	3.50	-25016.59	6.06	-24985.40	4.57	-24972.23	1.40
	-25156.93	-3.55	-25121.78	-1.08	-25087.41	0.60	-25054.17	1.17	-25017.51	5.14	-24984.88	5.09	-24966.60	7.03
	-25155.55	-2.17	-25123.09	-2.40	-25087.50	0.52	-25053.14	2.19	-25013.20	9.45	-24982.39	7.58	-24970.03	3.60
	-25155.32	-1.94	-25122.88	-2.19	-25087.49	0.53	-25054.29	1.04	-25019.48	3.17	-24985.30	4.67	-24964.85	8.78
	-25157.32	-3.94	-25121.48	-0.78	-25087.35	0.66	-25052.17	3.16	-25018.43	4.22	-24989.65	0.32	-24965.48	8.14
	-25156.98	-3.60	-25122.11	-1.41	-25085.19	2.82	-25053.33	2.00	-25020.28	2.37	-24985.34	4.63	-24962.89	10.74
	-25158.19	-4.81	-25123.24	-2.55	-25087.70	0.31	-25053.40	1.93	-25017.42	5.23	-24982.43	7.54	-24970.24	3.38
	-25156.63	-3.25	-25121.07	-0.38	-25084.64	3.38	-25052.52	2.81	-25019.04	3.61	-24986.54	3.43	-24969.01	4.62
	-25156.95	-3.57	-25121.52	-0.82	-25088.69	-0.67	-25051.28	4.05	-25019.53	3.12	-24981.98	7.99	-24968.15	5.47
	-25156.27	-2.89	-25124.58	-3.88	-25088.16	-0.14	-25053.12	2.22	-25020.02	2.63	-24986.96	3.01	-24962.72	10.91
	-25157.23	-3.85	-25122.14	-1.44	-25089.07	-1.06	-25047.21	8.12	-25014.59	8.06	-24982.25	7.72	-24968.89	4.73
	-25157.29	-3.91	-25120.91	-0.21	-25087.14	0.88	-25053.98	1.35	-25018.65	4.01	-24986.03	3.94	-24969.34	4.29
	-25155.52	-2.14	-25122.55	-1.85	-25087.62	0.39	-25054.46	0.88	-25018.62	4.04	-24988.55	1.42	-24968.62	5.01

-25156.47	-3.09	-25122.55	-1.85	-25086.17	1.84	-25054.03	1.30	-25015.40	7.25	-24984.26	5.71	-24967.70	5.92
-25156.49	-3.11	-25122.01	-1.31	-25088.03	-0.01	-25053.07	2.27	-25019.43	3.22	-24985.35	4.62	-24965.73	7.90
-25151.93	1.45	-25122.26	-1.56	-25087.34	0.68	-25054.80	0.53	-25018.13	4.52	-24986.48	3.48	-24965.73	7.90
-25157.88	-4.50	-25121.66	-0.96	-25088.39	-0.37	-25052.76	2.57	-25015.26	7.39	-24984.16	5.81	-24968.47	5.16
-25156.27	-2.89	-25121.37	-0.67	-25086.92	1.09	-25054.91	0.43	-25014.79	7.86	-24984.91	5.06	-24967.55	6.08
-25156.58	-3.20	-25122.76	-2.06	-25084.00	4.01	-25051.36	3.97	-25020.21	2.44	-24984.93	5.04	-24963.68	9.95
-25156.61	-3.23	-25122.66	-1.96	-25089.16	-1.14	-25053.20	2.13	-25020.15	2.51	-24987.77	2.20	-24971.33	2.30
-25156.00	-2.62	-25121.12	-0.43	-25087.16	0.85	-25052.14	3.20	-25021.55	1.10	-24986.46	3.50	-24967.18	6.45
-25155.89	-2.51	-25120.21	0.49	-25088.94	-0.92	-25053.73	1.61	-25019.64	3.01	-24986.54	3.43	-24968.92	4.71
-25156.76	-3.38	-25121.25	-0.55	-25084.69	3.32	-25051.06	4.28	-25018.81	3.84	-24985.65	4.32	-24963.91	9.72
-25156.53	-3.15	-25120.87	-0.18	-25086.73	1.28	-25051.95	3.39	-25018.66	3.99	-24981.87	8.10	-24968.05	5.57
-25156.86	-3.48	-25122.69	-1.99	-25087.38	0.64	-25047.94	7.40	-25022.90	-0.25	-24984.53	5.44	-24968.61	5.02
-25157.38	-4.00	-25122.73	-2.03	-25088.06	-0.04	-25056.78	-1.45	-25021.36	1.29	-24987.87	2.10	-24966.99	6.63
-25157.39	-4.01	-25122.87	-2.17	-25089.05	-1.04	-25055.56	-0.22	-25020.08	2.57	-24986.56	3.41	-24971.89	1.74
-25157.72	-4.34	-25120.18	0.52	-25086.33	1.69	-25055.87	-0.54	-25013.80	8.85	-24986.05	3.92	-24964.24	9.39
-25156.20	-2.82	-25123.64	-2.95	-25086.57	1.45	-25054.47	0.86	-25020.18	2.47	-24985.18	4.79	-24968.41	5.21
-25155.72	-2.34	-25123.14	-2.44	-25089.58	-1.56	-25051.83	3.50	-25022.25	0.40	-24980.00	9.96	-24968.89	4.74
-25156.48	-3.10	-25121.92	-1.23	-25086.80	1.22	-25052.28	3.06	-25018.58	4.07	-24980.69	9.28	-24966.44	7.19
-25156.76	-3.39	-25120.47	0.23	-25088.04	-0.03	-25051.09	4.24	-25016.17	6.48	-24984.94	5.03	-24968.41	5.22
-25156.87	-3.49	-25122.26	-1.56	-25089.32	-1.31	-25053.31	2.02	-25018.49	4.16	-24987.12	2.85	-24970.96	2.67
-25156.78	-3.40	-25122.95	-2.25	-25085.39	2.63	-25052.42	2.92	-25021.32	1.34	-24985.07	4.90	-24963.14	10.49
-25156.73	-3.35	-25121.31	-0.62	-25088.99	-0.97	-25056.54	-1.21	-25018.96	3.69	-24981.69	8.28	-24963.99	9.64
-25156.42	-3.05	-25121.27	-0.57	-25088.75	-0.74	-25052.93	2.40	-25019.92	2.73	-24987.23	2.74	-24968.01	5.62
-25156.90	-3.52	-25122.29	-1.60	-25084.87	3.15	-25053.76	1.57	-25018.70	3.95	-24982.93	7.04	-24968.07	5.56
-25157.79	-4.41	-25123.26	-2.56	-25086.42	1.59	-25056.46	-1.13	-25021.93	0.72	-24988.81	1.16	-24968.07	5.56
-25154.20	-0.82	-25122.86	-2.16	-25086.40	1.62	-25054.61	0.72	-25017.55	5.10	-24983.12	6.85	-24968.79	4.84
-25156.86	-3.48	-25122.67	-1.97	-25086.88	1.14	-25055.40	-0.06	-25016.59	6.06	-24986.90	3.07	-24970.41	3.22
-25158.08	-4.70	-25122.86	-2.16	-25087.00	1.02	-25051.15	4.19	-25020.47	2.19	-24984.65	5.32	-24969.51	4.12
-25156.06	-2.68	-25121.48	-0.78	-25087.83	0.19	-25049.57	5.77	-25018.34	4.31	-24985.26	4.71	-24966.35	7.28
-25156.54	-3.16	-25123.33	-2.63	-25089.67	-1.65	-25054.32	1.01	-25017.71	4.95	-24983.20	6.77	-24969.65	3.98
-25157.07	-3.69	-25122.82	-2.13	-25086.77	1.25	-25053.83	1.51	-25016.24	6.41	-24986.44	3.53	-24965.11	8.52
-25155.11	-1.73	-25118.74	1.96	-25090.43	-2.42	-25053.99	1.34	-25018.86	3.79	-24980.89	9.08	-24969.36	4.27
-25155.06	-1.68	-25118.67	2.03	-25088.94	-0.92	-25046.58	8.75	-25020.02	2.64	-24986.09	3.88	-24967.41	6.22
-25158.02	-4.64	-25120.85	-0.15	-25087.70	0.32	-25052.46	2.88	-25018.74	3.91	-24987.44	2.53	-24961.82	11.81
-25156.17	-2.79	-25122.12	-1.42	-25086.54	1.48	-25050.54	4.79	-25019.48	3.17	-24983.19	6.78	-24963.31	10.32
-25155.80	-2.42	-25121.06	-0.36	-25088.29	-0.27	-25055.89	-0.55	-25020.25	2.40	-24984.91	5.06	-24970.56	3.07
-25155.86	-2.48	-25121.08	-0.38	-25087.12	0.90	-25053.57	1.77	-25018.57	4.08	-24988.39	1.58	-24969.77	3.86
-25158.04	-4.66	-25123.46	-2.76	-25086.66	1.35	-25054.99	0.35	-25020.40	2.25	-24983.15	6.82	-24968.35	5.28
-25155.28	-1.90	-25122.36	-1.67	-25089.70	-1.69	-25054.13	1.20	-25017.24	5.41	-24984.07	5.90	-24963.58	10.05
-25157.18	-3.80	-25123.70	-3.00	-25085.91	2.10	-25053.58	1.75	-25023.49	-0.84	-24985.95	4.01	-24968.32	5.31
-25156.61	-3.23	-25120.70	-0.01	-25088.50	-0.48	-25046.38	8.96	-25021.98	0.67	-24986.23	3.74	-24970.28	3.34

-25156.64	-3.26	-25121.82	-1.12	-25086.48	1.53	-25054.56	0.78	-25017.87	4.78	-24982.07	7.89	-24969.75	3.88
-25156.49	-3.11	-25121.28	-0.58	-25089.16	-1.15	-25055.36	-0.03	-25018.14	4.51	-24981.83	8.14	-24969.81	3.82
-25155.64	-2.26	-25123.60	-2.91	-25088.99	-0.97	-25048.56	6.77	-25020.42	2.23	-24980.04	9.93	-24964.52	9.11
-25156.84	-3.46	-25122.94	-2.24	-25087.17	0.85	-25054.12	1.22	-25019.03	3.62	-24988.25	1.72	-24967.32	6.31
-25155.81	-2.43	-25120.25	0.45	-25086.96	1.06	-25050.82	4.51	-25020.10	2.56	-24984.91	5.06	-24971.00	2.63
-25158.06	-4.68	-25123.18	-2.48	-25088.51	-0.49	-25053.91	1.42	-25019.86	2.79	-24986.75	3.22	-24968.36	5.27
-25155.80	-2.42	-25120.18	0.52	-25090.14	-2.12	-25053.38	1.95	-25018.41	4.24	-24988.51	1.45	-24968.92	4.70
-25155.39	-2.02	-25123.70	-3.00	-25086.88	1.13	-25054.55	0.78	-25020.96	1.69	-24983.20	6.77	-24970.15	3.48
-25155.93	-2.55	-25123.34	-2.64	-25084.20	3.82	-25051.16	4.17	-25021.72	0.93	-24988.27	1.69	-24966.36	7.27
-25156.94	-3.57	-25122.71	-2.02	-25085.22	2.79	-25053.87	1.47	-25020.55	2.10	-24984.96	5.01	-24965.26	8.37
-25157.78	-4.40	-25122.18	-1.49	-25087.71	0.31	-25053.96	1.37	-25019.19	3.46	-24979.39	10.58	-24968.19	5.44
-25157.06	-3.68	-25120.69	0.01	-25085.07	2.95	-25049.93	5.40	-25019.09	3.56	-24985.02	4.95	-24968.64	4.99
-25156.99	-3.61	-25122.34	-1.64	-25086.89	1.13	-25055.93	-0.60	-25019.63	3.02	-24987.10	2.87	-24967.47	6.16
-25156.43	-3.05	-25120.22	0.48	-25086.44	1.58	-25053.98	1.35	-25017.46	5.19	-24985.88	4.09	-24967.81	5.82
-25156.16	-2.78	-25121.72	-1.02	-25089.05	-1.04	-25054.55	0.78	-25022.30	0.35	-24984.98	4.99	-24966.96	6.66
-25157.05	-3.67	-25120.12	0.58	-25086.65	1.36	-25053.32	2.02	-25018.62	4.03	-24984.70	5.27	-24967.28	6.35
-25156.71	-3.33	-25121.79	-1.09	-25086.96	1.05	-25052.77	2.56	-25019.37	3.29	-24986.45	3.52	-24970.48	3.14
-25156.32	-2.94	-25122.52	-1.82	-25087.43	0.59	-25054.25	1.09	-25021.06	1.59	-24983.46	6.51	-24967.44	6.19
-25156.71	-3.34	-25120.78	-0.08	-25088.49	-0.48	-25053.56	1.77	-25020.39	2.26	-24985.71	4.26	-24963.45	10.18
-25155.99	-2.61	-25123.44	-2.74	-25086.96	1.05	-25056.33	-1.00	-25017.10	5.55	-24986.80	3.17	-24971.01	2.62
-25157.89	-4.51	-25122.41	-1.72	-25087.68	0.34	-25051.64	3.69	-25010.14	12.51	-24985.25	4.72	-24965.88	7.74
-25156.72	-3.34	-25123.73	-3.03	-25089.36	-1.35	-25053.20	2.13	-25018.72	3.93	-24985.13	4.84	-24971.71	1.92
-25157.10	-3.72	-25122.23	-1.53	-25087.33	0.69	-25053.13	2.21	-25015.66	6.99	-24984.27	5.70	-24964.46	9.16
-25156.36	-2.98	-25122.33	-1.63	-25089.95	-1.93	-25047.48	7.86	-25019.72	2.93	-24986.33	3.64	-24961.88	11.75
-25157.62	-4.24	-25121.22	-0.52	-25089.98	-1.97	-25055.12	0.21	-25020.11	2.54	-24983.41	6.56	-24964.56	9.07
-25156.88	-3.50	-25123.37	-2.67	-25090.08	-2.06	-25053.63	1.70	-25018.41	4.24	-24985.93	4.04	-24967.30	6.32
-25156.49	-3.11	-25123.04	-2.35	-25088.42	-0.41	-25054.09	1.25	-25020.21	2.44	-24984.66	5.31	-24967.23	6.40
-25156.06	-2.68	-25123.03	-2.33	-25088.98	-0.96	-25049.94	5.39	-25020.29	2.36	-24988.48	1.49	-24965.59	8.04
-25156.69	-3.31	-25121.38	-0.69	-25083.61	4.40	-25049.24	6.10	-25022.79	-0.14	-24984.94	5.02	-24968.42	5.20
-25155.27	-1.89	-25122.45	-1.76	-25088.41	-0.40	-25055.10	0.24	-25018.73	3.92	-24986.51	3.45	-24966.99	6.64
-25156.58	-3.20	-25123.26	-2.56	-25088.92	-0.90	-25053.13	2.21	-25019.01	3.64	-24987.08	2.89	-24971.13	2.50
-25157.00	-3.62	-25123.25	-2.55	-25087.68	0.34	-25051.87	3.47	-25017.19	5.46	-24979.74	10.23	-24967.74	5.89
-25156.93	-3.55	-25123.16	-2.46	-25083.22	4.79	-25048.40	6.93	-25017.51	5.14	-24986.42	3.55	-24968.19	5.43
-25156.45	-3.07	-25122.72	-2.02	-25089.44	-1.42	-25053.95	1.39	-25020.65	2.00	-24985.35	4.62	-24969.31	4.31
-25157.07	-3.69	-25122.66	-1.96	-25087.12	0.90	-25051.75	3.58	-25020.01	2.65	-24984.19	5.78	-24969.80	3.83
-25156.83	-3.45	-25120.91	-0.22	-25086.60	1.42	-25051.50	3.83	-25018.25	4.40	-24983.81	6.16	-24965.55	8.08
-25157.85	-4.47	-25122.07	-1.37	-25089.06	-1.04	-25053.66	1.68	-25017.39	5.26	-24985.03	4.94	-24969.13	4.50
-25157.17	-3.79	-25122.89	-2.19	-25086.87	1.15	-25056.10	-0.76	-25017.24	5.41	-24988.35	1.61	-24960.14	13.49
-25156.47	-3.09	-25121.57	-0.87	-25089.27	-1.26	-25052.87	2.47	-25016.33	6.32	-24981.92	8.05	-24967.97	5.65
-25157.56	-4.18	-25122.57	-1.87	-25087.84	0.18	-25052.31	3.03	-25017.76	4.89	-24985.08	4.89	-24968.44	5.19
-25157.20	-3.82	-25121.23	-0.53	-25086.21	1.80	-25052.82	2.51	-25013.06	9.59	-24985.97	4.00	-24963.81	9.82

	-25157.36	-3.98	-25122.55	-1.85	-25087.99	0.02	-25056.63	-1.30	-25019.52	3.13	-24989.14	0.83	-24963.47	10.15
	-25156.77	-3.39	-25121.55	-0.85	-25088.11	-0.09	-25053.41	1.92	-25018.81	3.84	-24983.87	6.10	-24968.58	5.05
	-25154.88	-1.50	-25121.99	-1.29	-25086.71	1.30	-25055.71	-0.38	-25017.92	4.73	-24987.49	2.48	-24968.66	4.97
	-25156.09	-2.71	-25120.29	0.40	-25089.43	-1.41	-25055.35	-0.02	-25018.82	3.83	-24978.35	11.62	-24969.74	3.89
	-25155.90	-2.52	-25121.01	-0.32	-25087.61	0.41	-25052.25	3.08	-25015.89	6.76	-24981.49	8.48	-24967.92	5.71
	-25156.18	-2.80	-25122.62	-1.92	-25089.57	-1.56	-25053.66	1.67	-25020.91	1.74	-24985.05	4.92	-24967.65	5.97
	-25157.11	-3.73	-25122.41	-1.72	-25087.42	0.59					-24983.42	6.55	-24968.34	5.28
	-25154.57	-1.19												
Average energies	-25156.58	-3.20	-25122.06	-1.36	-25087.56	0.46	-25053.02	2.31	-25018.74	3.91	-24984.96	5.01	-24967.54	6.09

Pu

	ground state energy	solid solution energy												
concentration in solid solution (%)	3		6		9		12		15		18		21	
number of atoms	2		4		6		8		10		12		13	
	-25152.06	-4.67	-25110.35	-1.63	-25069.96	0.10	-25029.45	1.94	-24990.22	2.51	-24950.35	3.70	-24930.27	4.46
	-25150.77	-3.38	-25109.29	-0.56	-25070.84	-0.78	-25032.65	-1.25	-24989.59	3.13	-24948.18	5.88	-24922.05	12.67
	-25148.77	-1.38	-25110.75	-2.02	-25066.49	3.56	-25028.58	2.81	-24990.96	1.76	-24951.84	2.22	-24931.09	3.63
	-25150.74	-3.34	-25110.45	-1.73	-25071.56	-1.50	-25032.91	-1.52	-24990.76	1.96	-24954.83	-0.77	-24930.86	3.86
	-25151.07	-3.67	-25110.56	-1.83	-25071.18	-1.12	-25028.45	2.94	-24988.71	4.02	-24947.56	6.49	-24930.84	3.88
	-25151.28	-3.88	-25110.82	-2.09	-25068.13	1.93	-25029.55	1.84	-24992.21	0.51	-24951.71	2.35	-24926.48	8.25
	-25151.61	-4.21	-25110.30	-1.58	-25067.85	2.21	-25027.52	3.87	-24988.45	4.28	-24948.98	5.08	-24930.42	4.31
	-25151.33	-3.94	-25110.60	-1.87	-25070.93	-0.87	-25030.78	0.61	-24992.69	0.03	-24946.45	7.61	-24929.79	4.93
	-25150.79	-3.40	-25110.36	-1.64	-25071.82	-1.76	-25029.21	2.18	-24991.41	1.31	-24951.63	2.43	-24933.32	1.40
	-25151.08	-3.69	-25110.74	-2.01	-25070.65	-0.59	-25030.24	1.15	-24990.93	1.80	-24944.31	9.75	-24933.30	1.43
	-25152.79	-5.40	-25110.59	-1.86	-25069.00	1.06	-25031.21	0.18	-24992.11	0.62	-24947.85	6.20	-24932.06	2.66
	-25150.57	-3.17	-25111.09	-2.36	-25068.26	1.80	-25028.73	2.66	-24989.73	2.99	-24946.90	7.15	-24928.81	5.91
	-25148.09	-0.70	-25108.80	-0.07	-25070.86	-0.80	-25029.69	1.70	-24989.77	2.95	-24950.79	3.27	-24930.54	4.18
	-25151.58	-4.18	-25110.29	-1.56	-25066.98	3.08	-25030.86	0.53	-24989.15	3.57	-24948.88	5.17	-24927.90	6.82
	-25151.30	-3.91	-25109.91	-1.18	-25069.31	0.74	-25032.25	-0.86	-24989.40	3.33	-24949.18	4.87	-24928.81	5.91
	-25151.83	-4.43	-25111.03	-2.31	-25070.55	-0.49	-25029.62	1.77	-24989.55	3.18	-24952.52	1.54	-24930.75	3.98
	-25151.56	-4.17	-25109.27	-0.54	-25071.57	-1.51	-25029.90	1.49	-24990.40	2.33	-24946.26	7.79	-24929.86	4.86
	-25150.73	-3.34	-25108.87	-0.14	-25070.87	-0.81	-25028.25	3.14	-24989.62	3.10	-24950.45	3.60	-24931.80	2.93
	-25151.98	-4.58	-25110.98	-2.26	-25068.56	1.50	-25027.04	4.35	-24990.75	1.98	-24951.34	2.72	-24930.40	4.32
	-25151.08	-3.69	-25110.84	-2.12	-25071.33	-1.27	-25030.90	0.49	-24989.68	3.05	-24953.44	0.61	-24930.33	4.40
	-25150.42	-3.03	-25107.83	0.89	-25069.35	0.71	-25030.69	0.70	-24989.10	3.63	-24951.34	2.71	-24927.82	6.90
	-25150.13	-2.74	-25111.06	-2.33	-25071.89	-1.83	-25030.88	0.51	-24990.06	2.66	-24954.71	-0.65	-24925.40	9.32
	-25151.53	-4.13	-25111.67	-2.95	-25070.62	-0.56	-25031.09	0.31	-24989.18	3.54	-24948.15	5.91	-24932.77	1.95
	-25150.74	-3.35	-25109.92	-1.19	-25071.64	-1.58	-25031.15	0.25	-24989.81	2.91	-24952.05	2.01	-24929.35	5.37

-25150.75	-3.35	-25111.02	-2.29	-25069.94	0.12	-25030.59	0.80	-24990.38	2.35	-24947.34	6.71	-24929.70	5.02	
-25151.20	-3.80	-25109.92	-1.19	-25070.73	-0.67	-25031.15	0.24	-24991.84	0.88	-24950.68	3.38	-24929.21	5.52	
-25151.44	-4.04	-25110.23	-1.50	-25070.30	-0.24	-25029.81	1.58	-24993.30	-0.58	-24949.25	4.80	-24933.80	0.92	
-25151.62	-4.23	-25110.66	-1.93	-25068.85	1.21	-25027.56	3.83	-24990.45	2.27	-24949.59	4.46	-24931.85	2.87	
-25151.25	-3.86	-25110.61	-1.88	-25069.26	0.80	-25028.88	2.51	-24990.39	2.34	-24948.94	5.12	-24932.20	2.52	
-25150.40	-3.01	-25112.03	-3.30	-25071.66	-1.60	-25030.41	0.98	-24988.92	3.80	-24948.10	5.95	-24932.06	2.66	
-25149.74	-2.34	-25110.37	-1.65	-25069.08	0.98	-25028.00	3.39	-24990.41	2.31	-24947.89	6.16	-24928.80	5.92	
-25151.68	-4.29	-25109.63	-0.91	-25070.42	-0.36	-25032.64	-1.25	-24991.48	1.25	-24951.07	2.99	-24931.76	2.96	
-25150.28	-2.88	-25112.16	-3.43	-25071.63	-1.57	-25030.91	0.48	-24987.78	4.94	-24947.95	6.11	-24930.42	4.30	
-25150.00	-2.60	-25110.64	-1.91	-25069.22	0.84	-25029.44	1.95	-24992.26	0.47	-24951.80	2.26	-24925.34	9.38	
-25150.26	-2.86	-25110.83	-2.11	-25068.69	1.37	-25026.99	4.40	-24988.53	4.20	-24952.62	1.43	-24929.64	5.08	
-25150.84	-3.45	-25109.89	-1.17	-25071.12	-1.06	-25028.87	2.52	-24989.96	2.76	-24948.57	5.49	-24931.55	3.17	
-25151.31	-3.92	-25111.37	-2.65	-25070.91	-0.85	-25030.15	1.24	-24991.05	1.67	-24951.50	2.55	-24929.94	4.78	
-25150.86	-3.46	-25108.04	0.69	-25071.57	-1.51	-25030.56	0.84	-24989.80	2.92	-24949.63	4.43	-24927.79	6.93	
-25151.09	-3.70	-25110.13	-1.40	-25069.25	0.81	-25025.60	5.79	-24993.30	-0.58	-24949.99	4.07	-24931.49	3.23	
-25150.53	-3.13	-25110.39	-1.67	-25071.81	-1.75	-25030.96	0.43	-24987.49	5.24	-24951.21	2.84	-24929.52	5.21	
-25149.73	-2.33	-25110.37	-1.65	-25071.82	-1.76	-25029.66	1.73	-24989.89	2.84	-24949.61	4.45	-24931.69	3.03	
-25151.21	-3.81	-25110.61	-1.88	-25068.71	1.35	-25027.65	3.74	-24991.23	1.49	-24949.08	4.98	-24932.95	1.78	
-25151.30	-3.90	-25109.95	-1.22	-25068.17	1.89	-25027.09	4.30	-24991.83	0.89	-24949.75	4.30	-24926.51	8.22	
-25150.94	-3.54	-25110.20	-1.47	-25068.75	1.31	-25031.20	0.19	-24988.40	4.32	-24948.08	5.97	-24928.65	6.07	
-25151.65	-4.25	-25109.09	-0.36	-25070.60	-0.54	-25032.26	-0.86	-24989.80	2.92	-24945.53	8.53	-24930.41	4.32	
-25151.13	-3.73	-25112.27	-3.54	-25070.91	-0.85	-25030.20	1.19	-24991.44	1.28	-24951.06	2.99	-24928.87	5.85	
-25150.67	-3.28	-25112.22	-3.49	-25068.66	1.40	-25028.33	3.07	-24989.16	3.56	-24949.85	4.21	-24932.73	1.99	
-25152.68	-5.28	-25108.41	0.31	-25070.09	-0.03	-25029.31	2.08	-24991.62	1.11	-24950.18	3.88	-24931.21	3.51	
-25151.46	-4.06	-25109.63	-0.91	-25070.58	-0.52	-25033.04	-1.65	-24989.51	3.22	-24949.66	4.40	-24930.01	4.71	
-25152.07	-4.68	-25108.14	0.59	-25070.95	-0.89	-25031.19	0.20	-24988.21	4.51	-24953.77	0.29	-24927.95	6.78	
-25151.42	-4.03	-25109.90	-1.18	-25067.37	2.69	-25030.80	0.59	-24991.73	0.99	-24950.62	3.43	-24931.11	3.61	
-25151.21	-3.81	-25111.80	-3.08	-25066.09	3.97	-25030.20	1.19	-24991.82	0.91	-24945.94	8.12	-24932.63	2.10	
-25151.77	-4.37	-25109.29	-0.56	-25069.12	0.94	-25031.35	0.04	-24991.30	1.42	-24949.81	4.24	-24931.88	2.84	
-25149.76	-2.36	-25109.34	-0.61	-25070.08	-0.02	-25024.12	7.28	-24990.49	2.23	-24948.61	5.44	-24930.58	4.14	
-25150.51	-3.12	-25110.93	-2.21	-25072.69	-2.63	-25030.73	0.66	-24990.84	1.88	-24945.82	8.24	-24930.63	4.09	
-25150.71	-3.32	-25109.25	-0.52	-25070.41	-0.36	-25029.70	1.70	-24991.25	1.48	-24950.58	3.48	-24925.04	9.68	
-25150.74	-3.34	-25110.68	-1.95	-25072.51	-2.45	-25029.24	2.15	-24989.49	3.23	-24950.01	4.04	-24929.36	5.36	
-25149.81	-2.41	-25110.64	-1.91	-25072.00	-1.94	-25030.36	1.03	-24991.55	1.18	-24949.05	5.01	-24930.48	4.24	
-25151.13	-3.74	-25110.24	-1.52	-25069.17	0.89	-25031.22	0.17	-24990.49	2.24	-24950.07	3.98	-24930.76	3.97	
-25150.68	-3.28	-25109.30	-0.57	-25071.91	-1.85	-25031.01	0.38	-24992.62	0.11	-24950.38	3.68	-24929.03	5.69	
-25151.61	-4.21	-25111.25	-2.53	-25070.79	-0.73	-25025.52	5.87	-24987.23	5.50	-24949.86	4.19	-24930.35	4.37	
-25150.33	-2.94	-25107.76	0.97	-25069.80	0.26	-25028.00	3.39	-24993.44	-0.72	-24951.61	2.44	-24928.94	5.78	
-25151.24	-3.85	-25111.29	-2.57	-25070.48	-0.42	-25029.59	1.80	-24989.21	3.52	-24952.90	1.16	-24931.02	3.70	
-25150.76	-3.36	-25111.50	-2.78	-25072.06	-2.00	-25029.97	1.42	-24991.44	1.29	-24949.76	4.29	-24929.77	4.95	
-25151.25	-3.86	-25110.99	-2.27	-25069.72	0.34	-25031.34	0.05	-24992.03	0.69	-24951.07	2.99	-24932.52	2.20	

-25150.80	-3.41	-25109.30	-0.58	-25070.42	-0.36	-25029.69	1.70	-24993.03	-0.30	-24949.32	4.74	-24930.87	3.85
-25150.67	-3.27	-25110.77	-2.04	-25069.73	0.33	-25030.74	0.66	-24991.93	0.79	-24950.17	3.88	-24931.08	3.64
-25150.59	-3.19	-25111.33	-2.60	-25069.48	0.57	-25030.83	0.56	-24990.41	2.31	-24946.65	7.40	-24930.21	4.51
-25151.59	-4.20	-25110.90	-2.18	-25070.45	-0.39	-25031.37	0.02	-24988.73	3.99	-24952.56	1.50	-24927.03	7.69
-25149.56	-2.17	-25110.68	-1.96	-25067.57	2.48	-25028.88	2.51	-24992.49	0.23	-24951.71	2.35	-24929.30	5.42
-25149.30	-1.90	-25109.81	-1.08	-25070.98	-0.92	-25032.73	-1.34	-24989.27	3.45	-24952.24	1.81	-24929.59	5.13
-25150.72	-3.33	-25110.76	-2.03	-25070.11	-0.05	-25025.80	5.59	-24988.53	4.19	-24949.48	4.58	-24928.15	6.57
-25149.99	-2.60	-25108.86	-0.13	-25068.29	1.77	-25032.46	-1.07	-24991.11	1.62	-24951.12	2.94	-24930.76	3.96
-25151.30	-3.91	-25111.51	-2.79	-25068.32	1.74	-25030.33	1.06	-24985.16	7.57	-24950.42	3.63	-24930.82	3.91
-25150.08	-2.69	-25109.62	-0.90	-25070.36	-0.30	-25030.31	1.08	-24989.25	3.47	-24947.46	6.60	-24927.77	6.95
-25151.22	-3.83	-25110.62	-1.90	-25070.69	-0.63	-25027.06	4.33	-24987.80	4.92	-24948.20	5.86	-24932.18	2.54
-25151.44	-4.04	-25108.83	-0.10	-25072.09	-2.03	-25029.73	1.66	-24992.42	0.30	-24947.49	6.57	-24934.27	0.45
-25150.35	-2.96	-25111.65	-2.92	-25069.98	0.08	-25033.46	-2.06	-24993.42	-0.70	-24948.08	5.98	-24929.92	4.80
-25150.80	-3.41	-25111.09	-2.37	-25069.39	0.67	-25030.37	1.02	-24988.42	4.31	-24950.51	3.55	-24933.07	1.65
-25149.88	-2.49	-25109.17	-0.44	-25071.65	-1.59	-25033.42	-2.03	-24985.91	6.82	-24952.70	1.36	-24930.62	4.10
-25150.88	-3.49	-25110.58	-1.86	-25071.58	-1.53	-25031.09	0.30	-24986.86	5.87	-24947.96	6.09	-24928.63	6.10
-25152.21	-4.82	-25110.48	-1.76	-25070.85	-0.79	-25031.21	0.18	-24990.47	2.25	-24951.03	3.03	-24931.15	3.57
-25150.58	-3.18	-25109.98	-1.25	-25071.15	-1.09	-25028.49	2.90	-24991.89	0.83	-24951.38	2.68	-24926.40	8.32
-25150.41	-3.01	-25109.53	-0.81	-25071.01	-0.95	-25032.19	-0.80	-24991.69	1.04	-24953.39	0.67	-24931.80	2.92
-25151.62	-4.23	-25111.19	-2.47	-25069.46	0.60	-25029.36	2.03	-24990.48	2.24	-24951.22	2.84	-24929.66	5.06
-25149.98	-2.58	-25110.06	-1.33	-25067.62	2.44	-25032.41	-1.02	-24988.86	3.86	-24951.20	2.86	-24928.75	5.97
-25150.20	-2.80	-25108.55	0.18	-25070.31	-0.25	-25027.80	3.59	-24987.50	5.22	-24952.17	1.88	-24933.29	1.43
-25150.63	-3.23	-25109.15	-0.43	-25070.22	-0.16	-25030.49	0.90	-24992.26	0.47	-24951.73	2.32	-24932.82	1.91
-25150.73	-3.34	-25111.60	-2.87	-25071.25	-1.19	-25031.10	0.29	-24990.96	1.77	-24951.90	2.16	-24931.98	2.74
-25151.35	-3.96	-25111.69	-2.97	-25070.11	-0.05	-25027.86	3.53	-24991.35	1.38	-24947.55	6.50	-24930.47	4.26
-25151.52	-4.13	-25110.71	-1.98	-25071.76	-1.70	-25029.68	1.71	-24991.00	1.72	-24946.23	7.83	-24930.61	4.11
-25150.31	-2.91	-25110.03	-1.30	-25069.40	0.66	-25030.93	0.46	-24990.24	2.49	-24951.64	2.41	-24925.52	9.20
-25150.61	-3.22	-25109.55	-0.83	-25069.51	0.55	-25031.12	0.28	-24991.80	0.92	-24952.35	1.70	-24931.44	3.28
-25150.29	-2.90	-25110.36	-1.64	-25070.99	-0.93	-25025.36	6.03	-24990.64	2.08	-24952.32	1.74	-24931.58	3.14
-25150.92	-3.52	-25109.50	-0.77	-25071.46	-1.40	-25029.31	2.08	-24991.96	0.76	-24951.18	2.87	-24932.80	1.92
-25151.18	-3.79	-25079.92	28.81	-25070.45	-0.39	-25025.16	6.23	-24991.42	1.30	-24952.34	1.71	-24931.20	3.52
-25150.56	-3.16	-25112.06	-3.33	-25070.65	-0.59	-25029.93	1.46	-24986.72	6.00	-24952.08	1.97	-24929.68	5.05
-25151.35	-3.96	-25111.50	-2.78	-25070.53	-0.47	-25032.15	-0.76	-24992.89	-0.17	-24948.88	5.18	-24931.60	3.13
-25148.84	-1.44	-25112.33	-3.60	-25072.54	-2.48	-25031.53	-0.14	-24991.16	1.57	-24950.34	3.72	-24934.47	0.25
-25149.63	-2.24	-25110.38	-1.65	-25070.35	-0.29	-25032.08	-0.69	-24993.61	-0.89	-24953.19	0.86	-24931.08	3.65
-25150.32	-2.93	-25111.99	-3.26	-25068.30	1.76	-25027.81	3.58	-24993.02	-0.30	-24946.62	7.43	-24929.74	4.98
-25150.90	-3.51	-25111.57	-2.84	-25063.47	6.58	-25030.17	1.22	-24990.28	2.45	-24953.17	0.89	-24931.01	3.71
-25150.04	-2.64	-25110.03	-1.31	-25071.94	-1.88	-25030.76	0.63	-24990.44	2.28	-24949.25	4.81	-24930.06	4.66
-25150.77	-3.38	-25109.88	-1.16	-25070.39	-0.34	-25031.06	0.33	-24990.67	2.05	-24950.51	3.54	-24931.85	2.87
-25150.72	-3.32	-25109.76	-1.03	-25071.33	-1.27	-25029.42	1.97	-24990.44	2.28	-24950.89	3.17	-24931.19	3.53
-25150.94	-3.55	-25110.39	-1.66	-25071.42	-1.36	-25031.55	-0.16	-24990.92	1.81	-24948.64	5.41	-24928.54	6.18

	-25151.70	-4.31	-25108.50	0.23	-25072.67	-2.61	-25032.19	-0.80	-24990.92	1.81	-24952.01	2.05	-24933.30	1.43
	-25150.74	-3.34	-25109.26	-0.53	-25072.24	-2.18	-25032.35	-0.96	-24989.94	2.78	-24951.98	2.08	-24929.37	5.35
	-25151.33	-3.93	-25110.60	-1.88	-25070.78	-0.72	-25031.63	-0.24	-24990.52	2.21	-24950.09	3.97	-24928.72	6.01
	-25150.96	-3.56	-25106.55	2.18	-25067.96	2.10	-25031.67	-0.28	-24988.39	4.34	-24950.96	3.10	-24931.70	3.02
	-25151.14	-3.75	-25109.29	-0.56	-25070.94	-0.88	-25027.66	3.73	-24991.36	1.36	-24949.18	4.88	-24933.73	1.00
	-25150.68	-3.29	-25108.74	-0.01	-25073.28	-3.22	-25028.47	2.92	-24988.61	4.11	-24949.92	4.14	-24930.25	4.47
	-25150.13	-2.73	-25112.15	-3.42	-25070.71	-0.66	-25031.65	-0.26	-24992.88	-0.15	-24951.19	2.87	-24929.82	4.90
	-25151.17	-3.78	-25109.67	-0.94	-25071.62	-1.56	-25028.18	3.21	-24992.26	0.47	-24948.15	5.90	-24931.39	3.33
	-25150.94	-3.55	-25110.44	-1.71	-25067.18	2.87	-25029.84	1.55	-24990.93	1.79	-24947.09	6.97	-24932.46	2.26
	-25149.68	-2.29	-25111.10	-2.37	-25065.87	4.19							-24930.33	4.39
	-25150.47	-3.08	-25107.80	0.93	-25069.22	0.83								
	-25149.77	-2.38			-25071.29	-1.23								
	-25150.56	-3.17			-25071.18	-1.12								
Average energies	-25150.82	-3.43	-25110.01	-1.28	-25070.17	-0.11	-25029.98	1.41	-24990.44	2.28	-24950.06	4.00	-24930.34	4.38

Th

	ground state energy	solid solution energy												
concentration in solid solution (%)	3		6		9		12		15		18		21	
number of atoms	2		4		6		8		10		12		13	
	-25156.46	-3.44	-25121.66	-1.67	-25085.00	1.95	-25051.18	2.74	-25015.40	5.48	-24981.67	6.17	-24962.28	9.04
	-25156.19	-3.17	-25122.22	-2.23	-25088.31	-1.36	-25050.99	2.92	-25016.63	4.24	-24982.87	4.97	-24966.85	4.47
	-25155.36	-2.34	-25122.97	-2.98	-25089.21	-2.26	-25052.58	1.33	-25020.55	0.33	-24983.85	3.99	-24963.72	7.60
	-25156.45	-3.42	-25121.60	-1.61	-25088.62	-1.67	-25046.82	7.10	-25014.81	6.06	-24984.85	2.99	-24966.39	4.93
	-25157.52	-4.49	-25120.55	-0.56	-25086.19	0.76	-25048.90	5.02	-25013.64	7.24	-24985.12	2.72	-24962.82	8.50
	-25156.79	-3.76	-25121.15	-1.17	-25086.37	0.58	-25053.63	0.29	-25015.07	5.80	-24977.30	10.54	-24964.21	7.11
	-25156.31	-3.29	-25121.61	-1.63	-25086.03	0.92	-25052.75	1.17	-25018.97	1.91	-24983.37	4.47	-24969.17	2.15
	-25156.51	-3.48	-25122.02	-2.03	-25087.39	-0.44	-25055.87	-1.95	-25014.81	6.06	-24986.39	1.45	-24966.93	4.39
	-25156.32	-3.30	-25121.38	-1.39	-25085.85	1.10	-25055.46	-1.54	-25018.14	2.73	-24985.85	1.99	-24966.05	5.27
	-25156.32	-3.30	-25119.20	0.79	-25087.24	-0.29	-25046.21	7.71	-25017.98	2.90	-24983.66	4.18	-24967.41	3.91
	-25156.45	-3.42	-25121.67	-1.68	-25088.11	-1.16	-25050.21	3.70	-25019.41	1.46	-24985.65	2.19	-24963.36	7.96
	-25155.89	-2.87	-25124.17	-4.18	-25088.50	-1.55	-25052.97	0.94	-25021.28	-0.41	-24981.79	6.05	-24965.20	6.12
	-25156.24	-3.21	-25123.51	-3.52	-25088.56	-1.60	-25050.34	3.57	-25015.49	5.38	-24981.37	6.47	-24960.81	10.51
	-25157.40	-4.38	-25122.67	-2.68	-25087.82	-0.87	-25049.45	4.46	-25020.29	0.59	-24980.57	7.27	-24958.31	13.01
	-25155.90	-2.88	-25121.77	-1.78	-25089.40	-2.45	-25054.29	-0.38	-25019.07	1.81	-24985.24	2.60	-24971.71	-0.39
	-25156.46	-3.44	-25122.78	-2.79	-25087.96	-1.01	-25053.85	0.06	-25018.42	2.46	-24984.94	2.89	-24966.00	5.32
	-25155.31	-2.29	-25121.44	-1.45	-25085.16	1.79	-25052.30	1.61	-25016.67	4.20	-24983.49	4.35	-24968.84	2.48
	-25157.38	-4.35	-25120.52	-0.53	-25087.43	-0.48	-25047.83	6.08	-25014.97	5.91	-24984.56	3.28	-24966.82	4.50
	-25156.73	-3.70	-25120.78	-0.79	-25087.16	-0.20	-25049.33	4.59	-25012.34	8.53	-24984.19	3.65	-24964.33	6.99

-25156.79	-3.77	-25118.17	1.81	-25087.81	-0.86	-25052.51	1.41	-25014.19	6.69	-24986.25	1.59	-24965.29	6.03
-25154.71	-1.68	-25121.97	-1.98	-25089.53	-2.58	-25054.03	-0.12	-25017.99	2.89	-24980.20	7.64	-24967.25	4.07
-25156.28	-3.26	-25123.16	-3.18	-25086.11	0.84	-25050.76	3.16	-25016.96	3.91	-24977.17	10.67	-24964.54	6.78
-25155.57	-2.55	-25122.42	-2.44	-25088.38	-1.43	-25052.72	1.20	-25018.49	2.39	-24984.98	2.86	-24967.76	3.56
-25156.53	-3.50	-25119.02	0.97	-25085.23	1.72	-25043.56	10.35	-25015.04	5.83	-24984.09	3.75	-24965.81	5.51
-25156.09	-3.07	-25121.16	-1.17	-25083.02	3.93	-25052.72	1.20	-25016.06	4.82	-24984.19	3.65	-24963.69	7.63
-25155.59	-2.57	-25120.64	-0.65	-25087.72	-0.77	-25050.45	3.46	-25020.54	0.33	-24987.13	0.70	-24966.49	4.83
-25156.74	-3.71	-25122.43	-2.44	-25087.33	-0.38	-25049.63	4.29	-25021.49	-0.61	-24984.77	3.07	-24965.11	6.21
-25156.89	-3.87	-25123.52	-3.53	-25085.74	1.21	-25051.08	2.83	-25019.05	1.82	-24986.30	1.54	-24952.22	19.10
-25156.31	-3.28	-25118.21	1.78	-25087.36	-0.41	-25052.34	1.58	-25018.20	2.67	-24983.92	3.92	-24958.49	12.83
-25156.71	-3.68	-25119.60	0.39	-25087.93	-0.98	-25051.28	2.63	-25015.53	5.35	-24986.84	1.00	-24966.29	5.03
-25156.22	-3.20	-25121.50	-1.52	-25086.02	0.93	-25053.18	0.74	-25018.79	2.09	-24984.79	3.05	-24969.24	2.07
-25156.77	-3.75	-25120.41	-0.43	-25084.05	2.91	-25053.60	0.31	-25012.87	8.00	-24981.54	6.29	-24966.73	4.59
-25155.68	-2.66	-25121.44	-1.45	-25085.88	1.07	-25053.02	0.90	-25013.91	6.97	-24983.85	3.99	-24966.79	4.53
-25157.43	-4.41	-25120.38	-0.39	-25087.53	-0.58	-25052.78	1.14	-25019.24	1.63	-24983.06	4.78	-24953.44	17.88
-25156.59	-3.57	-25122.47	-2.48	-25086.83	0.12	-25046.30	7.61	-25016.15	4.73	-24979.67	8.17	-24966.64	4.68
-25157.41	-4.38	-25120.74	-0.76	-25085.03	1.92	-25052.06	1.85	-25019.04	1.83	-24983.35	4.49	-24968.09	3.23
-25155.85	-2.82	-25119.87	0.11	-25087.82	-0.87	-25052.62	1.29	-25014.47	6.41	-24985.28	2.56	-24964.43	6.89
-25157.43	-4.41	-25121.12	-1.14	-25088.59	-1.64	-25050.83	3.09	-25014.22	6.66	-24983.55	4.29	-24960.99	10.33
-25156.05	-3.03	-25122.08	-2.09	-25087.06	-0.11	-25056.00	-2.09	-25016.20	4.68	-24978.16	9.68	-24960.06	11.26
-25156.73	-3.71	-25121.50	-1.51	-25087.49	-0.54	-25053.17	0.74	-25014.44	6.43	-24984.62	3.22	-24966.53	4.79
-25156.39	-3.37	-25122.64	-2.65	-25087.36	-0.41	-25051.13	2.78	-25014.84	6.04	-24982.85	4.99	-24961.63	9.69
-25156.58	-3.55	-25121.26	-1.27	-25085.95	1.00	-25054.61	-0.69	-25018.96	1.91	-24983.77	4.07	-24963.07	8.25
-25156.80	-3.78	-25123.04	-3.05	-25084.37	2.58	-25052.24	1.67	-25021.35	-0.48	-24982.58	5.26	-24966.39	4.93
-25158.01	-4.99	-25122.09	-2.10	-25086.23	0.72	-25048.23	5.68	-25018.85	2.03	-24984.09	3.75	-24965.55	5.77
-25156.75	-3.73	-25120.84	-0.85	-25088.61	-1.66	-25051.35	2.56	-25018.44	2.43	-24978.15	9.69	-24965.61	5.71
-25156.18	-3.15	-25122.22	-2.23	-25083.73	3.22	-25054.18	-0.27	-25018.47	2.41	-24983.10	4.74	-24960.96	10.36
-25156.51	-3.48	-25120.19	-0.20	-25088.14	-1.19	-25053.28	0.63	-25018.10	2.78	-24983.17	4.67	-24964.18	7.14
-25157.24	-4.21	-25120.94	-0.95	-25083.91	3.04	-25049.97	3.94	-25021.65	-0.77	-24975.27	12.57	-24970.02	1.30
-25154.77	-1.74	-25121.81	-1.82	-25085.87	1.08	-25051.65	2.26	-25011.92	8.96	-24977.31	10.53	-24962.81	8.51
-25155.29	-2.26	-25121.95	-1.96	-25087.92	-0.97	-25051.44	2.47	-25013.49	7.39	-24976.43	11.41	-24964.73	6.59
-25156.26	-3.24	-25121.49	-1.50	-25087.57	-0.61	-25049.35	4.57	-25014.91	5.96	-24976.87	10.97	-24965.57	5.75
-25156.44	-3.42	-25123.09	-3.11	-25086.72	0.23	-25051.94	1.97	-25015.08	5.80	-24983.60	4.24	-24966.22	5.10
-25157.23	-4.20	-25119.24	0.75	-25088.77	-1.82	-25052.02	1.89	-25015.45	5.42	-24975.22	12.61	-24966.41	4.91
-25157.56	-4.53	-25120.49	-0.50	-25088.72	-1.77	-25050.49	3.43	-25018.62	2.26	-24980.67	7.16	-24967.99	3.33
-25156.18	-3.16	-25121.36	-1.37	-25083.29	3.66	-25051.59	2.33	-25018.21	2.67	-24979.31	8.52	-24966.98	4.34
-25155.66	-2.63	-25121.85	-1.86	-25088.31	-1.36	-25050.50	3.41	-25018.59	2.28	-24985.62	2.22	-24969.08	2.24
-25155.90	-2.88	-25121.59	-1.61	-25088.41	-1.46	-25051.04	2.88	-25016.52	4.36	-24978.86	8.98	-24964.76	6.56
-25155.60	-2.57	-25121.63	-1.64	-25087.74	-0.79	-25050.14	3.77	-25016.74	4.14	-24982.16	5.67	-24964.51	6.81
-25156.92	-3.90	-25122.41	-2.42	-25087.52	-0.57	-25052.59	1.33	-25020.27	0.60	-24984.24	3.60	-24966.01	5.31
-25156.82	-3.79	-25122.04	-2.05	-25083.01	3.94	-25050.32	3.59	-25021.09	-0.21	-24981.73	6.11	-24965.62	5.70

-25157.41	-4.39	-25122.64	-2.66	-25087.28	-0.33	-25052.43	1.48	-25012.72	8.15	-24980.17	7.67	-24968.50	2.82
-25157.59	-4.57	-25121.60	-1.61	-25087.56	-0.61	-25052.22	1.70	-25017.88	2.99	-24983.53	4.30	-24967.87	3.45
-25155.43	-2.41	-25120.11	-0.12	-25086.04	0.91	-25048.45	5.46	-25002.84	18.04	-24986.42	1.42	-24968.14	3.18
-25155.82	-2.80	-25122.19	-2.20	-25084.48	2.47	-25051.60	2.32	-25018.05	2.82	-24983.88	3.96	-24962.72	8.60
-25157.32	-4.29	-25121.98	-1.99	-25087.93	-0.98	-25051.27	2.64	-25013.86	7.01	-24982.50	5.34	-24964.97	6.35
-25156.44	-3.41	-25120.59	-0.61	-25086.08	0.87	-25052.65	1.26	-25017.82	3.05	-24976.10	11.74	-24965.27	6.05
-25156.05	-3.02	-25121.51	-1.52	-25081.96	4.99	-25052.65	1.27	-25012.53	8.35	-24983.50	4.33	-24968.07	3.25
-25156.67	-3.65	-25122.89	-2.90	-25088.81	-1.86	-25050.93	2.98	-25009.96	10.92	-24985.28	2.56	-24961.71	9.61
-25157.81	-4.78	-25122.34	-2.35	-25083.77	3.18	-25052.24	1.67	-25016.22	4.66	-24981.61	6.23	-24968.41	2.91
-25156.60	-3.58	-25121.47	-1.49	-25087.92	-0.97	-25051.18	2.73	-25016.71	4.16	-24987.00	0.84	-24963.75	7.57
-25157.17	-4.15	-25119.78	0.21	-25087.53	-0.58	-25051.74	2.18	-25021.62	-0.75	-24985.17	2.67	-24968.47	2.85
-25155.48	-2.46	-25120.40	-0.41	-25088.14	-1.19	-25051.99	1.92	-25018.05	2.82	-24986.57	1.27	-24967.07	4.25
-25156.90	-3.88	-25123.57	-3.59	-25085.02	1.93	-25050.38	3.53	-25017.61	3.27	-24984.80	3.04	-24963.53	7.79
-25155.30	-2.28	-25122.48	-2.49	-25087.50	-0.55	-25051.25	2.66	-25019.32	1.55	-24982.89	4.95	-24965.88	5.44
-25155.75	-2.73	-25122.12	-2.13	-25086.18	0.77	-25052.98	0.93	-25016.11	4.76	-24981.90	5.94	-24965.68	5.64
-25156.86	-3.84	-25121.41	-1.42	-25084.96	1.99	-25052.57	1.34	-25014.50	6.37	-24985.82	2.01	-24969.90	1.42
-25155.74	-2.72	-25121.77	-1.78	-25087.64	-0.69	-25050.76	3.16	-25018.95	1.92	-24985.57	2.27	-24965.03	6.29
-25157.54	-4.52	-25121.06	-1.08	-25085.53	1.42	-25050.26	3.65	-25021.79	-0.91	-24978.27	9.57	-24965.40	5.92
-25156.49	-3.46	-25122.54	-2.56	-25086.29	0.66	-25050.55	3.36	-25019.05	1.82	-24985.95	1.89	-24965.17	6.15
-25155.28	-2.26	-25123.71	-3.72	-25085.39	1.56	-25051.71	2.20	-25017.94	2.94	-24982.81	5.03	-24959.07	12.25
-25156.44	-3.42	-25121.19	-1.20	-25087.28	-0.33	-25051.66	2.25	-25021.57	-0.70	-24983.23	4.61	-24965.33	5.99
-25156.80	-3.77	-25121.31	-1.32	-25084.94	2.01	-25052.80	1.12	-25016.33	4.55	-24980.34	7.50	-24964.18	7.14
-25155.45	-2.42	-25122.19	-2.20	-25087.34	-0.39	-25051.85	2.07	-25019.00	1.87	-24986.34	1.50	-24963.98	7.34
-25155.98	-2.95	-25123.38	-3.39	-25089.65	-2.70	-25051.23	2.68	-25020.18	0.69	-24981.94	5.90	-24967.08	4.24
-25155.94	-2.91	-25121.94	-1.96	-25088.12	-1.17	-25046.93	6.98	-25018.40	2.48	-24984.57	3.27	-24965.99	5.33
-25157.80	-4.78	-25122.20	-2.21	-25087.36	-0.41	-25052.03	1.88	-25018.74	2.13	-24985.36	2.48	-24964.22	7.10
-25157.37	-4.35	-25121.85	-1.86	-25089.21	-2.26	-25050.79	3.12	-25012.98	7.89	-24983.04	4.80	-24966.49	4.83
-25156.66	-3.64	-25120.09	-0.10	-25086.18	0.77	-25046.89	7.02	-25015.38	5.50	-24985.12	2.72	-24966.15	5.17
-25156.96	-3.94	-25119.99	0.00	-25084.26	2.69	-25052.31	1.61	-25016.89	3.99	-24982.13	5.71	-24968.81	2.51
-25156.67	-3.65	-25122.76	-2.77	-25086.82	0.13	-25052.81	1.10	-25017.04	3.83	-24982.54	5.30	-24963.03	8.29
-25156.28	-3.26	-25121.83	-1.85	-25085.40	1.55	-25056.15	-2.24	-25012.36	8.52	-24976.54	11.30	-24963.20	8.12
-25155.92	-2.90	-25122.04	-2.05	-25087.37	-0.42	-25050.52	3.40	-25016.60	4.28	-24984.06	3.78	-24967.40	3.92
-25156.47	-3.44	-25123.13	-3.14	-25086.61	0.34	-25051.35	2.57	-25013.24	7.63	-24973.95	13.89	-24966.10	5.22
-25154.68	-1.66	-25121.22	-1.24	-25085.60	1.35	-25053.31	0.60	-25018.87	2.00	-24982.52	5.32	-24962.28	9.04
-25156.39	-3.37	-25122.35	-2.36	-25086.89	0.06	-25048.24	5.67	-25021.26	-0.38	-24984.88	2.96	-24966.53	4.79
-25155.11	-2.09	-25122.01	-2.02	-25085.60	1.35	-25053.79	0.12	-25017.76	3.11	-24983.34	4.49	-24964.80	6.52
-25157.31	-4.29	-25122.02	-2.04	-25084.47	2.48	-25052.40	1.52	-25016.64	4.24	-24978.82	9.02	-24965.66	5.66
-25155.88	-2.85	-25122.39	-2.40	-25086.33	0.62	-25054.47	-0.56	-25019.11	1.76	-24981.33	6.50	-24966.99	4.33
-25155.65	-2.63	-25121.21	-1.23	-25084.80	2.15	-25056.25	-2.33	-25014.72	6.16	-24981.63	6.20	-24963.35	7.97
-25157.04	-4.02	-25122.92	-2.93	-25088.73	-1.78	-25049.90	4.01	-25020.25	0.63	-24986.87	0.97	-24971.93	-0.61
-25155.87	-2.85	-25122.35	-2.36	-25088.64	-1.69	-25050.31	3.60	-25014.83	6.05	-24981.62	6.22	-24967.18	4.14

	-25157.09	-4.07	-25120.81	-0.82	-25088.34	-1.39	-25051.08	2.83	-25013.23	7.64	-24979.43	8.41	-24965.41	5.91
	-25155.68	-2.65	-25120.43	-0.44	-25083.28	3.67	-25053.89	0.02	-25016.77	4.10	-24983.84	3.99	-24964.73	6.59
	-25156.26	-3.24	-25119.79	0.20	-25089.51	-2.56	-25054.02	-0.11	-25013.86	7.02	-24977.75	10.08	-24962.25	9.07
	-25156.86	-3.83	-25121.63	-1.65	-25084.65	2.30	-25051.42	2.49	-25016.13	4.74	-24980.38	7.46	-24959.26	12.06
	-25156.36	-3.34	-25124.14	-4.16	-25086.05	0.90	-25048.07	5.85	-25014.39	6.49	-24983.89	3.95	-24968.35	2.97
	-25156.70	-3.68	-25121.31	-1.32	-25086.80	0.15	-25054.46	-0.55	-25019.41	1.47	-24985.38	2.46	-24971.71	-0.39
	-25157.49	-4.47	-25122.17	-2.18	-25086.51	0.44	-25049.52	4.40	-25015.32	5.56	-24984.66	3.18	-24969.52	1.80
	-25156.68	-3.66	-25122.93	-2.94	-25088.21	-1.26	-25053.59	0.33	-25019.12	1.76	-24985.37	2.47	-24965.05	6.27
	-25155.11	-2.08	-25122.74	-2.76	-25087.09	-0.14	-25049.94	3.97	-25018.52	2.36	-24982.49	5.35	-24963.10	8.22
	-25156.39	-3.36	-25120.54	-0.55	-25086.70	0.25	-25053.36	0.55	-25021.18	-0.30	-24982.90	4.94	-24964.67	6.65
	-25156.47	-3.45	-25123.48	-3.50	-25084.32	2.63	-25052.93	0.98	-25020.16	0.72	-24985.26	2.58	-24958.40	12.92
	-25156.80	-3.77	-25118.08	1.90	-25088.50	-1.55	-25054.93	-1.02	-25020.14	0.74	-24985.15	2.69	-24963.52	7.80
	-25154.89	-1.86	-25123.09	-3.10	-25087.93	-0.98	-25051.05	2.87	-25016.23	4.65	-24985.74	2.10	-24966.30	5.02
	-25156.20	-3.18	-25120.14	-0.15	-25086.61	0.34	-25050.44	3.47	-25018.85	2.02				
	-25156.73	-3.71			-25086.56	0.39	-25052.04	1.87	-25015.38	5.49				
	-25157.12	-4.10					-25053.37	0.54	-25020.17	0.71				
	-25156.12	-3.10							-25016.26	4.61				
Average energies	-25156.41	-3.39	-25121.61	-1.63	-25086.75	0.20	-25051.60	2.31	-25017.03	3.85	-24982.79	5.05	-24965.20	6.12
**U**	ground state energy	solid solution energy												
concentration in solid solution (%)	3		6		9		12		15		18		21	
number of atoms	2		4		6		8		10		12		13	
	-25160.74	-2.70	-25130.52	-0.50	-25093.99	8.02	-25074.93	-0.94	-25043.22	2.75	-25009.59	8.36	-24997.87	6.07
	-25160.50	-2.46	-25132.28	-2.26	-25099.87	2.14	-25069.86	4.13	-25043.14	2.83	-25017.44	0.51	-25000.44	3.50
	-25161.52	-3.48	-25129.32	0.71	-25100.91	1.09	-25071.52	2.47	-25044.68	1.29	-25015.61	2.34	-24995.01	8.93
	-25160.95	-2.90	-25127.57	2.45	-25096.30	5.71	-25073.98	0.01	-25042.93	3.04	-25015.13	2.82	-24993.52	10.42
	-25161.16	-3.12	-25131.37	-1.35	-25101.28	0.73	-25068.50	5.49	-25042.75	3.22	-25013.01	4.94	-24996.93	7.01
	-25159.60	-1.56	-25130.56	-0.54	-25103.88	-1.87	-25073.80	0.19	-25046.17	-0.20	-25010.65	7.30	-24996.68	7.27
	-25159.28	-1.23	-25130.56	-0.53	-25101.16	0.85	-25070.29	3.70	-25045.13	0.84	-25014.70	3.25	-25000.93	3.01
	-25160.59	-2.54	-25131.28	-1.26	-25103.25	-1.24	-25071.71	2.28	-25037.38	8.59	-25017.35	0.60	-25001.90	2.04
	-25160.61	-2.57	-25131.29	-1.27	-25099.72	2.28	-25073.51	0.48	-25044.02	1.95	-25008.49	9.46	-24998.62	5.32
	-25160.82	-2.77	-25131.84	-1.82	-25103.61	-1.60	-25073.20	0.79	-25035.71	10.26	-25014.50	3.45	-25002.47	1.47
	-25160.53	-2.48	-25129.39	0.64	-25102.84	-0.83	-25071.53	2.46	-25041.21	4.76	-25010.46	7.49	-24998.28	5.66
	-25162.13	-4.08	-25131.21	-1.19	-25101.17	0.84	-25077.01	-3.02	-25042.03	3.94	-25009.16	8.79	-24996.98	6.97
	-25160.38	-2.34	-25130.84	-0.82	-25098.16	3.85	-25072.15	1.84	-25043.01	2.96	-25015.79	2.16	-24996.60	7.34
	-25159.79	-1.75	-25129.44	0.59	-25102.29	-0.28	-25074.51	-0.52	-25044.87	1.10	-25011.40	6.55	-25000.35	3.59

-25160.78	-2.73	-25131.43	-1.41	-25100.32	1.69	-25072.03	1.96	-25044.01	1.96	-25011.26	6.69	-24992.64	11.30
-25161.51	-3.46	-25130.82	-0.79	-25101.21	0.79	-25073.26	0.73	-25039.94	6.03	-25015.22	2.73	-24999.62	4.32
-25159.64	-1.59	-25130.87	-0.84	-25102.01	-0.01	-25071.23	2.76	-25036.77	9.20	-25014.46	3.49	-25000.59	3.35
-25160.07	-2.02	-25129.78	0.25	-25100.97	1.04	-25064.37	9.62	-25043.55	2.42	-25008.28	9.67	-25001.02	2.92
-25161.40	-3.36	-25131.76	-1.74	-25102.38	-0.37	-25070.77	3.22	-25042.95	3.02	-25015.49	2.47	-25002.72	1.22
-25160.86	-2.82	-25132.43	-2.41	-25103.09	-1.09	-25072.71	1.28	-25044.91	1.06	-25013.82	4.13	-25004.90	-0.96
-25160.57	-2.53	-25131.94	-1.91	-25102.17	-0.16	-25071.56	2.42	-25043.04	2.93	-25012.21	5.74	-25000.00	3.94
-25160.04	-1.99	-25131.39	-1.36	-25103.31	-1.30	-25073.76	0.22	-25044.29	1.68	-25015.54	2.41	-24995.79	8.15
-25160.77	-2.73	-25133.15	-3.12	-25101.07	0.94	-25071.85	2.14	-25041.00	4.97	-25013.20	4.75	-25003.84	0.10
-25154.53	3.51	-25132.08	-2.05	-25101.29	0.71	-25072.13	1.85	-25043.22	2.75	-25013.32	4.63	-25000.99	2.96
-25160.23	-2.19	-25129.05	0.98	-25101.48	0.53	-25072.39	1.60	-25045.14	0.83	-25013.31	4.64	-25001.57	2.37
-25160.24	-2.20	-25132.52	-2.50	-25103.38	-1.37	-25072.66	1.32	-25047.02	-1.05	-25006.10	11.85	-24998.49	5.45
-25159.92	-1.88	-25131.32	-1.29	-25099.88	2.13	-25075.52	-1.53	-25038.30	7.67	-25013.37	4.58	-25000.52	3.42
-25160.18	-2.14	-25130.52	-0.50	-25101.39	0.62	-25074.20	-0.21	-25043.20	2.77	-25015.54	2.41	-25003.72	0.22
-25160.77	-2.73	-25129.25	0.78	-25102.22	-0.21	-25070.24	3.75	-25042.48	3.49	-25015.21	2.74	-25002.65	1.29
-25155.38	2.67	-25131.75	-1.73	-25100.27	1.74	-25071.19	2.79	-25042.60	3.37	-25014.35	3.60	-25002.13	1.82
-25160.15	-2.11	-25130.36	-0.34	-25099.39	2.62	-25073.25	0.74	-25043.56	2.41	-25014.16	3.79	-24996.17	7.78
-25160.03	-1.99	-25131.32	-1.30	-25101.97	0.03	-25068.91	5.08	-25042.38	3.59	-25012.72	5.23	-25002.25	1.69
-25159.53	-1.49	-25131.11	-1.08	-25105.17	-3.17	-25073.27	0.72	-25046.15	-0.18	-25014.34	3.61	-25000.08	3.86
-25160.95	-2.91	-25131.94	-1.92	-25098.08	3.93	-25071.32	2.66	-25044.89	1.08	-25016.16	1.79	-24996.78	7.16
-25161.45	-3.41	-25130.80	-0.77	-25099.24	2.77	-25074.88	-0.89	-25046.81	-0.84	-25012.13	5.82	-24995.17	8.77
-25159.41	-1.36	-25129.89	0.13	-25100.57	1.44	-25070.21	3.78	-25042.34	3.63	-25016.63	1.32	-24998.66	5.28
-25160.66	-2.62	-25131.22	-1.20	-25097.48	4.53	-25074.04	-0.05	-25043.19	2.78	-25015.53	2.42	-25000.04	3.90
-25160.06	-2.02	-25130.45	-0.43	-25102.25	-0.24	-25074.20	-0.21	-25044.45	1.52	-25013.94	4.01	-25000.18	3.76
-25161.51	-3.47	-25130.14	-0.11	-25101.11	0.89	-25070.35	3.64	-25044.86	1.11	-25011.79	6.16	-25002.82	1.12
-25159.85	-1.81	-25131.12	-1.09	-25104.18	-2.18	-25072.57	1.42	-25034.93	11.04	-25009.21	8.74	-25001.56	2.39
-25160.87	-2.82	-25130.44	-0.42	-25101.36	0.64	-25074.55	-0.56	-25045.34	0.63	-25013.64	4.31	-25001.44	2.50
-25160.48	-2.44	-25129.30	0.72	-25104.19	-2.18	-25069.47	4.51	-25044.53	1.44	-25012.03	5.92	-25002.05	1.89
-25160.67	-2.63	-25129.50	0.52	-25101.46	0.55	-25072.71	1.28	-25039.04	6.93	-25011.62	6.33	-24999.44	4.50
-25161.02	-2.97	-25131.70	-1.68	-25102.53	-0.52	-25071.59	2.40	-25035.56	10.41	-25016.23	1.72	-24999.94	4.00
-25160.69	-2.65	-25130.84	-0.82	-25098.88	3.12	-25073.14	0.84	-25039.75	6.22	-25012.19	5.76	-24997.08	6.86
-25160.02	-1.97	-25128.88	1.14	-25100.80	1.20	-25073.16	0.82	-25032.69	13.28	-25011.77	6.18	-24996.71	7.23
-25161.62	-3.58	-25131.42	-1.39	-25101.17	0.84	-25073.73	0.26	-25044.57	1.40	-25016.39	1.56	-24998.31	5.63
-25159.68	-1.63	-25131.01	-0.99	-25103.51	-1.50	-25073.41	0.58	-25043.37	2.60	-25012.84	5.11	-25002.42	1.52
-25160.63	-2.59	-25131.38	-1.36	-25100.45	1.55	-25068.19	5.80	-25042.24	3.73	-25008.14	9.81	-24988.86	15.08
-25161.98	-3.94	-25127.03	3.00	-25099.20	2.81	-25069.76	4.23	-25046.80	-0.83	-25015.92	2.03	-24994.16	9.78
-25160.18	-2.14	-25130.77	-0.74	-25102.89	-0.89	-25072.89	1.10	-25042.24	3.73	-25013.16	4.80	-25003.56	0.38
-25161.07	-3.03	-25130.54	-0.51	-25102.07	-0.06	-25071.69	2.29	-25042.08	3.89	-25012.90	5.05	-25001.91	2.04
-25160.13	-2.09	-25128.06	1.96	-25102.46	-0.45	-25072.05	1.93	-25040.18	5.79	-25014.51	3.44	-25000.90	3.04
-25161.42	-3.38	-25133.12	-3.09	-25102.06	-0.06	-25072.12	1.87	-25042.23	3.74	-25015.60	2.35	-24997.62	6.32
-25155.08	2.97	-25130.03	-0.01	-25100.72	1.29	-25072.74	1.25	-25035.90	10.07	-25012.55	5.40	-24997.58	6.37

-25161.84	-3.80	-25131.16	-1.14	-25103.70	-1.69	-25072.27	1.72	-25040.66	5.31	-25016.28	1.67	-24998.22	5.72
-25160.40	-2.36	-25129.76	0.26	-25099.92	2.09	-25075.00	-1.01	-25044.31	1.66	-25010.73	7.22	-25000.19	3.75
-25160.24	-2.20	-25131.43	-1.40	-25100.35	1.66	-25070.64	3.34	-25042.67	3.30	-25012.14	5.81	-25000.05	3.89
-25158.97	-0.93	-25131.92	-1.89	-25100.78	1.23	-25068.26	5.73	-25040.90	5.07	-25013.71	4.24	-25001.31	2.63
-25161.87	-3.83	-25128.96	1.07	-25102.80	-0.79	-25073.98	0.01	-25038.56	7.41	-25018.38	-0.43	-25002.66	1.28
-25161.31	-3.27	-25130.60	-0.57	-25101.90	0.10	-25068.70	5.29	-25042.82	3.15	-25014.16	3.79	-25001.73	2.21
-25160.71	-2.67	-25129.77	0.26	-25101.08	0.92	-25073.01	0.98	-25041.58	4.39	-25014.73	3.22	-25000.72	3.22
-25161.10	-3.05	-25130.97	-0.95	-25101.37	0.64	-25068.77	5.21	-25045.26	0.71	-25012.93	5.02	-25003.07	0.87
-25152.44	5.61	-25131.11	-1.09	-25102.36	-0.35	-25066.14	7.84	-25039.71	6.26	-25015.64	2.31	-25000.46	3.48
-25160.13	-2.09	-25130.94	-0.92	-25104.30	-2.30	-25072.47	1.52	-25045.15	0.82	-25015.54	2.41	-24998.23	5.71
-25160.45	-2.41	-25131.14	-1.12	-25075.69	26.32	-25073.23	0.76	-25040.93	5.04	-25015.22	2.73	-25001.17	2.77
-25160.50	-2.46	-25131.34	-1.31	-25097.84	4.16	-25074.56	-0.57	-25044.16	1.81	-25009.96	7.99	-24998.91	5.03
-25160.75	-2.70	-25132.18	-2.15	-25093.56	8.45	-25073.19	0.80	-25042.23	3.74	-25013.36	4.59	-24996.83	7.11
-25161.43	-3.38	-25131.39	-1.36	-25100.82	1.18	-25073.09	0.90	-25041.22	4.75	-25015.54	2.41	-25001.74	2.20
-25160.24	-2.20	-25124.28	5.75	-25101.02	0.98	-25068.71	5.27	-25044.82	1.15	-25011.28	6.67	-24998.65	5.29
-25160.11	-2.07	-25131.49	-1.47	-25100.16	1.84	-25070.76	3.23	-25036.90	9.07	-25016.64	1.31	-25001.15	2.79
-25160.60	-2.56	-25131.63	-1.61	-25100.26	1.75	-25068.73	5.25	-25044.05	1.92			-25000.88	3.06
-25160.76	-2.72	-25128.84	1.18	-25093.58	8.42	-25066.23	7.75	-25044.41	1.56			-25000.49	3.45
-25159.73	-1.69	-25132.37	-2.35	-25095.87	6.14	-25069.80	4.19	-25043.59	2.38			-24996.46	7.48
-25160.63	-2.58	-25132.54	-2.51	-25102.49	-0.48	-25074.94	-0.95	-25043.57	2.40			-24999.18	4.76
-25157.81	0.24	-25131.10	-1.08	-25104.03	-2.03	-25073.95	0.04	-25042.79	3.18			-25001.90	2.04
-25160.95	-2.91	-25130.34	-0.31	-25101.07	0.94	-25068.33	5.66	-25040.64	5.33				
-25158.90	-0.86	-25131.27	-1.24	-25103.17	-1.16			-25045.32	0.65				
-25161.70	-3.65	-25132.23	-2.20	-25102.19	-0.18			-25044.83	1.13				
-25153.42	4.62	-25131.47	-1.45	-25102.41	-0.40								
-25159.71	-1.67	-25131.91	-1.89	-25100.61	1.39								
-25161.10	-3.05	-25129.88	0.15										
-25160.59	-2.55	-25131.24	-1.22										
-25161.08	-3.04	-25132.39	-2.36										
-25158.23	-0.19	-25127.52	2.51										
-25159.70	-1.65	-25131.81	-1.79										
-25159.09	-1.05	-25132.58	-2.55										
-25159.41	-1.37												
-25159.98	-1.93												
-25158.78	-0.73												
-25153.48	4.56												
-25160.49	-2.45												
-25159.78	-1.74												
-25155.09	2.95												
-25161.81	-3.77												

Average energies	-25160.00	-1.96	-25130.77	-0.75	-25100.76	1.25	-25071.89	2.10	-25042.40	3.57	-25013.47	4.48	-24999.57	4.37

$Ca_{1-x}M_xZrTi_{2-2x}Fe_{2x}O_7$ M=(Ce, Pu, Th, U)

Ti(1)Ti(1)

N.B. All stated energies are in eV

Ce

	ground state energy	solid solution energy													
concentration in solid solution (%)	3		6		9		12		15		18		21		
number of atoms	2		4		6		8		10		12		13		
	-25143.09	-0.20	-25097.32	2.39	-25048.07	8.47	-25001.95	11.42	-24955.67	14.53	-24912.48	14.54	-24881.37	24.07	
	-25143.77	-0.88	-25096.21	3.50	-25044.61	11.93	-25002.53	10.84	-24955.44	14.76	-24910.93	16.10	-24883.92	21.52	
	-25143.26	-0.37	-25097.77	1.95	-25049.75	6.80	-25002.41	10.96	-24955.22	14.98	-24908.04	18.98	-24883.33	22.11	
	-25143.88	-1.00	-25096.06	3.65	-25046.96	9.58	-25001.93	11.44	-24953.59	16.61	-24908.93	18.09	-24924.96	-19.52	
	-25143.62	-0.73	-25098.08	1.64	-25046.99	9.55	-25005.70	7.67	-24956.25	13.95	-24908.66	18.36	-24884.41	21.02	
	-25142.70	0.19	-25097.26	2.46	-25050.58	5.96	-25004.89	8.48	-24955.58	14.62	-24906.61	20.42	-24885.36	20.08	
	-25143.03	-0.14	-25097.82	1.89	-25051.39	5.16	-24998.16	15.21	-24954.15	16.05	-24913.27	13.75	-24884.59	20.84	
	-25144.72	-1.83	-25094.80	4.91	-25050.24	6.30	-25000.64	12.73	-24956.18	14.02	-24907.15	19.88	-24875.30	30.14	
	-25144.71	-1.82	-25098.28	1.43	-25051.44	5.11	-24998.07	15.30	-24956.06	14.13	-24903.84	23.19	-24887.15	18.28	
	-25144.26	-1.37	-25098.50	1.22	-25048.52	8.02	-25001.82	11.55	-24958.31	11.88	-24906.61	20.41	-24888.91	16.53	
	-25145.43	-2.54	-25096.56	3.16	-25052.34	4.20	-25002.16	11.21	-24956.77	13.42	-24910.82	16.20	-24889.51	15.93	
	-25145.38	-2.49	-25093.51	6.21	-25046.34	10.21	-25001.30	12.07	-24954.58	15.61	-24906.90	20.12	-24887.26	18.17	
	-25144.95	-2.06	-25097.59	2.13	-25050.77	5.77	-25045.55	-32.18	-24954.03	16.17	-24913.66	13.36	-24885.47	19.96	
	-25144.61	-1.72	-25096.78	2.94	-25048.98	7.57	-24999.28	14.09	-24956.18	14.01	-24907.65	19.37	-24884.59	20.85	
	-25145.55	-2.66	-25097.97	1.75	-25049.69	6.86	-25002.96	10.41	-24958.11	12.08	-24910.25	16.77	-24883.08	22.36	
	-25144.49	-1.60	-25094.30	5.41	-25047.11	9.43	-25003.14	10.23	-24955.00	15.20	-24905.78	21.24	-24882.36	23.07	
	-25144.11	-1.22	-25095.81	3.90	-25048.63	7.91	-25003.99	9.38	-24958.50	11.69	-24910.59	16.43	-24885.00	20.44	
	-25143.34	-0.45	-25097.14	2.58	-25051.41	5.13	-25003.27	10.10	-24956.55	13.65	-24910.39	16.63	-24885.97	19.47	
	-25142.64	0.25	-25096.66	3.06	-25047.93	8.61	-25000.94	12.43	-24953.80	16.39	-24907.81	19.21	-24882.92	22.52	
	-25145.67	-2.78	-25099.75	-0.04	-25049.81	6.73	-25004.33	9.04	-24952.70	17.49	-24910.10	16.93	-24881.28	24.15	
	-25143.57	-0.68	-25098.63	1.08	-25050.58	5.96	-25000.60	12.77	-24955.16	15.04	-24904.49	22.53	-24883.12	22.31	
	-25144.87	-1.98	-25095.86	3.85	-25045.78	10.76	-25003.87	9.50	-24953.74	16.46	-24908.80	18.23	-24888.93	16.51	
	-25143.95	-1.06	-25097.41	2.30	-25051.07	5.47	-24998.66	14.71	-24957.78	12.42	-24908.41	18.62	-24884.89	20.55	
	-25143.81	-0.92	-25096.77	2.95	-25050.62	5.93	-25006.13	7.24	-24955.42	14.77	-24950.02	-23.00	-24886.73	18.70	
	-25144.82	-1.93	-25097.91	1.80	-25050.74	5.80	-25001.31	12.06	-24953.03	17.16	-24905.59	21.43	-24888.89	16.55	
	-25144.61	-1.73	-25097.47	2.25	-25050.96	5.59	-25004.03	9.34	-24956.16	14.04	-24911.83	15.19	-24884.14	21.30	
	-25144.19	-1.30	-25097.62	2.09	-25048.89	7.65	-25002.93	10.44	-24955.09	15.10	-24884.37	42.65	-24884.49	20.95	
	-25144.19	-1.31	-25097.69	2.02	-25048.41	8.14	-25001.25	12.12	-24956.79	13.40	-24909.39	17.64	-24883.29	22.15	
	-25144.12	-1.23	-25097.27	2.45	-25002.11	54.43	-24954.39	58.98	-24908.44	61.75	-24910.68	16.34	-24882.63	22.80	

-25144.69	-1.80	-25099.77	-0.05	-25051.45	5.10	-25000.40	12.97	-24955.58	14.62	-24952.97	-25.95	-24887.50	17.93
-25142.18	0.70	-25096.86	2.86	-25048.63	7.91	-25002.33	11.04	-24956.07	14.13	-24909.76	17.26	-24881.11	24.32
-25144.39	-1.50	-25098.66	1.05	-25048.24	8.30	-24994.42	18.95	-24955.15	15.05	-24911.11	15.91	-24889.15	16.29
-25144.21	-1.32	-25095.90	3.82	-25049.52	7.03	-25000.52	12.85	-24952.81	17.38	-24911.50	15.53	-24878.47	26.96
-25143.50	-0.61	-25096.03	3.69	-25051.55	5.00	-25003.14	10.23	-24953.73	16.46	-24910.89	16.14	-24884.23	21.21
-25144.97	-2.09	-25096.56	3.15	-25052.64	3.90	-25005.52	7.85	-24957.50	12.70	-24909.89	17.13	-24883.16	22.28
-25145.00	-2.11	-25097.77	1.95	-25051.03	5.52	-25003.83	9.54	-24954.47	15.73	-24910.90	16.12	-24884.43	21.00
-25145.19	-2.30	-25097.16	2.55	-25049.48	7.06	-25004.02	9.35	-24956.90	13.29	-24910.45	16.57	-24884.84	20.60
-25143.67	-0.78	-25095.30	4.41	-25048.43	8.11	-25000.84	12.53	-24951.19	19.01	-24904.99	22.03	-24888.56	16.88
-25142.78	0.11	-25096.80	2.91	-25049.12	7.42	-25004.21	9.16	-24955.85	14.35	-24909.26	17.77	-24882.46	22.97
-25145.99	-3.11	-25098.44	1.27	-25050.11	6.44	-25005.36	8.00	-24957.39	12.80	-24908.66	18.37	-24887.21	18.23
-25145.57	-2.68	-25098.95	0.77	-25045.23	11.32	-25002.66	10.71	-24955.57	14.63	-24907.95	19.07	-24926.84	-21.41
-25144.08	-1.19	-25095.42	4.30	-25049.47	7.07	-25002.35	11.02	-24955.26	14.93	-24910.41	16.61	-24887.86	17.58
-25144.71	-1.82	-25095.30	4.42	-25050.24	6.30	-25005.18	8.19	-24958.15	12.05	-24904.70	22.32	-24886.02	19.42
-25144.94	-2.06	-25097.17	2.55	-25050.12	6.42	-25000.04	13.33	-24958.97	11.23	-24908.33	18.69	-24884.14	21.30
-25143.90	-1.01	-25098.44	1.27	-25049.61	6.94	-25002.37	11.00	-24952.05	18.15	-24906.36	20.66	-24889.21	16.23
-25143.24	-0.35	-25098.38	1.34	-25043.33	13.21	-25004.81	8.56	-24953.27	16.92	-24909.45	17.57	-24877.76	27.68
-25145.20	-2.31	-25095.73	3.99	-25050.33	6.21	-25003.21	10.16	-24957.46	12.74	-24909.53	17.49	-24879.03	26.40
-25142.45	0.44	-25095.22	4.49	-25050.43	6.11	-25005.68	7.69	-24958.50	11.70	-24910.79	16.23	-24885.90	19.54
-25144.77	-1.88	-25094.85	4.86	-25051.09	5.45	-25001.64	11.73	-24954.03	16.16	-24906.26	20.76	-24887.03	18.41
-25144.09	-1.20	-25093.20	6.52	-25050.22	6.32	-25001.23	12.14	-24954.35	15.84	-24909.87	17.15	-24880.36	25.07
-25145.75	-2.86	-25097.52	2.20	-25051.14	5.40	-25001.58	11.79	-24957.02	13.18	-24904.11	22.91	-24883.98	21.46
-25145.34	-2.45	-25097.95	1.76	-25049.34	7.20	-24997.87	15.50	-24953.23	16.96	-24908.21	18.81	-24884.36	21.08
-25143.85	-0.97	-25097.81	1.91	-25051.00	5.54	-25004.07	9.30	-24958.34	11.85	-24908.64	18.38	-24887.23	18.20
-25144.25	-1.37	-25098.82	0.90	-25053.19	3.36	-25002.86	10.51	-24955.63	14.57	-24906.29	20.73	-24880.78	24.65
-25143.43	-0.54	-25098.27	1.44	-25048.52	8.02	-25001.17	12.20	-24957.49	12.70	-24909.91	17.11	-24886.04	19.40
-25144.77	-1.88	-25097.15	2.57	-25051.02	5.52	-24999.90	13.47	-24955.79	14.41	-24909.55	17.47	-24885.86	19.58
-25144.62	-1.73	-25096.55	3.17	-25050.62	5.92	-25001.64	11.73	-24957.99	12.20	-24902.08	24.95	-24888.22	17.22
-25145.80	-2.91	-25097.21	2.51	-25052.18	4.37	-24998.99	14.37	-24958.83	11.36	-24904.54	22.48	-24882.47	22.97
-25144.88	-1.99	-25096.57	3.15	-25043.58	12.96	-25006.40	6.97	-24955.92	14.27	-24905.85	21.17	-24883.27	22.16
-25144.53	-1.64	-25097.09	2.62	-25049.40	7.15	-24999.41	13.96	-24957.70	12.49	-24906.47	20.55	-24888.62	16.82
-25144.72	-1.83	-25099.40	0.32	-25047.62	8.92	-25002.57	10.80	-24953.99	16.20	-24913.19	13.84	-24882.68	22.75
-25142.28	0.61	-25098.64	1.08	-25052.05	4.49	-25003.03	10.34	-24954.39	15.81	-24906.91	20.11	-24885.55	19.88
-25145.34	-2.45	-25098.03	1.68	-25051.72	4.82	-25001.85	11.52	-24951.71	18.49	-24908.19	18.83	-24885.92	19.52
-25143.66	-0.77	-25097.55	2.17	-25049.89	6.66	-25002.27	11.10	-24955.56	14.63	-24913.11	13.91	-24880.61	24.83
-25144.15	-1.26	-25097.63	2.08	-25048.26	8.28	-24999.57	13.79	-24940.15	30.05	-24909.55	17.48	-24888.59	16.85
-25145.81	-2.92	-25098.97	0.75	-25047.95	8.59	-25006.30	7.07	-24957.02	13.18	-24909.14	17.89	-24883.32	22.12
-25145.82	-2.93	-25095.64	4.08	-25049.42	7.12	-25003.93	9.44	-24958.13	12.06	-24910.33	16.69	-24885.19	20.25
-25143.98	-1.09	-25097.73	1.99	-25050.38	6.16	-25004.19	9.18	-24956.62	13.58	-24906.74	20.28	-24883.22	22.21
-25142.03	0.86	-25098.01	1.71	-25046.89	9.65	-25004.74	8.63	-24954.54	15.66	-24910.38	16.64	-24884.92	20.52
-25144.35	-1.46	-25098.49	1.23	-25050.54	6.00	-25005.15	8.22	-24953.27	16.93	-24908.22	18.80	-24878.73	26.70

-25144.39	-1.50	-25097.20	2.51	-25051.01	5.53	-24998.56	14.81	-24953.69	16.51	-24910.38	16.64	-24883.39	22.04
-25143.47	-0.59	-25098.06	1.65	-25049.57	6.97	-25000.30	13.07	-24957.46	12.74	-24910.07	16.95	-24884.36	21.08
-25144.90	-2.01	-25095.93	3.79	-25050.47	6.08	-25003.22	10.15	-24951.54	18.66	-24905.13	21.89	-24882.02	23.41
-25144.88	-1.99	-25096.20	3.51	-25051.64	4.91	-25000.00	13.36	-24957.66	12.53	-24910.53	16.50	-24887.72	17.71
-25144.81	-1.93	-25097.26	2.46	-25048.76	7.78	-25004.62	8.75	-24954.29	15.90	-24908.04	18.98	-24884.94	20.50
-25143.86	-0.97	-25096.38	3.34	-25052.08	4.46	-25001.53	11.84	-24954.69	15.50	-24911.97	15.05	-24877.70	27.74
-25146.37	-3.48	-25098.50	1.21	-25050.13	6.41	-25000.41	12.96	-24951.43	18.76	-24905.94	21.08	-24880.85	24.59
-25144.41	-1.53	-25098.82	0.89	-25047.92	8.62	-25003.59	9.78	-24955.99	14.21	-24912.23	14.79	-24881.97	23.46
-25144.05	-1.16	-25099.12	0.60	-25049.35	7.19	-25001.96	11.41	-24953.48	16.72	-24883.28	43.74	-24887.78	17.66
-25145.37	-2.48	-25095.60	4.11	-25044.58	11.96	-25002.82	10.55	-24955.52	14.68	-24910.74	16.28	-24883.07	22.37
-25144.52	-1.64	-25098.31	1.40	-25050.89	5.65	-25001.02	12.35	-24958.74	11.46	-24904.70	22.32	-24889.63	15.81
-25144.82	-1.93	-25097.52	2.20	-25047.25	9.29	-24995.43	17.94	-24956.48	13.71	-24906.44	20.58	-24880.05	25.38
-25144.41	-1.52	-25096.86	2.86	-25048.51	8.04	-24956.31	57.06	-24955.74	14.46	-24907.82	19.20	-24885.45	19.99
-25143.87	-0.98	-25097.59	2.13	-25050.41	6.14	-25005.96	7.41	-24904.33	65.87	-24910.34	16.68	-24885.11	20.33
-25143.78	-0.89	-25098.89	0.83	-25048.90	7.64	-25000.30	13.07	-24955.26	14.93	-24912.68	14.34	-24887.25	18.19
-25144.68	-1.79	-25096.51	3.21	-25050.95	5.59	-25004.32	9.05	-24956.72	13.48	-24907.10	19.93	-24883.55	21.88
-25143.48	-0.60	-25095.83	3.89	-25050.49	6.05	-24998.03	15.34	-24953.71	16.48	-24906.18	20.84	-24882.23	23.20
-25144.21	-1.32	-25096.17	3.55	-25050.96	5.58	-25003.90	9.47	-24955.59	14.60	-24909.36	17.66	-24879.87	25.56
-25145.22	-2.33	-25095.38	4.34	-25052.61	3.93	-24997.21	16.16	-24947.81	22.39	-24910.49	16.54	-24885.65	19.79
-25145.27	-2.38	-25098.45	1.27	-25050.03	6.51	-25004.02	9.35	-24956.18	14.01	-24911.89	15.13	-24885.10	20.33
-25145.52	-2.63	-25099.11	0.61	-25049.84	6.70	-24999.61	13.76	-24960.14	10.06	-24912.18	14.84	-24886.52	18.91
-25144.45	-1.56	-25097.26	2.45	-25050.36	6.19	-25001.18	12.19	-24957.19	13.00	-24909.03	17.99	-24886.60	18.84
-25143.83	-0.94	-25099.00	0.71	-25050.83	5.71	-25005.15	8.22	-24953.81	16.39	-24904.77	22.26	-24879.33	26.11
-25144.48	-1.59	-25099.38	0.33	-25049.95	6.59	-25004.66	8.71	-24953.41	16.78	-24907.90	19.13	-24882.02	23.42
-25144.80	-1.91	-25093.70	6.02	-25048.56	7.98	-25003.56	9.81	-24954.04	16.16	-24910.75	16.28	-24885.95	19.49
-25143.85	-0.96	-25097.55	2.16	-25050.27	6.27	-25004.07	9.30	-24953.66	16.54	-24904.64	22.38	-24883.03	22.40
-25145.81	-2.93	-25098.95	0.77	-25050.98	5.56	-25001.97	11.40	-24956.59	13.61	-24906.88	20.14	-24886.28	19.16
-25145.44	-2.55	-25098.15	1.57	-25048.81	7.73	-25003.40	9.97	-24955.97	14.22	-24908.91	18.11	-24882.03	23.41
-25145.27	-2.38	-25096.45	3.27	-25049.24	7.30	-24999.41	13.96	-24955.61	14.59	-24906.07	20.95	-24885.77	19.67
-25144.51	-1.62	-25098.84	0.87	-25048.46	8.09	-25001.34	12.03	-24955.65	14.55	-24905.20	21.83	-24885.34	20.10
-25144.58	-1.69	-25098.72	1.00	-25049.72	6.83	-25000.58	12.79	-24956.04	14.15	-24906.68	20.34	-24886.71	18.73
-25144.73	-1.84	-25096.21	3.51	-25047.64	8.90	-25003.75	9.62	-24954.69	15.51	-24909.99	17.03	-24886.19	19.25
-25143.34	-0.45	-25098.61	1.10	-25051.38	5.16	-25001.48	11.89	-24954.79	15.41	-24908.45	18.57	-24885.44	19.99
-25145.63	-2.75	-25097.52	2.19	-25049.21	7.33	-25001.41	11.96	-24957.04	13.15	-24909.38	17.65	-24890.11	15.33
-25145.04	-2.15	-25094.87	4.84	-25047.83	8.71	-25002.03	11.34	-24958.36	11.84	-24906.99	20.04	-24884.91	20.52
-25144.00	-1.11	-25097.57	2.15	-25048.64	7.91	-25001.77	11.60	-24955.19	15.00	-24906.22	20.81	-24886.72	18.72
-25144.93	-2.04	-25096.58	3.14	-25049.87	6.68	-25000.92	12.45	-24957.88	12.32	-24906.77	20.25	-24878.18	27.25
-25144.15	-1.26	-25099.22	0.49	-25051.34	5.21	-25003.99	9.38	-24958.37	11.83	-24910.04	16.99	-24883.29	22.14
-25143.47	-0.58	-25097.03	2.68	-25047.73	8.81	-25003.75	9.62	-24954.72	15.47	-24908.37	18.66	-24887.97	17.46
-25142.99	-0.10	-25095.10	4.62	-25051.03	5.51	-25003.51	9.86	-24956.18	14.02	-24908.28	18.74	-24886.51	18.93
-25144.12	-1.24	-25097.40	2.31	-25049.73	6.81	-25001.90	11.47	-24957.50	12.69	-24908.75	18.28	-24887.05	18.39

	-25144.36	-1.47	-25097.77	1.95	-25050.90	5.64	-25002.55	10.82	-24957.03	13.17	-24878.58	48.44	-24888.39	17.04
	-25145.03	-2.14	-25098.33	1.39	-25049.93	6.62	-25000.09	13.28	-24951.79	18.41			-24886.68	18.76
	-25144.49	-1.60	-25097.83	1.89	-25050.98	5.56	-25002.47	10.90	-24958.14	12.05			-24886.59	18.85
	-25144.72	-1.84	-25096.97	2.75	-25047.48	9.06	-25000.32	13.05	-24946.92	23.28				
	-25144.72	-1.83	-25095.96	3.76	-25051.91	4.64	-24992.48	20.89	-24952.88	17.32				
	-25143.46	-0.57	-25098.22	1.49	-25049.14	7.40			-24959.72	10.48				
	-25146.25	-3.36			-25051.12	5.43			-24949.09	21.10				
	-25144.07	-1.18												
	-25144.98	-2.10												
Average energies	-25144.4	-1.51153	-25097.3	2.46279	-25049.2	7.346962	-25001.6	11.72851	-24954.5	15.69946	-24908.7	18.36525	-24885.3	20.13808

Pu

	ground state energy	solid solution energy												
concentration in solid solution (%)	3		6		9		12		15		18		21	
number of atoms	2		4		6		8		10		12		13	
	-25138.27	-1.37	-25086.45	1.29	-25032.30	6.28	-24980.53	8.90	-24925.94	14.33	-24875.55	15.56	-24847.04	19.49
	-25138.28	-1.37	-25087.18	0.57	-25031.43	7.15	-24981.67	7.76	-24930.26	10.01	-24868.16	22.95	-24848.04	18.49
	-25138.12	-1.22	-25086.34	1.40	-25032.34	6.25	-24979.71	9.72	-24925.73	14.54	-24875.37	15.74	-24850.56	15.97
	-25138.50	-1.59	-25085.18	2.56	-25030.81	7.77	-24979.67	9.75	-24926.34	13.92	-24877.81	13.30	-24887.56	-21.03
	-25139.20	-2.30	-25087.74	0.01	-25033.82	4.76	-24979.37	10.06	-24924.64	15.63	-24872.37	18.74	-24849.53	17.00
	-25138.50	-1.59	-25086.48	1.26	-25034.22	4.36	-24978.55	10.88	-24926.30	13.96	-24875.29	15.82	-24847.47	19.06
	-25137.07	-0.17	-25087.48	0.27	-25034.19	4.40	-24981.02	8.41	-24926.11	14.16	-24872.20	18.91	-24845.87	20.66
	-25139.40	-2.49	-25086.43	1.31	-25032.03	6.56	-24982.81	6.61	-24927.28	12.98	-24876.89	14.22	-24849.27	17.26
	-25139.10	-2.19	-25086.91	0.83	-25034.92	3.67	-24977.69	11.74	-24927.59	12.68	-24879.46	11.65	-24854.92	11.61
	-25139.61	-2.71	-25086.96	0.79	-25034.06	4.53	-24979.59	9.84	-24926.74	13.53	-24876.49	14.62	-24850.27	16.26
	-25139.10	-2.20	-25086.05	1.69	-25034.96	3.63	-24982.14	7.28	-24930.70	9.57	-24876.15	14.96	-24852.33	14.20
	-25138.80	-1.90	-25086.01	1.73	-25031.72	6.86	-24983.56	5.86	-24926.00	14.27	-24876.08	15.03	-24854.44	12.09
	-25138.07	-1.16	-25085.36	2.38	-25032.42	6.17	-24977.17	12.25	-24927.10	13.17	-24875.93	15.18	-24846.71	19.82
	-25139.72	-2.81	-25084.47	3.28	-25033.98	4.60	-24981.56	7.86	-24926.92	13.35	-24871.97	19.14	-24847.88	18.65
	-25138.20	-1.30	-25086.12	1.63	-25034.17	4.42	-24984.10	5.33	-24928.01	12.25	-24876.74	14.37	-24848.69	17.84
	-25138.57	-1.66	-25083.72	4.02	-25031.14	7.44	-24979.50	9.92	-24929.05	11.21	-24873.83	17.28	-24849.43	17.10
	-25139.03	-2.13	-25087.97	-0.22	-25031.88	6.71	-24982.81	6.61	-24926.60	13.67	-24877.60	13.51	-24848.73	17.80
	-25136.96	-0.05	-25083.18	4.57	-25033.05	5.54	-24979.65	9.78	-24927.53	12.74	-24876.69	14.41	-24851.72	14.81
	-25138.68	-1.77	-25085.64	2.10	-25030.70	7.88	-24976.25	13.18	-24929.92	10.35	-24872.54	18.57	-24852.46	14.07
	-25138.47	-1.56	-25087.78	-0.04	-25034.66	3.92	-24980.87	8.55	-24929.79	10.48	-24877.56	13.55	-24851.63	14.90
	-25138.93	-2.03	-25084.54	3.20	-25035.37	3.22	-24982.40	7.03	-24924.95	15.32	-24875.98	15.13	-24844.60	21.93
	-25138.48	-1.58	-25084.99	2.75	-25030.63	7.96	-24981.79	7.63	-24928.56	11.71	-24875.51	15.60	-24852.26	14.27
	-25137.29	-0.38	-25086.52	1.22	-25032.68	5.90	-24979.79	9.63	-24927.74	12.52	-24871.59	19.52	-24845.43	21.10

-25139.77	-2.86	-25086.57	1.18	-25033.15	5.44	-24981.93	7.50	-24927.52	12.75	-24876.60	14.51	-24852.10	14.43
-25137.77	-0.87	-25085.13	2.62	-25033.27	5.31	-24981.13	8.29	-24930.29	9.98	-24874.26	16.85	-24851.15	15.38
-25138.73	-1.83	-25084.88	2.87	-25033.91	4.67	-24982.69	6.74	-24929.20	11.07	-24876.14	14.97	-24848.59	17.94
-25139.47	-2.56	-25086.35	1.39	-25033.94	4.65	-24978.47	10.96	-24924.91	15.36	-24874.97	16.14	-24850.11	16.42
-25137.41	-0.51	-25087.16	0.58	-25029.48	9.11	-24982.16	7.26	-24927.63	12.64	-24876.70	14.41	-24847.32	19.21
-25139.79	-2.89	-25085.95	1.79	-25033.64	4.95	-24978.90	10.52	-24925.82	14.45	-24869.11	22.00	-24847.44	19.09
-25137.64	-0.74	-25086.91	0.84	-25034.41	4.17	-24979.82	9.60	-24930.55	9.72	-24873.79	17.31	-24851.66	14.87
-25138.56	-1.66	-25085.70	2.04	-25032.31	6.28	-24981.38	8.04	-24927.74	12.53	-24870.42	20.69	-24851.08	15.45
-25138.57	-1.67	-25086.76	0.98	-25034.76	3.82	-24975.63	13.79	-24925.50	14.77	-24876.38	14.73	-24849.66	16.87
-25139.34	-2.43	-25085.62	2.13	-25032.05	6.54	-24966.55	22.88	-24928.32	11.94	-24871.86	19.25	-24846.59	19.94
-25137.94	-1.04	-25085.63	2.11	-25031.49	7.10	-24981.79	7.63	-24926.07	14.20	-24873.40	17.71	-24851.37	15.16
-25139.35	-2.45	-25084.17	3.58	-25033.41	5.17	-24983.47	5.95	-24931.70	8.57	-24874.20	16.91	-24852.54	13.99
-25137.63	-0.72	-25086.77	0.98	-25031.84	6.75	-24981.23	8.20	-24925.14	15.13	-24874.32	16.79	-24851.19	15.34
-25138.88	-1.98	-25087.13	0.61	-25033.19	5.40	-24980.49	8.94	-24928.06	12.21	-24878.11	13.00	-24852.65	13.88
-25138.58	-1.68	-25084.84	2.90	-25030.15	8.43	-24978.82	10.60	-24928.22	12.05	-24878.28	12.83	-24849.55	16.98
-25138.14	-1.24	-25086.61	1.13	-25030.19	8.39	-24980.66	8.77	-24925.84	14.43	-24876.98	14.13	-24848.63	17.90
-25139.47	-2.56	-25086.24	1.51	-25035.05	3.54	-24981.82	7.61	-24930.63	9.64	-24876.76	14.35	-24847.48	19.05
-25136.89	0.01	-25087.08	0.67	-25036.08	2.51	-24979.05	10.38	-24926.25	14.02	-24877.16	13.95	-24852.50	14.03
-25138.35	-1.45	-25084.84	2.90	-25033.54	5.04	-24985.11	4.32	-24928.65	11.62	-24873.62	17.49	-24855.29	11.24
-25139.24	-2.33	-25085.18	2.57	-25034.01	4.58	-24981.36	8.06	-24930.00	10.27	-24874.49	16.62	-24847.46	19.07
-25138.76	-1.86	-25086.53	1.22	-25034.22	4.36	-24979.32	10.11	-24927.04	13.22	-24872.17	18.94	-24851.38	15.15
-25137.59	-0.68	-25086.17	1.57	-25034.19	4.39	-24979.58	9.84	-24929.20	11.07	-24878.99	12.12	-24848.26	18.27
-25137.61	-0.71	-25085.55	2.19	-25031.85	6.74	-24984.13	5.30	-24926.72	13.55	-24872.15	18.96	-24849.13	17.40
-25139.68	-2.77	-25086.41	1.34	-25031.22	7.37	-24981.35	8.07	-24926.92	13.35	-24877.46	13.65	-24851.23	15.30
-25138.35	-1.45	-25085.50	2.25	-25031.66	6.92	-24976.99	12.44	-24926.71	13.56	-24879.24	11.87	-24845.43	21.10
-25139.52	-2.62	-25087.47	0.28	-25033.20	5.39	-24981.01	8.41	-24926.96	13.30	-24877.53	13.58	-24850.68	15.85
-25138.82	-1.91	-25087.15	0.59	-25033.16	5.43	-24981.71	7.71	-24931.36	8.91	-24874.68	16.43	-24852.92	13.61
-25138.84	-1.94	-25086.63	1.11	-25034.94	3.65	-24983.45	5.98	-24927.14	13.13	-24877.34	13.77	-24848.39	18.14
-25139.16	-2.25	-25086.54	1.20	-25031.91	6.67	-24980.59	8.84	-24927.01	13.26	-24877.57	13.54	-24847.27	19.26
-25138.93	-2.02	-25085.74	2.01	-25032.66	5.93	-24980.45	8.97	-24922.10	18.17	-24872.68	18.43	-24851.16	15.37
-25136.71	0.20	-25086.32	1.43	-25032.62	5.97	-24974.93	14.50	-24929.67	10.60	-24873.85	17.26	-24842.96	23.57
-25138.69	-1.79	-25087.12	0.62	-25032.08	6.51	-24978.11	11.32	-24926.66	13.61	-24880.91	10.20	-24849.68	16.85
-25138.94	-2.04	-25085.38	2.37	-25032.31	6.27	-24981.71	7.71	-24929.80	10.46	-24879.21	11.90	-24853.56	12.97
-25138.17	-1.27	-25085.41	2.34	-25034.60	3.98	-24980.14	9.29	-24931.46	8.81	-24878.35	12.76	-24843.93	22.60
-25139.42	-2.52	-25084.94	2.81	-25033.77	4.81	-24978.39	11.04	-24928.00	12.27	-24879.28	11.82	-24850.24	16.30
-25137.74	-0.84	-25084.76	2.98	-25029.52	9.07	-24983.53	5.89	-24929.79	10.48	-24866.53	24.58	-24849.88	16.65
-25138.58	-1.68	-25087.39	0.35	-25033.31	5.28	-24980.41	9.02	-24927.96	12.31	-24870.82	20.29	-24851.18	15.35
-25139.29	-2.38	-25082.50	5.24	-25032.31	6.28	-24980.18	9.25	-24926.30	13.97	-24881.49	9.62	-24855.81	10.72
-25139.81	-2.90	-25085.06	2.68	-25030.33	8.26	-24982.30	7.13	-24928.16	12.10	-24873.60	17.51	-24850.45	16.08
-25138.74	-1.83	-25084.45	3.29	-25028.96	9.62	-24984.17	5.25	-24927.09	13.18	-24877.01	14.10	-24851.46	15.07
-25139.32	-2.41	-25085.80	1.94	-25032.57	6.01	-24976.82	12.61	-24926.97	13.29	-24849.77	41.34	-24849.77	16.76

-25138.59	-1.69	-25083.58	4.17	-25033.68	4.91	-24978.63	10.79	-24930.71	9.56	-24879.70	11.41	-24846.03	20.50
-25137.60	-0.70	-25086.67	1.07	-25035.44	3.14	-24980.64	8.78	-24928.97	11.30	-24880.73	10.38	-24851.61	14.92
-25139.01	-2.11	-25083.80	3.94	-25033.32	5.26	-24978.56	10.86	-24878.59	61.68	-24876.32	14.79	-24854.35	12.18
-25137.95	-1.05	-25086.01	1.74	-25031.49	7.10	-24930.25	59.17	-24929.39	10.88	-24875.82	15.29	-24848.82	17.71
-25138.00	-1.10	-25084.06	3.68	-25033.72	4.86	-24979.10	10.33	-24926.32	13.95	-24876.52	14.59	-24846.95	19.58
-25139.19	-2.29	-25087.65	0.10	-25033.18	5.41	-24979.94	9.49	-24923.22	17.04	-24875.91	15.20	-24846.35	20.18
-25138.95	-2.04	-25084.36	3.38	-25035.43	3.15	-24982.70	6.73	-24923.96	16.31	-24877.95	13.16	-24846.35	20.18
-25137.75	-0.85	-25083.68	4.06	-25033.40	5.18	-24978.75	10.67	-24927.97	12.30	-24873.09	18.02	-24852.60	13.93
-25138.68	-1.77	-25083.41	4.34	-25033.28	5.31	-24981.00	8.43	-24927.70	12.57	-24875.24	15.87	-24848.50	18.03
-25138.73	-1.83	-25084.80	2.95	-25033.71	4.88	-24981.34	8.09	-24924.26	16.01	-24875.75	15.36	-24851.42	15.11
-25138.46	-1.56	-25087.74	0.00	-25034.69	3.90	-24981.81	7.62	-24931.44	8.83	-24879.11	11.99	-24854.04	12.49
-25137.85	-0.95	-25085.07	2.68	-25034.60	3.98	-24978.75	10.68	-24927.11	13.16	-24877.55	13.56	-24848.83	17.70
-25138.70	-1.80	-25086.79	0.95	-25030.46	8.12	-24981.72	7.71	-24928.72	11.55	-24876.43	14.68	-24848.27	18.26
-25139.07	-2.17	-25088.03	-0.29	-25032.28	6.30	-24980.01	9.42	-24925.23	15.04	-24875.51	15.60	-24848.97	17.56
-25137.99	-1.08	-25088.11	-0.37	-25031.55	7.03	-24983.74	5.69	-24929.00	11.27	-24879.09	12.02	-24848.76	17.77
-25136.64	0.26	-25087.51	0.23	-25033.29	5.29	-24980.12	9.31	-24924.56	15.70	-24872.59	18.52	-24849.74	16.79
-25138.04	-1.14	-25085.80	1.94	-25031.61	6.98	-24981.18	8.25	-24930.37	9.90	-24874.10	17.01	-24841.73	24.80
-25138.97	-2.07	-25086.26	1.49	-25035.22	3.37	-24982.17	7.26	-24931.81	8.46	-24876.59	14.52	-24845.10	21.43
-25139.31	-2.41	-25086.90	0.85	-25033.98	4.60	-24982.54	6.89	-24928.85	11.42	-24876.48	14.63	-24845.29	21.24
-25137.84	-0.94	-25086.61	1.13	-25033.11	5.48	-25020.47	-31.04	-24927.98	12.29	-24878.51	12.60	-24846.61	19.92
-25139.06	-2.15	-25085.62	2.13	-25034.45	4.13	-24982.52	6.90	-24923.91	16.36	-24879.46	11.65	-24850.21	16.32
-25137.61	-0.71	-25086.72	1.02	-25029.69	8.90	-24980.15	9.27	-24923.90	16.37	-24878.32	12.78	-24848.00	18.53
-25139.09	-2.19	-25086.28	1.47	-25033.26	5.32	-24980.44	8.99	-24928.48	11.79	-24876.25	14.86	-24852.87	13.66
-25139.96	-3.06	-25087.45	0.29	-25029.91	8.67	-24977.96	11.46	-24930.27	10.00	-24849.94	41.17	-24845.97	20.56
-25138.19	-1.29	-25085.68	2.07	-24980.72	57.87	-24981.73	7.70	-24928.60	11.67	-24877.76	13.35	-24850.69	15.84
-25139.41	-2.50	-25085.17	2.57	-25034.26	4.33	-24925.94	63.48	-24929.60	10.67	-24873.76	17.35	-24847.35	19.18
-25137.00	-0.10	-25087.44	0.31	-25032.35	6.24	-24979.93	9.49	-24931.37	8.89	-24876.63	14.48	-24851.47	15.06
-25138.94	-2.04	-25035.02	52.73	-25032.60	5.98	-24982.32	7.11	-24877.23	63.04	-24876.10	15.01	-24849.12	17.41
-25085.14	51.76	-25085.63	2.12	-24979.07	59.52	-24981.63	7.80	-24926.42	13.84	-24874.81	16.30	-24850.69	15.84
-25139.39	-2.48	-25085.21	2.53	-25033.61	4.97	-24927.40	62.03	-24931.50	8.76	-24876.05	15.06	-24845.34	21.19
-25139.47	-2.57	-25087.81	-0.07	-25034.53	4.06	-24978.46	10.97	-24925.75	14.52	-24874.64	16.46	-24851.93	14.60
-25138.38	-1.47	-25082.51	5.23	-25032.28	6.31	-24983.34	6.09	-24875.87	64.40	-24876.35	14.76	-24851.71	14.82
-25140.78	-3.87	-25085.63	2.11	-25034.60	3.99	-24978.39	11.04	-24928.25	12.02	-24879.33	11.78	-24852.02	14.51
-25137.74	-0.84	-25086.78	0.97	-25033.77	4.82	-24979.84	9.59	-24929.05	11.21	-24876.25	14.86	-24851.98	14.55
-25138.54	-1.63	-25085.75	2.00	-25033.48	5.11	-24979.83	9.60	-24926.75	13.52	-24876.91	14.20	-24847.18	19.35
-25138.19	-1.29	-25086.61	1.13	-25033.18	5.40	-24984.51	4.92	-24928.29	11.98	-24876.53	14.58	-24852.93	13.60
-25138.47	-1.57	-25083.17	4.57	-25032.55	6.03	-24978.32	11.10	-24924.14	16.13	-24875.95	15.16	-24849.11	17.42
-25138.76	-1.85	-25085.89	1.85	-25033.40	5.19	-24979.39	10.04	-24926.80	13.47	-24874.21	16.90	-24847.58	18.95
-25139.75	-2.85	-25084.50	3.25	-25031.17	7.41	-24977.64	11.79	-24929.42	10.85	-24875.30	15.81	-24850.73	15.80
-25138.03	-1.12	-25085.60	2.14	-25032.51	6.07	-24981.59	7.84	-24931.49	8.78	-24878.14	12.97	-24853.18	13.35
-25135.78	1.12	-25084.33	3.42	-25031.73	6.86	-24979.30	10.13	-24928.91	11.36	-24879.11	12.00	-24850.82	15.71

	-25138.41	-1.51	-25085.15	2.60	-25028.75	9.83	-24980.34	9.08	-24930.36	9.91	-24874.38	16.73	-24851.45	15.08
	-25138.50	-1.60	-25085.72	2.02	-25035.28	3.31	-24981.02	8.40	-24927.82	12.45	-24873.86	17.25	-24852.12	14.41
	-25138.64	-1.74	-25085.98	1.77	-25033.38	5.21	-24980.22	9.21	-24931.69	8.58	-24876.94	14.17	-24849.20	17.33
	-25139.48	-2.58	-25081.19	6.55	-25033.90	4.68	-24975.96	13.46	-24921.96	18.31	-24876.13	14.98	-24849.96	16.57
	-25138.96	-2.06	-25086.41	1.33	-25034.91	3.68	-24981.92	7.50	-24924.07	16.20	-24876.84	14.27	-24854.41	12.12
	-25139.62	-2.71	-25086.54	1.20	-25033.94	4.64	-24980.67	8.76	-24928.61	11.65	-24875.13	15.98	-24848.99	17.54
	-25138.43	-1.52	-25085.27	2.47	-25035.26	3.32	-24983.28	6.15	-24930.13	10.14	-24876.55	14.56		
	-25138.77	-1.86	-25084.90	2.84	-25033.41	5.18	-24978.11	11.32	-24927.44	12.83	-24874.35	16.76		
	-25138.48	-1.58	-25085.09	2.66	-25031.67	6.91	-24977.34	12.09	-24966.93	-26.66				
	-25138.07	-1.17	-25086.91	0.83	-25036.33	2.26	-24984.12	5.31	-24926.97	13.30				
	-25137.25	-0.35	-25086.24	1.51					-24928.34	11.92				
	-25138.56	-1.65	-25085.93	1.81					-24929.99	10.28				
	-25138.80	-1.90	-25083.04	4.71					-24928.15	12.12				
Average energies	-25138.11	-1.21	-25085.39	2.36	-25032.05	6.53	-24979.40	10.03	-24926.83	13.44	-24875.35	15.76	-24850.03	16.50

Th

	ground state energy	solid solution energy												
concentration in solid solution (%)	3		6		9		12		15		18		21	
number of atoms	2		4		6		8		10		12		13	
	-25144.04	-1.51	-25097.16	1.84	-25049.54	5.94	-25001.80	10.15	-24954.93	13.49	-24907.06	17.83	-24882.63	20.50
	-25143.43	-0.90	-25095.98	3.03	-25049.95	5.53	-25000.95	11.00	-24955.00	13.42	-24905.13	19.76	-24880.65	22.48
	-25143.29	-0.76	-25095.72	3.29	-25049.95	5.53	-25042.82	-30.87	-24952.00	16.42	-24907.85	17.04	-24879.13	24.00
	-25145.20	-2.66	-25096.40	2.61	-25050.79	4.69	-25000.84	11.11	-24952.00	16.42	-24907.66	17.24	-24880.05	23.08
	-25143.24	-0.71	-25097.24	1.76	-25046.76	8.72	-25002.59	9.36	-24954.28	14.14	-24901.21	23.68	-24877.86	25.27
	-25144.06	-1.53	-25096.66	2.34	-25049.62	5.86	-25002.36	9.59	-24953.47	14.95	-24906.55	18.34	-24886.64	16.49
	-25144.46	-1.92	-25096.80	2.21	-25049.18	6.30	-25002.77	9.18	-24953.54	14.89	-24904.67	20.22	-24883.91	19.22
	-25144.26	-1.73	-25098.22	0.78	-25049.96	5.52	-25002.68	9.27	-24954.95	13.47	-24906.77	18.12	-24881.21	21.92
	-25145.07	-2.53	-25095.70	3.30	-25048.12	7.36	-25002.78	9.17	-24955.34	13.08	-24908.75	16.14	-24884.34	18.79
	-25143.56	-1.02	-25097.29	1.71	-25049.05	6.43	-25002.56	9.39	-24953.38	15.04	-24885.60	39.29	-24877.22	25.91
	-25145.63	-3.10	-25095.43	3.57	-25048.99	6.48	-25000.54	11.40	-24952.30	16.12	-24899.36	25.54	-24884.24	18.88
	-25143.56	-1.03	-25096.92	2.08	-25047.68	7.80	-25000.49	11.46	-24947.78	20.64	-24908.49	16.41	-24884.15	18.98
	-25145.24	-2.71	-25097.56	1.44	-25048.15	7.33	-25003.79	8.16	-24949.48	18.94	-24906.67	18.22	-24878.22	24.91
	-25141.35	1.19	-25096.56	2.44	-25044.16	11.31	-24998.52	13.43	-24955.59	12.83	-24905.13	19.77	-24882.35	20.78
	-25143.61	-1.08	-25097.88	1.13	-25048.43	7.05	-25001.83	10.12	-24955.21	13.21	-24904.65	20.24	-24883.93	19.20
	-25143.40	-0.87	-25095.13	3.87	-25047.61	7.86	-24998.31	13.64	-24955.74	12.68	-24906.42	18.48	-24886.18	16.95
	-25143.77	-1.24	-25097.53	1.47	-25051.39	4.08	-25003.01	8.94	-24958.12	10.30	-24899.47	25.42	-24880.30	22.83
	-25144.56	-2.03	-25096.07	2.94	-25049.02	6.45	-25003.49	8.46	-24950.32	18.10	-24904.52	20.38	-24885.73	17.40
	-25143.64	-1.10	-25096.27	2.73	-25050.20	5.27	-24998.91	13.04	-24953.72	14.70	-24900.51	24.38	-24883.79	19.34

-25142.96	-0.43	-25097.92	1.09	-25051.36	4.11	-24999.57	12.37	-24954.69	13.73	-24902.83	22.06	-24883.32	19.81
-25144.22	-1.69	-25096.24	2.77	-25049.73	5.75	-25000.82	11.13	-24954.57	13.85	-24903.56	21.33	-24885.05	18.08
-25145.02	-2.48	-25094.86	4.15	-25049.66	5.82	-25001.76	10.18	-24952.77	15.65	-24908.72	16.18	-24884.01	19.12
-25145.12	-2.59	-25095.65	3.36	-25049.80	5.67	-24999.62	12.33	-24911.17	57.25	-24885.59	39.30	-24885.95	17.18
-25144.33	-1.79	-25098.40	0.60	-25049.58	5.90	-25002.59	9.36	-24954.62	13.81	-24907.80	17.09	-24875.95	27.18
-25145.03	-2.50	-25096.79	2.21	-25051.22	4.26	-25000.06	11.89	-24951.38	17.04	-24900.83	24.06	-24883.49	19.64
-25144.72	-2.19	-25094.43	4.58	-25050.53	4.94	-25000.17	11.78	-24955.43	12.99	-24905.99	18.90	-24884.03	19.10
-25144.79	-2.25	-25098.22	0.79	-25050.35	5.13	-25000.42	11.53	-24953.94	14.48	-24907.34	17.56	-24881.63	21.50
-25144.29	-1.76	-25096.49	2.52	-25050.48	5.00	-25001.20	10.74	-24955.38	13.04	-24897.86	27.03	-24885.53	17.60
-25143.83	-1.30	-25098.67	0.33	-25048.74	6.74	-25000.10	11.84	-24953.59	14.83	-24904.19	20.70	-24885.34	17.78
-25143.91	-1.37	-25096.85	2.15	-25045.78	9.70	-24996.54	15.41	-24947.64	20.78	-24909.84	15.05	-24881.73	21.40
-25144.30	-1.77	-25098.68	0.33	-25050.05	5.42	-25001.01	10.94	-24954.15	14.27	-24904.33	20.56	-24880.13	23.00
-25142.51	0.03	-25097.74	1.26	-25049.03	6.45	-25004.50	7.45	-24957.36	11.06	-24907.38	17.51	-24885.14	17.98
-25144.51	-1.97	-25097.54	1.46	-25048.51	6.97	-24999.72	12.23	-24954.57	13.85	-24907.59	17.30	-24881.01	22.12
-25145.14	-2.61	-25097.30	1.70	-25050.43	5.04	-25001.74	10.21	-24956.92	11.50	-24905.53	19.36	-24879.28	23.85
-25144.01	-1.48	-25098.50	0.51	-25049.54	5.94	-24998.34	13.61	-24955.73	12.69	-24910.11	14.78	-24885.70	17.43
-25144.04	-1.51	-25092.55	6.45	-25049.73	5.75	-25003.29	8.66	-24955.39	13.03	-24907.70	17.19	-24881.80	21.33
-25144.22	-1.69	-25096.31	2.70	-25048.59	6.89	-25000.72	11.22	-24955.30	13.13	-24906.46	18.43	-24882.29	20.84
-25144.38	-1.84	-25097.34	1.67	-25046.80	8.68	-24999.71	12.24	-24956.26	12.16	-24905.04	19.85	-24882.80	20.33
-25143.88	-1.35	-25096.15	2.85	-25047.97	7.50	-25001.27	10.68	-24952.88	15.54	-24907.44	17.45	-24882.14	20.99
-25144.00	-1.46	-25096.85	2.15	-25047.22	8.26	-25000.24	11.71	-24952.86	15.56	-24905.28	19.61	-24880.39	22.74
-25143.94	-1.41	-25096.77	2.24	-25050.81	4.66	-25002.26	9.69	-24957.82	10.60	-24902.68	22.21	-24883.26	19.87
-25143.78	-1.24	-25096.94	2.06	-25048.97	6.50	-25003.51	8.44	-24950.59	17.83	-24902.62	22.27	-24881.34	21.79
-25144.06	-1.52	-25096.82	2.18	-25049.33	6.15	-25002.78	9.17	-24957.65	10.77	-24906.56	18.33	-24872.14	30.99
-25144.42	-1.88	-25095.26	3.74	-25047.74	7.74	-25000.12	11.83	-24955.63	12.79	-24883.45	41.44	-24881.81	21.32
-25144.06	-1.53	-25096.48	2.53	-25044.96	10.52	-24953.74	58.21	-24955.86	12.56	-24906.16	18.73	-24879.72	23.40
-25144.10	-1.56	-25097.79	1.21	-25048.05	7.43	-24998.76	13.19	-24902.76	65.66	-24902.47	22.42	-24876.14	26.99
-25145.30	-2.77	-25093.63	5.38	-25049.43	6.05	-24999.74	12.21	-24950.84	17.58	-24908.68	16.21	-24884.24	18.88
-25143.83	-1.29	-25095.07	3.93	-25049.63	5.84	-24997.66	14.29	-24952.95	15.47	-24905.55	19.34	-24886.26	16.87
-25143.92	-1.39	-25094.82	4.18	-25051.13	4.35	-25002.40	9.55	-24953.20	15.22	-24906.72	18.17	-24882.22	20.91
-25143.31	-0.78	-25096.24	2.76	-25047.77	7.71	-25002.31	9.63	-24954.19	14.23	-24904.76	20.14	-24884.35	18.78
-25144.83	-2.30	-25097.10	1.90	-25048.98	6.50	-25004.93	7.02	-24954.55	13.87	-24906.86	18.03	-24885.21	17.92
-25143.91	-1.38	-25094.88	4.13	-25048.34	7.14	-25000.34	11.61	-24954.19	14.23	-24907.74	17.15	-24883.70	19.43
-25144.38	-1.84	-25094.93	4.08	-25051.21	4.27	-25003.19	8.76	-24955.12	13.30	-24902.68	22.21	-24885.11	18.01
-25141.06	1.47	-25098.12	0.88	-25049.74	5.74	-25002.80	9.15	-24957.13	11.29	-24909.73	15.16	-24883.28	19.85
-25144.33	-1.80	-25096.72	2.29	-25046.98	8.49	-24999.09	12.86	-24956.41	12.01	-24901.64	23.25	-24882.51	20.62
-25143.62	-1.09	-25096.07	2.94	-25048.71	6.77	-25001.33	10.62	-24955.32	13.10	-24906.70	18.19	-24883.99	19.14
-25144.60	-2.07	-25096.89	2.11	-25049.09	6.39	-25002.39	9.56	-24952.16	16.26	-24906.60	18.29	-24879.66	23.46
-25143.26	-0.72	-25097.02	1.99	-25051.29	4.18	-24999.18	12.77	-24956.24	12.18	-24906.14	18.75	-24884.44	18.69
-25143.29	-0.75	-25098.31	0.69	-25049.27	6.21	-25002.07	9.88	-24953.83	14.59	-24897.57	27.32	-24880.42	22.71
-25143.41	-0.87	-25097.43	1.58	-25049.06	6.42	-25003.36	8.59	-24947.66	20.76	-24905.22	19.67	-24886.66	16.47

-25143.88	-1.34	-25093.01	5.99	-25048.11	7.37	-25001.22	10.73	-24954.47	13.95	-24908.89	16.00	-24885.81	17.31
-25144.00	-1.47	-25097.18	1.82	-25050.23	5.25	-24996.53	15.42	-24951.48	16.94	-24909.09	15.80	-24879.64	23.48
-25146.12	-3.59	-25098.10	0.91	-25048.76	6.72	-24999.45	12.50	-24956.95	11.47	-24908.47	16.43	-24879.85	23.28
-25144.77	-2.23	-25094.84	4.16	-25049.57	5.91	-25000.77	11.18	-24953.24	15.18	-24906.47	18.42	-24884.31	18.82
-25144.64	-2.11	-25098.09	0.91	-25049.24	6.23	-25002.45	9.50	-24951.62	16.80	-24906.37	18.52	-24883.37	19.76
-25144.61	-2.07	-25096.37	2.64	-25048.16	7.31	-25000.28	11.67	-24954.02	14.40	-24903.28	21.61	-24880.63	22.50
-25143.47	-0.93	-25097.05	1.95	-25049.72	5.76	-24995.88	16.07	-24951.51	16.91	-24907.87	17.02	-24881.57	21.56
-25144.50	-1.97	-25097.38	1.63	-25051.01	4.47	-25002.04	9.91	-24955.88	12.54	-24901.30	23.59	-24885.38	17.75
-25143.62	-1.09	-25097.18	1.82	-25048.86	6.61	-24999.66	12.29	-24947.82	20.60	-24906.87	18.03	-24883.21	19.92
-25144.74	-2.21	-25096.40	2.61	-25048.61	6.87	-25000.63	11.32	-24956.22	12.20	-24903.48	21.42	-24880.07	23.06
-25144.03	-1.50	-25096.95	2.05	-25051.32	4.15	-24996.59	15.36	-24952.80	15.62	-24904.73	20.16	-24883.14	19.98
-25144.22	-1.69	-25097.97	1.03	-25047.70	7.78	-25002.54	9.41	-24952.49	15.93	-24906.62	18.27	-24881.99	21.14
-25144.49	-1.96	-25096.30	2.71	-25048.69	6.79	-24997.77	14.18	-24955.61	12.81	-24906.33	18.56	-24883.42	19.71
-25143.08	-0.55	-25096.28	2.73	-25051.01	4.47	-25004.31	7.64	-24951.95	16.47	-24904.86	20.03	-24883.82	19.31
-25144.86	-2.33	-25095.42	3.59	-25049.11	6.36	-25001.88	10.07	-24955.43	12.99	-24909.74	15.15	-24886.16	16.97
-25144.87	-2.34	-25096.24	2.77	-25043.36	12.12	-25000.24	11.70	-24953.07	15.35	-24906.88	18.01	-24876.60	26.52
-25144.90	-2.37	-25096.01	2.99	-25049.60	5.88	-24999.70	12.25	-24951.73	16.69	-24903.56	21.33	-24883.42	19.70
-25145.17	-2.64	-25096.46	2.55	-25051.93	3.55	-25002.91	9.04	-24951.44	16.98	-24907.02	17.88	-24875.94	27.19
-25143.27	-0.73	-25096.64	2.36	-25046.53	8.95	-25003.56	8.39	-24953.02	15.40	-24908.17	16.72	-24869.17	33.96
-25143.68	-1.14	-25096.75	2.25	-25051.86	3.62	-25000.34	11.61	-24953.83	14.59	-24906.58	18.31	-24883.40	19.73
-25143.02	-0.49	-25095.82	3.19	-25050.96	4.52	-25005.24	6.71	-24953.59	14.83	-24908.82	16.07	-24886.24	16.89
-25143.10	-0.57	-25097.05	1.95	-25049.44	6.04	-24998.16	13.79	-24949.84	18.58	-24901.85	23.05	-24881.00	22.13
-25143.34	-0.80	-25095.12	3.89	-25047.58	7.90	-24999.23	12.72	-24953.04	15.38	-24909.29	15.60	-24879.36	23.76
-25143.76	-1.23	-25097.83	1.18	-25050.47	5.01	-24997.59	14.36	-24954.90	13.52	-24908.86	16.04	-24882.31	20.82
-25144.90	-2.37	-25096.49	2.52	-25045.54	9.93	-25002.79	9.16	-24951.09	17.33	-24906.34	18.55	-24879.44	23.69
-25144.96	-2.43	-25095.98	3.03	-25048.67	6.81	-25003.24	8.71	-24955.29	13.13	-24907.68	17.21	-24882.99	20.13
-25143.38	-0.84	-25097.93	1.08	-25049.81	5.67	-25001.40	10.55	-24955.14	13.28	-24909.06	15.83	-24877.27	25.86
-25144.15	-1.62	-25094.05	4.95	-25048.65	6.82	-25001.53	10.42	-24954.23	14.19	-24908.35	16.54	-24886.38	16.75
-25144.95	-2.42	-25093.34	5.66	-25049.33	6.15	-25003.99	7.96	-24956.83	11.59	-24908.48	16.41	-24883.74	19.39
-25143.57	-1.04	-25096.70	2.30	-25047.19	8.28	-25000.28	11.67	-24954.92	13.50	-24901.88	23.01	-24879.30	23.83
-25143.46	-0.92	-25096.50	2.51	-25047.77	7.71	-25002.52	9.43	-24951.16	17.26	-24905.03	19.86	-24874.86	28.27
-25143.68	-1.15	-25096.84	2.17	-25048.78	6.70	-25000.38	11.56	-24952.48	15.94	-24876.69	48.21	-24882.43	20.69
-25142.86	-0.33	-25095.03	3.98	-25050.32	5.16	-25000.63	11.32	-24956.26	12.16	-24907.69	17.20	-24884.73	18.39
-25144.07	-1.53	-25096.39	2.62	-25049.69	5.79	-25001.28	10.67	-24956.20	12.23	-24904.15	20.74	-24881.47	21.66
-25144.39	-1.85	-25094.73	4.28	-25050.22	5.26	-24998.21	13.74	-24953.71	14.71	-24907.50	17.39	-24882.92	20.21
-25144.90	-2.37	-25097.99	1.02	-25048.67	6.80	-24996.60	15.35	-24955.40	13.02	-24906.67	18.23	-24878.06	25.07
-25143.98	-1.45	-25097.99	1.01	-25049.98	5.49	-25001.25	10.69	-24956.46	11.96	-24906.24	18.65	-24883.38	19.74
-25144.53	-2.00	-25097.85	1.16	-25049.91	5.56	-25003.07	8.88	-24948.48	19.94	-24903.56	21.33	-24882.51	20.62
-25143.83	-1.29	-25098.45	0.55	-25048.89	6.58	-24999.43	12.52	-24951.29	17.13	-24905.87	19.03	-24880.65	22.48
-25144.40	-1.87	-25095.60	3.41	-25049.28	6.19	-25002.29	9.66	-24954.35	14.07	-24903.91	20.99	-24878.45	24.68
-25144.18	-1.64	-25095.74	3.27	-25049.36	6.12	-25003.34	8.61	-24954.06	14.36	-24904.75	20.14	-24882.00	21.12

	-25143.98	-1.44	-25096.04	2.96	-25049.14	6.34	-25003.33	8.62	-24954.11	14.31	-24908.79	16.10	-24878.72	24.41
	-25143.60	-1.07	-25096.91	2.10	-25048.84	6.64	-25003.05	8.90	-24954.72	13.70	-24906.41	18.48	-24878.55	24.58
	-25144.58	-2.05	-25098.27	0.73	-25050.04	5.44	-25002.51	9.44	-24952.20	16.22	-24908.58	16.32	-24887.94	15.19
	-25144.06	-1.52	-25095.54	3.46	-25087.96	-32.48	-25002.24	9.71	-24956.75	11.67	-24905.97	18.92		
	-25144.50	-1.97	-25097.43	1.57	-25048.71	6.77	-25001.27	10.68	-24957.46	10.96	-24908.68	16.22		
	-25144.80	-2.27	-25096.77	2.23	-25048.51	6.97	-25000.61	11.34	-24951.31	17.11	-24906.21	18.68		
	-25143.57	-1.04	-25095.64	3.37	-25049.47	6.01	-25002.41	9.54	-24950.33	18.09	-24907.38	17.51		
	-25144.18	-1.65	-25094.73	4.28	-25048.10	7.38	-25002.56	9.39	-24954.89	13.53	-24907.99	16.90		
	-25144.33	-1.80	-25097.45	1.55	-25047.60	7.87	-24998.93	13.02	-24952.93	15.49	-24906.14	18.75		
	-25143.21	-0.68	-25095.66	3.35	-25047.37	8.10	-25000.01	11.94	-24952.85	15.58	-24908.81	16.09		
	-25143.50	-0.96	-25096.57	2.43	-25049.33	6.15	-24997.63	14.32	-24953.58	14.84	-24907.13	17.76		
	-25143.78	-1.25	-25097.35	1.66	-25045.93	9.55	-25000.42	11.52	-24952.89	15.53	-24908.38	16.51		
	-25144.64	-2.11	-25097.74	1.26	-25048.52	6.96	-25002.54	9.41	-24954.49	13.93	-24908.49	16.41		
	-25142.88	-0.34	-25096.63	2.38	-25048.04	7.44	-24998.12	13.82	-24954.39	14.03	-24907.94	16.95		
	-25144.46	-1.92			-25051.10	4.38	-25000.57	11.38	-24956.45	11.97				
	-25144.10	-1.57			-25045.43	10.05	-25002.57	9.38	-24954.70	13.73				
	-25144.51	-1.97			-25048.97	6.51	-24999.93	12.02	-24950.68	17.74				
	-25146.16	-3.63							-24956.32	12.10				
Average energies	-25144.09	-1.56	-25096.56	2.45	-25049.33	6.15	-25000.99	10.96	-24953.04	15.38	-24905.17	19.72	-24882.03	21.10

U

	ground state energy	solid solution energy												
concentration in solid solution (%)	3		6		9		12		15		18		21	
number of atoms	2		4		6		8		10		12		13	
	-25146.74	0.81	-25107.14	1.90	-25063.49	7.04	-25021.55	10.47	-24971.24	22.28	-24941.23	13.77	-24912.96	22.79
	-25148.53	-0.98	-25103.81	5.23	-25062.35	8.18	-25021.44	10.58	-24978.03	15.49	-24935.22	19.78	-24908.04	27.71
	-25147.43	0.13	-25107.73	1.31	-25062.38	8.15	-25021.21	10.82	-24981.62	11.90	-24931.25	23.76	-24913.55	22.20
	-25149.52	-1.96	-25105.86	3.18	-25066.22	4.31	-25021.16	10.87	-24975.62	17.89	-24935.35	19.65	-24917.59	18.16
	-25149.44	-1.89	-25105.68	3.36	-25063.81	6.73	-25020.33	11.70	-24981.50	12.01	-24936.81	18.20	-24910.10	25.65
	-25147.35	0.20	-25101.73	7.31	-25062.10	8.43	-25023.08	8.94	-24982.66	10.85	-24933.85	21.15	-24915.32	20.43
	-25146.71	0.84	-25106.55	2.49	-25063.29	7.24	-25021.93	10.09	-24975.64	17.87	-24939.38	15.63	-24909.10	26.65
	-25147.06	0.49	-25106.99	2.05	-25063.05	7.49	-25023.95	8.08	-24981.78	11.74	-24938.59	16.42	-24906.15	29.60
	-25148.40	-0.84	-25104.49	4.55	-25062.75	7.79	-25018.40	13.62	-24977.29	16.22	-24936.04	18.97	-24915.07	20.68
	-25146.92	0.64	-25103.66	5.38	-25062.97	7.56	-25022.10	9.92	-24976.46	17.05	-24938.30	16.71	-24917.62	18.13
	-25148.94	-1.39	-25103.24	5.80	-25062.93	7.61	-25022.31	9.72	-24974.55	18.96	-24938.63	16.38	-24917.49	18.26
	-25148.88	-1.32	-25103.44	5.61	-25064.70	5.83	-25021.86	10.17	-24979.41	14.10	-24939.74	15.27	-24912.64	23.11
	-25150.38	-2.83	-25107.04	2.00	-25064.47	6.07	-25019.00	13.02	-24975.94	17.58	-24933.91	21.10	-24916.10	19.65
	-25146.21	1.34	-25108.76	0.28	-25064.59	5.94	-25020.38	11.64	-24978.04	15.48	-24940.11	14.89	-24916.30	19.45

-25148.18	-0.62	-25104.76	4.28	-25065.63	4.90	-25021.12	10.91	-24977.55	15.97	-24937.67	17.33	-24912.52	23.23
-25147.19	0.37	-25104.80	4.24	-25065.17	5.37	-25019.97	12.06	-24978.55	14.97	-24932.64	22.37	-24907.27	28.48
-25149.03	-1.47	-25106.88	2.16	-25063.71	6.82	-25020.46	11.57	-24978.91	14.61	-24939.00	16.00	-24919.44	16.31
-25143.32	4.23	-25105.79	3.25	-25066.56	3.98	-25021.85	10.18	-24980.23	13.28	-24935.45	19.55	-24917.29	18.46
-25148.77	-1.22	-25105.33	3.72	-25064.85	5.68	-25020.60	11.42	-24978.31	15.20	-24936.12	18.88	-24916.85	18.90
-25149.76	-2.21	-25105.22	3.82	-25063.84	6.70	-25020.72	11.30	-24978.15	15.37	-24939.54	15.46	-24916.82	18.93
-25148.87	-1.31	-25105.06	3.99	-25063.96	6.58	-25020.20	11.83	-24978.61	14.90	-24935.87	19.13	-24916.13	19.62
-25147.51	0.04	-25103.70	5.34	-25062.18	8.35	-25023.49	8.53	-24978.31	15.21	-24934.94	20.06	-24914.47	21.28
-25141.47	6.08	-25106.61	2.43	-25062.82	7.72	-25020.85	11.18	-24976.34	17.18	-24937.73	17.27	-24914.63	21.12
-25148.73	-1.17	-25104.55	4.50	-25063.48	7.05	-25020.07	11.96	-24979.02	14.49	-24934.83	20.17	-24912.05	23.69
-25149.00	-1.44	-25095.03	14.02	-25063.71	6.83	-25019.11	12.92	-24979.39	14.13	-24940.53	14.47	-24916.97	18.78
-25149.65	-2.10	-25106.61	2.43	-25063.16	7.37	-25020.59	11.43	-24980.02	13.49	-24939.17	15.84	-24916.01	19.74
-25148.61	-1.06	-25098.26	10.78	-25063.83	6.71	-25021.60	10.43	-24971.20	22.32	-24938.82	16.18	-24913.03	22.72
-25148.32	-0.77	-25105.41	3.63	-25063.89	6.64	-25021.27	10.75	-24978.32	15.19	-24934.97	20.03	-24912.74	23.01
-25149.41	-1.86	-25106.24	2.80	-25063.11	7.42	-25018.55	13.47	-24979.50	14.01	-24939.07	15.93	-24918.19	17.56
-25149.85	-2.29	-25105.43	3.61	-25065.47	5.06	-25020.49	11.53	-24981.54	11.98	-24936.06	18.94	-24914.88	20.87
-25148.29	-0.73	-25104.03	5.01	-25062.20	8.33	-25007.52	24.50	-24977.87	15.65	-24937.30	17.71	-24910.40	25.35
-25147.41	0.15	-25103.37	5.67	-25062.02	8.51	-25018.99	13.03	-24979.92	13.60	-24931.48	23.53	-24916.20	19.55
-25146.86	0.69	-25106.19	2.85	-25063.64	6.89	-25021.68	10.34	-24978.30	15.21	-24938.00	17.01	-24918.97	16.78
-25147.93	-0.37	-25107.85	1.19	-25064.59	5.94	-25024.32	7.71	-24978.91	14.61	-24933.56	21.45	-24915.31	20.44
-25146.56	0.99	-25105.81	3.24	-25061.06	9.48	-25018.80	13.23	-24975.80	17.71	-24935.95	19.05	-24918.43	17.32
-25148.21	-0.66	-25105.47	3.57	-25065.32	5.22	-25023.96	8.06	-24981.19	12.33	-24938.06	16.94	-24912.70	23.05
-25148.53	-0.98	-25102.51	6.53	-25062.22	8.32	-25022.45	9.57	-24977.87	15.64	-24932.33	22.68	-24910.00	25.75
-25149.02	-1.47	-25104.52	4.52	-25066.57	3.97	-25014.96	17.07	-24979.57	13.94	-24938.31	16.70	-24912.58	23.17
-25149.49	-1.94	-25105.12	3.93	-25064.63	5.91	-25023.76	8.26	-24975.73	17.79	-24929.96	25.04	-24913.88	21.87
-25139.55	8.00	-25104.73	4.31	-25063.65	6.88	-25022.12	9.91	-24977.91	15.60	-24992.98	-37.98	-24917.55	18.20
-25150.66	-3.11	-25105.90	3.14	-25063.79	6.75	-25023.43	8.59	-24980.05	13.47	-24938.81	16.19	-24917.86	17.89
-25148.61	-1.06	-25103.39	5.65	-25065.06	5.47	-25020.14	11.89	-24976.94	16.58	-24933.39	21.61	-24917.21	18.54
-25147.73	-0.18	-25105.74	3.31	-25066.48	4.05	-25023.65	8.38	-24975.13	18.39	-24938.65	16.36	-24919.29	16.45
-25147.92	-0.37	-25104.81	4.24	-25060.93	9.60	-25021.16	10.86	-24977.28	16.24	-24937.41	17.60	-24914.38	21.37
-25146.29	1.26	-25104.92	4.13	-25065.28	5.25	-25015.43	16.60	-24975.90	17.61	-24934.44	20.56	-24914.23	21.52
-25148.50	-0.94	-25105.29	3.75	-25065.64	4.89	-25023.67	8.35	-24980.28	13.23	-24937.54	17.46	-24919.33	16.42
-25147.68	-0.13	-25106.60	2.45	-25063.59	6.94	-25022.11	9.91	-24974.40	19.11	-24939.46	15.54	-24916.02	19.73
-25148.62	-1.07	-25106.50	2.55	-25060.07	10.46	-25021.80	10.22	-24971.26	22.25	-24939.72	15.28	-24914.47	21.28
-25147.48	0.08	-25106.54	2.51	-25094.47	-23.94	-25021.96	10.06			-24937.48	17.53	-24912.04	23.71
-25148.08	-0.53	-25105.43	3.61	-25063.31	7.22					-24934.35	20.65	-24916.04	19.71
-25148.61	-1.06	-25105.05	3.99	-25064.11	6.42					-24935.82	19.19	-24919.25	16.50
-25149.15	-1.59	-25105.66	3.39	-25063.71	6.83					-24935.39	19.61	-24909.90	25.85
-25147.49	0.06	-25106.05	2.99	-25063.60	6.94					-24938.77	16.23	-24913.13	22.62
-25148.87	-1.32	-25095.94	13.11							-24936.06	18.94	-24913.33	22.42
-25148.19	-0.63	-25104.08	4.96							-24937.68	17.32	-24912.39	23.36

	-25149.03	-1.47	-25106.71	2.34							-24934.29	20.72		
	-25147.25	0.30	-25105.49	3.55							-24937.71	17.30		
	-25147.24	0.31	-25106.18	2.87										
	-25136.89	10.66	-25104.03	5.01										
	-25148.74	-1.19	-25106.56	2.49										
	-25146.91	0.65	-25106.65	2.39										
	-25147.25	0.31	-25106.26	2.79										
	-25148.25	-0.70	-25104.73	4.31										
	-25146.12	1.44	-25085.09	23.96										
	-25139.90	7.65	-25105.10	3.94										
	-25146.61	0.95	-25108.35	0.70										
	-25149.44	-1.89												
	-25148.13	-0.58												
	-25146.78	0.77												
	-25138.71	8.84												
	-25145.50	2.06												
	-25147.63	-0.08												
	-25148.61	-1.05												
	-25148.44	-0.89												
Average energies	-25147.48	0.08	-25104.73	4.31	-25064.35	6.19	-25020.85	11.18	-24977.88	15.64	-24937.64	17.36	-24914.59	21.16

$Ca_{1-x}M_xZrTi_{2-2x}Fe_{2x}O_7$ M=(Ce, Pu, Th, U)

Ti(2)Ti(2)

N.B. All stated energies are in eV

Ce

	ground state energy	solid solution energy												
concentration in solid solution (%)	3		6		9		12		15		18		21	
number of atoms	2		4		6		8		10		12		13	
	-25147.37	-4.48	-25105.58	-5.86	-25064.99	-8.45	-25020.47	-7.10	-24977.94	-7.74	-24940.37	-13.34	-24917.12	-11.69
	-25147.84	-4.95	-25106.06	-6.34	-25059.05	-2.51	-25021.72	-8.35	-24979.80	-9.60	-24939.74	-12.72	-24913.42	-7.98
	-25147.30	-4.41	-25104.32	-4.61	-25063.26	-6.72	-25021.26	-7.89	-24977.14	-6.95	-24939.77	-12.75	-24917.36	-11.93
	-25147.82	-4.93	-25105.39	-5.68	-25062.21	-5.67	-25020.49	-7.12	-24977.62	-7.42	-24937.04	-10.02	-24914.96	-9.53
	-25148.37	-5.48	-25105.12	-5.40	-25062.82	-6.28	-25019.06	-5.69	-24976.29	-6.10	-24938.55	-11.53	-24913.94	-8.50
	-25147.92	-5.03	-25104.03	-4.32	-25062.15	-5.61	-25022.71	-9.34	-24980.09	-9.89	-24935.27	-8.25	-24915.18	-9.74
	-25148.25	-5.37	-25103.91	-4.19	-25063.79	-7.24	-25021.33	-7.96	-24974.88	-4.69	-24979.19	-52.17	-24919.00	-13.56
	-25148.95	-6.07	-25104.49	-4.77	-25062.08	-5.54	-25021.19	-7.82	-24978.94	-8.75	-24934.12	-7.10	-24917.44	-12.00
	-25148.01	-5.12	-25106.10	-6.38	-25062.65	-6.11	-25019.35	-5.98	-24976.79	-6.60	-24940.44	-13.42	-24917.01	-11.57
	-25148.55	-5.66	-25105.82	-6.10	-25063.18	-6.63	-25018.36	-4.99	-24979.90	-9.70	-24936.65	-9.63	-24917.19	-11.76
	-25146.96	-4.07	-25104.67	-4.96	-25064.13	-7.59	-25021.00	-7.63	-24979.07	-8.88	-24937.61	-10.59	-24909.38	-3.94
	-25147.47	-4.59	-25104.64	-4.92	-25063.44	-6.89	-25020.82	-7.45	-24979.34	-9.14	-24938.91	-11.89	-24919.05	-13.62
	-25147.77	-4.89	-25105.78	-6.07	-25061.70	-5.16	-25022.23	-8.86	-24978.94	-8.74	-24934.92	-7.89	-24915.02	-9.59
	-25149.07	-6.18	-25106.70	-6.98	-25064.54	-8.00	-25020.54	-7.18	-24980.40	-10.20	-24937.13	-10.10	-24915.25	-9.81
	-25148.55	-5.66	-25106.40	-6.68	-25062.59	-6.05	-25022.78	-9.41	-24978.84	-8.64	-24938.14	-11.12	-24917.42	-11.98
	-25148.77	-5.88	-25106.76	-7.05	-25062.40	-5.86	-25022.81	-9.44	-24978.62	-8.43	-24934.19	-7.17	-24915.74	-10.30
	-25148.75	-5.86	-25106.38	-6.66	-25062.83	-6.29	-25020.99	-7.62	-24977.05	-6.85	-24938.56	-11.54	-24915.04	-9.61
	-25147.26	-4.37	-25106.03	-6.31	-25063.14	-6.59	-25019.43	-6.06	-24979.75	-9.55	-24932.92	-5.90	-24916.90	-11.47
	-25149.95	-7.06	-25105.23	-5.51	-25061.85	-5.31	-25019.18	-5.81	-24980.04	-9.84	-24939.12	-12.10	-24913.85	-8.41
	-25149.79	-6.90	-25106.14	-6.43	-25063.55	-7.01	-25020.26	-6.89	-24977.56	-7.36	-24936.98	-9.96	-24914.10	-8.66
	-25148.48	-5.59	-25105.43	-5.71	-25063.21	-6.67	-25020.39	-7.03	-24978.10	-7.91	-24938.01	-10.99	-24915.49	-10.06
	-25148.90	-6.01	-25100.85	-1.13	-25062.49	-5.95	-25020.44	-7.07	-24978.75	-8.56	-24932.05	-5.03	-24917.10	-11.66
	-25148.09	-5.20	-25106.27	-6.55	-25062.84	-6.30	-25019.10	-5.73	-24980.86	-10.66	-24932.27	-5.25	-24916.34	-10.90
	-25147.75	-4.86	-25106.78	-7.07	-25058.24	-1.70	-25019.91	-6.54	-24977.91	-7.71	-24937.09	-10.07	-24915.50	-10.06
	-25147.70	-4.81	-25107.19	-7.47	-25062.01	-5.47	-25020.31	-6.94	-24979.97	-9.78	-24937.64	-10.62	-24912.90	-7.46
	-25147.87	-4.98	-25106.19	-6.47	-25064.05	-7.50	-25021.86	-8.49	-24981.26	-11.07	-24933.13	-6.11	-24915.53	-10.10
	-25148.56	-5.67	-25102.19	-2.47	-25063.11	-6.57	-25021.58	-8.22	-24980.16	-9.96	-24935.39	-8.37	-24921.22	-15.78
	-25146.65	-3.76	-25103.39	-3.68	-25062.48	-5.93	-25022.06	-8.69	-24977.56	-7.36	-24937.57	-10.55	-24916.75	-11.32
	-25148.93	-6.04	-25105.70	-5.98	-25064.51	-7.97	-25015.12	-1.76	-24977.03	-6.83	-24938.93	-11.91	-24918.59	-13.15

-25148.58	-5.69	-25105.36	-5.65	-25061.49	-4.95	-25022.15	-8.78	-24979.46	-9.26	-24937.16	-10.14	-24912.96	-7.52
-25148.58	-5.69	-25105.90	-6.18	-25063.73	-7.19	-25019.77	-6.40	-24980.81	-10.62	-24932.08	-5.06	-24916.54	-11.10
-25149.08	-6.19	-25104.97	-5.25	-25064.46	-7.92	-25021.88	-8.51	-24976.87	-6.67	-24937.89	-10.87	-24913.22	-7.78
-25148.71	-5.82	-25106.16	-6.45	-25064.23	-7.68	-25021.49	-8.12	-24976.60	-6.40	-24938.00	-10.98	-24917.76	-12.33
-25148.76	-5.87	-25105.51	-5.79	-25062.11	-5.57	-25020.99	-7.62	-24979.15	-8.96	-24939.90	-12.88	-24916.21	-10.77
-25148.04	-5.15	-25104.22	-4.50	-25062.58	-6.04	-25020.83	-7.46	-24981.52	-11.33	-24937.25	-10.22	-24915.32	-9.89
-25147.73	-4.84	-25103.44	-3.73	-25063.49	-6.94	-25022.47	-9.10	-24977.45	-7.25	-24936.18	-9.16	-24912.51	-7.08
-25148.09	-5.20	-25106.12	-6.41	-25062.16	-5.62	-25019.85	-6.48	-24973.72	-3.53	-24937.18	-10.16	-24914.65	-9.21
-25149.65	-6.76	-25107.43	-7.71	-25064.52	-7.98	-25019.92	-6.55	-24977.60	-7.41	-24937.75	-10.73	-24916.10	-10.67
-25148.11	-5.22	-25107.12	-7.40	-25063.19	-6.65	-25016.76	-3.40	-24977.80	-7.60	-24934.54	-7.52	-24916.25	-10.81
-25147.50	-4.61	-25105.12	-5.41	-25063.02	-6.47	-25020.16	-6.79	-24979.18	-8.98	-24934.92	-7.89	-24918.13	-12.69
-25147.74	-4.85	-25105.40	-5.69	-25061.84	-5.30	-25020.62	-7.26	-24980.08	-9.89	-24935.97	-8.94	-24918.40	-12.97
-25148.08	-5.19	-25107.38	-7.66	-25063.72	-7.18	-25019.68	-6.31	-24981.89	-11.69	-24935.49	-8.47	-24914.90	-9.46
-25149.41	-6.52	-25105.20	-5.48	-25064.35	-7.81	-25023.86	-10.49	-24979.92	-9.72	-24940.76	-13.74	-24918.42	-12.98
-25148.33	-5.44	-25105.33	-5.62	-25064.32	-7.78	-25021.24	-7.87	-24980.27	-10.07	-24936.91	-9.89	-24917.78	-12.35
-25148.92	-6.03	-25106.77	-7.06	-25064.01	-7.47	-25020.85	-7.48	-24978.38	-8.19	-24939.77	-12.75	-24916.05	-10.62
-25148.82	-5.93	-25106.02	-6.30	-25063.19	-6.64	-25019.38	-6.01	-24979.76	-9.57	-24938.99	-11.97	-24917.98	-12.55
-25148.95	-6.06	-25106.64	-6.92	-25065.98	-9.44	-25022.90	-9.53	-24979.31	-9.12	-24938.85	-11.83	-24918.52	-13.09
-25149.89	-7.00	-25106.31	-6.60	-25064.10	-7.56	-25020.43	-7.06	-24980.18	-9.98	-24939.14	-12.12	-24917.42	-11.98
-25148.69	-5.80	-25106.44	-6.73	-25064.49	-7.95	-25019.12	-5.75	-24978.26	-8.07	-24936.49	-9.47	-24915.10	-9.67
-25149.05	-6.16	-25107.11	-7.40	-25063.57	-7.03	-25020.92	-7.56	-24977.66	-7.47	-24935.14	-8.12	-24916.72	-11.28
-25148.13	-5.25	-25105.62	-5.91	-25064.24	-7.69	-25020.60	-7.23	-24980.31	-10.12	-24940.66	-13.64	-24918.61	-13.18
-25148.72	-5.83	-25103.69	-3.98	-25064.74	-8.20	-25021.51	-8.14	-24980.04	-9.84	-24934.58	-7.56	-24911.23	-5.80
-25146.98	-4.10	-25106.97	-7.26	-25063.84	-7.30	-25023.01	-9.65	-24979.17	-8.98	-24938.34	-11.32	-24917.50	-12.06
-25148.80	-5.91	-25105.39	-5.67	-25064.07	-7.53	-25023.61	-10.24	-24978.79	-8.60	-24938.60	-11.57	-24914.87	-9.43
-25147.31	-4.42	-25106.01	-6.30	-25063.34	-6.80	-25018.06	-4.69	-24979.91	-9.71	-24936.40	-9.38	-24916.38	-10.95
-25148.29	-5.40	-25104.95	-5.24	-25062.56	-6.02	-25024.06	-10.69	-24980.00	-9.80	-24937.88	-10.86	-24915.09	-9.65
-25149.52	-6.63	-25105.27	-5.55	-25061.58	-5.04	-25019.08	-5.71	-24977.20	-7.00	-24938.08	-11.06	-24920.69	-15.25
-25148.33	-5.44	-25104.02	-4.30	-25065.21	-8.67	-25021.90	-8.53	-24978.56	-8.37	-24935.68	-8.66	-24917.04	-11.61
-25147.76	-4.87	-25105.21	-5.50	-25062.64	-6.09	-25018.40	-5.03	-24978.77	-8.57	-24940.34	-13.31	-24915.64	-10.20
-25148.96	-6.07	-25105.01	-5.29	-25058.63	-2.08	-25018.02	-4.65	-24979.21	-9.01	-24938.92	-11.90	-24915.08	-9.64
-25149.26	-6.37	-25104.79	-5.07	-25064.44	-7.90	-25020.09	-6.72	-24980.38	-10.19	-24938.25	-11.23	-24913.12	-7.68
-25148.66	-5.77	-25104.37	-4.66	-25063.11	-6.57	-25022.21	-8.84	-24976.56	-6.37	-24939.27	-12.25	-24916.76	-11.32
-25148.64	-5.75	-25104.58	-4.86	-25062.10	-5.55	-25022.97	-9.60	-24982.53	-12.33	-24933.39	-6.36	-24917.18	-11.74
-25148.87	-5.98	-25104.11	-4.40	-25060.15	-3.61	-25019.87	-6.50	-24974.11	-3.92	-24937.16	-10.14	-24915.27	-9.83
-25148.88	-6.00	-25105.27	-5.56	-25063.81	-7.27	-25020.63	-7.26	-24978.25	-8.05	-24935.63	-8.60	-24915.86	-10.42
-25149.77	-6.88	-25104.69	-4.98	-25062.14	-5.59	-25022.17	-8.80	-24978.95	-8.75	-24935.49	-8.47	-24908.73	-3.30
-25148.71	-5.83	-25106.16	-6.44	-25062.21	-5.67	-25021.01	-7.64	-24978.67	-8.47	-24936.25	-9.23	-24916.59	-11.15
-25148.60	-5.71	-25107.04	-7.32	-25063.04	-6.49	-25017.45	-4.08	-24981.26	-11.06	-24938.64	-11.62	-24920.89	-15.46
-25148.86	-5.97	-25105.28	-5.56	-25062.48	-5.94	-25021.30	-7.93	-24978.89	-8.69	-24938.57	-11.55	-24915.30	-9.86
-25148.66	-5.77	-25105.43	-5.71	-25060.53	-3.98	-25020.93	-7.56	-24980.35	-10.15	-24937.55	-10.53	-24917.21	-11.78

-25147.79	-4.90	-25105.84	-6.13	-25062.68	-6.14	-25018.96	-5.59	-24980.90	-10.70	-24935.17	-8.15	-24911.64	-6.20
-25147.61	-4.72	-25104.72	-5.01	-25062.62	-6.07	-25022.22	-8.85	-24981.42	-11.22	-24933.06	-6.04	-24917.19	-11.75
-25148.39	-5.50	-25105.01	-5.29	-25064.45	-7.90	-25015.52	-2.15	-24976.57	-6.38	-24934.23	-7.21	-24912.76	-7.33
-25148.64	-5.75	-25103.89	-4.18	-25060.59	-4.05	-25021.34	-7.97	-24977.62	-7.43	-24939.37	-12.35	-24917.08	-11.65
-25148.45	-5.56	-25105.40	-5.68	-25064.45	-7.91	-25021.94	-8.58	-24979.86	-9.67	-24937.07	-10.05	-24911.75	-6.31
-25148.47	-5.58	-25104.23	-4.51	-25063.83	-7.29	-25020.38	-7.01	-24979.46	-9.27	-24930.36	-3.33	-24917.65	-12.21
-25148.30	-5.41	-25107.21	-7.50	-25061.14	-4.59	-25020.05	-6.68	-24978.21	-8.02	-24938.55	-11.53	-24915.53	-10.09
-25148.71	-5.82	-25104.84	-5.12	-25064.69	-8.15	-25019.86	-6.49	-24972.85	-2.66	-24938.41	-11.38	-24917.01	-11.58
-25149.83	-6.94	-25103.84	-4.12	-25063.55	-7.00	-25019.40	-6.03	-24979.99	-9.79	-24935.82	-8.80	-24918.67	-13.23
-25149.35	-6.47	-25106.64	-6.93	-25063.60	-7.06	-25022.94	-9.57	-24979.33	-9.13	-24937.99	-10.97	-24914.98	-9.54
-25148.13	-5.24	-25104.40	-4.69	-25059.43	-2.89	-25020.26	-6.89	-24980.40	-10.21	-24934.39	-7.36	-24917.79	-12.36
-25148.56	-5.67	-25106.14	-6.42	-25062.64	-6.10	-25017.27	-3.90	-24977.44	-7.24	-24936.72	-9.69	-24917.72	-12.28
-25146.51	-3.62	-25105.98	-6.26	-25063.29	-6.74	-25020.07	-6.71	-24978.07	-7.87	-24941.87	-14.85	-24916.58	-11.14
-25148.39	-5.50	-25106.36	-6.65	-25060.05	-3.51	-25019.45	-6.08	-24979.40	-9.20	-24937.76	-10.73	-24918.50	-13.06
-25146.82	-3.93	-25106.97	-7.25	-25063.07	-6.53	-25022.22	-8.85	-24979.05	-8.85	-24937.48	-10.45	-24914.23	-8.79
-25148.96	-6.07	-25105.03	-5.31	-25063.09	-6.55	-25018.96	-5.60	-24974.78	-4.59	-24933.90	-6.87	-24909.78	-4.35
-25147.19	-4.30	-25105.60	-5.89	-25062.95	-6.41	-25020.55	-7.18	-24975.70	-5.51	-24937.57	-10.54	-24916.52	-11.08
-25148.97	-6.08	-25105.78	-6.06	-25063.68	-7.14	-25021.11	-7.74	-24981.04	-10.85	-24938.99	-11.97	-24914.58	-9.15
-25149.08	-6.19	-25106.47	-6.76	-25064.43	-7.89	-25019.09	-5.72	-24977.66	-7.46	-24937.82	-10.80	-24914.91	-9.48
-25147.44	-4.55	-25106.66	-6.94	-25062.51	-5.97	-25018.33	-4.96	-24977.06	-6.87	-24936.17	-9.14	-24917.85	-12.41
-25147.12	-4.23	-25105.80	-6.09	-25064.06	-7.52	-25013.42	-0.05	-24980.32	-10.12	-24930.91	-3.89	-24914.53	-9.09
-25148.68	-5.79	-25102.70	-2.98	-25064.15	-7.60	-25020.73	-7.36	-24979.34	-9.14	-24935.34	-8.31	-24915.67	-10.24
-25149.64	-6.75	-25104.07	-4.35	-25063.78	-7.23	-25021.49	-8.12	-24978.43	-8.23	-24937.85	-10.83	-24918.25	-12.81
-25149.24	-6.36	-25104.46	-4.75	-25062.74	-6.19	-25022.61	-9.25	-24976.18	-5.99	-24933.87	-6.85	-24914.94	-9.50
-25148.15	-5.26	-25104.94	-5.23	-25061.97	-5.43	-25022.50	-9.13	-24978.99	-8.79	-24936.26	-9.23	-24910.62	-5.18
-25147.63	-4.75	-25104.21	-4.50	-25063.79	-7.25	-25019.01	-5.64	-24979.11	-8.91	-24933.67	-6.65	-24915.40	-9.97
-25149.08	-6.19	-25106.51	-6.79	-25063.62	-7.08	-25023.08	-9.72	-24981.71	-11.52	-24937.98	-10.96	-24912.61	-7.17
-25147.48	-4.59	-25105.91	-6.19	-25062.76	-6.22	-25021.92	-8.55	-24976.58	-6.39	-24939.87	-12.85	-24915.49	-10.06
-25147.77	-4.88	-25105.37	-5.66	-25064.63	-8.09	-25019.65	-6.28	-24981.74	-11.54	-24938.57	-11.54	-24917.91	-12.47
-25147.98	-5.09	-25106.32	-6.60	-25063.05	-6.51	-25020.13	-6.76	-24979.52	-9.33	-24935.75	-8.72	-24918.03	-12.59
-25146.52	-3.63	-25104.26	-4.55	-25059.36	-2.82	-25021.13	-7.76	-24979.75	-9.56	-24940.16	-13.13	-24914.91	-9.48
-25149.12	-6.23	-25105.46	-5.74	-25064.16	-7.61	-25020.57	-7.20	-24980.05	-9.86	-24936.62	-9.60	-24914.31	-8.88
-25148.79	-5.90	-25105.68	-5.96	-25063.97	-7.43	-25023.88	-10.51	-24979.02	-8.82	-24939.88	-12.85	-24914.82	-9.39
-25149.22	-6.33	-25106.53	-6.82	-25058.69	-2.15	-25021.77	-8.40	-24980.27	-10.07	-24931.61	-4.59	-24917.94	-12.50
-25147.75	-4.86	-25104.64	-4.93	-25063.10	-6.56	-25022.87	-9.51	-24978.50	-8.30	-24940.22	-13.20	-24914.64	-9.20
-25149.73	-6.84	-25105.41	-5.70	-25063.95	-7.41	-25019.08	-5.71	-24980.67	-10.47	-24939.11	-12.09	-24916.16	-10.73
-25148.17	-5.28	-25106.41	-6.70	-25065.41	-8.87	-25018.29	-4.92	-24978.28	-8.08	-24938.14	-11.12	-24913.32	-7.89
-25147.34	-4.45	-25105.56	-5.84	-25064.31	-7.77	-25020.47	-7.10	-24979.91	-9.71	-24939.50	-12.47	-24914.78	-9.34
-25149.71	-6.82	-25103.71	-4.00	-25062.16	-5.62	-25019.64	-6.28	-24979.34	-9.14	-24937.72	-10.70	-24909.78	-4.34
-25149.02	-6.13	-25104.99	-5.28	-25064.16	-7.62	-25020.81	-7.44	-24980.97	-10.78	-24938.28	-11.25	-24917.86	-12.43
-25148.97	-6.08	-25105.30	-5.58	-25063.98	-7.44	-25017.63	-4.26	-24978.36	-8.16	-24939.52	-12.50	-24919.28	-13.85

	-25148.30	-5.41	-25104.45	-4.73	-25062.68	-6.14	-25021.00	-7.63	-24979.46	-9.26	-24937.13	-10.11	-24915.87	-10.44
	-25148.11	-5.22	-25104.87	-5.15	-25064.28	-7.73	-25020.83	-7.46	-24977.72	-7.53	-24937.81	-10.79	-24914.50	-9.07
	-25148.75	-5.86	-25106.90	-7.19	-25065.85	-9.31	-25019.42	-6.05	-24980.01	-9.81	-24938.17	-11.15	-24915.75	-10.32
	-25147.70	-4.82	-25104.79	-5.08	-25063.16	-6.62	-25022.20	-8.83	-24978.97	-8.78	-24938.70	-11.68	-24917.37	-11.93
	-25148.76	-5.87	-25104.34	-4.63	-25064.61	-8.07	-25020.09	-6.72	-24979.92	-9.72	-24936.43	-9.41	-24915.09	-9.65
	-25149.72	-6.83	-25104.96	-5.25	-25061.02	-4.48	-25022.73	-9.36	-24977.70	-7.51	-24936.18	-9.16	-24914.80	-9.36
	-25148.94	-6.05	-25105.82	-6.11	-25058.31	-1.77	-25020.77	-7.40	-24977.10	-6.90	-24928.40	-1.38	-24916.36	-10.92
	-25148.92	-6.03	-25106.43	-6.71			-25021.78	-8.42	-24979.80	-9.60			-24916.88	-11.44
	-25148.13	-5.25							-24979.00	-8.80				
Average energies	-25148.42	-5.53	-25105.40	-5.69	-25062.99	-6.45	-25020.57	-7.21	-24978.85	-8.65	-24937.36	-10.33	-24915.83	-10.40

Pu

	ground state energy	solid solution energy												
concentration in solid solution (%)	3		6		9		12		15		18		21	
number of atoms	2		4		6		8		10		12		13	
	-25143.40	-6.50	-25093.87	-6.13	-25042.39	-3.80	-24996.87	-7.45	-24951.14	-10.87	-24903.88	-12.77	-24873.50	-6.97
	-25142.79	-5.88	-25094.24	-6.50	-25047.30	-8.72	-24997.83	-8.40	-24949.79	-9.52	-24902.21	-11.10	-24879.76	-13.23
	-25142.99	-6.08	-25093.28	-5.53	-25045.19	-6.60	-24997.40	-7.97	-24947.05	-6.79	-24900.33	-9.22	-24877.31	-10.78
	-25143.30	-6.39	-25092.68	-4.93	-25047.13	-8.54	-24999.29	-9.86	-24951.01	-10.75	-24898.01	-6.90	-24880.06	-13.53
	-25141.39	-4.48	-25094.03	-6.28	-25044.06	-5.48	-24997.28	-7.85	-24947.12	-6.85	-24903.70	-12.59	-24878.83	-12.30
	-25143.12	-6.21	-25095.14	-7.40	-25043.85	-5.26	-24995.55	-6.12	-24952.52	-12.25	-24903.35	-12.24	-24878.96	-12.43
	-25141.83	-4.92	-25093.80	-6.06	-25041.03	-2.44	-24996.88	-7.46	-24948.52	-8.25	-24902.78	-11.67	-24878.63	-12.10
	-25141.97	-5.07	-25093.81	-6.07	-25045.88	-7.30	-24997.43	-8.01	-24949.29	-9.02	-24901.80	-10.69	-24880.02	-13.49
	-25142.90	-6.00	-25094.84	-7.10	-25044.57	-5.98	-24997.61	-8.18	-24949.81	-9.54	-24901.78	-10.67	-24880.55	-14.02
	-25142.36	-5.45	-25094.33	-6.58	-25042.57	-3.99	-24997.26	-7.84	-24949.86	-9.59	-24904.23	-13.12	-24877.60	-11.07
	-25142.37	-5.46	-25092.07	-4.33	-25045.32	-6.73	-24998.36	-8.94	-24950.46	-10.19	-24903.01	-11.91	-24878.83	-12.30
	-25142.38	-5.48	-25093.69	-5.95	-25044.93	-6.34	-24999.38	-9.95	-24950.72	-10.45	-24900.71	-9.60	-24879.60	-13.07
	-25142.79	-5.89	-25092.78	-5.03	-25048.07	-9.48	-25000.52	-11.09	-24949.42	-9.15	-24904.08	-12.97	-24881.06	-14.53
	-25141.96	-5.05	-25094.40	-6.66	-25045.53	-6.94	-24994.28	-4.86	-24952.31	-12.05	-24900.80	-9.69	-24879.78	-13.25
	-25142.89	-5.98	-25094.69	-6.95	-25047.34	-8.75	-24996.69	-7.26	-24950.09	-9.82	-24903.03	-11.92	-24878.80	-12.27
	-25143.00	-6.09	-25093.13	-5.38	-25042.55	-3.97	-24994.85	-5.42	-24948.56	-8.29	-24900.66	-9.55	-24878.77	-12.24
	-25142.25	-5.35	-25095.54	-7.79	-25046.55	-7.97	-24999.70	-10.28	-24950.61	-10.34	-24901.23	-10.12	-24877.43	-10.90
	-25142.59	-5.69	-25093.37	-5.63	-25044.66	-6.08	-24995.99	-6.56	-24947.43	-7.16	-24902.23	-11.12	-24877.91	-11.38
	-25143.16	-6.26	-25092.80	-5.06	-25044.28	-5.69	-24999.58	-10.16	-24950.60	-10.33	-24899.63	-8.52	-24879.53	-13.00
	-25144.91	-8.01	-25092.62	-4.88	-25047.43	-8.85	-24997.84	-8.41	-24946.71	-6.44	-24905.09	-13.98	-24878.52	-11.99
	-25143.20	-6.29	-25093.83	-6.09	-25042.17	-3.59	-24997.40	-7.98	-24950.31	-10.04	-24904.82	-13.71	-24880.34	-13.81
	-25142.24	-5.34	-25094.67	-6.93	-25045.83	-7.24	-24997.90	-8.48	-24952.22	-11.95	-24902.98	-11.87	-24879.16	-12.63
	-25142.25	-5.34	-25092.17	-4.43	-25046.07	-7.48	-24998.88	-9.45	-24951.61	-11.34	-24903.30	-12.19	-24878.51	-11.98

-25142.62	-5.72	-25094.08	-6.33	-25043.47	-4.88	-24998.61	-9.18	-24950.75	-10.48	-24900.39	-9.28	-24878.87	-12.34
-25142.40	-5.49	-25091.38	-3.63	-25042.93	-4.34	-24998.69	-9.26	-24949.76	-9.49	-24904.05	-12.94	-24874.69	-8.16
-25141.32	-4.42	-25093.57	-5.82	-25041.54	-2.96	-24995.03	-5.61	-24949.93	-9.66	-24898.78	-7.67	-24877.86	-11.33
-25142.80	-5.89	-25093.33	-5.59	-25045.84	-7.25	-24999.50	-10.08	-24949.82	-9.55	-24903.13	-12.02	-24880.30	-13.77
-25142.17	-5.27	-25092.79	-5.04	-25046.44	-7.86	-24996.43	-7.01	-24952.24	-11.97	-24903.73	-12.62	-24879.93	-13.40
-25141.10	-4.20	-25093.36	-5.61	-25041.81	-3.22	-24999.41	-9.99	-24947.29	-7.02	-24900.92	-9.81	-24878.70	-12.17
-25142.85	-5.95	-25093.91	-6.17	-25044.80	-6.22	-24994.46	-5.04	-24948.55	-8.28	-24904.04	-12.93	-24880.86	-14.33
-25142.80	-5.90	-25094.20	-6.46	-25044.49	-5.90	-24997.76	-8.34	-24951.65	-11.39	-24904.99	-13.88	-24877.56	-11.03
-25143.53	-6.63	-25092.01	-4.27	-25043.52	-4.93	-24998.15	-8.73	-24949.18	-8.91	-24898.09	-6.98	-24878.21	-11.68
-25143.11	-6.20	-25094.20	-6.46	-25046.67	-8.09	-24999.17	-9.75	-24948.89	-8.62	-24902.35	-11.24	-24881.11	-14.58
-25141.41	-4.50	-25094.31	-6.57	-25043.44	-4.85	-24998.37	-8.95	-24949.91	-9.64	-24903.51	-12.40	-24878.00	-11.47
-25142.92	-6.02	-25094.88	-7.14	-25048.16	-9.57	-24996.95	-7.52	-24950.02	-9.75	-24905.10	-13.99	-24876.96	-10.43
-25142.07	-5.17	-25094.23	-6.48	-25046.11	-7.52	-25000.08	-10.65	-24952.13	-11.86	-24903.69	-12.58	-24876.59	-10.06
-25143.50	-6.59	-25095.10	-7.36	-25046.53	-7.94	-24997.97	-8.54	-24948.78	-8.51	-24899.35	-8.24	-24879.53	-13.00
-25142.29	-5.39	-25094.19	-6.45	-25045.20	-6.61	-24998.79	-9.37	-24950.14	-9.87	-24903.83	-12.72	-24877.56	-11.03
-25143.99	-7.09	-25093.21	-5.46	-25044.99	-6.40	-24998.82	-9.39	-24949.16	-8.89	-24900.89	-9.78	-24880.15	-13.62
-25142.56	-5.65	-25093.58	-5.83	-25045.30	-6.71	-24996.03	-6.61	-24948.90	-8.63	-24901.88	-10.77	-24877.07	-10.54
-25141.79	-4.88	-25094.69	-6.95	-25046.46	-7.87	-24994.44	-5.01	-24949.73	-9.46	-24900.42	-9.31	-24882.19	-15.66
-25142.65	-5.75	-25093.25	-5.50	-25045.86	-7.27	-24995.29	-5.86	-24949.23	-8.96	-24899.50	-8.39	-24879.43	-12.90
-25142.98	-6.08	-25093.04	-5.29	-25044.97	-6.38	-24996.48	-7.05	-24950.12	-9.85	-24905.68	-14.58	-24876.83	-10.30
-25142.03	-5.12	-25093.52	-5.78	-25045.96	-7.37	-24996.05	-6.63	-24948.84	-8.57	-24903.17	-12.06	-24879.28	-12.75
-25144.16	-7.25	-25093.86	-6.11	-25043.95	-5.37	-24997.33	-7.90	-24951.42	-11.15	-24902.78	-11.67	-24876.18	-9.65
-25142.99	-6.08	-25093.58	-5.84	-25046.06	-7.47	-24996.86	-7.43	-24951.87	-11.61	-24902.15	-11.04	-24880.05	-13.52
-25141.64	-4.74	-25095.06	-7.32	-25045.12	-6.54	-24997.50	-8.08	-24947.51	-7.24	-24904.43	-13.32	-24878.04	-11.51
-25141.59	-4.68	-25095.39	-7.65	-25044.41	-5.82	-24999.45	-10.02	-24951.56	-11.29	-24901.73	-10.62	-24879.13	-12.60
-25141.50	-4.60	-25093.45	-5.71	-25046.16	-7.57	-24997.49	-8.07	-24951.18	-10.91	-24903.46	-12.35	-24873.76	-7.23
-25142.01	-5.11	-25095.49	-7.75	-25046.94	-8.35	-24997.17	-7.74	-24949.42	-9.15	-24904.59	-13.48	-24880.45	-13.92
-25141.96	-5.06	-25093.98	-6.24	-25044.86	-6.28	-24998.53	-9.10	-24948.56	-8.30	-24898.44	-7.33	-24880.05	-13.52
-25142.52	-5.62	-25094.24	-6.50	-25046.38	-7.79	-24996.77	-7.34	-24951.15	-10.88	-24904.35	-13.24	-24879.56	-13.03
-25142.91	-6.00	-25092.35	-4.61	-25045.12	-6.53	-24998.45	-9.02	-24951.83	-11.57	-24903.44	-12.33	-24879.54	-13.01
-25140.45	-3.55	-25094.26	-6.52	-25046.16	-7.58	-24997.86	-8.44	-24950.83	-10.56	-24901.84	-10.73	-24878.36	-11.83
-25142.84	-5.94	-25093.81	-6.07	-25045.72	-7.14	-24998.33	-8.90	-24950.73	-10.47	-24904.06	-12.95	-24879.07	-12.54
-25142.66	-5.76	-25094.15	-6.40	-25046.90	-8.32	-24998.21	-8.78	-24950.15	-9.89	-24901.29	-10.18	-24880.70	-14.16
-25143.07	-6.16	-25092.89	-5.14	-25046.67	-8.09	-24992.23	-2.81	-24949.23	-8.96	-24904.82	-13.71	-24877.54	-11.01
-25142.63	-5.72	-25093.34	-5.60	-25045.90	-7.31	-24997.62	-8.19	-24950.01	-9.74	-24901.24	-10.13	-24878.18	-11.65
-25142.30	-5.39	-25093.79	-6.04	-25046.32	-7.74	-24997.48	-8.05	-24950.20	-9.93	-24900.48	-9.37	-24877.39	-10.86
-25143.22	-6.31	-25094.51	-6.76	-25046.93	-8.35	-24997.78	-8.36	-24949.70	-9.43	-24901.91	-10.80	-24879.66	-13.12
-25142.03	-5.12	-25093.96	-6.21	-25046.79	-8.21	-24997.20	-7.78	-24950.62	-10.35	-24899.46	-8.35	-24878.54	-12.01
-25141.90	-5.00	-25092.42	-4.67	-25047.24	-8.66	-24995.44	-6.02	-24950.82	-10.55	-24901.61	-10.50	-24878.35	-11.82
-25143.01	-6.11	-25092.23	-4.48	-25046.96	-8.37	-24996.04	-6.61	-24952.41	-12.14	-24900.14	-9.03	-24881.22	-14.69
-25142.66	-5.76	-25094.44	-6.69	-25045.59	-7.01	-24999.70	-10.28	-24951.76	-11.49	-24903.25	-12.14	-24880.90	-14.36

-25143.12	-6.21	-25093.36	-5.61	-25046.30	-7.72	-24998.54	-9.12	-24948.43	-8.16	-24904.51	-13.40	-24880.46	-13.93
-25141.49	-4.58	-25094.74	-7.00	-25043.79	-5.20	-24997.13	-7.70	-24948.57	-8.30	-24902.02	-10.91	-24875.62	-9.08
-25142.61	-5.71	-25094.88	-7.13	-25044.95	-6.37	-24997.54	-8.11	-24949.65	-9.39	-24902.90	-11.79	-24880.88	-14.35
-25142.73	-5.82	-25094.48	-6.74	-25043.76	-5.17	-24997.69	-8.26	-24950.95	-10.68	-24901.92	-10.81	-24880.69	-14.16
-25141.47	-4.57	-25092.70	-4.96	-25045.43	-6.84	-24997.31	-7.89	-24944.90	-4.63	-24901.87	-10.76	-24879.94	-13.41
-25143.69	-6.79	-25093.84	-6.10	-25046.42	-7.83	-24995.52	-6.10	-24947.82	-7.55	-24900.79	-9.68	-24881.19	-14.66
-25141.34	-4.44	-25092.81	-5.06	-24998.59	39.99	-24998.03	-8.60	-24904.83	35.44	-24877.98	13.13	-24882.15	-15.61
-25094.61	42.29	-25046.65	41.09	-25046.16	-7.57	-24950.77	38.66	-24951.03	-10.76	-24901.40	-10.29	-24878.12	-11.59
-25143.28	-6.38	-25095.04	-7.30	-25046.22	-7.63	-24998.77	-9.34	-24950.53	-10.26	-24903.68	-12.57	-24878.80	-12.27
-25142.21	-5.31	-25095.40	-7.65	-25045.78	-7.19	-24997.69	-8.26	-24952.32	-12.05	-24903.04	-11.94	-24880.55	-14.02
-25143.06	-6.16	-25093.39	-5.64	-25047.52	-8.93	-24995.76	-6.33	-24949.76	-9.49	-24901.48	-10.37	-24878.15	-11.62
-25139.95	-3.05	-25092.28	-4.53	-25045.75	-7.17	-24998.08	-8.66	-24950.14	-9.87	-24901.31	-10.20	-24878.32	-11.79
-25141.78	-4.88	-25094.46	-6.72	-25044.79	-6.20	-24996.12	-6.69	-24948.48	-8.21	-24903.84	-12.73	-24877.62	-11.09
-25143.17	-6.27	-25091.40	-3.66	-25044.60	-6.01	-24997.30	-7.88	-24950.39	-10.12	-24903.00	-11.89	-24880.86	-14.33
-25142.64	-5.73	-25093.18	-5.44	-25046.08	-7.50	-24997.38	-7.96	-24950.35	-10.08	-24904.66	-13.55	-24879.93	-13.40
-25141.39	-4.49	-25094.55	-6.80	-25046.78	-8.20	-24996.81	-7.38	-24950.06	-9.79	-24900.45	-9.34	-24879.94	-13.41
-25141.87	-4.96	-25093.90	-6.15	-25047.17	-8.59	-25000.63	-11.20	-24950.21	-9.94	-24902.07	-10.96	-24879.55	-13.02
-25141.50	-4.60	-25093.94	-6.19	-25045.37	-6.79	-24996.62	-7.20	-24951.52	-11.25	-24899.36	-8.25	-24877.84	-11.31
-25143.01	-6.10	-25094.40	-6.65	-25042.50	-3.91	-24997.72	-8.30	-24950.94	-10.67	-24903.74	-12.63	-24877.45	-10.92
-25141.86	-4.95	-25095.48	-7.74	-25046.18	-7.59	-24998.07	-8.64	-24952.06	-11.79	-24902.86	-11.75	-24879.35	-12.82
-25144.00	-7.10	-25092.39	-4.65	-25045.50	-6.92	-24997.31	-7.88	-24949.68	-9.41	-24902.92	-11.81	-24877.68	-11.15
-25142.36	-5.45	-25093.12	-5.38	-25047.09	-8.51	-24997.38	-7.96	-24950.81	-10.54	-24903.37	-12.26	-24881.20	-14.67
-25142.19	-5.28	-25093.46	-5.71	-25045.06	-6.48	-24994.74	-5.31	-24951.18	-10.91	-24901.02	-9.91	-24878.59	-12.06
-25141.92	-5.01	-25092.79	-5.05	-25044.60	-6.02	-24998.61	-9.18	-24951.30	-11.03	-24902.25	-11.14	-24880.63	-14.10
-25142.49	-5.59	-25095.08	-7.34	-25047.96	-9.37	-24996.95	-7.52	-24950.19	-9.92	-24903.86	-12.75	-24876.09	-9.56
-25142.89	-5.99	-25093.90	-6.15	-25044.97	-6.39	-24997.39	-7.96	-24948.46	-8.19	-24901.03	-9.92	-24875.93	-9.40
-25142.88	-5.98	-25092.77	-5.03	-25046.88	-8.29	-24997.27	-7.84	-24949.76	-9.49	-24901.84	-10.73	-24880.21	-13.68
-25141.78	-4.88	-25093.34	-5.59	-25046.42	-7.83	-24997.93	-8.51	-24950.34	-10.07	-24901.51	-10.40	-24877.51	-10.98
-25143.64	-6.73	-25093.92	-6.17	-25046.26	-7.68	-24998.39	-8.96	-24949.66	-9.39	-24900.75	-9.64	-24876.56	-10.03
-25142.49	-5.58	-25092.61	-4.86	-25044.58	-5.99	-24998.46	-9.03	-24948.35	-8.08	-24900.61	-9.51	-24881.02	-14.49
-25141.80	-4.90	-25093.31	-5.57	-25046.22	-7.63	-24999.92	-10.50	-24947.10	-6.83	-24902.01	-10.90	-24879.71	-13.18
-25142.17	-5.26	-25094.80	-7.06	-25046.38	-7.79	-24996.88	-7.45	-24949.66	-9.39	-24903.72	-12.61	-24875.66	-9.13
-25140.32	-3.41	-25093.58	-5.84	-25044.04	-5.45	-24993.83	-4.41	-24939.13	1.14	-24903.72	-12.61	-24878.00	-11.47
-25141.51	-4.60	-25092.11	-4.36	-25046.06	-7.47	-24998.41	-8.98	-24950.80	-10.53	-24903.19	-12.08	-24876.93	-10.40
-25142.93	-6.03	-25094.36	-6.62	-25045.46	-6.87	-24997.90	-8.48	-24950.60	-10.33	-24902.08	-10.97	-24878.81	-12.28
-25142.77	-5.87	-25093.92	-6.17	-25046.11	-7.52	-24998.64	-9.22	-24952.41	-12.14	-24902.77	-11.66	-24880.21	-13.68
-25142.13	-5.22	-25094.39	-6.65	-25047.29	-8.70	-24995.33	-5.90	-24948.50	-8.24	-24905.07	-13.96	-24876.10	-9.57
-25142.48	-5.58	-25093.03	-5.28	-25043.22	-4.63	-24996.08	-6.65	-24946.59	-6.32	-24902.33	-11.22	-24879.43	-12.90
-25143.23	-6.33	-25091.76	-4.01	-25044.92	-6.34	-24998.83	-9.40	-24947.22	-6.95	-24902.77	-11.66	-24880.34	-13.81
-25142.98	-6.08	-25093.20	-5.46	-25046.29	-7.71	-24997.11	-7.68	-24950.54	-10.27	-24902.02	-10.91	-24879.11	-12.58
-25140.88	-3.98	-25092.97	-5.22	-25046.75	-8.17	-24996.45	-7.02	-24950.51	-10.24	-24900.93	-9.82	-24878.97	-12.44

	-25143.28	-6.38	-25092.39	-4.64	-25046.09	-7.50	-24997.70	-8.27	-24949.59	-9.32	-24901.01	-9.90	-24878.20	-11.67
	-25142.36	-5.46	-25092.09	-4.35	-25045.93	-7.34	-25000.03	-10.60	-24948.17	-7.91	-24904.14	-13.03	-24879.27	-12.74
	-25141.43	-4.52	-25094.75	-7.00	-25045.07	-6.48	-24999.37	-9.95	-24948.73	-8.47	-24901.90	-10.79	-24881.17	-14.64
	-25143.17	-6.27	-25093.44	-5.69	-25044.45	-5.86	-24999.43	-10.00	-24946.18	-5.91	-24902.11	-11.00	-24878.03	-11.50
	-25143.15	-6.24	-25095.46	-7.71	-25046.47	-7.88	-25000.98	-11.56	-24949.39	-9.13	-24904.99	-13.88	-24882.11	-15.58
	-25142.06	-5.16	-25094.83	-7.09	-25047.24	-8.66	-24997.39	-7.96	-24948.50	-8.23	-24903.26	-12.15	-24879.05	-12.52
	-25142.68	-5.78	-25092.63	-4.89	-25047.24	-8.65	-24996.05	-6.62	-24949.88	-9.61	-24905.47	-14.36	-24879.61	-13.08
	-25141.30	-4.39	-25093.79	-6.05	-25047.32	-8.73	-24999.51	-10.08	-24948.71	-8.44	-24903.31	-12.20	-24879.60	-13.07
	-25141.79	-4.89	-25094.39	-6.65	-25045.12	-6.53	-24995.92	-6.50	-24951.33	-11.07	-24904.12	-13.01	-24876.79	-10.26
	-25142.46	-5.56	-25093.00	-5.25	-25046.36	-7.77	-24998.63	-9.20	-24947.26	-6.99	-24903.37	-12.26	-24881.08	-14.55
	-25143.17	-6.27	-25092.44	-4.70	-25044.82	-6.23	-24998.19	-8.76	-24950.03	-9.76	-24902.80	-11.69	-24878.58	-12.05
	-25141.70	-4.80	-25094.27	-6.53	-25045.51	-6.93	-24999.80	-10.37	-24949.82	-9.55	-24901.36	-10.25	-24880.15	-13.62
	-25141.86	-4.95	-25091.64	-3.89	-25045.38	-6.79	-24993.62	-4.19	-24951.17	-10.90	-24900.44	-9.33	-24880.45	-13.92
	-25141.11	-4.21	-25092.38	-4.63			-24996.09	-6.66			-24903.17	-12.06	-24877.67	-11.14
	-25142.77	-5.87	-25094.09	-6.35			-24997.85	-8.43						
Average energies	-25142	-5.12868	-25093.3	-5.53109	-25045.1	-6.52057	-24997.1	-7.71147	-24949.4	-9.11791	-24902.2	-11.0813	-24878.9	-12.3743

Th

	ground state energy	solid solution energy												
concentration in solid solution (%)	3		6		9		12		15		18		21	
number of atoms	2		4		6		8		10		12		13	
	-25147.61	-5.08	-25105.76	-6.76	-25062.48	-7.00	-25018.98	-7.03	-24977.11	-8.69	-24935.91	-11.01	-24913.56	-10.43
	-25146.54	-4.01	-25106.28	-7.27	-25061.96	-6.48	-25013.02	-1.07	-24975.06	-6.64	-24934.90	-10.00	-24912.88	-9.75
	-25146.80	-4.27	-25104.74	-5.73	-25063.33	-7.85	-25019.34	-7.39	-24978.51	-10.09	-24934.08	-9.18	-24913.99	-10.86
	-25148.77	-6.24	-25107.41	-8.40	-25060.73	-5.25	-25019.40	-7.45	-24976.64	-8.21	-24933.67	-8.77	-24917.56	-14.44
	-25148.70	-6.16	-25105.48	-6.47	-25061.50	-6.03	-25019.00	-7.05	-24976.29	-7.87	-24931.87	-6.97	-24914.75	-11.62
	-25148.91	-6.38	-25102.00	-3.00	-25062.75	-7.27	-25017.23	-5.29	-24979.06	-10.64	-24931.76	-6.87	-24911.53	-8.40
	-25147.06	-4.53	-25104.32	-5.32	-25063.04	-7.57	-25019.94	-7.99	-24978.09	-9.67	-24936.82	-11.93	-24916.33	-13.20
	-25148.12	-5.59	-25105.14	-6.14	-25059.85	-4.37	-25021.11	-9.16	-24979.79	-11.37	-24935.44	-10.55	-24914.23	-11.10
	-25149.08	-6.54	-25104.47	-5.46	-25063.42	-7.95	-25020.94	-8.99	-24975.42	-7.00	-24933.22	-8.33	-24912.83	-9.70
	-25147.75	-5.22	-25103.40	-4.39	-25062.96	-7.48	-25020.78	-8.83	-24975.46	-7.04	-24935.11	-10.22	-24913.11	-9.98
	-25148.10	-5.56	-25105.11	-6.11	-25063.29	-7.82	-25021.10	-9.15	-24977.29	-8.87	-24938.30	-13.41	-24914.49	-11.37
	-25147.86	-5.33	-25105.29	-6.29	-25062.32	-6.84	-25019.87	-7.92	-24979.41	-10.99	-24936.75	-11.86	-24913.30	-10.17
	-25148.04	-5.51	-25103.90	-4.90	-25060.18	-4.70	-25020.36	-8.41	-24975.49	-7.07	-24934.72	-9.83	-24914.22	-11.09
	-25147.67	-5.14	-25105.80	-6.80	-25062.39	-6.91	-25019.81	-7.86	-24976.95	-8.53	-24929.58	-4.69	-24915.55	-12.42
	-25146.50	-3.97	-25103.68	-4.67	-25059.68	-4.20	-25017.86	-5.91	-24977.27	-8.85	-24936.55	-11.66	-24910.17	-7.04
	-25146.47	-3.94	-25104.84	-5.83	-25063.77	-8.29	-25020.96	-9.01	-24978.82	-10.40	-24932.46	-7.57	-24916.61	-13.48
	-25147.03	-4.50	-25105.25	-6.25	-25058.44	-2.96	-25018.62	-6.67	-24978.34	-9.92	-24922.04	2.86	-24917.52	-14.39

-25146.59	-4.05	-25105.17	-6.16	-25063.26	-7.78	-25020.62	-8.67	-24975.35	-6.93	-24938.00	-13.10	-24916.34	-13.21
-25148.13	-5.59	-25107.64	-8.64	-25060.25	-4.77	-25019.25	-7.30	-24975.88	-7.46	-24934.65	-9.76	-24916.89	-13.76
-25147.20	-4.67	-25104.78	-5.78	-25060.96	-5.48	-25015.13	-3.18	-24979.47	-11.05	-24937.27	-12.38	-24913.11	-9.98
-25145.39	-2.85	-25103.83	-4.83	-25063.91	-8.43	-25018.62	-6.67	-24976.33	-7.91	-24934.56	-9.67	-24914.94	-11.81
-25148.14	-5.60	-25107.75	-8.75	-25064.26	-8.79	-25018.09	-6.14	-24976.45	-8.03	-24932.29	-7.40	-24912.44	-9.31
-25147.89	-5.36	-25105.94	-6.93	-25063.68	-8.21	-25020.03	-8.08	-24979.07	-10.65	-24938.70	-13.80	-24917.30	-14.17
-25147.47	-4.94	-25104.73	-5.73	-25062.73	-7.25	-25020.00	-8.05	-24978.56	-10.14	-24935.74	-10.85	-24915.98	-12.85
-25149.13	-6.60	-25106.05	-7.05	-25063.74	-8.26	-25017.93	-5.98	-24977.18	-8.76	-24932.87	-7.98	-24913.21	-10.08
-25148.05	-5.51	-25105.33	-6.32	-25064.00	-8.53	-25018.00	-6.05	-24976.80	-8.38	-24936.41	-11.52	-24915.83	-12.70
-25148.65	-6.12	-25104.95	-5.94	-25060.72	-5.25	-25017.50	-5.55	-24979.32	-10.90	-24934.83	-9.94	-24916.92	-13.80
-25148.91	-6.38	-25103.91	-4.90	-25062.41	-6.94	-25018.96	-7.02	-24979.63	-11.21	-24935.72	-10.83	-24913.52	-10.39
-25148.48	-5.95	-25104.49	-5.48	-25061.53	-6.06	-25021.21	-9.26	-24978.56	-10.14	-24936.16	-11.27	-24911.47	-8.34
-25148.36	-5.83	-25105.46	-6.45	-25063.49	-8.01	-25021.70	-9.75	-24974.82	-6.40	-24934.11	-9.22	-24905.70	-2.57
-25148.64	-6.11	-25105.82	-6.82	-25063.60	-8.12	-25020.36	-8.41	-24977.20	-8.78	-24936.95	-12.06	-24913.79	-10.66
-25148.96	-6.43	-25104.21	-5.20	-25063.74	-8.26	-25017.16	-5.21	-24979.23	-10.81	-24938.48	-13.58	-24917.52	-14.40
-25147.11	-4.58	-25104.73	-5.72	-25063.66	-8.18	-25022.23	-10.28	-24979.18	-10.76	-24935.59	-10.69	-24915.80	-12.67
-25147.18	-4.64	-25105.69	-6.69	-25061.98	-6.50	-25021.60	-9.65	-24978.57	-10.15	-24936.60	-11.70	-24914.99	-11.86
-25148.48	-5.94	-25105.21	-6.20	-25060.66	-5.18	-25020.52	-8.57	-24976.15	-7.73	-24936.90	-12.01	-24916.81	-13.69
-25148.17	-5.64	-25105.61	-6.61	-25061.62	-6.14	-25020.84	-8.89	-24978.98	-10.56	-24936.48	-11.59	-24916.45	-13.33
-25147.57	-5.04	-25103.59	-4.59	-25061.34	-5.86	-25020.82	-8.88	-24978.05	-9.63	-24932.17	-7.28	-24913.68	-10.55
-25149.40	-6.86	-25105.80	-6.79	-25065.22	-9.74	-25020.38	-8.43	-24976.07	-7.65	-24934.73	-9.84	-24916.12	-12.99
-25146.07	-3.53	-25104.08	-5.08	-25061.90	-6.43	-25011.97	-0.02	-24969.29	-0.87	-24938.22	-13.33	-24917.87	-14.74
-25148.83	-6.30	-25105.63	-6.62	-25059.08	-3.60	-25017.11	-5.16	-24976.95	-8.53	-24933.79	-8.90	-24916.52	-13.39
-25148.82	-6.28	-25105.68	-6.68	-25063.01	-7.54	-25019.06	-7.11	-24976.11	-7.69	-24937.62	-12.72	-24915.20	-12.07
-25146.98	-4.44	-25104.68	-5.68	-25062.37	-6.90	-25020.96	-9.01	-24971.59	-3.17	-24937.48	-12.58	-24915.12	-11.99
-25148.57	-6.03	-25103.56	-4.55	-25065.74	-10.26	-25017.91	-5.96	-24975.08	-6.66	-24934.64	-9.75	-24912.56	-9.43
-25148.56	-6.03	-25105.54	-6.54	-25060.92	-5.44	-25019.15	-7.20	-24978.40	-9.98	-24933.63	-8.74	-24916.36	-13.23
-25148.02	-5.49	-25103.53	-4.52	-25060.99	-5.51	-25019.33	-7.38	-24973.52	-5.10	-24936.26	-11.37	-24907.99	-4.86
-25147.88	-5.34	-25106.61	-7.61	-25062.67	-7.19	-25017.41	-5.46	-24978.68	-10.26	-24934.50	-9.60	-24916.87	-13.74
-25148.01	-5.48	-25104.67	-5.66	-25062.25	-6.77	-25019.98	-8.03	-24976.16	-7.74	-24935.20	-10.31	-24911.77	-8.64
-25149.22	-6.69	-25104.78	-5.77	-25062.55	-7.07	-25019.07	-7.12	-24978.68	-10.26	-24937.66	-12.77	-24914.20	-11.07
-25148.77	-6.23	-25102.42	-3.42	-25062.35	-6.87	-25017.81	-5.86	-24975.94	-7.52	-24936.09	-11.20	-24916.85	-13.72
-25146.75	-4.21	-25103.94	-4.94	-25060.52	-5.04	-25018.05	-6.10	-24978.51	-10.09	-24934.71	-9.82	-24914.75	-11.62
-25148.83	-6.30	-25104.56	-5.55	-25062.84	-7.37	-25018.38	-6.43	-24977.63	-9.21	-24934.05	-9.16	-24918.04	-14.91
-25148.34	-5.81	-25104.43	-5.42	-25062.05	-6.57	-25019.18	-7.23	-24978.63	-10.21	-24934.18	-9.28	-24917.36	-14.23
-25149.06	-6.53	-25103.66	-4.66	-25062.75	-7.28	-25020.57	-8.62	-24976.18	-7.76	-24938.24	-13.35	-24915.37	-12.24
-25149.36	-6.82	-25104.57	-5.57	-25063.73	-8.25	-25016.06	-4.11	-24979.86	-11.44	-24937.94	-13.05	-24909.16	-6.03
-25147.80	-5.26	-25106.51	-7.50	-25060.20	-4.73	-25014.08	-2.13	-24975.38	-6.96	-24933.91	-9.02	-24918.15	-15.02
-25148.24	-5.71	-25104.54	-5.53	-25062.56	-7.08	-25022.43	-10.48	-24979.15	-10.73	-24931.83	-6.94	-24915.69	-12.56
-25149.74	-7.21	-25104.79	-5.79	-25062.91	-7.43	-25019.11	-7.17	-24973.69	-5.27	-24935.45	-10.55	-24914.65	-11.53
-25148.86	-6.32	-25102.70	-3.69	-25059.95	-4.47	-25021.27	-9.32	-24975.47	-7.05	-24937.91	-13.02	-24913.87	-10.74

-25148.45	-5.92	-25103.64	-4.64	-25061.37	-5.89	-25019.50	-7.55	-24979.12	-10.70	-24935.15	-10.26	-24915.90	-12.77
-25149.14	-6.60	-25107.12	-8.12	-25063.57	-8.09	-25015.51	-3.56	-24977.19	-8.77	-24932.97	-8.08	-24910.44	-7.31
-25147.15	-4.62	-25105.70	-6.70	-25061.32	-5.85	-25020.68	-8.73	-24981.67	-13.25	-24939.18	-14.29	-24912.93	-9.80
-25147.93	-5.39	-25105.29	-6.28	-25061.77	-6.29	-25014.32	-2.37	-24977.03	-8.61	-24936.55	-11.66	-24916.85	-13.72
-25147.88	-5.34	-25104.55	-5.55	-25060.03	-4.56	-25019.81	-7.86	-24976.52	-8.10	-24935.68	-10.79	-24915.31	-12.18
-25148.30	-5.76	-25105.00	-6.00	-25062.42	-6.95	-25019.12	-7.17	-24972.48	-4.06	-24932.35	-7.45	-24916.45	-13.32
-25148.23	-5.70	-25104.21	-5.21	-25060.88	-5.40	-25021.01	-9.06	-24977.96	-9.54	-24934.34	-9.44	-24911.38	-8.26
-25148.70	-6.16	-25106.01	-7.01	-25061.40	-5.92	-25018.72	-6.77	-24977.44	-9.02	-24932.55	-7.65	-24913.09	-9.96
-25148.39	-5.86	-25105.44	-6.44	-25063.26	-7.79	-25013.30	-1.35	-24972.42	-3.99	-24935.41	-10.51	-24916.23	-13.10
-25148.73	-6.19	-25103.95	-4.94	-25062.00	-6.52	-25019.89	-7.94	-24978.23	-9.81	-24931.38	-6.48	-24913.62	-10.49
-25148.18	-5.64	-25106.27	-7.26	-25062.87	-7.40	-25016.70	-4.76	-24978.51	-10.09	-24937.32	-12.42	-24917.13	-14.00
-25147.46	-4.93	-25105.78	-6.77	-25062.18	-6.71	-25020.72	-8.77	-24975.76	-7.34	-24936.30	-11.41	-24915.45	-12.32
-25147.90	-5.37	-25105.45	-6.44	-25061.26	-5.78	-25021.16	-9.21	-24977.93	-9.51	-24932.33	-7.44	-24915.73	-12.60
-25147.21	-4.68	-25104.46	-5.45	-25062.03	-6.55	-25022.85	-10.90	-24977.01	-8.59	-24937.76	-12.87	-24911.86	-8.73
-25147.48	-4.95	-25102.98	-3.98	-25062.26	-6.78	-25018.85	-6.90	-24976.96	-8.54	-24932.91	-8.02	-24916.49	-13.37
-25147.91	-5.38	-25104.66	-5.66	-25062.54	-7.07	-25019.31	-7.36	-24970.90	-2.48	-24936.10	-11.21	-24915.63	-12.51
-25147.62	-5.09	-25104.80	-5.79	-25059.92	-4.44	-25018.77	-6.82	-24975.35	-6.93	-24938.10	-13.21	-24914.16	-11.03
-25146.23	-3.69	-25104.00	-5.00	-25062.13	-6.65	-25018.19	-6.24	-24978.25	-9.83	-24930.25	-5.36	-24914.68	-11.55
-25147.75	-5.22	-25106.05	-7.05	-25061.02	-5.54	-25019.56	-7.61	-24976.08	-7.66	-24933.67	-8.78	-24915.87	-12.74
-25148.40	-5.86	-25106.04	-7.04	-25062.46	-6.98	-25018.30	-6.35	-24979.71	-11.29	-24932.01	-7.11	-24917.62	-14.49
-25146.77	-4.24	-25105.79	-6.78	-25059.29	-3.81	-25019.19	-7.24	-24970.40	-1.98	-24932.09	-7.20	-24912.55	-9.42
-25147.25	-4.72	-25105.00	-5.99	-25059.26	-3.79	-25017.94	-5.99	-24977.33	-8.91	-24933.31	-8.42	-24914.57	-11.44
-25148.27	-5.73	-25103.52	-4.52	-25063.31	-7.84	-25019.78	-7.83	-24975.88	-7.46	-24935.07	-10.18	-24911.05	-7.92
-25147.72	-5.18	-25104.79	-5.79	-25063.01	-7.53	-25019.51	-7.56	-24977.31	-8.89	-24933.61	-8.72	-24915.32	-12.19
-25147.85	-5.32	-25103.88	-4.87	-25061.36	-5.88	-25020.58	-8.63	-24977.19	-8.77	-24936.23	-11.34	-24916.86	-13.73
-25147.50	-4.97	-25105.46	-6.46	-25063.96	-8.48	-25019.59	-7.64	-24978.29	-9.87	-24935.97	-11.07	-24915.63	-12.50
-25148.22	-5.68	-25101.64	-2.64	-25062.55	-7.07	-25018.18	-6.23	-24979.09	-10.67	-24938.36	-13.47	-24913.96	-10.83
-25148.45	-5.91	-25108.01	-9.00	-25064.89	-9.41	-25020.11	-8.16	-24980.54	-12.12	-24936.57	-11.68	-24915.04	-11.91
-25147.87	-5.34	-25103.93	-4.93	-25063.02	-7.54	-25019.66	-7.71	-24980.43	-12.01	-24934.10	-9.21	-24913.16	-10.03
-25148.77	-6.23	-25101.85	-2.84	-25063.68	-8.20	-25017.02	-5.07	-24972.48	-4.06	-24938.86	-13.97	-24911.40	-8.27
-25146.64	-4.11	-25106.30	-7.30	-25060.92	-5.44	-25020.11	-8.16	-24975.11	-6.69	-24932.77	-7.88	-24914.44	-11.31
-25148.27	-5.74	-25105.37	-6.36	-25062.99	-7.51	-25018.25	-6.30	-24975.63	-7.21	-24933.60	-8.70	-24914.34	-11.21
-25148.22	-5.68	-25105.53	-6.52	-25061.40	-5.92	-25023.47	-11.52	-24975.91	-7.49	-24932.60	-7.71	-24915.70	-12.57
-25147.24	-4.71	-25106.34	-7.34	-25059.85	-4.37	-25017.73	-5.78	-24977.82	-9.40	-24935.46	-10.57	-24916.16	-13.03
-25147.00	-4.47	-25104.10	-5.09	-25062.74	-7.26	-25022.22	-10.27	-24976.58	-8.16	-24937.61	-12.72	-24915.38	-12.25
-25149.39	-6.85	-25103.27	-4.26	-25060.81	-5.33	-25019.28	-7.33	-24976.84	-8.42	-24937.67	-12.77	-24912.87	-9.74
-25148.15	-5.62	-25105.62	-6.61	-25062.69	-7.21	-25021.12	-9.17	-24971.62	-3.20	-24931.06	-6.17	-24911.42	-8.29
-25147.61	-5.07	-25105.55	-6.54	-25061.35	-5.87	-25019.14	-7.19	-24980.74	-12.32	-24932.32	-7.43	-24914.67	-11.55
-25148.44	-5.90	-25101.94	-2.94	-25061.93	-6.46	-25021.63	-9.68	-24975.77	-7.35	-24936.89	-11.99	-24916.07	-12.94
-25147.53	-5.00	-25105.35	-6.35	-25061.22	-5.74	-25020.61	-8.66	-24977.65	-9.23	-24937.27	-12.38	-24916.37	-13.24
-25148.83	-6.30	-25103.68	-4.67	-25064.61	-9.13	-25020.58	-8.63	-24974.16	-5.74	-24938.21	-13.32	-24916.32	-13.19

	-25147.21	-4.68	-25103.12	-4.12	-25061.50	-6.02	-25018.55	-6.60	-24978.80	-10.38	-24934.25	-9.36	-24917.13	-14.01
	-25147.73	-5.20	-25101.65	-2.65	-25061.35	-5.87	-25019.16	-7.22	-24975.29	-6.87	-24934.54	-9.65	-24914.87	-11.75
	-25147.55	-5.01	-25105.81	-6.81	-25062.80	-7.32	-25023.26	-11.31	-24980.51	-12.09	-24936.29	-11.40	-24916.27	-13.14
	-25146.22	-3.68	-25105.89	-6.89	-25062.17	-6.70	-25019.50	-7.55	-24973.67	-5.25	-24936.98	-12.09	-24917.57	-14.45
	-25148.56	-6.03	-25103.12	-4.12	-25063.53	-8.05	-25019.18	-7.23	-24974.23	-5.81	-24937.26	-12.37	-24912.64	-9.51
	-25148.12	-5.58	-25106.45	-7.44	-25064.75	-9.28	-25019.42	-7.47	-24976.22	-7.80	-24933.61	-8.72	-24912.09	-8.96
	-25146.87	-4.34	-25105.24	-6.23	-25065.04	-9.56	-25018.32	-6.37	-24933.21	35.21	-24915.36	9.53	-24914.41	-11.28
	-25148.92	-6.39	-25107.36	-8.35	-25063.73	-8.26	-25018.83	-6.88	-24978.12	-9.70	-24939.17	-14.28	-24915.30	-12.17
	-25148.33	-5.80	-25105.04	-6.03	-25059.85	-4.37	-25019.80	-7.85	-24976.02	-7.60	-24934.60	-9.70	-24917.17	-14.04
	-25149.04	-6.51	-25104.71	-5.71	-25064.28	-8.80	-25016.15	-4.20	-24978.04	-9.62	-24935.31	-10.41	-24913.77	-10.64
	-25148.46	-5.92	-25105.01	-6.00	-25060.72	-5.24	-25017.93	-5.98	-24977.07	-8.65	-24934.26	-9.36	-24913.30	-10.17
	-25147.70	-5.17	-25104.05	-5.04	-25058.98	-3.51	-25015.69	-3.74	-24976.85	-8.43	-24931.50	-6.61	-24911.58	-8.45
	-25148.88	-6.34	-25105.76	-6.76	-25063.49	-8.01	-25019.58	-7.63	-24979.44	-11.02	-24935.38	-10.48	-24915.07	-11.94
	-25147.73	-5.20	-25105.83	-6.83	-25062.17	-6.70	-25018.57	-6.63	-24976.94	-8.52	-24938.04	-13.15	-24910.70	-7.57
	-25147.67	-5.13	-25104.38	-5.37	-25061.41	-5.93	-25017.30	-5.35	-24978.71	-10.29	-24937.51	-12.62	-24918.43	-15.30
	-25145.98	-3.45	-25104.64	-5.63	-25064.93	-9.45	-25020.85	-8.90	-24977.80	-9.38	-24936.18	-11.28	-24916.25	-13.12
	-25149.19	-6.66	-25102.80	-3.79	-25063.70	-8.23	-25021.29	-9.34	-24975.31	-6.89	-24934.50	-9.60	-24907.96	-4.83
	-25148.28	-5.75	-25107.36	-8.36	-25059.10	-3.63	-25020.40	-8.45	-24979.10	-10.68	-24937.51	-12.61	-24916.51	-13.39
	-25148.10	-5.57	-25104.10	-5.10	-25064.68	-9.20	-25021.45	-9.50	-24978.40	-9.98	-24934.40	-9.51	-24914.49	-11.36
	-25145.67	-3.13	-25104.51	-5.51			-25021.62	-9.67	-24978.42	-10.00	-24934.91	-10.02	-24914.89	-11.76
									-24974.20	-5.78	-24936.69	-11.80		
Average energies	-25147.96	-5.42	-25104.87	-5.87	-25062.20	-6.73	-25019.22	-7.27	-24976.59	-8.17	-24934.97	-10.07	-24914.62	-11.50

U

	ground state energy	solid solution energy												
concentration in solid solution (%)	3		6		9		12		15		18		21	
number of atoms	2		4		6		8		10		12		13	
	-25152.86	-5.31	-25115.35	-6.31	-25073.83	-3.29	-25040.69	-8.66	-25003.89	-10.38	-24964.73	-9.73	-24945.18	-9.43
	-25153.63	-6.07	-25109.33	-0.29	-25075.82	-5.29	-25041.65	-9.62	-25000.78	-7.27	-24965.01	-10.00	-24946.86	-11.11
	-25151.90	-4.35	-25110.96	-1.92	-25077.90	-7.36	-25039.49	-7.47	-25005.56	-12.05	-24967.26	-12.26	-24948.06	-12.31
	-25152.42	-4.87	-25113.61	-4.56	-25078.10	-7.56	-25042.38	-10.36	-25004.13	-10.62	-24963.67	-8.66	-24951.36	-15.62
	-25151.75	-4.20	-25116.40	-7.35	-25074.07	-3.54	-25037.25	-5.22	-25004.34	-10.82	-24966.02	-11.02	-24949.08	-13.33
	-25152.54	-4.99	-25115.09	-6.05	-25074.48	-3.95	-25040.45	-8.42	-25002.67	-9.16	-24966.09	-11.09	-24949.65	-13.90
	-25152.69	-5.14	-25114.66	-5.62	-25077.87	-7.34	-25036.61	-4.59	-25001.84	-8.33	-24968.51	-13.50	-24947.90	-12.15
	-25152.58	-5.03	-25114.40	-5.36	-25078.47	-7.93	-25042.19	-10.17	-25004.61	-11.09	-24963.02	-8.02	-24947.18	-11.43
	-25152.31	-4.76	-25109.99	-0.94	-25078.21	-7.68	-25040.71	-8.69	-25002.40	-8.88	-24969.97	-14.96	-24948.01	-12.26
	-25152.71	-5.16	-25114.36	-5.32	-25075.70	-5.17	-25036.70	-4.67	-25002.41	-8.90	-24966.52	-11.51	-24949.66	-13.91
	-25152.36	-4.81	-25115.36	-6.32	-25079.46	-8.92	-25039.96	-7.94	-25002.14	-8.62	-24964.51	-9.50	-24946.01	-10.26

-25152.23	-4.68	-25113.80	-4.75	-25075.45	-4.92	-25038.53	-6.50	-25002.84	-9.32	-24964.79	-9.79	-24943.87	-8.12
-25153.05	-5.50	-25116.30	-7.26	-25075.45	-4.91	-25038.89	-6.87	-25001.03	-7.52	-24965.61	-10.61	-24951.13	-15.38
-25151.68	-4.13	-25113.82	-4.78	-25077.73	-7.19	-25039.85	-7.83	-25002.48	-8.97	-24967.14	-12.14	-24950.93	-15.18
-25151.10	-3.55	-25112.52	-3.48	-25075.39	-4.85	-25041.70	-9.68	-24999.02	-5.51	-24967.89	-12.89	-24948.16	-12.41
-25151.54	-3.99	-25115.15	-6.11	-25078.90	-8.37	-25038.60	-6.57	-24999.11	-5.60	-24964.90	-9.90	-24947.33	-11.58
-25152.91	-5.35	-25114.21	-5.16	-25075.12	-4.59	-25041.52	-9.50	-25004.37	-10.86	-24963.54	-8.53	-24951.26	-15.51
-25152.45	-4.90	-25115.50	-6.46	-25079.49	-8.96	-25041.96	-9.93	-25002.40	-8.89	-24965.97	-10.96	-24947.44	-11.69
-25152.04	-4.49	-25112.37	-3.33	-25077.94	-7.40	-25039.47	-7.44	-25003.14	-9.62	-24965.01	-10.01	-24946.84	-11.09
-25153.31	-5.76	-25114.19	-5.15	-25078.57	-8.04	-25041.12	-9.09	-25001.36	-7.84	-24964.32	-9.31	-24942.80	-7.05
-25152.54	-4.98	-25113.00	-3.95	-25075.95	-5.42	-25036.26	-4.23	-25006.34	-12.83	-24967.33	-12.32	-24947.82	-12.07
-25151.87	-4.32	-25113.05	-4.00	-25077.62	-7.09	-25040.31	-8.29	-24997.48	-3.97	-24966.30	-11.30	-24947.62	-11.87
-25151.97	-4.41	-25139.05	-30.01	-25078.42	-7.88	-25038.47	-6.45	-25003.39	-9.88	-24964.39	-9.38	-24951.27	-15.52
-25150.25	-2.70	-25114.57	-5.53	-25078.80	-8.26	-25041.43	-9.41	-25002.03	-8.51	-24968.78	-13.78	-24949.77	-14.02
-25152.28	-4.73	-25112.18	-3.14	-25075.30	-4.76	-25042.15	-10.13	-25002.03	-8.51	-24964.29	-9.29	-24946.58	-10.83
-25152.85	-5.29	-25115.54	-6.49	-25077.14	-6.60	-25038.52	-6.50	-25002.82	-9.30	-24966.89	-11.89	-24948.65	-12.90
-25151.69	-4.13	-25113.62	-4.58	-25076.42	-5.88	-25039.09	-7.06	-25004.63	-11.12	-24965.57	-10.57	-24944.88	-9.13
-25152.89	-5.34	-25113.71	-4.67	-25076.38	-5.85	-25037.65	-5.62	-24998.81	-5.30	-24966.37	-11.37	-24948.62	-12.87
-25153.58	-6.02	-25116.22	-7.18	-25077.20	-6.67	-25041.89	-9.86	-25001.33	-7.81	-24961.68	-6.68	-24944.17	-8.42
-25151.21	-3.65	-25113.03	-3.99	-25077.74	-7.21	-25041.20	-9.18	-25006.11	-12.60	-24966.63	-11.63	-24949.85	-14.10
-25152.59	-5.04	-25115.28	-6.24	-25078.77	-8.23	-25042.95	-10.93	-25005.08	-11.57	-24965.00	-10.00	-24949.93	-14.18
-25152.47	-4.92	-25115.12	-6.08	-25078.36	-7.83	-25043.58	-11.56	-25004.51	-11.00	-24964.31	-9.31	-24946.21	-10.46
-25152.62	-5.07	-25114.65	-5.61	-25076.81	-6.28	-25042.35	-10.32	-25005.49	-11.98	-24969.23	-14.22	-25024.06	-88.31
-25153.04	-5.49	-25113.38	-4.34	-25074.15	-3.62	-25040.00	-7.97	-25003.45	-9.93	-24963.64	-8.63	-24950.88	-15.13
-25151.55	-4.00	-25114.66	-5.62	-25074.31	-3.77	-25037.72	-5.70	-25002.27	-8.75	-24967.13	-12.13	-24951.45	-15.70
-25146.52	1.03	-25114.54	-5.50	-25076.62	-6.09	-25042.20	-10.17	-25004.41	-10.90	-24969.52	-14.52	-24950.08	-14.33
-25150.78	-3.22	-25113.84	-4.79	-25077.96	-7.42	-25043.15	-11.13	-25003.92	-10.41	-24943.32	11.69	-24948.00	-12.25
-25152.83	-5.28	-25114.33	-5.29	-25078.07	-7.54	-25040.41	-8.39	-25003.48	-9.97	-24968.88	-13.88	-24950.97	-15.22
-25151.72	-4.16	-25108.73	0.31	-25077.00	-6.46	-25039.51	-7.49	-25004.11	-10.59	-24966.28	-11.27	-24946.62	-10.87
-25151.75	-4.20	-25113.88	-4.84	-25077.98	-7.45	-25040.56	-8.53	-25005.83	-12.32	-24966.37	-11.36	-24947.87	-12.12
-25153.30	-5.75	-25113.96	-4.91	-25076.00	-5.47	-25040.17	-8.14	-25005.29	-11.78	-24962.90	-7.90	-24948.61	-12.86
-25153.11	-5.56	-25115.65	-6.61	-25077.49	-6.95	-25038.90	-6.87	-25004.10	-10.59	-24967.67	-12.67	-24948.60	-12.85
-25151.49	-3.94	-25114.52	-5.48	-25076.95	-6.42	-25039.24	-7.21	-25001.40	-7.89	-24965.52	-10.52	-24947.65	-11.90
-25151.56	-4.01	-25113.41	-4.36	-25078.96	-8.43	-25039.75	-7.72	-25005.13	-11.62	-24966.85	-11.85	-24950.38	-14.63
-25153.46	-5.91	-25116.10	-7.06	-25077.27	-6.74	-25042.61	-10.58	-25003.29	-9.77	-24966.72	-11.72	-24950.07	-14.32
-25152.46	-4.90	-25115.87	-6.83	-25078.10	-7.57	-25038.76	-6.73	-25003.51	-10.00	-24966.84	-11.84	-24949.72	-13.97
-25151.51	-3.96	-25115.32	-6.28	-25077.97	-7.43	-25041.61	-9.58	-24998.57	-5.06	-24967.18	-12.17	-24949.31	-13.56
-25152.13	-4.58	-25115.39	-6.35	-25078.68	-8.14	-25039.28	-7.26	-25002.12	-8.60	-24963.27	-8.27	-24947.75	-12.00
-25152.51	-4.96	-25114.56	-5.52	-25077.70	-7.17	-25038.68	-6.66	-25003.41	-9.90	-24966.19	-11.18	-24948.90	-13.15
-25152.16	-4.61	-25115.49	-6.45	-25076.68	-6.15	-25039.44	-7.41	-25001.65	-8.13	-24967.15	-12.14	-24946.90	-11.15
-25150.00	-2.45	-25114.22	-5.18	-25079.00	-8.46	-25039.35	-7.33	-25002.63	-9.11	-24960.69	-5.69	-24943.26	-7.52
-25151.68	-4.12	-25115.91	-6.86	-25078.38	-7.85	-25040.35	-8.32	-25002.02	-8.50	-24964.79	-9.79	-24950.44	-14.69

-25151.91	-4.35	-25114.56	-5.51	-25078.90	-8.37	-25040.17	-8.14	-25005.23	-11.72	-24966.23	-11.22	-24949.07	-13.32
-25152.64	-5.09	-25115.58	-6.54	-25075.83	-5.30	-25037.57	-5.55	-25000.90	-7.39	-24963.64	-8.64	-24947.64	-11.89
-25152.33	-4.78	-25115.38	-6.34	-25078.56	-8.03	-25042.48	-10.45	-25002.50	-8.98	-24964.69	-9.69	-24947.94	-12.19
-25152.08	-4.53	-25113.44	-4.40	-25076.65	-6.11	-25039.13	-7.11	-25000.94	-7.43	-24964.12	-9.12	-24949.96	-14.21
-25147.23	0.33	-25114.36	-5.31	-25077.41	-6.88	-25036.07	-4.05	-25003.39	-9.87	-24964.42	-9.42	-24947.59	-11.84
-25152.61	-5.06	-25112.68	-3.63	-25078.84	-8.31	-25040.54	-8.52	-25005.52	-12.01	-24968.09	-13.09	-24950.35	-14.60
-25153.28	-5.72	-25112.94	-3.90	-25079.88	-9.35	-25040.20	-8.18	-25007.44	-13.93	-24966.97	-11.96	-24946.01	-10.26
-25151.15	-3.60	-25116.32	-7.28	-25076.94	-6.41	-25041.04	-9.02	-25005.51	-11.99	-24964.76	-9.75	-25023.22	-87.47
-25152.28	-4.73	-25114.32	-5.27	-25077.18	-6.65	-25039.69	-7.67	-25000.37	-6.85	-24965.49	-10.48	-24948.83	-13.08
-25147.03	0.52	-25116.34	-7.30	-25077.41	-6.88	-25038.56	-6.54	-25005.89	-12.37	-24961.91	-6.90	-24946.65	-10.90
-25149.71	-2.16	-25112.50	-3.46	-25075.02	-4.48	-25040.08	-8.06	-25004.98	-11.46	-24960.59	-5.59	-24944.60	-8.85
-25151.34	-3.78	-25114.21	-5.17	-25076.80	-6.27	-25041.78	-9.76	-25002.08	-8.56	-24965.90	-10.89	-24951.98	-16.23
-25153.84	-6.29	-25114.64	-5.60	-25077.55	-7.02	-25037.55	-5.53	-25003.43	-9.91	-24961.58	-6.58	-24945.40	-9.65
-25151.97	-4.42	-25115.25	-6.21	-25077.31	-6.78	-25039.46	-7.44	-25000.70	-7.18	-24969.72	-14.71	-24951.44	-15.69
-25151.01	-3.46	-25116.06	-7.02	-25074.94	-4.41	-25037.35	-5.33	-25004.18	-10.67	-24965.07	-10.06	-24950.17	-14.42
-25150.73	-3.18	-25114.34	-5.30	-25078.93	-8.39	-25041.90	-9.87	-25003.38	-9.86	-24967.55	-12.54	-24948.78	-13.03
-25151.85	-4.30	-25114.76	-5.71	-25080.76	-10.23	-25040.03	-8.01	-24996.52	-3.01	-24965.13	-10.13	-24950.31	-14.56
-25152.30	-4.74	-25112.67	-3.63	-25079.65	-9.11	-25041.61	-9.59	-24999.05	-5.53	-24969.64	-14.64	-24947.27	-11.52
-25151.99	-4.44	-25115.44	-6.40			-25037.53	-5.51	-25005.32	-11.81	-24969.54	-14.54	-24947.87	-12.13
-25152.90	-5.35	-25113.77	-4.73			-25040.81	-8.78	-25005.47	-11.96	-24964.89	-9.89	-24951.44	-15.69
-25152.16	-4.61	-25115.38	-6.34			-25037.44	-5.41	-24998.74	-5.23	-24968.21	-13.20	-24948.60	-12.85
-25153.15	-5.60	-25113.42	-4.38			-25038.62	-6.60	-25004.14	-10.62	-24962.02	-7.01	-24949.81	-14.06
-25153.26	-5.71	-25116.08	-7.04			-25040.73	-8.71	-25005.67	-12.16	-24967.70	-12.69	-24948.62	-12.87
-25152.02	-4.47	-25114.12	-5.08			-25041.34	-9.32	-25005.68	-12.17	-24964.18	-9.18	-24944.95	-9.20
-25152.20	-4.65	-25115.23	-6.19			-25041.31	-9.29	-25002.54	-9.03	-24966.57	-11.57	-24946.46	-10.71
-25151.99	-4.44	-25115.80	-6.76			-25038.70	-6.68	-25004.79	-11.27	-24962.03	-7.03	-24944.58	-8.83
-25153.04	-5.49	-25113.76	-4.72			-25039.08	-7.05	-25003.78	-10.27	-24966.96	-11.95	-24948.68	-12.93
-25151.65	-4.10	-25114.76	-5.72			-25037.78	-5.76	-25001.89	-8.37	-24966.99	-11.99	-24947.19	-11.44
-25152.27	-4.72					-25042.27	-10.25	-25004.81	-11.30	-24967.53	-12.52	-24946.40	-10.65
-25151.98	-4.43					-25037.77	-5.75	-25002.18	-8.67	-24967.44	-12.43	-24941.67	-5.92
-25151.01	-3.46					-25038.48	-6.46	-25001.09	-7.58	-24968.65	-13.64	-24946.40	-10.65
-25152.99	-5.44					-25038.46	-6.44	-25004.77	-11.26	-24964.37	-9.37	-24945.59	-9.84
-25151.97	-4.42					-25038.12	-6.09	-25003.53	-10.02	-24966.18	-11.17	-24944.71	-8.96
-25152.78	-5.23					-25041.07	-9.04	-25004.76	-11.24	-24966.67	-11.67	-24949.08	-13.33
-25151.94	-4.39					-25033.87	-1.84	-25004.24	-10.73	-24962.75	-7.74	-24942.80	-7.05
-25153.48	-5.93					-25039.12	-7.09	-25005.71	-12.19	-24963.15	-8.14	-24947.03	-11.28
-25153.22	-5.67					-25039.00	-6.98	-25001.82	-8.31	-24956.32	-1.32	-24950.95	-15.20
-25151.56	-4.01					-25037.74	-5.71	-25001.73	-8.21	-24965.18	-10.18	-24942.84	-7.09
-25153.32	-5.77					-25040.92	-8.89	-25004.98	-11.46	-24964.11	-9.11	-24949.91	-14.16
-25153.01	-5.46									-24966.83	-11.82	-24951.21	-15.46
										-24966.57	-11.56	-24947.52	-11.77

											-24967.65	-12.64	-24952.40	-16.65
											-24965.05	-10.04	-24947.04	-11.29
											-24961.99	-6.98	-24948.67	-12.92
											-24963.18	-8.18	-24950.27	-14.52
											-24967.2	-12.1656	-24948.3	-12.5201
											-24967.2	-12.1478	-24950.5	-14.7443
													-24949.2	-13.4645
Average energies	-25152.06	-4.51	-25114.60	-5.56	-25077.30	-6.76	-25039.81	-7.79	-25003.09	-9.57	-24965.40	-10.40	-24949.64	-13.89

$Ca_{1-x}M_xZrTi_{2-2x}Fe_{2x}O_7$ M=(Ce, Pu, Th, U)

Ti(3)Ti(3)

N.B. All stated energies are in eV

Ce

	ground state energy	solid solution energy													
concentration in solid solution (%)	3		6		9		12		15		18		21	
number of atoms	2		4		6		8		10		12		14	
	-25143.79	-0.91	-25096.39	3.32	-25048.52	8.02	-25002.09	11.28	-24952.52	17.68	-24906.41	20.61	-24859.63	24.22
	-25145.25	-2.36	-25096.38	3.33	-25051.08	5.46	-25001.79	11.58	-24956.46	13.74	-24906.49	20.53	-24860.14	23.71
	-25144.63	-1.74	-25095.70	4.02	-25048.03	8.52	-25000.15	13.22	-24955.65	14.55	-24905.23	21.79	-24858.85	25.00
	-25142.32	0.57	-25095.16	4.56	-25050.04	6.50	-24999.31	14.06	-24953.81	16.38	-24906.31	20.71	-24862.47	21.38
	-25142.15	0.74	-25096.79	2.93	-25051.06	5.49	-25002.19	11.17	-24953.65	16.54	-24907.22	19.81	-24862.80	21.05
	-25142.62	0.26	-25094.82	4.90	-25048.38	8.16	-25000.93	12.44	-24952.67	17.53	-24911.29	15.73	-24860.84	23.01
	-25144.61	-1.72	-25096.37	3.34	-25049.34	7.20	-25001.16	12.21	-24956.34	13.86	-24906.52	20.51	-24862.01	21.84
	-25142.12	0.77	-25096.75	2.97	-25048.14	8.41	-25003.47	9.90	-24955.15	15.04	-24906.07	20.95	-24861.40	22.45
	-25143.64	-0.76	-25096.87	2.84	-25047.45	9.10	-25002.12	11.25	-24956.82	13.38	-24909.50	17.53	-24863.27	20.58
	-25142.56	0.33	-25093.06	6.66	-25041.57	14.97	-24999.75	13.62	-24949.36	20.84	-24907.05	19.97	-24859.08	24.77
	-25144.30	-1.41	-25097.06	2.65	-25048.74	7.80	-25000.90	12.47	-24953.95	16.25	-24911.20	15.82	-24857.90	25.95
	-25142.53	0.36	-25097.19	2.53	-25047.77	8.77	-25002.36	11.01	-24954.95	15.25	-24901.56	25.46	-24861.55	22.30
	-25143.86	-0.97	-25095.94	3.78	-25049.53	7.01	-25003.39	9.98	-24954.10	16.10	-24906.57	20.45	-24861.67	22.18
	-25143.01	-0.12	-25095.32	4.40	-25045.89	10.65	-25004.56	8.81	-24954.99	15.21	-24911.25	15.77	-24863.57	20.28
	-25141.29	1.60	-25094.87	4.85	-25047.29	9.26	-25000.51	12.86	-24952.21	17.99	-24908.16	18.86	-24860.89	22.96
	-25142.92	-0.03	-25095.90	3.82	-25048.18	8.36	-24993.50	19.87	-24951.63	18.57	-24908.47	18.55	-24859.44	24.41
	-25143.00	-0.11	-25097.11	2.60	-25048.97	7.57	-25001.89	11.48	-24952.89	17.31	-24905.81	21.21	-24861.33	22.52
	-25143.20	-0.31	-25090.85	8.86	-25048.30	8.24	-25000.97	12.40	-24955.05	15.15	-24909.59	17.43	-24857.06	26.79
	-25143.51	-0.62	-25096.64	3.07	-25046.34	10.20	-25003.58	9.79	-24956.03	14.17	-24907.28	19.74	-24862.36	21.49
	-25143.25	-0.36	-25096.74	2.97	-25047.75	8.79	-24999.02	14.35	-24954.96	15.24	-24907.32	19.70	-24860.35	23.50
	-25143.18	-0.30	-25096.96	2.76	-25048.35	8.19	-24996.49	16.88	-24947.05	23.15	-24905.32	21.70	-24854.21	29.64
	-25143.24	-0.35	-25098.14	1.57	-25049.76	6.78	-25001.73	11.64	-24954.68	15.51	-24905.47	21.55	-24859.07	24.78
	-25141.62	1.27	-25095.93	3.79	-25049.61	6.93	-24998.44	14.93	-24951.68	18.52	-24905.65	21.37	-24863.71	20.13
	-25142.86	0.03	-25094.66	5.05	-25048.65	7.89	-25002.86	10.51	-24955.58	14.61	-24907.79	19.23	-24860.12	23.73
	-25142.81	0.07	-25097.25	2.46	-25046.44	10.10	-25001.27	12.10	-24952.46	17.74	-24909.20	17.82	-24863.01	20.84
	-25143.83	-0.94	-25094.40	5.32	-25051.29	5.26	-25002.50	10.87	-24956.75	13.45	-24900.33	26.69	-24860.00	23.85
	-25144.20	-1.31	-25096.03	3.69	-25047.69	8.85	-25001.15	12.22	-24953.15	17.05	-24906.18	20.84	-24864.49	19.36
	-25142.98	-0.09	-25095.47	4.25	-25048.83	7.71	-25001.01	12.35	-24954.80	15.39	-24908.32	18.70	-24864.08	19.77
	-25144.03	-1.14	-25098.03	1.69	-25046.99	9.55	-25000.75	12.62	-24956.96	13.23	-24906.16	20.87	-24862.47	21.38

-25142.79	0.09	-25096.20	3.52	-25049.11	7.43	-25000.92	12.45	-24958.06	12.14	-24906.53	20.49	-24855.90	27.95
-25144.83	-1.94	-25095.43	4.29	-25048.67	7.88	-25001.55	11.82	-24951.21	18.98	-24907.61	19.41	-24863.49	20.36
-25136.55	6.34	-25095.72	3.99	-25049.24	7.30	-25001.38	11.99	-24956.39	13.81	-24908.17	18.86	-24862.36	21.49
-25142.88	0.01	-25095.52	4.20	-25049.67	6.88	-25004.21	9.16	-24906.87	63.33	-24908.96	18.06	-24859.96	23.89
-25143.47	-0.58	-25093.19	6.52	-25049.78	6.76	-25003.95	9.42	-24954.42	15.77	-24863.04	63.98	-24865.46	18.39
-25143.81	-0.92	-25095.35	4.37	-25049.89	6.65	-24955.73	57.63	-24952.40	17.80	-24907.03	19.99	-24860.45	23.40
-25096.61	46.28	-25048.29	51.42	-25050.19	6.35	-25002.66	10.71	-24953.39	16.81	-24903.71	23.31	-24859.53	24.32
-25143.43	-0.54	-25095.87	3.84	-25047.25	9.30	-25000.82	12.55	-24953.18	17.02	-24908.53	18.49	-24860.38	23.47
-25143.34	-0.45	-25095.78	3.93	-25049.98	6.56	-24999.98	13.39	-24954.70	15.49	-24910.19	16.83	-24856.35	27.50
-25143.84	-0.95	-25096.02	3.69	-25049.53	7.01	-24998.93	14.44	-24955.27	14.93	-24905.88	21.14	-24859.65	24.20
-25142.63	0.25	-25095.67	4.05	-25050.14	6.41	-25001.49	11.88	-24950.37	19.83	-24904.99	22.03	-24860.17	23.68
-25142.90	-0.01	-25096.09	3.62	-25047.47	9.08	-25004.14	9.22	-24949.02	21.18	-24908.91	18.12	-24857.70	26.15
-25143.22	-0.33	-25097.46	2.26	-25049.70	6.84	-24999.24	14.13	-24956.63	13.56	-24907.26	19.76	-24858.56	25.29
-25144.47	-1.58	-25097.04	2.67	-25049.40	7.14	-25001.63	11.73	-24953.69	16.51	-24907.73	19.29	-24860.63	23.22
-25143.77	-0.88	-25097.15	2.57	-25050.50	6.05	-25002.39	10.98	-24951.75	18.44	-24905.40	21.63	-24862.07	21.78
-25145.25	-2.37	-25095.80	3.92	-25048.63	7.91	-25001.40	11.97	-24957.36	12.83	-24908.62	18.40	-24860.20	23.65
-25143.86	-0.97	-25096.28	3.43	-25047.36	9.18	-25001.69	11.68	-24951.97	18.22	-24908.38	18.64	-24861.56	22.29
-25143.28	-0.39	-25096.93	2.79	-25046.72	9.82	-25000.99	12.38	-24955.91	14.29	-24910.26	16.76	-24859.25	24.60
-25143.27	-0.39	-25095.63	4.09	-25047.04	9.51	-25001.77	11.60	-24954.77	15.43	-24909.41	17.61	-24861.32	22.53
-25142.73	0.16	-25094.40	5.32	-25049.82	6.73	-25001.81	11.56	-24954.38	15.82	-24904.59	22.44	-24861.44	22.41
-25143.68	-0.79	-25095.82	3.89	-25049.80	6.74	-25000.87	12.50	-24954.44	15.76	-24907.63	19.40	-24861.93	21.92
-25141.96	0.93	-25095.12	4.59	-25050.45	6.09	-25004.82	8.55	-24954.18	16.02	-24905.79	21.23	-24864.76	19.09
-25141.42	1.47	-25092.84	6.87	-25048.23	8.31	-25003.16	10.21	-24956.98	13.22	-24906.28	20.74	-24862.17	21.68
-25143.31	-0.42	-25095.67	4.05	-25047.83	8.71	-25005.79	7.58	-24955.09	15.10	-24907.01	20.01	-24863.01	20.84
-25143.35	-0.46	-25093.46	6.25	-25049.67	6.87	-25001.46	11.91	-24954.07	16.13	-24906.50	20.53	-24854.72	29.13
-25142.85	0.04	-25092.97	6.75	-25049.35	7.19	-25000.94	12.43	-24953.04	17.16	-24905.54	21.48	-24860.10	23.75
-25141.07	1.82	-25094.30	5.42	-25050.16	6.38	-25000.90	12.47	-24955.67	14.53	-24899.59	27.44	-24862.01	21.84
-25144.16	-1.27	-25095.31	4.41	-25047.63	8.91	-25003.22	10.14	-24951.84	18.36	-24907.04	19.99	-24861.93	21.92
-25143.26	-0.37	-25091.91	7.81	-25048.30	8.24	-25001.59	11.78	-24954.50	15.70	-24911.46	15.57	-24859.83	24.02
-25143.02	-0.13	-25095.39	4.33	-25049.09	7.45	-24999.53	13.84	-24954.69	15.51	-24906.30	20.72	-24860.07	23.78
-25142.57	0.32	-25096.57	3.14	-25049.49	7.05	-25000.42	12.95	-24956.22	13.97	-24909.28	17.74	-24859.94	23.91
-25142.89	-0.01	-25095.74	3.97	-25049.77	6.77	-25003.40	9.97	-24955.71	14.49	-24906.24	20.79	-24858.66	25.19
-25142.66	0.23	-25095.25	4.46	-25049.53	7.01	-24997.04	16.33	-24951.57	18.63	-24903.26	23.77	-24860.44	23.41
-25143.68	-0.79	-25095.95	3.77	-25047.79	8.76	-25003.33	10.04	-24953.08	17.12	-24902.71	24.32	-24861.83	22.02
-25144.28	-1.39	-25095.51	4.20	-25049.89	6.65	-25001.59	11.78	-24955.72	14.47	-24907.04	19.98	-24861.52	22.33
-25143.04	-0.15	-25096.42	3.30	-25049.87	6.67	-25000.20	13.17	-24953.54	16.66	-24907.32	19.70	-24859.76	24.09
-25143.18	-0.29	-25094.85	4.87	-25048.70	7.84	-25002.44	10.93	-24954.86	15.34	-24908.97	18.06	-24859.70	24.15
-25143.76	-0.87	-25095.75	3.97	-25048.89	7.65	-25002.61	10.76	-24952.14	18.06	-24907.64	19.38	-24862.27	21.58
-25143.15	-0.26	-25093.72	5.99	-25043.59	12.96	-25003.07	10.30	-24956.06	14.14	-24908.46	18.56	-24858.37	25.48
-25142.49	0.40	-25096.03	3.69	-25049.09	7.45	-25002.00	11.37	-24951.58	18.62	-24907.82	19.21	-24861.88	21.97
-25144.01	-1.12	-25096.74	2.97	-25045.66	10.88	-25002.22	11.15	-24956.08	14.11	-24908.23	18.79	-24860.90	22.95

-25142.96	-0.07	-25096.40	3.31	-25045.82	10.72	-25003.88	9.49	-24953.89	16.30	-24907.26	19.76	-24862.92	20.93
-25143.60	-0.71	-25094.93	4.79	-25049.59	6.96	-25001.12	12.25	-24906.30	63.89	-24906.23	20.79	-24862.78	21.07
-25143.32	-0.43	-25097.39	2.32	-25047.14	9.40	-25000.89	12.48	-24954.28	15.92	-24909.27	17.75	-24862.24	21.61
-25142.75	0.14	-25096.66	3.06	-25048.93	7.61	-24953.26	60.11	-24956.03	14.16	-24910.89	16.13	-24856.49	27.36
-25144.78	-1.89	-25095.95	3.77	-25001.50	55.04	-24999.70	13.67	-24955.89	14.30	-24859.45	67.57	-24860.52	23.33
-25142.92	-0.03	-25094.70	5.02	-25047.68	8.86	-24999.58	13.79	-24952.60	17.60	-24906.76	20.26	-24864.19	19.66
-25143.81	-0.92	-25097.48	2.23	-25047.65	8.89	-24999.58	13.79	-24951.01	19.18	-24910.12	16.91	-24858.11	25.74
-25142.04	0.84	-25096.56	3.15	-25048.50	8.04	-25000.51	12.86	-24953.22	16.98	-24903.70	23.32	-24859.45	24.40
-25144.29	-1.40	-25097.20	2.51	-25047.68	8.86	-25003.01	10.36	-24953.86	16.33	-24910.31	16.71	-24859.75	24.10
-25143.24	-0.35	-25095.21	4.50	-25049.39	7.15	-25000.38	12.99	-24954.16	16.04	-24910.96	16.06	-24862.06	21.79
-25144.30	-1.41	-25096.40	3.31	-25048.28	8.26	-25002.94	10.43	-24954.33	15.86	-24911.01	16.01	-24864.20	19.65
-25143.69	-0.81	-25094.88	4.84	-25048.31	8.23	-25000.30	13.07	-24954.09	16.10	-24901.00	26.02	-24859.47	24.38
-25141.64	1.25	-25093.96	5.75	-25047.36	9.18	-25003.68	9.69	-24957.68	12.52	-24907.96	19.06	-24859.53	24.31
-25143.63	-0.74	-25095.31	4.41	-25048.63	7.92	-25003.08	10.29	-24955.46	14.73	-24909.31	17.71	-24859.35	24.50
-25144.06	-1.17	-25096.11	3.60	-25043.99	12.55	-25000.63	12.74	-24954.96	15.24	-24909.89	17.14	-24860.66	23.19
-25143.36	-0.47	-25095.57	4.14	-25046.99	9.55	-25003.45	9.91	-24950.14	20.06	-24905.01	22.01	-24858.06	25.79
-25144.06	-1.17	-25095.95	3.77	-25049.40	7.14	-25002.44	10.93	-24953.66	16.53	-24902.78	24.24	-24862.09	21.76
-25143.06	-0.17	-25095.04	4.68	-25049.78	6.76	-25004.25	9.11	-24949.22	20.97	-24862.88	64.14	-24861.89	21.96
-25143.85	-0.96	-25095.00	4.72	-25048.79	7.75	-24998.43	14.94	-24957.64	12.55	-24906.11	20.91	-24859.26	24.59
-25143.31	-0.42	-25095.10	4.62	-25047.38	9.16	-24999.02	14.35	-24954.95	15.25	-24908.10	18.92	-24860.81	23.04
-25142.38	0.51	-25094.36	5.35	-25046.64	9.90	-25001.23	12.14	-24952.94	17.25	-24907.09	19.93	-24861.22	22.63
-25141.70	1.18	-25097.24	2.48	-25048.50	8.04	-24999.19	14.18	-24954.83	15.36	-24907.67	19.35	-24860.76	23.09
-25143.14	-0.25	-25096.97	2.74	-25050.64	5.91	-25002.94	10.43	-24954.64	15.56	-24908.52	18.50	-24864.11	19.74
-25143.62	-0.73	-25098.21	1.50	-25050.49	6.05	-25002.16	11.21	-24954.88	15.32	-24909.78	17.25	-24856.62	27.23
-25143.52	-0.63	-25094.10	5.62	-25049.13	7.42	-25000.11	13.26	-24953.70	16.50	-24907.30	19.73	-24859.03	24.82
-25143.29	-0.40	-25094.90	4.82	-25049.17	7.37	-25001.37	12.00	-24955.77	14.42	-24907.27	19.76	-24860.29	23.56
-25142.54	0.35	-25096.79	2.93	-25048.43	8.11	-25004.17	9.20	-24957.08	13.11	-24907.05	19.98	-24863.19	20.66
-25142.77	0.11	-25093.87	5.84	-25050.19	6.35	-25004.75	8.62	-24954.15	16.04	-24909.25	17.77	-24862.38	21.47
-25143.41	-0.52	-25097.31	2.40	-25049.70	6.84	-25000.56	12.80	-24954.19	16.01	-24909.25	17.77	-24862.43	21.42
-25142.88	0.01	-25097.62	2.10	-25050.11	6.43	-24998.58	14.79	-24955.57	14.63	-24905.47	21.55	-24858.89	24.96
-25142.90	-0.01	-25094.46	5.25	-25046.34	10.20	-24998.86	14.51	-24951.22	18.98	-24904.96	22.07	-24852.55	31.30
-25143.08	-0.19	-25094.87	4.85	-25049.10	7.44	-25003.34	10.03	-24953.55	16.65	-24907.65	19.38	-24863.32	20.53
-25143.22	-0.34	-25098.12	1.59	-25047.45	9.09	-25002.74	10.63	-24952.87	17.32	-24910.02	17.00	-24860.17	23.68
-25144.46	-1.57	-25095.52	4.19	-25049.97	6.57	-24998.53	14.84	-24955.31	14.89	-24906.26	20.76	-24858.96	24.89
-25143.83	-0.94	-25095.76	3.96	-25046.32	10.22	-24999.31	14.06	-24956.61	13.59	-24907.61	19.41	-24858.32	25.53
-25142.88	0.01	-25095.86	3.86	-25048.46	8.09	-25001.67	11.70	-24955.31	14.89	-24908.93	18.09	-24861.18	22.67
-25144.85	-1.97	-25094.86	4.85	-25049.25	7.30	-25004.44	8.93	-24952.77	17.42	-24907.78	19.24	-24859.04	24.81
-25142.79	0.10	-25096.54	3.18	-25048.75	7.79	-25003.45	9.91	-24955.78	14.41	-24905.93	21.09	-24861.98	21.86
-25142.59	0.29	-25096.60	3.12	-25048.87	7.68	-25002.44	10.93	-24956.57	13.63	-24907.23	19.79	-24863.87	19.98
-25143.36	-0.47	-25095.73	3.98	-25049.68	6.86	-25000.85	12.52	-24955.75	14.45	-24904.20	22.83	-24860.91	22.94
-25143.86	-0.97	-25096.75	2.97	-25048.16	8.38	-25002.43	10.94	-24954.88	15.32	-24904.54	22.48	-24861.59	22.26

	-25141.93	0.96	-25096.94	2.78	-25048.12	8.42	-25000.48	12.89	-24955.05	15.15	-24903.09	23.93	-24860.30	23.55
	-25144.39	-1.50	-25095.18	4.54	-25049.01	7.54	-25003.16	10.21			-24906.56	20.47	-24861.82	22.03
	-25143.32	-0.43	-25096.80	2.92	-25049.52	7.02	-24999.03	14.34			-24904.73	22.29	-24860.98	22.87
	-25143.35	-0.47	-25095.19	4.53	-25048.42	8.12	-25001.56	11.81			-24909.00	18.02	-24859.24	24.61
	-25143.51	-0.62	-25094.83	4.88	-25046.70	9.84					-24905.86	21.17		
	-25144.42	-1.53	-25094.26	5.46	-25045.91	10.63					-24908.01	19.01		
	-25140.96	1.93	-25096.43	3.28	-25049.52	7.02								
Average energies	-25142.81	0.08	-25095.34	4.38	-25048.12	8.42	-25000.65	12.72	-24953.36	16.84	-24905.98	21.05	-24860.67	23.18

Pu

	ground state energy	solid solution energy												
concentration in solid solution (%)	3		6		9		12		15		18		21	
number of atoms	2		4		6		8		10		12		14	
	-25138.63	-1.73	-25083.45	4.29	-25030.88	7.71	-24976.35	13.08	-24922.98	17.28	-24874.33	16.78	-24822.00	19.95
	-25136.19	0.72	-25085.85	1.90	-25033.01	5.57	-24976.59	12.84	-24925.71	14.56	-24871.95	19.16	-24819.76	22.19
	-25133.90	3.00	-25085.35	2.40	-25032.69	5.89	-24976.53	12.90	-24924.75	15.51	-24873.11	18.00	-24861.98	-20.03
	-25136.63	0.28	-25085.39	2.36	-25026.47	12.11	-24976.57	12.85	-24925.04	15.23	-24875.44	15.67	-24820.26	21.69
	-25137.12	-0.22	-25084.61	3.14	-25031.91	6.68	-24979.84	9.58	-24926.42	13.85	-24873.03	18.08	-24821.08	20.87
	-25137.29	-0.39	-25083.38	4.37	-25031.63	6.95	-24975.75	13.68	-24927.70	12.57	-24870.80	20.31	-24814.36	27.59
	-25137.47	-0.57	-25084.65	3.09	-25030.70	7.89	-24974.10	15.32	-24924.47	15.80	-24874.45	16.66	-24820.23	21.72
	-25137.58	-0.67	-25084.07	3.68	-25031.08	7.51	-24978.28	11.15	-24923.29	16.98	-24872.54	18.57	-24821.13	20.82
	-25136.28	0.62	-25083.70	4.05	-25024.82	13.77	-24975.55	13.88	-24924.80	15.47	-24873.52	17.59	-24820.92	21.03
	-25136.19	0.71	-25084.17	3.57	-25029.99	8.60	-24979.22	10.21	-24927.47	12.80	-24876.54	14.57	-24820.02	21.93
	-25136.88	0.02	-25080.59	7.15	-25031.93	6.65	-24977.89	11.54	-24927.28	12.99	-24874.12	16.99	-24822.43	19.52
	-25138.11	-1.21	-25081.97	5.77	-25033.25	5.33	-24973.85	15.58	-24925.49	14.78	-24874.51	16.60	-24817.47	24.48
	-25137.12	-0.22	-25084.89	2.85	-25030.62	7.96	-24975.40	14.03	-24927.07	13.19	-24874.29	16.82	-24818.45	23.50
	-25138.23	-1.32	-25082.26	5.49	-25029.49	9.09	-24979.24	10.19	-24924.98	15.29	-24875.90	15.20	-24819.97	21.98
	-25135.80	1.10	-25082.75	5.00	-25031.34	7.25	-24977.45	11.97	-24924.78	15.49	-24874.39	16.72	-24820.26	21.69
	-25137.17	-0.27	-25083.20	4.54	-25032.84	5.74	-24978.29	11.14	-24925.63	14.63	-24874.18	16.93	-24819.28	22.67
	-25138.17	-1.26	-25083.98	3.76	-25029.33	9.26	-24978.40	11.03	-24925.61	14.66	-24870.07	21.04	-24819.13	22.82
	-25136.82	0.08	-25085.39	2.35	-25030.00	8.59	-24979.95	9.48	-24925.66	14.61	-24874.27	16.84	-24820.34	21.61
	-25137.34	-0.44	-25084.91	2.84	-25030.76	7.83	-24976.98	12.45	-24927.41	12.86	-24868.10	23.01	-24820.12	21.83
	-25137.18	-0.28	-25083.60	4.15	-25030.37	8.21	-24978.13	11.30	-24924.85	15.42	-24874.11	17.00	-24819.65	22.30
	-25138.67	-1.77	-25083.38	4.36	-25032.03	6.56	-24977.62	11.80	-24926.98	13.29	-24874.13	16.98	-24820.85	21.10
	-25137.70	-0.80	-25081.79	5.96	-25033.26	5.32	-24981.29	8.14	-24924.84	15.43	-24873.42	17.69	-24821.66	20.29
	-25137.84	-0.94	-25082.31	5.43	-25029.61	8.98	-24979.50	9.93	-24923.57	16.69	-24872.14	18.97	-24819.07	22.88
	-25137.45	-0.54	-25085.16	2.58	-25029.49	9.10	-24976.44	12.99	-24925.57	14.70	-24874.25	16.86	-24822.19	19.76
	-25137.26	-0.36	-25084.12	3.62	-25030.44	8.15	-24977.25	12.18	-24924.30	15.97	-24872.83	18.28	-24819.03	22.92

-25137.23	-0.33	-25083.12	4.63	-25032.48	6.11	-24977.89	11.54	-24919.56	20.71	-24870.63	20.48	-24820.36	21.59
-25139.12	-2.22	-25083.80	3.94	-25033.19	5.40	-24978.65	10.78	-24922.83	17.44	-24872.44	18.67	-24822.17	19.78
-25138.25	-1.34	-25085.82	1.92	-25030.84	7.74	-24979.54	9.89	-24924.93	15.34	-24873.63	17.48	-24819.93	22.02
-25135.91	0.99	-25083.77	3.98	-25032.34	6.24	-24977.59	11.84	-24927.41	12.86	-24871.06	20.05	-24819.83	22.12
-25136.99	-0.08	-25085.79	1.95	-25027.32	11.26	-24979.30	10.12	-24923.90	16.37	-24873.80	17.31	-24818.76	23.19
-25138.46	-1.55	-25084.41	3.34	-25032.89	5.70	-24978.26	11.17	-24924.18	16.09	-24870.97	20.14	-24821.50	20.45
-25138.48	-1.58	-25084.36	3.38	-25030.42	8.16	-24981.16	8.27	-24924.87	15.40	-24875.41	15.70	-24820.04	21.91
-25135.05	1.85	-25084.58	3.16	-25031.31	7.27	-24979.00	10.43	-24925.35	14.92	-24874.89	16.22	-24820.79	21.16
-25136.72	0.18	-25084.76	2.98	-25033.28	5.30	-24976.01	13.42	-24927.71	12.55	-24875.46	15.65	-24819.59	22.36
-25136.88	0.02	-25084.90	2.84	-25032.98	5.60	-24978.53	10.90	-24926.49	13.77	-24873.61	17.50	-24821.07	20.89
-25137.31	-0.41	-25085.33	2.41	-25032.65	5.93	-24979.24	10.19	-24926.47	13.80	-24871.93	19.18	-24821.92	20.03
-25136.86	0.04	-25085.64	2.10	-25030.96	7.63	-24976.08	13.35	-24925.35	14.91	-24872.18	18.93	-24823.59	18.36
-25137.39	-0.48	-25083.43	4.31	-25029.71	8.87	-24977.12	12.31	-24925.87	14.39	-24875.24	15.87	-24815.61	26.34
-25137.39	-0.48	-25084.89	2.86	-25031.49	7.09	-24975.57	13.85	-24926.84	13.43	-24875.89	15.22	-24822.07	19.88
-25137.15	-0.24	-25085.74	2.00	-25032.79	5.79	-24974.70	14.73	-24927.43	12.84	-24873.89	17.22	-24819.80	22.15
-25138.04	-1.14	-25082.86	4.88	-25031.09	7.50	-24976.45	12.97	-24924.25	16.02	-24871.80	19.31	-24822.23	19.72
-25138.04	-1.13	-25084.43	3.32	-25033.11	5.48	-24978.37	11.06	-24926.32	13.95	-24873.48	17.63	-24822.67	19.29
-25136.33	0.57	-25084.96	2.79	-25030.40	8.18	-24977.79	11.64	-24926.01	14.26	-24873.89	17.22	-24820.43	21.52
-25135.72	1.19	-25083.94	3.80	-25031.24	7.35	-24976.18	13.25	-24923.91	16.36	-24868.89	22.22	-24820.62	21.33
-25137.00	-0.10	-25083.63	4.11	-25032.67	5.91	-24976.57	12.86	-24925.43	14.84	-24874.93	16.18	-24822.62	19.33
-25137.86	-0.96	-25084.48	3.27	-25030.96	7.63	-24979.35	10.07	-24924.74	15.52	-24871.82	19.29	-24818.13	23.82
-25137.79	-0.89	-25083.02	4.72	-25031.45	7.14	-24975.71	13.71	-24924.56	15.70	-24873.47	17.64	-24819.98	21.97
-25137.64	-0.73	-25083.65	4.09	-25029.64	8.94	-24977.02	12.40	-24926.44	13.83	-24872.89	18.22	-24824.37	17.58
-25136.27	0.63	-25086.15	1.60	-25028.20	10.38	-24978.54	10.89	-24925.46	14.81	-24873.83	17.28	-24820.77	21.18
-25138.32	-1.42	-25085.81	1.93	-25032.42	6.17	-24976.91	12.52	-24929.05	11.21	-24872.05	19.06	-24820.71	21.24
-25137.71	-0.81	-25084.61	3.13	-25032.48	6.11	-24977.92	11.51	-24925.35	14.91	-24874.59	16.52	-24819.21	22.74
-25138.25	-1.35	-25083.74	4.01	-25027.79	10.80	-24979.07	10.36	-24923.57	16.70	-24873.24	17.87	-24820.22	21.73
-25137.17	-0.27	-25083.49	4.25	-25031.07	7.52	-24980.10	9.32	-24923.02	17.25	-24873.24	17.87	-24820.99	20.96
-25137.20	-0.29	-25084.57	3.18	-25028.74	9.85	-24978.54	10.88	-24926.02	14.25	-24871.96	19.15	-24820.12	21.83
-25138.22	-1.32	-25085.41	2.34	-25030.73	7.86	-24979.32	10.11	-24925.51	14.76	-24875.17	15.94	-24819.24	22.71
-25136.04	0.86	-25086.86	0.89	-25031.40	7.19	-24979.17	10.26	-24925.47	14.80	-24872.28	18.83	-24823.79	18.16
-25138.59	-1.69	-25085.36	2.39	-25032.87	5.71	-24979.34	10.08	-24926.92	13.35	-24870.68	20.43	-24820.40	21.55
-25137.18	-0.28	-25086.90	0.85	-25032.56	6.03	-24976.21	13.22	-24926.04	14.23	-24875.54	15.57	-24820.11	21.84
-25138.58	-1.68	-25082.71	5.03	-25032.00	6.58	-24980.12	9.31	-24920.77	19.50	-24872.93	18.18	-24821.93	20.02
-25135.97	0.94	-25082.11	5.64	-25030.20	8.38	-24977.13	12.29	-24925.40	14.87	-24872.59	18.52	-24821.60	20.35
-25138.09	-1.19	-25084.83	2.91	-25029.68	8.91	-24979.65	9.77	-24924.62	15.65	-24873.53	17.58	-24819.48	22.47
-25135.79	1.11	-25083.03	4.71	-25031.49	7.09	-24980.36	9.07	-24928.18	12.09	-24871.75	19.35	-24820.43	21.52
-25136.58	0.32	-25085.18	2.57	-25031.84	6.75	-24979.33	10.10	-24923.05	17.22	-24873.72	17.39	-24820.47	21.48
-25137.95	-1.05	-25083.90	3.85	-25030.72	7.87	-24976.77	12.65	-24928.06	12.21	-24873.83	17.28	-24818.40	23.55
-25136.10	0.80	-25084.66	3.08	-25030.42	8.17	-24978.67	10.76	-24926.47	13.80	-24874.39	16.72	-24823.69	18.27
-25137.44	-0.54	-25083.48	4.26	-25033.13	5.46	-24978.03	11.40	-24927.02	13.25	-24873.04	18.07	-24822.31	19.64

-25136.87	0.03	-25085.07	2.67	-25028.52	10.06	-24974.70	14.73	-24924.86	15.41	-24874.46	16.65	-24819.48	22.47
-25137.60	-0.70	-25086.44	1.30	-25030.15	8.44	-24976.55	12.88	-24926.38	13.89	-24874.86	16.25	-24819.66	22.29
-25138.19	-1.29	-25084.76	2.98	-25031.03	7.56	-24977.07	12.36	-24924.14	16.13	-24872.89	18.22	-24821.22	20.73
-25136.61	0.30	-25082.40	5.34	-25032.28	6.30	-24978.99	10.44	-24925.08	15.19	-24874.21	16.90	-24822.89	19.06
-25138.09	-1.19	-25084.57	3.18	-25032.39	6.19	-24980.42	9.01	-24927.07	13.19	-24865.94	25.17	-24820.00	21.96
-25136.60	0.31	-25085.24	2.50	-25029.34	9.24	-24978.99	10.44	-24922.31	17.96	-24874.75	16.36	-24820.74	21.21
-25136.32	0.59	-25082.76	4.99	-25032.92	5.66	-24979.48	9.95	-24926.24	14.03	-24869.42	21.69	-24821.13	20.82
-25133.53	3.38	-25082.01	5.74	-25032.62	5.96	-24977.76	11.66	-24922.86	17.41	-24869.94	21.17	-24817.89	24.06
-25137.68	-0.78	-25083.42	4.33	-25029.18	9.40	-24977.11	12.32	-24924.91	15.36	-24871.36	19.75	-24820.74	21.21
-25137.71	-0.81	-25084.11	3.64	-25033.29	5.30	-24976.87	12.56	-24925.97	14.30	-24874.73	16.38	-24818.57	23.38
-25137.80	-0.90	-25082.25	5.49	-25028.77	9.82	-24979.32	10.10	-24925.48	14.79	-24876.13	14.98	-24823.14	18.81
-25138.14	-1.24	-25083.66	4.09	-25032.56	6.02	-24978.32	11.11	-24929.75	10.52	-24870.64	20.47	-24819.45	22.50
-25137.46	-0.56	-25084.87	2.87	-25032.79	5.79	-24978.41	11.01	-24923.67	16.60	-24873.34	17.77	-24822.16	19.79
-25136.91	0.00	-25085.35	2.39	-25031.38	7.20	-24977.63	11.80	-24926.03	14.24	-24821.13	69.98	-24820.84	21.11
-25138.17	-1.26	-25084.76	2.98	-25029.71	8.88	-24978.68	10.75	-24926.29	13.98	-24869.13	21.98	-24817.81	24.15
-25136.92	-0.01	-25083.34	4.40	-25030.89	7.70	-24979.09	10.34	-24924.95	15.32	-24870.03	21.08	-24820.17	21.78
-25137.04	-0.14	-25081.52	6.23	-25029.14	9.44	-24980.36	9.06	-24919.97	20.30	-24868.94	22.17	-24820.55	21.40
-25137.31	-0.41	-25084.03	3.72	-25031.97	6.62	-24977.38	12.04	-24926.44	13.83	-24871.30	19.81	-24817.99	23.96
-25135.98	0.92	-25083.83	3.92	-25028.39	10.20	-24978.56	10.87	-24922.48	17.79	-24873.41	17.70	-24822.32	19.63
-25137.01	-0.11	-25084.35	3.40	-25031.76	6.82	-24976.49	12.94	-24923.86	16.41	-24869.98	21.13	-24818.48	23.47
-25136.84	0.06	-25085.47	2.27	-25032.01	6.58	-24979.18	10.25	-24923.41	16.86	-24871.91	19.20	-24823.06	18.89
-25137.69	-0.79	-25086.26	1.48	-25031.78	6.81	-24976.64	12.79	-24925.90	14.37	-24874.73	16.38	-24822.48	19.47
-25136.90	0.00	-25084.40	3.34	-25029.98	8.60	-24975.43	14.00	-24928.87	11.40	-24871.21	19.90	-24818.30	23.65
-25137.79	-0.89	-25083.29	4.45	-25032.16	6.42	-24979.49	9.94	-24923.09	17.18	-24872.61	18.50	-24820.46	21.50
-25137.22	-0.31	-25084.27	3.47	-25031.25	7.34	-24978.37	11.06	-24926.88	13.39	-24873.11	18.00	-24814.61	27.34
-25136.95	-0.04	-25084.44	3.30	-25030.78	7.80	-24980.57	8.86	-24926.08	14.19	-24871.85	19.26	-24820.40	21.55
-25138.87	-1.97	-25083.65	4.09	-25030.06	8.53	-24979.24	10.19	-24926.19	14.08	-24872.60	18.51	-24819.53	22.42
-25137.84	-0.93	-25084.69	3.05	-25030.95	7.64	-24979.34	10.08	-24926.30	13.97	-24872.83	18.28	-24818.33	23.62
-25139.06	-2.16	-25084.54	3.20	-25031.78	6.81	-24979.13	10.30	-24926.40	13.87	-24821.31	69.80	-24823.40	18.55
-25137.69	-0.79	-25085.51	2.24	-25032.59	5.99	-24979.95	9.48	-24927.64	12.62	-24875.07	16.04	-24819.55	22.40
-25136.92	-0.02	-25083.45	4.30	-25031.64	6.94	-24976.80	12.63	-24871.92	68.35	-24876.06	15.05	-24819.27	22.69
-25135.96	0.95	-25085.84	1.90	-25030.97	7.61	-24977.22	12.21	-24925.14	15.13	-24873.74	17.37	-24820.35	21.60
-25136.83	0.08	-25081.96	5.79	-25031.01	7.58	-24976.56	12.86	-24926.19	14.08	-24875.13	15.98	-24819.58	22.37
-25136.81	0.10	-25087.42	0.33	-25031.42	7.17	-24979.52	9.91	-24924.91	15.36	-24875.25	15.86	-24822.37	19.58
-25137.46	-0.56	-25083.62	4.13	-25029.23	9.35	-24976.25	13.17	-24922.80	17.47	-24871.10	20.01	-24821.24	20.71
-25138.49	-1.59	-25083.83	3.91	-25032.60	5.99	-24979.76	9.66	-24927.35	12.92	-24874.14	16.97	-24820.24	21.71
-25137.28	-0.37	-25083.69	4.06	-25030.17	8.41	-24977.31	12.12	-24925.37	14.90	-24874.27	16.84	-24816.72	25.23
-25137.94	-1.03	-25085.39	2.36	-25031.13	7.46	-24979.97	9.46	-24925.35	14.92	-24872.96	18.15	-24819.31	22.64
-25138.32	-1.41	-25084.38	3.37	-25032.89	5.69	-24977.81	11.62	-24926.41	13.86	-24872.00	19.11	-24821.94	20.01
-25137.07	-0.17	-25084.52	3.22	-25030.44	8.15	-24977.87	11.55	-24927.84	12.43	-24871.86	19.25	-24819.14	22.81
-25137.82	-0.92	-25084.04	3.70	-25032.66	5.92	-24977.29	12.14	-24926.60	13.67	-24873.67	17.44	-24821.29	20.66

	-25137.82	-0.92	-25083.64	4.11	-25030.03	8.56	-24980.96	8.47	-24928.54	11.73	-24874.70	16.41	-24819.41	22.54
	-25138.96	-2.06	-25082.36	5.38	-25029.73	8.85	-24978.36	11.07	-24924.65	15.62	-24873.26	17.85	-24819.79	22.16
	-25137.73	-0.83	-25083.59	4.15	-25029.10	9.49	-24977.36	12.07	-24925.90	14.37	-24873.09	18.02	-24820.65	21.30
	-25137.39	-0.49	-25083.03	4.71	-25032.23	6.35	-24980.46	8.97	-24925	15.27433	-24872.11	18.99	-24821.62	20.33
	-25138.75	-1.85	-25085.02	2.73	-25029.99	8.59	-24922.5	66.95727	-24925.2	15.09013	-24821.30	69.81	-24819.9	22.07393
	-25136.77	0.13	-25083.24	4.50	-25029.93	8.66	-24978.3	11.15519	-24877.4	62.82361	-24872.86	18.25	-24816.4	25.58176
	-25137.68	-0.78	-25030.02	57.72	-24977.00	61.58	-24977	12.39393	-24926	14.31275	-24871.3	19.80135	-24821.8	20.12987
	-25084.81	52.09	-25085.77	1.98	-25028.14	10.45	-24977.8	11.60351	-24925.4	14.8254	-24874.9	16.17537	-24818.4	23.57531
	-25137.15	-0.25	-25084.99	2.75	-25032.89	5.70	-24978.9	10.53572	-24923.9	16.35456	-24872.1	19.04699		
	-25137.33	-0.43	-25085.4	2.321192	-25030.1	8.470109			-24926.7	13.60151				
	-25136.9	0.027494	-25084.5	3.281741	-25030.3	8.296498								
	-25137	-0.11984												
Average energies	-25136.86	0.05	-25083.76	3.98	-25030.57	8.02	-24977.54	11.88	-24924.58	15.69	-24871.69	19.41	-24820.68	21.27
**Th**	ground state energy	solid solution energy												
concentration in solid solution (%)	3		6		9		12		15		18		21	
number of atoms	2		4		6		8		10		12		14	
	-25142.63	-0.10	-25094.68	4.33	-25047.68	7.80	-24996.17	15.78	-24954.99	13.43	-24905.13	19.76	-24858.43	22.94
	-25143.78	-1.24	-25094.98	4.03	-25046.57	8.91	-24999.83	12.12	-24955.13	13.29	-24907.53	17.36	-24855.05	26.32
	-25142.51	0.03	-25096.48	2.52	-25046.85	8.62	-25002.16	9.78	-24950.45	17.97	-24906.27	18.63	-24858.15	23.22
	-25143.00	-0.47	-25095.95	3.05	-25048.99	6.49	-24995.98	15.97	-24953.35	15.07	-24907.53	17.36	-24856.08	25.28
	-25143.07	-0.53	-25096.22	2.79	-25047.00	8.47	-25002.23	9.72	-24952.21	16.21	-24907.33	17.56	-24856.83	24.54
	-25143.13	-0.59	-25094.41	4.60	-25047.63	7.85	-24999.30	12.65	-24953.50	14.92	-24907.17	17.72	-24853.66	27.70
	-25142.90	-0.37	-25094.95	4.06	-25048.26	7.22	-25001.58	10.36	-24953.27	15.15	-24905.40	19.49	-24853.22	28.14
	-25143.32	-0.78	-25095.15	3.86	-25048.13	7.35	-24999.45	12.50	-24949.53	18.89	-24906.77	18.12	-24857.25	24.12
	-25142.05	0.48	-25095.89	3.11	-25044.16	11.31	-24999.06	12.89	-24949.73	18.69	-24905.50	19.40	-24854.08	27.28
	-25141.97	0.56	-25097.29	1.71	-25048.14	7.34	-25001.33	10.62	-24950.21	18.21	-24902.96	21.94	-24860.39	20.98
	-25144.26	-1.72	-25095.37	3.64	-25047.42	8.06	-24999.23	12.72	-24951.09	17.33	-24904.69	20.21	-24855.48	25.89
	-25139.75	2.79	-25094.78	4.23	-25047.18	8.30	-25000.56	11.39	-24950.50	17.92	-24900.18	24.71	-24861.36	20.00
	-25143.26	-0.73	-25094.74	4.26	-25048.44	7.03	-25001.03	10.92	-24953.65	14.77	-24902.22	22.67	-24857.03	24.34
	-25142.77	-0.23	-25096.11	2.89	-25049.97	5.51	-24999.43	12.52	-24951.32	17.10	-24906.69	18.20	-24857.72	23.65
	-25143.93	-1.40	-25095.78	3.23	-25048.20	7.27	-24999.91	12.04	-24952.25	16.17	-24906.10	18.79	-24861.15	20.21
	-25141.36	1.17	-25095.57	3.43	-25052.49	2.98	-24996.87	15.08	-24949.94	18.48	-24902.39	22.50	-24855.58	25.79
	-25142.87	-0.34	-25095.31	3.70	-25047.52	7.95	-24999.92	12.03	-24948.37	20.05	-24897.17	27.73	-24854.28	27.08
	-25143.03	-0.49	-25095.71	3.29	-25045.65	9.83	-24994.40	17.55	-24952.75	15.67	-24902.55	22.34	-24859.09	22.28
	-25141.67	0.86	-25094.99	4.01	-25043.98	11.50	-25002.17	9.78	-24952.55	15.87	-24901.38	23.51	-24858.93	22.44
	-25142.49	0.04	-25096.49	2.52	-25048.61	6.87	-24999.78	12.17	-24950.40	18.02	-24904.25	20.64	-24858.56	22.81

-25143.80	-1.26	-25094.89	4.12	-25048.71	6.77	-24999.81	12.14	-24949.90	18.52	-24902.60	22.30	-24856.37	24.99
-25143.59	-1.06	-25095.45	3.56	-25048.61	6.87	-24999.20	12.75	-24954.67	13.75	-24903.93	20.96	-24859.68	21.68
-25142.74	-0.21	-25096.46	2.55	-25045.34	10.14	-24999.28	12.67	-24951.88	16.54	-24907.39	17.50	-24858.44	22.92
-25143.54	-1.00	-25094.48	4.53	-25047.44	8.03	-25000.91	11.04	-24955.17	13.25	-24905.95	18.94	-24861.41	19.96
-25142.17	0.36	-25093.47	5.54	-25046.07	9.40	-25001.11	10.84	-24953.20	15.22	-24906.14	18.75	-24859.51	21.86
-25144.74	-2.20	-25093.77	5.24	-25047.49	7.99	-25000.22	11.73	-24952.45	15.97	-24906.68	18.21	-24860.62	20.74
-25144.10	-1.56	-25092.96	6.05	-25044.73	10.75	-25001.67	10.28	-24952.56	15.86	-24903.58	21.31	-24861.41	19.95
-25143.39	-0.86	-25095.62	3.38	-25047.31	8.17	-24998.38	13.57	-24950.15	18.27	-24903.61	21.28	-24861.20	20.17
-25143.76	-1.23	-25095.07	3.94	-25047.30	8.18	-24998.87	13.08	-24951.32	17.10	-24907.34	17.55	-24859.42	21.94
-25142.76	-0.23	-25095.69	3.31	-25044.60	10.88	-24998.40	13.55	-24950.51	17.91	-24902.70	22.20	-24854.51	26.85
-25142.05	0.49	-25096.09	2.92	-25048.64	6.84	-25000.73	11.21	-24952.31	16.11	-24908.05	16.85	-24858.24	23.12
-25144.31	-1.78	-25094.67	4.34	-25047.97	7.51	-25002.41	9.54	-24952.58	15.84	-24902.74	22.15	-24858.06	23.30
-25142.85	-0.32	-25095.47	3.54	-25048.64	6.83	-25002.24	9.71	-24955.11	13.31	-24905.40	19.49	-24858.28	23.08
-25142.74	-0.21	-25096.01	3.00	-25049.03	6.44	-24998.46	13.49	-24950.00	18.43	-24905.87	19.02	-24860.04	21.33
-25142.02	0.51	-25095.39	3.62	-25049.86	5.62	-24999.92	12.02	-24957.06	11.36	-24905.42	19.48	-24857.01	24.35
-25143.36	-0.83	-25095.24	3.77	-25045.48	10.00	-25000.47	11.48	-24954.17	14.25	-24902.60	22.29	-24852.22	29.14
-25142.85	-0.32	-25092.72	6.29	-25046.83	8.65	-24999.86	12.09	-24954.79	13.63	-24907.20	17.70	-24860.17	21.20
-25142.05	0.48	-25094.01	5.00	-25046.98	8.50	-25000.64	11.31	-24951.61	16.81	-24903.37	21.52	-24859.20	22.17
-25143.01	-0.48	-25098.17	0.84	-25045.90	9.57	-25001.82	10.13	-24954.33	14.09	-24905.77	19.12	-24860.04	21.33
-25143.49	-0.96	-25094.34	4.66	-25047.47	8.01	-24999.15	12.80	-24955.82	12.60	-24895.13	29.77	-24852.08	29.29
-25142.07	0.47	-25094.50	4.50	-25045.66	9.81	-25000.25	11.70	-24951.03	17.39	-24905.79	19.10	-24856.66	24.70
-25143.24	-0.71	-25094.84	4.17	-25049.10	6.38	-24999.39	12.56	-24949.29	19.13	-24901.99	22.90	-24857.02	24.34
-25141.79	0.74	-25094.80	4.20	-25047.04	8.44	-24997.76	14.19	-24953.54	14.88	-24908.76	16.13	-24850.98	30.38
-25142.07	0.46	-25094.95	4.05	-25045.93	9.55	-25002.37	9.58	-24951.45	16.97	-24907.95	16.95	-24859.72	21.64
-25143.20	-0.67	-25094.56	4.44	-25047.50	7.98	-25001.82	10.13	-24949.98	18.44	-24905.44	19.45	-24856.22	25.15
-25143.58	-1.05	-25094.26	4.75	-25045.46	10.02	-25001.16	10.79	-24953.66	14.76	-24906.75	18.15	-24858.96	22.40
-25143.83	-1.29	-25095.33	3.67	-25050.19	5.28	-25000.69	11.26	-24954.09	14.34	-24904.73	20.17	-24861.50	19.87
-25142.55	-0.02	-25093.79	5.22	-25046.59	8.88	-24999.89	12.06	-24947.32	21.10	-24906.19	18.70	-24856.71	24.65
-25142.93	-0.40	-25092.81	6.20	-25048.38	7.09	-25001.77	10.18	-24952.56	15.86	-24898.54	26.35	-24856.69	24.68
-25141.28	1.25	-25096.07	2.93	-25048.14	7.33	-25003.38	8.57	-24954.01	14.41	-24905.31	19.59	-24862.42	18.94
-25142.85	-0.32	-25094.86	4.14	-25047.56	7.92	-24999.49	12.45	-24951.99	16.43	-24906.06	18.83	-24859.78	21.59
-25143.87	-1.33	-25095.06	3.95	-25048.08	7.40	-24997.91	14.04	-24953.81	14.61	-24903.68	21.21	-24857.52	23.84
-25143.27	-0.74	-25093.21	5.80	-25047.64	7.84	-24998.07	13.88	-24949.27	19.15	-24908.00	16.89	-24851.78	29.59
-25143.26	-0.72	-25095.32	3.69	-25049.52	5.96	-25000.68	11.26	-24951.82	16.60	-24904.99	19.90	-24859.13	22.24
-25143.33	-0.79	-25095.37	3.64	-25047.74	7.74	-24999.89	12.06	-24951.62	16.80	-24904.70	20.19	-24856.17	25.20
-25138.14	4.40	-25094.10	4.91	-25048.72	6.76	-25001.10	10.85	-24952.54	15.88	-24895.81	29.08	-24857.69	23.67
-25142.02	0.52	-25096.72	2.29	-25048.43	7.05	-25000.00	11.95	-24951.06	17.36	-24906.02	18.87	-24855.64	25.72
-25143.85	-1.32	-25096.15	2.86	-25044.98	10.50	-25001.14	10.81	-24954.83	13.59	-24906.32	18.58	-24855.33	26.03
-25143.92	-1.39	-25092.41	6.59	-25047.72	7.76	-25000.95	10.99	-24955.35	13.07	-24906.98	17.91	-24859.29	22.08
-25143.40	-0.87	-25096.56	2.45	-25047.11	8.37	-24997.48	14.46	-24955.05	13.37	-24905.75	19.15	-24859.70	21.66
-25143.32	-0.79	-25096.30	2.71	-25045.41	10.07	-25000.90	11.05	-24953.81	14.61	-24906.16	18.73	-24857.16	24.20

-25143.92	-1.39	-25094.27	4.74	-25047.49	7.99	-24998.86	13.09	-24950.48	17.94	-24904.57	20.32	-24858.03	23.34
-25142.38	0.15	-25095.95	3.06	-25048.17	7.31	-24997.38	14.57	-24950.75	17.67	-24905.83	19.06	-24860.98	20.39
-25143.29	-0.76	-25094.37	4.63	-25047.45	8.02	-25001.97	9.97	-24953.26	15.16	-24903.66	21.23	-24858.60	22.76
-25144.04	-1.51	-25094.37	4.64	-25048.01	7.46	-25000.68	11.27	-24952.53	15.89	-24905.11	19.79	-24860.58	20.79
-25143.76	-1.22	-25095.14	3.86	-25050.64	4.84	-25001.44	10.51	-24949.02	19.40	-24904.06	20.83	-24857.88	23.48
-25143.24	-0.71	-25095.23	3.78	-25044.45	11.03	-25001.93	10.02	-24951.39	17.03	-24903.64	21.25	-24861.64	19.72
-25143.15	-0.62	-25097.33	1.67	-25046.75	8.73	-24997.12	14.83	-24949.37	19.05	-24910.32	14.58	-24855.89	25.47
-25143.55	-1.02	-25092.81	6.19	-25046.46	9.02	-25002.11	9.84	-24953.87	14.55	-24898.06	26.83	-24859.70	21.66
-25142.99	-0.46	-25092.15	6.86	-25046.65	8.83	-24999.85	12.10	-24952.38	16.04	-24908.87	16.02	-24862.27	19.09
-25143.11	-0.58	-25095.01	4.00	-25045.57	9.90	-25000.38	11.57	-24952.22	16.20	-24905.06	19.83	-24854.56	26.81
-25142.85	-0.31	-25094.44	4.57	-25049.02	6.45	-24997.41	14.54	-24949.60	18.82	-24897.54	27.35	-24859.85	21.51
-25143.39	-0.86	-25095.84	3.16	-25047.53	7.95	-25000.57	11.38	-24951.12	17.31	-24904.11	20.78	-24856.33	25.03
-25142.85	-0.32	-25095.52	3.49	-25046.79	8.69	-24998.53	13.42	-24955.90	12.52	-24907.28	17.61	-24859.35	22.01
-25144.93	-2.40	-25096.15	2.85	-25049.66	5.82	-24953.21	58.74	-24949.12	19.30	-24903.17	21.72	-24861.92	19.44
-25143.26	-0.73	-25094.62	4.39	-25049.19	6.29	-25000.83	11.12	-24951.11	17.31	-24905.15	19.74	-24860.19	21.17
-25143.52	-0.99	-25094.31	4.69	-25044.95	10.52	-25000.45	11.50	-24949.75	18.67	-24905.42	19.47	-24859.32	22.04
-25142.34	0.19	-25095.22	3.79	-25047.93	7.55	-25002.20	9.74	-24951.54	16.88	-24905.04	19.86	-24860.06	21.31
-25143.57	-1.03	-25095.54	3.46	-25047.48	8.00	-24996.15	15.80	-24904.57	63.85	-24903.35	21.54	-24857.73	23.64
-25141.76	0.77	-25045.24	53.76	-25047.70	7.78	-25002.26	9.69	-24953.70	14.73	-24906.96	17.93	-24855.34	26.02
-25094.05	48.49	-25095.97	3.04	-24999.15	56.33	-25001.19	10.76	-24953.37	15.05	-24855.58	69.31	-24857.45	23.91
-25142.50	0.03	-25095.14	3.87	-25047.20	8.27	-25000.21	11.74	-24954.97	13.45	-24904.62	20.27	-24855.13	26.23
-25144.47	-1.93	-25094.32	4.69	-25049.30	6.18	-25001.17	10.78	-24952.27	16.16	-24907.29	17.60	-24851.21	30.15
-25141.66	0.88	-25094.91	4.09	-25047.91	7.57	-24999.47	12.48	-24951.69	16.73	-24906.19	18.71	-24860.22	21.15
-25143.08	-0.55	-25095.85	3.16	-25047.34	8.13	-24998.16	13.79	-24952.65	15.77	-24899.07	25.82	-24859.93	21.43
-25141.72	0.81	-25095.50	3.51	-25046.36	9.12	-24998.94	13.01	-24949.38	19.04	-24904.41	20.48	-24861.55	19.81
-25141.81	0.72	-25095.92	3.08	-25042.51	12.97	-25002.17	9.78	-24950.51	17.91	-24901.66	23.24	-24855.15	26.21
-25143.92	-1.39	-25097.03	1.97	-25047.79	7.69	-25000.37	11.58	-24953.80	14.62	-24907.22	17.67	-24855.72	25.64
-25141.68	0.85	-25095.63	3.38	-25048.07	7.41	-24998.63	13.32	-24953.97	14.46	-24855.10	69.79	-24857.93	23.44
-25141.67	0.86	-25093.77	5.24	-25048.75	6.73	-24998.54	13.41	-24950.88	17.54	-24901.55	23.34	-24858.24	23.13
-25142.84	-0.31	-25096.88	2.12	-25047.14	8.34	-25000.33	11.62	-24952.98	15.44	-24906.86	18.03	-24859.47	21.89
-25142.57	-0.04	-25095.10	3.90	-25048.86	6.62	-25000.59	11.36	-24956.23	12.20	-24904.76	20.13	-24859.52	21.84
-25143.45	-0.92	-25093.87	5.14	-25047.44	8.04	-25003.69	8.26	-24950.83	17.59	-24907.26	17.63	-24859.73	21.64
-25142.50	0.03	-25097.02	1.99	-25048.21	7.26	-24998.98	12.97	-24949.77	18.65	-24903.44	21.45	-24858.80	22.57
-25142.87	-0.34	-25097.70	1.30	-25045.97	9.51	-24999.92	12.03	-24953.30	15.12	-24907.69	17.20	-24856.51	24.86
-25143.10	-0.56	-25092.74	6.27	-25049.93	5.54	-24998.86	13.09	-24953.46	14.96	-24904.39	20.50	-24861.72	19.64
-25143.61	-1.08	-25097.46	1.54	-25047.42	8.06	-24999.86	12.09	-24948.33	20.09	-24908.76	16.13	-24854.99	26.37
-25142.94	-0.40	-25095.68	3.33	-25044.87	10.61	-25002.57	9.38	-24955.48	12.94	-24905.52	19.38	-24859.33	22.03
-25142.44	0.10	-25096.95	2.06	-25049.14	6.34	-25000.85	11.10	-24951.53	16.89	-24902.93	21.97	-24858.05	23.32
-25143.31	-0.78	-25093.85	5.15	-25048.89	6.59	-24995.86	16.09	-24954.67	13.75	-24906.16	18.73	-24860.07	21.29
-25142.65	-0.12	-25096.06	2.94	-25049.01	6.47	-24999.99	11.96	-24954.31	14.11	-24904.80	20.09	-24861.65	19.71
-25142.27	0.26	-25094.79	4.21	-25047.47	8.01	-25001.47	10.47	-24954.59	13.83	-24906.28	18.62	-24857.27	24.09

	-25143.18	-0.65	-25095.51	3.50	-25045.87	9.61	-24997.22	14.72	-24949.24	19.18	-24905.47	19.42	-24857.68	23.69
	-25143.55	-1.01	-25096.42	2.59	-25047.08	8.40	-25000.87	11.08	-24908.05	60.37	-24905.36	19.53	-24858.71	22.66
	-25144.19	-1.66	-25095.75	3.25	-25047.66	7.82	-25000.70	11.24	-24953.10	15.32	-24905.77	19.12	-24856.77	24.59
	-25143.44	-0.90	-25095.90	3.11	-25047.16	8.32	-25000.33	11.62	-24950.63	17.79	-24859.27	65.62	-24863.13	18.23
	-25142.88	-0.35	-25096.43	2.58	-25047.28	8.19	-25000.89	11.06	-24951.59	16.83	-24903.91	20.99	-24862.37	18.99
	-25143.28	-0.75	-25095.51	3.50	-25047.13	8.35	-25002.79	9.16	-24951.18	17.24	-24904.24	20.65	-24857.09	24.27
	-25143.91	-1.37	-25093.90	5.10	-25044.43	11.04	-25001.04	10.91	-24947.57	20.85	-24900.42	24.48		
	-25143.77	-1.24	-25096.07	2.93	-25045.46	10.01	-24998.30	13.65	-24954.43	13.99	-24907.10	17.79		
	-25143.10	-0.57	-25095.93	3.07	-25048.09	7.39	-25001.02	10.92	-24955.46	12.96	-24903.58	21.31		
	-25141.47	1.06	-25094.80	4.21	-25047.27	8.20	-25003.33	8.61	-24950.54	17.88	-24858.05	66.85		
	-25143.64	-1.11	-25095.00	4.00	-25050.55	4.93			-24950.35	18.07	-24907.67	17.23		
	-25143.63	-1.10	-25092.35	6.66	-25047.24	8.24			-24944.18	24.24	-24902.16	22.74		
	-25142.49	0.04	-25095.43	3.58	-25047.25	8.23			-24952.96	15.46	-24908.26	16.63		
	-25143.61	-1.07	-25094.38	4.62	-25047.63	7.85					-24903.76	21.14		
	-25143.07	-0.54	-25097.17	1.84	-25048.62	6.86					-24905.00	19.90		
	-25143.52	-0.99	-25095.18	3.83							-24904.20	20.69		
	-25143.85	-1.31	-25094.76	4.25										
	-25142.51	0.03												
Average energies	-25142.56	-0.02	-25094.78	4.22	-25047.04	8.44	-24999.66	12.29	-24951.33	17.09	-24903.13	21.76	-24858.07	23.29

U

	ground state energy	solid solution energy												
concentration in solid solution (%)	3		6		9		12		15		18		21	
number of atoms	2		4		6		8		10		12		14	
	-25147.30	0.26	-25104.69	4.36	-25063.26	7.27	-25013.62	18.40	-24975.52	18.00	-24933.84	21.16	-24894.19	22.30
	-25147.56	-0.01	-25104.77	4.27	-25061.27	9.26	-25019.95	12.07	-24977.12	16.39	-24938.22	16.78	-24892.31	24.19
	-25148.25	-0.70	-25106.84	2.20	-25062.90	7.63	-25019.66	12.37	-24978.39	15.12	-24936.01	18.99	-24891.54	24.95
	-25147.50	0.05	-25104.91	4.13	-25063.76	6.77	-25022.43	9.59	-24976.67	16.84	-24930.46	24.54	-24893.31	23.19
	-25147.53	0.02	-25097.34	11.70	-25063.61	6.92	-25021.35	10.67	-24979.84	13.67	-24935.96	19.04	-24892.01	24.49
	-25146.44	1.11	-25104.41	4.63	-25060.52	10.01	-25022.38	9.64	-24975.96	17.55	-24938.40	16.60	-24892.55	23.94
	-25147.10	0.45	-25106.40	2.64	-25058.48	12.05	-25017.53	14.49	-24979.47	14.05	-24936.82	18.18	-24895.44	21.06
	-25145.84	1.72	-25106.87	2.18	-25060.95	9.58	-25015.44	16.58	-24981.22	12.29	-24934.74	20.26	-24895.68	20.81
	-25147.37	0.19	-25103.94	5.10	-25061.30	9.23	-25021.84	10.18	-24977.92	15.60	-24937.40	17.61	-24892.41	24.08
	-25147.58	-0.03	-25105.68	3.36	-25060.47	10.06	-25017.86	14.16	-24977.76	15.75	-24938.57	16.43	-24894.65	21.85
	-25147.70	-0.15	-25103.04	6.01	-25061.95	8.58	-25017.46	14.57	-24978.21	15.30	-24936.28	18.73	-24896.13	20.37
	-25148.92	-1.37	-25107.05	2.00	-25062.22	8.31	-25023.28	8.74	-24980.79	12.73	-24936.74	18.27	-24895.41	21.09
	-25147.21	0.35	-25105.74	3.30	-25062.83	7.70	-25023.34	8.68	-24978.98	14.54	-24937.57	17.44	-24891.75	24.74
	-25148.50	-0.95	-25107.07	1.97	-25062.33	8.20	-25019.86	12.16	-24977.52	15.99	-24935.28	19.72	-24890.99	25.51

-25148.71	-1.16	-25104.57	4.47	-25061.55	8.99	-25018.09	13.94	-24981.44	12.07	-24935.10	19.91	-24895.14	21.36
-25147.69	-0.14	-25104.94	4.11	-25063.35	7.18	-25015.25	16.77	-24979.16	14.35	-24935.91	19.09	-24899.44	17.06
-25148.24	-0.69	-25105.90	3.14	-25060.93	9.61	-25018.76	13.27	-24981.12	12.39	-24938.95	16.05	-24891.34	25.16
-25147.30	0.25	-25098.81	10.24	-25062.84	7.69	-25020.23	11.79	-24973.03	20.49	-24936.94	18.06	-24893.00	23.49
-25148.01	-0.46	-25100.18	8.86	-25059.13	11.40	-25015.98	16.04	-24976.95	16.56	-24936.66	18.34	-24896.19	20.31
-25148.39	-0.84	-25106.18	2.86	-25060.65	9.88	-25019.41	12.61	-24978.02	15.49	-24936.82	18.18	-24886.41	30.09
-25147.03	0.53	-25105.05	3.99	-25061.47	9.06	-25020.39	11.63	-24977.95	15.57	-24937.85	17.15	-24894.55	21.95
-25147.00	0.55	-25104.75	4.29	-25061.34	9.20	-25021.27	10.75	-24979.11	14.40	-24938.68	16.32	-24892.59	23.91
-25140.88	6.67	-25103.20	5.85	-25063.19	7.35	-25020.52	11.50	-24981.15	12.36	-24939.09	15.92	-24894.50	22.00
-25148.64	-1.09	-25102.78	6.26	-25061.83	8.70	-25018.87	13.15	-24973.06	20.46	-24932.99	22.02	-24893.12	23.37
-25146.66	0.89	-25105.59	3.45	-25062.74	7.79	-25021.31	10.72	-24976.59	16.92	-24931.36	23.64	-24889.68	26.81
-25147.29	0.26	-25105.51	3.53	-25062.79	7.74	-25020.61	11.42	-24976.23	17.28	-24935.49	19.52	-24893.20	23.29
-25147.81	-0.25	-25105.95	3.09	-25061.39	9.14	-25022.77	9.25	-24978.25	15.27	-24936.72	18.28	-24894.18	22.31
-25147.67	-0.12	-25104.00	5.05	-25065.19	5.34	-25021.43	10.59	-24978.67	14.84	-24934.70	20.30	-24898.07	18.43
-25139.74	7.81	-25102.77	6.27	-25062.14	8.40	-25022.32	9.70	-24978.98	14.54	-24932.73	22.28	-24895.12	21.38
-25147.27	0.28	-25104.87	4.18	-25062.78	7.75	-25017.63	14.39	-24978.89	14.63	-24937.32	17.68	-24892.78	23.71
-25140.98	6.58	-25104.35	4.69	-25064.97	5.57	-25017.63	14.39	-24978.85	14.66	-24936.71	18.29	-24893.19	23.31
-25140.43	7.12	-25103.70	5.34	-25061.89	8.65	-25019.96	12.07	-24978.67	14.84	-24937.01	17.99	-24894.50	21.99
-25148.37	-0.82	-25106.08	2.96	-25061.89	8.65	-25014.27	17.75	-24978.54	14.97	-24937.51	17.50	-24885.88	30.61
-25140.83	6.72	-25107.31	1.74	-25063.07	7.46	-25016.49	15.53	-24980.71	12.81	-24936.16	18.84	-24895.62	20.88
-25145.72	1.83	-25105.00	4.04	-25062.94	7.59	-25018.50	13.53	-24979.79	13.72	-24936.30	18.70	-24892.37	24.12
-25147.36	0.19	-25098.66	10.38	-25061.76	8.77	-25019.39	12.63	-24974.94	18.57	-24937.13	17.88	-24892.21	24.29
-25147.86	-0.31	-25105.08	3.97	-25057.70	12.84	-25017.12	14.91	-24979.95	13.56	-24933.88	21.12	-24894.22	22.27
-25148.53	-0.97	-25105.03	4.01	-25063.67	6.87	-25020.02	12.01	-24980.39	13.12	-24933.93	21.07	-24893.07	23.42
-25148.41	-0.86	-25105.63	3.41	-25064.34	6.19	-25020.66	11.36	-24980.02	13.49	-24932.02	22.98	-24897.64	18.85
-25146.40	1.15	-25102.98	6.07	-25065.26	5.27	-25018.00	14.03	-24977.11	16.40	-24933.87	21.13	-24891.54	24.96
-25146.76	0.80	-25106.08	2.96	-25061.73	8.80	-25015.67	16.35	-24979.50	14.01	-24935.40	19.60	-24892.13	24.37
-25146.37	1.18	-25104.14	4.90	-25061.76	8.77	-25020.45	11.57	-24979.72	13.80	-24938.16	16.84	-24890.75	25.75
-25146.87	0.68	-25101.29	7.75	-25063.56	6.97	-25019.90	12.13	-24978.96	14.55	-24934.52	20.49	-24894.82	21.68
-25145.81	1.74	-25104.12	4.92	-25062.45	8.09	-25021.12	10.90	-24976.98	16.53	-24936.99	18.01	-24895.87	20.63
-25148.65	-1.10	-25102.99	6.06	-25056.80	13.74	-25020.95	11.07	-24978.15	15.37	-24932.55	22.46	-24894.37	22.12
-25147.52	0.03	-25103.87	5.18	-25063.52	7.02	-25020.01	12.01	-24981.14	12.38	-24936.55	18.45	-24893.92	22.58
-25148.20	-0.64	-25105.99	3.05	-25062.26	8.27	-25022.15	9.87	-24974.76	18.75	-24934.51	20.50	-24896.39	20.10
-25146.70	0.86	-25105.37	3.68	-25063.02	7.52	-25018.33	13.69	-24978.41	15.11	-24935.68	19.33	-24896.06	20.44
-25148.84	-1.29	-25103.86	5.18	-25063.81	6.72	-25019.27	12.75	-24974.81	18.70	-24934.16	20.85	-24895.96	20.54
-25148.86	-1.31	-25104.16	4.88	-25061.96	8.57	-25023.92	8.11	-24980.84	12.68	-24936.52	18.48	-24890.60	25.90
-25145.94	1.61	-25103.33	5.71	-25062.36	8.18	-25018.55	13.48	-24978.17	15.34	-24933.85	21.16	-24893.52	22.97
-25146.65	0.90	-25106.62	2.42	-25061.59	8.95	-25022.44	9.58	-24976.09	17.43	-24936.45	18.55	-24892.57	23.92
-25148.93	-1.38	-25102.85	6.19	-25065.43	5.11	-25018.60	13.42	-24979.54	13.98	-24931.46	23.54	-24894.41	22.08
-25148.96	-1.41	-25103.04	6.01	-25059.38	11.15	-25021.32	10.70	-24981.02	12.49	-24934.26	20.74	-24893.42	23.07
-25146.89	0.66	-25106.08	2.96	-25060.11	10.42	-25020.73	11.29	-24977.30	16.21	-24931.50	23.51	-24895.09	21.40

	-25147.34	0.21	-25104.98	4.06	-25061.85	8.68	-25018.77	13.25	-24980.24	13.28	-24934.30	20.71	-24895.70	20.80
	-25147.69	-0.14	-25098.93	10.11	-25062.34	8.20	-25013.61	18.42	-24979.00	14.52	-24937.77	17.24	-24892.69	23.81
	-25147.57	-0.02	-25105.30	3.74	-25060.93	9.60	-25024.02	8.01	-24980.14	13.37	-24933.41	21.59	-24894.99	21.50
	-25147.17	0.39	-25104.89	4.16	-25060.84	9.69	-25020.36	11.66	-24976.31	17.20	-24932.73	22.28	-24897.03	19.46
	-25148.28	-0.73	-25106.49	2.56	-25062.32	8.21	-25024.34	7.68	-24976.84	16.68	-24926.52	28.48	-24889.71	26.79
	-25147.80	-0.25	-25104.61	4.43	-25062.24	8.29	-25021.40	10.63			-24936.12	18.89	-24894.88	21.61
	-25149.12	-1.57	-25102.71	6.33	-25064.14	6.40	-25022.71	9.31			-24933.63	21.37	-24892.71	23.78
	-25148.12	-0.57	-25105.56	3.49	-25063.07	7.46					-24939.17	15.84	-24895.21	21.28
	-25146.32	1.23	-25103.11	5.93	-25060.23	10.31					-24936.79	18.21	-24897.35	19.14
	-25148.42	-0.87	-25105.28	3.76	-25061.53	9.01					-24937.12	17.88	-24895.01	21.48
	-25146.94	0.61	-25105.34	3.70	-25065.90	4.63					-24933.22	21.79	-24887.40	29.10
	-25147.31	0.25	-25104.93	4.11	-25061.13	9.40					-24934.09	20.91	-24895.42	21.08
	-25147.80	-0.25	-25105.14	3.90	-25062.87	7.66								
	-25147.37	0.18	-25105.26	3.78	-25062.85	7.69								
	-25149.11	-1.55	-25105.75	3.29	-25064.58	5.96								
	-25148.02	-0.47	-25106.44	2.61	-25062.61	7.92								
	-25148.42	-0.86	-25105.32	3.72	-25062.31	8.23								
	-25148.12	-0.57	-25106.78	2.26	-25062.51	8.03								
	-25148.67	-1.12	-25106.73	2.31	-25061.19	9.35								
	-25148.61	-1.05	-25104.90	4.15	-25063.12	7.41								
	-25148.11	-0.56	-25105.83	3.21	-25060.01	10.52								
	-25147.84	-0.29	-25105.44	3.61	-25060.45	10.08								
	-25146.65	0.90	-25105.40	3.64	-25063.84	6.69								
	-25146.56	1.00	-25104.54	4.50	-25060.97	9.56								
	-25148.85	-1.30	-25104.99	4.05										
	-25148.42	-0.87	-25107.09	1.95										
	-25147.76	-0.21	-25104.49	4.56										
	-25148.03	-0.48	-25105.51	3.53										
			-25094.51	14.53										
			-25105.45	3.59										
Average energies	-25147.23	0.30	-25104.54	4.50	-25062.19	8.34	-25019.88	12.15	-24978.45	15.07	-24935.54	19.47	-24893.75	22.75

$Ca_{1-x}M_xZrTi_{2-2x}Fe_{2x}O_7$ M=(Ce, Pu, Th, U)

Ti(1)Ti(2)

N.B. All stated energies are in eV

Ce

	ground state energy	solid solution energy												
concentration in solid solution (%)	3		6		9		12		15		18		21	
number of atoms	2		4		6		8		10		12		13	
	-25146.04	-3.15	-25100.84	-1.12	-25058.60	-2.06	-25005.60	7.77	-24961.17	9.03	-24910.30	16.72	-24896.11	9.33
	-25146.60	-3.71	-25100.18	-0.46	-25056.59	-0.05	-25009.25	4.12	-24967.82	2.37	-24923.17	3.86	-24898.22	7.22
	-25146.91	-4.02	-25101.17	-1.46	-25057.28	-0.74	-25010.56	2.81	-24968.60	1.60	-24921.31	5.71	-24902.94	2.50
	-25143.93	-1.04	-25102.47	-2.75	-25057.53	-0.99	-25012.33	1.04	-24967.62	2.57	-24919.34	7.69	-24899.80	5.64
	-25147.79	-4.90	-25099.87	-0.15	-25056.20	0.34	-25014.25	-0.88	-24967.52	2.68	-24925.96	1.06	-24899.54	5.90
	-25145.64	-2.75	-25098.81	0.90	-25057.00	-0.45	-25009.72	3.65	-25007.88	-37.69	-24920.15	6.87	-24898.41	7.02
	-25147.53	-4.64	-25101.28	-1.56	-25056.28	0.26	-25009.62	3.75	-24963.77	6.43	-24915.10	11.92	-24898.45	6.99
	-25146.72	-3.83	-25101.71	-1.99	-25053.99	2.55	-25009.19	4.18	-24964.95	5.25	-24921.30	5.72	-24895.23	10.21
	-25145.89	-3.01	-25099.23	0.49	-25053.60	2.94	-25013.34	0.03	-24962.13	8.07	-24922.00	5.02	-24897.65	7.79
	-25147.40	-4.51	-25099.23	0.48	-25059.03	-2.49	-25011.26	2.11	-24960.62	9.57	-24920.80	6.22	-24898.76	6.67
	-25146.74	-3.86	-25099.76	-0.04	-25054.43	2.11	-25011.33	2.04	-24963.16	7.04	-24921.59	5.43	-24899.85	5.58
	-25145.42	-2.54	-25099.64	0.08	-25054.96	1.58	-25009.81	3.56	-25006.12	-35.93	-24920.69	6.34	-24900.97	4.47
	-25145.76	-2.87	-25103.14	-3.43	-25056.81	-0.27	-25011.82	1.55	-24966.86	3.34	-24923.48	3.54	-24900.06	5.37
	-25146.94	-4.06	-25099.78	-0.07	-25055.31	1.23	-25011.13	2.24	-24969.79	0.41	-24920.67	6.35	-24895.82	9.62
	-25147.15	-4.26	-25098.93	0.78	-25055.71	0.84	-25010.30	3.07	-24962.74	7.45	-24925.86	1.16	-24897.87	7.57
	-25146.51	-3.62	-25101.58	-1.86	-25055.53	1.01	-25012.13	1.24	-24968.94	1.26	-24919.16	7.87	-24893.13	12.30
	-25145.91	-3.02	-25100.53	-0.82	-25052.84	3.70	-25011.78	1.58	-24965.06	5.13	-24918.45	8.57	-24895.15	10.28
	-25146.75	-3.86	-25100.13	-0.41	-25052.20	4.34	-25005.05	8.32	-24959.22	10.98	-24918.91	8.12	-24899.21	6.22
	-25145.88	-2.99	-25101.27	-1.56	-25052.54	4.00	-25009.04	4.33	-24965.37	4.82	-24920.32	6.70	-24890.07	15.36
	-25146.50	-3.61	-25101.81	-2.09	-25055.90	0.65	-25013.03	0.34	-24969.31	0.88	-24922.87	4.15	-24900.43	5.00
	-25147.29	-4.40	-25100.41	-0.70	-25055.24	1.30	-25010.87	2.50	-24964.55	5.65	-24922.78	4.24	-24899.47	5.96
	-25147.75	-4.86	-25101.17	-1.46	-25053.11	3.44	-25012.20	1.17	-24967.57	2.63	-24923.30	3.73	-24901.37	4.07
	-25146.92	-4.03	-25101.46	-1.75	-25054.89	1.65	-25011.56	1.81	-24958.38	11.82	-24919.79	7.24	-24900.69	4.75
	-25146.80	-3.91	-25101.93	-2.21	-25057.11	-0.56	-25007.68	5.68	-24964.40	5.80	-24921.71	5.32	-24890.89	14.55
	-25147.19	-4.30	-25100.58	-0.86	-25056.31	0.23	-25008.18	5.19	-24965.18	5.02	-24917.89	9.14	-24894.10	11.33
	-25145.35	-2.47	-25100.35	-0.64	-25055.82	0.73	-25011.70	1.67	-24965.30	4.90	-24922.96	4.06	-24893.04	12.39
	-25146.94	-4.05	-25102.23	-2.51	-25056.30	0.24	-25006.59	6.78	-24960.24	9.95	-24924.04	2.98	-24896.22	9.22
	-25146.39	-3.50	-25103.04	-3.32	-25053.27	3.27	-25008.39	4.98	-24967.29	2.90	-24923.03	3.99	-24896.81	8.63
	-25146.80	-3.91	-25100.80	-1.08	-25056.88	-0.34	-25014.71	-1.35	-24966.77	3.43	-24917.70	9.33	-24894.54	10.89

-25144.88	-1.99	-25102.17	-2.46	-25056.21	0.33	-25011.92	1.45	-24969.90	0.30	-24921.20	5.83	-24898.48	6.96
-25145.69	-2.80	-25099.74	-0.03	-25057.07	-0.53	-25008.32	5.05	-24965.79	4.40	-24918.72	8.30	-24902.15	3.28
-25148.25	-5.36	-25098.46	1.25	-25056.16	0.39	-25013.00	0.37	-24966.09	4.11	-24918.75	8.27	-24899.77	5.67
-25146.41	-3.52	-25102.09	-2.38	-25057.76	-1.22	-25011.82	1.54	-24968.96	1.24	-24910.40	16.63	-24898.88	6.56
-25146.94	-4.05	-25100.51	-0.79	-25055.74	0.80	-25010.24	3.13	-24968.65	1.54	-24923.41	3.61	-24891.31	14.13
-25146.18	-3.29	-25102.24	-2.53	-25056.83	-0.29	-25012.23	1.14	-24966.59	3.60	-24916.80	10.23	-24896.41	9.03
-25146.31	-3.43	-25101.28	-1.56	-25054.38	2.16	-25011.72	1.65	-24963.85	6.35	-24917.45	9.57	-24901.69	3.75
-25147.03	-4.14	-25100.75	-1.04	-25056.99	-0.44	-25010.00	3.37	-24965.32	4.88	-24920.25	6.77	-24897.88	7.56
-25146.73	-3.84	-25100.97	-1.26	-25057.81	-1.27	-25013.25	0.12	-24967.11	3.09	-24918.56	8.46	-24903.22	2.22
-25146.19	-3.30	-25102.80	-3.08	-25055.24	1.30	-25011.77	1.60	-24967.51	2.68	-24920.13	6.89	-24895.84	9.60
-25146.03	-3.14	-25101.76	-2.04	-25055.76	0.78	-25013.19	0.18	-24967.24	2.95	-24921.49	5.53	-24901.28	4.16
-25146.56	-3.67	-25101.70	-1.98	-25056.21	0.33	-25012.94	0.42	-24966.55	3.64	-24922.94	4.08	-24896.11	9.32
-25145.35	-2.46	-25100.51	-0.79	-25056.73	-0.19	-25009.34	4.03	-24967.06	3.13	-24923.09	3.93	-24896.83	8.61
-25147.25	-4.36	-25101.76	-2.05	-25056.85	-0.31	-25012.63	0.74	-24967.81	2.39	-24921.15	5.87	-24892.99	12.45
-25146.17	-3.28	-25102.03	-2.32	-25056.81	-0.27	-25010.50	2.87	-24968.66	1.54	-24922.29	4.73	-24899.87	5.56
-25146.86	-3.97	-25103.50	-3.78	-25029.09	27.45	-25011.32	2.05	-24966.23	3.96	-24916.94	10.08	-24893.82	11.61
-25146.28	-3.39	-25101.06	-1.35	-25058.56	-2.02	-25011.87	1.50	-24967.28	2.92	-24923.92	3.11	-24898.77	6.67
-25145.86	-2.97	-25101.28	-1.56	-25052.47	4.07	-25012.40	0.97	-24967.93	2.27	-24918.76	8.26	-24896.21	9.22
-25145.98	-3.09	-25101.46	-1.74	-25055.07	1.47	-25013.28	0.09	-24968.66	1.53	-24923.46	3.56	-24899.72	5.72
-25145.49	-2.60	-25099.77	-0.06	-25055.37	1.18	-25013.87	-0.50	-24968.97	1.23	-24920.09	6.93	-24903.66	1.78
-25145.68	-2.79	-25101.65	-1.94	-25056.64	-0.09	-25010.45	2.92	-24964.70	5.49	-24923.03	3.99	-24897.42	8.02
-25145.99	-3.11	-25101.31	-1.59	-25056.48	0.07	-25014.19	-0.82	-24967.07	3.13	-24925.97	1.05	-24901.14	4.30
-25146.19	-3.30	-25101.76	-2.04	-25056.85	-0.31	-25004.21	9.16	-24961.18	9.02	-24917.71	9.32	-24896.91	8.52
-25146.02	-3.13	-25102.06	-2.35	-25055.25	1.29	-25012.07	1.30	-24967.48	2.71	-24921.56	5.46	-24897.37	8.06
-25146.22	-3.33	-25100.98	-1.26	-25056.01	0.53	-25013.37	0.00	-24959.51	10.68	-24921.29	5.73	-24898.68	6.75
-25146.93	-4.04	-25098.31	1.40	-25058.04	-1.49	-25005.64	7.73	-24966.18	4.02	-24921.83	5.19	-24900.22	5.21
-25145.77	-2.88	-25100.66	-0.95	-25056.29	0.25	-25009.71	3.66	-24964.92	5.27	-24921.03	6.00	-24895.95	9.49
-25146.49	-3.60	-25102.12	-2.40	-25057.11	-0.57	-25011.69	1.68	-24966.64	3.55	-24918.85	8.18	-24897.34	8.09
-25144.21	-1.32	-25101.13	-1.41	-25057.63	-1.08	-25012.40	0.96	-24964.31	5.88	-24922.06	4.96	-24900.16	5.28
-25146.06	-3.17	-25098.50	1.22	-25057.32	-0.78	-25012.10	1.27	-24960.77	9.43	-24924.34	2.69	-24900.91	4.53
-25146.64	-3.75	-25100.57	-0.85	-25054.33	2.22	-25007.14	6.23	-24964.73	5.46	-24920.45	6.57	-24898.44	6.99
-25146.87	-3.98	-25100.87	-1.16	-25047.29	9.25	-25010.01	3.36	-24965.68	4.52	-24917.64	9.38	-24896.25	9.18
-25145.98	-3.09	-25100.31	-0.59	-25057.81	-1.26	-25010.41	2.96	-24968.42	1.78	-24924.34	2.69	-24899.78	5.66
-25146.74	-3.85	-25102.92	-3.20	-25057.15	-0.60	-25012.22	1.15	-24966.10	4.10	-24919.21	7.81	-24895.34	10.09
-25147.59	-4.70	-25100.70	-0.98	-25051.63	4.91	-25009.98	3.39	-24965.82	4.37	-24922.29	4.73	-24898.15	7.29
-25146.17	-3.29	-25099.86	-0.15	-25055.41	1.14	-25011.18	2.19	-24968.34	1.86	-24924.31	2.71	-24901.15	4.28
-25147.18	-4.29	-25100.83	-1.11	-25055.29	1.25	-25011.93	1.44	-24966.60	3.60	-24920.15	6.88	-24899.36	6.07
-25146.81	-3.92	-25099.97	-0.26	-25059.03	-2.49	-25010.92	2.45	-24966.50	3.69	-24921.00	6.02	-24897.84	7.60
-25143.45	-0.56	-25101.47	-1.75	-25056.03	0.51	-25014.00	-0.63	-24918.11	52.09	-24914.57	12.45	-24902.29	3.14
-25143.94	-1.05	-25102.52	-2.81	-25056.22	0.32	-24964.18	49.18	-24965.52	4.68	-24898.41	28.61	-24900.81	4.63
-25145.07	-2.18	-25101.48	-1.76	-25057.22	-0.68	-25012.55	0.82	-24966.92	3.28	-24925.63	1.39	-24893.26	12.17

-25146.35	-3.46	-25100.68	-0.96	-25057.06	-0.52	-25012.01	1.36	-24970.78	-0.58	-24921.34	5.68	-24902.48	2.95
-25146.44	-3.55	-25102.06	-2.35	-25056.15	0.39	-25013.43	-0.06	-24967.86	2.33	-24920.46	6.56	-24898.82	6.61
-25147.25	-4.36	-25100.98	-1.26	-25049.29	7.25	-25007.51	5.85	-24963.78	6.41	-24920.05	6.97	-24895.37	10.07
-25146.24	-3.35	-25101.51	-1.79	-25053.69	2.85	-25009.83	3.54	-24959.99	10.20	-24919.58	7.45	-24903.97	1.46
-25147.35	-4.46	-25101.15	-1.44	-25056.53	0.01	-25013.27	0.09	-24967.25	2.95	-24924.04	2.98	-24899.55	5.89
-25146.74	-3.86	-25097.41	2.30	-25056.22	0.32	-25010.45	2.92	-24966.03	4.17	-24917.91	9.11	-24902.87	2.57
-25145.47	-2.59	-25098.91	0.81	-25057.49	-0.95	-25014.59	-1.22	-24963.34	6.86	-24917.39	9.64	-24900.95	4.49
-25148.20	-5.31	-25102.34	-2.63	-25058.24	-1.70	-25008.87	4.49	-24966.10	4.10	-24924.30	2.72	-24896.27	9.17
-25147.36	-4.47	-25100.25	-0.54	-25055.32	1.22	-25008.79	4.58	-24967.77	2.43	-24917.95	9.07	-24896.80	8.63
-25144.83	-1.94	-25100.02	-0.30	-25053.60	2.94	-25011.47	1.89	-24968.30	1.90	-24916.43	10.60	-24904.22	1.22
-25146.58	-3.69	-25100.15	-0.44	-25054.51	2.03	-25010.52	2.85	-24967.72	2.48	-24923.01	4.01	-24894.75	10.69
-25146.62	-3.73	-25101.89	-2.17	-25057.31	-0.77	-25012.94	0.43	-24966.42	3.77	-24902.83	24.20	-24896.17	9.27
-25146.57	-3.68	-25102.62	-2.90	-25054.68	1.86	-25008.81	4.56	-24959.79	10.41	-24918.64	8.38	-24899.85	5.59
-25146.11	-3.22	-25099.98	-0.27	-25056.32	0.22	-25009.23	4.14	-24965.14	5.06	-24916.23	10.80	-24897.45	7.98
-25145.22	-2.33	-25101.48	-1.77	-25056.98	-0.43	-25009.74	3.63	-24966.54	3.66	-24923.55	3.47	-24901.89	3.55
-25146.56	-3.67	-25098.07	1.64	-25057.46	-0.92	-25004.39	8.98	-24965.87	4.33	-24922.07	4.95	-24904.73	0.71
-25146.94	-4.05	-25099.73	-0.01	-25056.49	0.06	-25014.06	-0.69	-24963.88	6.31	-24923.47	3.55	-24901.15	4.29
-25145.80	-2.91	-25100.84	-1.12	-25053.29	3.25	-25010.82	2.55	-24965.26	4.93	-24917.41	9.61	-24899.73	5.71
-25145.07	-2.18	-25099.55	0.17	-25057.48	-0.93	-25011.51	1.86	-24968.05	2.15	-24924.54	2.48	-24896.92	8.52
-25144.76	-1.87	-25098.09	1.63	-25057.27	-0.73	-25008.59	4.78	-24960.91	9.28	-24919.80	7.23	-24899.10	6.34
-25145.64	-2.75	-25101.47	-1.76	-25055.29	1.25	-25008.55	4.82	-24967.87	2.33	-24917.58	9.44	-24899.08	6.35
-25146.57	-3.68	-25099.01	0.70	-25055.33	1.21	-25011.81	1.56	-24968.60	1.60	-24915.98	11.04	-24895.38	10.05
-25146.42	-3.53	-25100.19	-0.47	-25057.08	-0.54	-25010.80	2.57	-24965.83	4.37	-24923.66	3.37	-24896.43	9.01
-25144.19	-1.31	-25101.28	-1.56	-25058.01	-1.47	-25010.61	2.76	-24965.72	4.47	-24925.74	1.28	-24898.81	6.63
-25146.43	-3.55	-25101.18	-1.46	-25057.35	-0.81	-25003.01	10.36	-24964.49	5.70	-24917.53	9.50	-24890.94	14.49
-25146.82	-3.93	-25099.57	0.14	-25052.50	4.04	-25013.63	-0.26	-24963.63	6.56	-24926.02	1.00	-24894.88	10.55
-25146.76	-3.87	-25101.17	-1.46	-25055.92	0.62	-25014.05	-0.68	-24968.65	1.54	-24923.88	3.14	-24899.19	6.25
-25146.14	-3.25	-25099.73	-0.01	-25056.71	-0.17	-25010.77	2.60	-24967.90	2.29	-24919.01	8.01	-24897.70	7.73
-25143.56	-0.67	-25102.26	-2.55	-25056.74	-0.19	-25011.81	1.56	-24961.55	8.64	-24924.32	2.70	-24898.99	6.44
-25146.53	-3.64	-25100.55	-0.84	-25055.90	0.64	-25012.56	0.81	-24967.38	2.82	-24924.79	2.23	-24894.47	10.97
-25146.96	-4.07	-25099.57	0.15	-25056.07	0.48	-25009.34	4.03	-24968.52	1.68	-24917.18	9.85	-24899.60	5.84
-25146.50	-3.61	-25102.06	-2.34	-25055.11	1.43	-25011.72	1.65	-24964.72	5.47	-24921.62	5.40	-24900.25	5.19
-25147.13	-4.24	-25100.44	-0.73	-25056.16	0.38	-25008.54	4.83	-24968.97	1.23	-24918.48	8.54	-24893.42	12.01
-25145.19	-2.30	-25099.24	0.47	-25053.93	2.61	-25010.31	3.06	-24965.10	5.09	-24919.67	7.35	-24899.53	5.90
-25146.54	-3.65	-25101.50	-1.78	-25056.08	0.47	-25006.93	6.44	-24963.26	6.93	-24922.76	4.26	-24900.51	4.93
-25147.11	-4.22	-25100.86	-1.14	-25057.79	-1.25	-25012.12	1.25	-24963.02	7.18	-24920.57	6.45	-24896.82	8.61
-25146.11	-3.22	-25100.46	-0.74	-25057.14	-0.60	-25006.58	6.79	-24968.86	1.34	-24923.02	4.01	-24901.94	3.49
-25143.74	-0.85	-25101.47	-1.75	-25057.98	-1.44	-25009.59	3.78	-24964.30	5.89	-24922.05	4.98	-24898.28	7.15
-25146.82	-3.93	-25102.54	-2.83	-25054.71	1.83	-25010.21	3.16	-24967.52	2.68	-24920.24	6.78	-24900.69	4.75
-25147.63	-4.74	-25100.92	-1.21	-25054.45	2.09	-25008.87	4.50	-24956.80	13.39	-24924.22	2.80	-24896.27	9.17
-25146.83	-3.94	-25101.76	-2.05	-25054.83	1.71	-25010.55	2.82	-24967.16	3.04	-24922.61	4.41	-24898.63	6.80

	-25146.99	-4.10	-25101.03	-1.32	-25058.42	-1.88	-24967.66	45.71	-24923.11	47.08	-24919.15	7.87	-24893.73	11.70
	-25145.89	-3.00	-25101.61	-1.90	-25055.10	1.45	-25008.50	4.87	-24968.75	1.44	-24899.96	27.06	-24889.54	15.90
	-25147.04	-4.15	-25102.25	-2.54	-25058.09	-1.55	-25011.50	1.87	-24969.82	0.38	-24922.46	4.56	-24938.90	-33.46
	-25146.53	-3.65	-25101.94	-2.22	-25053.90	2.65	-25014.49	-1.12	-24967.74	2.45	-24919.69	7.33		
	-25145.29	-2.40	-25102.30	-2.58	-25058.22	-1.67	-25011.38	1.99	-24960.26	9.93	-24918.94	8.08		
	-25146.09	-3.20	-25101.74	-2.02	-25057.06	-0.51	-25010.89	2.48	-24966.51	3.69	-24917.34	9.69		
			-25100.44	-0.72	-25056.55	-0.01					-24923.48	3.54		
			-25101.83	-2.12	-25054.68	1.86								
					-25057.48	-0.94								
Average energies	-25146.29	-3.40	-25100.90	-1.19	-25055.69	0.85	-25009.97	3.40	-24965.70	4.50	-24920.28	6.74	-24898.47	6.97

Pu

	ground state energy	solid solution energy												
concentration in solid solution (%)	3		6		9		12		15		18		21	
number of atoms	2		4		6		8		10		12		13	
	-25108.20	28.70	-25057.67	30.07	-25007.71	30.87	-24954.72	34.71	-24911.16	29.11	-24859.55	31.55	-24828.63	37.90
	-25107.55	29.35	-25057.69	30.06	-25007.99	30.59	-24960.78	28.65	-24908.36	31.91	-24854.23	36.88	-24833.80	32.73
	-25108.17	28.73	-25060.00	27.74	-24961.42	77.17	-24912.24	77.19	-24857.34	82.93	-24832.77	58.34	-24827.98	38.55
	-25110.36	26.54	-25058.02	29.72	-25007.11	31.47	-24955.51	33.92	-24909.34	30.93	-24859.58	31.53	-24833.78	32.75
	-25109.10	27.80	-25058.76	28.99	-25011.57	27.02	-24961.44	27.99	-24911.65	28.62	-24861.20	29.91	-24836.61	29.92
	-25107.35	29.55	-25058.91	28.84	-25010.57	28.01	-24958.56	30.87	-24910.53	29.74	-24858.68	32.43	-24828.19	38.34
	-25108.67	28.24	-25058.13	29.61	-25010.42	28.17	-24957.45	31.98	-24904.44	35.83	-24855.35	35.76	-24835.93	30.60
	-25109.63	27.27	-25060.00	27.74	-25008.50	30.09	-24959.04	30.39	-24912.00	28.27	-24857.23	33.88	-24830.46	36.07
	-25109.81	27.10	-25061.49	26.26	-25002.60	35.99	-24961.26	28.16	-24903.94	36.33	-24858.68	32.43	-24831.82	34.71
	-25108.95	27.95	-25058.83	28.92	-25010.83	27.76	-24936.85	52.57	-24909.81	30.46	-24860.87	30.24	-24839.15	27.38
	-25109.84	27.06	-25059.73	28.01	-25007.20	31.39	-24959.28	30.15	-24907.96	32.31	-24855.66	35.45	-24832.46	34.07
	-25109.73	27.18	-25059.27	28.48	-25009.79	28.79	-24959.18	30.24	-24910.12	30.15	-24857.34	33.77	-24833.95	32.58
	-25109.46	27.44	-25059.20	28.54	-25007.80	30.78	-24956.71	32.72	-24908.70	31.57	-24849.90	41.21	-24832.35	34.18
	-25110.00	26.90	-25060.18	27.57	-25008.16	30.43	-24958.01	31.42	-24910.23	30.03	-24856.00	35.11	-24833.33	33.20
	-25096.34	40.57	-25059.88	27.86	-25008.38	30.21	-24962.01	27.42	-24909.62	30.65	-24860.29	30.82	-24838.21	28.32
	-25109.43	27.47	-25059.07	28.68	-25008.13	30.45	-24958.57	30.86	-24905.48	34.79	-24856.75	34.36	-24834.07	32.46
	-25109.68	27.22	-25056.40	31.34	-25009.36	29.23	-24958.94	30.48	-24910.84	29.42	-24860.65	30.46	-24834.57	31.96
	-25110.36	26.54	-25057.36	30.38	-24992.66	45.93	-24960.93	28.49	-24909.62	30.65	-24855.62	35.49	-24830.02	36.51
	-25109.89	27.01	-25059.25	28.49	-25009.63	28.95	-24959.51	29.92	-24907.92	32.35	-24861.61	29.50	-24835.37	31.16
	-25107.91	29.00	-25059.65	28.10	-25006.49	32.10	-24960.28	29.15	-24909.32	30.95	-24857.82	33.29	-24834.91	31.62
	-25110.15	26.75	-25057.94	29.80	-25010.37	28.22	-24959.10	30.33	-24906.37	33.90	-24856.63	34.48	-24832.94	33.59
	-25108.20	28.70	-25058.59	29.15	-25008.49	30.09	-24960.31	29.12	-24904.83	35.44	-24859.25	31.86	-24831.41	35.12
	-25108.91	27.99	-25060.00	27.74	-25006.28	32.31	-24959.46	29.97	-24906.95	33.32	-24859.67	31.44	-24826.54	39.99

-25108.85	28.05	-25056.37	31.37	-25007.02	31.56	-24961.63	27.79	-24912.46	27.81	-24857.62	33.49	-24830.66	35.87
-25108.93	27.97	-25056.00	31.74	-25009.52	29.06	-24950.19	39.24	-24905.70	34.57	-24860.55	30.56	-24821.92	44.61
-25107.94	28.97	-25059.52	28.23	-25009.62	28.97	-24961.09	28.33	-24908.65	31.62	-24851.01	40.10	-24834.16	32.37
-25108.77	28.13	-25058.67	29.07	-25011.37	27.21	-24958.72	30.70	-24912.56	27.71	-24856.26	34.85	-24838.14	28.39
-25107.34	29.56	-25054.81	32.93	-25007.92	30.67	-24960.33	29.09	-24908.09	32.18	-24861.16	29.95	-24836.08	30.45
-25107.98	28.92	-25060.27	27.48	-25005.95	32.63	-24961.14	28.28	-24908.03	32.24	-24858.78	32.33	-24824.94	41.59
-25109.88	27.02	-25057.72	30.03	-25009.73	28.85	-24961.67	27.76	-24904.37	35.90	-24851.66	39.45	-24832.69	33.84
-25109.89	27.02	-25059.84	27.90	-25010.84	27.74	-24956.94	32.49	-24912.17	28.10	-24860.23	30.88	-24833.98	32.55
-25108.42	28.48	-25059.59	28.16	-25003.86	34.73	-24959.91	29.52	-24911.36	28.90	-24852.16	38.95	-24828.61	37.92
-25109.62	27.28	-25059.57	28.18	-25010.08	28.50	-24956.71	32.72	-24903.20	37.07	-24852.70	38.41	-24832.85	33.68
-25107.12	29.78	-25059.49	28.26	-25010.51	28.08	-24958.43	31.00	-24912.56	27.70	-24861.63	29.48	-24834.13	32.40
-25110.02	26.88	-25059.62	28.13	-25007.17	31.42	-24956.86	32.57	-24901.24	39.03	-24841.93	49.18	-24831.56	34.97
-25109.21	27.69	-25059.81	27.94	-25004.73	33.85	-24961.98	27.45	-24910.23	30.04	-24857.16	33.95	-24833.94	32.59
-25108.54	28.37	-25059.06	28.68	-25008.17	30.41	-24957.93	31.50	-24904.54	35.73	-24861.16	29.95	-24836.92	29.61
-25109.00	27.90	-25060.07	27.67	-25005.61	32.97	-24954.13	35.30	-24907.99	32.28	-24860.58	30.53	-24826.38	40.15
-25109.80	27.10	-25059.50	28.24	-25007.52	31.07	-24960.99	28.43	-24908.46	31.81	-24856.28	34.83	-24838.74	27.79
-25109.39	27.51	-25058.23	29.52	-25009.23	29.35	-24958.48	30.95	-24909.93	30.34	-24860.89	30.22	-24834.29	32.24
-25109.55	27.36	-25058.54	29.21	-25009.83	28.75	-24959.19	30.24	-24909.87	30.40	-24856.13	34.98	-24836.01	30.52
-25109.86	27.04	-25059.88	27.87	-25008.95	29.64	-24959.59	29.84	-24908.59	31.68	-24861.98	29.13	-24837.64	28.89
-25109.10	27.80	-25055.53	32.21	-25010.62	27.96	-24958.25	31.18	-24909.02	31.25	-24858.18	32.93	-24829.38	37.15
-25109.93	26.98	-25055.64	32.11	-25008.65	29.94	-24962.58	26.85	-24907.57	32.70	-24861.50	29.61	-24838.94	27.60
-25108.74	28.17	-25059.98	27.76	-25008.49	30.10	-24960.05	29.38	-24910.33	29.94	-24857.74	33.37	-24834.94	31.59
-25109.25	27.65	-25058.05	29.69	-25007.29	31.29	-24959.58	29.85	-24909.21	31.05	-24857.16	33.95	-24835.66	30.87
-25108.69	28.21	-25057.69	30.05	-25008.50	30.09	-24958.98	30.44	-24907.94	32.33	-24857.97	33.14	-24840.65	25.88
-25109.74	27.16	-25059.67	28.08	-25007.78	30.80	-24959.26	30.17	-24908.53	31.74	-24851.79	39.32	-24830.41	36.12
-25107.86	29.04	-25057.57	30.18	-25009.40	29.19	-24959.65	29.77	-24908.75	31.52	-24855.17	35.94	-24835.36	31.17
-25108.67	28.24	-25059.26	28.48	-25008.02	30.57	-24959.98	29.45	-24908.72	31.54	-24829.62	61.49	-24837.25	29.28
-25108.17	28.74	-25058.83	28.92	-25003.07	35.52	-24956.76	32.67	-24910.21	30.06	-24861.96	29.15	-24835.77	30.76
-25108.20	28.70	-25060.32	27.42	-25007.91	30.68	-24958.91	30.52	-24909.22	31.04	-24854.67	36.44	-24836.77	29.76
-25108.45	28.45	-25058.10	29.65	-25009.19	29.39	-24959.49	29.94	-24909.45	30.82	-24859.55	31.56	-24835.54	30.99
-25107.93	28.98	-25058.44	29.31	-25006.79	31.80	-24956.81	32.61	-24908.34	31.93	-24857.71	33.40	-24837.82	28.71
-25107.17	29.74	-25058.20	29.54	-25009.30	29.28	-24954.88	34.55	-24909.00	31.27	-24858.40	32.71	-24832.13	34.40
-25109.58	27.32	-25054.44	33.30	-25008.05	30.54	-24960.30	29.13	-24910.54	29.73	-24853.26	37.85	-24835.17	31.36
-25109.84	27.06	-25059.60	28.14	-25009.00	29.58	-24957.78	31.64	-24906.41	33.86	-24860.19	30.92	-24833.42	33.11
-25108.87	28.04	-25056.06	31.68	-25007.15	31.44	-24960.54	28.88	-24909.32	30.95	-24857.84	33.27	-24833.61	32.92
-25107.95	28.95	-25058.40	29.34	-25006.97	31.61	-24959.50	29.93	-24907.81	32.46	-24862.31	28.80	-24833.95	32.58
-25108.55	28.36	-25056.44	31.30	-25009.59	29.00	-24958.88	30.55	-24906.73	33.54	-24857.52	33.59	-24834.03	32.50
-25109.47	27.44	-25057.21	30.53	-25009.06	29.53	-24951.63	37.80	-24906.19	34.08	-24862.06	29.05	-24831.82	34.71
-25108.67	28.23	-25060.05	27.70	-25007.83	30.75	-24958.61	30.82	-24903.23	37.04	-24862.95	28.16	-24836.29	30.24
-25107.76	29.14	-25059.90	27.84	-25008.62	29.96	-24962.09	27.34	-24905.69	34.58	-24862.38	28.73	-24835.80	30.73
-25108.89	28.01	-25056.40	31.34	-25005.27	33.32	-24956.65	32.78	-24911.02	29.25	-24860.81	30.30	-24826.78	39.75

-25109.32	27.58	-25054.87	32.88	-25009.81	28.77	-24959.69	29.74	-24910.84	29.43	-24862.77	28.34	-24834.84	31.69
-25109.62	27.28	-25058.73	29.01	-25008.35	30.23	-24959.62	29.81	-24911.84	28.42	-24857.04	34.07	-24823.25	43.28
-25108.89	28.02	-25060.28	27.46	-25009.11	29.48	-24955.65	33.78	-24909.97	30.30	-24856.70	34.41	-24832.91	33.62
-25107.85	29.05	-25059.47	28.27	-25009.40	29.19	-24957.55	31.88	-24904.87	35.40	-24859.32	31.79	-24838.01	28.52
-25109.63	27.27	-25060.16	27.59	-25006.61	31.98	-24960.39	29.04	-24905.04	35.23	-24855.74	35.37	-24835.70	30.83
-25107.91	28.99	-25060.28	27.46	-25010.41	28.18	-24959.88	29.55	-24907.78	32.49	-24855.09	36.01	-24837.70	28.83
-25109.18	27.73	-25059.24	28.51	-25008.68	29.91	-24958.03	31.40	-24855.59	84.68	-24860.59	30.52	-24837.57	28.96
-25108.28	28.63	-25058.87	28.87	-25006.57	32.01	-24959.23	30.20	-24898.96	41.31	-24856.68	34.43	-24830.50	36.03
-25108.83	28.07	-25057.63	30.11	-25008.43	30.16	-24959.33	30.10	-24909.52	30.75	-24856.55	34.56	-24838.19	28.34
-25109.55	27.36	-25059.41	28.34	-24958.09	80.50	-24957.49	31.94	-24911.50	28.77	-24861.89	29.22	-24839.50	27.03
-25107.81	29.09	-25053.56	34.19	-25008.99	29.60	-24959.52	29.91	-24910.78	29.49	-24858.14	32.97	-24830.32	36.22
-25106.61	30.29	-25056.42	31.33	-25007.92	30.67	-24961.58	27.84	-24906.37	33.90	-24859.39	31.72	-24835.64	30.89
-25109.19	27.71	-25060.30	27.45	-25009.70	28.88	-24906.03	83.39	-24909.21	31.06	-24826.69	64.42	-24833.71	32.82
-25110.48	26.42	-25060.89	26.86	-25008.29	30.29	-24957.70	31.73	-24909.95	30.32	-24851.98	39.13	-24836.73	29.80
-25107.85	29.05	-25059.22	28.53	-25009.75	28.84	-24958.48	30.95	-24910.54	29.73	-24858.27	32.84	-24837.43	29.10
-25110.78	26.12	-25056.87	30.87	-25010.13	28.46	-24957.23	32.20	-24907.36	32.91	-24860.30	30.81	-24830.17	36.36
-25109.20	27.71	-25057.54	30.20	-25010.38	28.20	-24957.13	32.29	-24907.02	33.25	-24854.61	36.50	-24838.16	28.38
-25108.62	28.28	-25059.59	28.15	-25009.96	28.62	-24960.05	29.38	-24906.48	33.79	-24857.10	34.01	-24827.34	39.19
-25108.25	28.66	-25060.56	27.19	-25008.46	30.12	-24960.22	29.20	-24911.84	28.43	-24859.45	31.66	-24833.73	32.80
-25109.79	27.11	-25057.49	30.26	-25009.64	28.95	-24958.32	31.11	-24907.54	32.73	-24851.66	39.45	-24833.20	33.33
-25110.11	26.79	-25059.52	28.22	-25008.82	29.77	-24959.70	29.72	-24913.67	26.60	-24853.98	37.12	-24836.47	30.06
-25109.97	26.93	-25060.37	27.38	-25008.55	30.04	-24958.82	30.60	-24911.69	28.58	-24860.16	30.95	-24830.89	35.64
-25109.61	27.29	-25059.18	28.56	-25008.01	30.58	-24958.21	31.22	-24909.71	30.55	-24860.63	30.48	-24834.21	32.32
-25107.84	29.06	-25059.29	28.46	-25009.14	29.44	-24958.62	30.80	-24911.34	28.93	-24858.00	33.11	-24833.38	33.15
-25109.42	27.48	-25058.38	29.36	-25008.64	29.95	-24959.94	29.49	-24909.25	31.02	-24863.59	27.51	-24832.51	34.02
-25109.70	27.20	-25059.67	28.08	-25007.22	31.37	-24958.42	31.01	-24908.55	31.72	-24851.81	39.30	-24836.48	30.05
-25108.41	28.50	-25059.85	27.89	-25007.50	31.08	-24958.45	30.97	-24853.12	87.15	-24858.97	32.14	-24834.93	31.60
-25109.00	27.90	-25058.74	29.01	-25007.79	30.80	-24956.34	33.09	-24908.15	32.11	-24855.11	36.00	-24834.94	31.59
-25108.21	28.69	-25058.94	28.80	-25011.29	27.29	-24958.78	30.65	-24913.82	26.45	-24857.29	33.82	-24838.73	27.80
-25108.57	28.33	-25059.71	28.04	-25008.00	30.59	-24959.88	29.55	-24902.95	37.32	-24852.98	38.13	-24825.35	41.18
-25110.18	26.72	-25060.51	27.24	-25008.09	30.50	-24905.38	84.05	-24908.53	31.74	-24857.15	33.96	-24831.54	34.99
-25109.18	27.72	-25056.99	30.75	-25009.51	29.07	-24953.80	35.63	-24904.97	35.30	-24832.99	58.12	-24834.60	31.93
-25109.48	27.43	-25059.09	28.66	-25010.63	27.96	-24956.13	33.30	-24907.13	33.14	-24860.94	30.17	-24832.39	34.14
-25108.93	27.97	-25058.70	29.05	-25007.50	31.09	-24961.88	27.55	-24908.44	31.83	-24858.21	32.90	-24830.40	36.13
-25109.45	27.45	-25059.99	27.76	-25007.18	31.40	-24959.37	30.06	-24910.22	30.05	-24859.37	31.74	-24833.64	32.89
-25108.56	28.34	-25059.42	28.33	-25005.50	33.08	-24956.53	32.90	-24904.69	35.58	-24859.35	31.76	-24834.2	32.36
-25107.75	29.16	-25059.78	27.97	-25009.72	28.86	-24956.25	33.17	-24903.78	36.49	-24860.48	30.63	-24829.9	36.67266
-25109.22	27.68	-25059.24	28.50	-25008.69	29.90	-24953.46	35.96	-24908.52	31.75	-24859.61	31.50	-24832	34.49421
-25110.61	26.29	-25058.94	28.80	-25007.70	30.89	-24952.64	36.79	-24907.96	32.31	-24857.55	33.56	-24837.9	28.62337
-25108.09	28.81	-25059.91	27.84	-25008.17	30.42	-24960.42	29.01	-24908.00	32.27	-24854.71	36.40	-24836.4	30.17265
-25109.01	27.90	-25058.61	29.13	-25010.99	27.60	-24959.17	30.26	-24899.15	41.12	-24858.60	32.51	-24837.2	29.29349

-25108.97	27.93	-25058.90	28.85	-25009.42	29.17	-24959.91	29.51	-24903.8	36.49699	-24857.20	33.91	-24834.9	31.60481
-25108.60	28.31	-25056.94	30.81	-25007.99	30.60	-24958.54	30.89	-24912.8	27.46406	-24857.11	34.00	-24834.1	32.45993
-25108.70	28.21	-25059.25	28.50	-25009.33	29.25	-24952.6	36.78234	-24909.4	30.89374	-24857.90	33.21	-24831.1	35.43485
-25107.70	29.21	-25058.95	28.79	-25009.26	29.33	-24955.9	33.53221	-24910	30.29715	-24859.91	31.20	-24838.8	27.7311
-25109.05	27.85	-25059.91	27.83	-25006.91	31.67	-24956.8	32.57935	-24906	34.2864	-24860.48	30.63	-24838.1	28.42323
-25109.83	27.07	-25059.54	28.20	-25009.41	29.18	-24958.9	30.49606	-24907	33.30485	-24856.93	34.18	-24830.4	36.1318
-25108.93	27.97	-25058.89	28.86	-25010.41	28.18	-24957.7	31.71643	-24907.9	32.39956	-24856.00	35.11	-24836.8	29.76614
-25109.84	27.07	-25059.28	28.47	-25006.68	31.91	-24955.9	33.54909			-24855.68	35.43	-24829.7	36.79479
-25110.53	26.37	-25058.47	29.27	-25009.6	28.96139	-24958.1	31.33781			-24856.89	34.22	-24831.4	35.09464
-25108.84	28.06	-25054.11	33.64	-25009.6	28.94723	-24954.8	34.59254			-24857.50	33.61	-24830.7	35.83184
-25108.83	28.07	-25056.4	31.34645	-25006	32.60937	-24960.9	28.49092			-24858.5	32.60413	-24831.1	35.38448
-25108.92	27.98	-25059.5	28.25667			-24959.4	29.98548			-24856.5	34.63477		
-25109.8	27.0768					-24957.1	32.27751			-24862.1	28.96729		
-25108.9	28.02392									-24863.6	27.55774		
-25108.9	27.95894												

Average energies	-25108.87	28.03	-25058.67	29.07	-25007.48	31.11	-24957.03	32.40	-24906.90	33.37	-24856.92	34.19	-24833.63	32.90

Th

	ground state energy	solid solution energy												
concentration in solid solution (%)	3		6		9		12		15		18		21	
number of atoms	2		4		6		8		10		12		13	
	-25114.65	27.88	-25069.45	29.56	-25022.41	33.07	-24975.23	36.72	-24935.65	32.77	-24878.07	46.82	-24867.46	35.67
	-25114.99	27.55	-25070.00	29.01	-25025.97	29.51	-24978.50	33.45	-24933.81	34.61	-24892.16	32.73	-24870.46	32.67
	-25114.44	28.09	-25067.09	31.92	-25024.07	31.41	-24976.52	35.43	-24927.87	40.55	-24884.24	40.65	-24868.94	34.19
	-25115.58	26.95	-25069.48	29.53	-25022.90	32.57	-24978.10	33.85	-24936.27	32.15	-24896.58	28.31	-24859.20	43.93
	-25114.85	27.68	-25071.05	27.95	-25023.79	31.69	-24979.78	32.17	-24934.15	34.27	-24889.96	34.93	-24873.47	29.66
	-25114.87	27.67	-25072.03	26.98	-25020.94	34.54	-24979.78	32.16	-24934.90	33.52	-24894.25	30.64	-24871.14	31.99
	-25113.16	29.37	-25070.62	28.39	-25023.20	32.28	-24979.40	32.54	-24932.76	35.66	-24889.42	35.48	-24876.91	26.22
	-25114.85	27.69	-25067.80	31.21	-25022.25	33.23	-24978.13	33.82	-24931.44	36.98	-24891.68	33.21	-24868.41	34.72
	-25114.64	27.90	-25069.01	30.00	-25027.08	28.39	-24983.08	28.87	-24936.12	32.30	-24889.74	35.16	-24869.92	33.21
	-25115.19	27.34	-25071.39	27.61	-25022.22	33.25	-24982.96	28.99	-24928.57	39.85	-24887.13	37.76	-24872.57	30.56
	-25114.92	27.61	-25069.02	29.98	-25022.07	33.41	-24981.44	30.51	-24934.45	33.97	-24888.20	36.69	-24870.45	32.67
	-25114.17	28.37	-25068.55	30.46	-25023.66	31.81	-24981.22	30.73	-24934.52	33.90	-24895.73	29.17	-24864.71	38.42
	-25113.57	28.97	-25068.20	30.81	-25003.52	51.96	-24961.93	50.02	-24934.84	33.58	-24883.57	41.32	-24868.31	34.82
	-25115.95	26.58	-25067.42	31.58	-25023.86	31.61	-24982.64	29.31	-24938.83	29.59	-24893.42	31.47	-24865.15	37.98
	-25113.06	29.47	-25069.66	29.35	-25022.30	33.17	-24978.21	33.74	-24935.20	33.22	-24888.19	36.70	-24865.62	37.51
	-25115.35	27.19	-25069.60	29.40	-25020.96	34.52	-24980.35	31.60	-24932.79	35.63	-24891.32	33.57	-24871.86	31.27
	-25114.53	28.01	-25069.86	29.14	-25024.17	31.31	-24979.39	32.56	-24934.09	34.33	-24891.20	33.69	-24868.99	34.14

-25114.22	28.31	-25070.12	28.88	-25020.80	34.68	-24980.70	31.25	-24935.21	33.21	-24891.53	33.36	-24870.78	32.35
-25115.06	27.48	-25066.74	32.26	-25025.60	29.87	-24980.24	31.71	-24934.50	33.92	-24888.96	35.93	-24861.91	41.21
-25114.99	27.55	-25069.53	29.47	-25026.07	29.41	-24974.32	37.63	-24932.60	35.82	-24876.21	48.69	-24873.11	30.02
-25113.66	28.88	-25070.06	28.95	-25020.06	35.42	-24978.23	33.72	-24938.90	29.52	-24889.03	35.86	-24855.78	47.34
-25115.16	27.37	-25069.19	29.82	-25026.16	29.32	-24977.75	34.20	-24935.32	33.10	-24894.08	30.81	-24865.75	37.38
-25113.43	29.10	-25070.24	28.77	-25020.68	34.80	-24980.89	31.06	-24931.02	37.40	-24885.38	39.52	-24870.49	32.64
-25115.85	26.68	-25070.62	28.39	-25024.93	30.55	-24979.10	32.85	-24932.60	35.82	-24894.90	29.99	-24864.22	38.91
-25114.05	28.48	-25071.07	27.94	-25022.14	33.34	-24978.77	33.18	-24931.40	37.02	-24886.42	38.47	-24871.07	32.06
-25114.25	28.29	-25067.78	31.22	-25025.93	29.54	-24980.04	31.91	-24936.74	31.68	-24884.50	40.39	-24871.51	31.62
-25113.99	28.55	-25070.92	28.09	-25022.51	32.96	-24981.09	30.86	-24937.92	30.50	-24892.10	32.80	-24861.14	41.99
-25115.40	27.13	-25069.12	29.88	-25025.69	29.78	-24980.78	31.17	-24937.24	31.18	-24894.27	30.63	-24867.56	35.56
-25112.87	29.67	-25070.12	28.89	-25025.74	29.73	-24979.41	32.54	-24934.53	33.89	-24895.05	29.85	-24874.57	28.56
-25113.54	28.99	-25069.75	29.26	-25021.38	34.10	-24975.29	36.66	-24936.27	32.15	-24888.59	36.30	-24870.71	32.42
-25114.85	27.68	-25069.86	29.14	-25025.12	30.35	-24978.92	33.03	-24937.12	31.30	-24890.82	34.08	-24843.82	59.31
-25113.19	29.34	-25068.42	30.58	-25017.84	37.64	-24981.96	29.99	-24937.35	31.07	-24892.48	32.41	-24865.37	37.76
-25115.16	27.37	-25069.12	29.89	-25024.10	31.37	-24978.08	33.87	-24930.44	37.99	-24887.71	37.18	-24867.35	35.78
-25115.27	27.26	-25063.61	35.40	-25025.59	29.89	-24977.94	34.01	-24937.17	31.25	-24890.53	34.36	-24870.21	32.92
-25113.94	28.60	-25069.43	29.58	-25023.86	31.61	-24979.93	32.02	-24934.65	33.77	-24891.49	33.40	-24860.51	42.62
-25114.82	27.72	-25069.57	29.43	-25023.75	31.73	-24976.69	35.26	-24934.17	34.25	-24886.39	38.50	-24871.16	31.97
-25114.48	28.06	-25066.84	32.16	-25026.69	28.78	-24982.08	29.87	-24936.01	32.41	-24887.31	37.59	-24859.40	43.72
-25114.06	28.47	-25072.15	26.86	-25020.49	34.99	-24971.54	40.41	-24932.58	35.84	-24888.74	36.15	-24863.96	39.17
-25115.61	26.92	-25068.62	30.39	-25020.53	34.94	-24979.85	32.10	-24934.73	33.69	-24890.82	34.07	-24868.29	34.84
-25115.70	26.83	-25071.06	27.95	-25021.49	33.98	-24976.57	35.38	-24935.60	32.82	-24891.69	33.20	-24872.08	31.05
-25113.79	28.74	-25068.99	30.02	-25026.48	29.00	-24970.10	41.85	-24933.41	35.01	-24881.92	42.97	-24870.39	32.74
-25113.82	28.71	-25071.64	27.36	-25025.65	29.83	-24970.89	41.06	-24930.75	37.67	-24888.51	36.38	-24864.62	38.51
-25113.72	28.81	-25070.72	28.29	-25024.02	31.46	-24980.56	31.39	-24925.46	42.96	-24887.51	37.38	-24872.46	30.67
-25113.59	28.95	-25071.56	27.45	-25023.26	32.22	-24982.59	29.36	-24932.56	35.86	-24893.35	31.54	-24857.86	45.26
-25114.49	28.05	-25068.83	30.18	-25025.72	29.76	-24978.55	33.40	-24930.38	38.04	-24887.22	37.68	-24861.05	42.07
-25114.20	28.33	-25071.76	27.25	-25021.09	34.39	-24981.61	30.34	-24931.69	36.73	-24883.01	41.89	-24872.83	30.30
-25115.73	26.80	-25071.23	27.77	-25024.78	30.70	-24976.26	35.69	-24931.99	36.44	-24888.66	36.23	-24875.24	27.89
-25114.46	28.07	-25070.98	28.02	-25024.92	30.56	-24977.18	34.77	-24935.25	33.17	-24890.37	34.52	-24862.08	41.05
-25114.89	27.65	-25068.68	30.32	-25022.88	32.59	-24977.04	34.91	-24928.37	40.05	-24893.60	31.29	-24864.36	38.77
-25114.70	27.84	-25068.98	30.02	-25026.75	28.73	-24981.13	30.82	-24937.44	30.99	-24885.48	39.41	-24869.41	33.72
-25114.81	27.73	-25068.39	30.61	-25022.73	32.75	-24981.39	30.56	-24923.95	44.47	-24892.04	32.85	-24864.78	38.35
-25116.04	26.49	-25069.92	29.08	-25026.09	29.38	-24980.09	31.86	-24932.99	35.43	-24881.47	43.42	-24870.55	32.57
-25114.22	28.31	-25070.42	28.59	-25022.40	33.08	-24978.46	33.48	-24929.81	38.61	-24890.86	34.04	-24865.45	37.68
-25113.89	28.65	-25069.01	30.00	-25019.09	36.39	-24977.63	34.32	-24935.61	32.81	-24889.13	35.76	-24864.58	38.55
-25115.41	27.12	-25068.34	30.67	-25024.59	30.89	-24980.04	31.91	-24930.48	37.94	-24890.45	34.44	-24868.70	34.42
-25114.04	28.50	-25068.66	30.35	-25023.27	32.20	-24984.42	27.52	-24924.16	44.26	-24886.38	38.51	-24872.74	30.39
-25113.06	29.47	-25068.35	30.65	-25024.38	31.10	-24975.46	36.49	-24933.52	34.90	-24892.68	32.21	-24863.95	39.18
-25112.26	30.27	-25066.78	32.22	-25025.15	30.33	-24983.27	28.68	-24934.48	33.94	-24892.18	32.72	-24872.03	31.10

-25114.03	28.51	-25070.83	28.17	-25026.79	28.68	-24981.10	30.85	-24928.24	40.18	-24892.89	32.00	-24863.34	39.79
-25113.16	29.37	-25069.11	29.90	-25024.05	31.43	-24979.31	32.64	-24936.23	32.19	-24889.11	35.79	-24861.99	41.13
-25114.91	27.62	-25069.68	29.33	-25018.77	36.71	-24937.42	74.53	-24887.65	80.77	-24886.76	38.13	-24862.65	40.48
-25113.70	28.83	-25068.93	30.07	-25023.68	31.80	-24976.49	35.46	-24934.00	34.42	-24890.07	34.82	-24862.57	40.56
-25113.64	28.90	-25070.17	28.84	-25025.87	29.61	-24982.96	28.99	-24930.28	38.15	-24871.85	53.04	-24863.98	39.15
-25114.99	27.55	-25070.29	28.72	-25025.95	29.53	-24981.75	30.20	-24937.32	31.10	-24887.83	37.06	-24869.01	34.12
-25112.99	29.54	-25068.18	30.82	-25025.49	29.99	-24978.31	33.64	-24936.39	32.03	-24891.33	33.56	-24864.12	39.01
-25114.29	28.24	-25071.47	27.53	-25021.96	33.52	-24982.10	29.85	-24937.23	31.19	-24891.86	33.03	-24870.68	32.45
-25113.72	28.81	-25069.78	29.22	-25023.67	31.80	-24978.80	33.14	-24933.18	35.24	-24883.97	40.92	-24868.55	34.58
-25114.35	28.18	-25069.61	29.40	-25025.46	30.01	-24980.42	31.53	-24933.89	34.53	-24889.62	35.27	-24859.60	43.53
-25112.43	30.10	-25070.93	28.08	-25021.00	34.48	-24971.91	40.04	-24935.74	32.68	-24895.32	29.57	-24870.68	32.45
-25114.98	27.55	-25070.54	28.47	-25027.06	28.41	-24976.09	35.86	-24929.00	39.42	-24889.69	35.20	-24871.23	31.90
-25114.05	28.49	-25070.27	28.74	-25024.29	31.19	-24981.55	30.40	-24928.82	39.60	-24888.59	36.31	-24869.36	33.77
-25114.85	27.68	-25066.56	32.45	-25021.95	33.53	-24982.28	29.67	-24932.65	35.77	-24886.98	37.91	-24868.05	35.08
-25114.44	28.09	-25071.24	27.77	-25024.91	30.57	-24979.24	32.71	-24935.36	33.06	-24892.64	32.25	-24868.81	34.32
-25115.15	27.38	-25069.44	29.57	-25022.46	33.02	-24977.57	34.38	-24931.60	36.82	-24889.60	35.29	-24867.82	35.31
-25114.66	27.88	-25067.61	31.39	-25021.01	34.47	-24981.27	30.67	-24934.75	33.67	-24888.26	36.63	-24868.64	34.48
-25115.21	27.33	-25071.54	27.47	-25024.41	31.07	-24976.59	35.36	-24925.30	43.12	-24888.30	36.60	-24864.76	38.36
-25113.43	29.10	-25069.42	29.59	-25023.99	31.48	-24967.32	44.63	-24936.97	31.45	-24888.43	36.47	-24867.24	35.89
-25115.94	26.59	-25068.64	30.37	-25025.27	30.20	-24982.10	29.85	-24930.76	37.66	-24889.54	35.35	-24873.28	29.85
-25114.18	28.35	-25069.67	29.34	-25024.64	30.84	-24983.05	28.90	-24936.06	32.36	-24891.71	33.19	-24861.84	41.28
-25115.17	27.37	-25069.38	29.62	-25023.07	32.41	-24978.95	33.00	-24936.68	31.74	-24865.47	59.43	-24860.88	42.24
-25115.33	27.21	-25066.53	32.48	-25025.61	29.86	-24977.15	34.80	-24885.57	82.85	-24884.92	39.97	-24864.76	38.37
-25115.35	27.18	-25069.71	29.29	-25024.86	30.61	-24972.93	39.02	-24934.96	33.46	-24892.57	32.32	-24865.55	37.58
-25115.29	27.24	-25070.41	28.60	-25025.80	29.67	-24979.36	32.59	-24931.78	36.64	-24895.21	29.69	-24872.68	30.45
-25113.68	28.85	-25067.63	31.37	-25024.62	30.86	-24977.49	34.46	-24937.01	31.41	-24890.67	34.22	-24866.70	36.43
-25115.65	26.88	-25070.75	28.26	-25024.63	30.85	-24982.38	29.57	-24934.80	33.63	-24891.71	33.19	-24866.95	36.18
-25113.79	28.75	-25069.00	30.00	-25026.50	28.98	-24980.36	31.59	-24933.74	34.68	-24891.76	33.13	-24870.71	32.42
-25115.29	27.24	-25070.44	28.56	-25024.71	30.77	-24982.23	29.72	-24936.30	32.12	-24890.68	34.21	-24872.97	30.16
-25113.51	29.02	-25065.41	33.60	-25024.11	31.37	-24975.62	36.33	-24933.66	34.76	-24888.85	36.04	-24869.51	33.62
-25113.72	28.81	-25064.77	34.23	-25020.72	34.75	-24979.49	32.46	-24935.56	32.86	-24888.27	36.62	-24862.68	40.45
-25114.21	28.33	-25069.97	29.04	-25024.25	31.23	-24979.59	32.36	-24934.26	34.16	-24886.74	38.15	-24866.18	36.95
-25114.86	27.67	-25070.72	28.28	-25025.42	30.06	-24982.01	29.94	-24933.71	34.71	-24888.63	36.26	-24871.95	31.17
-25114.36	28.17	-25071.16	27.84	-25023.64	31.83	-24982.05	29.90	-24932.72	35.70	-24897.10	27.79	-24869.89	33.24
-25114.04	28.50	-25069.00	30.00	-25023.60	31.88	-24979.53	32.42	-24937.98	30.44	-24894.21	30.69	-24863.67	39.46
-25115.51	27.03	-25066.27	32.74	-25024.77	30.71	-24983.21	28.74	-24934.36	34.06	-24885.56	39.33	-24866.93	36.20
-25114.95	27.58	-25069.34	29.66	-25023.54	31.94	-24976.44	35.51	-24938.78	29.64	-24890.80	34.09	-24866.20	36.93
-25114.97	27.56	-25068.88	30.13	-25025.79	29.68	-24976.67	35.27	-24933.60	34.82	-24886.45	38.44	-24871.19	31.94
-25115.30	27.23	-25070.60	28.40	-25021.62	33.85	-24977.92	34.03	-24934.73	33.69	-24891.39	33.50	-24862.61	40.52
-25110.67	31.87	-25067.70	31.31	-25025.29	30.19	-24980.68	31.27	-24934.02	34.40	-24891.08	33.81	-24866.43	36.70
-25113.68	28.86	-25068.76	30.25	-25026.10	29.37	-24981.08	30.86	-24934.96	33.46	-24889.87	35.02	-24866.60	36.52

	-25113.70	28.84	-25071.12	27.89	-25023.73	31.75	-24974.91	37.04	-24938.11	30.31	-24889.41	35.48	-24869.65	33.48
	-25114.19	28.34	-25066.86	32.14	-25027.38	28.10	-24978.65	33.30	-24937.92	30.50	-24888.17	36.72	-24867.22	35.91
	-25114.86	27.67	-25067.89	31.12	-25023.75	31.73	-24977.70	34.25	-24928.73	39.69	-24885.69	39.20	-24868.34	34.79
	-25114.74	27.79	-25068.40	30.61	-25026.12	29.36	-24980.36	31.59	-24937.41	31.01	-24889.98	34.91	-24867.07	36.06
	-25115.55	26.99	-25069.61	29.39	-25024.96	30.52	-24979.66	32.29	-24929.15	39.27	-24889.44	35.45	-24866.96	36.16
	-25115.93	26.60	-25071.77	27.23	-25022.17	33.30	-24981.03	30.92	-24936.36	32.06	-24894.86	30.03	-24870.49	32.64
	-25113.48	29.06	-25069.85	29.15	-25024.95	30.53	-24976.98	34.97	-24928.86	39.56	-24888.28	36.61	-24871.03	32.10
	-25113.81	28.72	-25070.60	28.40	-25024.38	31.10	-24980.25	31.70	-24935.31	33.11	-24887.59	37.30	-24870.15	32.98
	-25113.79	28.74	-25067.44	31.57	-25023.40	32.07	-24976.86	35.09	-24930.85	37.57	-24894.39	30.50	-24868.56	34.57
	-25114.74	27.80	-25069.86	29.14	-25026.56	28.92	-24982.13	29.82	-24928.29	40.13	-24888.19	36.71	-24867.46	35.67
	-25114.19	28.35	-25070.46	28.55	-25022.67	32.80	-24976.35	35.60	-24929.09	39.34	-24893.82	31.08	-24868.35	34.78
	-25114.95	27.59	-25068.80	30.20	-25023.22	32.26	-24978.56	33.38	-24934.91	33.51	-24885.26	39.64	-24869.85	33.28
	-25114.69	27.84	-25070.11	28.89	-25025.76	29.72	-24980.17	31.78	-24929.88	38.54	-24894.02	30.88	-24866.08	37.05
	-25115.26	27.27	-25065.70	33.31	-25024.96	30.52	-24981.83	30.12	-24935.13	33.29	-24889.93	34.97	-24865.21	37.92
	-25114.76	27.77	-25070.23	28.78	-25027.13	28.34	-24978.89	33.06	-24935.10	33.32	-24891.94	32.96		
	-25114.71	27.83	-25070.66	28.35	-25026.03	29.44	-24977.02	34.93	-24934.56	33.86	-24888.06	36.83		
	-25109.63	32.90	-25068.29	30.71	-25025.18	30.30	-24982.89	29.06	-24936.92	31.50	-24880.29	44.60		
	-25113.46	29.07			-25022.11	33.37								
	-25116.02	26.51			-25026.54	28.94								
	-25113.14	29.39												
Average energies	-25114.40	28.13	-25069.36	29.65	-25023.80	31.68	-24978.54	33.41	-24932.84	35.58	-24889.17	35.72	-24867.32	35.81

U

	ground state energy	solid solution energy												
concentration in solid solution (%)	3		6		9		12		15		18		21	
number of atoms	2		4		6		8		10		12		13	
	-25149.28	-1.73	-25110.10	-1.06	-25071.45	-0.91	-25030.14	1.88	-24992.02	1.50	-24950.70	4.30	-24931.26	4.49
	-25150.97	-3.41	-25108.82	0.23	-25073.30	-2.77	-25029.41	2.62	-24989.43	4.08	-24950.10	4.91	-24929.12	6.63
	-25151.67	-4.12	-25111.87	-2.83	-25070.30	0.24	-25031.39	0.63	-24990.38	3.13	-24946.27	8.74	-24931.27	4.48
	-25151.06	-3.50	-25110.25	-1.21	-25069.61	0.92	-25032.48	-0.46	-24987.46	6.05	-24954.46	0.55	-24929.32	6.43
	-25149.00	-1.45	-25110.02	-0.98	-25068.32	2.21	-25030.57	1.45	-24980.60	12.91	-24948.72	6.29	-24933.92	1.83
	-25151.01	-3.45	-25107.89	1.15	-25069.02	1.51	-25021.45	10.58	-24986.10	7.41	-24952.09	2.92	-24928.21	7.54
	-25150.38	-2.83	-25108.70	0.34	-25071.26	-0.73	-25025.22	6.81	-24990.83	2.68	-24949.33	5.68	-24930.49	5.26
	-25149.24	-1.69	-25109.95	-0.91	-25071.07	-0.54	-25032.53	-0.51	-24993.16	0.35	-24953.69	1.31	-24934.28	1.47
	-25148.74	-1.19	-25111.69	-2.64	-25070.05	0.49	-25022.94	9.09	-24989.51	4.01	-24944.25	10.75	-24925.39	10.36
	-25142.02	5.53	-25112.21	-3.17	-25068.61	1.92	-25029.95	2.07	-24990.69	2.82	-24952.51	2.49	-24932.55	3.20
	-25151.73	-4.18	-25110.44	-1.40	-25068.80	1.73	-25030.88	1.14	-24987.38	6.13	-24950.06	4.94	-24932.83	2.92

-25149.92	-2.37	-25110.56	-1.51	-25071.01	-0.48	-25029.38	2.64	-24989.90	3.62	-24947.59	7.41	-24931.75	4.00
-25149.57	-2.02	-25110.46	-1.42	-25067.48	3.06	-25027.99	4.04	-24986.68	6.83	-24952.39	2.61	-24931.47	4.28
-25148.67	-1.12	-25109.42	-0.38	-25066.85	3.69	-25028.40	3.62	-24990.29	3.23	-24947.65	7.36	-24932.32	3.43
-25150.89	-3.34	-25111.78	-2.74	-25069.85	0.68	-25030.39	1.63	-24991.66	1.85	-24943.34	11.66	-24924.44	11.31
-25148.78	-1.23	-25112.52	-3.48	-25070.07	0.46	-25029.48	2.54	-24990.04	3.47	-24949.54	5.46	-24926.23	9.52
-25149.89	-2.34	-25109.90	-0.86	-25070.67	-0.14	-25026.27	5.75	-24991.30	2.22	-24939.43	15.57	-24928.99	6.75
-25150.33	-2.78	-25110.76	-1.72	-25068.91	1.63	-25030.59	1.44	-24989.76	3.76	-24948.51	6.49	-24927.52	8.23
-25148.33	-0.78	-25109.46	-0.41	-25070.24	0.30	-25029.82	2.21	-24990.38	3.13	-24950.41	4.60	-24928.37	7.37
-25150.35	-2.80	-25110.77	-1.73	-25067.34	3.20	-25027.31	4.71	-24990.86	2.66	-24947.16	7.84	-24930.59	5.16
-25150.41	-2.86	-25110.12	-1.08	-25070.58	-0.04	-25031.92	0.11	-24988.75	4.77	-24949.97	5.03	-24930.92	4.83
-25150.02	-2.47	-25109.58	-0.54	-25067.00	3.53	-25028.66	3.36	-24992.23	1.28	-24953.48	1.53	-24931.49	4.26
-25148.91	-1.36	-25110.08	-1.04	-25063.71	6.82	-25028.86	3.16	-24993.52	0.00	-24950.95	4.06	-24928.28	7.46
-25150.27	-2.72	-25110.27	-1.23	-25067.96	2.58	-25030.81	1.21	-24990.78	2.74	-24945.61	9.39	-24930.13	5.62
-25142.09	5.46	-25111.39	-2.35	-25069.66	0.87	-25028.92	3.11	-24992.01	1.51	-24948.60	6.40	-24933.78	1.97
-25150.35	-2.80	-25108.21	0.83	-25070.27	0.26	-25030.56	1.46	-24992.16	1.36	-24949.51	5.50	-24932.87	2.88
-25148.05	-0.49	-25110.75	-1.71	-25068.93	1.60	-25029.44	2.58	-24988.95	4.56	-24947.34	7.66	-24930.33	5.42
-25149.13	-1.58	-25110.31	-1.27	-25069.51	1.02	-25029.99	2.04	-24991.22	2.29	-24951.48	3.53	-24928.44	7.31
-25150.10	-2.54	-25110.46	-1.42	-25068.31	2.22	-25030.88	1.14	-24989.65	3.86	-24945.55	9.45	-24930.20	5.55
-25150.95	-3.39	-25108.62	0.42	-25070.70	-0.16	-25030.10	1.93	-24988.94	4.57	-24951.95	3.06	-24926.68	9.07
-25149.74	-2.19	-25110.02	-0.98	-25067.92	2.61	-25030.80	1.23	-24992.29	1.22	-24946.00	9.00	-24932.18	3.57
-25150.34	-2.79	-25110.18	-1.13	-25069.16	1.37	-25029.06	2.97	-24991.68	1.83	-24950.66	4.34	-24928.82	6.93
-25151.25	-3.69	-25108.00	1.04	-25069.08	1.46	-25030.01	2.02	-24993.82	-0.31	-24948.40	6.60	-24926.73	9.02
-25150.03	-2.48	-25110.86	-1.82	-25071.82	-1.28	-25029.64	2.38	-24991.13	2.38	-24950.15	4.85	-24925.62	10.13
-25143.39	4.16	-25111.45	-2.41	-25067.90	2.63	-25033.23	-1.21	-24989.48	4.03	-24948.92	6.08	-24926.53	9.22
-25150.98	-3.43	-25110.40	-1.36	-25069.38	1.16	-25030.40	1.62	-24988.42	5.09	-24942.23	12.78	-24932.41	3.34
-25150.87	-3.32	-25111.29	-2.25	-25072.24	-1.70	-25031.62	0.40	-24988.29	5.22	-24952.41	2.59	-24926.34	9.41
-25150.00	-2.45	-25108.02	1.02	-25069.97	0.56	-25030.97	1.06	-24992.40	1.12	-24952.49	2.52	-24932.21	3.54
-25151.08	-3.53	-25109.72	-0.68	-25068.99	1.55	-25031.01	1.01	-24990.26	3.25	-24950.05	4.96	-24929.26	6.49
-25150.53	-2.98	-25110.95	-1.91	-25069.32	1.22	-25029.83	2.19	-24984.09	9.42	-24945.89	9.11	-24926.83	8.92
-25150.23	-2.68	-25110.68	-1.64	-25071.03	-0.50	-25029.89	2.14	-24987.97	5.55	-24952.28	2.72	-24930.52	5.22
-25149.39	-1.84	-25110.88	-1.84	-25070.84	-0.31	-25026.78	5.24	-24987.91	5.61	-24953.20	1.81	-24930.98	4.77
-25150.96	-3.41	-25112.44	-3.40	-25071.14	-0.61	-25028.51	3.51	-24987.85	5.67	-24949.83	5.18	-24926.27	9.48
-25149.88	-2.33	-25109.60	-0.55	-25070.52	0.02	-25030.87	1.15	-24995.48	-1.96	-24947.98	7.03	-24928.97	6.78
-25149.72	-2.16	-25108.90	0.15	-25066.45	4.08	-25031.85	0.17	-24990.27	3.24	-24951.17	3.83	-24926.84	8.91
-25150.34	-2.78	-25109.51	-0.46	-25071.99	-1.46	-25030.08	1.94	-24991.08	2.44	-24950.42	4.59	-24929.87	5.88
-25151.32	-3.77	-25110.11	-1.06	-25070.57	-0.04	-25027.87	4.15	-24992.55	0.96	-24944.77	10.23	-24932.82	2.93
-25149.65	-2.09	-25109.99	-0.95	-25069.14	1.39	-25029.58	2.44	-24990.69	2.82	-24951.23	3.77	-24930.33	5.41
-25151.20	-3.65	-25109.36	-0.32	-25069.93	0.60	-25029.04	2.99	-24992.15	1.36	-24951.50	3.50	-24931.27	4.48
-25150.04	-2.48	-25109.69	-0.64	-25069.85	0.68	-25027.96	4.06	-24988.78	4.74	-24946.66	8.34	-24932.73	3.02
-25150.59	-3.04	-25108.83	0.21	-25068.48	2.05	-25028.68	3.35	-24989.57	3.95	-24952.00	3.01	-24930.62	5.13
-25149.58	-2.03	-25111.21	-2.17	-25070.75	-0.22	-25028.84	3.18	-24991.95	1.56	-24949.01	5.99	-24932.20	3.55

-25149.64	-2.09	-25110.87	-1.83	-25068.42	2.11	-25032.17	-0.15	-24992.70	0.82	-24948.61	6.40	-24927.11	8.64
-25149.27	-1.72	-25110.24	-1.20	-25070.96	-0.43	-25027.22	4.81	-24989.97	3.55	-24949.35	5.66	-24934.05	1.70
-25151.45	-3.90	-25112.27	-3.23	-25070.93	-0.39	-25026.91	5.12	-24990.89	2.62	-24944.93	10.08	-24930.60	5.14
-25150.51	-2.96	-25109.93	-0.89	-25071.39	-0.86	-25031.12	0.90	-24990.59	2.93	-24946.50	8.50	-24927.72	8.03
-25146.02	1.54	-25110.85	-1.81	-25070.04	0.49	-25027.45	4.57	-24988.47	5.04	-24953.16	1.84	-24930.30	5.45
-25150.35	-2.80	-25109.10	-0.06	-25072.65	-2.12	-25030.25	1.78	-24990.15	3.37	-24953.03	1.97	-24929.65	6.10
-25142.57	4.99	-25111.35	-2.30	-25070.43	0.11	-25022.63	9.39	-24987.33	6.19	-24949.30	5.70	-24931.37	4.38
-25151.66	-4.11	-25107.93	1.11	-25072.23	-1.69	-25027.69	4.33	-24990.32	3.20	-24949.01	5.99	-24932.09	3.66
-25141.39	6.16	-25111.30	-2.26	-25069.71	0.82	-25032.23	-0.20	-24985.95	7.56	-24945.80	9.21	-24928.47	7.28
-25150.47	-2.92	-25109.54	-0.50	-25071.37	-0.83	-25027.41	4.62	-24982.17	11.34	-24952.48	2.52	-24921.75	14.00
-25150.33	-2.78	-25109.92	-0.88	-25070.89	-0.36	-25028.34	3.68	-24990.22	3.29	-24946.55	8.46	-24924.65	11.10
-25151.38	-3.82	-25110.25	-1.21	-25070.06	0.47	-25028.87	3.15			-24947.79	7.22	-24933.64	2.11
-25150.45	-2.90	-25110.95	-1.91	-25070.07	0.46	-25030.15	1.87			-24945.45	9.55	-24927.74	8.01
-25150.99	-3.44	-25110.62	-1.58	-25071.31	-0.78	-25029.95	2.07			-24952.84	2.17	-24934.25	1.50
-25149.89	-2.33	-25110.61	-1.57	-25072.30	-1.77	-25029.83	2.19			-24950.37	4.64	-24930.89	4.86
-25151.12	-3.56	-25111.00	-1.96	-25069.20	1.33					-24948.49	6.52	-24931.35	4.40
-25149.14	-1.59	-25108.71	0.33	-25068.78	1.75					-24949.14	5.86	-24934.13	1.62
-25150.46	-2.90	-25109.99	-0.95	-25071.11	-0.58					-24941.86	13.14	-24922.12	13.63
-25148.04	-0.49	-25107.56	1.48	-25071.84	-1.31					-24947.19	7.81	-24929.30	6.45
-25150.72	-3.17	-25109.57	-0.52	-25070.03	0.51					-24949.05	5.95	-24925.14	10.61
-25150.84	-3.28	-25110.75	-1.71	-25067.35	3.19							-24927.35	8.40
-25150.00	-2.45	-25110.50	-1.46	-25070.98	-0.45							-24932.25	3.50
-25150.97	-3.42			-25070.99	-0.46								
-25152.19	-4.64			-25071.84	-1.30								
-25149.57	-2.02			-25065.72	4.81								
-25148.58	-1.03			-25068.67	1.86								
-25149.59	-2.03			-25068.00	2.53								
-25149.95	-2.39			-25069.24	1.29								
-25150.14	-2.59			-25069.37	1.16								
-25150.91	-3.36			-25071.03	-0.50								
-25151.22	-3.67												
-25148.45	-0.89												
-25149.15	-1.60												
-25150.33	-2.77												
-25150.62	-3.06												
-25149.85	-2.30												
-25151.07	-3.51												
-25150.66	-3.11												
-25150.21	-2.66												
-25149.64	-2.09												

Average energies	-25149.71	-2.15	-25110.18	-1.14	-25069.81	0.73	-25029.34	2.69	-24989.90	3.62	-24949.04	5.97	-24929.73	6.02

$Ca_{1-x}M_xZrTi_{2-2x}Fe_{2x}O_7$ M=(Ce, Pu, Th, U)

Ti(1)Ti(3)

N.B. All stated energies are in eV

Ce

	ground state energy	solid solution energy												
concentration in solid solution (%)	3		6		9		12		15		18		21	
number of atoms	2		4		6		8		10		12		13	
	-25154.13	-11.24	-25118.38	-18.66	-25078.07	-21.53	-25042.25	-28.88	-25003.97	-33.78	-24966.98	-39.96	-24930.59	-25.16
	-25154.64	-11.75	-25116.81	-17.10	-25073.58	-17.04	-25041.19	-27.82	-25005.75	-35.55	-24969.05	-42.03	-24933.07	-27.64
	-25153.42	-10.53	-25116.41	-16.70	-25080.04	-23.50	-25043.71	-30.34	-25004.38	-34.18	-24968.24	-41.22	-24932.27	-26.83
	-25153.05	-10.16	-25116.39	-16.68	-25077.56	-21.02	-25042.18	-28.81	-25006.19	-35.99	-24964.88	-37.85	-24931.37	-25.93
	-25153.23	-10.35	-25116.24	-16.53	-25079.08	-22.54	-25043.18	-29.81	-25004.10	-33.91	-24968.93	-41.90	-24933.21	-27.77
	-25154.41	-11.52	-25117.41	-17.69	-25080.11	-23.57	-25042.38	-29.01	-25005.27	-35.08	-24968.29	-41.27	-24924.83	-19.39
	-25154.50	-11.61	-25116.54	-16.82	-25077.53	-20.99	-25043.57	-30.20	-25008.73	-38.53	-24965.79	-38.77	-24925.72	-20.28
	-25153.72	-10.83	-25118.08	-18.36	-25078.49	-21.95	-25036.24	-22.87	-25005.38	-35.18	-24968.62	-41.59	-24933.60	-28.16
	-25154.18	-11.29	-25116.82	-17.10	-25078.29	-21.75	-25040.93	-27.57	-25000.35	-30.15	-24968.31	-41.29	-24927.95	-22.51
	-25153.74	-10.85	-25118.97	-19.26	-25040.60	15.94	-25006.43	6.94	-24966.44	3.76	-24931.12	-4.10	-24929.86	-24.42
	-25154.06	-11.17	-25116.68	-16.97	-25079.79	-23.25	-25042.10	-28.73	-25006.55	-36.35	-24969.98	-42.96	-24929.80	-24.36
	-25154.04	-11.15	-25116.26	-16.54	-25079.65	-23.11	-25041.07	-27.70	-24998.35	-28.15	-25012.58	-85.56	-24934.14	-28.70
	-25153.24	-10.35	-25116.62	-16.90	-25077.31	-20.77	-25040.30	-26.94	-25006.69	-36.49	-24969.69	-42.67	-24931.15	-25.71
	-25154.87	-11.98	-25115.05	-15.34	-25080.17	-23.62	-25043.92	-30.55	-25005.21	-35.01	-24964.39	-37.36	-24930.19	-24.75
	-25154.09	-11.21	-25115.71	-15.99	-25079.81	-23.27	-25040.79	-27.42	-25007.20	-37.00	-24963.62	-36.60	-24931.21	-25.77
	-25153.89	-11.00	-25117.99	-18.27	-25077.46	-20.92	-25043.01	-29.64	-25003.46	-33.27	-24968.47	-41.45	-24930.19	-24.76
	-25154.32	-11.44	-25116.05	-16.33	-25079.92	-23.38	-25043.77	-30.40	-25000.31	-30.12	-24970.36	-43.34	-24931.45	-26.02
	-25151.80	-8.91	-25115.57	-15.86	-25078.79	-22.25	-25041.50	-28.13	-25003.06	-32.86	-24966.24	-39.21	-24933.25	-27.81
	-25153.72	-10.83	-25117.32	-17.60	-25080.24	-23.70	-25040.11	-26.74	-25001.94	-31.74	-24966.92	-39.89	-24928.83	-23.40
	-25153.54	-10.65	-25116.31	-16.59	-25042.57	13.97	-25001.94	11.43	-24966.75	3.45	-24968.21	-41.18	-24931.01	-25.57
	-25153.82	-10.93	-25117.74	-18.03	-25080.64	-24.10	-25041.51	-28.14	-25005.77	-35.57	-24966.06	-39.04	-24933.87	-28.43
	-25152.36	-9.47	-25116.58	-16.87	-25081.41	-24.87	-25042.41	-29.04	-25004.17	-33.98	-24968.90	-41.88	-24928.44	-23.01
	-25153.94	-11.05	-25118.33	-18.61	-25079.35	-22.80	-25041.96	-28.59	-25005.32	-35.13	-24969.63	-42.61	-24932.88	-27.44
	-25151.80	-8.91	-25117.40	-17.68	-25081.11	-24.57	-25041.30	-27.94	-25004.82	-34.63	-24934.14	-7.12	-24933.76	-28.32
	-25153.73	-10.84	-25116.92	-17.21	-25081.11	-24.57	-25044.71	-31.34	-25004.61	-34.42	-24969.18	-42.16	-24930.69	-25.25
	-25154.40	-11.51	-25118.42	-18.70	-25079.11	-22.56	-25040.29	-26.93	-25001.70	-31.51	-24965.02	-37.99	-24931.78	-26.34
	-25154.12	-11.23	-25116.78	-17.07	-25077.39	-20.85	-25041.31	-27.94	-25006.47	-36.27	-24963.31	-36.29	-24927.23	-21.80
	-25153.46	-10.57	-25117.45	-17.74	-25080.48	-23.94	-25043.91	-30.54	-25005.58	-35.38	-24934.80	-7.77	-24934.15	-28.71
	-25154.68	-11.79	-25117.21	-17.49	-25078.33	-21.78	-25040.68	-27.31	-25006.15	-35.96	-24961.32	-34.29	-24928.31	-22.87

-25154.20	-11.31	-25117.23	-17.51	-25080.35	-23.81	-25043.42	-30.05	-25006.39	-36.20	-24967.59	-40.57	-24932.41	-26.97
-25154.06	-11.17	-25116.81	-17.09	-25079.71	-23.17	-25043.39	-30.02	-25008.41	-38.22	-24969.21	-42.19	-24932.01	-26.57
-25154.04	-11.15	-25117.19	-17.47	-25078.06	-21.52	-25043.95	-30.58	-25001.78	-31.59	-24963.37	-36.35	-24931.15	-25.71
-25152.60	-9.71	-25116.40	-16.68	-25078.29	-21.74	-25045.06	-31.69	-25005.90	-35.70	-24928.44	-1.41	-24931.76	-26.32
-25153.66	-10.77	-25117.17	-17.45	-25079.38	-22.83	-25041.39	-28.02	-25006.78	-36.58	-24972.04	-45.01	-24931.02	-25.58
-25154.55	-11.66	-25118.41	-18.69	-25080.66	-24.12	-25044.29	-30.92	-25006.04	-35.84	-24966.97	-39.94	-24927.15	-21.72
-25153.86	-10.97	-25117.11	-17.40	-25078.79	-22.25	-25040.50	-27.13	-25006.03	-35.83	-24968.95	-41.93	-24929.70	-24.27
-25154.70	-11.81	-25117.12	-17.41	-25077.70	-21.16	-25043.29	-29.93	-25005.27	-35.08	-24968.89	-41.86	-24930.66	-25.22
-25153.33	-10.44	-25116.06	-16.34	-25079.39	-22.85	-25038.23	-24.86	-25007.34	-37.14	-25008.58	-81.55	-24925.49	-20.05
-25154.34	-11.45	-25116.02	-16.30	-25081.56	-25.02	-25043.79	-30.42	-25006.22	-36.02	-24964.52	-37.50	-24922.35	-16.91
-25154.30	-11.41	-25116.96	-17.25	-25079.02	-22.48	-25039.46	-26.09	-25003.13	-32.93	-24970.27	-43.25	-24927.40	-21.96
-25154.95	-12.06	-25116.86	-17.15	-25080.49	-23.95	-25044.77	-31.40	-24969.52	0.68	-24934.86	-7.83	-24933.25	-27.82
-25154.72	-11.83	-25080.03	19.69	-25040.88	15.66	-25004.23	9.14	-25002.75	-32.55	-24966.29	-39.27	-24933.34	-27.90
-25154.09	-11.20	-25116.30	-16.59	-25080.90	-24.36	-25044.01	-30.64	-25006.17	-35.97	-24968.38	-41.36	-24931.53	-26.09
-25154.80	-11.91	-25115.41	-15.70	-25080.42	-23.88	-25044.19	-30.82	-25005.69	-35.49	-24967.09	-40.07	-24929.12	-23.69
-25153.80	-10.91	-25115.37	-15.65	-25077.72	-21.17	-25040.31	-26.94	-25004.56	-34.36	-24970.95	-43.92	-24930.69	-25.25
-25154.64	-11.75	-25117.72	-18.00	-25081.32	-24.77	-25040.62	-27.25	-24969.02	1.18	-24929.72	-2.70	-24932.42	-26.98
-25154.18	-11.29	-25079.90	19.82	-25044.56	11.98	-25009.35	4.02	-24968.76	1.43	-24928.56	-1.54	-24929.78	-24.35
-25153.05	-10.16	-25115.57	-15.86	-25079.75	-23.21	-25004.93	8.44	-25007.20	-37.00	-24965.73	-38.70	-24925.02	-19.58
-25154.03	-11.14	-25116.40	-16.68	-25077.90	-21.36	-25043.35	-29.99	-25004.12	-33.93	-24969.78	-42.75	-24931.51	-26.08
-25153.80	-10.91	-25117.35	-17.64	-25078.79	-22.25	-25042.54	-29.17	-25007.31	-37.12	-24966.31	-39.29	-24928.37	-22.93
-25153.79	-10.90	-25116.49	-16.78	-25078.95	-22.41	-25043.38	-30.01	-25004.85	-34.65	-24966.45	-39.42	-24932.22	-26.79
-25152.93	-10.04	-25117.56	-17.85	-25078.44	-21.90	-25039.22	-25.85	-25004.47	-34.27	-25011.03	-84.01	-24933.23	-27.79
-25153.92	-11.03	-25115.91	-16.20	-25080.69	-24.15	-25042.63	-29.27	-24928.88	41.31	-24968.25	-41.23	-24929.61	-24.17
-25154.99	-12.10	-25079.37	20.34	-25041.96	14.58	-25004.18	9.19	-25007.55	-37.35	-24970.56	-43.54	-24928.61	-23.17
-25152.98	-10.09	-25116.76	-17.05	-25079.97	-23.43	-25040.80	-27.43	-25002.96	-32.77	-24931.53	-4.51	-24927.14	-21.71
-25153.42	-10.53	-25117.91	-18.19	-25078.79	-22.25	-25043.19	-29.82	-24999.34	-29.14	-24966.77	-39.75	-24933.08	-27.65
-25153.86	-10.97	-25116.75	-17.03	-25077.61	-21.07	-25043.58	-30.21	-25007.52	-37.33	-24971.00	-43.98	-24830.00	75.43
-25153.22	-10.33	-25117.70	-17.98	-25078.88	-22.34	-25043.53	-30.16	-25007.57	-37.37	-24961.91	-34.89	-24829.18	76.26
-25153.49	-10.61	-25116.63	-16.92	-25077.59	-21.05	-25040.04	-26.67	-25004.86	-34.67	-24966.94	-39.92	-24827.93	77.51
-25152.73	-9.84	-25115.72	-16.01	-25077.84	-21.30	-25040.44	-27.07	-25001.91	-31.72	-24963.73	-36.71	-24827.45	77.99
-25154.45	-11.57	-25119.08	-19.36	-25079.53	-22.98	-25040.26	-26.89	-25004.28	-34.09	-24968.35	-41.33	-24827.06	78.37
-25154.93	-12.04	-25116.78	-17.07	-25078.08	-21.53	-25041.11	-27.74	-25003.77	-33.57	-24965.37	-38.34	-24827.59	77.84
-25154.81	-11.92	-25115.88	-16.17	-25081.21	-24.67	-25045.12	-31.75	-25001.91	-31.71	-24965.14	-38.11	-24826.58	78.86
-25154.00	-11.11	-25116.93	-17.22	-25078.81	-22.27	-25042.23	-28.86	-25001.27	-31.08	-24968.69	-41.67	-24823.99	81.45
-25153.39	-10.50	-25116.02	-16.30	-25077.57	-21.03	-25043.02	-29.65	-24963.88	6.32	-24865.27	61.75	-24827.69	77.75
-25154.73	-11.85	-25115.94	-16.22	-25078.50	-21.96	-25041.55	-28.18	-25002.61	-32.41	-24868.34	58.68	-24831.28	74.16
-25152.81	-9.93	-25116.75	-17.04	-25078.99	-22.45	-25043.19	-29.82	-24923.94	46.26	-24876.59	50.44	-24827.79	77.65
-25111.91	30.97	-25065.18	34.53	-25019.14	37.40	-24967.62	45.75	-24919.80	50.40	-24872.73	54.29	-24825.67	79.76
-25110.67	32.21	-25062.77	36.94	-25016.21	40.33	-24969.79	43.58	-24921.11	49.08	-24876.51	50.52	-24829.27	76.16
-25112.08	30.81	-25064.24	35.48	-25014.36	42.18	-24964.74	48.63	-24922.61	47.59	-24872.58	54.44	-24831.53	73.91

-25111.10	31.79	-25063.61	36.10	-25017.12	39.42	-24965.97	47.40	-24920.22	49.97	-24875.12	51.90	-24819.48	85.96
-25112.29	30.59	-25063.89	35.83	-25020.11	36.43	-24967.77	45.60	-24919.75	50.45	-24872.45	54.57	-24823.62	81.81
-25111.98	30.91	-25064.30	35.42	-25018.79	37.75	-24968.61	44.76	-24920.28	49.92	-24870.76	56.26	-24829.64	75.80
-25108.85	34.04	-25064.97	34.75	-25017.51	39.03	-24969.15	44.21	-24923.25	46.94	-24875.06	51.96	-24827.93	77.51
-25111.95	30.94	-25063.90	35.81	-25015.76	40.78	-24969.53	43.84	-24924.37	45.83	-24875.21	51.82	-24825.20	80.23
-25110.35	32.54	-25063.75	35.97	-25016.23	40.31	-24966.50	46.87	-24926.03	44.17	-24875.24	51.78	-24829.65	75.79
-25111.75	31.14	-25065.87	33.85	-25016.54	40.00	-24969.12	44.25	-24921.82	48.38	-24873.96	53.06	-24828.24	77.20
-25110.75	32.14	-25064.94	34.77	-25015.92	40.63	-24968.84	44.53	-24925.02	45.18	-24870.98	56.04	-24826.28	79.15
-25111.94	30.95	-25065.07	34.65	-25014.18	42.37	-24968.50	44.87	-24920.32	49.88	-24872.67	54.36	-24830.71	74.73
-25110.66	32.23	-25062.79	36.93	-25016.88	39.66	-24969.92	43.45	-24922.72	47.48	-24878.36	48.66	-24826.98	78.46
-25111.81	31.08	-25065.26	34.46	-25013.41	43.13	-24964.91	48.46	-24919.52	50.67	-24875.05	51.97	-24829.24	76.20
-25111.85	31.04	-25062.51	37.20	-25016.47	40.08	-24969.23	44.14	-24915.43	54.76	-24873.58	53.45	-24830.87	74.57
-25111.68	31.21	-25065.70	34.01	-25018.44	38.11	-24964.75	48.61	-24921.69	48.51	-24874.48	52.54	-24828.04	77.40
-25112.04	30.85	-25064.56	35.16	-25012.99	43.55	-24968.05	45.32	-24921.79	48.40	-24866.15	60.87	-24827.23	78.20
-25110.77	32.12	-25063.29	36.42	-25018.66	37.88	-24971.81	41.56	-24924.02	46.17	-24878.07	48.95	-24831.23	74.20
-25111.16	31.73	-25064.39	35.33	-25017.04	39.50	-24963.93	49.44	-24924.80	45.40	-24876.44	50.58	-24818.90	86.54
-25110.71	32.18	-25063.50	36.22	-25014.06	42.48	-24969.00	44.37	-24921.19	49.01	-24877.61	49.42	-24831.73	73.70
-25107.72	35.16	-25065.35	34.37	-25016.79	39.75	-24968.60	44.77	-24922.70	47.50	-24875.84	51.18	-24832.18	73.25
-25111.55	31.34	-25062.96	36.76	-25017.68	38.86	-24969.30	44.07	-24923.03	47.16	-24876.89	50.14	-24821.53	83.91
-25110.49	32.40	-25064.93	34.78	-25017.65	38.90	-24968.02	45.35	-24919.28	50.92	-24872.95	54.07	-24826.33	79.10
-25112.43	30.46	-25065.91	33.80	-25016.63	39.91	-24971.74	41.63	-24919.45	50.74	-24877.51	49.51	-24830.15	75.29
-25111.16	31.73	-25061.20	38.51	-25017.31	39.24	-24967.70	45.67	-24921.29	48.91	-24874.55	52.48	-24823.49	81.95
-25111.05	31.84	-25063.33	36.38	-25014.75	41.79	-24968.83	44.54	-24921.76	48.44	-24873.54	53.48	-24828.74	76.69
-25111.29	31.60	-25063.08	36.64	-25017.24	39.31	-24968.22	45.15	-24920.39	49.81	-24876.31	50.71	-24828.52	76.92
-25112.08	30.81	-25064.14	35.58	-25009.98	46.56	-24968.26	45.11	-24924.74	45.46	-24879.51	47.52	-24829.03	76.41
-25111.50	31.39	-25063.80	35.92	-25018.78	37.76	-24968.60	44.77	-24924.18	46.01	-24873.16	53.87	-24829.29	76.15
-25111.63	31.26	-25064.61	35.11	-25017.44	39.11	-24972.60	40.77	-24922.03	48.17	-24871.12	55.90	-24827.69	77.75
-25109.83	33.06	-25064.71	35.00	-25017.79	38.75	-24966.92	46.45	-24923.85	46.34	-24873.06	53.97	-24828.29	77.15
-25110.26	32.63	-25061.38	38.33	-25015.08	41.46	-24969.81	43.56	-24912.30	57.89	-24875.47	51.55	-24827.09	78.35
-25112.89	30.00	-25064.59	35.12	-25015.02	41.53	-24949.99	63.38	-24917.87	52.33	-24872.49	54.53	-24828.26	77.17
-25110.51	32.38	-25064.09	35.63	-25017.22	39.32	-24971.02	42.35	-24921.57	48.63	-24871.04	55.98	-24824.72	80.72
-25110.93	31.96	-25064.45	35.27	-25018.88	37.66	-24967.22	46.15	-24918.79	51.40	-24866.83	60.19	-24820.96	84.48
-25111.13	31.76	-25064.01	35.71	-25017.91	38.63	-24966.18	47.19	-24918.87	51.33	-24876.21	50.81	-24831.10	74.33
-25111.32	31.57	-25065.03	34.69	-25011.96	44.58	-24970.29	43.08	-24921.01	49.19	-24874.34	52.69	-24830.01	75.43
-25110.76	32.13	-25066.26	33.45	-25015.52	41.02	-24966.53	46.84	-24920.60	49.60	-24873.56	53.47		
-25110.27	32.62	-25063.73	35.99	-25018.43	38.11	-24967.07	46.30	-24922.73	47.46	-24872.01	55.01		
-25112.75	30.13	-25064.07	35.64	-25012.98	43.56	-24970.69	42.68	-24922.44	47.76	-24875.18	51.84		
-25112.42	30.47	-25065.56	34.16	-25012.88	43.67	-24970.57	42.80	-24925.65	44.55	-24870.17	56.85		
-25111.52	31.37	-25063.04	36.68	-25016.18	40.36	-24964.42	48.95	-24918.71	51.48	-24875.17	51.86		
-25111.86	31.03	-25063.26	36.46	-25018.06	38.48	-24967.29	46.07	-24920.49	49.71	-24875.63	51.39		
-25111.56	31.33	-25065.63	34.08	-25013.18	43.37	-24963.21	50.15	-24920.20	50.00	-24873.57	53.45		

	-25111.59	31.29	-25064.21	35.50	-25015.80	40.75	-24970.42	42.95	-24918.59	51.60	-24873.97	53.05		
	-25111.05	31.84	-25065.88	33.84	-25016.20	40.34	-24966.34	47.03	-24925.82	44.37	-24875.88	51.14		
	-25112.44	30.44	-25064.80	34.92	-25016.87	39.67	-24969.52	43.84			-24879.74	47.28		
	-25109.77	33.12	-25063.67	36.04	-25016.14	40.40	-24970.25	43.12			-24868.62	58.40		
	-25112.46	30.43	-25063.32	36.40	-25013.25	43.30	-24970.58	42.79						
	-25111.61	31.27	-25063.67	36.05	-25017.53	39.01	-24964.29	49.08						
	-25111.76	31.13			-25016.77	39.77	-24966.69	46.68						
	-25111.05	31.84			-25015.60	40.94	-24971.85	41.52						
	-25110.30	32.59			-25016.89	39.65								
Average energies	-25135.05	7.84	-25093.39	6.32	-25049.81	6.73	-25007.83	5.54	-24967.42	2.77	-24924.55	2.48	-24882.98	22.45

Pu

	ground state energy	solid solution energy												
concentration in solid solution (%)	3		6		9		12		15		18		21	
number of atoms	2		4		6		8		10		12		13	
	-25138.6	-1.74252	-25086.3	1.399932	-25032.2	6.416771	-24980.9	8.534834	-24928.7	11.60507	-24873.7	17.42753	-24840.5	26.07599
	-25137.7	-0.78777	-25084.7	3.013177	-25029.9	8.732853	-24977.7	11.68993	-24919.9	20.40918	-24872.3	18.76134	-24842.1	24.39377
	-25138.5	-1.62104	-25084.3	3.40043	-25031.4	7.193939	-24982	7.416536	-24926.1	14.16249	-24874.7	16.39354	-24845.7	20.7953
	-25136.1	0.819202	-25085.1	2.628137	-25030	8.562454	-24980	9.413301	-24926.3	13.96165	-24874.2	16.95403	-24846.3	20.19821
	-25138.1	-1.22623	-25086.7	1.001717	-25031.9	6.666796	-24979.1	10.28231	-24925	15.26284	-24871.7	19.44341	-24845.8	20.74565
	-25137.9	-0.95973	-25084.8	2.982988	-25030.8	7.770741	-24980.3	9.158699	-24927.6	12.64335	-24872.3	18.79834	-24847.2	19.34862
	-25138	-1.05537	-25084.2	3.580471	-25033.3	5.313099	-24977	12.4189	-24926.8	13.44766	-24874.5	16.6141	-24846.2	20.32724
	-25137.2	-0.27575	-25085	2.783896	-25031.8	6.832784	-24981.3	8.110947	-24924.3	15.93572	-24869.2	21.944	-24844.6	21.9339
	-25137.7	-0.82495	-25086	1.696374	-25032.4	6.180342	-24923.1	66.29495	-24873.6	66.64892	-24850.5	40.57904	-24846.6	19.96985
	-25138.4	-1.53577	-25083.8	3.929758	-25028.5	10.06414	-24978.6	10.83595	-24926.4	13.89832	-24872.1	19.01931	-24843.6	22.88575
	-25137.3	-0.36582	-25085.4	2.350879	-25034	4.622476	-24978.1	11.32844	-24923.5	16.76935	-24873.3	17.85443	-24845.3	21.19709
	-25138.2	-1.30029	-25083.7	4.033056	-25032.1	6.487689	-24980.3	9.131578	-24926.5	13.74333	-24869.4	21.71439	-24850.8	15.6961
	-25137.8	-0.85635	-25085.2	2.508385	-25031.1	7.533444	-24980.6	8.863424	-24922.5	17.77911	-24871.6	19.54079	-24848.8	17.72676
	-25137.7	-0.76359	-25087.5	0.218405	-25030.7	7.916463	-24980.6	8.829882	-24926.6	13.71294	-24872.3	18.78757	-24845.1	21.4723
	-25139.1	-2.15324	-25083.9	3.861751	-25031.3	7.282445	-24980.6	8.792737	-24926.8	13.46224	-24872.3	18.77498	-24849.9	16.66167
	-25137.8	-0.9134	-25085.4	2.364639	-25031.9	6.704604	-24977.5	11.90927	-24926.6	13.70467	-24875	16.1484	-24842.2	24.35105
	-25137.7	-0.79569	-25084.7	3.015109	-25031.4	7.138751	-24980.1	9.361729	-24927.4	12.89287	-24873.8	17.29124	-24850.4	16.14101
	-25138	-1.12433	-25085.8	1.989214	-25032.5	6.082659	-24979.5	9.90193	-24927.1	13.17104	-24870.5	20.62923	-24843.2	23.35588
	-25137.8	-0.88973	-25086.7	1.038519	-25035.3	3.255234	-24979.6	9.856062	-24925.3	14.99893	-24873.8	17.26288	-24848.9	17.66069
	-25137.7	-0.8339	-25084	3.792549	-25030.8	7.76082	-24976.1	13.31639	-24923.9	16.32847	-24872.5	18.5763	-24849.5	17.03616
	-25137.3	-0.36297	-25086.7	1.088596	-25031.7	6.861391	-24977.5	11.9237	-24925.4	14.82827	-24872.2	18.9274	-24846.9	19.65872
	-25138	-1.13574	-25085.2	2.583618	-25031.6	7.032752	-24978	11.42521	-24923.9	16.37425	-24874.4	16.71152	-24848.2	18.28195
	-25139.6	-2.68291	-25082.2	5.512755	-25031.1	7.458801	-24982	7.443964	-24924.3	16.00755	-24875.2	15.92259	-24848.6	17.89872

-25139.4	-2.51294	-25084.5	3.263913	-25031.1	7.459148	-24979.7	9.76745	-24922.9	17.36782	-24873.3	17.82711	-24846.3	20.22513
-25138.9	-1.9725	-25084	3.708254	-25033.2	5.342261	-24979.1	10.32313	-24922.6	17.65238	-24875.4	15.73885	-24846	20.48324
-25138.5	-1.58821	-25085.2	2.556423	-25029.4	9.145647	-24975	14.38228	-24926.5	13.73692	-24874.2	16.91639	-24846.9	19.6342
-25137.1	-0.19486	-25086.5	1.252001	-25031.3	7.303925	-24980.3	9.16336	-24927.4	12.82267	-24876.6	14.46793	-24843	23.57904
-25138.5	-1.55112	-25081.7	6.085813	-25031	7.571803	-24981.9	7.566825	-24927.1	13.11939	-24873.3	17.79141	-24847.2	19.2951
-25138.3	-1.37752	-25084.3	3.466422	-25030.7	7.855201	-24981.1	8.314205	-24928.4	11.82589	-24879.1	11.97815	-24848.9	17.66515
-25138.6	-1.71922	-25085.2	2.527479	-25032.9	5.643681	-24972.9	16.49427	-24923.2	17.11066	-24872.4	18.74771	-24847.4	19.16178
-25138.1	-1.1556	-25084.5	3.27	-24981.1	57.52997	-24923	66.3861	-24877.9	62.40133	-24877.3	13.83396	-24846.8	19.68915
-25138.9	-2.03684	-25083	4.720688	-25029.9	8.671597	-24977	12.43163	-24927.6	12.67706	-24843.4	47.7252	-24848.2	18.30668
-25138.2	-1.32786	-25085.1	2.644818	-25032.7	5.920435	-24979.9	9.497367	-24927.3	12.92989	-24868.5	22.64663	-24842.4	24.16937
-25139.2	-2.26691	-25085.2	2.545582	-25031.2	7.419614	-24980.7	8.711615	-24927.8	12.4954	-24876.2	14.86675	-24849.4	17.11221
-25138.1	-1.18837	-25083.6	4.14219	-25031.7	6.840694	-24980.4	9.056073	-24924.8	15.43302	-24876.6	14.49155	-24840.8	25.68541
-25139	-2.0468	-25085.1	2.627148	-25029.9	8.67163	-24978.6	10.78203	-24922.4	17.88432	-24872.8	18.27263	-24844.3	22.2003
-25137.3	-0.35447	-25085.4	2.386641	-25031.5	7.10759	-24980.5	8.953312	-24925.9	14.35788	-24871.7	19.36003	-24840.5	26.04321
-25138.7	-1.76672	-25087.4	0.374646	-25029.6	9.027699	-24977	12.46375	-24925.4	14.84813	-24874.9	16.24589	-24843	23.50966
-25138.1	-1.23709	-25086	1.715089	-25029.8	8.814594	-24980	9.438777	-24928.6	11.68905	-24867.8	23.28976	-24850.1	16.41326
-25138.5	-1.64629	-25085.5	2.207708	-25033.1	5.513262	-24980.4	9.00944	-24926	14.2925	-24872.8	18.33958	-24847.7	18.80305
-25137.6	-0.72632	-25082.1	5.633785	-25031.9	6.670328	-24977.1	12.28872	-24927.4	12.88557	-24877.4	13.70863	-24847.2	19.36498
-25137.7	-0.79852	-25084.3	3.459067	-25033.1	5.50911	-24978.7	10.75121	-24925.6	14.70133	-24876.7	14.4132	-24850.5	16.07101
-25138.8	-1.85998	-25086.7	1.064239	-25030.1	8.520403	-24979	10.38776	-24925.2	15.05912	-24874	17.10544	-24849.6	16.89424
-25136.2	0.693418	-25081.8	5.978017	-25031.3	7.237642	-24976.4	13.05965	-24927.5	12.75094	-24870.2	20.89007	-24848.8	17.72969
-25137.6	-0.70873	-25086.6	1.190184	-25029.4	9.201865	-24979.7	9.74182	-24923.6	16.68228	-24868.8	22.26664	-24848	18.55467
-25138.4	-1.53762	-25085.3	2.428074	-25031.9	6.696815	-24979.5	9.956791	-24927.2	13.02377	-24872.9	18.25297	-24849.5	17.05225
-25139.2	-2.27822	-25084.5	3.269161	-25033	5.562619	-24977.6	11.87334	-24923.5	16.72523	-24874.4	16.73215	-24843.9	22.62764
-25138.1	-1.21847	-25086.6	1.102342	-25035.6	3.025935	-24978	11.42187	-24920.1	20.15358	-24873.2	17.95056	-24850.1	16.39765
-25138.8	-1.89516	-25086.6	1.191186	-25033.5	5.113332	-24979.6	9.788447	-24921.5	18.80469	-24872.2	18.89386	-24847.1	19.40028
-25137.3	-0.43379	-25085.9	1.854532	-25032.8	5.777772	-24977.9	11.47826	-24928	12.29187	-24875.5	15.59069	-24847.1	19.42315
-25138.5	-1.60741	-25084.3	3.461983	-25031.3	7.241795	-24978.8	10.59547	-24925.4	14.83503	-24877.9	13.22707	-24847.9	18.67968
-25139.5	-2.60547	-25086.4	1.345645	-25033.3	5.242169	-24980.7	8.689011	-24928.2	12.03436	-24874.3	16.81878	-24846.6	19.90096
-25137.7	-0.80478	-25084.3	3.483992	-25031.3	7.299082	-24978.1	11.29484	-24928.9	11.41636	-24871.5	19.5899	-24843	23.52203
-25137.1	-0.23512	-25084.7	3.007056	-25031.1	7.508484	-24979.1	10.28788	-24924.8	15.42429	-24870.6	20.51003	-24845.1	21.47493
-25138.2	-1.31844	-25083.7	4.043702	-25032.5	6.102973	-24982.6	6.858986	-24926.6	13.63652	-24873.1	17.98634	-24847.2	19.32603
-25137.8	-0.86039	-25085.8	1.915888	-25033.5	5.1232	-24980.1	9.336904	-24924.2	16.05785	-24870.6	20.49713	-24844.9	21.62012
-25138.2	-1.26446	-25084.4	3.316251	-25032.3	6.246804	-24978.5	10.90847	-24928.1	12.21817	-24872.3	18.81905	-24850.9	15.58913
-25138.7	-1.7855	-25085	2.745475	-25030.1	8.449415	-24980	9.38794	-24927.4	12.84644	-24876.1	15.03702	-24846.8	19.7266
-25138	-1.13008	-25084.8	2.945912	-25034	4.568402	-24974.5	14.94276	-24924.4	15.89634	-24874.8	16.26984	-24847.9	18.64399
-25137.8	-0.91997	-25082.5	5.207459	-25033	5.592534	-24977	12.45635	-24924.1	16.18855	-24873.6	17.51152	-24845.7	20.82843
-25136.2	0.72004	-25083.9	3.857213	-25031.2	7.335858	-24978.5	10.97484	-24925.6	14.69727	-24874.2	16.90106	-24847.1	19.38525
-25138.1	-1.23488	-25084.8	2.896932	-25035	3.613801	-24979.2	10.21248	-24925.3	14.94332	-24873.2	17.94122	-24845.6	20.9228
-25138.4	-1.48107	-25086.7	1.003775	-25032.1	6.532204	-24978.1	11.36266	-24922.3	17.99447	-24873.7	17.40313	-24847.9	18.62386
-25137.2	-0.26292	-25086.8	0.950515	-25031.8	6.835034	-24977.6	11.8244	-24922.4	17.84544	-24877.5	13.57005	-24847.8	18.7391

-25137.9	-0.98343	-25084.3	3.437211	-25034.4	4.186811	-24979.3	10.14625	-24923.9	16.37761	-24869.8	21.29234	-24848.4	18.12998
-25138.1	-1.23371	-25033.2	54.55215	-25031.3	7.276765	-24976.3	13.1614	-24926.3	13.95336	-24872.6	18.53803	-24842.4	24.11884
-25137.7	-0.77508	-25084	3.787541	-25031.9	6.676284	-24980.3	9.08367	-24873.8	66.41959	-24874.2	16.95631	-24847.3	19.20145
-25139.3	-2.42391	-25083.8	3.901077	-25031.4	7.182285	-24979.2	10.2421	-24926	14.31064	-24876.7	14.36659	-24852.2	14.29233
-25137.4	-0.50581	-25084.4	3.310631	-25032.4	6.21205	-24922.9	66.54113	-24924.9	15.35181	-24872.4	18.74329	-24847.2	19.30481
-25138.8	-1.90218	-25083.4	4.318596	-24976.4	62.19909	-24977.2	12.23237	-24925.8	14.43565	-24846.7	44.44179	-24847.5	18.99015
-25137.1	-0.16111	-25086.5	1.207562	-25073	-34.4048	-24979.2	10.2552	-24925.9	14.37488	-24874.8	16.33262	-24841.7	24.86472
-25137.9	-1.01115	-25084.5	3.231307	-25034.3	4.274382	-24979.6	9.798493	-24927.3	12.94588	-24875.3	15.78047	-24846.1	20.41156
-25137.8	-0.87675	-25084.8	2.920401	-25030.3	8.237503	-24981.2	8.178424	-24925.2	15.11427	-24871.4	19.69124	-24843.2	23.34352
-25138.5	-1.55791	-25085.4	2.321565	-25032.6	6.018866	-24981.5	7.926759	-24928.5	11.76864	-24872.3	18.80324	-24841.8	24.77979
-25139	-2.05906	-25084.9	2.876464	-25031.1	7.522364	-24979.2	10.23799	-24929.8	10.4854	-24875.1	16.0166	-24844.2	22.36736
-25138.4	-1.4636	-25086.4	1.347178	-25031.5	7.111103	-24978.3	11.1304	-24924.2	16.11256	-24872.8	18.30198	-24849.4	17.17926
-25138.3	-1.35883	-25083.8	3.903665	-25033	5.581561	-24978.8	10.64304	-24925.4	14.87269	-24874.6	16.54436	-24847.7	18.80057
-25138.3	-1.38862	-25084.7	3.035473	-25031.9	6.722539	-24979.4	10.0003	-24923.9	16.4176	-24876	15.08609	-24849	17.51045
-25137.2	-0.2908	-25084.8	2.985454	-25031.9	6.73067	-24979.3	10.176	-24926.2	14.07362	-24873.6	17.50915	-24845.6	20.95394
-25138.4	-1.45807	-25081.5	6.241095	-25029.1	9.459222	-24977.9	11.50618	-24922.7	17.52923	-24876.2	14.93883	-24843.3	23.27697
-25137.2	-0.29305	-25084.1	3.653945	-25031.4	7.207382	-24980.1	9.327006	-24926.3	13.99058	-24870.1	21.03545	-24850.2	16.30964
-25138.1	-1.16518	-25084.8	2.9376	-25029.1	9.440866	-24979.3	10.09707	-24925.5	14.74221	-24873.1	18.04993	-24842.5	24.03622
-25136.6	0.259118	-25086.1	1.694512	-25033.1	5.438172	-24977.8	11.5914	-24926.9	13.33546	-24871.5	19.56623	-24847.2	19.32543
-25138.6	-1.69055	-25085.4	2.334567	-25030.7	7.873754	-24974.8	14.58375	-24926.9	13.36262	-24874.4	16.71532	-24844.6	21.96615
-25138.5	-1.56297	-25084	3.763122	-25032.1	6.504636	-24980.1	9.338427	-24922.9	17.40258	-24871.2	19.87625	-24848.7	17.83226
-25138.2	-1.28986	-25085.8	1.898735	-25031.5	7.12924	-24980.2	9.223611	-24929.1	11.17502	-24875.7	15.40337	-24846.9	19.60651
-25138	-1.09026	-25083.7	4.076672	-25030.7	7.911577	-24976.8	12.67407	-24927.6	12.69993	-24874.9	16.20758	-24850.5	15.98876
-25140.1	-3.21073	-25086.2	1.526335	-25032.2	6.359068	-24980.2	9.259035	-24926.5	13.75675	-24875.9	15.20084	-24846.9	19.63785
-25138.7	-1.81088	-25084.2	3.536444	-25034.3	4.245241	-24981.2	8.214632	-24925.8	14.47771	-24875.2	15.909	-24888.5	-21.9439
-25137.5	-0.57018	-25086.6	1.169941	-25024.4	14.18864	-24981.6	7.865008	-24927.1	13.16304	-24875.2	15.94821	-24842.4	24.14547
-25138.6	-1.70694	-25085.8	1.966214	-25031.6	6.975629	-24977.2	12.24102	-24927.8	12.45398	-24871.9	19.22359	-24843.8	22.73694
-25137.5	-0.63783	-25081.8	5.894988	-25032.2	6.380701	-24978.6	10.7893	-24925.9	14.36058	-24873	18.10973	-24847.4	19.16059
-25136.9	0.049619	-25084.3	3.42681	-25031.4	7.146465	-24979	10.3927	-24872.8	67.44071	-24873.1	18.01756	-24846.1	20.46645
-25138	-1.11285	-25085.7	2.010848	-25029.7	8.867132	-24980.3	9.151315	-24925	15.22385	-24872.7	18.44735	-24847.2	19.28597
-25138	-1.14125	-25080.7	7.023635	-25032.4	6.180452	-24978.6	10.82689	-24929.8	10.51331	-24872.3	18.81351	-24848.6	17.95217
-25138.6	-1.7216	-25085.3	2.467218	-25032.2	6.351311	-24979.2	10.23171	-24927.9	12.41104	-24877.8	13.33075	-24850.1	16.44166
-25138.6	-1.67037	-25085.6	2.180317	-25030.7	7.899561	-24978.3	11.08076	-24923.9	16.35163	-24916.1	-25.001	-24848.9	17.61138
-25138	-1.1193	-25086.4	1.337509	-25031	7.5934	-24979.3	10.16306	-24924.2	16.04266	-24849.1	41.97445	-24842.8	23.70381
-25138.1	-1.16012	-25086.9	0.844695	-25030.1	8.48789	-24980.1	9.290821	-24927.2	13.10643	-24916.4	-25.2446	-24847.3	19.24318
-25137.9	-0.9959	-25084.4	3.381186	-25032.2	6.360908	-24980.3	9.106331	-24927.9	12.35966	-24875.1	16.0342	-24849.5	17.0711
-25138.2	-1.29159	-25085.1	2.670382	-25031.8	6.792893	-24979.2	10.2332	-24926	14.22004	-24870.8	20.30048	-24845	21.56563
-25137.8	-0.87855	-25085	2.731867	-25031.2	7.345748	-24979	10.40114	-24926.3	13.99683	-24874.1	16.97059	-24848	18.52798
-25137.9	-0.96013	-25083.4	4.379365	-25031.9	6.69458	-24976.5	12.91321	-24925.7	14.58623	-24871.7	19.39563	-24848.9	17.66546
-25138.4	-1.48767	-25086.5	1.231479	-25032.1	6.443911	-24979.1	10.35046	-24928.3	11.98355	-24873.4	17.69778	-24846.7	19.87251
-25138	-1.06975	-25083.1	4.60114	-25032.7	5.854607	-24981.5	7.917755	-24926.7	13.56938	-24871.5	19.59256	-24847.3	19.18668

	-25137.3	-0.37925	-25085.1	2.646546	-25031.7	6.931754	-24977.9	11.5725	-24926.3	13.97111	-24875.5	15.56105	-24849.9	16.66598
	-25137.4	-0.48841	-25086.1	1.609699	-25033.3	5.265933	-24975.8	13.65017	-24917.8	22.44354	-24877.7	13.36936	-24847.3	19.27777
	-25137.9	-1.03019	-25084.7	3.081305	-25031.9	6.674172	-24979.6	9.863057	-24922.7	17.55126	-24876.2	14.8734		
	-25136.4	0.466204	-25086.1	1.639016	-25022.5	16.04458	-24979	10.47698	-24872.7	67.53232	-24874.8	16.31047		
	-25137.1	-0.16838	-25085.8	1.981806	-25033.9	4.706258	-24978.4	11.05093	-24929	11.24274	-24872.1	19.0058		
	-25138.1	-1.23713	-25087	0.77157	-25030.4	8.20114	-24975.8	13.61132	-24927.6	12.70148	-24868.8	22.28352		
	-25139.2	-2.27474	-25085.7	2.047501	-25033.2	5.426602	-24925.4	64.0715	-24923.2	17.11339	-24869.6	21.46737		
	-25138.1	-1.19664	-25085.4	2.384249	-24980.4	58.23309	-24979.7	9.740978			-24876.2	14.9528		
	-25137.6	-0.71182	-25085.8	1.916233	-25030.7	7.908742	-24978.9	10.49939			-24872.1	18.98183		
	-25138.6	-1.67673			-25030.7	7.912793	-24979.9	9.559509			-24847.5	43.59989		
	-25139.3	-2.38445			-25032.2	6.394942	-24978.7	10.73514			-24874.1	17.00334		
	-25136.5	0.415469									-24874.9	16.22927		
	-25140.3	-3.40015									-24873.6	17.49934		
	-25138.7	-1.79426									-24873.8	17.28945		
Average energies	-25138.08	-1.17	-25084.48	3.26	-25030.63	7.96	-24977.08	12.35	-24923.42	16.85	-24873.14	17.97	-24847.02	19.51

Th

	ground state energy	solid solution energy												
concentration in solid solution (%)	3		6		9		12		15		18		21	
number of atoms	2		4		6		8		10		12		13	
	-25143.63	-1.09	-25095.81	3.20	-25044.63	10.85	-24999.81	12.14	-24947.01	21.41	-24877.51	47.38	-24884.12	19.01
	-25143.90	-1.37	-25094.88	4.12	-25048.55	6.93	-25001.39	10.56	-24954.62	13.80	-24900.33	24.56	-24885.21	17.92
	-25142.77	-0.23	-25098.27	0.73	-25049.29	6.19	-24997.60	14.35	-24954.07	14.35	-24902.59	22.30	-24886.28	16.85
	-25143.21	-0.68	-25094.83	4.18	-25046.67	8.81	-25003.68	8.27	-24904.24	64.18	-24884.16	40.73	-24879.06	24.06
	-25144.60	-2.06	-25095.45	3.55	-25048.99	6.49	-25000.30	11.65	-24952.14	16.28	-24907.18	17.72	-24878.82	24.31
	-25143.45	-0.91	-25095.19	3.81	-25048.21	7.27	-25001.43	10.52	-24950.11	18.31	-24906.98	17.92	-24881.72	21.41
	-25143.50	-0.97	-25095.72	3.28	-25047.69	7.78	-25001.53	10.42	-24949.08	19.34	-24900.31	24.58	-24879.24	23.89
	-25143.45	-0.91	-25096.09	2.91	-25051.07	4.41	-24999.63	12.32	-24949.07	19.35	-24904.36	20.53	-24883.37	19.76
	-25143.14	-0.61	-25094.82	4.18	-25048.75	6.73	-25000.39	11.56	-24956.26	12.16	-24907.60	17.29	-24878.73	24.40
	-25144.83	-2.30	-25096.73	2.27	-25047.48	7.99	-25002.73	9.22	-24953.86	14.56	-24903.03	21.86	-24881.26	21.87
	-25144.99	-2.45	-25096.90	2.11	-25050.79	4.68	-24999.93	12.01	-24949.58	18.84	-24907.41	17.48	-24879.24	23.89
	-25142.16	0.37	-25096.58	2.42	-25048.19	7.29	-24997.04	14.91	-24948.57	19.85	-24900.44	24.45	-24883.59	19.54
	-25143.54	-1.00	-25097.30	1.70	-25047.35	8.13	-24998.99	12.96	-24945.81	22.61	-24905.32	19.58	-24882.70	20.43
	-25143.47	-0.94	-25095.02	3.98	-25047.93	7.54	-25001.15	10.80	-24950.85	17.57	-24905.31	19.59	-24882.36	20.77
	-25143.38	-0.85	-25095.20	3.81	-25048.46	7.02	-25001.31	10.64	-24953.85	14.57	-24899.79	25.10	-24880.48	22.65
	-25143.65	-1.11	-25094.80	4.21	-25048.45	7.03	-25001.47	10.48	-24955.14	13.28	-24904.25	20.64	-24882.68	20.45
	-25143.50	-0.96	-25095.38	3.62	-25046.53	8.94	-25000.82	11.13	-24953.38	15.04	-24903.08	21.81	-24882.38	20.75
	-25142.71	-0.18	-25094.74	4.27	-25048.76	6.72	-25002.70	9.25	-24953.63	14.79	-24905.72	19.18	-24883.48	19.65

-25144.18	-1.64	-25095.57	3.44	-25050.18	5.30	-25001.21	10.74	-24952.69	15.73	-24908.25	16.64	-24875.25	27.88
-25143.91	-1.37	-25094.30	4.71	-25048.53	6.94	-24999.50	12.45	-24947.08	21.34	-24905.94	18.95	-24882.79	20.34
-25144.27	-1.73	-25091.53	7.47	-25046.57	8.91	-24999.82	12.13	-24991.37	-22.95	-24907.22	17.67	-24883.17	19.95
-25143.78	-1.24	-25095.14	3.86	-25048.83	6.65	-24999.88	12.07	-24953.98	14.44	-24900.88	24.01	-24883.64	19.49
-25144.58	-2.05	-25096.05	2.96	-25050.47	5.01	-25002.39	9.55	-24953.34	15.08	-24906.00	18.89	-24884.00	19.13
-25142.55	-0.01	-25094.93	4.08	-25048.38	7.10	-25000.94	11.01	-24949.52	18.90	-24903.31	21.58	-24881.30	21.83
-25142.93	-0.40	-25094.63	4.37	-25046.80	8.68	-24995.94	16.01	-24952.45	15.97	-24901.07	23.82	-24879.35	23.78
-25142.07	0.46	-25096.68	2.32	-25049.39	6.09	-25001.18	10.77	-24951.85	16.57	-24905.54	19.36	-24881.69	21.44
-25144.06	-1.52	-25096.32	2.69	-25048.97	6.51	-24998.44	13.50	-24953.36	15.07	-24902.27	22.62	-24878.56	24.57
-25142.41	0.13	-25096.32	2.68	-25048.69	6.79	-25002.31	9.64	-24952.11	16.31	-24904.35	20.54	-24879.93	23.20
-25145.37	-2.83	-25095.65	3.36	-25049.03	6.45	-24999.13	12.81	-24951.23	17.19	-24907.26	17.63	-24884.54	18.58
-25142.62	-0.08	-25097.17	1.84	-25047.95	7.53	-25000.98	10.97	-24951.67	16.75	-24900.90	23.99	-24880.49	22.64
-25141.33	1.20	-25096.49	2.51	-25050.32	5.16	-24998.45	13.50	-24945.04	23.38	-24909.68	15.21	-24879.34	23.78
-25142.74	-0.21	-25091.87	7.13	-25046.63	8.85	-24999.82	12.13	-24949.45	18.97	-24905.32	19.57	-24880.25	22.87
-25143.44	-0.91	-25096.51	2.50	-25048.45	7.03	-25000.97	10.98	-24954.18	14.24	-24906.04	18.85	-24886.25	16.88
-25144.16	-1.62	-25095.12	3.88	-25048.64	6.84	-25003.10	8.85	-24954.04	14.38	-24906.38	18.51	-24884.47	18.66
-25141.34	1.20	-25096.14	2.87	-25049.15	6.33	-25000.38	11.57	-24952.75	15.67	-24903.26	21.63	-24882.88	20.25
-25143.28	-0.75	-25095.71	3.30	-25050.09	5.39	-24999.67	12.28	-24954.64	13.78	-24903.74	21.15	-24882.44	20.69
-25143.65	-1.11	-25094.55	4.46	-25049.99	5.48	-25001.22	10.73	-24951.84	16.58	-24903.75	21.15	-24884.49	18.64
-25144.88	-2.35	-25094.37	4.63	-25048.39	7.09	-24999.32	12.63	-24953.50	14.92	-24902.68	22.21	-24883.64	19.48
-25144.30	-1.77	-25096.74	2.27	-25050.24	5.23	-25000.11	11.84	-24951.47	16.95	-24902.16	22.73	-24882.07	21.06
-25143.31	-0.77	-25099.12	-0.12	-25049.32	6.16	-24998.31	13.63	-24949.75	18.67	-24906.09	18.80	-24870.71	32.42
-25142.33	0.20	-25094.02	4.99	-25047.58	7.90	-24999.02	12.93	-24953.87	14.55	-24901.16	23.73	-24874.85	28.27
-25143.85	-1.32	-25096.71	2.29	-25047.75	7.73	-24996.73	15.22	-24955.22	13.20	-24906.44	18.45	-24878.36	24.77
-25145.61	-3.08	-25095.87	3.13	-25045.60	9.88	-25001.79	10.16	-24952.86	15.56	-24903.33	21.56	-24882.20	20.93
-25143.37	-0.84	-25096.66	2.35	-25046.62	8.85	-25004.41	7.54	-24953.53	14.89	-24905.22	19.67	-24880.69	22.43
-25143.32	-0.79	-25095.19	3.82	-25046.98	8.50	-24998.87	13.08	-24952.18	16.24	-24901.23	23.66	-24882.95	20.18
-25143.91	-1.37	-25096.04	2.97	-25049.25	6.23	-24998.08	13.87	-24953.05	15.37	-24902.69	22.20	-24877.67	25.45
-25144.79	-2.25	-25094.96	4.05	-25045.60	9.88	-24954.22	57.73	-24951.85	16.57	-24904.69	20.20	-24883.65	19.48
-25144.12	-1.58	-25094.77	4.24	-25049.04	6.44	-24999.67	12.28	-24953.27	15.15	-24890.94	33.95	-24881.48	21.65
-25143.90	-1.36	-25092.99	6.02	-25045.88	9.60	-25000.38	11.56	-24949.75	18.67	-24878.44	46.45	-24883.55	19.58
-25145.05	-2.51	-25097.67	1.34	-24998.45	57.03	-24999.65	12.30	-24954.69	13.73	-24905.55	19.34	-24880.26	22.86
-25144.01	-1.48	-25047.74	51.27	-25047.94	7.54	-24998.67	13.28	-24905.69	62.73	-24900.86	24.03	-24880.93	22.20
-25144.70	-2.17	-25092.66	6.35	-25049.27	6.21	-25001.58	10.37	-24951.65	16.77	-24903.48	21.41	-24875.49	27.64
-25144.44	-1.90	-25096.23	2.77	-25048.74	6.74	-25001.02	10.92	-24953.92	14.50	-24905.93	18.96	-24882.62	20.50
-25144.10	-1.57	-25095.68	3.32	-25047.33	8.15	-24997.41	14.54	-24947.44	20.98	-24906.70	18.20	-24883.92	19.21
-25143.34	-0.80	-25095.53	3.47	-25042.84	12.63	-24995.72	16.23	-24953.93	14.49	-24906.43	18.46	-24873.53	29.60
-25143.84	-1.30	-25096.52	2.48	-25047.77	7.71	-24998.35	13.60	-24954.13	14.29	-24905.26	19.64	-24880.27	22.85
-25142.54	-0.01	-25094.07	4.93	-25047.72	7.76	-25000.99	10.96	-24949.81	18.61	-24902.13	22.76	-24880.97	22.15
-25143.98	-1.44	-25096.44	2.57	-25049.60	5.88	-25000.70	11.25	-24953.11	15.31	-24907.71	17.18	-24876.62	26.51
-25142.79	-0.26	-25096.23	2.78	-25045.49	9.99	-24998.32	13.63	-24955.50	12.92	-24903.25	21.65	-24875.37	27.76

-25143.71	-1.18	-25093.39	5.62	-25045.35	10.12	-25000.23	11.72	-24954.56	13.86	-24905.94	18.95	-24880.27	22.85
-25143.70	-1.17	-25096.28	2.73	-25049.87	5.61	-25001.16	10.78	-24953.64	14.78	-24903.23	21.66	-24878.87	24.26
-25143.99	-1.46	-25095.52	3.49	-25047.69	7.79	-24999.85	12.10	-24953.30	15.12	-24901.97	22.92	-24873.83	29.30
-25143.56	-1.02	-25096.82	2.18	-25049.46	6.01	-24998.26	13.68	-24946.98	21.44	-24896.55	28.34	-24884.11	19.02
-25143.18	-0.65	-25095.99	3.01	-25047.24	8.23	-24999.25	12.70	-24953.31	15.11	-24903.27	21.62	-24880.20	22.93
-25143.82	-1.28	-25096.81	2.19	-25048.16	7.32	-24997.17	14.78	-24952.95	15.47	-24898.93	25.96	-24882.29	20.84
-25142.62	-0.09	-25095.35	3.66	-25048.05	7.43	-25001.20	10.75	-24953.00	15.42	-24907.02	17.88	-24879.76	23.37
-25141.90	0.63	-25094.75	4.26	-25049.10	6.38	-24999.18	12.77	-24950.93	17.49	-24905.55	19.34	-24880.65	22.48
-25142.62	-0.09	-25097.04	1.97	-25047.76	7.72	-24998.33	13.62	-24955.06	13.36	-24901.60	23.29	-24878.75	24.38
-25144.26	-1.73	-25095.50	3.50	-25047.37	8.11	-25000.90	11.05	-24953.94	14.48	-24906.03	18.86	-24884.05	19.08
-25144.59	-2.06	-25095.03	3.98	-25043.21	12.27	-25000.22	11.73	-24952.72	15.70	-24903.00	21.89	-24881.24	21.89
-25144.47	-1.94	-25096.87	2.14	-25048.53	6.94	-24997.35	14.60	-24953.57	14.85	-24904.98	19.91	-24881.03	22.09
-25143.66	-1.12	-25095.09	3.91	-25049.02	6.46	-24996.34	15.61	-24953.06	15.36	-24884.72	40.18	-24875.80	27.33
-25144.85	-2.31	-25093.44	5.57	-25046.45	9.02	-25002.38	9.57	-24952.75	15.67	-24904.86	20.03	-24880.73	22.40
-25143.43	-0.89	-25097.40	1.60	-25041.43	14.05	-25003.57	8.38	-24903.37	65.05	-24904.43	20.46	-24871.20	31.92
-25144.01	-1.47	-25094.49	4.51	-25046.13	9.35	-24999.62	12.33	-24948.58	19.85	-24908.55	16.34	-24881.34	21.79
-25142.16	0.38	-25095.40	3.60	-25049.06	6.41	-25002.97	8.98	-24954.14	14.29	-24904.84	20.05	-24877.15	25.98
-25138.66	3.87	-25094.80	4.21	-25050.02	5.45	-24998.15	13.80	-24953.53	14.89	-24905.78	19.11	-24881.70	21.43
-25143.71	-1.18	-25093.52	5.48	-25049.90	5.57	-25003.65	8.29	-24949.61	18.81	-24899.98	24.92	-24878.25	24.87
-25142.07	0.47	-25094.71	4.29	-25048.37	7.10	-24995.40	16.55	-24950.87	17.55	-24906.98	17.92	-24876.73	26.40
-25143.76	-1.23	-25096.73	2.28	-25047.24	8.24	-24948.90	63.05	-24954.80	13.62	-24902.91	21.98	-24882.20	20.92
-25144.67	-2.14	-25095.99	3.01	-25048.60	6.88	-25001.32	10.63	-24954.09	14.33	-24907.73	17.16	-24881.25	21.88
-25143.70	-1.17	-25094.80	4.20	-25047.79	7.69	-25002.41	9.54	-24951.51	16.91	-24903.63	21.27	-24879.94	23.19
-25143.45	-0.91	-25095.62	3.38	-25049.45	6.03	-24955.00	56.95	-24954.85	13.57	-24884.15	40.74	-24880.61	22.52
-25143.21	-0.67	-25096.79	2.21	-25049.03	6.44	-24999.03	12.92	-24950.20	18.22	-24899.95	24.94	-24875.16	27.97
-25143.24	-0.71	-25096.03	2.98	-25048.89	6.59	-25001.10	10.85	-24951.26	17.16	-24881.15	43.74	-24881.94	21.19
-25144.09	-1.56	-25095.75	3.26	-24999.73	55.75	-24998.24	13.71	-24903.53	64.89	-24905.44	19.45	-24876.66	26.47
-25144.44	-1.90	-25098.10	0.91	-25049.08	6.40	-25002.62	9.33	-24954.80	13.62	-24903.75	21.14	-24874.76	28.36
-25142.88	-0.35	-25095.58	3.42	-25047.02	8.45	-25000.75	11.20	-24945.49	22.93	-24875.28	49.61	-24882.18	20.95
-25143.56	-1.03	-25046.58	52.43	-25045.70	9.77	-25000.16	11.79	-24904.29	64.13	-24903.44	21.45	-24884.05	19.08
-25143.83	-1.29	-25095.42	3.58	-25043.71	11.77	-25000.29	11.66	-24945.79	22.63	-24905.01	19.88	-24875.87	27.26
-25143.05	-0.52	-25097.47	1.54	-25044.69	10.79	-24999.96	11.99	-24948.28	20.14	-24904.03	20.86	-24880.09	23.03
-25143.13	-0.59	-25097.44	1.57	-25049.21	6.26	-24999.67	12.28	-24949.70	18.72	-24894.66	30.23	-24884.53	18.60
-25143.28	-0.75	-25096.90	2.10	-25047.08	8.40	-25000.08	11.87	-24947.89	20.53	-24908.17	16.73	-24877.73	25.39
-25145.40	-2.87	-25094.68	4.33	-25047.77	7.71	-24998.54	13.41	-24950.76	17.66	-24906.28	18.61	-24879.45	23.67
-25144.69	-2.16	-25096.20	2.81	-25042.08	13.40	-25000.87	11.08	-24951.07	17.35	-24904.54	20.35	-24880.64	22.49
-25143.61	-1.07	-25095.79	3.21	-25047.07	8.41	-24997.45	14.50	-24954.19	14.23	-24904.43	20.46	-24874.75	28.38
-25143.59	-1.05	-25096.67	2.34	-25050.23	5.25	-25000.13	11.82	-24953.44	14.98	-24906.92	17.97	-24884.63	18.50
-25144.01	-1.47	-25095.16	3.85	-25048.00	7.48	-24999.60	12.35	-24944.49	23.94	-24907.25	17.64	-24876.25	26.87
-25143.24	-0.71	-25094.61	4.39	-25046.79	8.69	-25002.30	9.65	-24948.45	19.97	-24907.30	17.59	-24877.09	26.04
-25145.70	-3.17	-25091.63	7.38	-25047.93	7.54	-24998.61	13.34	-24949.00	19.42	-24901.77	23.12	-24879.26	23.87

	-25143.24	-0.70	-25095.54	3.47	-25045.27	10.21	-24999.62	12.33	-24952.79	15.63	-24908.74	16.15	-24881.81	21.32
	-25144.80	-2.27	-25096.05	2.96	-25049.11	6.37	-25000.60	11.35	-24954.20	14.22	-24905.55	19.35	-24880.06	23.07
	-25144.11	-1.58	-25096.19	2.82	-25047.41	8.06	-25000.24	11.70	-24951.18	17.24	-24908.39	16.50	-24877.62	25.50
	-25142.41	0.12	-25094.67	4.34	-25049.82	5.66	-24998.77	13.18	-24953.30	15.12	-24901.89	23.00	-24883.00	20.12
	-25143.76	-1.23	-25095.37	3.63	-25046.65	8.83	-24999.91	12.04	-24952.57	15.85	-24900.45	24.44	-24883.62	19.51
	-25143.16	-0.62	-25096.89	2.12	-25050.44	5.04	-24995.29	16.65	-24955.87	12.55	-24900.15	24.75	-24882.53	20.60
	-25144.64	-2.11	-25095.87	3.14	-25046.03	9.45	-25002.49	9.46	-24952.61	15.81	-24904.83	20.06		
	-25143.10	-0.57	-25097.43	1.57	-25047.97	7.51	-25002.60	9.35	-24947.32	21.10	-24903.14	21.75		
	-25143.57	-1.03	-25096.09	2.91	-25045.28	10.20	-25001.20	10.75	-24954.39	14.03	-24908.26	16.63		
	-25143.41	-0.87	-25096.17	2.84	-25046.17	9.31	-24998.56	13.39	-24950.97	17.45	-24901.05	23.85		
	-25143.38	-0.85	-25096.21	2.80	-25051.72	3.75	-24993.85	18.10	-24955.06	13.36	-24903.71	21.18		
	-25143.87	-1.34	-25094.58	4.42	-25048.54	6.94	-24999.53	12.42	-24994.92	-26.50	-24904.96	19.93		
	-25143.58	-1.04	-25094.70	4.31	-25048.77	6.71			-24952.61	15.81	-24903.20	21.70		
	-25143.99	-1.46	-25094.80	4.21	-25048.75	6.73			-24954.33	14.09	-24901.69	23.20		
	-25144.13	-1.60	-25096.21	2.80	-25048.15	7.33			-24955.38	13.04	-24906.70	18.19		
	-25143.63	-1.10	-25096.27	2.73	-25049.32	6.15			-24952.15	16.28				
	-25142.47	0.07	-25094.20	4.80										
	-25144.08	-1.55	-25095.04	3.97										
	-25143.60	-1.07	-25096.46	2.55										
Average energies	-25143.59	-1.06	-25094.80	4.20	-25047.08	8.40	-24998.71	13.24	-24950.62	17.80	-24902.68	22.22	-24880.44	22.68

U

	ground state energy	solid solution energy												
concentration in solid solution (%)	3		6		9		12		15		18		21	
number of atoms	2		4		6		8		10		12		13	
	-25148.21	-0.66	-25107.62	1.42	-25064.06	6.48	-25021.62	10.40	-24977.94	15.58	-24937.78	17.22	-24911.71	24.04
	-25139.66	7.89	-25106.66	2.38	-25064.04	6.49	-25020.84	11.19	-24977.43	16.08	-24936.69	18.32	-24915.49	20.26
	-25147.57	-0.02	-25095.10	13.95	-25060.52	10.01	-25023.13	8.89	-24978.46	15.06	-24931.99	23.02	-24906.65	29.10
	-25139.46	8.10	-25105.01	4.04	-25063.61	6.92	-25016.72	15.30	-24977.06	16.45	-24939.38	15.62	-24918.48	17.27
	-25148.27	-0.72	-25105.31	3.73	-25062.11	8.42	-25021.81	10.22	-24971.46	22.05	-24936.80	18.20	-24916.88	18.87
	-25138.88	8.67	-25106.73	2.31	-25063.27	7.27	-25021.70	10.32	-24977.81	15.70	-24938.05	16.95	-24915.70	20.05
	-25148.69	-1.14	-25106.07	2.98	-25061.32	9.21	-25020.43	11.59	-24978.99	14.52	-24935.67	19.34	-24908.91	26.84
	-25148.81	-1.26	-25105.53	3.51	-25061.39	9.14	-25020.76	11.27	-24974.43	19.09	-24934.79	20.21	-24916.01	19.74
	-25147.70	-0.15	-25105.06	3.98	-25058.55	11.98	-25019.47	12.55	-24978.80	14.71	-24935.98	19.03	-24915.54	20.21
	-25147.15	0.40	-25106.04	3.00	-25059.25	11.28	-25023.30	8.72	-24980.94	12.57	-24935.56	19.44	-24916.08	19.67
	-25147.15	0.40	-25105.87	3.17	-25059.08	11.45	-25021.90	10.13	-24979.60	13.92	-24938.71	16.29	-24914.40	21.35
	-25146.33	1.22	-25106.18	2.87	-25061.02	9.51	-25020.36	11.66	-24977.29	16.22	-24934.10	20.90	-24916.18	19.57

-25140.20	7.35	-25107.55	1.49	-25064.65	5.88	-25020.52	11.50	-24976.82	16.69	-24937.42	17.58	-24912.11	23.64
-25148.64	-1.09	-25103.46	5.58	-25064.88	5.65	-25014.85	17.17	-24976.72	16.79	-24938.44	16.56	-24910.47	25.28
-25148.17	-0.62	-25104.52	4.52	-25064.51	6.02	-25016.78	15.25	-24978.20	15.31	-24937.03	17.98	-24915.96	19.79
-25147.14	0.41	-25105.51	3.54	-25059.66	10.87	-25007.63	24.39	-24979.49	14.02	-24932.49	22.51	-24913.46	22.29
-25147.77	-0.21	-25097.77	11.27	-25062.65	7.88	-25017.67	14.35	-24980.85	12.67	-24933.69	21.31	-24912.05	23.70
-25148.16	-0.61	-25103.95	5.09	-25061.85	8.68	-25020.11	11.91	-24977.70	15.82	-24937.19	17.81	-24910.60	25.15
-25149.09	-1.54	-25107.83	1.21	-25061.95	8.58	-25020.18	11.84	-24977.84	15.68	-24934.27	20.73	-24918.83	16.92
-25139.66	7.89	-25105.05	4.00	-25064.53	6.00	-25023.39	8.63	-24978.61	14.91	-24936.34	18.67	-24915.72	20.03
-25148.06	-0.50	-25105.10	3.95	-25064.62	5.91	-25018.33	13.69	-24975.98	17.54	-24934.96	20.04	-24913.83	21.92
-25148.02	-0.46	-25105.28	3.77	-25062.68	7.85	-25019.19	12.84	-24972.46	21.06	-24936.93	18.08	-24909.79	25.96
-25145.67	1.88	-25104.42	4.62	-25061.41	9.13	-25021.11	10.92	-24979.26	14.25	-24935.26	19.74	-24916.56	19.19
-25146.15	1.40	-25106.24	2.80	-25058.07	12.47	-25020.38	11.65	-24976.40	17.11	-24933.85	21.16	-24912.33	23.42
-25145.88	1.68	-25104.79	4.26	-25063.92	6.61	-25020.80	11.23	-24980.07	13.44	-24938.25	16.75	-24917.55	18.20
-25148.95	-1.40	-25105.82	3.22	-25065.18	5.35	-25019.11	12.91	-24975.51	18.01	-24931.49	23.51	-24911.40	24.35
-25148.23	-0.68	-25105.78	3.26	-25063.50	7.03	-25018.00	14.02	-24976.82	16.69	-24935.98	19.02	-24913.01	22.74
-25148.18	-0.63	-25104.54	4.50	-25061.59	8.95	-25022.89	9.13	-24973.80	19.71	-24992.54	-37.54	-24914.60	21.15
-25147.08	0.47	-25105.82	3.23	-25065.33	5.20	-25021.40	10.62	-24980.52	12.99	-24935.61	19.39	-24906.98	28.77
-25147.28	0.27	-25106.58	2.46	-25062.81	7.72	-25021.72	10.30	-24976.69	16.83	-24934.19	20.81	-24910.89	24.86
-25147.39	0.16	-25104.56	4.49	-25061.76	8.77	-25020.12	11.91	-24979.18	14.34	-24930.88	24.12	-24915.48	20.27
-25148.10	-0.55	-25104.64	4.41	-25063.47	7.06	-25020.06	11.97	-24976.48	17.03	-24934.33	20.68	-24912.52	23.23
-25146.73	0.83	-25105.83	3.21	-25058.93	11.60	-25021.26	10.77	-24977.08	16.43	-24931.24	23.76	-24913.43	22.32
-25148.64	-1.09	-25105.50	3.55	-25061.63	8.90	-25017.38	14.65	-24975.60	17.91	-24927.79	27.21	-24917.09	18.66
-25148.55	-0.99	-25103.27	5.77	-25063.62	6.91	-25021.38	10.64	-24979.42	14.10	-24939.80	15.21	-24913.50	22.25
-25146.88	0.68	-25101.06	7.98	-25060.24	10.29	-25016.61	15.41	-24970.61	22.90	-24936.39	18.62	-24915.43	20.32
-25147.86	-0.31	-25104.00	5.04	-25064.03	6.51	-25023.10	8.93	-24975.61	17.91	-24931.74	23.27	-24910.80	24.95
-25147.96	-0.40	-25107.42	1.63	-25060.87	9.67	-25011.45	20.58	-24976.27	17.25	-24932.89	22.12	-24911.49	24.26
-25147.78	-0.23	-25104.10	4.94	-25063.76	6.77	-25018.85	13.17	-24972.81	20.70	-24931.31	23.70	-24915.25	20.50
-25148.87	-1.32	-25102.63	6.41	-25065.07	5.46	-25021.02	11.00	-24980.43	13.08	-24933.54	21.46	-24912.78	22.97
-25147.46	0.09	-25101.77	7.27	-25065.75	4.78	-25018.09	13.93	-24978.62	14.89	-24935.81	19.19	-24913.20	22.55
-25147.72	-0.17	-25106.67	2.37	-25063.12	7.41	-25020.99	11.03	-24980.22	13.30	-24934.54	20.46	-24914.81	20.94
-25144.95	2.60	-25106.03	3.02	-25061.83	8.71	-25014.45	17.58	-24974.54	18.97	-24930.27	24.74	-24915.71	20.04
-25145.42	2.13	-25104.07	4.98	-25061.58	8.96	-25021.55	10.48	-24980.65	12.87	-24933.74	21.26	-24913.19	22.56
-25147.58	-0.03	-25124.42	-15.37	-25061.32	9.22	-25020.50	11.52	-24978.32	15.20	-24937.25	17.75	-24912.08	23.67
-25148.12	-0.56	-25102.25	6.79	-25063.70	6.84	-25017.61	14.41	-24977.91	15.60	-24935.92	19.08	-24914.88	20.87
-25148.94	-1.39	-25104.45	4.60	-25050.04	20.50	-25020.76	11.26	-24978.98	14.53	-24935.23	19.78	-24913.73	22.02
-25149.49	-1.94	-25103.65	5.39	-25060.40	10.14	-25022.95	9.07	-24979.80	13.72	-24938.02	16.99	-24910.65	25.09
-25147.16	0.40	-25105.08	3.96	-25060.73	9.81	-25021.47	10.55	-24977.70	15.82	-24933.76	21.24	-24914.46	21.29
-25148.10	-0.55	-25102.77	6.27	-25061.51	9.02	-25019.63	12.40	-24979.65	13.86	-24934.73	20.27	-24916.74	19.01
-25146.75	0.80	-25105.46	3.58	-25059.07	11.47	-25018.17	13.85	-24974.31	19.20	-24937.52	17.48	-24906.30	29.45
-25139.68	7.88	-25103.95	5.09	-25063.66	6.87	-25018.97	13.05	-24980.07	13.45	-24935.49	19.52	-24915.38	20.37
-25147.50	0.06	-25104.48	4.56	-25063.15	7.39	-25022.84	9.18	-24971.39	22.12	-24936.34	18.66	-24916.47	19.28

	-25148.37	-0.81	-25106.56	2.49	-25063.33	7.20	-25020.42	11.61	-24975.58	17.93	-24935.77	19.24	-24912.88	22.87
	-25147.95	-0.40	-25106.13	2.92	-25064.11	6.42	-25021.43	10.59	-24971.91	21.61	-24938.54	16.47	-24915.81	19.94
	-25147.44	0.11	-25105.86	3.19	-25062.63	7.90	-25011.47	20.55	-24975.12	18.39	-24937.69	17.31	-24915.08	20.67
	-25147.95	-0.39	-25106.56	2.48	-25065.61	4.92	-25020.56	11.46	-24977.40	16.12	-24936.54	18.46	-24915.23	20.52
	-25148.28	-0.73	-25105.43	3.61	-25063.99	6.54	-25018.67	13.36	-24981.11	12.40	-24937.98	17.02	-24914.22	21.53
	-25147.44	0.11	-25106.54	2.50	-25061.68	8.85	-25020.53	11.49	-24972.51	21.00	-24934.44	20.57	-24915.47	20.28
	-25147.40	0.15	-25104.49	4.55	-25062.66	7.87	-25018.45	13.58	-24981.43	12.09	-24931.24	23.76	-24914.60	21.15
	-25148.94	-1.39	-25104.45	4.59	-25061.30	9.23	-25021.86	10.16	-24975.98	17.54	-24933.26	21.74	-24909.27	26.48
	-25147.20	0.35	-25101.72	7.32	-25063.64	6.89	-25021.22	10.80	-24976.16	17.36	-24935.82	19.18	-24916.50	19.25
	-25148.59	-1.04	-25096.68	12.36	-25062.64	7.90	-25021.37	10.65	-24977.13	16.38	-24930.34	24.67	-24911.38	24.37
	-25148.98	-1.43	-25106.22	2.82	-25062.70	7.83	-25017.68	14.35	-24978.45	15.06	-24936.72	18.28	-24915.50	20.25
	-25147.98	-0.43	-25107.23	1.81			-25023.17	8.85	-24974.88	18.63			-24913.08	22.67
	-25148.50	-0.94	-25105.92	3.13			-25019.33	12.70	-24980.13	13.39				
	-25148.02	-0.47	-25103.30	5.74			-25019.17	12.85						
	-25148.42	-0.87	-25105.41	3.63			-25020.90	11.12						
	-25147.13	0.42	-25105.26	3.78			-25020.25	11.77						
	-25147.45	0.10	-25105.96	3.08										
	-25148.37	-0.82	-25105.78	3.26										
	-25147.60	-0.04	-25106.99	2.05										
	-25148.67	-1.11	-25105.36	3.68										
	-25147.81	-0.26	-25105.00	4.04										
	-25148.01	-0.46	-25104.79	4.25										
	-25149.78	-2.23	-25106.55	2.50										
	-25147.67	-0.12	-25105.71	3.33										
	-25147.31	0.24												
	-25148.80	-1.25												
	-25148.62	-1.07												
	-25147.77	-0.21												
	-25148.78	-1.22												
	-25147.70	-0.15												
	-25149.45	-1.90												
	-25147.02	0.54												
	-25139.13	8.42												
Average energies	-25147.17	0.39	-25105.11	3.94	-25062.27	8.26	-25019.74	12.29	-24977.23	16.28	-24936.07	18.94	-24913.73	22.02

$Ca_{1-x}M_xZrTi_{2-2x}Fe_{2x}O_7$ M=(Ce, Pu, Th, U)

Ti(2)Ti(3)

N.B. All stated energies are in eV

Ce

	ground state energy	solid solution energy													
concentration in solid solution (%)	3		6		9		12		15		18		21		
number of atoms	2		4		6		8		10		12		13		
	-25114.07	28.81	-25067.46	32.26	-25022.15	34.39	-24978.69	34.68	-24935.24	34.95	-24896.32	30.71	-24870.43	35.00	
	-25115.01	27.88	-25070.51	29.20	-25025.10	31.44	-24978.70	34.67	-24937.10	33.10	-24893.54	33.48	-24867.69	37.74	
	-25114.22	28.67	-25069.38	30.34	-25026.94	29.60	-24980.45	32.92	-24938.41	31.78	-24890.07	36.95	-24873.77	31.67	
	-25114.33	28.55	-25068.78	30.94	-25024.69	31.85	-24982.02	31.35	-24931.21	38.99	-24891.43	35.59	-24865.50	39.93	
	-25114.19	28.69	-25070.22	29.50	-25025.18	31.36	-24982.95	30.42	-24938.47	31.72	-24893.01	34.01	-24868.45	36.99	
	-25114.48	28.40	-25070.00	29.71	-25026.43	30.12	-24974.65	38.72	-24932.34	37.86	-24891.64	35.38	-24868.26	37.17	
	-25113.75	29.14	-25068.96	30.76	-25023.39	33.15	-24982.40	30.97	-24935.34	34.86	-24892.60	34.42	-24865.38	40.05	
	-25112.24	30.65	-25070.74	28.98	-25024.00	32.54	-24979.63	33.74	-24936.92	33.27	-24891.45	35.57	-24871.28	34.16	
	-25113.49	29.39	-25069.33	30.39	-25026.38	30.17	-24978.30	35.07	-24940.46	29.73	-24893.23	33.79	-24872.03	33.40	
	-25113.58	29.31	-25069.81	29.90	-25026.59	29.95	-24978.67	34.70	-24934.78	35.41	-24888.42	38.60	-24871.30	34.14	
	-25112.35	30.54	-25071.41	28.30	-25024.72	31.83	-24978.07	35.30	-24933.36	36.84	-24893.29	33.73	-24871.95	33.48	
	-25113.45	29.44	-25069.30	30.42	-25026.31	30.23	-24981.72	31.65	-24935.53	34.67	-24892.16	34.87	-24873.83	31.60	
	-25112.36	30.53	-25070.64	29.08	-25022.68	33.86	-24982.83	30.54	-24935.29	34.91	-24892.06	34.97	-24871.30	34.13	
	-25114.87	28.02	-25070.15	29.56	-25025.58	30.97	-24984.23	29.14	-24936.24	33.95	-24895.86	31.16	-24871.36	34.08	
	-25113.51	29.37	-25069.85	29.86	-25023.34	33.20	-24981.33	32.04	-24939.00	31.19	-24888.53	38.49	-24865.62	39.81	
	-25114.93	27.96	-25070.28	29.43	-25025.55	30.99	-24977.67	35.70	-24937.39	32.80	-24892.27	34.75	-24870.77	34.66	
	-25114.86	28.03	-25067.12	32.59	-25024.25	32.30	-24980.75	32.62	-24933.91	36.29	-24888.67	38.35	-24874.64	30.80	
	-25115.61	27.28	-25068.22	31.49	-25024.08	32.46	-24980.68	32.68	-24936.30	33.90	-24892.90	34.12	-24867.73	37.71	
	-25113.92	28.97	-25071.12	28.59	-25025.27	31.28	-24980.11	33.26	-24930.74	39.46	-24895.75	31.27	-24872.45	32.99	
	-25113.38	29.51	-25070.74	28.98	-25024.35	32.20	-24981.89	31.48	-24937.99	32.20	-24891.63	35.39	-24868.16	37.28	
	-25114.49	28.40	-25071.74	27.97	-25024.89	31.65	-24978.45	34.92	-24935.01	35.19	-24888.62	38.40	-24868.02	37.42	
	-25113.98	28.91	-25068.54	31.18	-25024.70	31.84	-24981.63	31.74	-24937.74	32.46	-24893.21	33.82	-24865.28	40.15	
	-25115.25	27.64	-25068.64	31.08	-25002.15	54.40	-24978.66	34.71	-24935.05	35.15	-24889.10	37.92	-24874.66	30.78	
	-25113.73	29.16	-25071.15	28.56	-25023.18	33.36	-24978.95	34.42	-24936.13	34.07	-24894.58	32.45	-24871.22	34.22	
	-25114.59	28.30	-25070.27	29.45	-25023.87	32.67	-24981.57	31.80	-24934.99	35.20	-24888.78	38.24	-24870.69	34.74	
	-25113.15	29.74	-25068.42	31.29	-25021.55	35.00	-24974.44	38.93	-24937.60	32.59	-24894.06	32.96	-24864.40	41.04	
	-25113.88	29.01	-25070.90	28.81	-25024.97	31.57	-24980.74	32.63	-24929.35	40.85	-24894.89	32.14	-24871.08	34.35	
	-25110.74	32.15	-25069.19	30.53	-25026.51	30.03	-24981.79	31.58	-24935.59	34.60	-24893.25	33.77	-24872.00	33.43	
	-25112.53	30.36	-25067.49	32.23	-25026.26	30.28	-24982.14	31.23	-24935.21	34.98	-24892.10	34.92	-24871.86	33.58	

-25114.55	28.34	-25068.50	31.21	-25024.54	32.01	-24980.01	33.36	-24932.79	37.41	-24894.48	32.55	-24868.39	37.05
-25114.84	28.05	-25070.59	29.12	-25025.45	31.09	-24982.46	30.91	-24936.58	33.61	-24890.03	36.99	-24867.91	37.53
-25114.05	28.84	-25068.39	31.33	-25022.88	33.67	-24979.70	33.67	-24936.82	33.37	-24892.67	34.35	-24865.30	40.13
-25114.35	28.53	-25069.09	30.62	-25025.15	31.40	-24979.55	33.82	-24936.04	34.16	-24890.92	36.10	-24871.12	34.31
-25113.65	29.23	-25069.10	30.61	-25023.93	32.61	-24982.31	31.06	-24930.48	39.72	-24895.35	31.67	-24870.64	34.79
-25113.43	29.46	-25068.25	31.46	-25025.22	31.32	-24977.18	36.19	-24935.30	34.89	-24892.22	34.80	-24866.10	39.34
-25114.91	27.98	-25052.45	47.27	-25025.13	31.42	-24980.00	33.37	-24932.21	37.99	-24890.22	36.81	-24873.20	32.24
-25114.32	28.56	-25070.13	29.59	-25020.38	36.16	-24980.67	32.70	-24937.43	32.77	-24894.10	32.92	-24870.07	35.37
-25114.71	28.18	-25067.57	32.15	-25025.86	30.68	-24977.61	35.75	-24938.32	31.87	-24894.96	32.07	-24871.25	34.19
-25113.80	29.09	-25065.73	33.98	-25023.72	32.82	-24979.81	33.56	-24940.81	29.39	-24889.31	37.72	-24865.59	39.84
-25114.69	28.19	-25068.64	31.08	-25025.78	30.76	-24980.12	33.25	-24936.84	33.35	-24892.12	34.90	-24870.31	35.12
-25115.56	27.33	-25071.40	28.32	-25022.51	34.03	-24974.77	38.60	-24934.91	35.29	-24896.54	30.48	-24870.48	34.95
-25110.46	32.43	-25071.42	28.29	-25024.28	32.26	-24984.22	29.15	-24936.38	33.82	-24894.49	32.54	-24870.18	35.26
-25114.66	28.23	-25070.98	28.73	-25023.72	32.82	-24982.46	30.90	-24939.99	30.20	-24892.99	34.03	-24873.98	31.46
-25114.60	28.29	-25072.06	27.66	-25027.11	29.43	-24983.58	29.79	-24938.20	32.00	-24886.74	40.28	-24865.41	40.03
-25114.05	28.83	-25068.03	31.69	-25026.96	29.58	-24981.90	31.47	-24891.97	78.22	-24889.33	37.69	-24868.96	36.47
-25113.52	29.37	-25070.74	28.97	-25024.38	32.17	-24977.92	35.45	-24930.84	39.35	-24891.72	35.31	-24870.04	35.40
-25113.35	29.54	-25069.69	30.03	-25022.83	33.71	-24981.21	32.16	-24937.19	33.00	-24896.61	30.41	-24871.59	33.85
-25113.37	29.52	-25068.85	30.87	-25027.41	29.13	-24977.28	36.09	-24939.19	31.00	-24892.35	34.67	-24874.57	30.86
-25114.18	28.71	-25070.33	29.38	-25025.53	31.02	-24980.34	33.03	-24935.33	34.87	-24886.72	40.30	-24864.15	41.28
-25114.33	28.56	-25067.29	32.43	-25026.89	29.65	-24980.30	33.07	-24931.91	38.29	-24893.67	33.35	-24864.38	41.06
-25116.05	26.84	-25070.02	29.69	-25026.63	29.91	-24983.50	29.86	-24937.61	32.58	-24890.99	36.04	-24870.65	34.78
-25115.04	27.85	-25070.66	29.06	-25021.82	34.72	-24980.50	32.86	-24937.88	32.32	-24897.56	29.46	-24870.43	35.00
-25114.41	28.48	-25069.09	30.63	-25026.48	30.06	-24980.64	32.73	-24936.14	34.05	-24890.97	36.05	-24869.24	36.20
-25115.33	27.56	-25068.81	30.90	-25025.87	30.67	-24983.96	29.41	-24937.94	32.26	-24894.33	32.69	-24872.78	32.66
-25113.82	29.07	-25068.99	30.73	-25020.93	35.61	-24982.56	30.81	-24936.70	33.49	-24892.00	35.02	-24872.82	32.61
-25111.46	31.43	-25070.72	29.00	-25023.42	33.12	-24983.72	29.65	-24940.05	30.15	-24893.53	33.49	-24870.87	34.57
-25115.13	27.76	-25070.94	28.77	-25024.44	32.10	-24979.05	34.31	-24935.57	34.63	-24891.05	35.97	-24869.06	36.38
-25112.68	30.21	-25070.38	29.33	-25023.94	32.60	-24976.04	37.33	-24935.32	34.88	-24895.03	32.00	-24872.78	32.65
-25114.18	28.71	-25069.37	30.34	-25024.86	31.69	-24979.32	34.05	-24937.22	32.97	-24886.73	40.29	-24867.95	37.49
-25114.49	28.40	-25070.25	29.46	-25025.74	30.80	-24982.23	31.14	-24936.55	33.64	-24893.73	33.29	-24870.59	34.85
-25112.57	30.32	-25070.70	29.02	-25027.66	28.88	-24981.20	32.17	-24934.66	35.54	-24895.72	31.30	-24870.43	35.01
-25111.51	31.37	-25069.44	30.28	-25022.78	33.77	-24973.23	40.14	-24934.29	35.90	-24889.71	37.31	-24862.66	42.77
-25113.52	29.37	-25063.98	35.73	-25027.67	28.87	-24980.81	32.56	-24935.97	34.22	-24892.96	34.07	-24865.82	39.61
-25114.04	28.85	-25065.47	34.24	-25026.62	29.92	-24978.66	34.71	-24936.64	33.56	-24894.91	32.12	-24872.24	33.20
-25112.11	30.78	-25069.33	30.39	-25024.89	31.65	-24981.74	31.63	-24936.43	33.76	-24890.38	36.64	-24867.86	37.57
-25113.05	29.84	-25068.63	31.08	-25021.72	34.82	-24980.78	32.59	-24936.72	33.48	-24892.13	34.89	-24873.22	32.21
-25116.13	26.76	-25069.00	30.71	-25027.03	29.52	-24981.52	31.85	-24939.22	30.97	-24893.97	33.06	-24870.96	34.48
-25113.06	29.83	-25068.10	31.61	-25025.08	31.46	-24981.68	31.69	-24935.72	34.48	-24891.78	35.24	-24866.58	38.86
-25115.28	27.61	-25071.58	28.13	-25023.19	33.35	-24981.12	32.25	-24936.08	34.11	-24889.56	37.46	-24869.66	35.77
-25114.31	28.58	-25070.65	29.06	-25028.61	27.94	-24973.58	39.79	-24933.85	36.34	-24889.23	37.79	-24872.81	32.63

-25114.16	28.73	-25071.56	28.15	-25024.01	32.53	-24981.38	31.99	-24940.70	29.50	-24885.96	41.06	-24873.66	31.77
-25113.35	29.54	-25066.98	32.74	-25025.01	31.54	-24982.48	30.89	-24940.16	30.03	-24896.32	30.70	-24873.70	31.74
-25113.93	28.96	-25069.48	30.23	-25023.86	32.69	-24975.97	37.40	-24935.32	34.88	-24890.76	36.26	-24865.72	39.72
-25114.61	28.28	-25068.96	30.76	-25026.94	29.60	-24981.83	31.54	-24934.68	35.51	-24895.74	31.28	-24865.95	39.49
-25113.01	29.88	-25071.74	27.98	-25027.21	29.33	-24977.42	35.94	-24936.95	33.24	-24896.89	30.13	-24871.38	34.06
-25115.44	27.45	-25069.94	29.77	-25023.33	33.21	-24982.36	31.01	-24936.32	33.88	-24889.68	37.34	-24871.34	34.10
-25114.50	28.39	-25070.99	28.72	-25024.58	31.96	-24981.19	32.17	-24937.74	32.46	-24895.38	31.64	-24871.42	34.01
-25112.74	30.15	-25067.17	32.55	-25026.23	30.31	-24981.46	31.91	-24936.41	33.79	-24891.78	35.25	-24873.59	31.84
-25112.54	30.35	-25071.18	28.54	-25027.31	29.23	-24979.26	34.11	-24936.48	33.72	-24886.50	40.52	-24866.68	38.76
-25105.11	37.78	-25070.67	29.05	-25023.06	33.48	-24981.75	31.62	-24937.61	32.58	-24893.66	33.36	-24867.09	38.35
-25114.32	28.57	-25070.28	29.44	-25021.48	35.06	-24980.69	32.68	-24939.86	30.33	-24895.54	31.48	-24868.67	36.77
-25114.59	28.30	-25070.32	29.39	-25023.57	32.97	-24976.98	36.39	-24934.72	35.48	-24893.67	33.35	-24870.08	35.36
-25114.63	28.26	-25067.82	31.89	-25023.91	32.63	-24984.32	29.05	-24937.41	32.78	-24894.32	32.71	-24870.58	34.86
-25114.93	27.96	-25069.07	30.64	-25021.10	35.45	-24982.15	31.22	-24936.83	33.36	-24891.76	35.27	-24871.18	34.26
-25114.41	28.48	-25067.85	31.87	-25022.94	33.60	-24981.19	32.18	-24936.61	33.58	-24894.59	32.43	-24868.84	36.60
-25112.87	30.02	-25068.25	31.46	-25024.77	31.77	-24980.70	32.67	-24936.22	33.98	-24890.43	36.60	-24870.89	34.54
-25115.31	27.57	-25068.01	31.71	-25026.75	29.79	-24981.33	32.04	-24939.23	30.96	-24893.85	33.18	-24866.80	38.63
-25113.91	28.98	-25069.96	29.76	-25024.39	32.15	-24980.71	32.66	-24934.65	35.54	-24893.95	33.08	-24871.46	33.98
-25115.28	27.61	-25069.88	29.83	-25023.52	33.02	-24978.94	34.43	-24938.64	31.55	-24891.91	35.11	-24869.65	35.78
-25114.33	28.55	-25069.76	29.95	-25026.50	30.04	-24980.05	33.32	-24938.58	31.61	-24892.72	34.30	-24866.02	39.41
-25114.50	28.39	-25069.02	30.69	-25025.36	31.18	-24984.74	28.63	-24938.65	31.54	-24891.14	35.88	-24874.03	31.41
-25115.27	27.62	-25070.06	29.66	-25024.55	31.99	-24979.06	34.31	-24926.58	43.62	-24893.26	33.76	-24868.48	36.96
-25114.24	28.64	-25072.03	27.69	-25014.92	41.62	-24981.31	32.06	-24936.42	33.77	-24890.48	36.55	-24875.51	29.92
-25114.06	28.82	-25070.33	29.39	-25025.28	31.26	-24978.59	34.78	-24935.18	35.02	-24892.76	34.26	-24866.69	38.74
-25114.32	28.57	-25069.86	29.86	-25024.55	32.00	-24974.00	39.37	-24937.33	32.87	-24891.30	35.72	-24864.73	40.71
-25113.87	29.02	-25068.34	31.38	-25025.89	30.65	-24982.73	30.64	-24936.27	33.93	-24896.73	30.29	-24867.30	38.14
-25114.23	28.66	-25070.57	29.15	-25027.13	29.41	-24981.47	31.90	-24935.13	35.06	-24891.31	35.72	-24869.99	35.45
-25113.65	29.24	-25070.76	28.95	-25027.43	29.11	-24982.87	30.50	-24936.68	33.52	-24894.15	32.87	-24867.28	38.15
-25114.86	28.02	-25069.89	29.82	-25026.39	30.15	-24975.72	37.65	-24937.67	32.52	-24895.19	31.83	-24869.01	36.43
-25114.63	28.25	-25069.69	30.03	-25027.06	29.48	-24973.01	40.35	-24941.66	28.54	-24885.93	41.10	-24870.11	35.32
-25112.64	30.25	-25069.15	30.57	-25022.92	33.62	-24982.56	30.81	-24936.26	33.93	-24893.50	33.52	-24870.94	34.50
-25114.66	28.23	-25070.96	28.76	-25024.56	31.98	-24978.54	34.83	-24936.76	33.43	-24891.05	35.98	-24875.25	30.19
-25114.59	28.30	-25071.24	28.47	-25026.71	29.84	-24979.06	34.31	-24934.96	35.24	-24889.14	37.88	-24869.09	36.35
-25111.11	31.78	-25070.79	28.93	-25024.27	32.28	-24974.16	39.21	-24935.56	34.64	-24890.26	36.76	-24871.47	33.96
-25114.83	28.06	-25070.24	29.48	-25019.84	36.70	-24982.22	31.15	-24936.58	33.61	-24893.08	33.94	-24871.79	33.65
-25114.88	28.01	-25071.16	28.55	-25026.60	29.94	-24981.10	32.27	-24937.00	33.20	-24897.64	29.38	-24872.63	32.81
-25115.38	27.51	-25069.44	30.28	-25025.61	30.93	-24978.08	35.29	-24935.52	34.68	-24887.98	39.05	-24866.78	38.65
-25113.85	29.04	-25070.08	29.63	-25023.58	32.96	-24981.14	32.23	-24935.06	35.13	-24892.11	34.92	-24870.72	34.72
-25112.29	30.60	-25070.64	29.08	-25026.40	30.14	-24982.98	30.39	-24933.69	36.51	-24894.11	32.92	-24861.80	43.64
-25114.27	28.62	-25068.74	30.98	-25024.61	31.94	-24979.31	34.06	-24931.94	38.26	-24897.70	29.33	-24869.01	36.42
-25113.74	29.15	-25070.12	29.59	-25027.38	29.16	-24979.66	33.71	-24934.98	35.22	-24893.08	33.94	-24871.05	34.39

	-25114.43	28.46	-25068.68	31.03	-25024.28	32.26	-24980.75	32.62	-24931.67	38.53	-24888.08	38.94	-24866.24	39.19
	-25115.04	27.85	-25069.24	30.47	-25025.32	31.22	-24984.56	28.81	-24939.78	30.42	-24897.76	29.26	-24863.87	41.57
	-25112.61	30.28	-25068.56	31.15	-25026.55	29.99	-24981.20	32.17	-24934.89	35.31	-24883.43	43.59	-24871.54	33.90
	-25114.31	28.58	-25068.31	31.41	-25024.72	31.82	-24983.52	29.85	-24937.21	32.99	-24888.39	38.64	-24872.94	32.49
	-25114.15	28.74	-25067.71	32.00	-25023.82	32.73	-24981.78	31.59	-24935.83	34.36	-24891.71	35.32	-24870.16	35.28
	-25113.09	29.80	-25068.42	31.30	-25026.09	30.45	-24981.51	31.86	-24934.07	36.13	-24893.26	33.77	-24872.20	33.23
	-25114.78	28.10	-25070.21	29.51	-25027.73	28.81			-24937.13	33.06			-24870.90	34.53
	-25112.62	30.26	-25070.05	29.66	-25024.62	31.93								
Average energies	-25113.91	28.98	-25069.41	30.30	-25024.61	31.93	-24980.27	33.10	-24935.75	34.45	-24892.28	34.74	-24869.75	35.69

Pu

	ground state energy	solid solution energy												
concentration in solid solution (%)	3		6		9		12		15		18		21	
number of atoms	2		4		6		8		10		12		13	
	-25108.43	28.47	-25057.65	30.10	-25009.03	29.56	-24957.04	32.39	-24907.16	33.10	-24856.95	34.16	-24832.48	34.05
	-25107.43	29.48	-25057.51	30.23	-25010.01	28.58	-24959.91	29.51	-24905.17	35.10	-24853.86	37.25	-24821.65	44.88
	-25107.43	29.47	-25058.77	28.97	-25003.52	35.06	-24956.04	33.39	-24908.99	31.28	-24859.18	31.93	-24833.95	32.58
	-25109.24	27.66	-25059.35	28.39	-25007.38	31.21	-24960.38	29.04	-24908.80	31.47	-24862.67	28.44	-24833.48	33.05
	-25108.46	28.44	-25056.65	31.10	-25009.01	29.57	-24955.68	33.75	-24904.38	35.89	-24854.00	37.11	-24833.45	33.08
	-25108.35	28.55	-25057.90	29.85	-25004.99	33.59	-24947.86	41.57	-24908.61	31.66	-24859.57	31.54	-24827.52	39.01
	-25109.08	27.82	-25057.15	30.60	-25004.48	34.10	-24954.38	35.04	-24904.23	36.04	-24856.24	34.87	-24832.91	33.62
	-25109.21	27.69	-25056.91	30.83	-25007.85	30.73	-24954.49	34.94	-24910.66	29.60	-24850.96	40.15	-24832.12	34.41
	-25108.87	28.03	-25057.08	30.67	-25006.89	31.70	-24957.17	32.26	-24909.49	30.78	-24857.22	33.89	-24835.17	31.36
	-25108.89	28.01	-25057.51	30.23	-25008.19	30.40	-24956.92	32.50	-24908.85	31.42	-24849.03	42.08	-24835.94	30.59
	-25109.66	27.25	-25057.10	30.65	-25006.37	32.21	-24955.94	33.48	-24910.31	29.96	-24852.67	38.44	-24835.08	31.45
	-25108.94	27.96	-25057.39	30.35	-25005.78	32.81	-24957.81	31.62	-24906.51	33.76	-24853.40	37.71	-24830.82	35.71
	-25103.28	33.63	-25056.23	31.52	-25007.96	30.63	-24955.80	33.63	-24905.48	34.79	-24861.40	29.71	-24832.92	33.61
	-25108.29	28.61	-25057.61	30.13	-25004.89	33.70	-24948.13	41.30	-24907.29	32.98	-24856.23	34.88	-24828.92	37.61
	-25108.91	27.99	-25057.75	30.00	-25006.04	32.55	-24959.27	30.15	-24906.16	34.10	-24855.40	35.71	-24830.12	36.41
	-25107.23	29.68	-25058.85	28.90	-25008.15	30.43	-24960.03	29.40	-24907.25	33.02	-24855.49	35.62	-24832.13	34.40
	-25109.67	27.24	-25056.52	31.22	-25009.81	28.78	-24956.36	33.07	-24907.19	33.08	-24860.65	30.46	-24831.70	34.83
	-25108.44	28.46	-25055.99	31.75	-25007.27	31.32	-24957.53	31.90	-24906.08	34.19	-24853.28	37.83	-24830.73	35.80
	-25108.96	27.95	-25057.74	30.00	-25004.20	34.38	-24954.41	35.02	-24906.39	33.88	-24858.24	32.87	-24832.28	34.25
	-25108.24	28.66	-25057.18	30.57	-25007.74	30.84	-24955.82	33.60	-24909.39	30.87	-24859.49	31.62	-24829.23	37.30
	-25107.46	29.45	-25058.68	29.07	-25007.49	31.10	-24957.65	31.78	-24906.30	33.96	-24861.71	29.40	-24826.61	39.92
	-25109.19	27.71	-25054.77	32.97	-25008.28	30.30	-24957.99	31.44	-24907.32	32.95	-24859.40	31.71	-24834.76	31.77
	-25108.54	28.36	-25058.41	29.33	-25008.06	30.52	-24957.28	32.15	-24907.21	33.06	-24861.70	29.41	-24832.06	34.47
	-25108.25	28.65	-25059.02	28.73	-25009.83	28.76	-24958.34	31.09	-24906.38	33.88	-24853.72	37.39	-24833.07	33.46

-25108.76	28.15	-25057.65	30.10	-25007.72	30.87	-24959.02	30.41	-24907.89	32.38	-24858.23	32.88	-24830.83	35.70
-25107.99	28.91	-25057.95	29.80	-25008.48	30.11	-24957.92	31.50	-24908.71	31.55	-24854.02	37.09	-24836.09	30.44
-25108.48	28.43	-25057.50	30.24	-25007.01	31.57	-24958.81	30.62	-24910.05	30.22	-24857.04	34.06	-24830.87	35.66
-25109.61	27.29	-25055.71	32.03	-25006.04	32.55	-24957.54	31.89	-24909.85	30.42	-24856.12	34.99	-24834.54	31.99
-25108.29	28.62	-25059.03	28.72	-25004.49	34.10	-24951.97	37.45	-24906.78	33.49	-24855.62	35.49	-24836.00	30.53
-25107.13	29.78	-25057.60	30.15	-25008.59	29.99	-24955.55	33.88	-24905.64	34.62	-24855.06	36.05	-24836.42	30.12
-25106.95	29.96	-25058.97	28.78	-25005.71	32.87	-24956.63	32.79	-24908.44	31.83	-24855.22	35.89	-24829.76	36.77
-25108.70	28.20	-25057.76	29.99	-25007.94	30.64	-24954.38	35.05	-24908.69	31.58	-24855.46	35.65	-24831.80	34.73
-25100.21	36.70	-25058.93	28.81	-25009.71	28.87	-24960.42	29.01	-24905.51	34.75	-24858.63	32.48	-24835.79	30.74
-25107.77	29.13	-25057.74	30.00	-25006.80	31.79	-24959.00	30.43	-24909.40	30.87	-24859.40	31.71	-24829.82	36.71
-25108.67	28.23	-25057.71	30.03	-25005.39	33.20	-24955.50	33.92	-24907.25	33.02	-24859.52	31.59	-24825.34	41.19
-25108.69	28.22	-25057.92	29.83	-25008.03	30.55	-24952.18	37.25	-24906.68	33.59	-24855.47	35.64	-24831.95	34.58
-25109.11	27.79	-25056.52	31.23	-25007.03	31.55	-24956.24	33.18	-24908.73	31.53	-24859.06	32.05	-24831.04	35.49
-25107.64	29.27	-25056.42	31.32	-25006.42	32.16	-24957.65	31.78	-24908.33	31.94	-24856.83	34.28	-24834.48	32.06
-25107.98	28.93	-25056.53	31.21	-25006.26	32.33	-24958.79	30.64	-24910.05	30.22	-24856.50	34.61	-24831.48	35.05
-25109.53	27.37	-25058.45	29.30	-25009.19	29.40	-24952.13	37.30	-24903.33	36.94	-24857.87	33.24	-24828.79	37.74
-25107.56	29.34	-25058.03	29.72	-25009.15	29.44	-24953.57	35.86	-24906.25	34.02	-24857.79	33.32	-24831.87	34.66
-25109.21	27.69	-25058.56	29.18	-25006.61	31.98	-24958.40	31.03	-24908.36	31.91	-24853.65	37.46	-24829.93	36.60
-25107.07	29.83	-25056.39	31.36	-25006.42	32.17	-24955.54	33.89	-24909.44	30.83	-24856.90	34.21	-24836.77	29.76
-25108.18	28.72	-25058.36	29.39	-25006.80	31.79	-24954.66	34.77	-24906.67	33.60	-24854.82	36.29	-24832.51	34.02
-25108.66	28.24	-25056.90	30.84	-25008.87	29.72	-24952.77	36.66	-24909.41	30.85	-24850.01	41.10	-24827.67	38.86
-25107.85	29.05	-25060.07	27.67	-25008.30	30.29	-24958.14	31.28	-24907.43	32.83	-24857.17	33.94	-24831.59	34.94
-25107.77	29.13	-25059.10	28.65	-25005.82	32.77	-24957.87	31.56	-24906.47	33.79	-24857.49	33.62	-24833.08	33.45
-25109.86	27.05	-25054.86	32.89	-25007.12	31.46	-24955.88	33.55	-24909.60	30.66	-24855.64	35.47	-24828.27	38.26
-25109.43	27.48	-25056.43	31.32	-25008.76	29.83	-24956.60	32.82	-24907.48	32.79	-24857.99	33.12	-24835.69	30.84
-25108.32	28.58	-25055.14	32.60	-25007.08	31.51	-24960.64	28.79	-24903.50	36.77	-24860.22	30.89	-24834.26	32.27
-25108.15	28.75	-25057.04	30.70	-25004.34	34.24	-24957.95	31.48	-24910.26	30.00	-24857.10	34.01	-24830.86	35.67
-25108.25	28.66	-25059.81	27.93	-25003.12	35.47	-24958.17	31.25	-24908.10	32.16	-24856.72	34.39	-24829.56	36.97
-25109.39	27.51	-25055.48	32.27	-25006.73	31.86	-24954.71	34.72	-24907.95	32.31	-24852.00	39.11	-24834.83	31.70
-25106.67	30.24	-25055.15	32.59	-25007.17	31.42	-24956.96	32.46	-24909.80	30.47	-24857.11	34.00	-24834.65	31.88
-25107.84	29.06	-25058.26	29.49	-25009.26	29.32	-24948.54	40.89	-24908.01	32.26	-24854.42	36.69	-24833.84	32.69
-25108.68	28.23	-25057.67	30.08	-25008.30	30.29	-24959.36	30.07	-24906.60	33.67	-24852.34	38.77	-24833.70	32.83
-25108.58	28.32	-25056.84	30.91	-25012.00	26.58	-24958.63	30.79	-24907.03	33.24	-24858.97	32.14	-24831.14	35.39
-25108.50	28.41	-25057.45	30.29	-25008.98	29.61	-24956.09	33.33	-24907.90	32.37	-24856.01	35.10	-24826.34	40.19
-25108.66	28.25	-25056.50	31.24	-25006.63	31.95	-24958.11	31.32	-24908.45	31.82	-24857.08	34.03	-24833.22	33.31
-25109.32	27.58	-25056.80	30.94	-25010.97	27.62	-24956.83	32.60	-24907.59	32.68	-24856.75	34.36	-24833.55	32.98
-25109.20	27.71	-25057.18	30.57	-25009.58	29.01	-24955.56	33.87	-24910.07	30.20	-24856.19	34.92	-24832.35	34.18
-25108.49	28.41	-25059.26	28.49	-25007.02	31.57	-24959.18	30.24	-24902.69	37.58	-24857.56	33.55	-24830.12	36.41
-25107.72	29.18	-25054.07	33.67	-25008.06	30.53	-24958.71	30.71	-24910.56	29.71	-24856.79	34.32	-24833.07	33.46
-25108.91	28.00	-25058.41	29.33	-25008.95	29.63	-24952.62	36.81	-24906.12	34.15	-24859.14	31.97	-24832.55	33.98
-25109.32	27.58	-25058.13	29.62	-25007.01	31.58	-24955.14	34.29	-24909.98	30.29	-24860.00	31.11	-24832.40	34.13

-25108.99	27.91	-25058.91	28.83	-25007.09	31.50	-24957.58	31.85	-24910.55	29.71	-24855.70	35.41	-24831.43	35.10
-25108.69	28.21	-25056.68	31.06	-25006.06	32.53	-24958.23	31.19	-24910.13	30.14	-24859.52	31.59	-24834.40	32.13
-25108.96	27.94	-25060.07	27.68	-25007.68	30.90	-24958.57	30.86	-24909.10	31.16	-24854.32	36.79	-24831.29	35.24
-25108.94	27.96	-25058.56	29.18	-25007.19	31.39	-24958.01	31.42	-24907.49	32.78	-24858.70	32.41	-24830.96	35.57
-25106.04	30.86	-25059.41	28.34	-25003.53	35.06	-24958.11	31.32	-24904.92	35.35	-24852.55	38.56	-24831.66	34.87
-25108.01	28.89	-25058.88	28.87	-25009.17	29.41	-24956.99	32.43	-24908.88	31.38	-24858.36	32.75	-24827.91	38.62
-25108.26	28.64	-25056.48	31.27	-25008.14	30.45	-24958.09	31.33	-24904.77	35.50	-24859.76	31.35	-24831.86	34.67
-25107.10	29.80	-25058.66	29.09	-25004.85	33.74	-24954.75	34.68	-24903.79	36.48	-24858.96	32.14	-24832.96	33.57
-25109.20	27.70	-25056.39	31.35	-25004.82	33.77	-24959.71	29.72	-24907.26	33.01	-24855.77	35.34	-24833.00	33.53
-25108.54	28.36	-25058.37	29.38	-25006.81	31.78	-24951.61	37.82	-24899.57	40.70	-24857.07	34.04	-24829.89	36.64
-25109.02	27.89	-25057.15	30.59	-25009.62	28.97	-24958.99	30.43	-24906.71	33.56	-24852.83	38.28	-24832.68	33.85
-25108.75	28.15	-25058.41	29.33	-25007.54	31.05	-24956.25	33.18	-24906.03	34.24	-24855.80	35.31	-24833.15	33.38
-25108.61	28.30	-25057.70	30.05	-25010.33	28.26	-24957.36	32.06	-24910.18	30.08	-24855.49	35.62	-24830.35	36.18
-25109.71	27.19	-25058.97	28.77	-25008.21	30.38	-24953.23	36.20	-24910.17	30.10	-24856.67	34.44	-24835.66	30.87
-25107.79	29.11	-25059.76	27.98	-25006.59	31.99	-24958.00	31.43	-24909.20	31.07	-24857.54	33.57	-24838.46	28.07
-25108.82	28.08	-25057.27	30.48	-25009.87	28.71	-24961.60	27.83	-24904.79	35.48	-24858.84	32.26	-24832.37	34.16
-25110.39	26.51	-25059.20	28.54	-25007.20	31.38	-24957.82	31.60	-24903.69	36.58	-24853.56	37.55	-24834.40	32.13
-25107.85	29.05	-25057.74	30.00	-25008.36	30.22	-24961.40	28.02	-24904.09	36.18	-24857.54	33.57	-24833.65	32.88
-25108.85	28.05	-25059.09	28.66	-25008.30	30.29	-24957.14	32.29	-24908.39	31.88	-24860.20	30.91	-24829.16	37.37
-25108.64	28.26	-25057.69	30.05	-25008.99	29.60	-24956.37	33.06	-24910.74	29.53	-24861.44	29.67	-24832.45	34.08
-25108.31	28.59	-25057.71	30.03	-25007.21	31.38	-24956.38	33.05	-24910.09	30.18	-24857.69	33.42	-24827.62	38.91
-25108.64	28.26	-25057.14	30.60	-25005.85	32.73	-24957.76	31.67	-24907.60	32.67	-24857.73	33.38	-24833.62	32.91
-25108.98	27.93	-25054.80	32.95	-25006.42	32.17	-24952.77	36.65	-24905.95	34.32	-24857.91	33.20	-24832.11	34.42
-25109.60	27.30	-25056.63	31.11	-25007.76	30.83	-24957.57	31.86	-24904.08	36.19	-24857.89	33.22	-24831.40	35.13
-25108.59	28.31	-25059.00	28.75	-25008.16	30.42	-24960.36	29.07	-24908.88	31.39	-24860.06	31.05	-24835.23	31.30
-25107.36	29.55	-25059.09	28.65	-25007.50	31.09	-24955.61	33.82	-24909.16	31.11	-24854.81	36.30	-24832.72	33.81
-25108.30	28.60	-25058.90	28.85	-25007.48	31.10	-24956.14	33.29	-24903.91	36.36	-24851.51	39.60	-24832.48	34.05
-25108.58	28.32	-25058.57	29.18	-25006.86	31.73	-24957.57	31.86	-24910.64	29.62	-24859.76	31.35	-24832.82	33.71
-25109.08	27.82	-25057.29	30.46	-25007.37	31.21	-24954.53	34.90	-24906.47	33.80	-24860.36	30.75	-24834.36	32.17
-25107.97	28.93	-25058.18	29.57	-25009.18	29.41	-24955.89	33.53	-24907.77	32.50	-24857.59	33.52	-24826.16	40.37
-25108.63	28.28	-25055.58	32.16	-25008.59	30.00	-24958.58	30.85	-24908.88	31.39	-24859.37	31.74	-24833.06	33.47
-25110.03	26.87	-25058.36	29.39	-25007.79	30.79	-24957.70	31.73	-24907.74	32.53	-24860.57	30.54	-24833.53	33.01
-25106.95	29.96	-25058.99	28.75	-25008.15	30.44	-24950.97	38.46	-24908.62	31.64	-24854.33	36.78	-24833.77	32.76
-25106.45	30.46	-25058.79	28.96	-25007.07	31.51	-24955.47	33.96	-24908.98	31.29	-24858.44	32.67	-24833.11	33.42
-25107.24	29.66	-25059.30	28.44	-25010.37	28.21	-24948.01	41.41	-24904.86	35.41	-24859.04	32.07	-24830.08	36.45
-25109.20	27.71	-25056.44	31.31	-25008.66	29.93	-24957.60	31.83	-24909.83	30.44	-24852.50	38.61	-24834.18	32.35
-25107.00	29.91	-25058.52	29.22	-25004.77	33.81	-24957.39	32.04	-24907.96	32.31	-24858.30	32.81	-24836.69	29.84
-25108.03	28.87	-25059.92	27.83	-25000.77	37.81	-24959.30	30.12	-24910.40	29.87	-24860.45	30.65	-24833.38	33.15
-25109.04	27.86	-25057.90	29.84	-25010.02	28.57	-24957.59	31.84	-24911.11	29.16	-24854.43	36.68	-24830.29	36.24
-25108.71	28.19	-25058.86	28.89	-25009.09	29.50	-24955.40	34.03	-24907.84	32.43	-24858.27	32.84	-24831.64	34.89
-25108.70	28.20	-25057.72	30.02	-25008.30	30.28	-24956.42	33.01	-24905.78	34.49	-24857.54	33.57	-24832.10	34.43

	-25109.17	27.73	-25056.09	31.65	-25009.42	29.16	-24958.89	30.53	-24907.20	33.07	-24854.59	36.52	-24833.07	33.46
	-25108.21	28.69	-25059.17	28.57	-25009.52	29.07	-24959.41	30.01	-24907.20	33.07	-24859.20	31.91	-24833.86	32.67
	-25108.46	28.44	-25055.62	32.12	-25009.74	28.85	-24956.54	32.88	-24908.19	32.08	-24859.30	31.81	-24830.92	35.61
	-25108.48	28.42	-25057.57	30.18	-25008.10	30.48	-24958.41	31.02	-24909.34	30.92	-24857.54	33.57	-24836.33	30.20
	-25108.60	28.30	-25059.38	28.36	-25006.07	32.52	-24959.87	29.56	-24907.52	32.75	-24856.35	34.76	-24831.90	34.64
	-25107.79	29.11	-25054.73	33.01	-25005.70	32.89	-24958.99	30.44	-24905.45	34.82	-24857.66	33.45	-24830.96	35.57
	-25109.25	27.65	-25056.79	30.95	-25010.03	28.56	-24958.96	30.47	-24908.18	32.09	-24857.02	34.09	-24834.83	31.70
	-25108.12	28.78	-25056.12	31.62	-25006.82	31.76	-24959.34	30.09	-24906.03	34.24	-24859.63	31.47	-24837.16	29.37
	-25106.59	30.32	-25058.47	29.27	-25008.57	30.01	-24952.98	36.44	-24908.49	31.78	-24833.61	57.50	-24833.82	32.71
	-25107.83	29.08	-25058.60	29.14	-25003.77	34.82	-24952.40	37.03	-24906.32	33.95	-24861.86	29.25	-24830.31	36.22
	-25108.13	28.77	-25057.31	30.44	-25000.75	37.84	-24958.30	31.12	-24909.87	30.40	-24854.98	36.13	-24834.05	32.48
	-25109.65	27.26	-25054.60	33.15	-25005.73	32.86	-24954.89	34.54	-24910.35	29.92			-24833.58	32.95
					-25009.21	29.37	-24958.10	31.33	-24908.44	31.83			-24830.23	36.30
					-25008.47	30.12								
Average energies	-25108.34	28.56	-25057.64	30.10	-25007.42	31.16	-24956.66	32.77	-24907.62	32.65	-24856.78	34.33	-24832.24	34.29

Th

	ground state energy	solid solution energy												
concentration in solid solution (%)	3		6		9		12		15		18		21	
number of atoms	2		4		6		8		10		12		13	
	-25114.70	27.83	-25068.33	30.67	-25022.36	33.12	-24978.62	33.33	-24932.03	36.39	-24890.36	34.54	-24864.49	38.63
	-25115.11	27.42	-25069.48	29.52	-25024.95	30.53	-24979.98	31.97	-24931.81	36.61	-24888.76	36.13	-24868.49	34.64
	-25114.32	28.22	-25070.36	28.65	-25026.35	29.12	-24971.25	40.70	-24936.76	31.66	-24891.12	33.77	-24866.34	36.78
	-25113.42	29.11	-25068.86	30.15	-25026.03	29.45	-24975.46	36.49	-24931.20	37.22	-24893.10	31.79	-24868.45	34.68
	-25113.77	28.76	-25067.83	31.18	-25023.37	32.11	-24979.98	31.97	-24930.26	38.16	-24891.75	33.14	-24864.71	38.42
	-25113.65	28.89	-25069.24	29.76	-25021.85	33.63	-24977.77	34.18	-24931.99	36.43	-24884.44	40.45	-24865.80	37.33
	-25113.61	28.92	-25069.53	29.47	-25020.83	34.65	-24982.54	29.41	-24935.16	33.26	-24889.29	35.60	-24871.41	31.72
	-25113.30	29.23	-25068.71	30.29	-25024.64	30.84	-24982.77	29.18	-24927.31	41.11	-24891.53	33.36	-24868.67	34.45
	-25113.79	28.74	-25066.50	32.51	-25022.07	33.41	-24972.44	39.51	-24937.68	30.74	-24892.80	32.09	-24869.17	33.96
	-25113.49	29.04	-25070.21	28.80	-25022.95	32.52	-24977.14	34.81	-24934.32	34.10	-24890.23	34.66	-24860.89	42.24
	-25113.02	29.51	-25070.73	28.27	-25025.33	30.15	-24980.40	31.55	-24935.52	32.90	-24893.25	31.65	-24864.63	38.50
	-25112.36	30.17	-25070.31	28.70	-25024.96	30.51	-24965.79	46.16	-24937.01	31.41	-24885.87	39.02	-24866.71	36.42
	-25113.07	29.46	-25069.43	29.58	-25025.64	29.84	-24977.68	34.27	-24933.79	34.63	-24888.83	36.06	-24862.63	40.50
	-25114.25	28.29	-25068.06	30.95	-25026.13	29.35	-24975.23	36.71	-24937.41	31.01	-24889.10	35.80	-24860.77	42.36
	-25115.10	27.44	-25069.56	29.45	-25024.41	31.07	-24982.01	29.94	-24935.76	32.66	-24884.27	40.62	-24874.74	28.39
	-25114.81	27.72	-25068.22	30.79	-25024.47	31.01	-24979.85	32.10	-24935.51	32.91	-24891.35	33.54	-24865.01	38.12
	-25113.52	29.02	-25068.58	30.42	-25021.79	33.69	-24975.20	36.75	-24934.17	34.25	-24893.57	31.33	-24867.99	35.14
	-25114.53	28.00	-25067.21	31.79	-25025.72	29.76	-24974.64	37.31	-24931.71	36.71	-24892.23	32.66	-24868.49	34.64

-25113.60	28.93	-25065.33	33.67	-25024.16	31.31	-24978.56	33.39	-24930.88	37.54	-24889.80	35.09	-24868.59	34.54
-25113.86	28.68	-25069.11	29.90	-25024.02	31.46	-24981.61	30.33	-24931.64	36.78	-24891.44	33.45	-24864.83	38.30
-25112.95	29.58	-25069.66	29.35	-25027.23	28.25	-24976.80	35.15	-24934.79	33.63	-24894.01	30.88	-24867.45	35.68
-25113.38	29.16	-25069.65	29.36	-25023.46	32.01	-24978.87	33.08	-24933.59	34.83	-24885.97	38.92	-24871.51	31.62
-25113.67	28.87	-25066.34	32.66	-25025.14	30.34	-24969.12	42.83	-24935.48	32.94	-24882.97	41.93	-24865.06	38.07
-25114.10	28.43	-25066.91	32.10	-25023.36	32.12	-24979.73	32.22	-24933.65	34.77	-24891.56	33.33	-24868.20	34.93
-25113.49	29.04	-25068.31	30.70	-25024.29	31.18	-24978.59	33.36	-24932.44	35.98	-24888.72	36.18	-24868.64	34.49
-25113.53	29.01	-25068.58	30.43	-25018.71	36.77	-24976.67	35.28	-24936.38	32.04	-24894.18	30.71	-24864.69	38.44
-25114.20	28.33	-25070.81	28.20	-25025.30	30.18	-24979.57	32.38	-24937.36	31.06	-24890.24	34.65	-24868.40	34.73
-25114.12	28.41	-25065.59	33.41	-25023.28	32.20	-24978.95	33.00	-24936.32	32.10	-24887.48	37.41	-24866.73	36.40
-25114.08	28.45	-25068.41	30.60	-25021.71	33.77	-24980.32	31.63	-24935.11	33.31	-24892.65	32.24	-24855.11	48.02
-25114.20	28.33	-25068.71	30.30	-25026.40	29.08	-24980.58	31.37	-24933.01	35.41	-24890.61	34.28	-24859.42	43.71
-25114.01	28.52	-25068.64	30.36	-25025.22	30.26	-24980.68	31.27	-24935.40	33.02	-24894.88	30.01	-24868.02	35.11
-25115.12	27.41	-25070.04	28.97	-25021.55	33.93	-24980.88	31.07	-24928.39	40.03	-24892.05	32.84	-24872.10	31.03
-25114.02	28.52	-25070.39	28.61	-25020.51	34.96	-24980.33	31.62	-24930.65	37.77	-24887.51	37.38	-24868.26	34.87
-25115.84	26.69	-25067.28	31.73	-25022.25	33.23	-24972.57	39.38	-24936.04	32.38	-24890.00	34.90	-24868.40	34.73
-25112.78	29.76	-25065.12	33.89	-25025.04	30.43	-24979.29	32.66	-24931.82	36.60	-24890.80	34.09	-24855.59	47.54
-25115.11	27.42	-25069.15	29.85	-25022.90	32.58	-24981.24	30.70	-24937.06	31.36	-24884.51	40.38	-24868.44	34.69
-25112.88	29.65	-25068.59	30.41	-25020.54	34.94	-24977.02	34.93	-24933.16	35.26	-24878.16	46.73	-24869.67	33.46
-25113.96	28.57	-25068.40	30.61	-25026.44	29.04	-24982.11	29.84	-24906.08	62.34	-24890.04	34.85	-24866.85	36.28
-25113.48	29.05	-25069.43	29.57	-25025.21	30.27	-24980.47	31.48	-24932.88	35.54	-24891.76	33.13	-24861.67	41.45
-25113.58	28.95	-25069.89	29.12	-25021.46	34.02	-24976.97	34.98	-24931.09	37.33	-24891.02	33.87	-24861.19	41.94
-25113.93	28.60	-25070.11	28.90	-25024.66	30.81	-24982.38	29.57	-24930.49	37.93	-24883.57	41.32	-24867.66	35.46
-25113.37	29.17	-25069.02	29.98	-25024.20	31.28	-24979.75	32.20	-24935.95	32.47	-24889.00	35.89	-24861.19	41.94
-25113.26	29.27	-25069.18	29.82	-25023.51	31.96	-24978.46	33.49	-24938.39	30.04	-24889.01	35.88	-24865.04	38.09
-25115.86	26.67	-25069.76	29.24	-25021.19	34.29	-24981.79	30.16	-24934.70	33.72	-24889.61	35.28	-24867.89	35.24
-25114.00	28.53	-25067.03	31.98	-25023.07	32.40	-24978.83	33.12	-24935.50	32.92	-24889.81	35.08	-24868.30	34.82
-25113.87	28.66	-25069.68	29.33	-25024.71	30.76	-24976.24	35.71	-24933.70	34.72	-24893.11	31.78	-24868.35	34.78
-25114.01	28.52	-25066.44	32.57	-25020.12	35.36	-24979.79	32.16	-24934.28	34.14	-24884.70	40.20	-24861.54	41.59
-25114.10	28.43	-25070.69	28.32	-25022.03	33.45	-24979.43	32.52	-24939.58	28.84	-24888.53	36.36	-24867.32	35.81
-25114.42	28.11	-25068.40	30.60	-25021.47	34.01	-24975.27	36.68	-24938.92	29.50	-24889.06	35.83	-24872.04	31.09
-25113.50	29.04	-25071.04	27.96	-25023.37	32.11	-24981.21	30.74	-24928.01	40.41	-24881.11	43.78	-24864.94	38.19
-25114.09	28.45	-25069.07	29.94	-25024.67	30.80	-24978.11	33.84	-24928.88	39.54	-24882.65	42.24	-24870.79	32.34
-25113.79	28.75	-25068.57	30.44	-25023.70	31.78	-24979.07	32.88	-24929.20	39.22	-24882.54	42.35	-24868.01	35.12
-25115.05	27.49	-25068.54	30.46	-25024.24	31.24	-24978.79	33.16	-24931.43	36.99	-24884.72	40.18	-24867.03	36.10
-25114.87	27.66	-25070.53	28.48	-25025.07	30.40	-24977.15	34.80	-24929.95	38.47	-24889.55	35.34	-24866.54	36.59
-25115.13	27.40	-25067.35	31.66	-25024.07	31.40	-24977.03	34.92	-24937.03	31.39	-24879.96	44.93	-24866.67	36.46
-25110.33	32.20	-25069.71	29.29	-25020.78	34.69	-24977.24	34.71	-24933.93	34.49	-24887.68	37.21	-24859.52	43.61
-25113.79	28.74	-25070.25	28.76	-25024.85	30.62	-24981.01	30.94	-24936.35	32.07	-24885.59	39.30	-24870.92	32.20
-25113.31	29.22	-25068.88	30.12	-25025.83	29.64	-24977.29	34.66	-24932.55	35.87	-24891.64	33.26	-24867.61	35.51
-25113.06	29.47	-25069.33	29.67	-25023.22	32.26	-24979.21	32.74	-24933.32	35.10	-24885.75	39.14	-24869.63	33.50

-25114.61	27.92	-25068.88	30.12	-25025.55	29.93	-24976.10	35.85	-24938.20	30.22	-24888.41	36.48	-24863.81	39.32
-25114.30	28.23	-25067.81	31.19	-25023.49	31.99	-24978.61	33.34	-24937.90	30.52	-24891.20	33.69	-24866.55	36.57
-25114.71	27.83	-25069.92	29.08	-25020.10	35.37	-24977.96	33.99	-24929.46	38.96	-24887.38	37.51	-24867.31	35.81
-25114.16	28.37	-25069.62	29.39	-25023.78	31.70	-24982.21	29.74	-24935.14	33.28	-24887.40	37.49	-24867.84	35.29
-25113.90	28.63	-25066.64	32.37	-25024.16	31.32	-24978.27	33.68	-24917.82	50.60	-24889.48	35.41	-24872.25	30.88
-25113.77	28.76	-25070.38	28.62	-25022.64	32.83	-24969.26	42.69	-24934.38	34.04	-24893.59	31.31	-24867.75	35.38
-25115.57	26.96	-25069.91	29.10	-25020.16	35.32	-24981.89	30.06	-24930.52	37.90	-24891.26	33.64	-24870.89	32.24
-25113.41	29.12	-25070.41	28.60	-25024.82	30.66	-24977.38	34.57	-24935.41	33.01	-24889.92	34.97	-24864.22	38.91
-25113.24	29.29	-25069.60	29.41	-25026.38	29.10	-24979.00	32.95	-24929.19	39.23	-24882.39	42.50	-24865.56	37.57
-25113.69	28.85	-25066.83	32.18	-25023.52	31.96	-24976.71	35.24	-24925.83	42.59	-24891.43	33.46	-24868.40	34.72
-25114.15	28.38	-25066.83	32.18	-25019.08	36.40	-24978.18	33.77	-24935.40	33.02	-24889.71	35.18	-24870.23	32.90
-25113.35	29.18	-25070.84	28.17	-25025.02	30.46	-24979.12	32.83	-24932.44	35.98	-24890.34	34.55	-24862.25	40.88
-25114.41	28.12	-25069.90	29.10	-25020.26	35.21	-24978.19	33.76	-24940.26	28.16	-24887.48	37.41	-24871.64	31.49
-25114.23	28.30	-25069.26	29.75	-25024.95	30.53	-24978.26	33.69	-24935.85	32.57	-24892.56	32.34	-24864.32	38.81
-25113.28	29.25	-25068.23	30.78	-25024.44	31.04	-24979.75	32.20	-24933.87	34.55	-24892.45	32.44	-24871.97	31.16
-25113.11	29.43	-25068.93	30.07	-25025.35	30.12	-24978.01	33.94	-24937.16	31.26	-24892.34	32.55	-24868.92	34.20
-25113.24	29.30	-25067.81	31.19	-25021.96	33.52	-24978.25	33.70	-24932.77	35.65	-24893.45	31.45	-24866.22	36.91
-25112.93	29.61	-25070.74	28.27	-25023.14	32.34	-24976.88	35.07	-24932.54	35.88	-24890.04	34.85	-24867.68	35.44
-25114.89	27.65	-25069.48	29.52	-25022.93	32.54	-24978.28	33.67	-24936.01	32.41	-24887.36	37.54	-24868.66	34.46
-25113.07	29.46	-25068.69	30.32	-25022.20	33.28	-24978.36	33.59	-24935.90	32.52	-24893.69	31.20	-24873.51	29.62
-25114.10	28.44	-25070.11	28.90	-25024.77	30.71	-24979.35	32.60	-24938.98	29.44	-24893.17	31.72	-24867.14	35.99
-25114.81	27.72	-25070.77	28.24	-25023.01	32.47	-24980.39	31.56	-24935.91	32.51	-24885.84	39.06	-24867.91	35.22
-25113.67	28.86	-25068.58	30.43	-25021.77	33.71	-24978.83	33.12	-24936.17	32.25	-24893.62	31.27	-24867.37	35.76
-25113.97	28.56	-25065.36	33.64	-25020.09	35.39	-24975.91	36.03	-24937.14	31.29	-24890.42	34.47	-24865.47	37.66
-25114.40	28.13	-25068.76	30.24	-25024.73	30.75	-24974.90	37.05	-24933.06	35.36	-24890.54	34.36	-24861.84	41.28
-25113.77	28.76	-25068.63	30.37	-25021.85	33.62	-24979.82	32.13	-24934.32	34.10	-24894.75	30.15	-24866.54	36.58
-25114.78	27.75	-25067.76	31.24	-25024.01	31.47	-24978.79	33.16	-24937.07	31.35	-24892.20	32.69	-24864.77	38.36
-25114.00	28.53	-25066.43	32.57	-25026.86	28.62	-24971.80	40.15	-24934.18	34.24	-24887.21	37.68	-24864.13	39.00
-25115.22	27.31	-25069.34	29.66	-25023.97	31.51	-24976.55	35.40	-24938.08	30.34	-24889.94	34.95	-24868.25	34.88
-25114.03	28.50	-25067.68	31.33	-25025.57	29.91	-24980.23	31.72	-24929.41	39.01	-24891.69	33.20	-24867.96	35.17
-25114.45	28.08	-25067.89	31.12	-25019.70	35.78	-24982.76	29.18	-24931.56	36.86	-24893.20	31.69	-24866.35	36.78
-25114.63	27.90	-25068.49	30.51	-25024.22	31.26	-24978.80	33.15	-24933.93	34.49	-24888.39	36.50	-24870.31	32.82
-25113.43	29.10	-25070.58	28.42	-25022.45	33.03	-24978.74	33.21	-24933.24	35.18	-24883.45	41.44	-24869.91	33.21
-25113.97	28.56	-25067.74	31.27	-25025.39	30.09	-24981.12	30.83	-24929.34	39.08	-24891.83	33.06	-24871.76	31.37
-25113.74	28.79	-25068.71	30.30	-25023.76	31.71	-24974.36	37.59	-24932.30	36.12	-24890.40	34.49	-24866.90	36.23
-25112.98	29.56	-25068.33	30.67	-25022.92	32.56	-24979.90	32.05	-24929.84	38.58	-24879.82	45.07	-24864.86	38.27
-25114.14	28.40	-25068.96	30.04	-25024.54	30.94	-24980.53	31.42	-24936.12	32.30	-24890.19	34.70	-24868.53	34.60
-25111.41	31.13	-25069.54	29.46	-25022.64	32.84	-24981.06	30.89	-24937.92	30.50	-24891.73	33.16	-24867.41	35.71
-25114.85	27.68	-25068.74	30.26	-25022.53	32.94	-24983.17	28.78	-24934.67	33.75	-24891.03	33.86	-24862.83	40.30
-25113.37	29.16	-25069.39	29.62	-25023.33	32.15	-24977.60	34.35	-24933.55	34.87	-24884.68	40.22	-24867.31	35.82
-25113.90	28.64	-25070.39	28.61	-25022.02	33.46	-24979.64	32.31	-24935.65	32.77	-24887.29	37.60	-24865.34	37.78

	-25113.42	29.11	-25070.11	28.90	-25025.27	30.20	-24979.08	32.86	-24931.29	37.13	-24890.26	34.63	-24866.27	36.86
	-25112.21	30.33	-25070.27	28.74	-25026.31	29.17	-24978.85	33.09	-24936.82	31.60	-24892.86	32.04	-24868.92	34.21
	-25113.44	29.09	-25068.21	30.79	-25024.95	30.53	-24981.20	30.75	-24931.68	36.74	-24889.04	35.85	-24865.82	37.31
	-25112.83	29.71	-25067.16	31.84	-25020.09	35.39	-24978.92	33.03	-24930.34	38.08	-24888.74	36.15	-24875.14	27.99
	-25113.67	28.86	-25069.09	29.92	-25026.27	29.21	-24975.22	36.73	-24932.04	36.38	-24888.34	36.55	-24868.78	34.35
	-25113.13	29.40	-25072.05	26.96	-25022.07	33.41	-24981.51	30.44	-24931.49	36.93	-24883.53	41.36	-24867.62	35.50
	-25111.88	30.65	-25069.14	29.87	-25023.33	32.15	-24977.15	34.80	-24932.22	36.20	-24885.81	39.08	-24867.78	35.35
	-25113.18	29.35	-25069.52	29.49	-25024.88	30.59	-24980.88	31.07	-24930.22	38.20	-24891.23	33.66	-24863.26	39.87
	-25115.38	27.15	-25070.30	28.70	-25024.03	31.45	-24977.51	34.44	-24936.60	31.82	-24892.50	32.40	-24871.53	31.59
	-25113.61	28.92	-25070.66	28.35	-25023.91	31.57	-24979.63	32.31	-24932.78	35.64	-24889.04	35.86	-24875.07	28.06
	-25112.13	30.40	-25067.93	31.07	-25025.17	30.31	-24980.60	31.35	-24936.11	32.31	-24891.04	33.85	-24870.71	32.42
	-25113.87	28.67	-25069.77	29.24	-25024.20	31.28	-24982.84	29.11	-24934.76	33.66	-24889.42	35.47	-24865.69	37.44
	-25114.54	27.99	-25063.90	35.10	-25021.87	33.60	-24978.08	33.87	-24937.43	30.99	-24887.63	37.26	-24862.99	40.13
	-25114.07	28.46	-25069.46	29.54	-25026.09	29.39	-24977.21	34.74	-24938.01	30.41	-24889.82	35.07	-24864.00	39.12
	-25112.00	30.54	-25066.14	32.86	-25024.45	31.02	-24978.49	33.46	-24933.15	35.27	-24892.19	32.70	-24867.49	35.64
	-25113.66	28.87			-25025.75	29.73	-24979.64	32.31	-24932.35	36.07			-24860.57	42.56
	-25113.49	29.04			-25020.66	34.82			-24936.39	32.03			-24865.79	37.33
	-25115.06	27.48							-24931.69	36.73				
	-25113.65	28.89							-24938.24	30.19				
									-24931.34	37.08				
Average energies	-25113.8	28.6982	-25068.8	30.17325	-25023.6	31.88605	-24978.4	33.50523	-24933.6	34.85123	-24889.3	35.63533	-24866.9	36.20916

U

	ground state energy	solid solution energy												
concentration in solid solution (%)	3		6		9		12		15		18		21	
number of atoms	2		4		6		8		10		12		13	
	-25150.62	-3.07	-25109.43	-0.39	-25067.32	3.21	-25026.92	5.10	-24992.47	1.04	-24950.48	4.53	-24928.66	7.09
	-25149.64	-2.09	-25110.43	-1.39	-25069.95	0.59	-25028.31	3.71	-24989.32	4.19	-24944.82	10.18	-24928.49	7.26
	-25150.96	-3.41	-25109.03	0.02	-25069.77	0.76	-25029.19	2.84	-24987.74	5.77	-24951.41	3.59	-24927.82	7.93
	-25148.83	-1.28	-25106.26	2.79	-25068.44	2.09	-25032.51	-0.49	-24989.99	3.53	-24951.20	3.80	-24932.63	3.12
	-25149.24	-1.68	-25109.09	-0.05	-25063.63	6.90	-25026.98	5.05	-24985.35	8.17	-24949.97	5.03	-24928.05	7.70
	-25150.52	-2.97	-25108.75	0.29	-25070.29	0.25	-25029.22	2.81	-24990.66	2.86	-24948.91	6.09	-24925.28	10.47
	-25149.08	-1.53	-25107.49	1.55	-25071.53	-0.99	-25028.93	3.09	-24986.78	6.74	-24947.87	7.13	-24923.60	12.15
	-25150.07	-2.52	-25110.78	-1.73	-25069.09	1.44	-25032.65	-0.63	-24989.38	4.14	-24949.10	5.91	-24927.71	8.04
	-25149.49	-1.94	-25110.22	-1.18	-25061.05	9.49	-25029.63	2.40	-24985.88	7.63	-24951.45	3.55	-24926.48	9.27
	-25149.61	-2.06	-25110.35	-1.31	-25068.15	2.38	-25027.98	4.04	-24988.13	5.38	-24944.39	10.61	-24925.54	10.21
	-25150.52	-2.97	-25108.73	0.31	-25069.78	0.76	-25029.71	2.31	-24990.11	3.41	-24946.18	8.83	-24933.38	2.37
	-25150.44	-2.89	-25109.45	-0.40	-25069.06	1.48	-25024.33	7.70	-24990.60	2.92	-24950.09	4.92	-24930.88	4.87

-25149.40	-1.85	-25106.88	2.16	-25068.17	2.37	-25028.23	3.80	-24990.15	3.37	-24944.50	10.51	-24931.62	4.13
-25149.96	-2.41	-25109.16	-0.12	-25070.89	-0.35	-25029.35	2.67	-24988.86	4.66	-24946.21	8.79	-24926.97	8.78
-25148.64	-1.09	-25104.96	4.08	-25071.14	-0.60	-25031.60	0.42	-24989.30	4.22	-24944.13	10.87	-24929.42	6.33
-25149.30	-1.74	-25110.50	-1.45	-25068.78	1.75	-25027.50	4.52	-24991.00	2.52	-24947.27	7.74	-24928.70	7.05
-25150.80	-3.25	-25111.22	-2.17	-25068.49	2.05	-25028.77	3.25	-24990.36	3.15	-24951.70	3.30	-24919.52	16.23
-25150.22	-2.66	-25110.89	-1.84	-25070.69	-0.16	-25031.01	1.01	-24990.87	2.64	-24949.66	5.35	-24927.21	8.54
-25150.74	-3.19	-25109.05	-0.01	-25069.19	1.35	-25030.49	1.54	-24987.98	5.53	-24947.74	7.27	-24925.87	9.88
-25149.91	-2.36	-25109.97	-0.93	-25071.78	-1.24	-25030.18	1.84	-24991.92	1.59	-24949.70	5.31	-24925.92	9.83
-25149.13	-1.58	-25109.95	-0.91	-25069.89	0.64	-25023.37	8.65	-24987.08	6.43	-24950.61	4.40	-24928.87	6.88
-25150.78	-3.23	-25109.25	-0.21	-25070.14	0.40	-25028.30	3.73	-24987.48	6.04	-24951.40	3.60	-24927.02	8.73
-25150.22	-2.66	-25109.87	-0.82	-25069.89	0.65	-25027.79	4.23	-24989.12	4.39	-24948.77	6.23	-24931.54	4.21
-25149.54	-1.98	-25109.85	-0.81	-25044.52	26.01	-25029.98	2.05	-24990.86	2.66	-24944.67	10.34	-24930.94	4.81
-25149.94	-2.39	-25108.43	0.61	-25071.04	-0.51	-25027.86	4.16	-24993.03	0.48	-24948.26	6.75	-24933.78	1.97
-25148.94	-1.38	-25109.21	-0.17	-25067.25	3.29	-25030.19	1.84	-24985.58	7.93	-24951.80	3.20	-24927.66	8.09
-25150.61	-3.06	-25109.60	-0.56	-25068.76	1.77	-25027.72	4.30	-24987.93	5.59	-24950.99	4.02	-24930.49	5.26
-25150.14	-2.58	-25111.95	-2.91	-25068.23	2.30	-25026.87	5.15	-24988.05	5.47	-24952.52	2.49	-24927.13	8.62
-25150.15	-2.60	-25109.63	-0.59	-25070.26	0.27	-25027.28	4.74	-24990.70	2.81	-24951.92	3.09	-24934.34	1.41
-25149.82	-2.27	-25102.60	6.44	-25068.19	2.34	-25029.74	2.29	-24989.91	3.60	-24950.40	4.61	-24930.90	4.85
-25149.47	-1.91	-25109.89	-0.85	-25069.73	0.80	-25026.74	5.28	-24992.36	1.16	-24949.53	5.47	-24929.28	6.47
-25149.73	-2.18	-25108.34	0.70	-25072.09	-1.56	-25031.15	0.87	-24990.34	3.18	-24948.67	6.33	-24929.00	6.75
-25150.28	-2.73	-25109.88	-0.83	-25068.64	1.90	-25026.50	5.52	-24990.84	2.67	-24950.64	4.37	-24934.52	1.23
-25150.52	-2.97	-25109.25	-0.21	-25071.18	-0.64	-25028.63	3.40	-24991.54	1.98	-24950.76	4.24	-24931.59	4.16
-25150.80	-3.25	-25108.10	0.94	-25070.42	0.11	-25029.30	2.72	-24992.96	0.56	-24950.32	4.69	-24929.63	6.12
-25149.42	-1.87	-25109.01	0.04	-25068.48	2.06	-25030.83	1.19	-24990.28	3.23	-24951.78	3.22	-24932.62	3.13
-25150.70	-3.15	-25111.18	-2.14	-25072.88	-2.35	-25029.10	2.93	-24992.14	1.37	-24952.16	2.84	-24928.73	7.02
-25151.37	-3.81	-25108.23	0.81	-25069.13	1.41	-25029.76	2.26	-24990.60	2.91	-24952.13	2.87	-24933.68	2.07
-25149.16	-1.61	-25110.21	-1.17	-25069.72	0.81	-25029.56	2.46	-24988.01	5.50	-24950.48	4.52	-24931.08	4.67
-25149.33	-1.77	-25110.83	-1.78	-25068.02	2.51	-25029.71	2.31	-24989.51	4.00	-24944.36	10.65	-24927.89	7.86
-25149.78	-2.23	-25110.47	-1.42	-25067.99	2.54	-25025.54	6.48	-24983.00	10.52	-24947.89	7.12	-24929.96	5.79
-25149.90	-2.35	-25111.09	-2.04	-25068.27	2.26	-25027.25	4.77	-24992.71	0.80	-24950.70	4.30	-24932.60	3.15
-25150.33	-2.78	-25110.66	-1.62	-25071.38	-0.84	-25030.07	1.95	-24985.32	8.19	-24947.94	7.06	-24928.12	7.63
-25149.70	-2.15	-25109.04	0.00	-25071.07	-0.53	-25030.25	1.78	-24989.45	4.06	-24952.68	2.33	-24928.89	6.86
-25149.85	-2.30	-25109.35	-0.31	-25067.68	2.86	-25030.03	2.00	-24980.10	13.41	-24944.81	10.19	-24932.30	3.45
-25149.42	-1.86	-25106.69	2.36	-25067.05	3.48	-25032.39	-0.36	-24990.58	2.94	-24948.08	6.92	-24931.48	4.27
-25149.39	-1.83	-25111.71	-2.67	-25070.32	0.21	-25023.85	8.17	-24987.40	6.11	-24946.66	8.34	-24931.54	4.21
-25152.00	-4.44	-25110.16	-1.11	-25070.29	0.25	-25022.90	9.13	-24980.49	13.02	-24952.29	2.72	-24931.16	4.59
-25150.58	-3.03	-25110.63	-1.59	-25072.58	-2.05	-25025.30	6.72	-24990.28	3.23	-24948.21	6.79	-24928.23	7.52
-25150.21	-2.66	-25107.76	1.29	-25067.32	3.21	-25011.33	20.70	-24989.62	3.90	-24946.21	8.79	-24929.42	6.33
-25149.30	-1.75	-25109.96	-0.92	-25067.94	2.59	-25025.91	6.12	-24988.64	4.87	-24947.19	7.82	-24932.99	2.76
-25150.77	-3.22	-25106.89	2.15	-25070.41	0.12	-25023.93	8.09	-24988.48	5.03	-24952.58	2.43	-24927.95	7.80
-25150.15	-2.60	-25109.75	-0.71	-25068.37	2.16	-25024.75	7.27	-24989.94	3.58	-24948.13	6.87	-24931.86	3.89

	-25149.49	-1.94	-25106.81	2.23	-25068.51	2.02	-25029.50	2.52	-24987.08	6.43	-24949.79	5.21	-24921.45	14.30
	-25148.55	-0.99	-25109.23	-0.19	-25070.77	-0.24	-25022.28	9.74	-24980.63	12.89	-24947.34	7.66	-24928.85	6.90
	-25151.41	-3.86	-25108.70	0.34	-25069.81	0.72	-25028.89	3.13	-24986.37	7.15	-24950.17	4.84	-24927.37	8.38
	-25150.58	-3.03	-25109.71	-0.67	-25069.52	1.01	-25032.36	-0.34	-24986.96	6.55	-24949.15	5.85	-24929.18	6.57
	-25149.93	-2.37	-25109.75	-0.71	-25069.84	0.69	-25029.69	2.33	-24989.63	3.89	-24952.26	2.74	-24931.57	4.18
	-25151.06	-3.51	-25111.54	-2.49	-25066.55	3.98	-25027.72	4.31	-24990.38	3.13	-24951.57	3.43	-24931.33	4.42
	-25148.67	-1.11	-25110.11	-1.07	-25065.36	5.17	-25030.98	1.04	-24986.75	6.77	-24947.54	7.47	-24931.01	4.73
	-25149.36	-1.80	-25109.37	-0.33	-25069.27	1.26	-25024.99	7.03	-24984.29	9.22	-24948.70	6.30	-24931.46	4.29
	-25150.57	-3.02	-25110.65	-1.61	-25068.88	1.65	-25030.23	1.79	-24989.35	4.17	-24946.03	8.97	-24931.95	3.80
	-25149.77	-2.22	-25103.84	5.20	-25062.33	8.20	-25031.25	0.78	-24989.23	4.29	-24948.19	6.81	-24926.21	9.54
	-25149.64	-2.09	-25110.97	-1.92	-25068.02	2.51	-25028.69	3.34	-24984.87	8.64	-24950.16	4.85	-24931.14	4.60
	-25149.81	-2.26	-25110.05	-1.00	-25068.48	2.06	-25027.22	4.80	-24987.98	5.53	-24948.30	6.70	-24929.31	6.44
	-25149.59	-2.04	-25107.88	1.16	-25066.66	3.87	-25027.42	4.60	-24990.72	2.80	-24949.23	5.77	-24928.45	7.30
	-25149.79	-2.24	-25110.79	-1.75	-25069.59	0.94	-25030.24	1.78	-24989.45	4.06	-24954.49	0.52	-24933.86	1.89
	-25150.53	-2.98	-25111.90	-2.86	-25070.56	-0.03	-25030.87	1.15	-24990.06	3.45	-24949.66	5.35	-24931.02	4.73
	-25149.95	-2.40	-25110.36	-1.32	-25067.76	2.77	-25026.00	6.02	-24990.09	3.43	-24951.21	3.80	-24928.47	7.27
	-25147.36	0.19	-25107.73	1.32	-25069.18	1.35			-24990.78	2.73	-24950.10	4.91	-24930.26	5.49
	-25150.09	-2.54	-25110.10	-1.06	-25067.67	2.86			-24990.48	3.04	-24950.11	4.89	-24927.71	8.04
	-25142.71	4.84	-25107.60	1.44	-25070.24	0.29			-24988.86	4.66	-24951.74	3.26	-24930.82	4.93
	-25150.08	-2.53	-25111.64	-2.59	-25069.62	0.92			-24988.62	4.90	-24954.26	0.75	-24927.10	8.65
	-25150.02	-2.46	-25106.57	2.47	-25062.89	7.64			-24987.26	6.26	-24951.76	3.24	-24927.68	8.07
	-25150.37	-2.82	-25109.64	-0.60	-25070.77	-0.24			-24989.98	3.54	-24950.44	4.57		
	-25149.61	-2.06	-25109.00	0.04	-25068.33	2.20			-24990.36	3.15	-24950.94	4.06		
	-25147.68	-0.12			-25067.40	3.14			-24987.82	5.69	-24947.27	7.73		
	-25150.78	-3.22			-25071.01	-0.48			-24992.13	1.38	-24951.81	3.20		
	-25148.33	-0.78							-24989.52	3.99				
	-25148.88	-1.33							-24989.94	3.57				
	-25149.93	-2.38							-24987.96	5.55				
	-25150.96	-3.41												
	-25151.11	-3.56												
	-25149.43	-1.88												
Average energies	-25149.83	-2.27	-25109.29	-0.25	-25068.66	1.88	-25028.20	3.82	-24988.87	4.64	-24949.42	5.59	-24929.42	6.33

Calculated ground state energies for the reactants and products used in the calculations for the solid solution energy

Material	Calculated ground state energy (eV)
CeO_2	-105.15
PuO_2	-102.15
ThO_2	-104.97
UO_2	-106.52
Al_2O_3	-157.59
Fe_2O_3	-152.34
TiO_2(rutile)	-120.74
$CaTiO_3$	-158.33
$ZrCaTi_2O_7$	-3148.26

Each ground state energy was multiplied to agree with the amount of atoms being replaced in the simulation cell and to agree with the amount of atoms in the cell
